# Borane‐Mediated Highly Secondary Selective Deoxyfluorination of Alcohols

**DOI:** 10.1002/anie.202418495

**Published:** 2025-01-09

**Authors:** Dominic R. Willcox, Nojus Cironis, Laura Winfrey, Sven Kirschner, Gary S. Nichol, Stephen P. Thomas, Michael J. Ingleson

**Affiliations:** ^1^ EaStCHEM School of Chemistry University of Edinburgh Edinburgh EH9 3FJ UK

**Keywords:** Deoxyfluorination, chemoselectivity, fluorine, boranes, chlorination

## Abstract

Organofluorine compounds are vital across multiple sectors, hence highly selective methods to install fluorine are of considerable importance. The deoxyfluorination of alcohols is a key approach to prepare organofluorine compounds, however, a highly secondary (2°)‐selective deoxyfluorination of alcohols has not been realized to date. Herein, we report that borane‐mediated deoxyfluorination results in high 2°‐selectivity in inter‐ and intra‐molecular competition reactions versus primary (1°), tertiary (3°) and even benzylic (Bn) alcohols. This is an operationally simple method using only commercial reagents (e.g., Et_3_N ⋅ 3HF) that starts from the alcohol which is converted to the *O*‐alkyl‐*N*‐H‐isourea in situ. The origin of the high 2°‐selectivity was elucidated to be due to the relative barriers to carbodiimide elimination from the *O*‐alkyl‐*N*‐(BR_2_)‐isoureas. As the selectivity controlling step does not involve fluoride, this borane‐mediated approach can be applied to other nucleophiles, as demonstrated by 2°‐selective deoxychlorination using HCl occurring in preference to substitution of 1° and Bn analogues. This borane‐mediated nucleophilic substitution therefore provides a new approach to circumvent the selectivity limitations inherent in classical S_N_2 and S_N_1 type reactions.

## Graphical abstract

Borane‐mediated deoxyfluorination of in situ generated *N*‐H‐*O*‐alkyl‐isoureas using Et_3_N ⋅ 3HF represents a novel approach to achieve a highly 2°‐alcohol‐selective substitution in preference to benzylic, 1° and 3° alcohol deoxyfluorination. The key to the 2° selectivity is the relative stability of the *N*‐BBN‐*O*‐alkyl‐isoureas towards elimination of the carbodiimide.

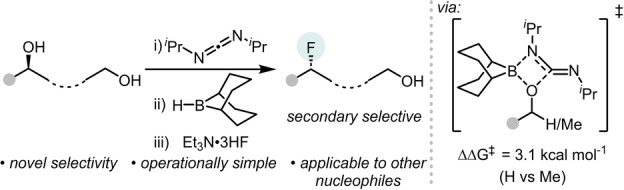

## Introduction

The incorporation of fluorine is widely used in the pharmaceutical, agrochemical and materials sectors to impart unique properties to functional compounds.[^1^, ^2^, ^3^, ^4^, ^5^] This is illustrated by >50 % of new small molecule drugs approved by the FDA between 2015 and 2020 containing at least one fluorine atom,^[6]^ with more in the pipeline.^[7]^ Deoxyfluorination offers a direct method for the synthesis of C−F bonds from widely‐available alcohols.[^8^, ^9^, ^10^] Multiple deoxyfluorination methods have been developed based on reagents containing reactive S−F (e.g., DAST, XtalFluor, pyFluor etc.)[^11^, ^12^, ^13^, ^14^, ^15^] or C−F units (e.g., PhenoFluor, CpFluor etc.),[^16^, ^17^] through activation of an alcohol, followed by nucleophilic fluorination. Significant effort has resulted in alcohol deoxyfluorination becoming a powerful and highly selective (for substitution over elimination) reaction.^[18]^ However, the chemoselective deoxyfluorination of polyols is less developed and is desirable as it would provide rapid access to organic molecules containing both fluoro and alcohol moieties. This functional group combination is present in multiple pharmaceuticals (e.g., Clevudine, Clofarabine, Paramethasone and Fludroxycortide).

**Scheme 1:**
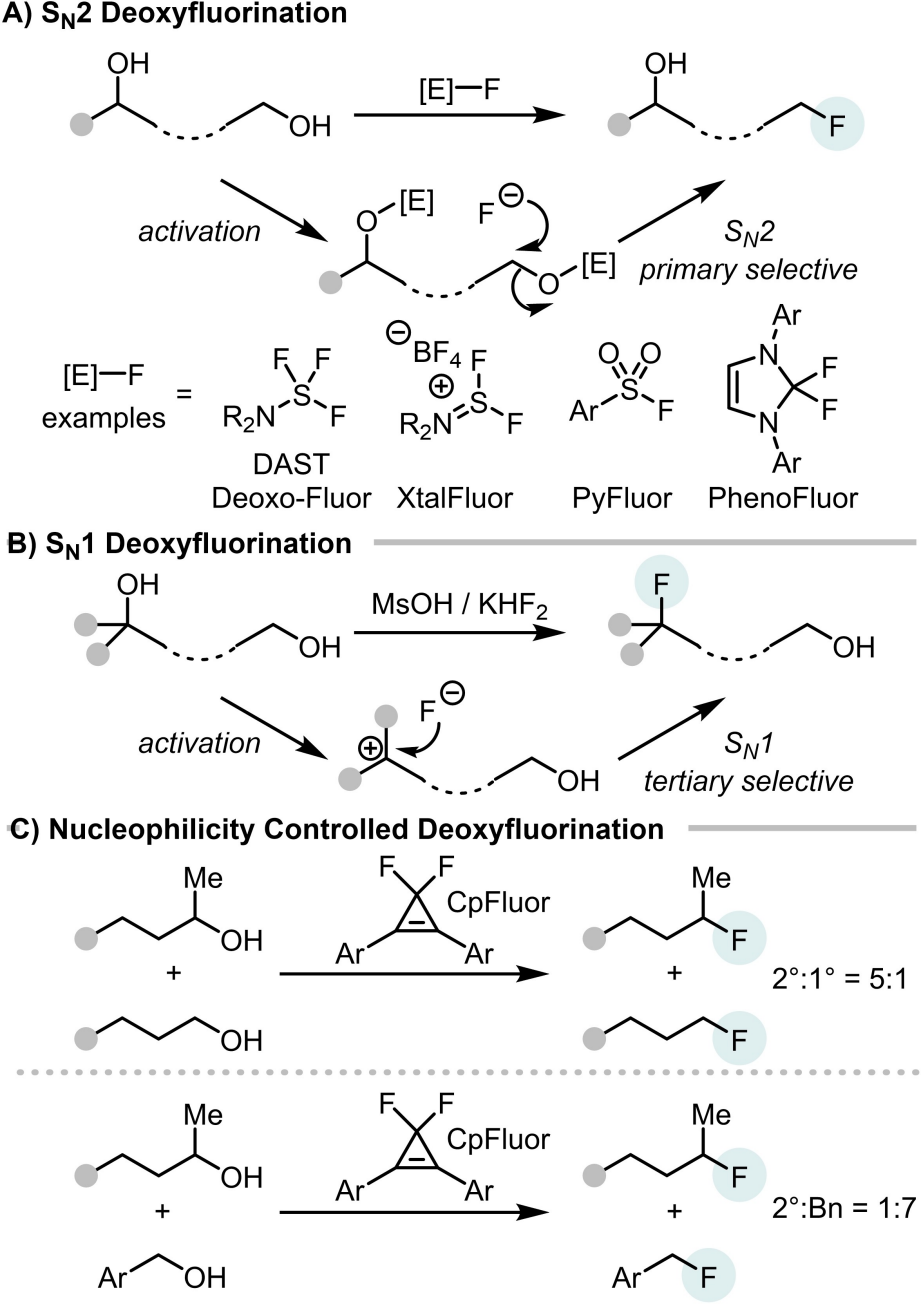
A−C=select previous work on selective alcohol deoxyfluorination.

Although, 1° and 3° selective deoxyfluorination of polyols is possible based on the intrinsic reactivity of activated alcohols in nucleophilic substitution (Scheme 1A and 1B),[^16^, ^19^] 2°‐alcohol selective deoxyfluorination remains a significant challenge. Assessment of common deoxyfluorination reagents showed that moderate 2° selectivity could be achieved using CpFluor, but only in cases where the electronics of the alcohol were already biased towards this selectivity (Scheme 1C).^[16]^ In other competition reactions the typical S_N_2 selectivity (reactivity: Bn>1°>2°) was observed using CpFluor (e.g. Scheme 1C). Therefore, developing novel approaches to realize 2°‐selective alcohol deoxyfluorination is still required. Furthermore, achieving a highly 2°‐alcohol selective deoxyfluorination using inexpensive and commercial reagents under operationally simple conditions would be a particularly attractive addition to the deoxyfluorination toolbox.

**Scheme 2:**
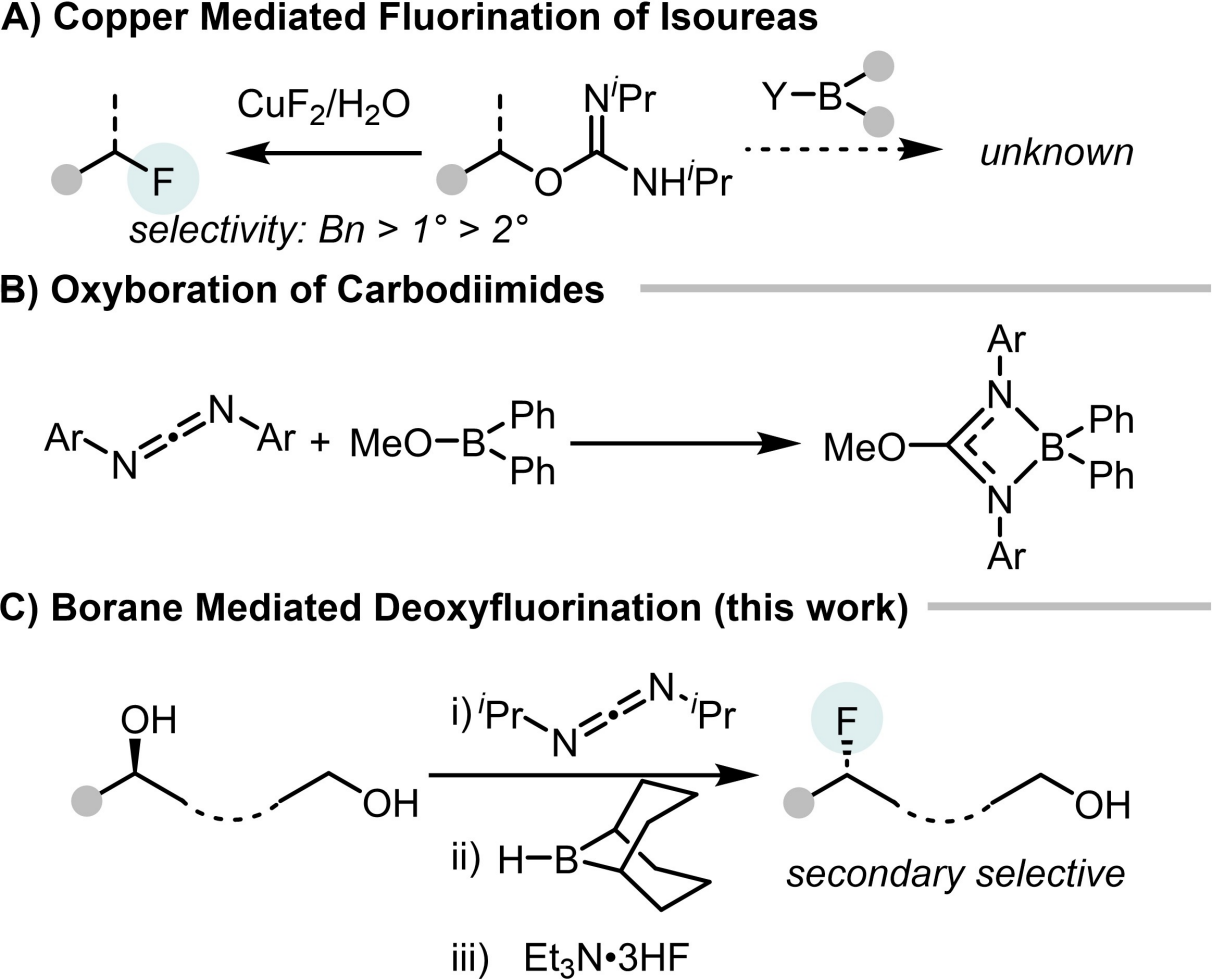
A) CuF_2_/H_2_O reacting with *O*‐alkyl‐isoureas by deoxyfluorination whereas the reactivity with boranes is not reported to our knowledge. B) previous work reporting a 1,2‐oxyboration of carbodiimides. C) Borane mediated secondary selective deoxyfluorination (this work).

The in situ conversion of alkyl‐alcohols into *O*‐alkyl‐isoureas by reaction with a carbodiimide is an established approach for activating alcohols towards S_N_2 reactivity.^[20]^ This approach has been applied recently for the one‐pot deoxyfluorination of alcohols using Lewis acidic metal fluorides, particularly CuF_2_/H_2_O (Scheme 2A left), with typical S_N_2 chemoselectivity observed (e.g., substitution of 1° alcohols favored over 2°).^[21]^ Interestingly, how an archetypal family of Lewis acids, the boranes, react with *O*‐alkyl‐isoureas has not been studied previously. The most relevant previous report is from1966 where it was concluded that carbodiimides underwent 1,2‐oxyboration with boranes such as Ph_2_BOMe to form the diarylborane‐coordinated isourea (Scheme 2B).^[22]^ This is relevant as this is the product from formal addition of Ph_2_B−Y to an *O*‐alkyl‐*N*‐H‐isourea followed by loss of HY.

Herein, we report our studies into understanding how boranes react with *O*‐alkyl‐isoureas and how this can be harnessed to control selectivity in the borane‐mediated deoxyfluorination of *O*‐alkyl‐isoureas using Et_3_N ⋅ 3HF (Scheme 2C). This resulted in the development of an operationally simple and highly 2°‐selective deoxyfluorination of polyols, with 2° alcohol deoxyfluorination proceeding in preference to the deoxyfluorination of 1°, 3° and even benzylic alcohols. Furthermore, the same protocol enabled the 2°‐selective deoxychlorination, indicating that this approach has potential as a widely applicable method for the chemoselective substitution of 2° alcohols.

## Results and Discussion

**Scheme 3:**
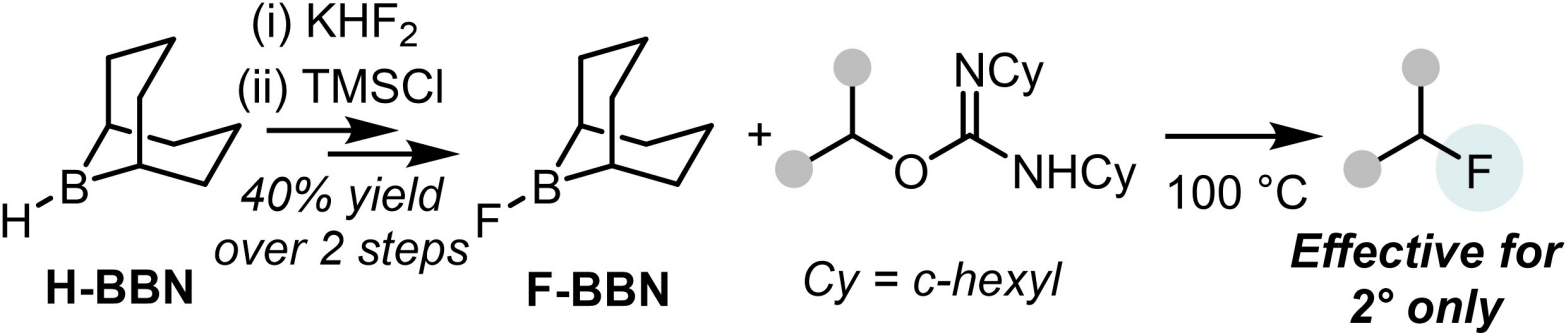
F‐BBN synthesis and use in the deoxyfluorination of *O*‐alkyl isoureas.

Building on recent studies into the use of less Lewis acidic organoboranes in fluorination by our group^[23]^ and others,[^24^, ^25^, ^26^, ^27^] we commenced an investigation into how Lewis acidic fluoroboranes react with *O*‐alkyl‐isoureas. Our studies utilized 9‐fluoro‐9‐borabicyclo(3.3.1)nonane (termed F‐BBN herein, Scheme 3).^[28]^ Initially, we developed a simple route to form F‐BBN from commercially available KHF_2_ and 9‐borabicyclo(3.3.1)nonane (H‐BBN, see SI).

**Scheme 4:**
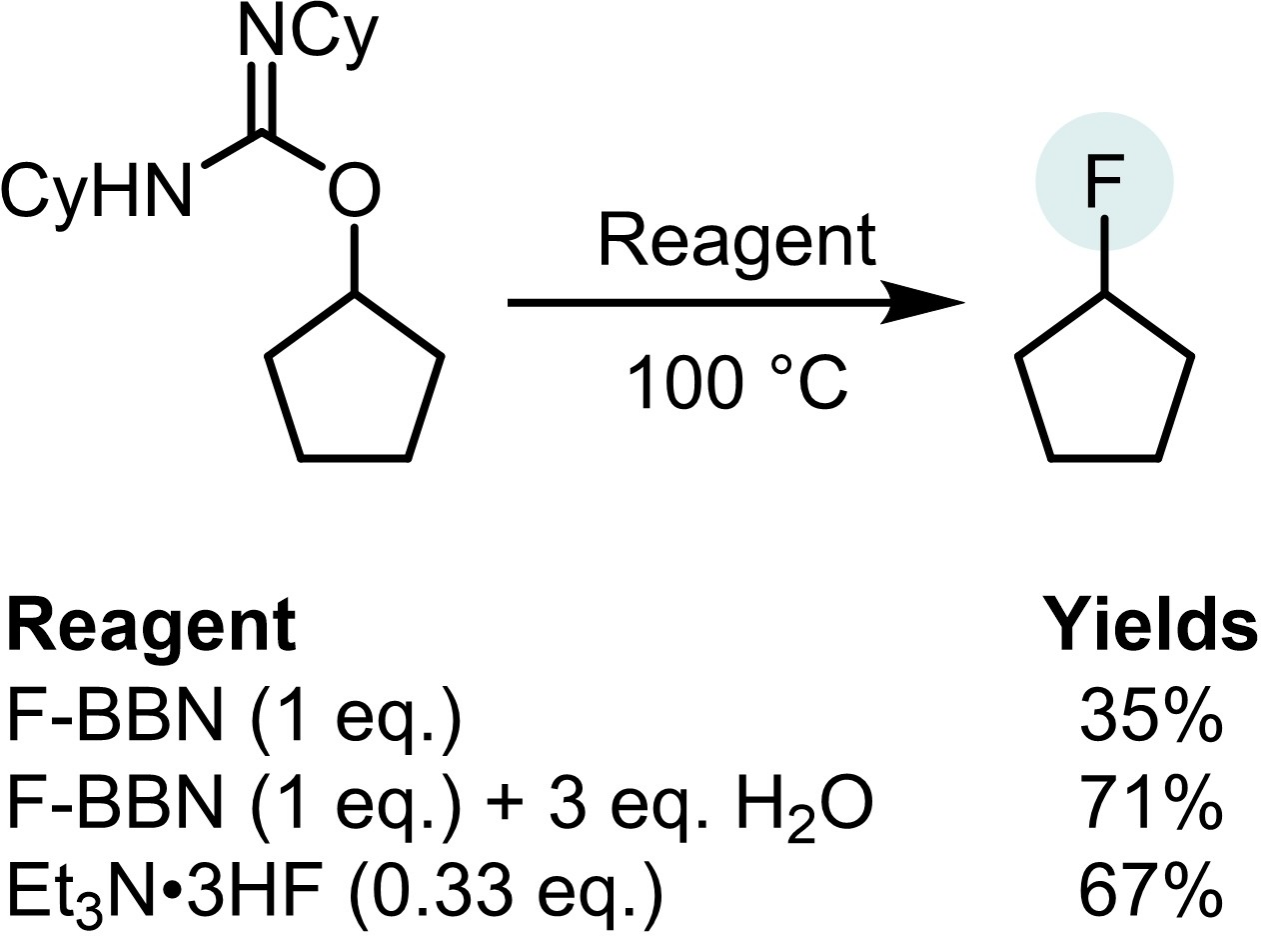
Deoxyfluorination using different fluoride sources. Yield by ^19^F NMR spectroscopy versus an internal standard.

Despite extensive studies, only 2° isoureas converted to any significant extent (>30 %) to the organofluorine compound using F‐BBN (by in situ quantitative ^19^F NMR spectroscopy). Yields of the alkylfluorides starting from 1° isoureas were <10 %, while an *O*‐benzyl‐isourea led to only ca. 15 % yield of the benzyl fluoride. This is in contrast to deoxyfluorinations using CuF_2_/H_2_O, which was effective for 1°, 2° and benzylic *O*‐alkyl‐isoureas.^[21]^ Notably, given the importance of water in the CuF_2_ methodology, the hydrolysis of F‐BBN with water was performed prior to the addition of an isourea. This led to a significant increase in the yield of the alkylfluoride (Scheme 4). This suggested a role for HF in deoxyfluorination under our conditions.^[29]^ The importance of HF was supported by the use of 0.33 equivalents of Et_3_N ⋅ 3HF in place of F‐BBN under otherwise identical conditions leading to a comparable deoxyfluorination outcome to that using F‐BBN/H_2_O (Scheme 4). Note, in contrast to more reactive HF sources,^[30]^ Et_3_N ⋅ 3HF does not effect the deoxyfluorination of alcohols directly.^[21]^ It should be noted that other fluoride sources, including CsF, KF, KHF_2_ and Bu_4_NF, led to minimal (<5 %) deoxyfluorination of primary and secondary alcohol derived isoureas under identical conditions, indicating a Brønsted acidic fluoride source is essential. An investigation into the scope of the deoxyfluorination of DCC (DCC=*N,N′‐*dicyclohexylcarbodiimide) derived *O*‐alkyl‐isoureas with Et_3_N ⋅ 3HF revealed it was applicable to 1°, 2° and benzylic isoureas (see section S3), with all deoxyfluorination reactions proceeding with moderate to good conversions and minimal (<5 %) elimination products observed.

**Scheme 5:**
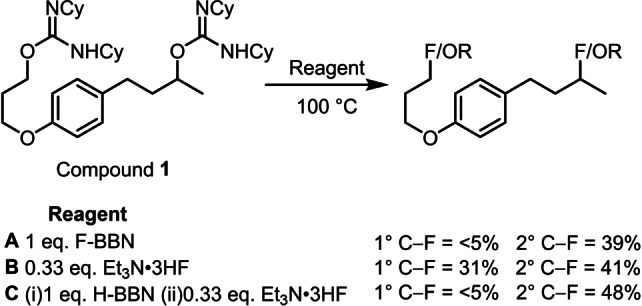
Outcomes using different BBN/HF sources in the deoxyfluorination of **1**. Yields by ^19^F NMR spectroscopy vs. internal standard. R=isourea or another substituent.

The effective deoxyfluorination of Bn, 1° and 2° isoureas using Et_3_N ⋅ 3HF was in contrast to results using F‐BBN which was only effective for 2° substrates. This indicated a role for the borane beyond acting as a fluoride or HF source. The 2°‐selectivity of F‐BBN was confirmed through an intramolecular competition reaction using bis‐isourea **1** (Scheme 5). The use of Et_3_N ⋅ 3HF displayed poor selectivity in the deoxyfluorination of **1**, whereas the use of F‐BBN led to highly 2°‐selective deoxyfluorination. It was hypothesized that F‐BBN was fulfilling two roles in this reaction: (i) acting in combination with the N−H unit as a source of HF; (ii) forming a BBN containing compound that controls the selectivity of the reaction. To test this hypothesis the use of H‐BBN and Et_3_N ⋅ 3HF in place of F‐BBN was explored, this was based on the hypothesis that both H‐BBN and F‐BBN will react with *O*‐alkyl‐isoureas in a similar manner, albeit evolving H_2_ and HF, respectively (Et_3_N ⋅ 3HF would then be required when using H‐BBN to provide a source of HF). Significantly, a highly 2°‐selective deoxyfluorination was achieved using H‐BBN and Et_3_N ⋅ 3HF in place of F‐BBN (Scheme 5).

Given the advantages of using H‐BBN/Et_3_N ⋅ 3HF (both are inexpensive, commercially available and easy to handle reagents)^[31]^ and the unprecedented highly 2°‐selective nature of this deoxyfluorination (2° : 1°>10 : 1 for **1**, Scheme 5), an optimization study was performed. This revealed that when used in combination with Et_3_N ⋅ 3HF, another dialkylborane, HB(cyclohexyl)_2_, was also effective. However, pinacolborane (HBpin) gave very low conversions to the deoxyfluorination product (Table S4). Next, it was found that the di*iso*propylcarbodiimide (DIC)‐derived bis‐isourea analogue of **1**, **1**‐^***i***^ **Pr**, gave marginally better outcomes than **1**, therefore DIC‐derived isoureas were used from hereon. Ultimately the optimization study led to a one‐pot deoxyfluorination process commencing directly from the alcohol, with the isourea made and transformed in situ. Furthermore, the optimized procedure is performed in air using only commercial reagents (H‐BBN as a THF solution), non‐purified solvents (CPME) and using standard laboratory glassware (see Supporting Information for full optimization details).

A series of intermolecular competition reactions were carried out to assess the selectivity profile under the optimized conditions, both in the presence and absence of H‐BBN (Table 1). The competition between 3‐phenyl‐1‐propanol (**2 a**) and 4‐phenyl‐2‐butanol (**2 b**) gave excellent selectivity for 2° deoxyfluorination when borane‐mediated (15 : 1, 2° : 1°, **3 b** : **3 a**), which is in contrast to the poor selectivity observed in the absence of H‐BBN (1.4 : 1, **3 b** : **3 a**). Note, the 2° selectivity using our H‐BBN mediated methodology is significantly higher than that using Cp‐Fluor which gave 4.8 : 1 2° : 1° selectivity.^[16]^ Even starting from an activated (more electrophilic) benzylic alcohol, 4‐nitrobenzyl alcohol (**2 c**), the selectivity towards the 2°‐alcohol deoxyfluorination of 4‐phenyl‐2‐butanol **2 b**, was maintained when using H‐BBN (8 : 1, 2° : 1° Bn, **3 b** : **3 c**). Importantly, there was minimal selectivity in the borane‐free reaction (1.4 : 1, 2° : 1° Bn, **3 b** : **3 c**). A similar 2°‐selective outcome was observed when reacting 2° alkyl alcohol **2 b** in the presence of a non‐activated benzylic alcohol, 1‐naphthyl methanol (**2 d**) with H‐BBN (4 : 1, 2° : 1° Bn, **3 b** : **3 d**). Without H‐BBN there was poor selectivity (1.6 : 1, 2° : 1° Bn). This is in contrast to deoxyfluorination reactions using Cp‐Fluor (Scheme 1C right),^[16]^ again indicating higher selectivity using this borane‐mediated process compared to reactions using Cp‐Fluor. Comparing a 1° and a 2° benzyl alcohol, **2 d** and 1‐phenylethanol **2 e**, respectively, the preference for 2° alcohol deoxyfluorination was still observed using H‐BBN/Et_3_N ⋅ 3HF (5 : 1, 2° Bn : 1° Bn, **3 e** : **3 d**), with poor selectivity observed again in the borane‐free reaction (1.6 : 1, 2° Bn : 1° Bn, **3 e** : **3 d**).

**Table 1:**
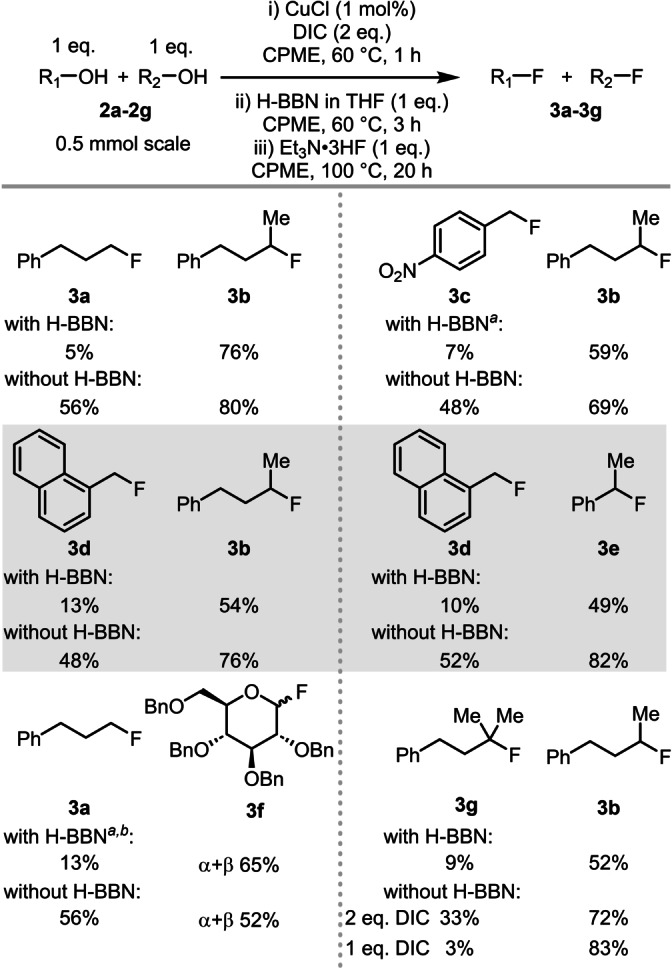
Intermolecular competition reactions.

[*a*] Filtration through an alumina plug prior to addition of Et_3_ N ⋅ 3HF (see Supporting Information for details). [*b*] The starting alcohol was a mixture of alpha and beta stereoisomers. % values are yields by ^19^F NMR spectroscopy versus an internal standard.

The selectivity also was assessed when the 2° alcohol can form an oxocarbenium cation. This is important as the fluorination of sugars at the anomeric position is routinely carried out, most often using highly reactive S−F based deoxyfluorination reagents.^[32]^ The competition was carried out between *O*‐benzyl‐protected **2 f** and 3‐phenyl‐1‐propanol **2 a**, which revealed high selectivity towards anomeric deoxyfluorination when borane‐mediated (5 : 1, anomeric:1°, **3 f** : **3 a**), but effectively no selectivity without H‐BBN (1 : 1.1, anomeric : 1°). Next, we assessed how a 3° alcohol performed in a competition reaction by using 4‐phenyl‐2‐butanol (**2 b**) and 4‐phenyl‐2‐methyl‐2‐butanol (**2 g**). The 2°‐selectivity remained high under the standard conditions, being 6 : 1 2° : 3° (**3 b** : **3 g**) in the presence of H‐BBN but again being poorly selective in the absence of H‐BBN (2 : 1, 2° : 3°, **3 b** : **3 g**). Cognizant that *O*‐alkyl‐isourea formation from carbodiimides is slower starting from 3° alcohols relative to 2°,^[33]^ conditions using only 1 eq. DIC (relative to 2 eq. alcohol) and no H‐BBN was explored as this would lead to conversion of only the 2° alcohol to the isourea. Under these conditions, deoxyfluorination using Et_3_N ⋅ 3HF was highly 2° selective in the absence of H‐BBN.

Given that excellent 2° selectivity can be achieved in competition reactions with both 1° and 3° alcohols, a three‐way competition between three alcohols: 4‐phenyl‐2‐methyl‐2‐butanol **2 g**, 4‐phenyl‐2‐butanol **2 b**, and 3‐phenyl‐1‐propanol **2 a** was explored (Table 2). The standard borane‐mediated deoxyfluorination conditions gave good selectivity for 2° fluorination over both 1° and 3° (4 : 1 : 0, 2° : 1° : 3°), significantly better than the reaction without H‐BBN (1.2 : 1 : 0, 2° : 1° : 3°).^[34]^ When this reaction was repeated with an extra equivalent of H‐BBN the reaction now proceeded with excellent 2° selectivity (19 : 1 : 0, 2° : 1° : 3°). This was rationalized by the equivalent of O−H remaining in this reaction needing to be accounted for, this requires an extra equivalent of H‐BBN due to formation of the 3° RO‐BBN species from reaction of the 3° ROH and H‐BBN.

**Table 2:**
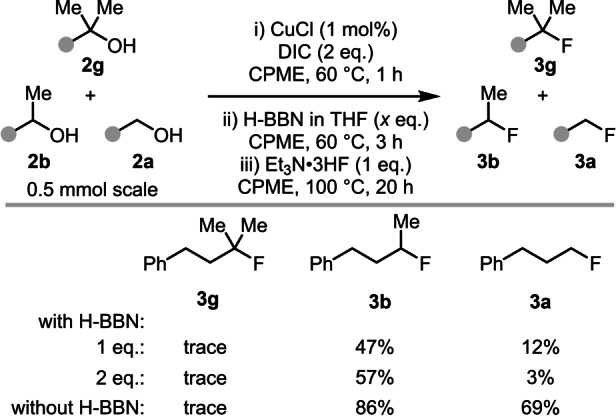
Outcomes from three‐way competition reactions.

% values are yields are by ^19^F NMR spectroscopy versus an internal standard.

Next a series of intramolecular competition reactions were carried out on diols containing both 1° and 2° alcohols (Table 3). The reaction of diol **4 a** gave **5 a** (63 %) with high selectivity (16 : 1, 2° : 1° deoxyfluorination). Notably, this selectivity is significantly higher than that reported for the same diol using CpFluor (5 : 1 2° : 1°), with a comparable yield.^[16]^ Using a steroid structure, 5β‐cholane‐3α,24‐diol **4 b**, the reaction proceeded to give **5 b** in moderate yield and with good selectivity and (42 %; 4 : 1, 2° : 1°). A diol derived from the NSAID loxoprofen **4 c**, gave **5 c** in moderate yield with high selectivity. The formation of fluoro‐alcohol **5 c** is notable as studies using DAST on related 2‐alkyl‐cyclopentanols led to formation of quaternary alkylfluorides as the major product, through carbocation mediated rearrangement.^[35]^ For the more challenging diol **4 d** containing a reducible alkene unit, deoxyfluorination using our standard conditions gave **5 d** in moderate yield with excellent 2° selectivity. However, it should be noted that more flexible diol substrates, e.g., **4 e**, proved not to be amenable giving minimal deoxyfluorination product **5 e** (<5 %). In this case, this is presumably due to intramolecular cyclisation as observed in several related systems.[^36^, ^37^]

**Table 3:**
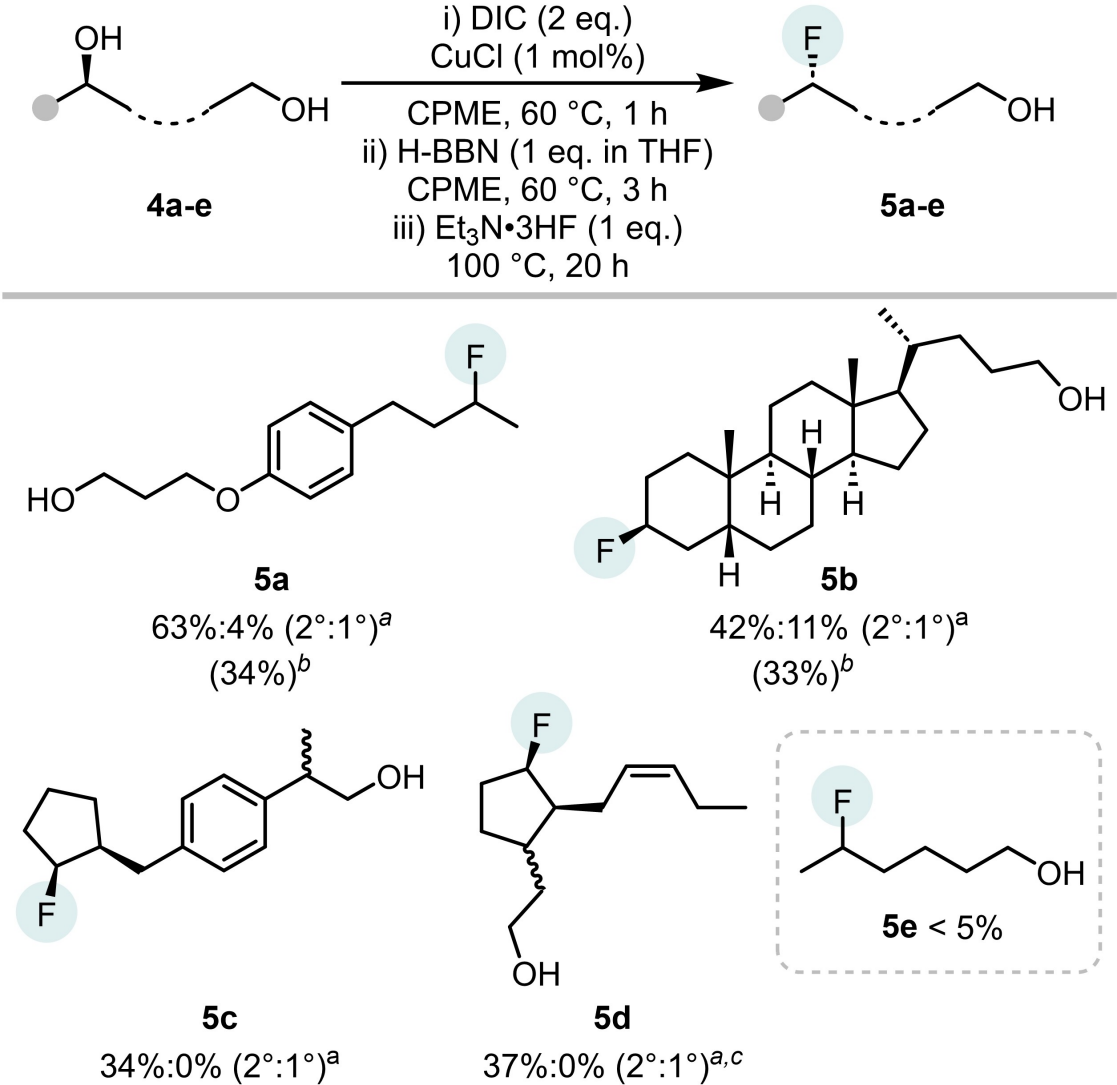
Applicability and selectivity towards diols.

[*a*] Yield by ^19^F NMR spectroscopy using 1,3,5‐trifluorobenzene and C_6_F_6_ as internal standards. [*b*] Isolated yield. [*c*] From the isolated bis‐isourea and run‐in toluene.

### Mechanistic Studies

**Scheme 6:**
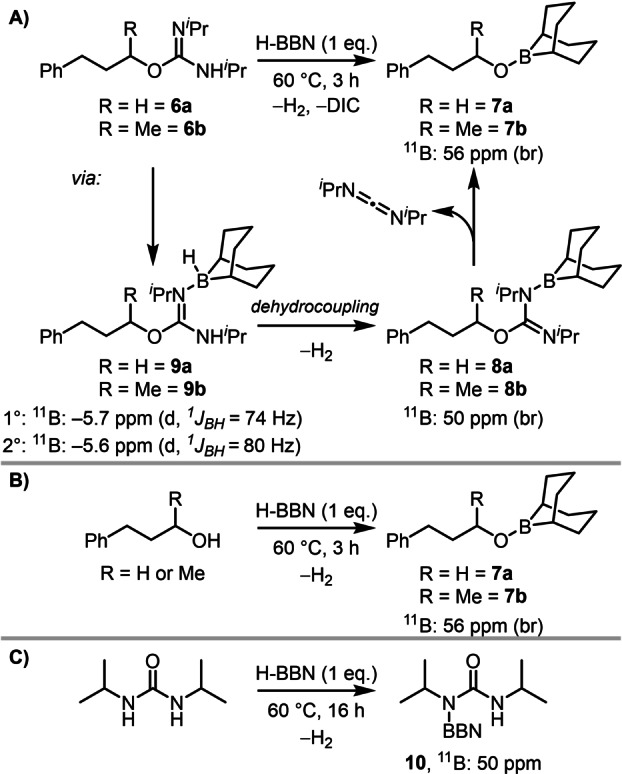
A) Outcomes from the reaction of *O*‐alkyl‐isoureas with H‐BBN. B) Independent synthesis of **7 a** and **7 b**. C) Reaction of *N*,*N′*‐diisopropylurea with H‐BBN.

Given the high 2°‐selectivity observed, initial mechanistic work sought to determine if these deoxyfluorination reactions using Et_3_N ⋅ 3HF and H‐BBN/Et_3_N ⋅ 3HF proceeded via an S_N_2 process. Complete inversion of the isourea derived from the enantiopure secondary alcohol (*R*)‐4‐phenylbutan‐2‐ol (*R*‐**2 b**) occurred during deoxyfluorination under both conditions consistent with an S_N_2 mechanism. The key question thus became how does the addition of H‐BBN enable 2°‐selectivity in a deoxyfluorination that proceeds via an S_N_2 process? This was addressed by analyzing stoichiometric reactions starting from the isolated 1° and 2° *O*‐alkyl‐isoureas **6 a** and **6 b**, respectively, by in situ NMR spectroscopy (Scheme 6A). Combining the isourea derivatives **6 a** and **6 b** separately with 1 eq. of H‐BBN at ambient temperature revealed multiple new boron species, including a minor doublet at δ_11B_=−5.7/−5.6 (starting from **6 a**/**6 b** respectively), consistent with formation of a Lewis adduct of H‐BBN. Broad resonances at δ_11B_=50 and 56 ppm also were observed. On heating to 60 °C both reactions led to clean conversion to compounds with δ_11B_=56. The ^11^B chemical shift at 56 ppm is consistent with RO‐BBN species,^[38]^ in this case these would be the RO‐BBN derivatives **7 a** and **7 b** (Scheme 6A). Compounds **7 a** and **7 b** were formed independently from combining the appropriate alcohol with H‐BBN, confirming their identity (Scheme 6B). The mass balance for the reaction of isoureas **6 a**/**6 b** and H‐BBN to form **7 a**/**7 b** would be the carbodiimide DIC and H_2_, with H_2_ and DIC both observed to form, with DIC increasing in intensity concomitant with the RO‐BBN species **7 a**/**7 b** (by multinuclear NMR spectroscopy). The intermediate observed at δ_11B_ 50 was assigned to the dehydrocoupling products **8 a** and **8 b**, termed BBN‐isoureas, by comparison with independently prepared analogues (e.g., compound **10**, Scheme 6C, and compound **12**‐see below). Therefore, the two *O*‐alkyl‐isoureas **6 a**/**6 b** react with H‐BBN at 60 °C by initial Lewis adduct formation (**9 a**/**9 b**), then loss of H_2_ to form **8 a**/**8 b** and finally elimination of DIC to give the alkoxyboranes **7 a**/**7 b**. This process is thermodynamically favored as the reverse reaction, oxyboration, the 1,2‐addition of MeO‐BBN to DIC, led to no observable reaction under a range of conditions. Furthermore, identical outcomes were observed when heating DCC‐derived *O*‐alkyl isoureas and H‐BBN (in this case forming DCC alongside the RO‐BBN species).

**Scheme 7:**
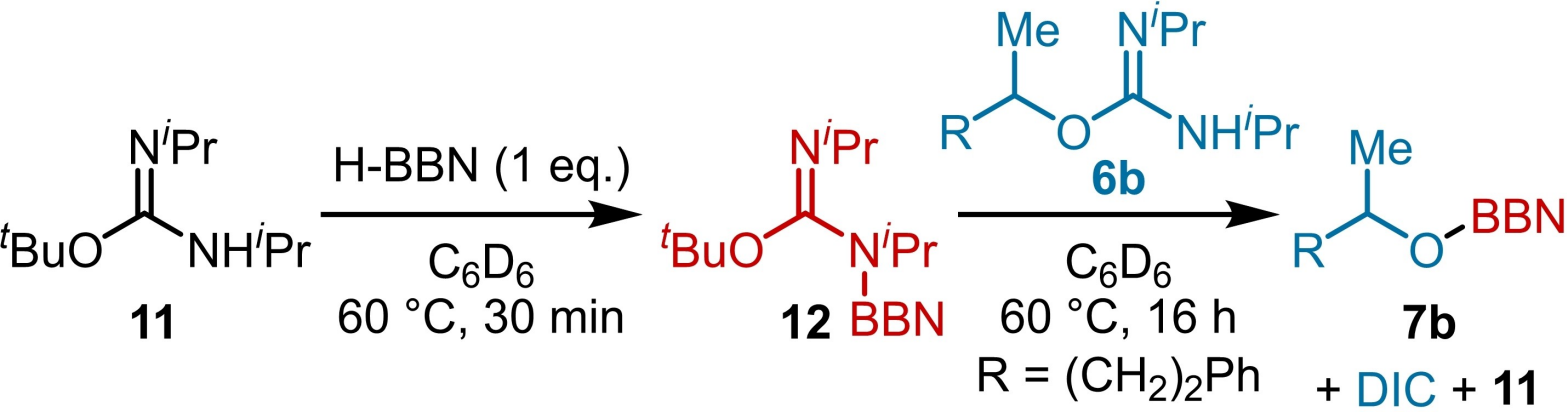
Exchange between N−H/N‐BBN O‐alkyl isoureas prior to DIC elimination.

During the in situ monitoring of the stoichiometric reactions it was observed that the elimination of DIC from 1° alcohol derivative **8 a** was faster than the elimination of DIC from the 2° alcohol derivative **8 b**. In addition, control reactions showed that the products from DIC elimination, the RO‐BBN compounds, do not undergo deoxyfluorination with Et_3_N ⋅ 3HF under the optimized conditions. This is significant as under the reaction conditions for 2°‐selective deoxyfluorination, H‐BBN is used as a limiting reagent (1 equivalent of H‐BBN is used relative to 2 equivalents of *O*‐alkyl isourea). Therefore, the selective elimination of DIC from a 1° BBN‐isourea to form a 1° RO‐BBN species (e.g., DIC elimination occurring only from **8 a** in mixtures of **8 a**/**8 b**) would account for the observed 2° selectivity, as only the 1° site is deactivated towards S_N_2. For this to be a feasible explanation for the observed high 2° selectivity it requires either (or a combination of): (i) H‐BBN to react selectively and irreversibly with the 1° isourea (e.g. a 1 : 1 mixture of isoureas **6 a**/**6 b** forms **8 a** selectively on addition of 1 equiv. of H‐BBN); or (ii) 1 equiv. H‐BBN to react with 1 : 1 **6 a**/**6 b** mixtures to give both the 1° and 2° BBN‐isoureas (e.g. **8 a** and **8 b** are both formed) but these are in a rapid exchange (relative to DIC elimination) with the remaining *N*‐H‐*O*‐alkyl‐isoureas (e.g. **6 a**/**6 b**). In the latter scenario the relative barriers to DIC elimination from the 1° and 2° BBN‐isoureas (e.g., **8 a** *vs* **8 b**) will dictate the selectivity of the deoxyfluorination reaction. A third possibility was discounted based on the absence of any reaction between **7 b** and **6 a**. Stoichiometric reactions revealed that (ii) is key for the high 2° selectivity observed. Specifically, it was found that under the optimized conditions, while the formation of the 1° BBN‐isourea is favored kinetically over the 2° congener, these BBN‐isoureas are in equilibrium with the *N*‐H‐*O*‐alkyl isoureas. This equilibrium was confirmed by using 3° isourea derivatives **11** and **12**. The BBN‐isourea **12** was ideal for this as it does not undergo DIC elimination at 60 °C (the conditions used to form the BBN‐isoureas by dehydrocoupling). Combining BBN‐isourea **12** and 2° *N*‐H‐isourea **6 b** led to selective formation of the elimination products, DIC and the RO‐BBN species **7 b** on heating, with the *N*−H isourea **11** formed as the expected by‐product (Scheme 7). This confirms that BBN/H exchange occurs between *N*−H and *N*−BBN isoureas at 60 °C.

**Scheme 8:**
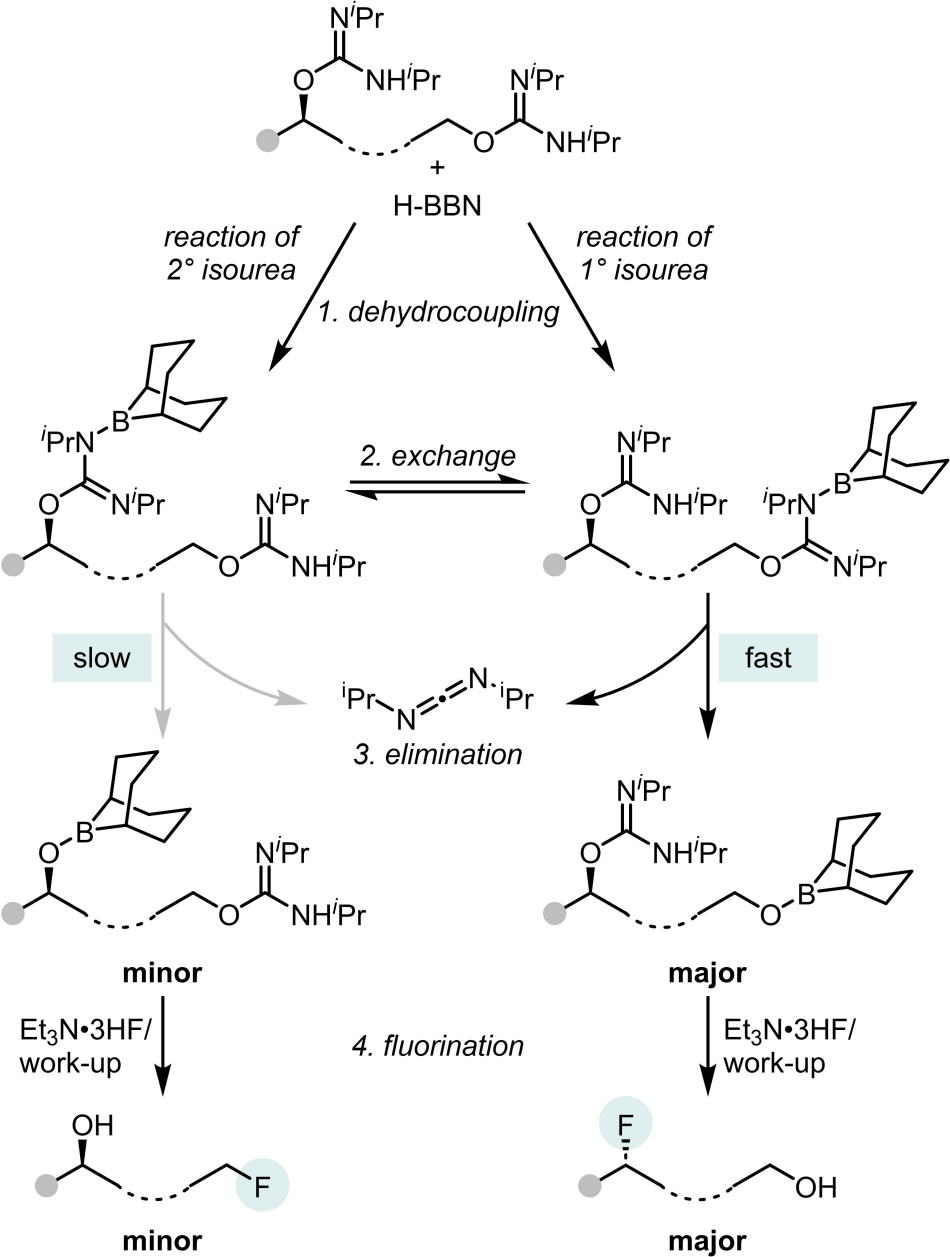
Proposed origin of the high 2° selectivity in borane mediated deoxyfluorination.

A model that explains the borane mediated 2°‐selective deoxyfluorination consistent with the data is shown in Scheme 8. This involves formation of a mixture of *N*‐H/*N*‐BBN *O*‐alkyl isoureas (step 1) that are in equilibrium (step 2), with DIC elimination (step 3) occurring preferentially for the 1° *N*‐BBN‐*O*‐alkyl derivative (right‐hand side, Scheme 8). The selectivity in the elimination step is presumably steric in origin given the greater barrier to DIC elimination observed for the 3° derivative **12**.

The final question was how does fluorination (step 4 in Scheme 8) proceed? Analyzing the in situ NMR spectra revealed the growth of significant resonances at δ_11B_=+13 and δ_19F_=−150. These corresponded to the borate anion [F_2_‐BBN]^−^, as confirmed by independent synthesis. This anion potentially forms from the addition of excess Et_3_N ⋅ 3HF to the RO‐BBN species.^[39]^ However, control reactions using *O*‐(2‐(4‐phenyl)butyl)‐*N*,*N′*‐di*iso*propyl isourea (**6 b**) showed that [F_2_‐BBN]^−^ is not the nucleophilic fluoride source (Figures S82–85). A relatively high fluoride affinity is calculated for F‐BBN (the “conjugate” Lewis acid of [F_2_‐BBN]^−^),^[40]^ which confirms that [F_2_‐BBN]^−^ is a relatively poorly nucleophilic source of fluoride.^[23]^ The formation of [F_2_‐BBN]^−^ during the deoxyfluorination therefore acts as a fluoride trap, explaining the requirement for excess fluoride (i.e. 1 equivalent of Et_3_N ⋅ 3HF which equates to three equivalents of fluoride) to obtain satisfactory deoxyfluorination yields. In contrast, in the absence of borane, deoxyfluorination yields of >60 % can be achieved using just 0.33 equiv. Et_3_N ⋅ 3HF (e.g., Scheme 4), with this reaction also facilitated by Et_3_N ⋅ 3HF being highly soluble in low polarity solvents.

**Scheme 9:**
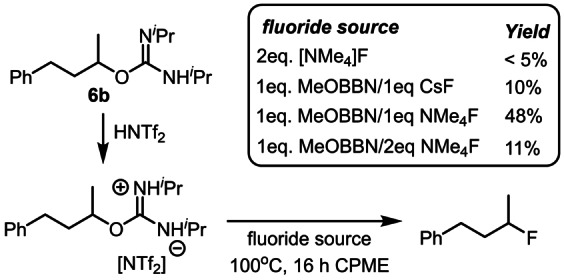
Stepwise protonation/fluorination studies.

Given the requirement for HF sources, protonation of the *O*‐alkyl‐isoureas was proposed as essential for enabling deoxyfluorination. To assess this **6 b** was protonated using 1 eq. triflimidic acid, HNTf_2_, followed by addition of 2 eq. [NMe_4_]F. However, this resulted in <5 % deoxyfluorination (Scheme 9), with a biphasic mixture observed even at 100 °C. As boranes can function as fluoride phase transfer agents,^[23]^ the addition of MeO‐BBN and a fluoride salt was explored. While MeO‐BBN/CsF gave poor results, the addition of 1 : 1 MeO‐BBN/[NMe_4_]F to protonated **6 b** resulted in 48 % deoxyfluorination (Scheme 9). In this reaction [MeO(F)‐BBN]^−^ is presumably the nucleophilic fluoride source. This reaction also confirms that iso‐urea protonation is important under these conditions as no deoxyfluorination is observed in the absence of a Brønsted acid. Notably, using a 1 : 2 ratio of MeO‐BBN/[NMe_4_]F led to only 11 % deoxyfluorination (Scheme 9). ^11^B NMR spectroscopy revealed that the major borate present in this reaction was [F_2_BBN]^−^ (triplet at 10.6 ppm) with a minor second borate also observed (at +8.5 ppm) and assigned as [MeO(F)‐BBN]^−^ (Figure S90). These results indicate that [RO(F)‐BBN]^−^ is a more effective source of nucleophilic fluoride than [F_2_‐BBN]^−^ consistent with MeO‐BBN having a lower fluoride affinity than F‐BBN.^[41]^ With [F_2_‐BBN]^−^ observed as the major borate in solution under the optimized conditions the use of MeO‐BBN as an additive was explored. It was hypothesized that additional RO‐BBN would favor formation of [RO(F)‐BBN]^−^ thereby reducing the amount of [F_2_‐BBN]^−^ formed and thus improving the deoxyfluorination outcome. Indeed, the use of 2 eq. of MeO‐BBN alongside 1 eq. of H‐BBN led to the deoxyfluorination of **6 b** in higher yield than when using just 1 eq. H‐BBN (both run with 1 eq. Et_3_N ⋅ 3HF, see Table S16). Therefore, in this borane mediated deoxyfluorination the active fluoride nucleophile can be the remaining 0.33 eq. Et_3_N ⋅ 3HF or [RO(F)‐BBN]^−^. Finally, we would note that regardless of the identity of the fluoride nucleophile it does not play a significant role in the selectivity determining step if our model is correct.

### Computational Studies

**Figure 1:**
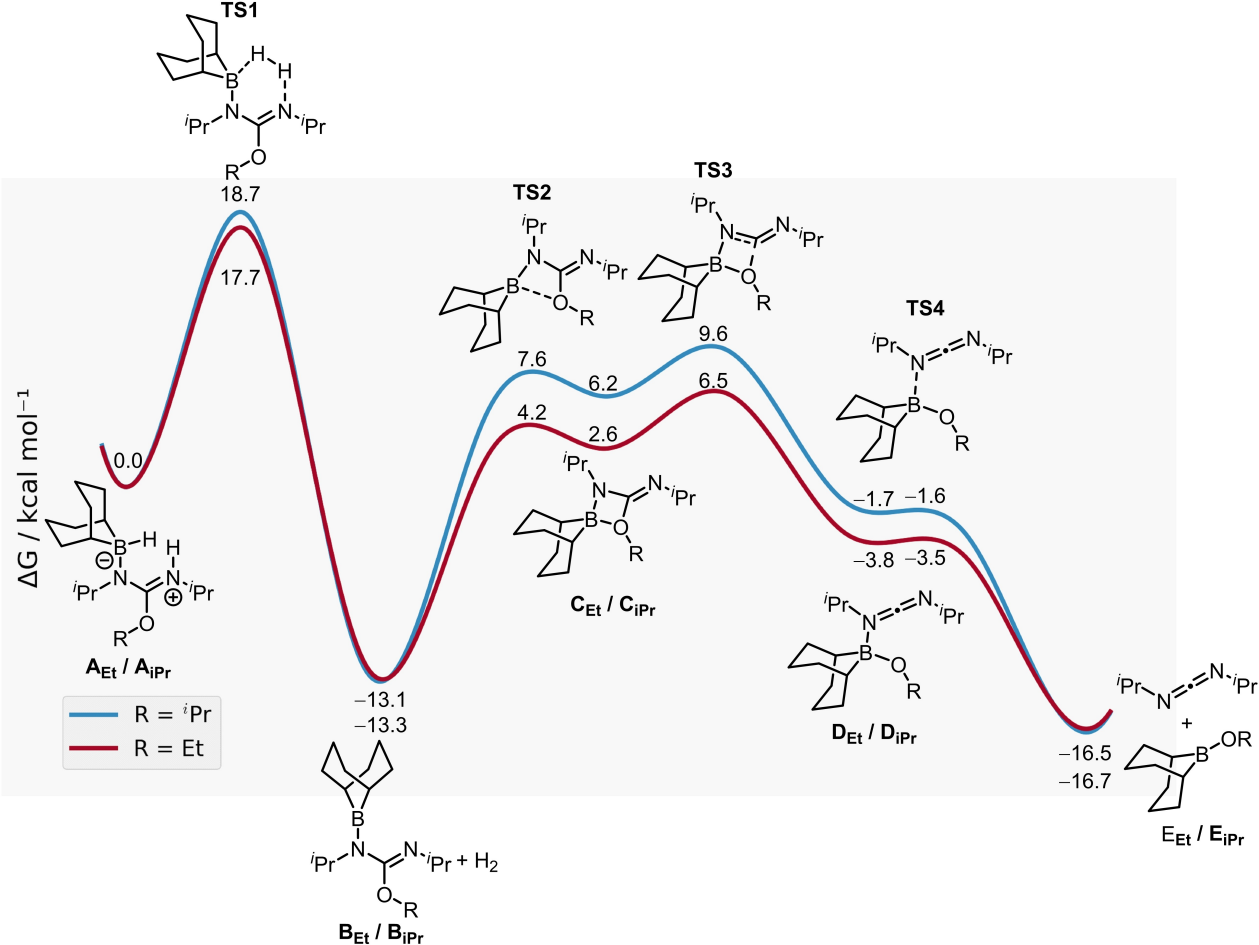
Calculated dehydrocoupling and elimination steps from H‐BBN/isourea Lewis adducts.

Our proposal for the origin of the high 2°‐selectivity was assessed by DFT calculations at the MN15/Def2‐TZVP(SMD‐toluene) level of theory. To accurately assess the steric effects for the ethoxy and *iso*propoxy groups a workflow to examine ground‐ and transition‐state conformers was developed (see Supporting Information for details). For each state the conformers were generated using CREST at the GFN2‐xTB(ALPB‐toluene) level of theory,[^42^, ^43^] and ranked at the M06‐2X(D3)/Def2‐SVP(SMD‐toluene) level of theory. The Lewis adducts **A_Et_** and **A_iPr_** (Figure 1) both form in an exergonic step (ΔG=−6.3 and −5.8 kcal mol^−1^, respectively) from H‐BBN and the respective *O*‐alkyl‐isourea. Starting from **A_Et_** and **A_iPr_** there is minimal difference between the dehydrocoupling barriers for these two congeners (Figure 1 **TS** **1** ΔΔG^≠^=1.0 kcal mol^−1^) which lose H_2_ to give the *N*‐BBN‐isoureas **B_Et_** and **B_iPr_**. While this step has a slightly lower barrier for the ethoxy derivative it is marginally more exergonic for the ^*i*^Pr derivative. The elimination of DIC then was found to occur through a 4‐membered transition state (**TS** **3**) with elimination and then dissociation of DIC being exergonic for both systems by 3.4 kcal mol^−1^, consistent with the reverse reaction (the oxyboration of DIC) not being observed by NMR spectroscopy. Notably, there is a significant difference in the barrier for the elimination step (**TS** **3** ΔΔG^≠^=3.3 kcal mol^−1^), with DIC elimination from the 1° BBN‐isourea **B_Et_** having the lower overall barrier. Analysis of **TS** **3** revealed an earlier transition state for the ^*i*^Pr congener (**TS** **3** (^i^PrN)_2_C−O=1.815 Å for R=^i^Pr vs. 1.842 Å for R=Et) and a greater distortion energy for the ^i^Pr congener (based on activation‐strain analysis). The latter is consistent with greater steric crowding in **TS** **3** leading to the higher barrier for DIC elimination observed for the 2° systems.

**Figure 2:**
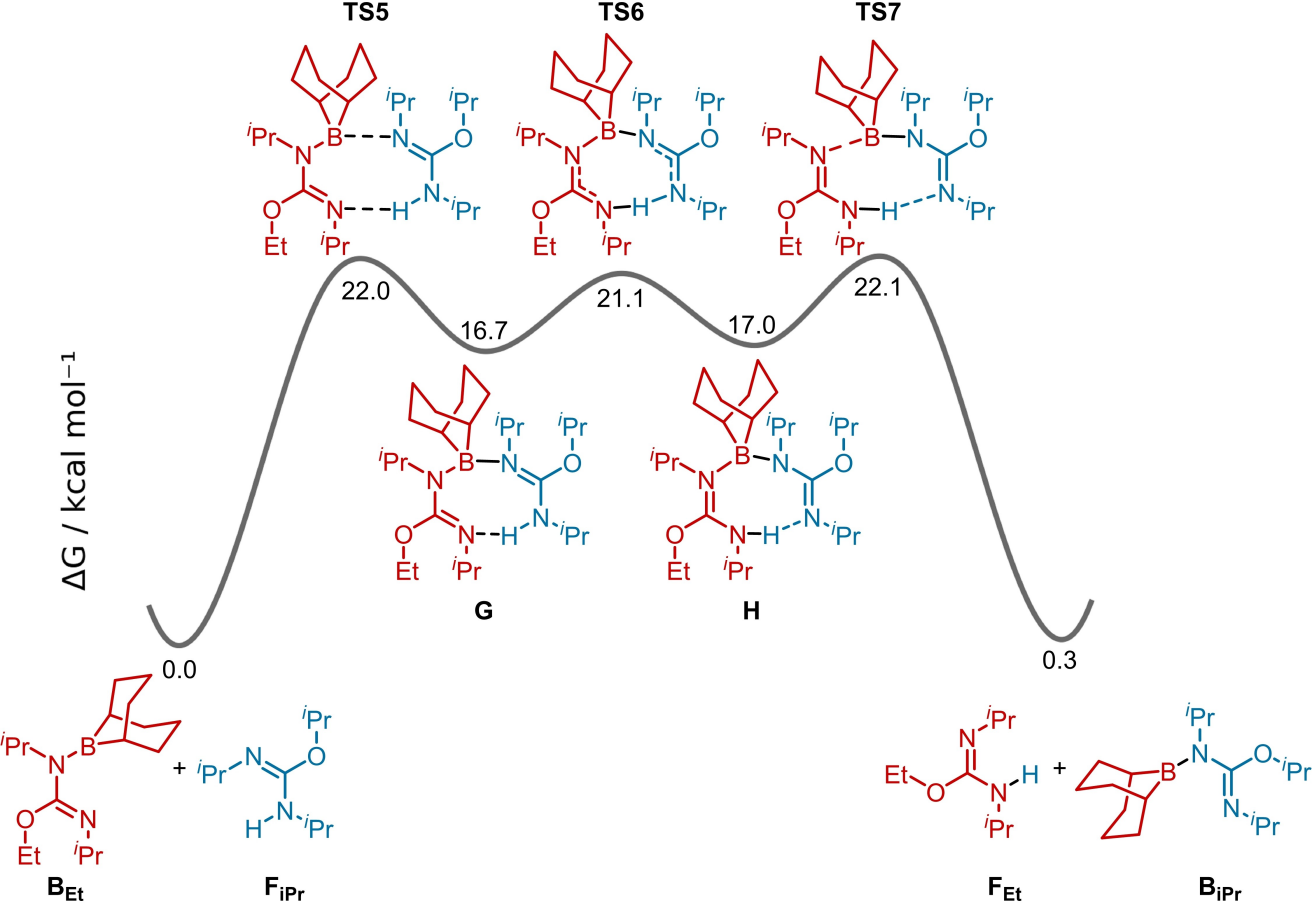
H/BBN exchange between 1° and 2° *O*‐alkyl isoureas.

The barrier to H/BBN exchange was next assessed for 1°/2° mixtures. The mechanism was explored starting from the *O*‐ethyl‐*N*‐BBN‐isourea **B_Et_** and *O*‐*iso*propyl‐N−H‐isourea **C_iPr_** (Figure 2). The exchange was found to be effectively thermoneutral proceeding via 8‐membered transition states involving concerted H/BBN transfer. Notably, the barrier to H/BBN exchange was found to be lower than the barrier to DIC elimination from the 2° *N*‐BBN‐isourea **B_iPr_** (a 21.8 kcal mol^−1^ barrier for exchange starting from **C_Et_** and **B_iPr_** Vs. 22.9 kcal mol^−1^ barrier for DIC elimination from **B_iPr_**). This indicates that H/BBN exchange in mixed 1°/2° systems can occur more rapidly than DIC elimination from the 2° *N*‐BBN‐isourea (e.g., from **B_iPr_**). This is consistent with our observations in the competition reactions where the 1° alkoxy‐BBN was formed predominantly under conditions where H‐BBN was the limiting reagent. The high 2° selectivity in this borane‐mediated deoxyfluorination therefore occurs due to the relative 1° versus 2° DIC elimination barriers combined with the fact the BBN‐isoureas are in rapid exchange (relative to DIC elimination from the 2° BBN‐isourea) with the N−H isoureas. This equilibrium enables replenishment of the 1° BBN‐isourea and its consumption by DIC elimination will proceed until all of the BBN‐isoureas are converted predominantly to the 1° alkoxy‐BBN. This leaves predominantly the 2° *O*‐alkyl‐isourea in solution ready to undergo deoxyfluorination to form the 2° alkylfluoride. In contrast, the 1° alkoxy‐BBN species do not undergo deoxyfluorination under these conditions.

These calculations also indicate that the origin of the 2° selectivity in this borane‐mediated nucleophilic substitution process is independent of nucleophile. If this is correct other nucleophiles should give high 2° selectivity in competition reactions using this approach.

### Deoxychlorination

**Scheme 10:**
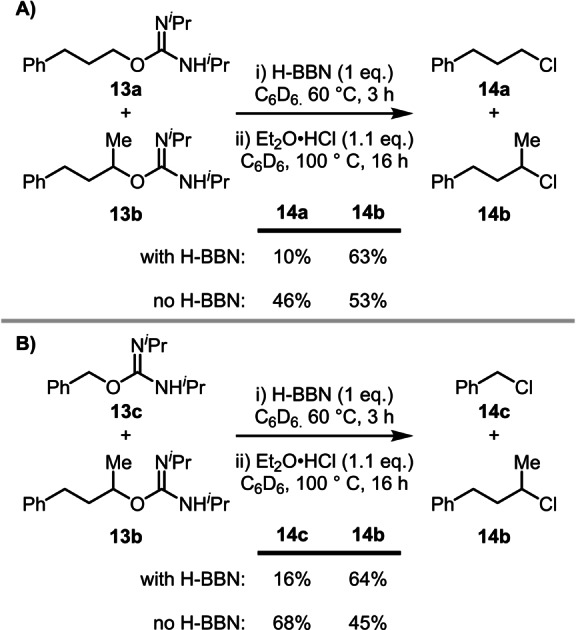
2°‐selective deoxychlorination using boranes.

Deoxychlorination using HCl (in Et_2_O) was explored to test if 2° selectivity could be realized beyond deoxyfluorination. Using comparable conditions to the borane‐mediated deoxyfluorination, an intermolecular competition between a 2° (**13 b**) and a 1° (**13 a**) *O*‐alkyl isourea gave good selectivity for 2° chlorination (6 : 1, 2° : 1°, **14 b** : **14 a**). Significantly, there was extremely poor selectivity in the borane‐free reaction (1.2 : 1, 2° : 1°, Scheme 10A).

In a further competition reaction between the 2° substrate **13 b** and the primary benzylic substrate **13 c**, 2° selectivity for chlorination was observed again when borane mediated (4 : 1, 2° : 1° Bn, **14 b** : **14 c**), with poor selectivity in this case favoring benzylic substitution observed in the H‐BBN free reaction (1 : 1.5, 2° : 1° Bn). This confirms that this borane‐mediated approach to achieve 2°‐selective nucleophilic substitution is not limited to fluoride nucleophiles. The 2°‐selective chlorination is also notable given classic alcohol chlorination routes follow the normal S_N_2 or S_N_1 selectivity trends.[^44^, ^45^]

## Conclusions

The borane‐mediated deoxyfluorination of in situ generated *N*‐H‐*O*‐alkyl‐isoureas represents a novel approach to achieve a highly 2°‐alcohol‐selective substitution in preference to benzylic, 1° and 3° alcohol deoxyfluorination. This general 2° alcohol selectivity is distinct from established deoxyfluorination methods which give products with the selectivity expected from S_N_2 (Bn>1°>2°) or S_N_1 (3°>2°>1°) processes. As borane‐mediated deoxyfluorination can be performed in one‐pot from the starting alcohol under air and requires only commercially available reagents, it represents an operationally simple methodology. The key to the high 2° selectivity is the relative stability of the *N*‐BBN‐*O*‐alkyl‐isoureas towards elimination of the carbodiimide. This elimination step proceeds via a four‐membered transition state in which larger *O*‐alkyl groups induce more distortion resulting in higher DIC elimination barriers. This effect, combined with the rapid (relative to carbodiimide elimination) exchange between *N*‐H‐ and *N*‐BBN‐*O*‐alkyl‐isoureas, results in the selective deactivation of the Bn/1° *O*‐alkyl‐isoureas towards deoxyfluorination by formation of RO‐BBN species. Notably, as the origin of the selectivity does not involve the nucleophile the same approach can be applied with HCl to achieve good 2°‐selective chlorination. Therefore, this indicates significant potential for this borane mediated 2° selective nucleophilic substitution methodology.

## Conflict of Interests

The authors declare no conflict of interest.

1

## Supporting information

As a service to our authors and readers, this journal provides supporting information supplied by the authors. Such materials are peer reviewed and may be re‐organized for online delivery, but are not copy‐edited or typeset. Technical support issues arising from supporting information (other than missing files) should be addressed to the authors.

Supporting Information

## Data Availability

The data that support the findings of this study are available in the supplementary material of this article.
