# Supplementary material for: Borane‐Mediated Highly Secondary Selective Deoxyfluorination of Alcohols
Source: Angew Chem Int Ed Engl. 2025 Jan 9;64(6):e202418495. doi: 10.1002/anie.202418495 (PMC11795720; doi:10.1002/anie.202418495)
Supplement: Supplementary file 1 — Supporting Information [file ANIE-64-e202418495-s001.pdf]

Angewandte Chemie
Eine Zeitschrift der Gesellschaft Deutscher Chemiker

Supporting Information

Borane-Mediated Highly Secondary Selective Deoxyfluorination of Alcohols

D. R. Willcox, N. Cironis, L. Winfrey, S. Kirschner, G. S. Nichol, S. P. Thomas, M. J. Ingleson**

Supporting Information for:

Borane-mediated Highly Secondary Selective Deoxyfluorination of Alcohols

Dominic R. Willcox†, Nojus Cironis†, Laura Winfrey, Sven Kirschner, Gary S. Nichol, Stephen P. Thomas*, and Michael J. Ingleson*

EaStCHEM School of Chemistry, University of Edinburgh, Edinburgh, EH9 3FJ, UK

Email: michael.ingleson@ed.ac.uk

Contents

1. General Information 2
2. F-BBN Mediated Fluorination of Isoureas 3
3. $Et_3N \cdot 3HF$ Mediated Fluorination of Isoureas 17
4. Secondary-Selective Isourea Fluorination Optimisation 36
5. “One-pot” Secondary-Selective Alcohol Deoxyfluorination Optimisation 48
6. Intermolecular Deoxyfluorination Competitions 49
7. Secondary-Selective Deoxyfluorination of Diols 67
8. Enantiospecificity Investigations 74
9. NMR Investigations 76
10. Deoxychlorination 109
11. Synthesis of Alcohols 113
12. Synthesis of Isoureas 117
13. Synthesis of F-BBN 143
14. Computational Details 145
15. X-Ray Crystallography Data 150
16. NMR Spectra of Isolated Compounds 152
17. References 228

1. General Information

Reaction setup: Unless otherwise stated handling of air- and moisture-sensitive reagents was carried out under an inert atmosphere using either standard Schlenk techniques or an *MBraun* glovebox (< 0.1 ppm H_2O/O_2).

Solvents: were obtained from an *Inert PureSolv MD5* solvent purification system. $CDCl_3$ was distilled over CaH_2 and stored over 3 Å molecular sieves protected from air and direct sunlight. Cyclopentyl methyl ether and C_6D_6 were degassed and stored over 3 Å molecular sieves protected from air and direct sunlight.

NMR spectroscopy: (^{1}H, ^{11}B, ^{13}C{^{1}H}, and ^{19}F NMR) were recorded on *Bruker Avance III 400*, *Bruker Avance III 500 MHz*, *Bruker PRO 500 MHz*, or *Bruker Avance I 600 MHz* spectrometers. Chemical shifts are reported as dimensionless δ values and are frequency referenced relative to residual protio-solvent signals in the NMR solvents for ^{1}H and ^{13}C{^{1}H}, while ^{11}B and ^{19}F shifts are referenced relative to external $BF_3{\cdot}Et_2O$ and C_6F_6, respectively. It should be noted that the very broad signals observed at ca. 0 ppm in the ^{11}B NMR spectra are due to the use of borosilicate glass NMR tubes and boron containing parts in the NMR cavity. ^{13}C{^{1}H} NMR spectra are listed as ^{13}C. Multiplicities are shown as s (singlet), d (doublet), t (triplet), q (quartet), quin. (quintet), sext. (sextet), sept. (septet). Coupling constants J are given in Hertz (Hz) as positive values regardless of their real individual signs. Unless otherwise stated NMR spectra were recorded at 20 °C. Reaction yields and conversions were measured by quantitative ^{19}F NMR spectroscopy in a Bruker Avance III 500 MHz spectrometer or a Bruker Avance I 600 MHz spectrometer (zg30, acquisition time = 1.17 s, relaxation delay = 20 s, number of scans = 8). NMR spectra were analysed by MestReNova processing software.

Mass spectrometry: was performed by the *Scottish Instrumentation and Resource Centre for Advanced Mass Spectrometry* (*SIRCAMS*) of the University of Edinburgh using either electron impact (EI) or electrospray ionisation (ESI) techniques. Accurate masses are calculated using the most abundant isotopes of each element.

Column chromatography: Column chromatography was carried out on a Teledyne ISCO CombiFlash NextGen 300+ using RediSep Rf normal phase silica flash columns (12, 25, 40, or 80 g; 20-40 microns). Substrates were purified using 40/60 petroleum ether and EtOAc on a gradient of 100:0 to 0:100 with flow rates of 10-110 mL min^{-1} depending on the size of column and ΔR_f.

Chemicals: All reagents were purchased from Sigma Aldrich, Alfa Aesar, Acros organics, Tokyo Chemical Industries UK, Fluorochem or Strem UK and were used without further purification unless otherwise stated. H-BBN dimer was prepared according to literature procedure.[1] H-BBN in THF (0.5 M) was purchased from Strem UK, transferred to a J-Young ampoule under an atmosphere of N_2, and used without further purification. $Et_3N{\cdot}3HF$ was purchased from Fluorochem and used without further purification (*safety: anything in contact with $Et_3N{\cdot}3HF$ was quenched with saturated $CaCl_{2(aq)}$ solution; all work was carried out with a calcium gluconate gel kit nearby to treat potential exposure*). CsF was obtained from Sigma Aldrich and dried at 120 °C for 6 h *in vacuo*. The dried material was then finely ground and stored in a glovebox. KF was obtained from *Honeywell* (>99%, spray dried), finely ground and stored in a glovebox without any further purification.

2. F-BBN Mediated Fluorination of Isoureas

Controls

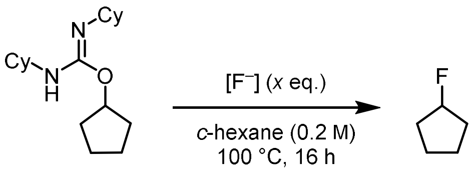

In a J-Young NMR tube, a solution of *O*-(cyclopentyl)-*N*,*N'*-dicyclohexylisourea (29.2 mg, 0.100 mmol) and the [F⁻] source (*x* mmol) in cyclohexane (0.600 mL) was heated at 100 °C for 16 hours. The yield was determined by ^{19}F NMR spectroscopy using 1,3,5-trifluorobenzene and hexafluorobenzene as internal standards.

Table S1. Isourea fluorination using different fluoride sources.

Entry	[F⁻] (eq.)	Yield / %
1	F-BBN (1)	35
2	F-BBN (1) + H_2O (3)	71
3	Et_3N•3HF (0.33)	67
4	F-BBN (2) + H_2O (3)	72
5	F-BBN (2)	42

Competitions

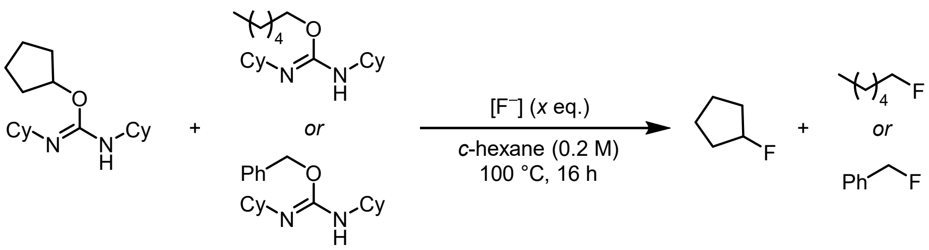

In a J-Young NMR tube under argon, a solution of *O*-(cyclopentyl)-*N*,*N′*-dicyclohexylisourea (29.2 mg, 0.100 mmol), *isourea 2* (0.100 mmol), and [F⁻] source (*x* mmol) in cyclohexane (0.600 mL) was heated at 100 °C for 16 hours. The yield was determined using 1,3,5-trifluorobenzene and hexafluorobenzene as internal standards.

Table S2. Intermolecular isourea fluorination competition.

Entry	Isourea 2	[F⁻] (eq.)	2°-F / %	1°-F / %
1	1-Hexyl	F-BBN (1)	63	trace
2	1-Hexyl	Et_3N•3HF (0.33)	65	27

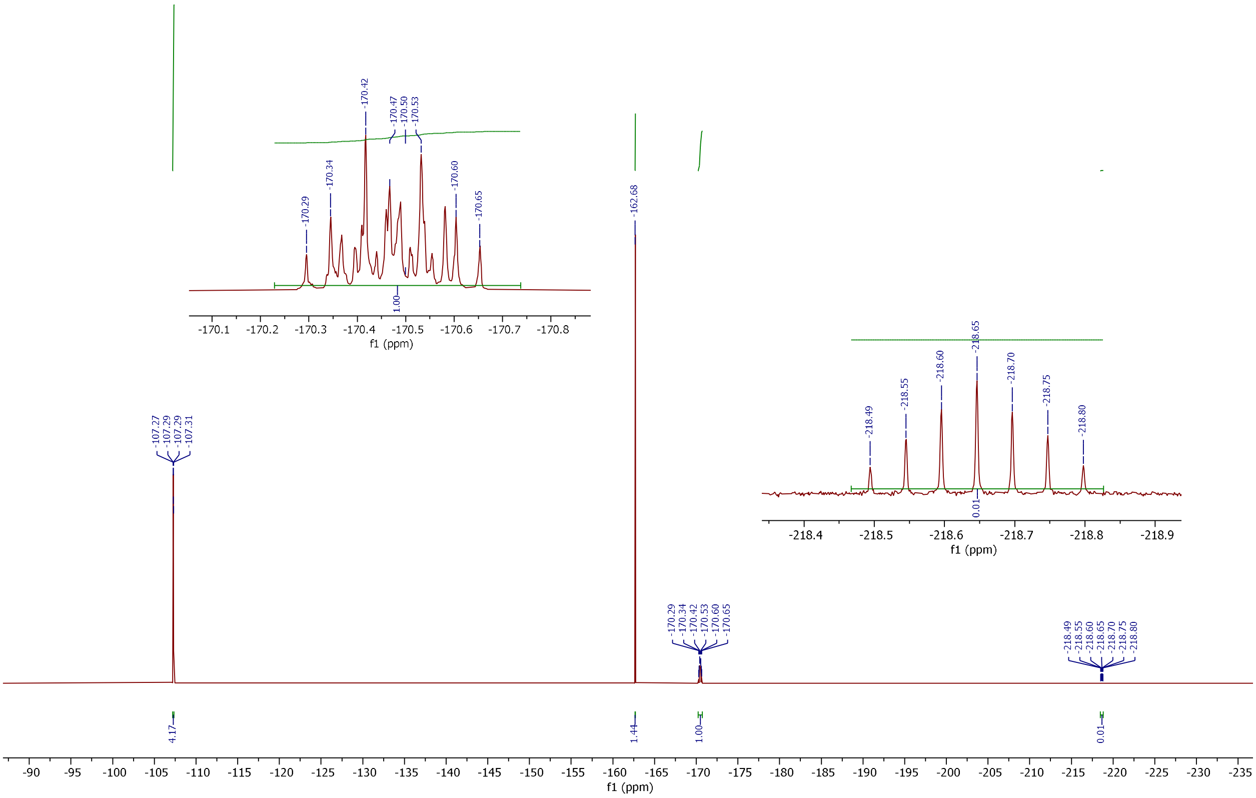

Figure S1. Crude ^{19}F NMR spectrum for entry 1 the competition reaction between *O*-(cyclopentyl)-*N*,*N′*-dicyclohexylisourea and *O*-(hexyl)-*N*,*N′*-dicyclohexylisourea with hexafluorobenzene (−162.68 ppm, 3.0 mg) and 1,3,5-trifluorobenzene (−107.29 ppm, 11.1 mg) as internal standards. 3-fluorocyclopentane (−170.47 ppm) and 1-fluorohexane (−218.65 ppm).

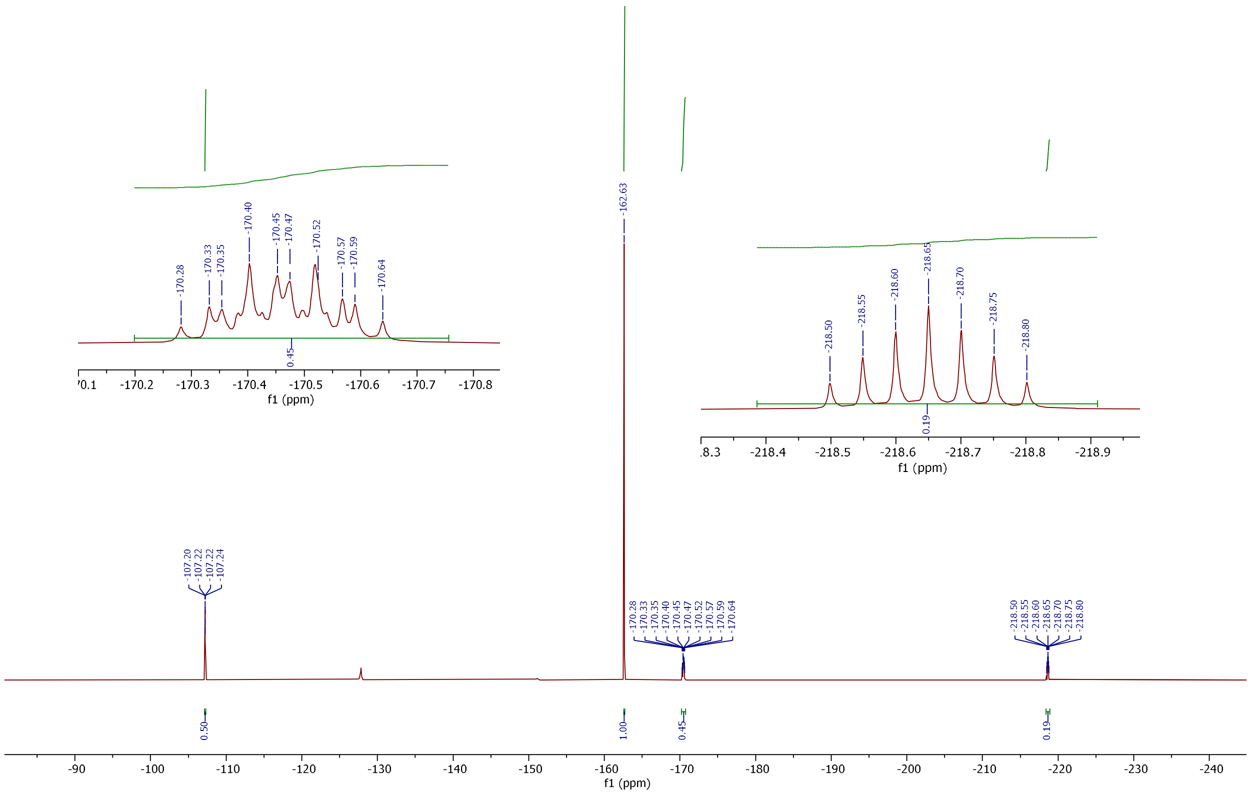

Figure S2. Crude ^{19}F NMR spectrum for entry 2 the competition reaction between *O*-(cyclopentyl)-*N*,*N'*-dicyclohexylisourea and *O*-(hexyl)-*N*,*N'*-dicyclohexylisourea with hexafluorobenzene (−162.63 ppm, 4.5 mg) and 1,3,5-trifluorobenzene (−107.22 ppm, 3.8 mg) as internal standards. 3-fluorocyclopentane (−170.47 ppm) and 1-fluorohexane (−218.65 ppm).

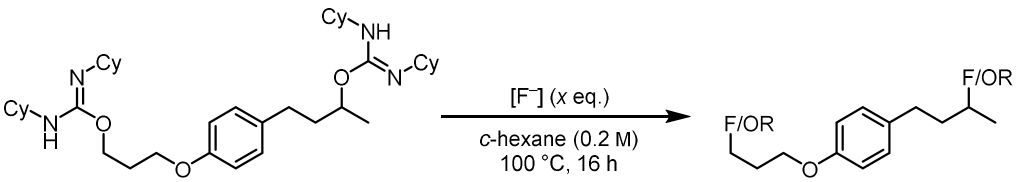

In a J-Young NMR tube, a solution of 4-(4-(3-*O*-(*N*,*N'*-dicyclohexylcarbamimidyl)-propoxy)phenyl)butan-2-*O*-*N*,*N'*-dicyclohexylcarbamimidate (63.7 mg, 0.100 mmol) and [F$^-$] source (*x* mmol) in cyclohexane (0.600 mL) was heated at 100 °C for 16 hours. The yield was determined using 1,3,5-trifluorobenzene and hexafluorobenzene as internal standards.

Table S3. Intramolecular isourea fluorination competition.

Entry	[F$^-$] (eq.)	2°-F / %	1°-F / %
1	F-BBN (1)	39	Trace
2	Et_3N•3HF (0.33)	41	31

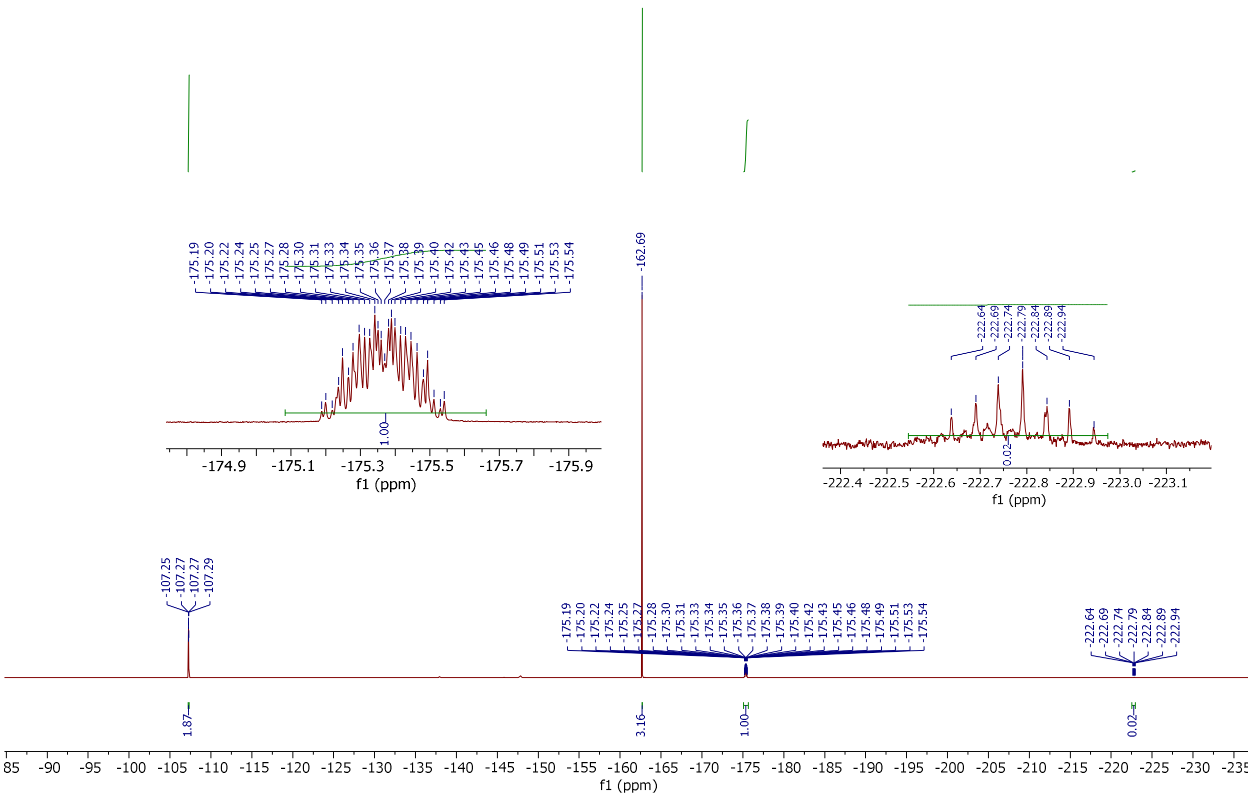

Figure S3. Crude ^{19}F NMR spectrum for entry 1 for the intramolecular competition reaction with bis-isourea with hexafluorobenzene (−162.69 ppm, 4.1 mg) and 1,3,5-trifluorobenzene (−107.27 ppm, 3.5 mg) as internal standards. Primary fluorination at −222.79 ppm and secondary fluorination at −175.36 ppm.

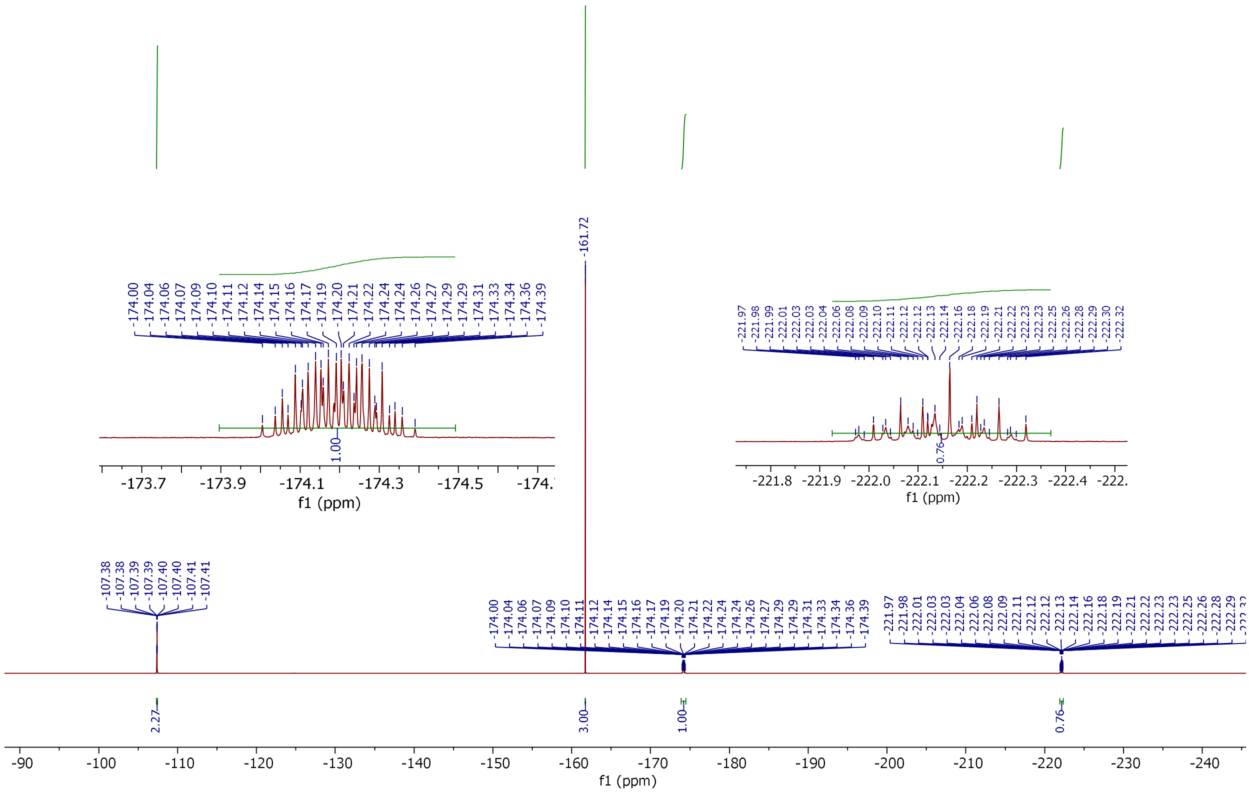

Figure S4. Crude ^{19}F NMR spectrum for entry 2 for the intramolecular competition reaction with bis-isourea with hexafluorobenzene (−162.72 ppm, 4.2 mg) and 1,3,5-trifluorobenzene (−107.40 ppm, 3.7 mg) as internal standards. Primary fluorination at −222.14 ppm and secondary fluorination at −174.22 ppm.

Substrate Scope

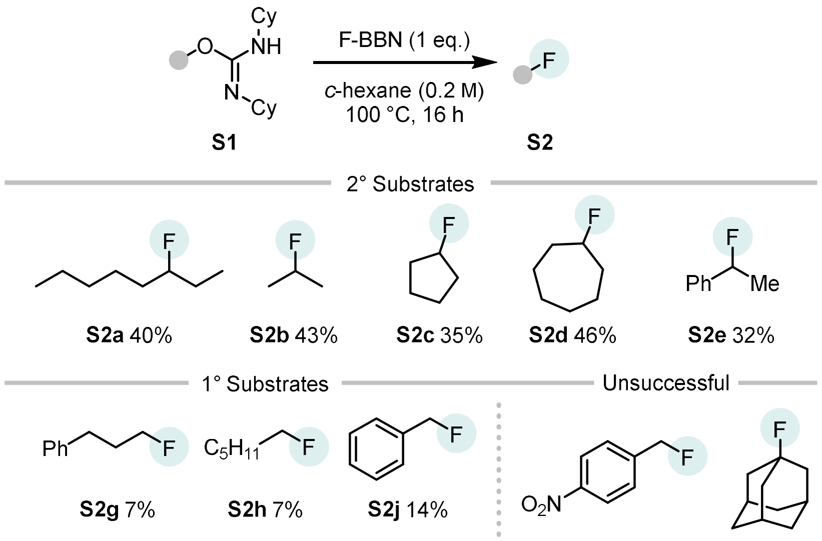

General Procedure A:

In a J-Young NMR tube, a solution of isourea (0.100 mmol) and F-BBN (14.0 mg, 0.100 mmol) in cyclohexane (0.600 mL) was heated at 100 °C for 16 hours. The yield was determined using 1,3,5-trifluorobenzene and/or hexafluorobenzene added as internal standards at the end of the reaction.

3-Fluorooctane S2a

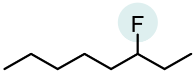

According to general procedure A, *O*-(3-octyl)-*N*,*N'*-dicyclohexylisourea (33.7 mg, 0.100 mmol) was reacted to give 3-fluorooctane (40%).

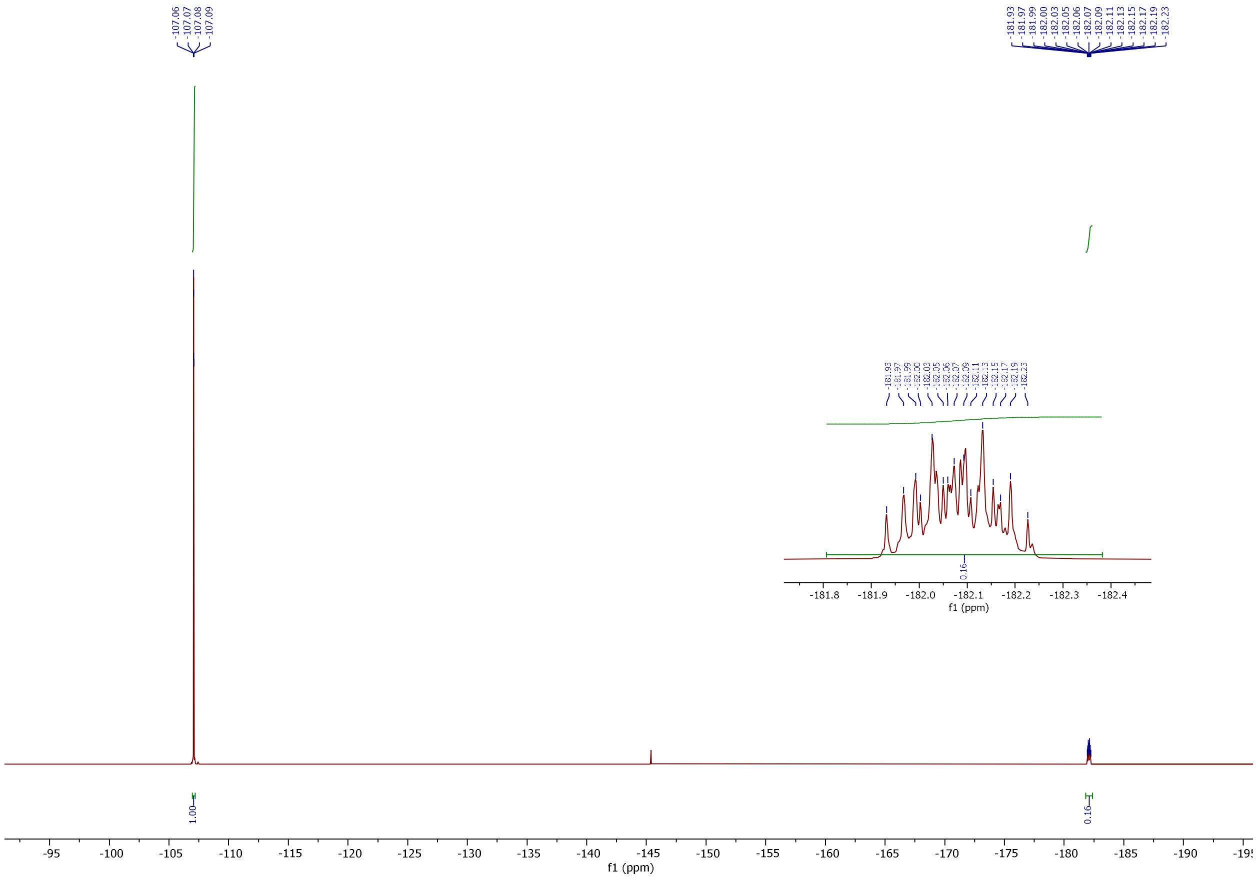

Figure S5. Crude ^{19}F NMR spectrum for 3-fluorooctane (−182.09 ppm) with 1,3,5 – trifluorobenzene (−107.07 ppm, 9.0 mg) as an internal standard. Data were in accordance to those previously reported.[2]

2-Fluoropropane S2b

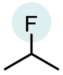

According to general procedure A, *O*-(2-propyl)-*N*,*N'*-dicyclohexylisourea (26.6 mg, 0.100 mmol) was reacted to give 2-fluoropropane (43%).

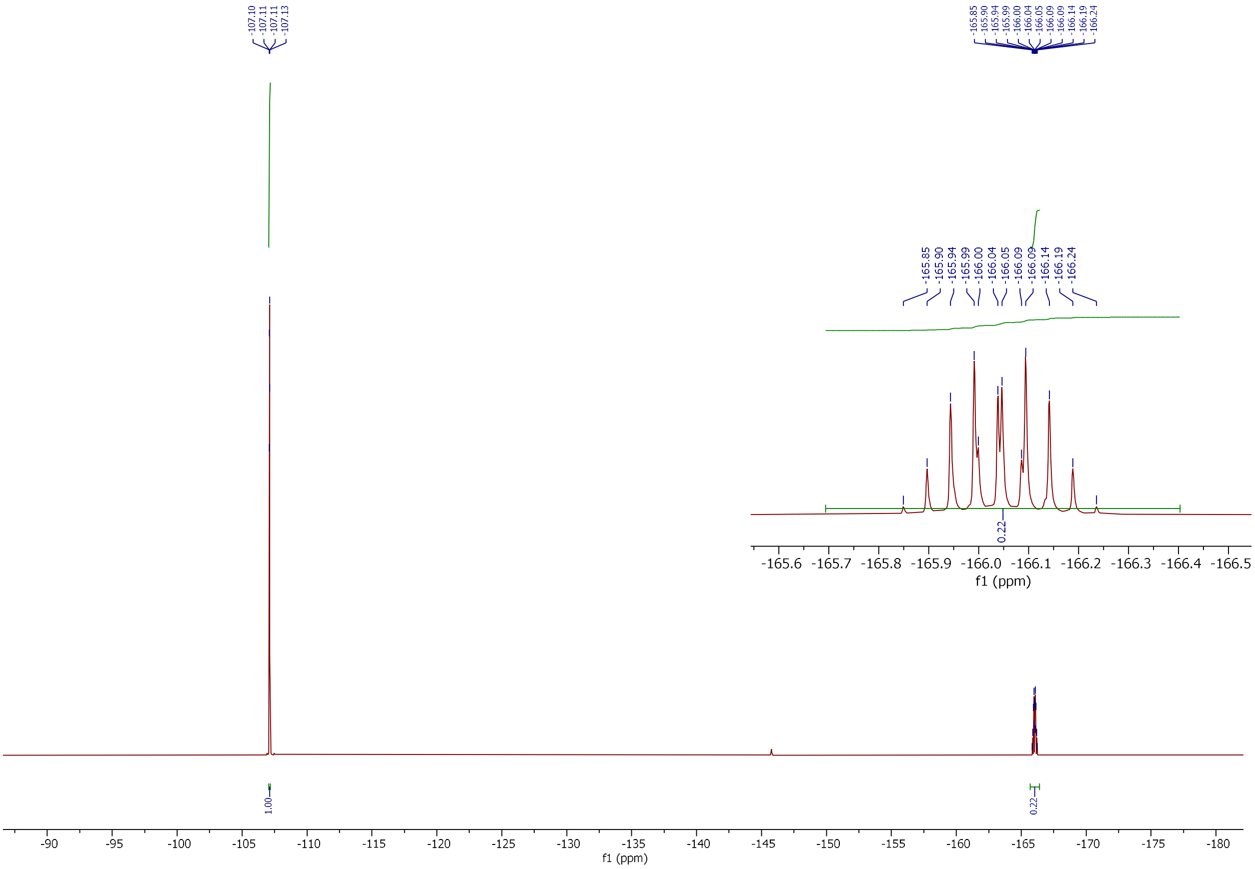

Figure S6. Crude ^{19}F NMR spectrum for 2-fluoropropane (−166.05 ppm) with 1,3,5 –trifluorobenzene (−107.11 ppm, 7.6 mg) as an internal standard. Data were in accordance to those previously reported.[3]

Fluorocyclopentane S2c

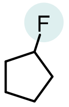

According to general procedure A, *O*-(cyclopentyl)-*N*,*N'*-dicyclohexylisourea (29.2 mg, 0.100 mmol) was reacted to give fluorocyclopentane (35%).

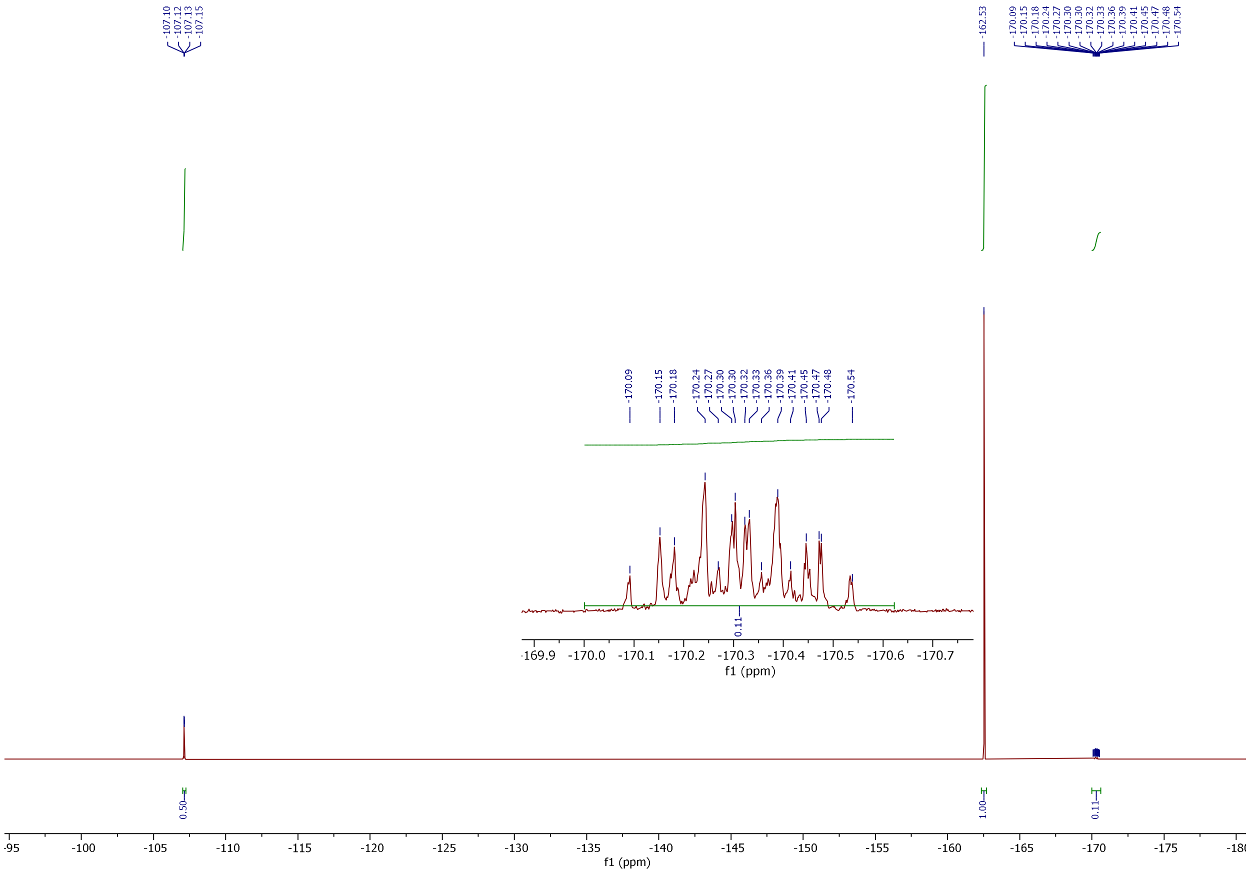

Figure S7. Crude ^{19}F NMR spectrum for fluorocyclopentane (−170.33 ppm) with hexafluorobenzene (−162.53 ppm, 9.6 mg) and 1,3,5 – trifluorobenzene (−107.13 ppm, 7.1 mg) as internal standards. Data were in accordance to those previously reported.[4]

Fluorocycloheptane S2d

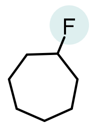

According to general procedure A, *O*-(cycloheptyl)-*N*,*N'*-dicyclohexylisourea (32.1 mg, 0.100 mmol) was reacted to give fluorocycloheptane (46%).

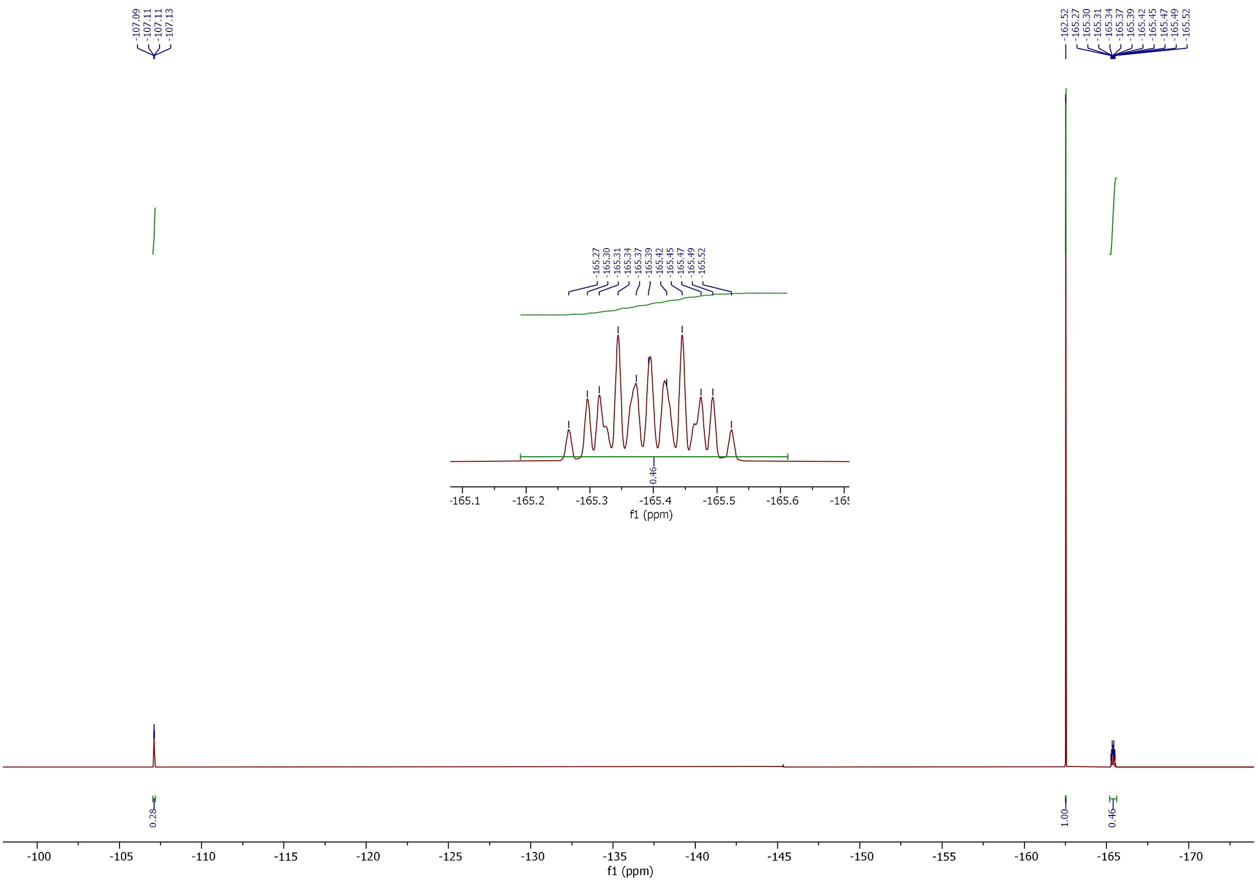

Figure S8. Crude ^{19}F NMR spectrum for fluorocycloheptane (−169.39 ppm) with hexafluorobenzene (−162.52 ppm, 7.5 mg) and 1,3,5 – trifluorobenzene (−107.11 ppm, 9.0 mg) as internal standards. Data were in accordance to those previously reported.[5]

1-Fluoro-1-phenylethane S2e

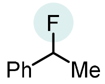

According to general procedure A, *O*-(1-phenyl-1-ethyl)-*N*,*N'*-dicyclohexylisourea (32.9 mg, 0.100 mmol) was reacted to give 1-fluoro-1-phenylethane (32%).

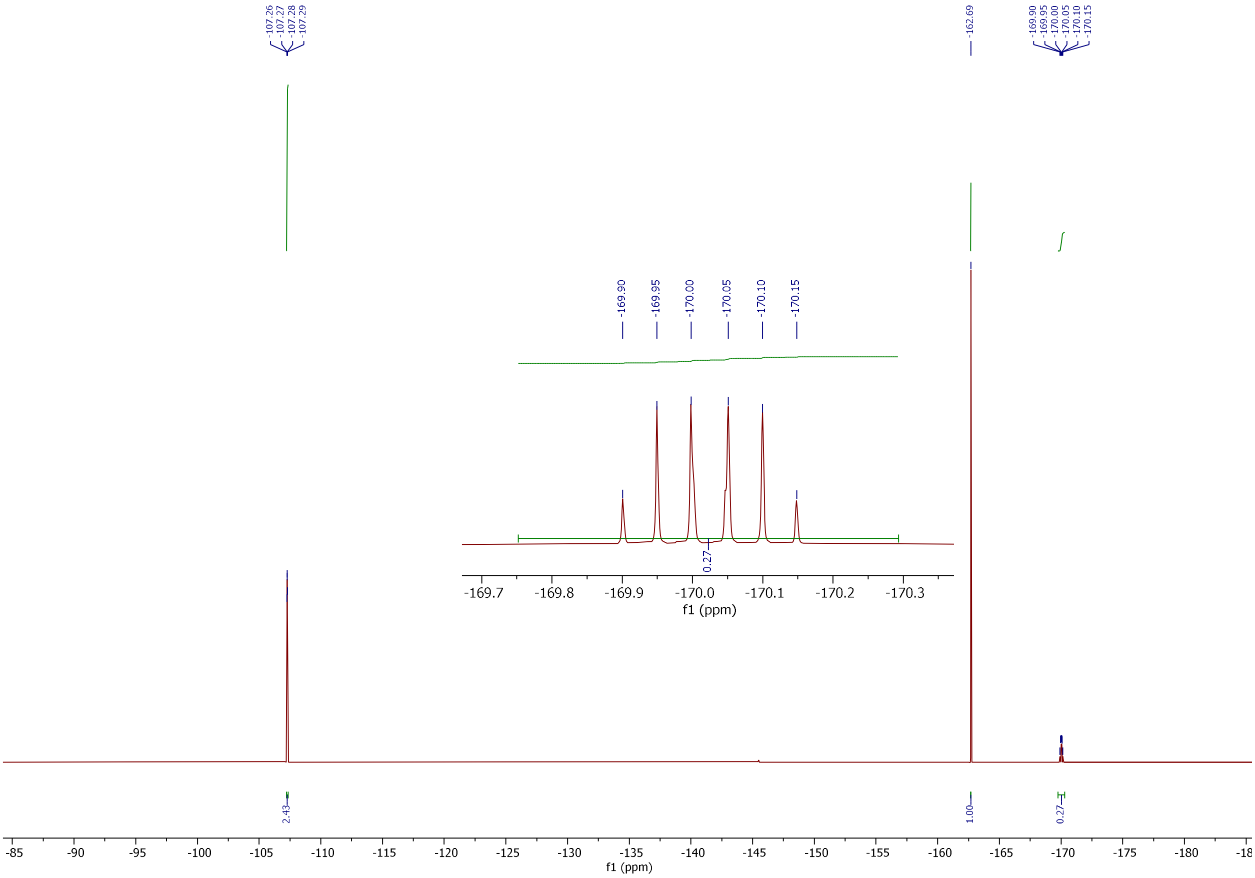

Figure S9. Crude ^{19}F NMR spectrum for 1-fluoro-1-phenylethane (−170.00 ppm) with hexafluorobenzene (−162.69 ppm, 3.8 mg) and 1,3,5 – trifluorobenzene (−107.28 ppm, 11.3 mg) as internal standards. Data were in accordance to those previously reported.[6]

1-Fluoro-3-phenylpropane S2g

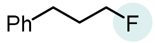

According to general procedure A, *O*-(3-phenyl-1-propyl)-*N*,*N'*-dicyclohexylisourea (34.3 mg, 0.100 mmol) was reacted to give 1-fluoro-3-phenylpropane (7%).

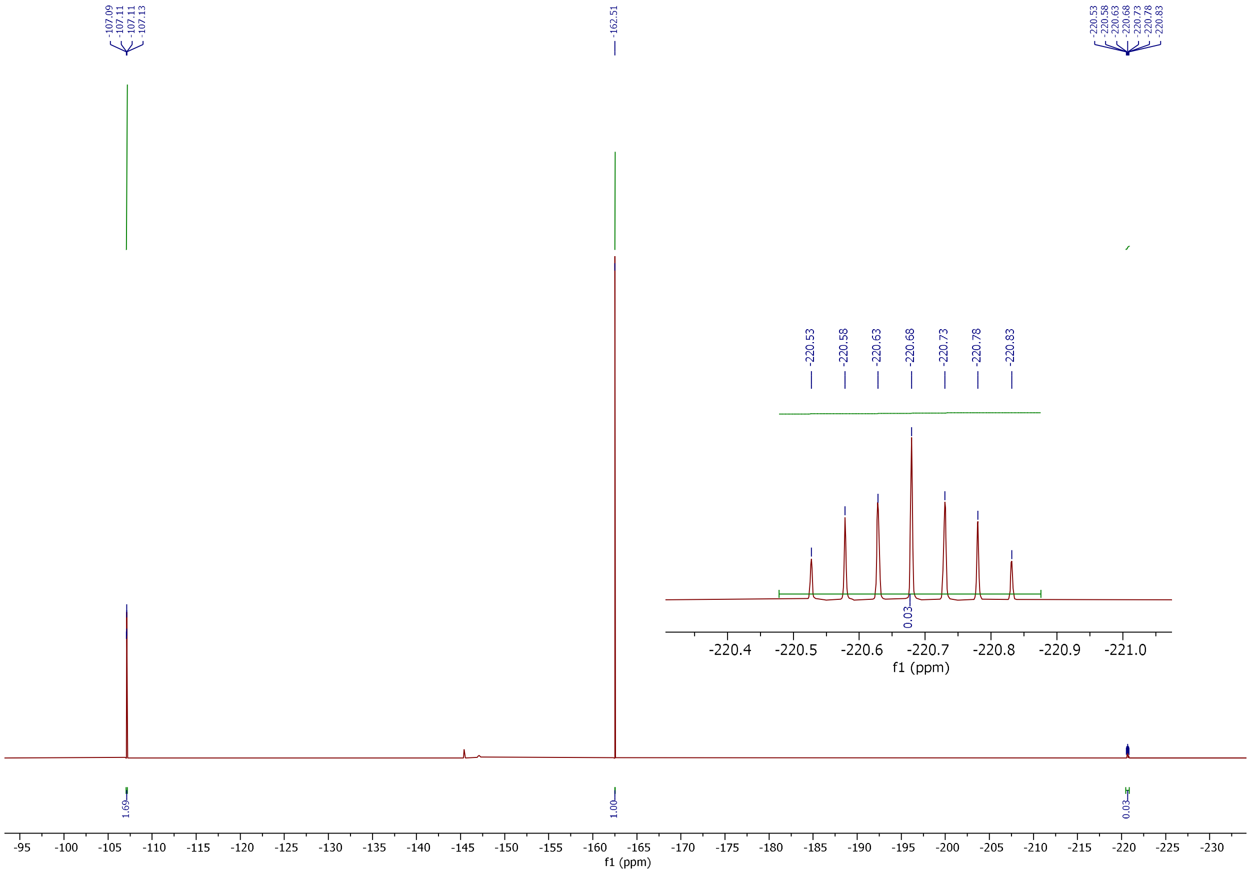

Figure S10. Crude ^{19}F NMR spectrum for 1-fluoro-3-phenylpropane (−220.68 ppm) with hexafluorobenzene (−162.51 ppm, 5.1 mg) and 1,3,5 – trifluorobenzene (−107.11 ppm, 12.0 mg) as internal standards. Data were in accordance to those previously reported.[7]

1-Fluorohexane S2h

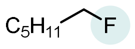

According to general procedure A, *O*-(1-hexyl)-*N*,*N'*-dicyclohexylisourea (30.9 mg, 0.100 mmol) was reacted to give 1-fluorohexane (7%).

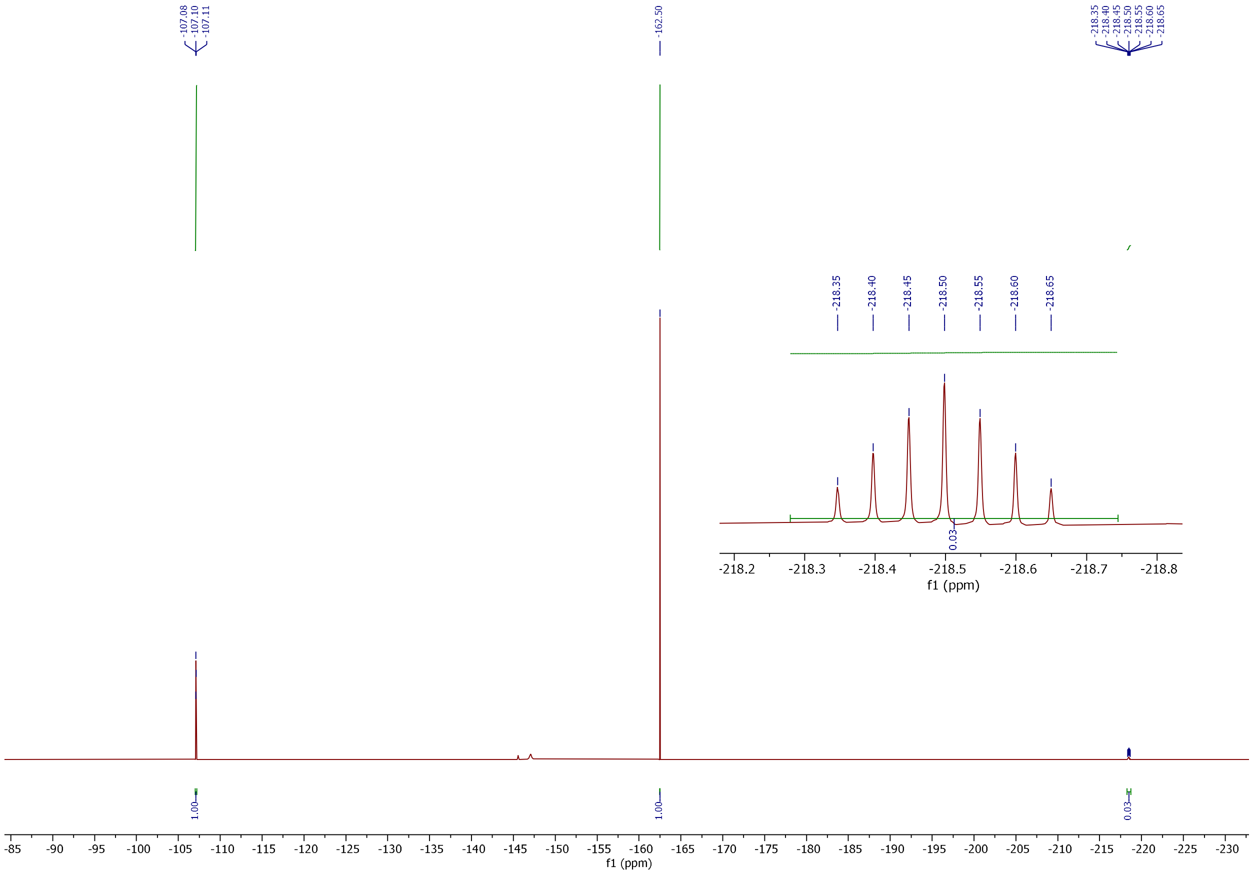

Figure S11. Crude ^{19}F NMR spectrum for 1-fluorohexane (−218.50 ppm) with hexafluorobenzene (−162.50 ppm, 5.2 mg) and 1,3,5 – trifluorobenzene (−107.10 ppm, 9.4 mg) as internal standards. Data were in accordance to those previously reported.[8]

Benzyl Fluoride S2j

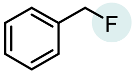

According to general procedure A, *O*-benzyl-*N*,*N'*-dicyclohexylisourea (31.4 mg, 0.100 mmol) was reacted to give benzyl fluoride (14%).

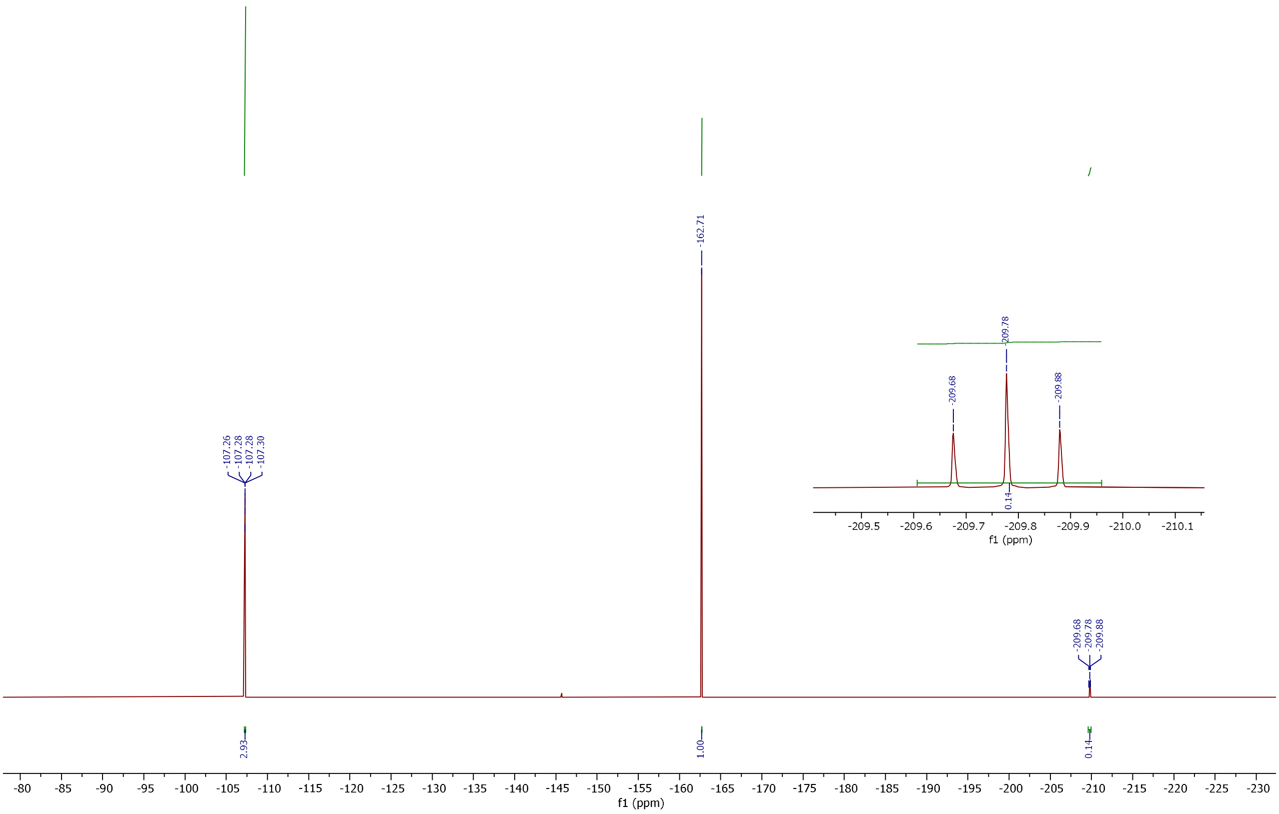

Figure S12. Crude ^{19}F NMR spectrum for the benzyl fluoride product (−209.78 ppm) with hexafluorobenzene (−162.71 ppm, 3.3 mg) and 1,3,5 – trifluorobenzene (−107.26 ppm, 12.1 mg) as internal standards. Data were in accordance to those previously reported.[9]

3. $Et_3N \cdot 3HF$ Mediated Fluorination of Isoureas

Scope

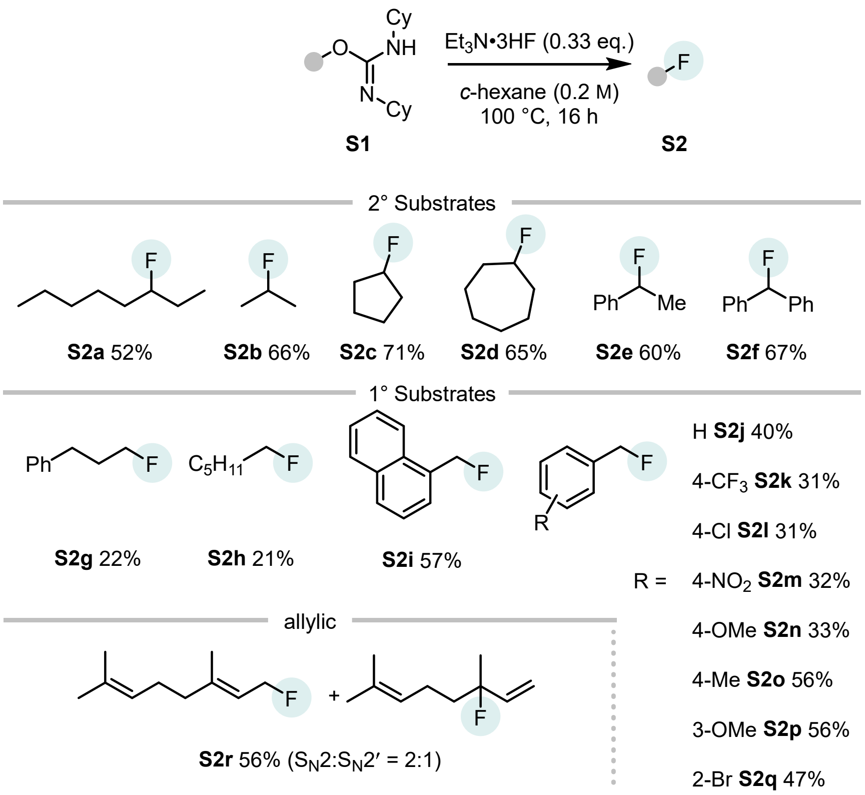

General Procedure B:

In a J-Young NMR tube, a solution of isourea (0.100 mmol) and $Et_3N \cdot 3HF$ (5.4 µL, 33.0 µmol) in cyclohexane (0.600 mL) was heated at 100 °C for 16 hours. The yield was determined using 1,3,5-trifluorobenzene and/or hexafluorobenzene as internal standards.

3-Fluorooctane S2a

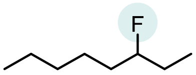

According to general procedure B, *O*-(3-octyl)-*N*,*N'*-dicyclohexylisourea (33.7 mg, 0.100 mmol) was reacted to give 3-fluorooctane (52%).

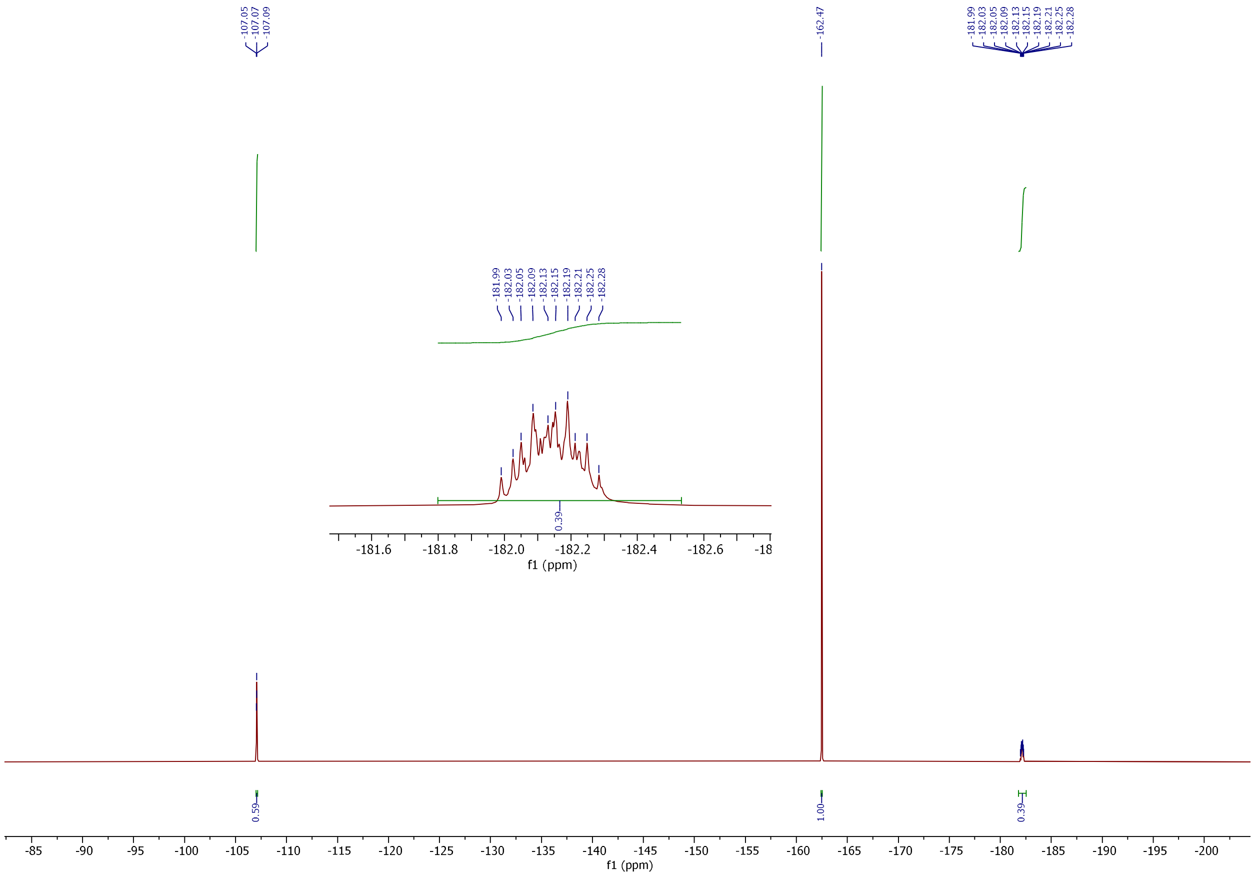

Figure S13. Crude ^{19}F NMR spectrum for 3-fluorooctane (−182.15 ppm) with hexafluorobenzene (−162.47 ppm, 8.2 mg) and 1,3,5 – trifluorobenzene (−107.07 ppm, 6.7 mg) as internal standards. Data were in accordance to those previously reported.[2]

2-Fluoropropane S2b

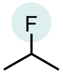

According to general procedure B, *O*-(2-propyl)-*N*,*N'*-dicyclohexylisourea (26.6 mg, 0.100 mmol) was reacted to give 2-fluoropropane (66%).

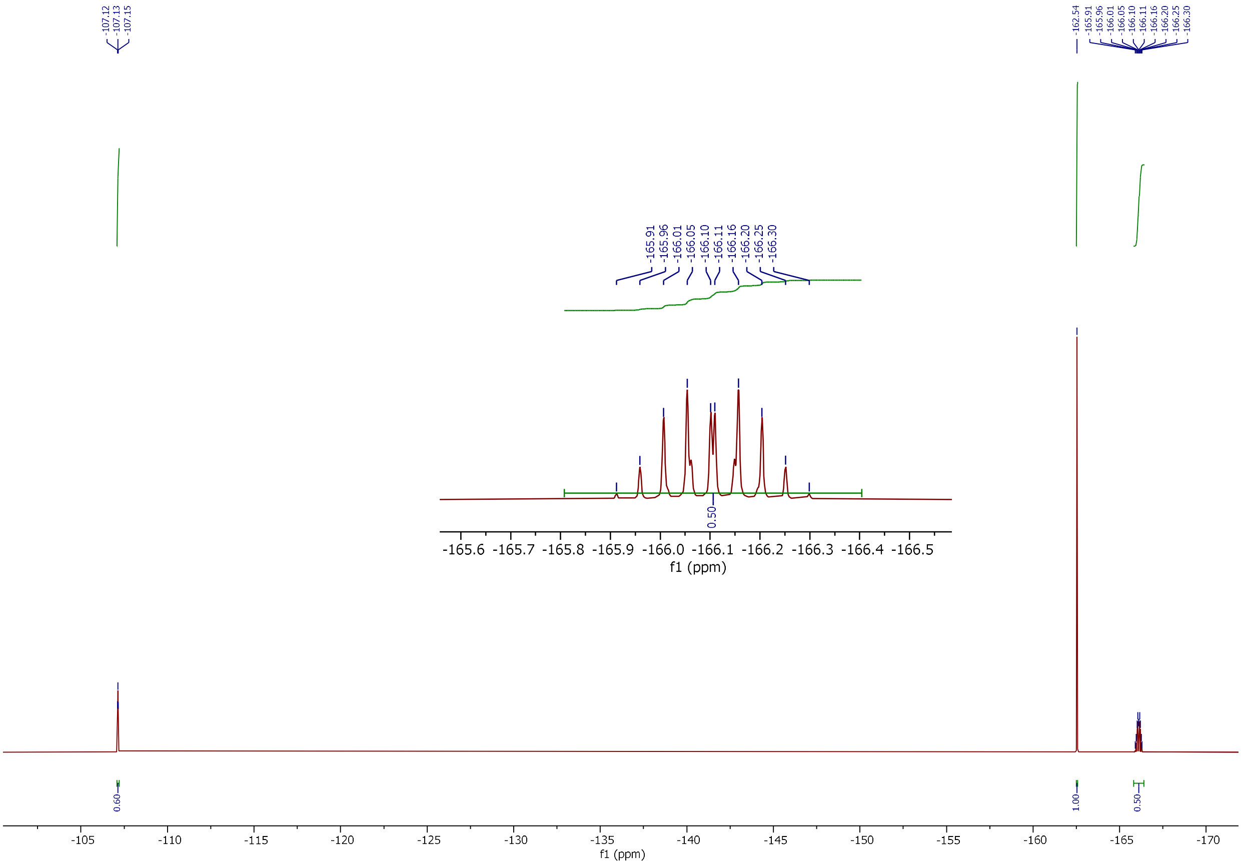

Figure S14. Crude ^{19}F NMR spectrum for 2-fluoropropane (−166.10 ppm) with hexafluorobenzene (−162.54 ppm, 8.3 mg) and 1,3,5 – trifluorobenzene (−107.13 ppm, 6.6 mg) as internal standards. Data were in accordance to those previously reported.[3]

Fluorocyclopentane S2c

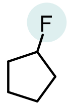

According to general procedure B, *O*-(cyclopentyl)-*N*,*N'*-dicyclohexylisourea (29.2 mg, 0.100 mmol) was reacted to give fluorocyclopentane (71%).

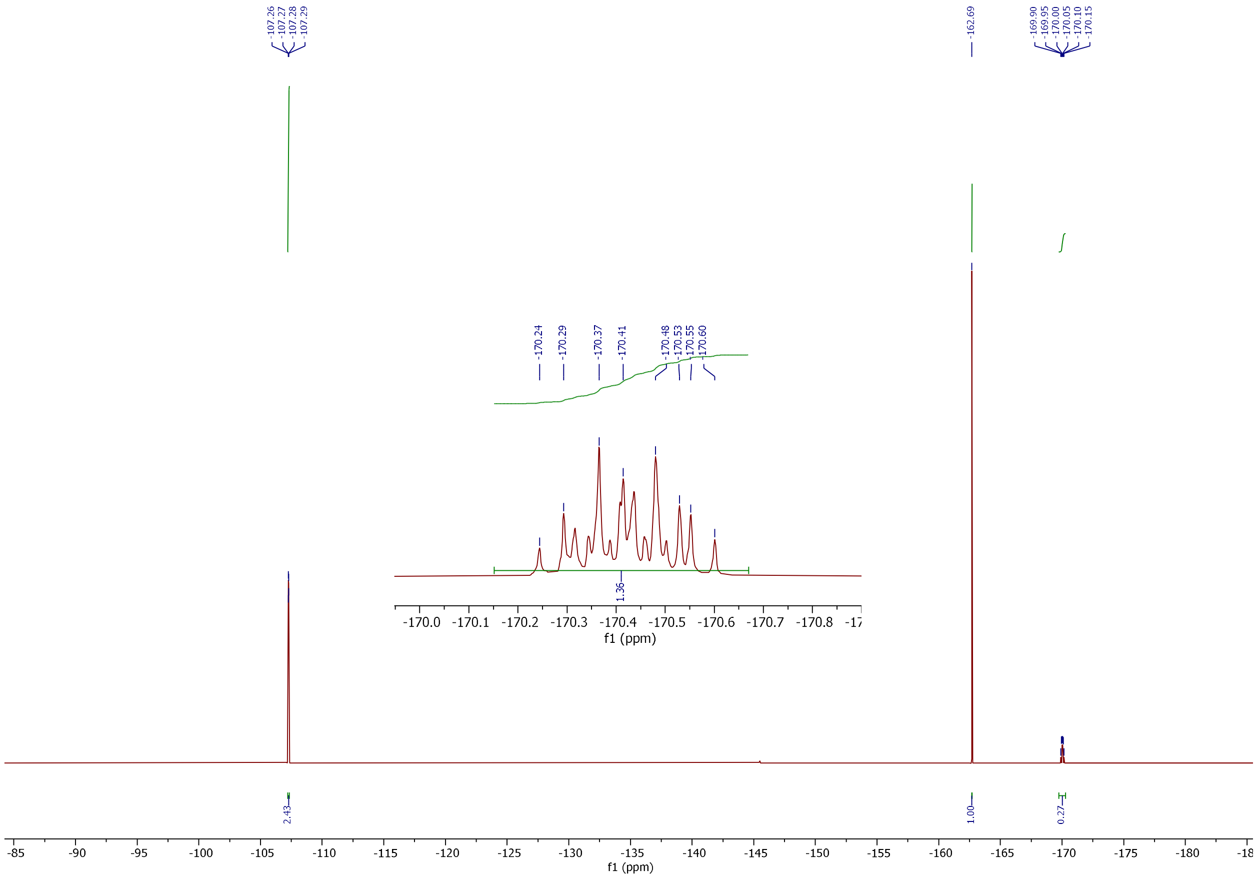

Figure S15. Crude ^{19}F NMR spectrum for 1-fluorocyclopentane (−170.41 ppm) with hexafluorobenzene (−162.56 ppm, 3.4 mg) and 1,3,5 – trifluorobenzene (−107.16 ppm, 6.4 mg) as internal standards. Data were in accordance to those previously reported.[4]

Fluorocycloheptane S2d

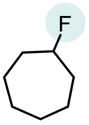

According to general procedure B, *O*-(cycloheptyl)-*N*,*N'*-dicyclohexylisourea (32.1 mg, 0.100 mmol) was reacted to give fluorocycloheptane (65%).

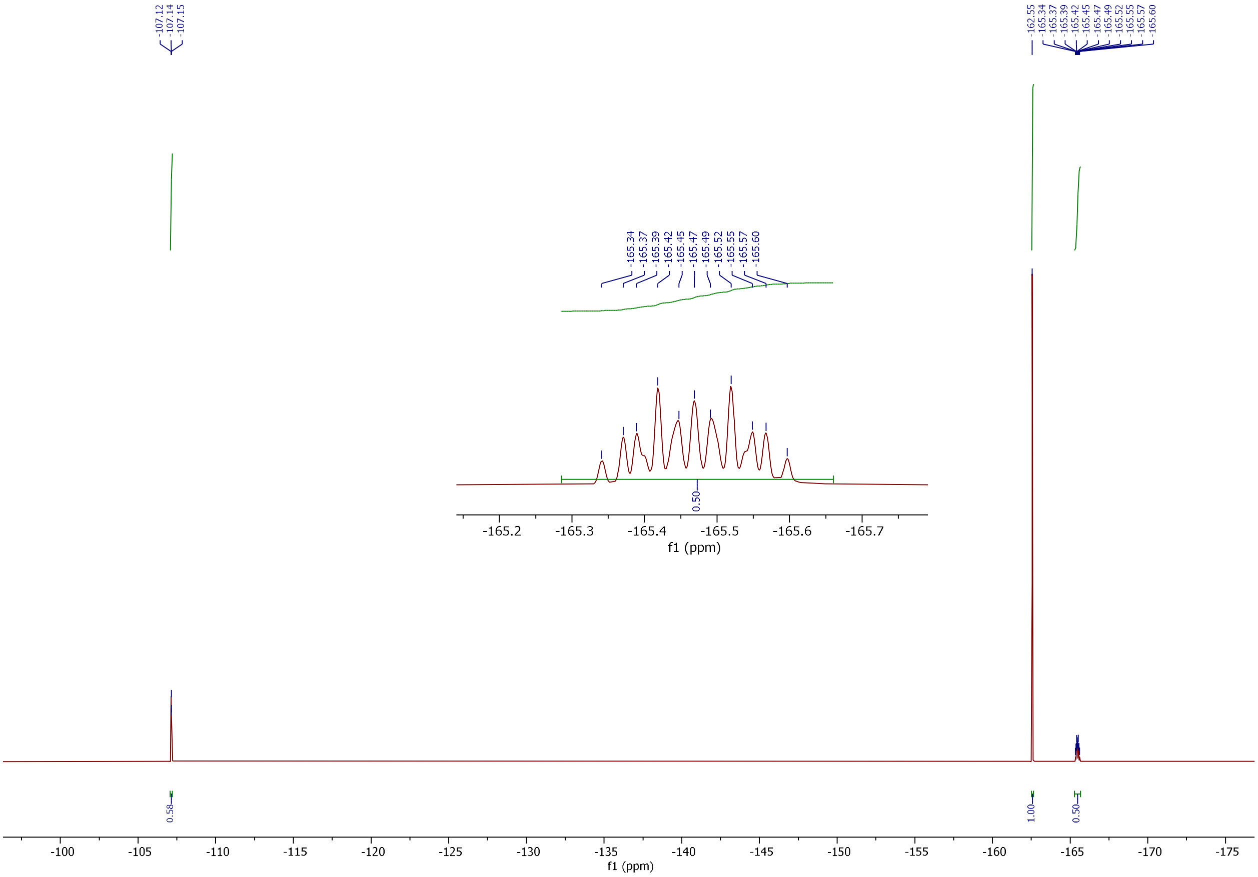

Figure S16. Crude ^{19}F NMR spectrum for fluorocycloheptane (−165.47 ppm) with hexafluorobenzene (−162.55 ppm, 7.9 mg) and 1,3,5 – trifluorobenzene (−107.14 ppm, 6.7 mg) as internal standards. Data were in accordance to those previously reported.[5]

1-Fluoro-1-phenylethane S2e

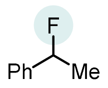

According to general procedure B, *O*-(1-phenyl-1-ethyl)-*N*,*N'*-dicyclohexylisourea (32.9 mg, 0.100 mmol) was reacted to give 1-fluoro-1-phenylethane (60%).

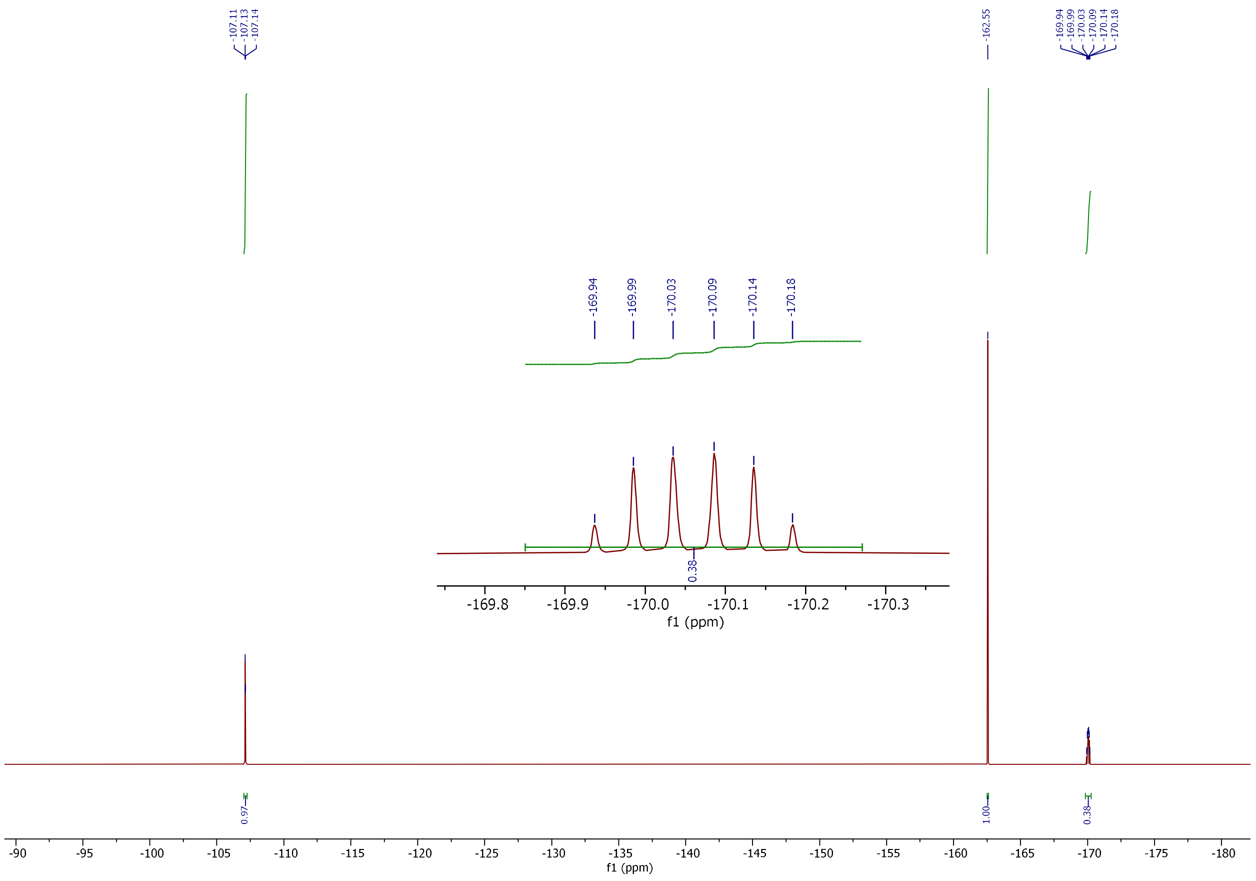

Figure S17. Crude ^{19}F NMR spectrum for the 1-fluoro-1-phenylethane product (−170.06 ppm) with hexafluorobenzene (−162.55 ppm, 5.0 mg) and 1,3,5 – trifluorobenzene (−107.13 ppm, 6.8 mg) as internal standards. Data were in accordance to those previously reported.[6]

Fluorodiphenylmethane S2f

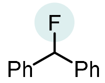

According to general procedure B, *O*-(diphenylmethyl)-*N*,*N′*-dicyclohexylisourea (39.1 mg, 0.100 mmol) was reacted to give fluorodiphenylmethane (67%).

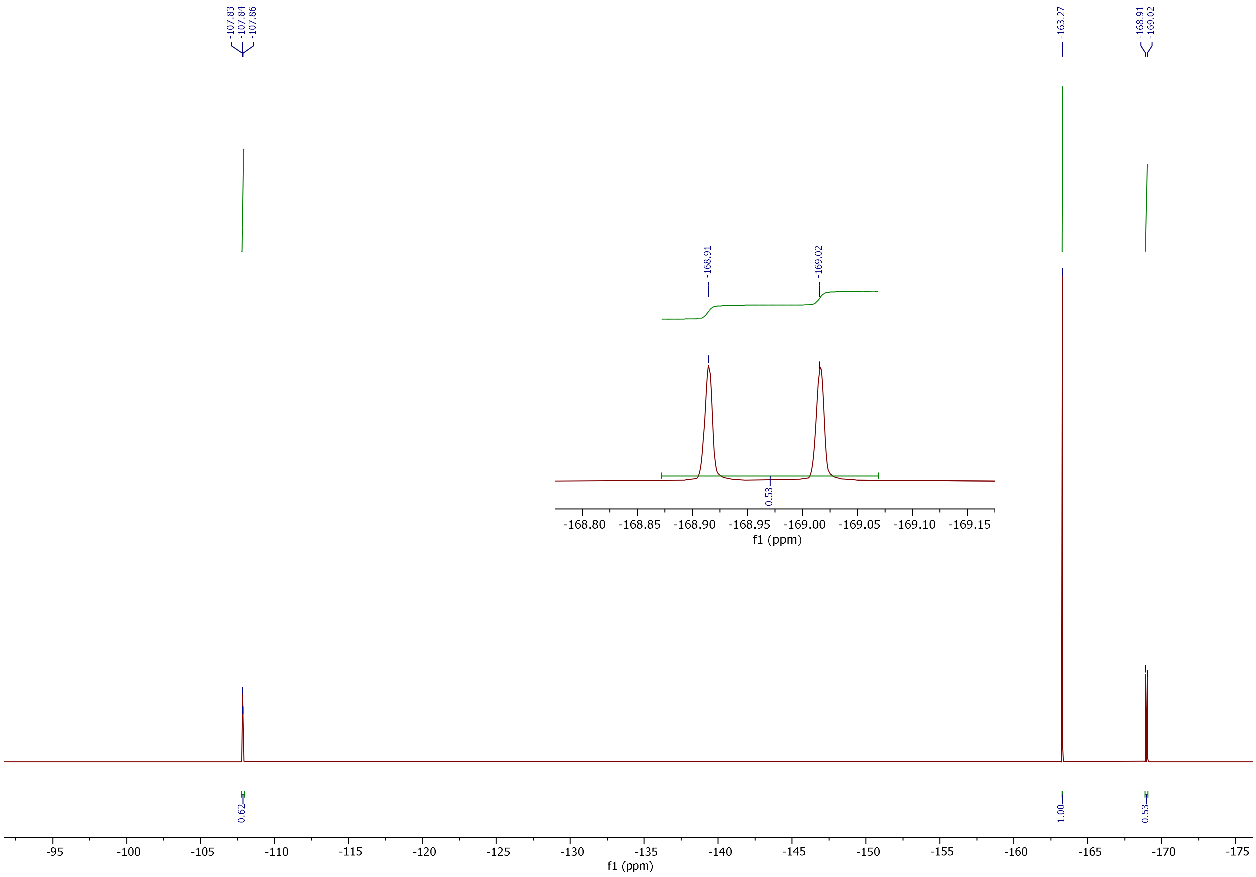

Figure S18. Crude ^{19}F NMR spectrum for fluorodiphenylmethane (−168.96 ppm) with hexafluorobenzene (−162.27 ppm, 8.0 mg) and 1,3,5 – trifluorobenzene (−107.84 ppm, 6.7 mg) as internal standards. Data were in accordance to those previously reported.[6]

1-Fluoro-3-phenylpropane S2g

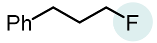

According to general procedure B, *O*-(3-phenyl-1-propyl)-*N*,*N'*-dicyclohexylisourea (34.3 mg, 0.100 mmol) was reacted to give 1-fluoro-3-phenylpropane (22%).

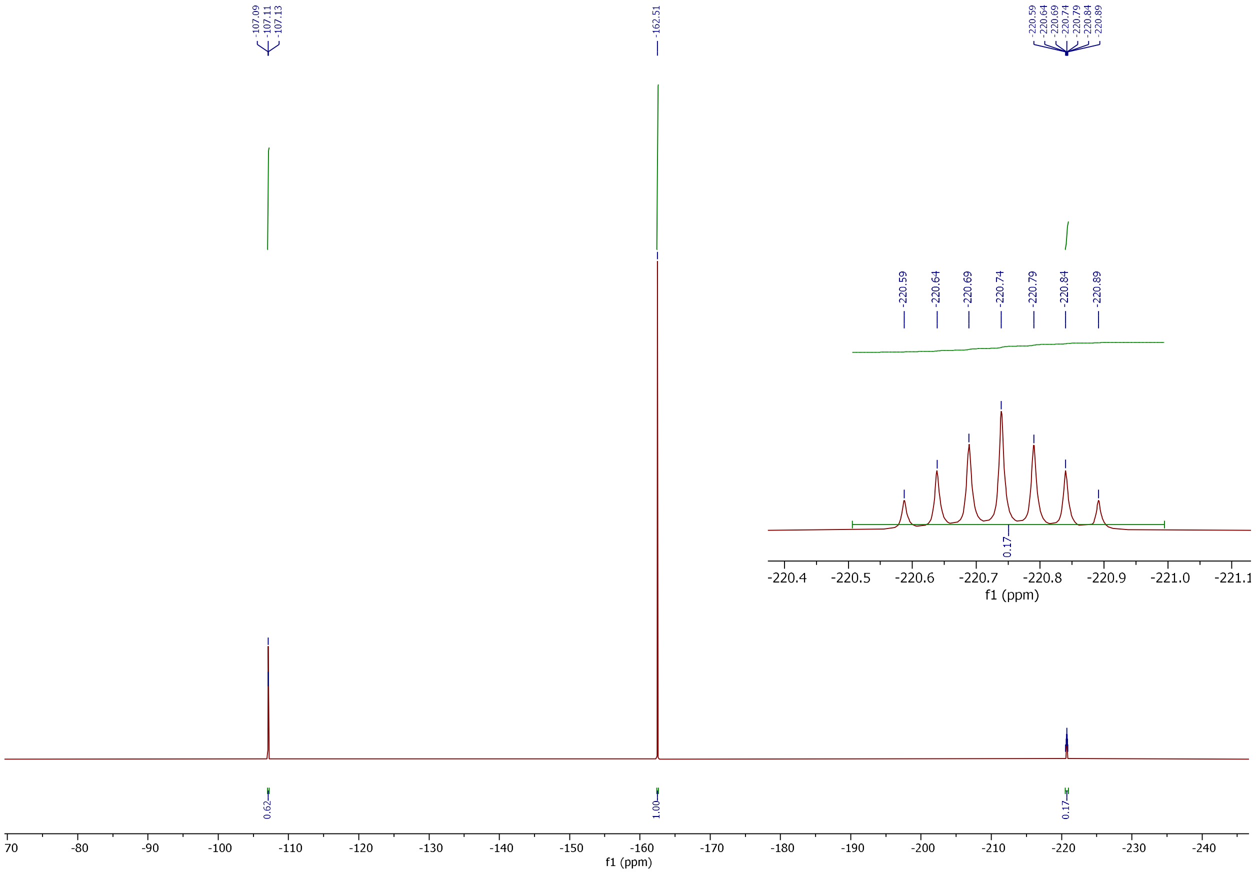

Figure S19. Crude ^{19}F NMR spectrum for 1-fluoro-3-phenylpropane (−220.74 ppm) with hexafluorobenzene (−162.51 ppm, 8.0 mg) and 1,3,5 – trifluorobenzene (−107.16 ppm, 6.9 mg) as internal standards. Data were in accordance to those previously reported.[7]

1-Fluorohexane S2h

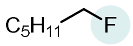

According to general procedure B, *O*-(1-hexyl)-*N*,*N'*-dicyclohexylisourea (30.9 mg, 0.100 mmol) was reacted to give 1-fluorohexane (21%).

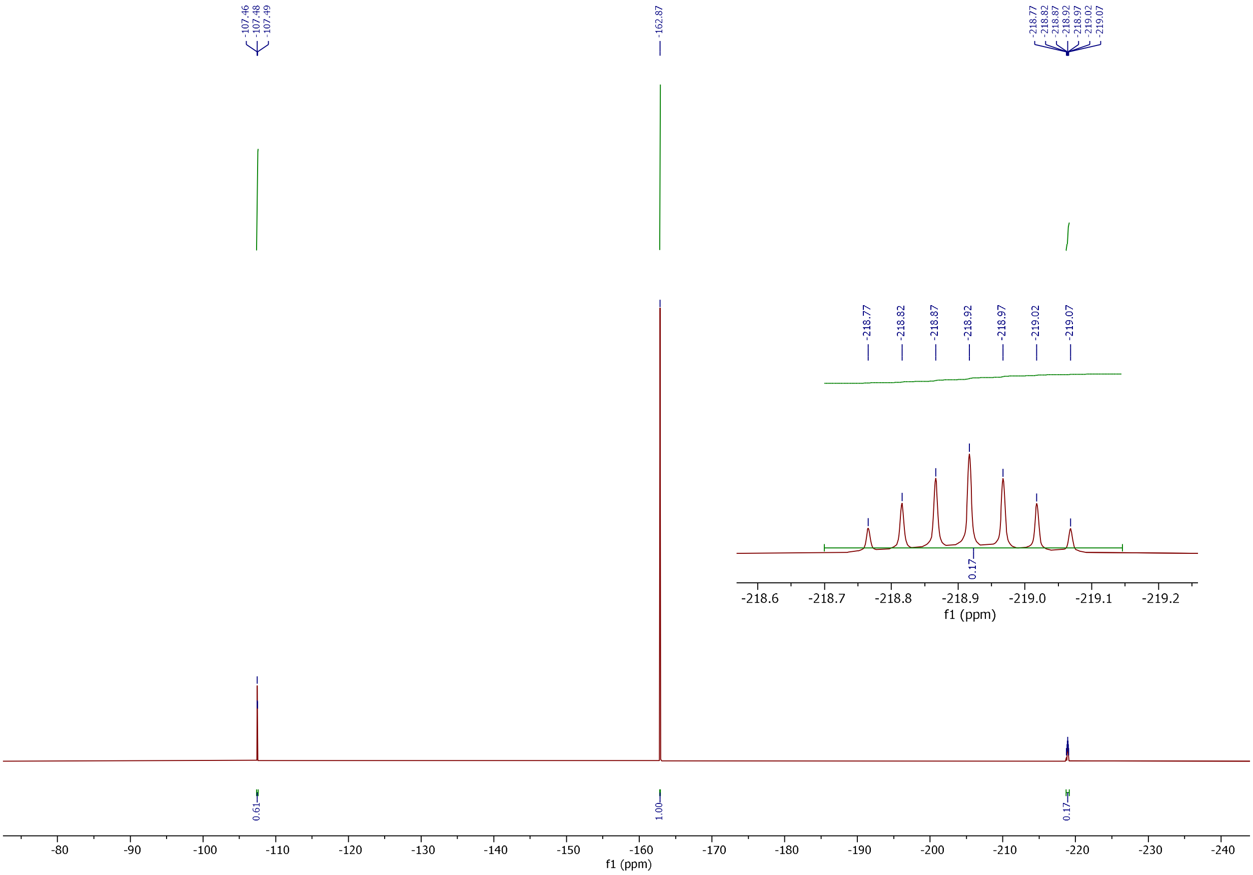

Figure S20. Crude ^{19}F NMR spectrum for 1-fluorohexane (−218.92 ppm) with hexafluorobenzene (−162.87 ppm, 8.5 mg) and 1,3,5 – trifluorobenzene (−107.48 ppm, 6.4 mg) as internal standards. Data were in accordance to those previously reported.[8]

1-(Fluoromethyl)naphthalene S2i

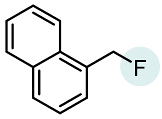

According to general procedure B, *O*-(naphthylmethyl)-*N*,*N′*-dicyclohexylisourea (36.5 mg, 0.100 mmol) was reacted to give 1-(fluoromethyl)naphthalene (57%).

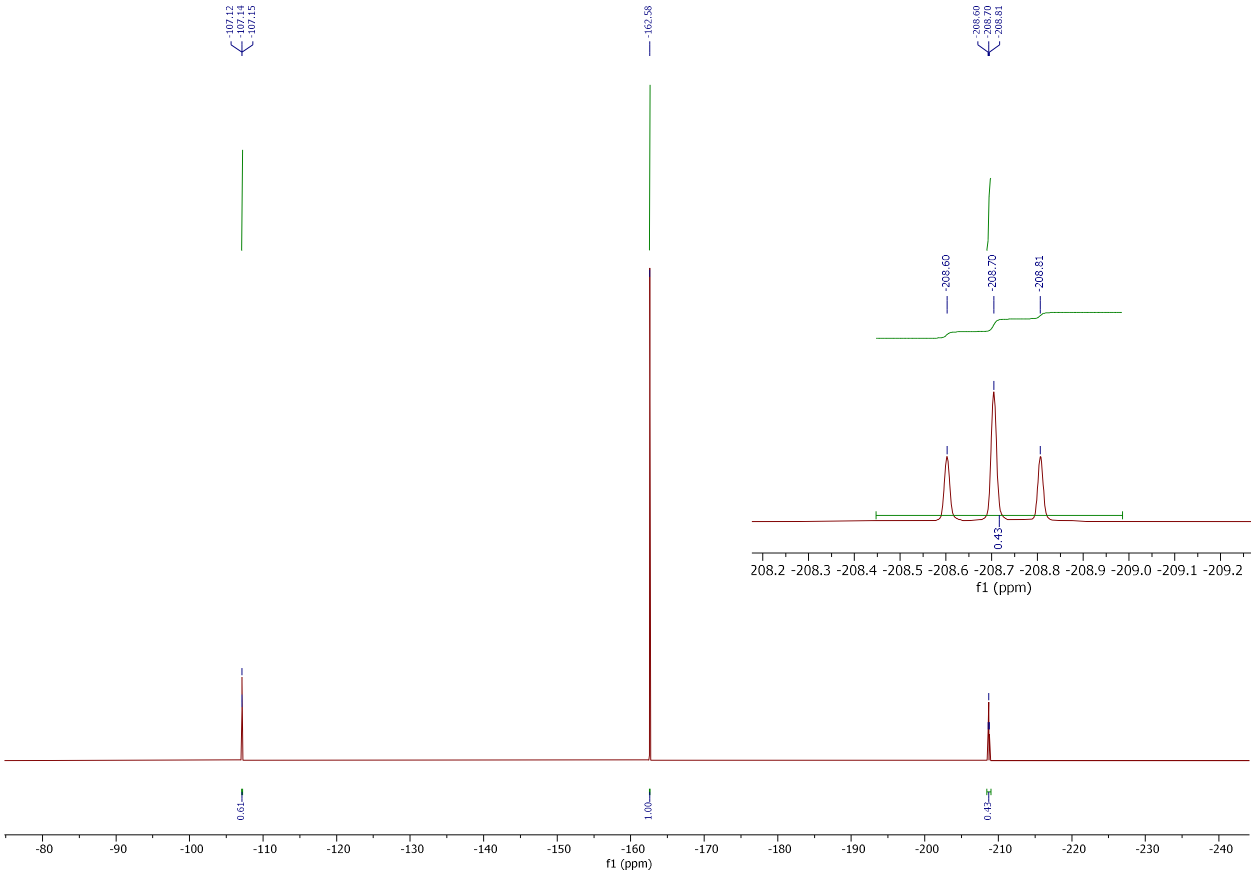

Figure S21. Crude ^{19}F NMR spectrum for 1-(fluoromethyl)naphthalene (−208.70 ppm) with hexafluorobenzene (−162.58 ppm, 8.1 mg) and 1,3,5 – trifluorobenzene (−107.14 ppm, 6.6 mg) as internal standards. Data were in accordance to those previously reported.[10]

Benzyl fluoride S2j

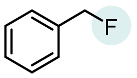

According to general procedure B, *O*-benzyl-*N*,*N'*-dicyclohexylisourea (31.4 mg, 0.100 mmol) was reacted to give benzyl fluoride (40%).

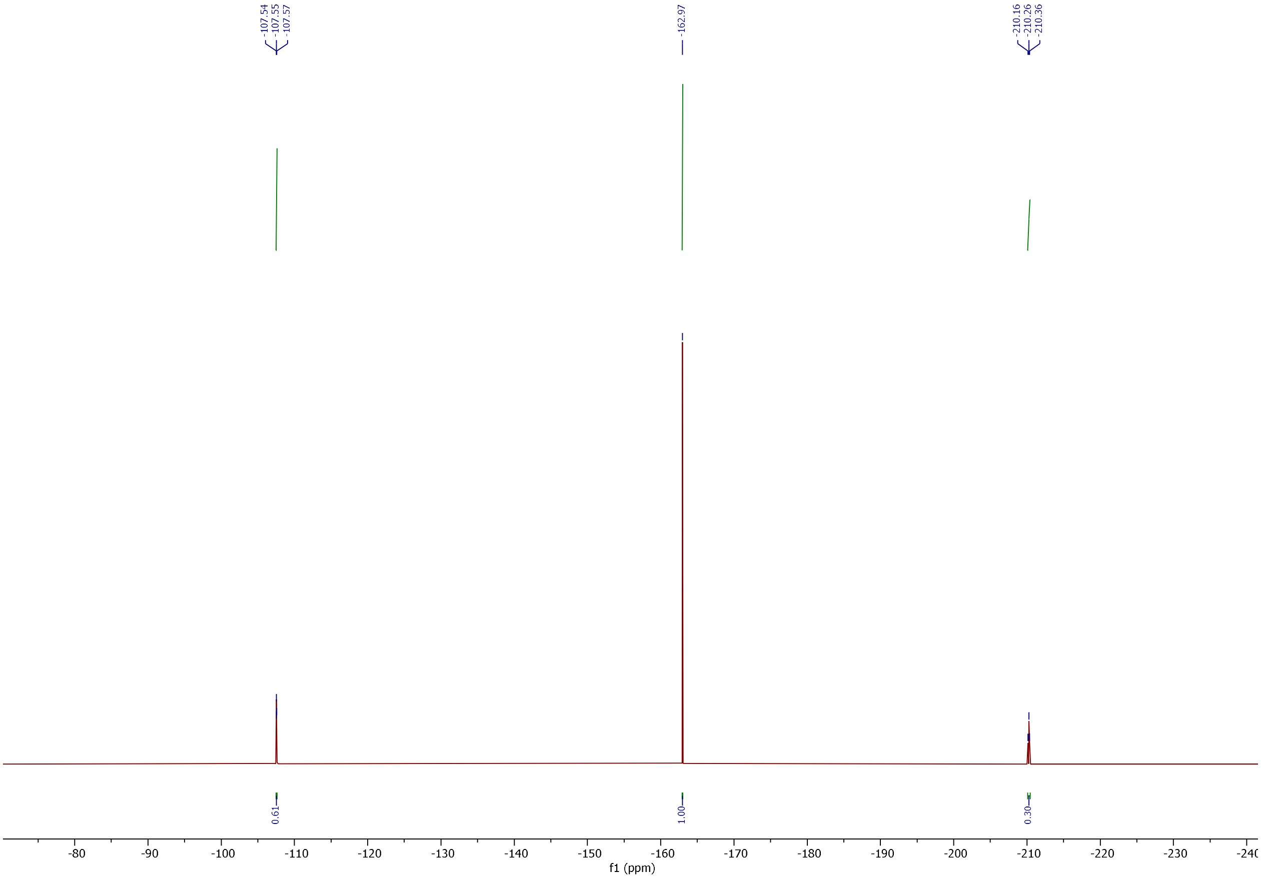

Figure S22. Crude ^{19}F NMR spectrum for benzyl fluoride (−210.26 ppm) with hexafluorobenzene (−162.97 ppm, 8.1 mg) and 1,3,5 – trifluorobenzene (−107.55 ppm, 7.0 mg) as internal standards. Data were in accordance to those previously reported.[9]

1-(Fluoromethyl)-4-(trifluoromethyl)benzene S2k

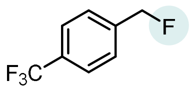

According to general procedure B, *O*-(4-trifluoromethylbenzyl)-*N*,*N'*-dicyclohexylisourea (38.2 mg, 0.100 mmol) was reacted to give 1-(fluoromethyl)-4-(trifluoromethyl)benzene (31%).

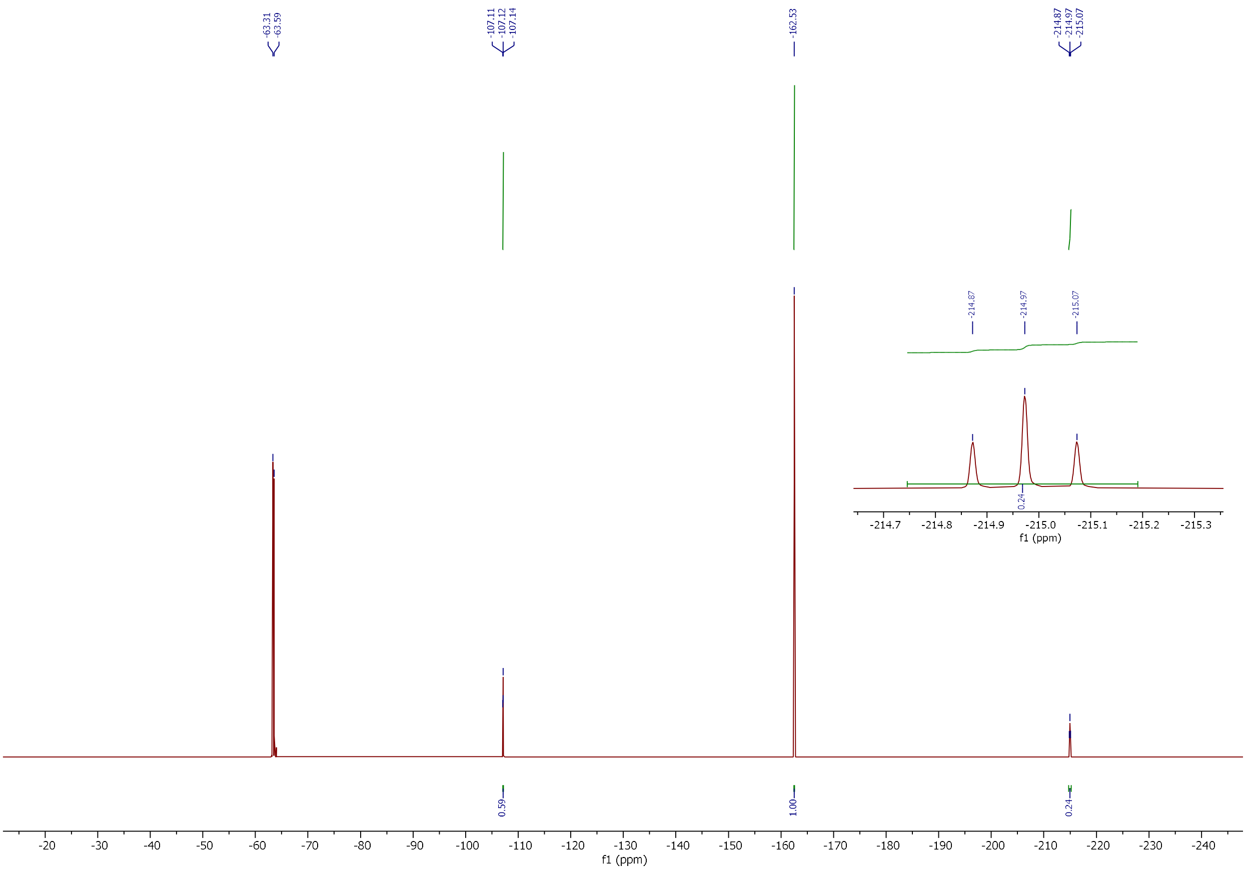

Figure S23. Crude ^{19}F NMR spectrum for 1-(fluoromethyl)-4-(trifluoromethyl)benzene (214.97 ppm) with hexafluorobenzene (−162.53 ppm, 8.1 mg) and 1,3,5 – trifluorobenzene (−107.12 ppm, 6.4 mg) as internal standards. Data were in accordance to those previously reported.[11]

1-Chloro-4-(fluoromethyl)benzene S2l

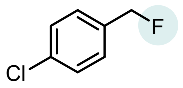

According to general procedure B, *O*-(4-chlorobenzyl)-*N*,*N'*-dicyclohexylisourea (34.9 mg, 0.100 mmol) was reacted to give 1-chloro-4-(fluoromethyl)benzene (31%).

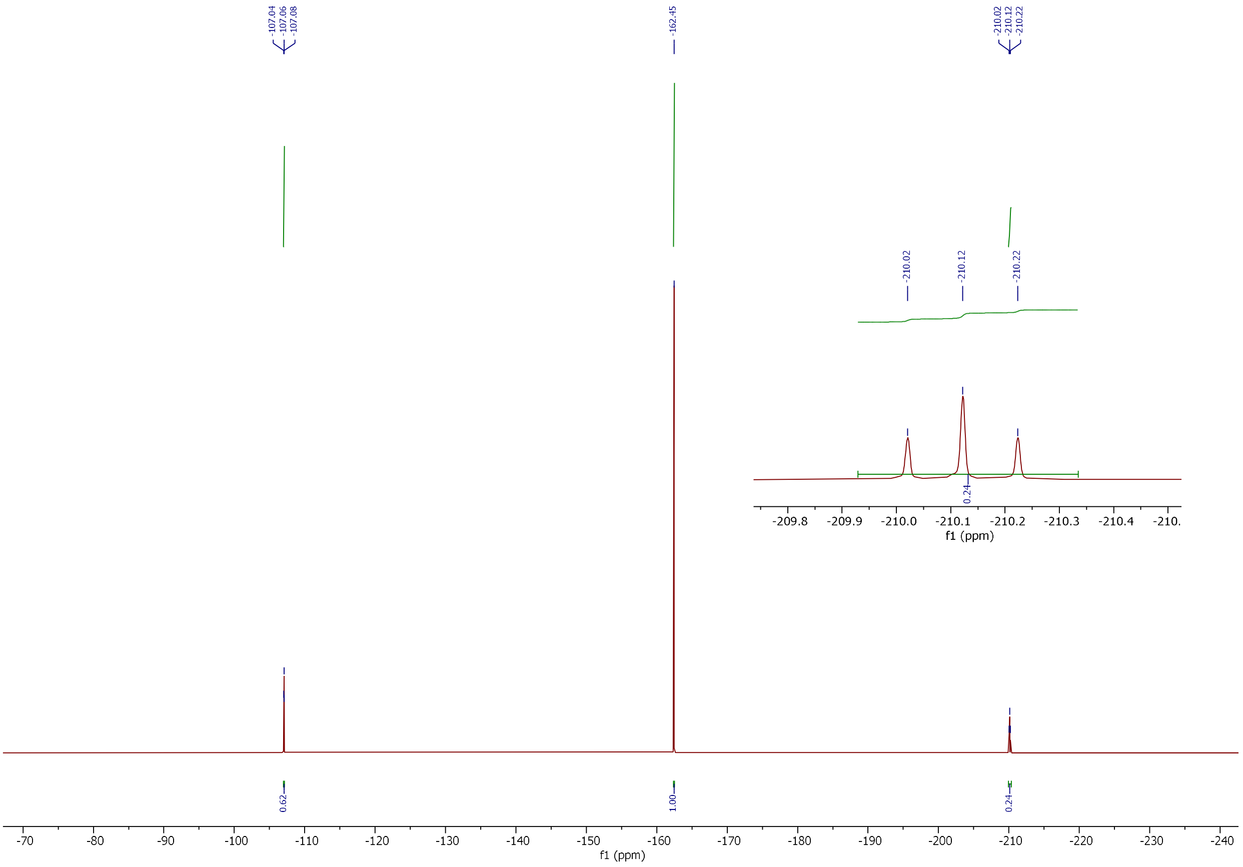

Figure S24. Crude ^{19}F NMR spectrum for 1-chloro-4-(fluoromethyl)benzene (−210.12 ppm) with hexafluorobenzene (−162.45 ppm, 8.1 mg) and 1,3,5 – trifluorobenzene (−107.06 ppm, 6.9 mg) as internal standards. Data were in accordance to those previously reported.[12]

1-Nitro-4-(fluoromethyl)benzene S2m

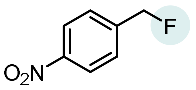

According to general procedure B, *O*-(4-nitrobenzyl)-*N*,*N'*-dicyclohexylisourea (35.9 mg, 0.100 mmol) was reacted to give 1-nitro-4-(fluoromethyl)benzene (32%).

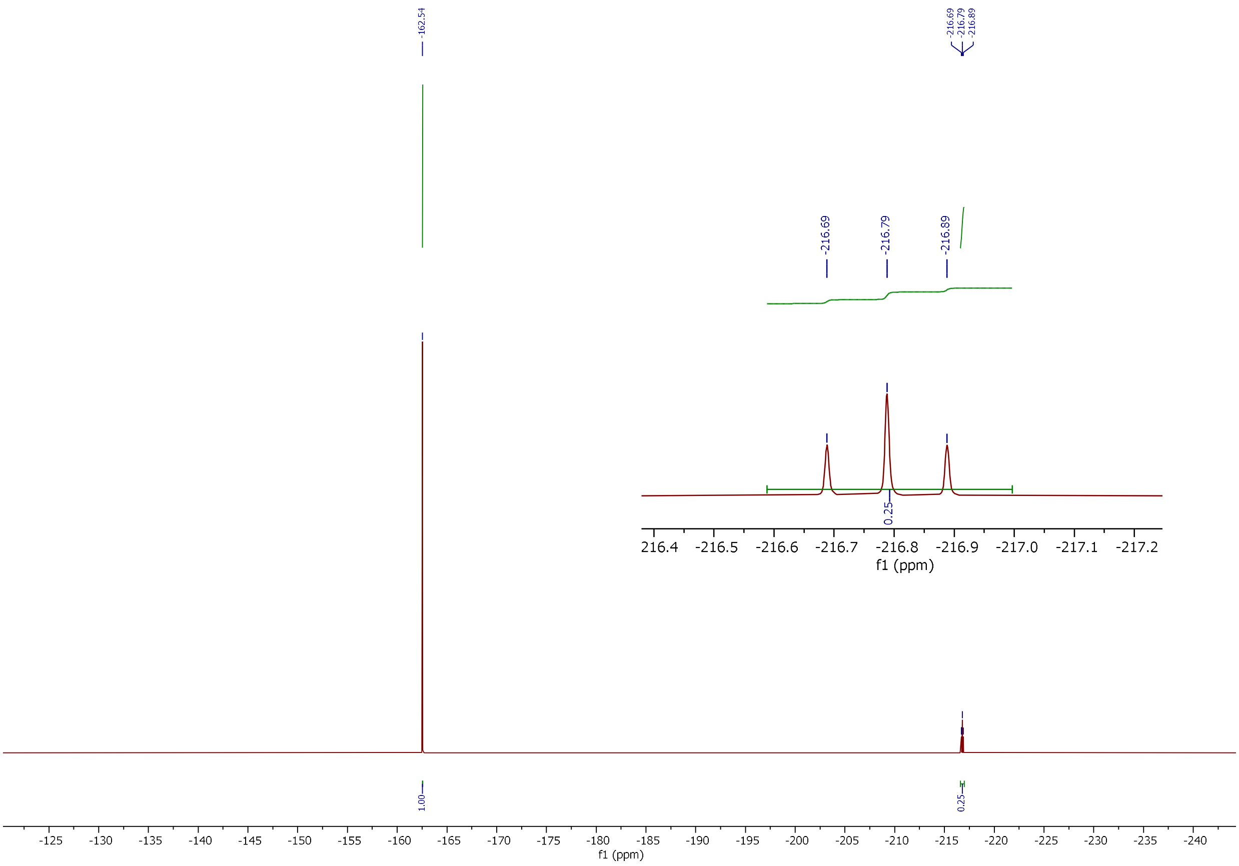

Figure S25. Crude ^{19}F NMR spectrum for 1-nitro-4-(fluoromethyl)benzene (−216.79 ppm) with hexafluorobenzene (−162.54 ppm, 8.2 mg) as internal standard. Data were in accordance to those previously reported.[11]

1-(Fluoromethyl)-4-methoxybenzene S2n

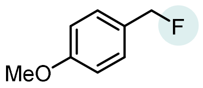

According to general procedure B, *O*-(4-methoxybenzyl)-*N*,*N'*-dicyclohexylisourea (34.5 mg, 0.100 mmol) was reacted to give 1-(fluoromethyl)-4-methoxybenzene (33%).

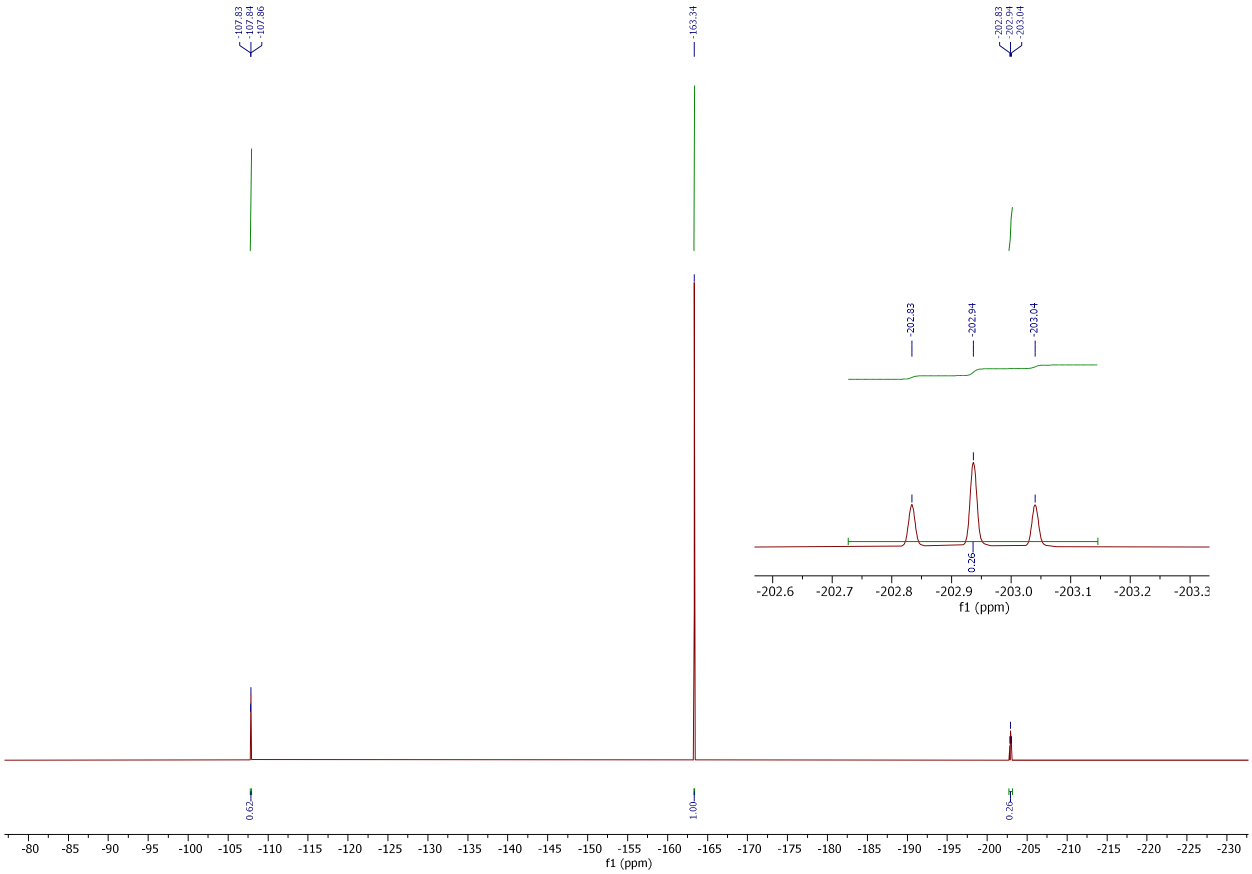

Figure S26. Crude ^{19}F NMR spectrum for 1-(fluoromethyl)-4-methoxybenzene (−209.94 ppm) with hexafluorobenzene (−163.34 ppm, 8.2 mg) and 1,3,5 – trifluorobenzene (−107.84 ppm, 6.6 mg) as internal standards. Data were in accordance to those previously reported.[10]

1-(Fluoromethyl)-4-methylbenzene S2o

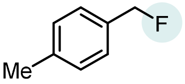

According to general procedure B, *O*-(4-methylbenzyl)-*N*,*N'*-dicyclohexylisourea (32.9 mg, 0.100 mmol) was reacted to give 1-(fluoromethyl)-4-methylbenzene (56%).

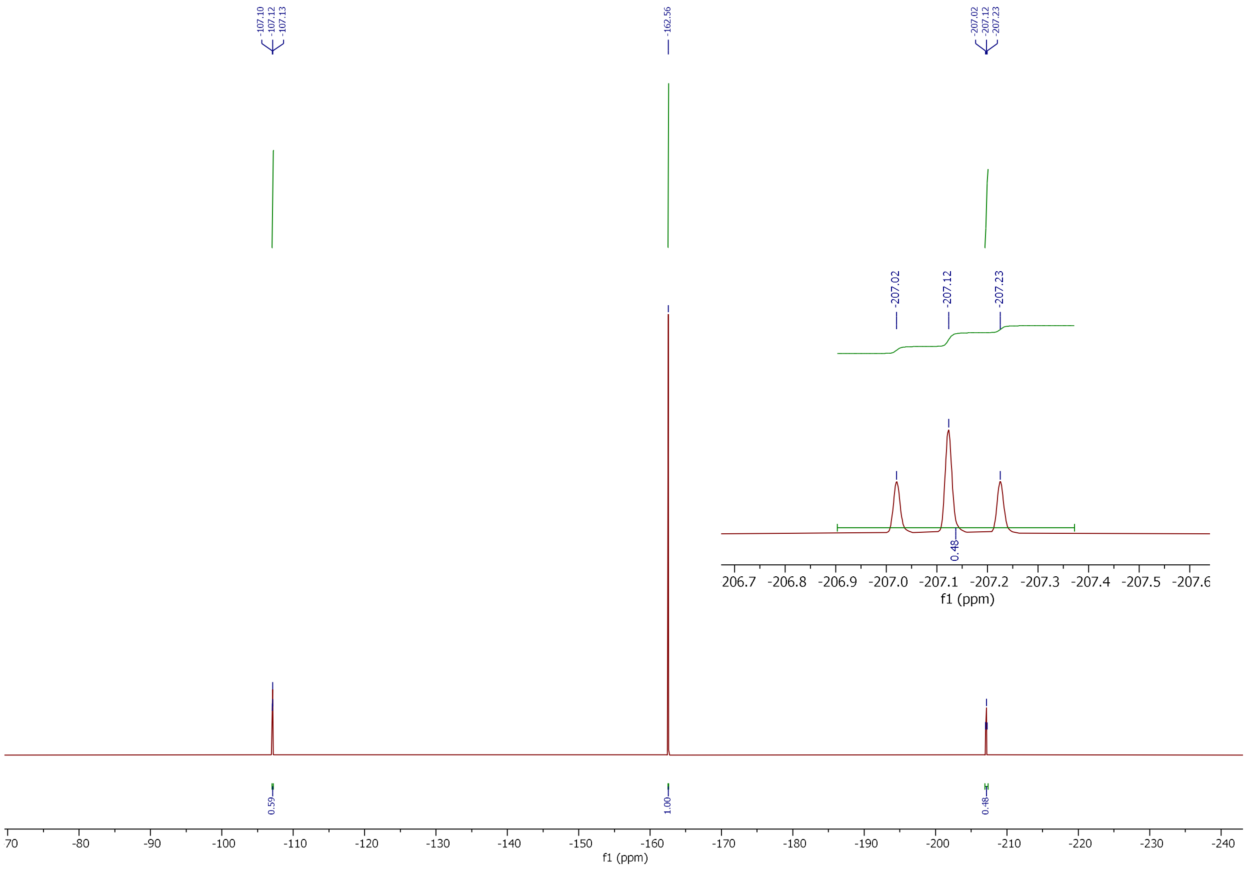

Figure S27. Crude ^{19}F NMR spectrum for 1-(fluoromethyl)-4-methylbenzene (−207.12 ppm) with hexafluorobenzene (−162.56 ppm, 7.3 mg) and 1,3,5 – trifluorobenzene (−107.12 ppm, 6.1 mg) as internal standards. Data were in accordance to those previously reported.[13]

1-(Fluoromethyl)-3-methoxybenzene S2p

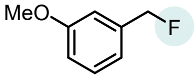

According to general procedure B, *O*-(4-trifluoromethyl-benzyl)-*N*,*N'*-dicyclohexylisourea (34.5 mg, 0.100 mmol) was reacted to give 1-(fluoromethyl)-3-methoxybenzene (56%).

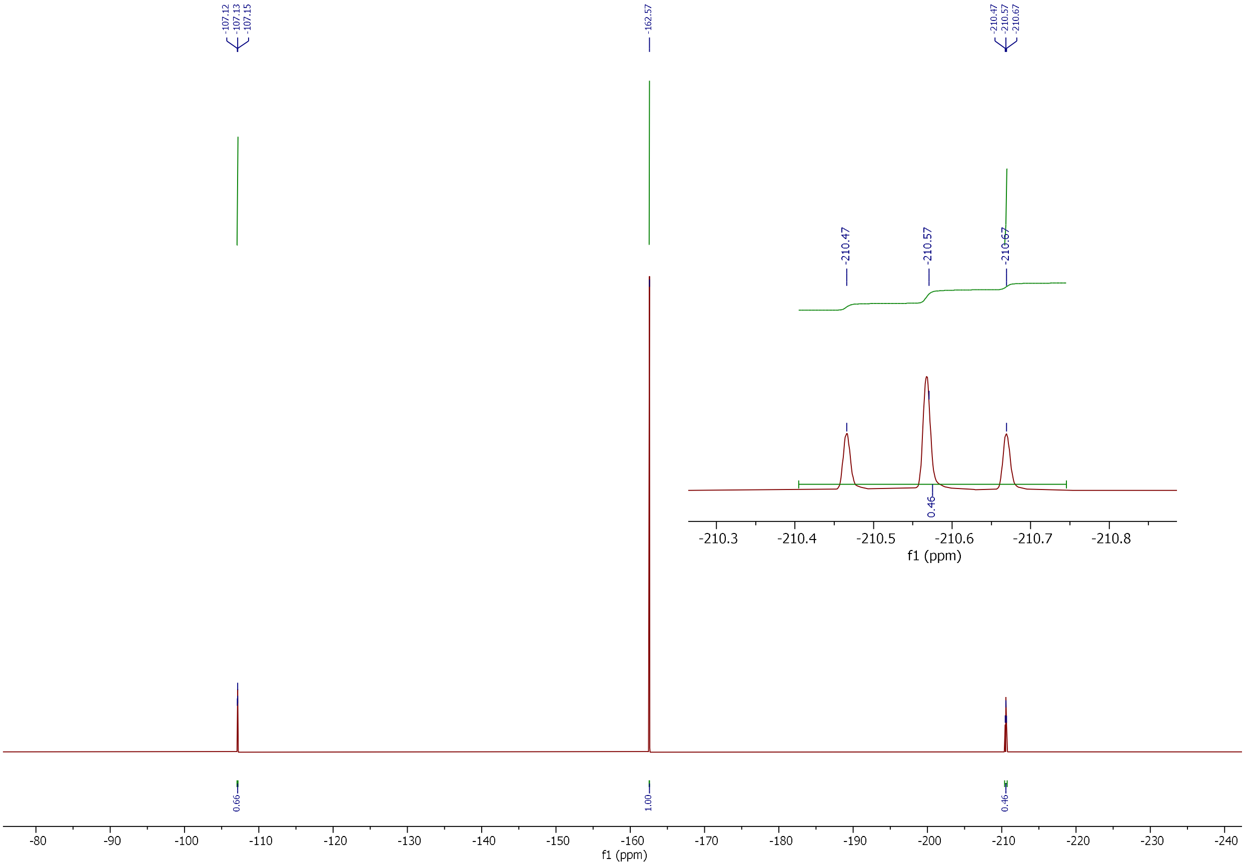

Figure S28. Crude ^{19}F NMR spectrum for 1-(fluoromethyl)-3-methoxybenzene (−210.57 ppm) with hexafluorobenzene (−162.57 ppm, 7.8 mg) and 1,3,5 – trifluorobenzene (−107.13 ppm, 6.8 mg) as internal standards. Data were in accordance to those previously reported.[13]

1-(Fluoromethyl)-2-bromobenzene S2q

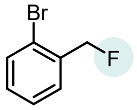

According to general procedure B, *O*-(2-bromobenzyl)-*N*,*N'*-dicyclohexylisourea (39.3 mg, 0.100 mmol) was reacted to give 1-(fluoromethyl)-2-bromobenzene (47%).

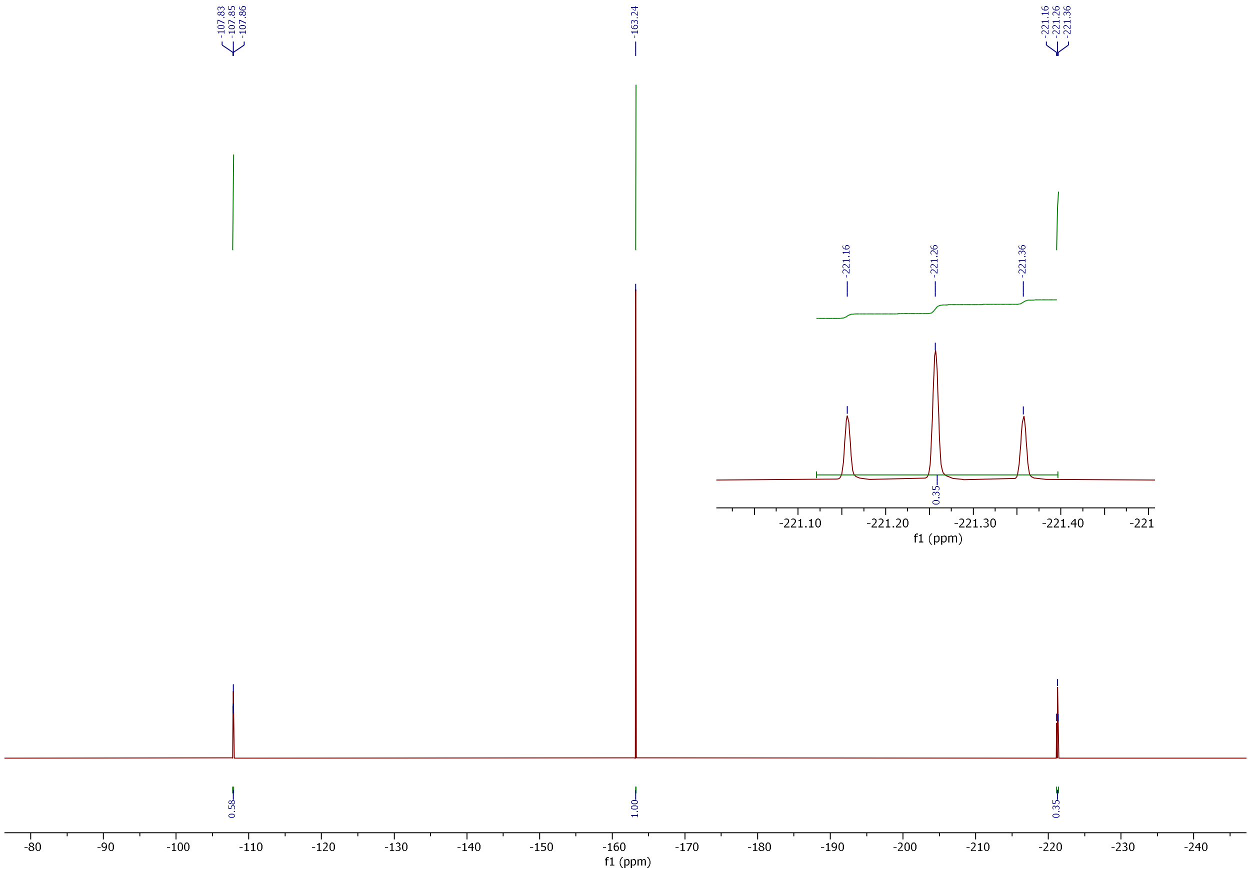

Figure S29. Crude ^{19}F NMR spectrum for 1-(fluoromethyl)-2-bromobenzene (−221.26 ppm) with hexafluorobenzene (−162.24 ppm, 8.8 mg) and 1,3,5 – trifluorobenzene (−107.85 ppm, 6.6 mg) as internal standards. Data were in accordance to those previously reported.[14]

1-Fluoro-3,7-dimethylocta-2*E*,6-diene and 3-fluoro-3,7-dimethylocta-1,6-diene S2r

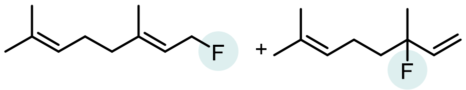

According to general procedure B, *O*-(2-(*E*)-3,7-dimethyl-octa-2,6-dienyl)-*N*,*N'*-dicyclohexylisourea (36.1 mg, 0.100 mmol) was reacted to give 1-fluoro-3,7-dimethylocta-2*E*,6-diene (38%) and 3-fluoro-3,7-dimethylocta-1,6-diene (18%).

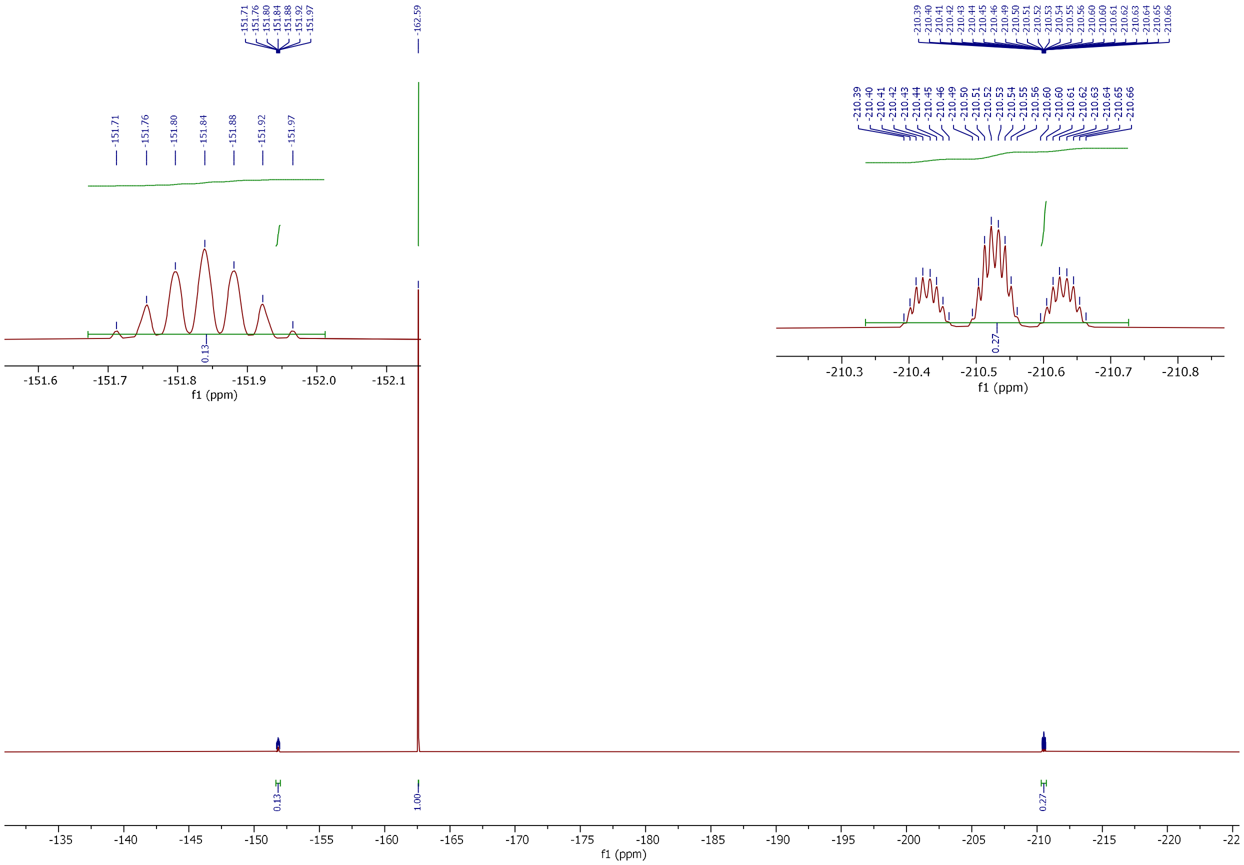

Figure S30. Crude ^{19}F NMR spectrum for 1-fluoro-3,7-dimethylocta-2E,6-diene (−151.84 ppm) and 3-fluoro-3,7-dimethylocta-1,6-diene (−210.52 ppm) with hexafluorobenzene (−162.59 ppm, 8.8 mg) as internal standard. Data were in accordance to those previously reported.[15,16]

4. Secondary-Selective Isourea Fluorination Optimisation

NMR Tube Reactions: Boranes

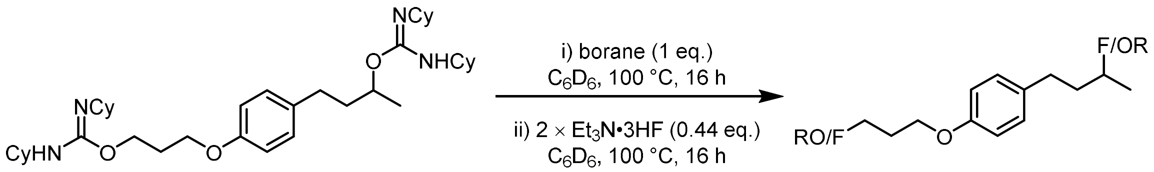

In a J-Young NMR tube, a solution of 4-(4-(3-*O*-(*N*,*N'*-dicyclohexylcarbamimidyl)-propoxy)phenyl)butan-2-*O*-*N*,*N'*-dicyclohexylcarbamimidate (64.0 mg, 0.100 mmol), and borane (0.100 mmol) in C_6D_6 (0.600 mL) was heated at 60 °C for 16 hours. $Et_3N{\cdot}3HF$ (7.0 μL, 44 μmol) was added and the mixture heated at 100 °C for 16 hours, followed by another portion of $Et_3N{\cdot}3HF$ (7.0 μL, 44 μmol) and further heating at 100 °C for 16 hours. The yield was determined using 1,3,5-trifluorobenzene and hexafluorobenzene as internal standards.

Both H-BBN and $HBCy_2$ were found to be selective for secondary fluorination, H-BBN was taken forward due to its commercial availability.

Table S4. Borane screening outcome.

Entry	Borane	2°-F /%	1°-F /%	Ratio
1	H-BBN	57	11	5:1
2	HBpin	14	trace	-
3	$HBCy_2$	49	8	6:1

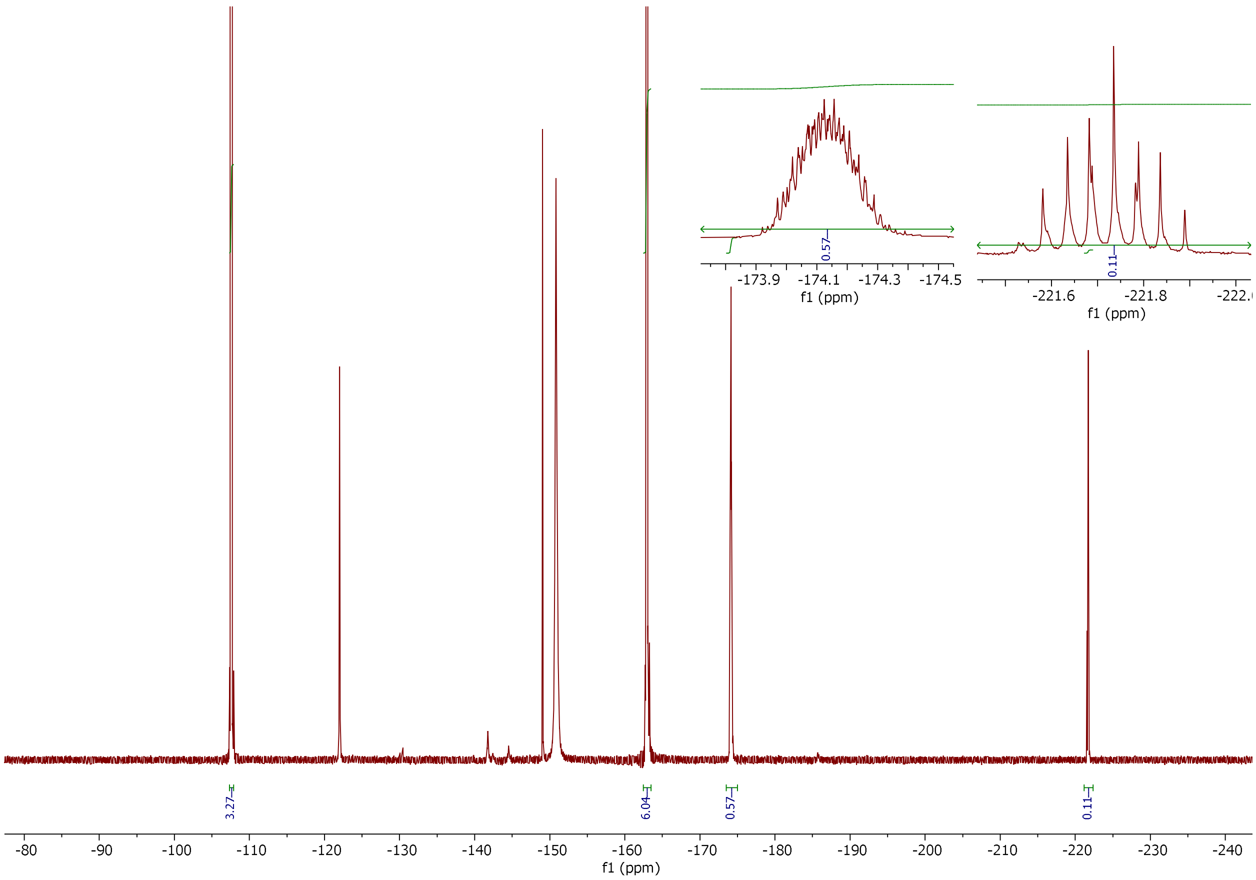

Figure S31. Crude ^{19}F NMR (471 MHz, CH_2Cl_2) for entry 1. Other experiments in this section gave similar spectra with differences in yield of fluorination, thus this spectrum is representative.

NMR Tube Reactions: Step (i) Temperature

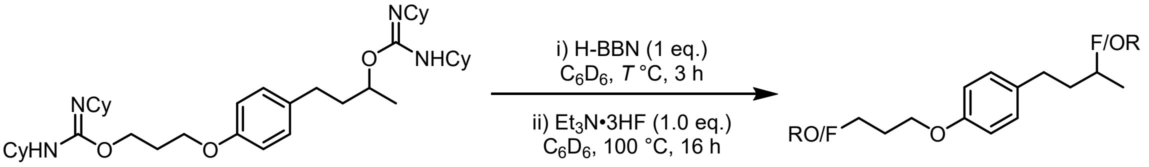

In a J-Young NMR tube, a solution of 4-(4-(3-*O*-(*N*,*N′*-dicyclohexylcarbamimidyl)-propoxy)phenyl)butan-2-*O*-*N*,*N′*-dicyclohexylcarbamimidate (64.0 mg, 0.100 mmol), and H-BBN (12.0 mg, 0.100 mmol) in C_6D_6 (0.600 mL) was heated at *T* °C for 3 hours. $Et_3N{\cdot}3HF$ (16.0 μL, 0.100 mmol) was added and the mixture heated at 100 °C for 16 hours. The yield was determined using 1,3,5-trifluorobenzene and hexafluorobenzene as internal standards.

Higher temperature gave a slight depreciation in yield, and the room temperature reaction showed that the dehydrocoupling step was not complete in three hours (based on ^{11}B NMR spectroscopy showing remaining H-BBN, δ = 28 ppm), so to avoid any potential complications with step 2, 60 °C was taken forward as NMR spectroscopy showed no B−H species remaining.

Table S5. Dehydrocoupling temperature screen outcome.

Entry	*T* / °C	2°-F /%	1°-F /%	Ratio
1	20	64	3	>20:1
2	60	64	3	>20:1
3	100	55	3	18:1

Fluorination Temperature

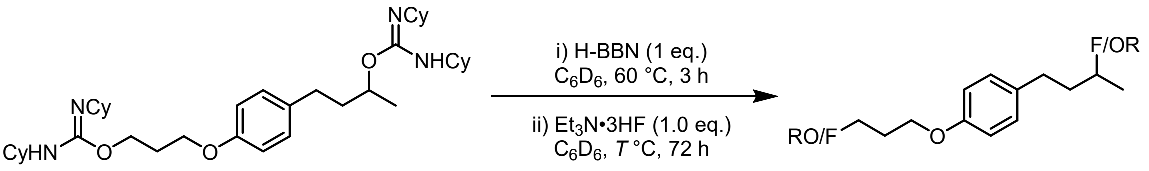

In a catalysis vial, a solution of 4-(4-(3-*O*-(*N*,*N'*-dicyclohexylcarbamimidyl)-propoxy)phenyl)butan-2-*O*-*N*,*N'*-dicyclohexylcarbamimidate (64.0 mg, 0.100 mmol), and H-BBN (12.0 mg, 0.100 mmol) in C_6D_6 (0.600 mL) was heated at 60 °C for 3 hours. Et_3N·3HF (16.0 µL, 0.100 mmol) was added and the mixture heated at *T* °C for 72 hours. The yield was determined using 1,3,5-trifluorobenzene and hexafluorobenzene as internal standards.

For higher throughput and wider applicability, catalysis vials were used in place of J-Young NMR tubes for further optimisation, this led to a drop-off in yield, so optimisation started again from this point. 100 °C was required for fluorination to occur.

Table S6. Fluorination temperature screen outcome.

Entry	*T* / °C	2°-F /%	1°-F /%	Ratio
1	r.t.	0	0	-
2	60	1	0	-
3	100	36	2	18:1

Fluoride Source

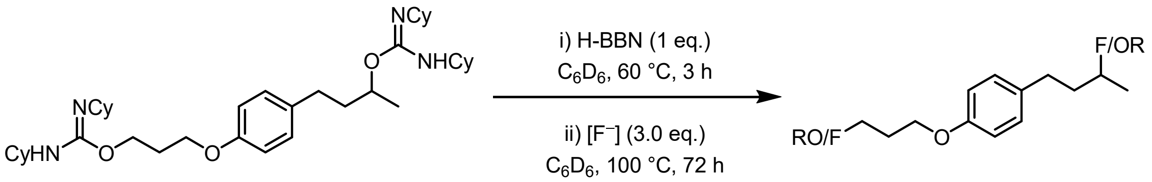

In a catalysis vial, a solution of 4-(4-(3-*O*-(*N*,*N'*-dicyclohexylcarbamimidyl)-propoxy)phenyl)butan-2-*O*-*N*,*N'*-dicyclohexylcarbamimidate (64.0 mg, 0.100 mmol), and H-BBN (12.0 mg, 0.100 mmol) in C_6D_6 (0.600 mL) was heated at 60 °C for 3 hours. [F^-] source (3 eq. fluoride) was added and the mixture heated at 100 °C for 72 hours. The yield was determined using 1,3,5-trifluorobenzene and hexafluorobenzene as internal standards.

Py·HF_x led to significant etching of glassware, which may be the origin of the reduced yield compared to Et_3N·3HF, which highlights the greater ease of handling of the latter.

Table S7. Fluoride source screen.

Entry	[F^-]	2°-F /%	1°-F /%	Ratio
1	TBAF (in THF)	3	1	3:1
2	CsF	0	0	-
3	KF	0	0	-
4	Py·HF_x	14	trace	-
5	KHF_2	0	0	-

Solvent Screen

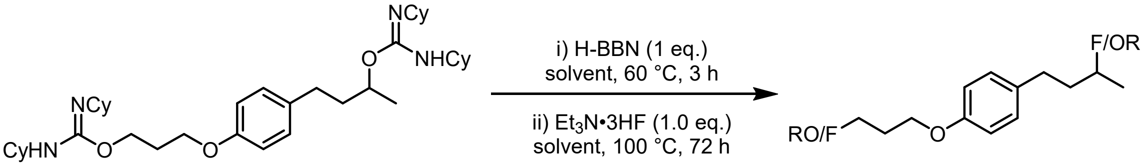

In a catalysis vial, a solution of 4-(4-(3-*O*-(*N*,*N'*-dicyclohexylcarbamimidyl)-propoxy)phenyl)butan-2-*O*-*N*,*N'*-dicyclohexylcarbamimidate (64.0 mg, 0.100 mmol), and H-BBN (12.0 mg, 0.100 mmol) in *solvent* (0.600 mL) was heated at 60 °C for 3 hours. $Et_3N{\cdot}3HF$ (16.0 µL, 0.100 mmol) was added and the mixture heated at 100 °C for 72 hours. The yield was determined using 1,3,5-trifluorobenzene and hexafluorobenzene as internal standards.

Toluene and hexane gave comparable results, due to the lower toxicity of toluene it was taken forward for further optimisation.

Table S8. Solvent screen outcome.

Entry	Solvent	2°-F /%	1°-F /%	Ratio
1	toluene	57	2	>20:1
2	acetonitrile	38	1	>20:1
3	DMF	11	0	-
4	*n*-hexane	59	2	>20:1
5	THF	37	trace	-

Toluene Temperature and Time

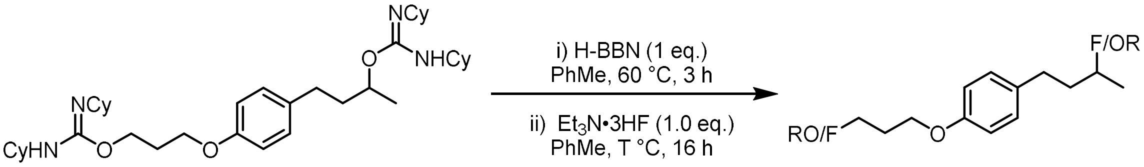

In a catalysis vial, a solution of 4-(4-(3-*O*-(*N*,*N'*-dicyclohexylcarbamimidyl)-propoxy)phenyl)butan-2-*O*-*N*,*N'*-dicyclohexylcarbamimidate (64.0 mg, 0.100 mmol), and H-BBN (12.0 mg, 0.100 mmol) in toluene (0.600 mL) was heated at 60 °C for 3 hours. $Et_3N \cdot 3HF$ (16.0 µL, 0.100 mmol) was added and the mixture heated at *T* °C for 16 hours. The yield was determined using 1,3,5-trifluorobenzene and hexafluorobenzene as internal standards.

Lowering the reaction time gave an improved yield compared to 72 hours at 100 °C. Decreasing the temperature gave a dramatically reduced yield. Increasing the temperature gave a slight increase in yield, but reduced selectivity.

Table S9. Temperature and time screen with toluene as solvent.

Entry	*T* / °C	2°-F /%	1°-F /%	Ratio
1	80	20	1	20:1
2	100	70	5	14:1
3	120	78	19	4:1

Concentration

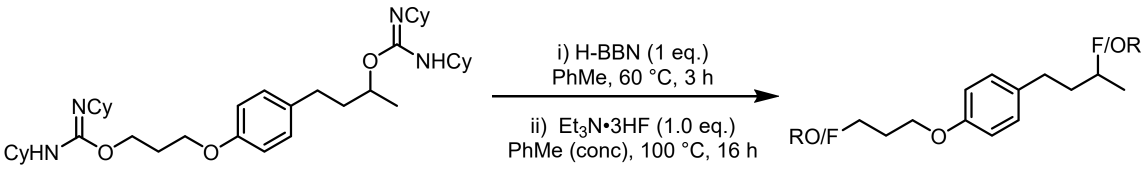

In a catalysis vial, a solution of 4-(4-(3-*O*-(*N*,*N'*-dicyclohexylcarbamimidyl)-propoxy)phenyl)butan-2-*O*-*N*,*N'*-dicyclohexylcarbamimidate (64.0 mg, 0.100 mmol), and H-BBN (12.0 mg, 0.100 mmol) in toluene (*x* mL) was heated at 60 °C for 3 hours. $Et_3N\cdot3HF$ (16.0 µL, 0.100 mmol) was added and the mixture heated at 100 °C for 16 hours. The yield was determined using 1,3,5-trifluorobenzene and hexafluorobenzene as internal standards.

Lower concentration favoured higher selectivity with a slightly decreased yield. 0.1 M was taken forward for further optimisation, but 0.17 M was found to be better when scaled up (0.5 mmol scale, see below).

Table S10. Concentration screen outcome.

Entry	Conc. / M	2°-F /%	1°-F /%	Ratio
1	1.00	63	6	11:1
2	0.50	73	9	8:1
3	0.17	70	5	14:1
4	0.10	67	4	17:1

H-BBN Equivalents

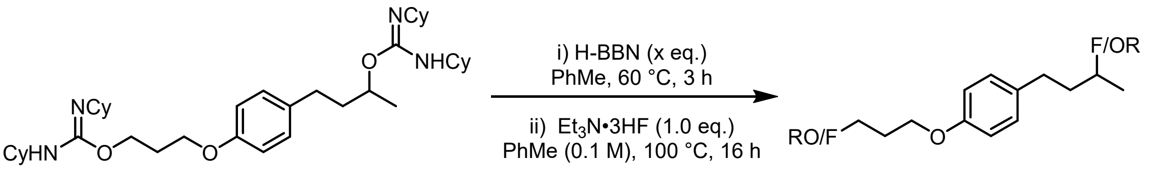

In a catalysis vial, a solution of 4-(4-(3-*O*-(*N*,*N′*-dicyclohexylcarbamimidyl)-propoxy)phenyl)butan-2-*O*-*N*,*N′*-dicyclohexylcarbamimidate (64.0 mg, 0.100 mmol), and H-BBN (*x* eq.) in toluene (1.00 mL) was heated at 60 °C for 3 hours. $Et_3N \cdot 3HF$ (16.0 µL, 0.100 mmol) was added and the mixture heated at 100 °C for 16 hours. The yield was determined using 1,3,5-trifluorobenzene and hexafluorobenzene as internal standards.

As H-BBN equivalents increase, the selectivity increases but the yield decreases. One equivalent was found to be the optimal compromise.

Table S11. H-BBN equivalence screen outcome.

Entry	Equiv.	2°-F /%	1°-F /%	Ratio
1	0	66	21	3:1
2	0.5	66	13	5:1
3	0.8	66	7	9:1
4	1.0	67	4	17:1
5	1.2	61	3	20:1
6	1.5	46	3	15:1
7	2.0	43	2	22:1

$Et_3N·3HF$ Equivalents

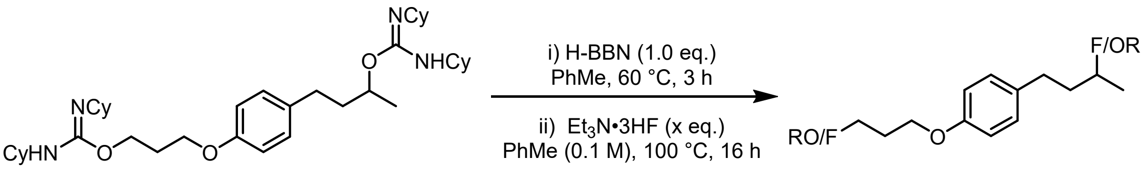

In a catalysis vial, a solution of 4-(4-(3-*O*-(*N*,*N'*-dicyclohexylcarbamimidyl)-propoxy)phenyl)butan-2-*O*-*N*,*N'*-dicyclohexylcarbamimidate (64.0 mg, 0.100 mmol), and H-BBN (12.0 mg, 0.100 mmol) in toluene (1.00 mL) was heated at 60 °C for 3 hours. $Et_3N·3HF$ (*x* eq.) was added and the mixture heated at 100 °C for 16 hours. The yield was determined using 1,3,5-trifluorobenzene and hexafluorobenzene as internal standards.

As $Et_3N·3HF$ equivalents increase, the selectivity decreases but the yield increases. One equivalent was found to be the optimal compromise.

Table S12. $Et_3N·3HF$ equivalence screen outcome.

Entry	Equiv.	2°-F /%	1°-F /%	Ratio
1	0.33	13	0	-
2	1.0	67	4	17:1
3	1.5	62	10	6:1
4	2.0	67	12	6:1
5	3.0	60	11	5:1

Isourea (0.1 mmol)

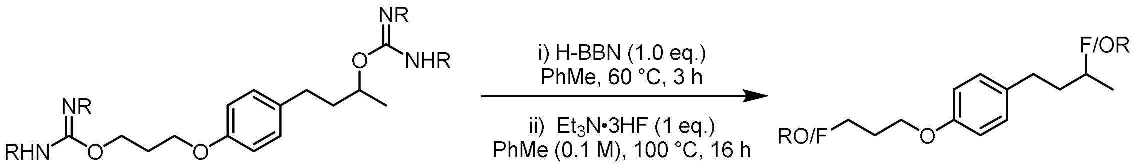

In a catalysis vial, a solution of *bis*-isourea (0.100 mmol), and H-BBN (12.0 mg, 0.100 mmol) in toluene (1.00 mL) was heated at 60 °C for 3 hours. $Et_3N\cdot 3HF$ (16.0 μL, 0.100 mmol) was added and the mixture heated at 100 °C for 16 hours. The yield was determined using 1,3,5-trifluorobenzene and hexafluorobenzene as internal standards.

Table S13. isopropyl vs cyclohexyl isourea screen at 0.1 mmol scale.

**Entry**	**R**	**2°-F /%**	**1°-F /%**	**Ratio**
1	Cy	67	4	17:1
2	^{i}Pr	77	5	15:1

Isourea (0.5 mmol)

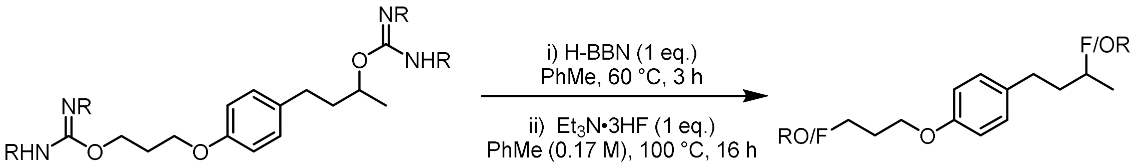

In a J-Young Schlenk, a solution of *bis*-isourea (0.500 mmol), and H-BBN (61.0 mg, 0.500 mmol) in toluene (3.00 mL) was heated at 60 °C for 3 hours. $Et_3N{\cdot}3HF$ (81.0 µL, 0.500 mmol) was added and the mixture heated at 100 °C for 16 hours. The yield was determined using 1,3,5-trifluorobenzene and hexafluorobenzene as internal standards.

Scaling up the reaction gave generally slightly poorer yield and selectivity, but again the isopropyl *bis*-isourea gave better yield and selectivity then the cyclohexyl. On both scales the isopropyl *bis*-isourea gave a greater yield than the cyclohexyl, with comparable selectivity, so was taken forward.

Table S14. isopropyl vs cyclohexyl isourea screen at 0.5 mmol scale.

Entry	R	2°-F /%	1°-F /%	Ratio
1	Cy	60	9	7:1
2	^{i}Pr	67	5	13:1

5. “One-pot” Secondary-Selective Alcohol Deoxyfluorination Optimisation

Note: all one-pot reactions are performed under ambient atmosphere.

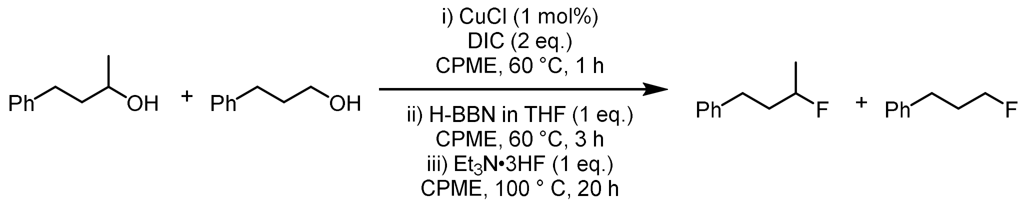

A solution of 4-phenylbutan-2-ol (75.0 mg, 0.500 mmol), 3-phenylpropan-1-ol (70.0 mg, 0.500 mmol), di*iso*propyl carbodiimide (165 µL, 1.00 mmol), and copper(I) chloride (1 mg, 5 µmol) in cyclopentyl methyl ether (1.00 mL) was stirred in a sealed vessel under air at 60 °C for one hour. The mixture was allowed to cool to room temperature and H-BBN (1.00 mL, 0.5 M in THF, 0.500 mmol) added in a single portion (caution: H_2 generation), and the mixture stirred at 60 °C for three hours. The mixture was allowed to cool to room temperature, $Et_3N{\cdot}3HF$ (81.0 µL, 0.500 mmol) was added in a single portion, and the mixture stirred in a sealed vessel at 100 °C for 20 hours. After cooling to room temperature, the mixture was diluted with dichloromethane (10 mL) and the *in situ* yield measured by ^{19}F NMR spectroscopy using 1,3,5-trifluorobenzene and hexafluorobenzene as internal standards.

To translate the reaction to a one-pot procedure directly from alcohols, the process was re-optimised. Watson’s one-pot deoxyfluorination procedure using CPME[17] was taken as a starting point, as toluene was found to be a poor solvent for isourea formation and gave lower secondary-selectivity under these conditions (entry 2). Using solid H-BBN vs H-BBN in THF gave minimal difference in yield and selectivity, so the THF solution was favoured due to ease of use (entry 3). Increasing DIC equivalents or the time of the isourea formation led to poorer yields and selectivity (entries 4 and 5).

Table S15. One-pot alcohol deoxyfluorination optimisation.

Entry	Deviation	2°-F /%	1° /%	ratio 2°:1°
1	None	76	5	15:1
2	Toluene instead of CPME	51	12	4:1
3	H-BBN solid	72	9	8:1
4	3 eq. DIC	63	8	8:1
5	Step i) 2 h	67	7	10:1

6. Intermolecular Deoxyfluorination Competitions

Note: all reactions in this section are run under ambient atmosphere with no special precautions to exclude air/moisture.

General Procedure C:

A solution of alcohol 1 (0.500 mmol), alcohol 2 (0.500 mmol), di*iso*propyl carbodiimide (165 µL, 1.00 mmol), and copper(I) chloride (1 mg, 5 µmol) in cyclopentyl methyl ether (1.00 mL) was stirred in a sealed vessel at 60 °C for one hour. The mixture was allowed to cool to room temperature and H-BBN (1.00 mL, 0.5 M in THF, 0.500 mmol) added in a single portion (caution: H_2 generation), and the mixture stirred at 60 °C for three hours. The mixture was allowed to cool to room temperature, $Et_3N{\cdot}3HF$ (81.0 µL, 0.500 mmol) was added in a single portion, and the mixture stirred in a sealed vessel at 100 °C for 20 hours. After cooling to room temperature, the mixture was diluted with dichloromethane (10 mL) and the *in situ* yield measured by ^{19}F NMR spectroscopy using 1,3,5-trifluorobenzene and hexafluorobenzene as internal standards.

General Procedure D:

A solution of alcohol 1 (0.500 mmol), alcohol 2 (0.500 mmol), di*iso*propyl carbodiimide (165 µL, 1.00 mmol), and copper(I) chloride (1 mg, 5 µmol) in cyclopentyl methyl ether (1.00 mL) was stirred in a sealed vessel at 60 °C for four hours. The mixture was allowed to cool to room temperature, $Et_3N{\cdot}3HF$ (81.0 µL, 0.500 mmol) was added in a single portion, and the mixture stirred in a sealed vessel at 100 °C for 20 hours. After cooling to room temperature, the mixture was diluted with dichloromethane (10 mL) and the *in situ* yield measured by ^{19}F NMR spectroscopy using 1,3,5-trifluorobenzene and hexafluorobenzene as internal standards.

General Procedure E:

A solution of alcohol 1 (0.500 mmol), alcohol 2 (0.500 mmol), di*iso*propyl carbodiimide (165 µL, 1.00 mmol), and copper(I) chloride (1 mg, 5 µmol) in cyclopentyl methyl ether (1.00 mL) was stirred in a sealed vessel at 60 °C for one hour. The mixture was allowed to cool to room temperature and H-BBN (1.00 mL, 0.5 M in THF, 0.500 mmol) added in a single portion (caution: H_2 generation), and the mixture stirred at 60 °C for three hours. The mixture then was passed through a plug of neutral alumina (*ca.* 1 g, EtOAc), concentrated *in vacuo* and then re-dissolved in cyclopentyl methyl ether (1.00 ml). To this was added $Et_3N{\cdot}3HF$ (81.0 µL, 0.500 mmol) in a single portion, and the mixture stirred in a sealed vessel at 100 °C for 20 hours. After cooling to room temperature, the mixture was diluted with dichloromethane (10 mL) and the *in situ* yield measured by ^{19}F NMR spectroscopy using 1,3,5-trifluorobenzene and hexafluorobenzene as internal standards.

Note: the ^{19}F NMR resonances at −150 ppm and −127 ppm seen in all spectra correspond to unidentified species from the background reaction of $Et_3N{\cdot}3HF$ with glass, confirmed independently by heating $Et_3N{\cdot}3HF$ in toluene at 100 °C for 16 hours.

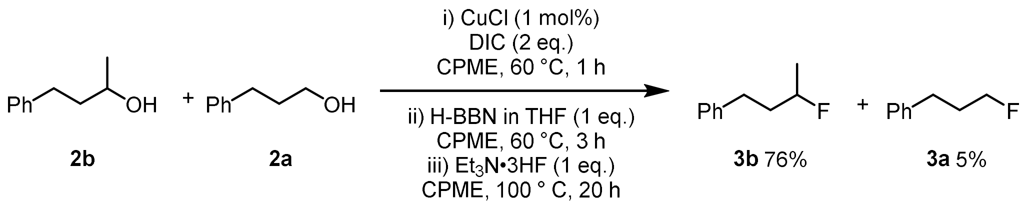

According to general procedure C, 4-phenylbutan-2-ol (75.0 mg, 0.500 mmol) and 3-phenylpropan-1-ol (70.0 mg, 0.500 mmol) were reacted to give 2-fluoro-4-phenylbutane (76%) and 1-fluoro-3-phenylpropane (5%).

^{19}F NMR (565 MHz, CH_2Cl_2) δ −174.6 (m, 0.76F), −220.5 (tt, J = 47.3, 24.9 Hz, 0.05F).

The data were in accordance with those previously reported.[7,18]

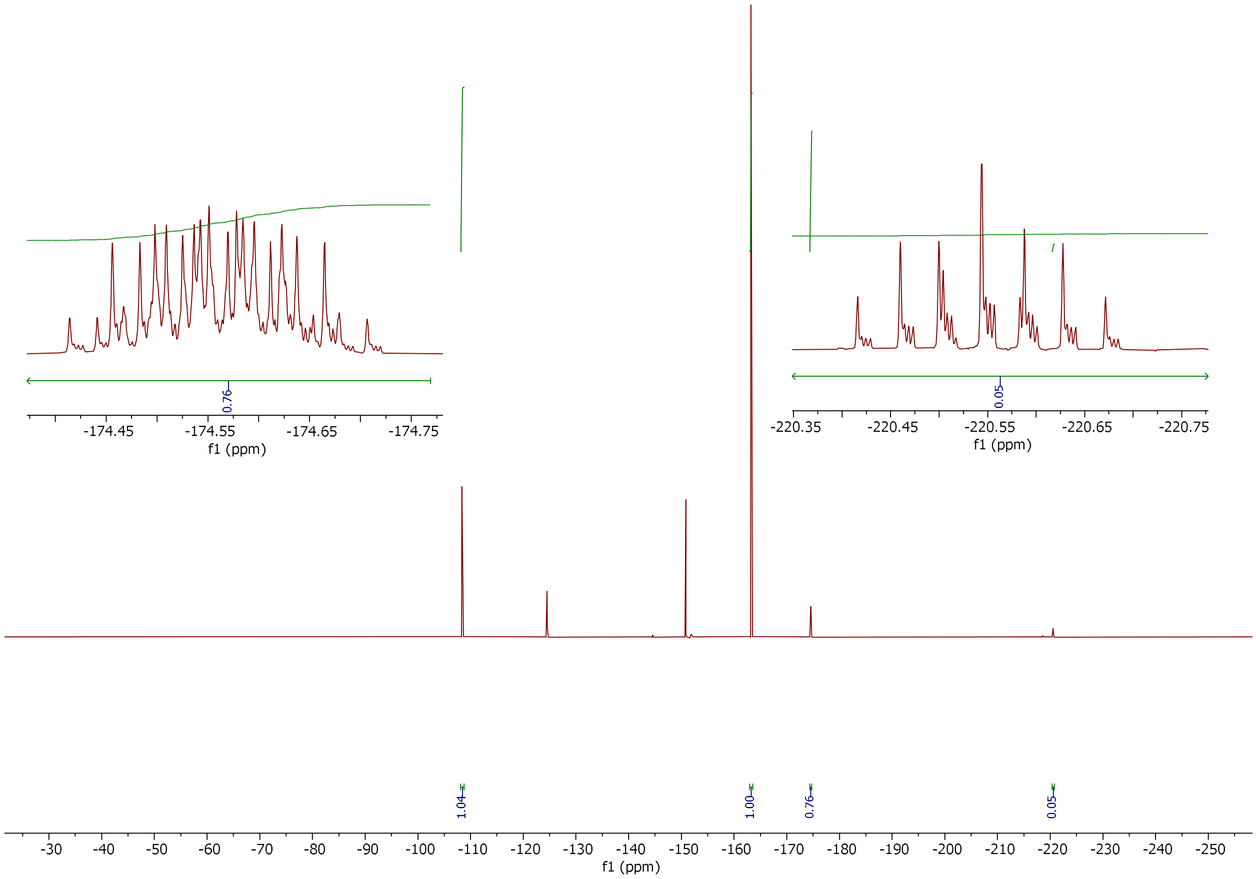

Figure S32. Crude ^{19}F NMR (565 MHz, CH_2Cl_2) spectrum showing competition between **2a** and **2b**.

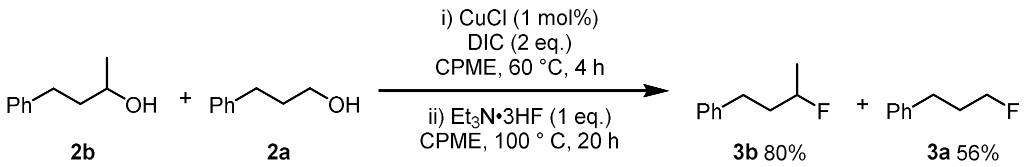

According to general procedure D, 4-phenylbutan-2-ol (75.0 mg, 0.500 mmol) and 3-phenylpropan-1-ol (70.0 mg, 0.500 mmol) were reacted to give 2-fluoro-4-phenylbutane (80%) and 1-fluoro-3-phenylpropane (56%).

^{19}F NMR (471 MHz, CH_2Cl_2) δ −174.1 (m, 0.80F), −220.1 (m, 0.56F).

The data were in accordance with those previously reported.[7,18]

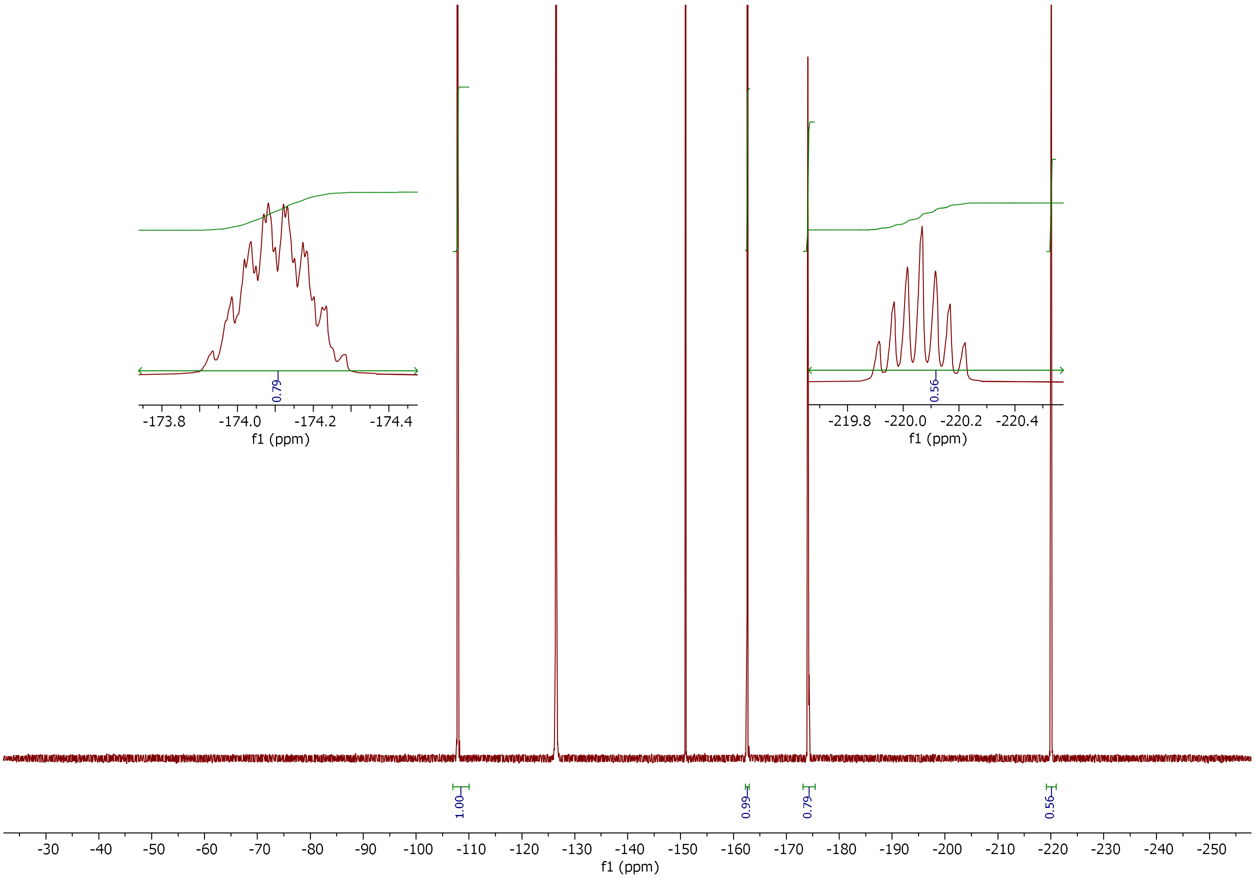

Figure S33. Crude ^{19}F NMR (471 MHz, CH_2Cl_2) spectrum showing competition between **2a** and **2b**.

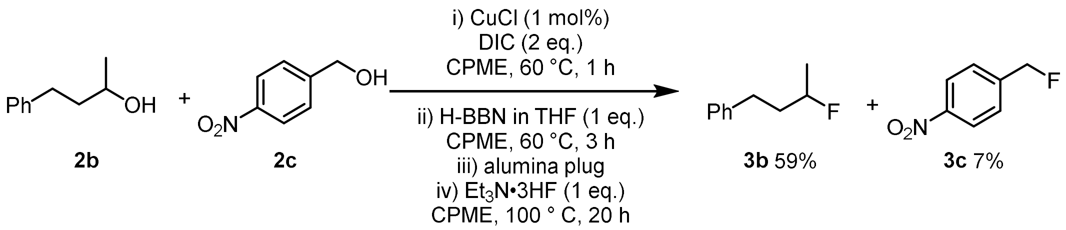

According to general procedure E, 4-phenylbutan-2-ol (75.0 mg, 0.500 mmol) and 4-nitrobenzyl alcohol (77.0 mg. 0.500 mmol) were reacted to give 2-fluoro-4-phenylbutane (59%) and 4-nitrobenzyl fluoride (7%).

^{19}F NMR (565 MHz, CH_2Cl_2) δ −174.1 (m, 0.59F), −215.7 (m, 0.07F).

The data were in accordance with those previously reported.[10]

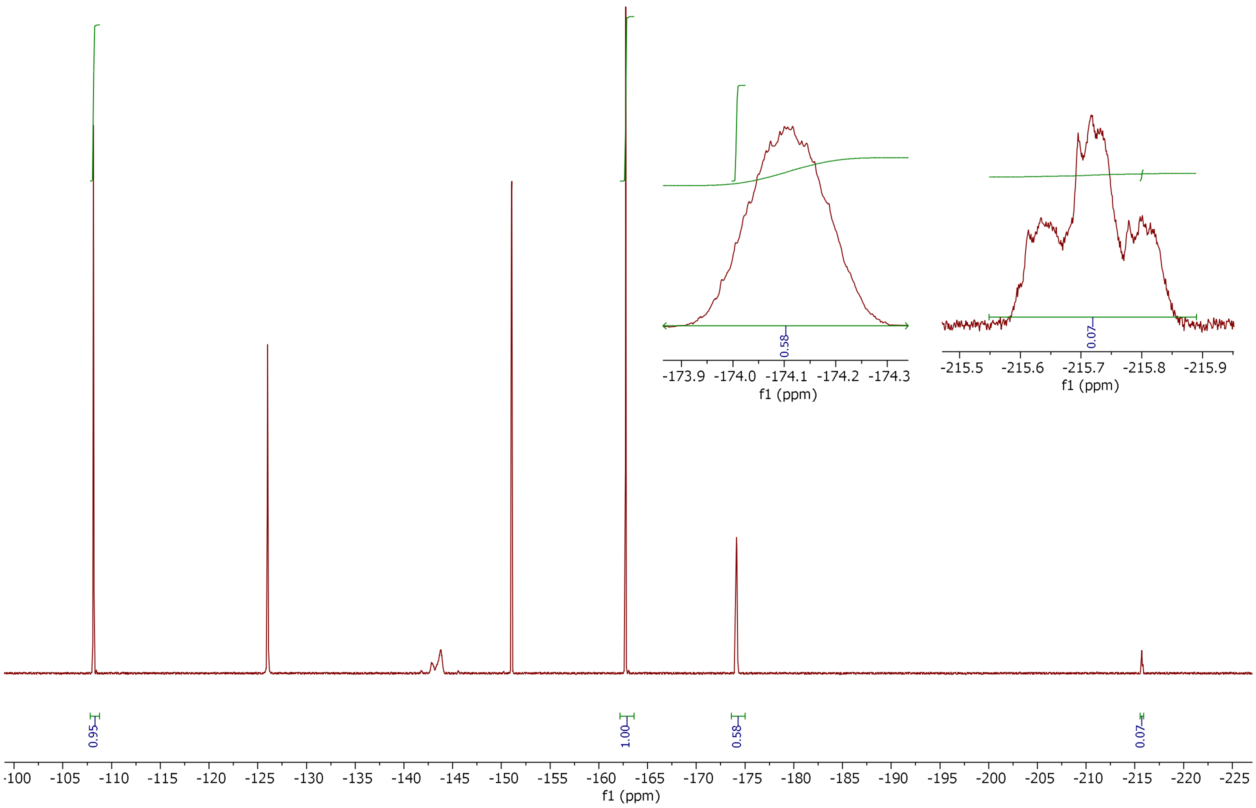

Figure S34. Crude ^{19}F NMR (565 MHz, CH_2Cl_2) spectrum showing competition between **2c** and **2b**.

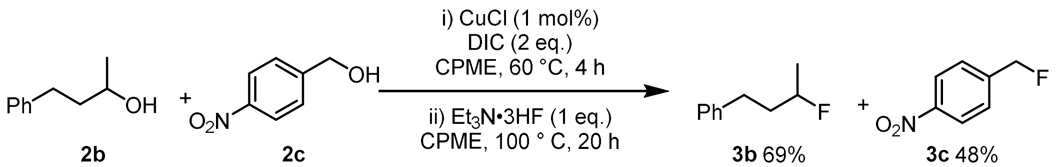

2b **2c** **3b** 69% **3c** 48%

According to general procedure D, 4-phenylbutan-2-ol (75.0 mg, 0.500 mmol) and 4-nitrobenzyl alcohol (77.0 mg. 0.500 mmol) were reacted to give 2-fluoro-4-phenylbutane (69%) and 4-nitrobenzyl fluoride (48%).

^{19}F NMR (471 MHz, CH_2Cl_2) δ −174.1 (m, 0.69F), −215.7 (td, J = 46.4, 5.3 Hz, 0.48F).

The data were in accordance with those previously reported.[10]

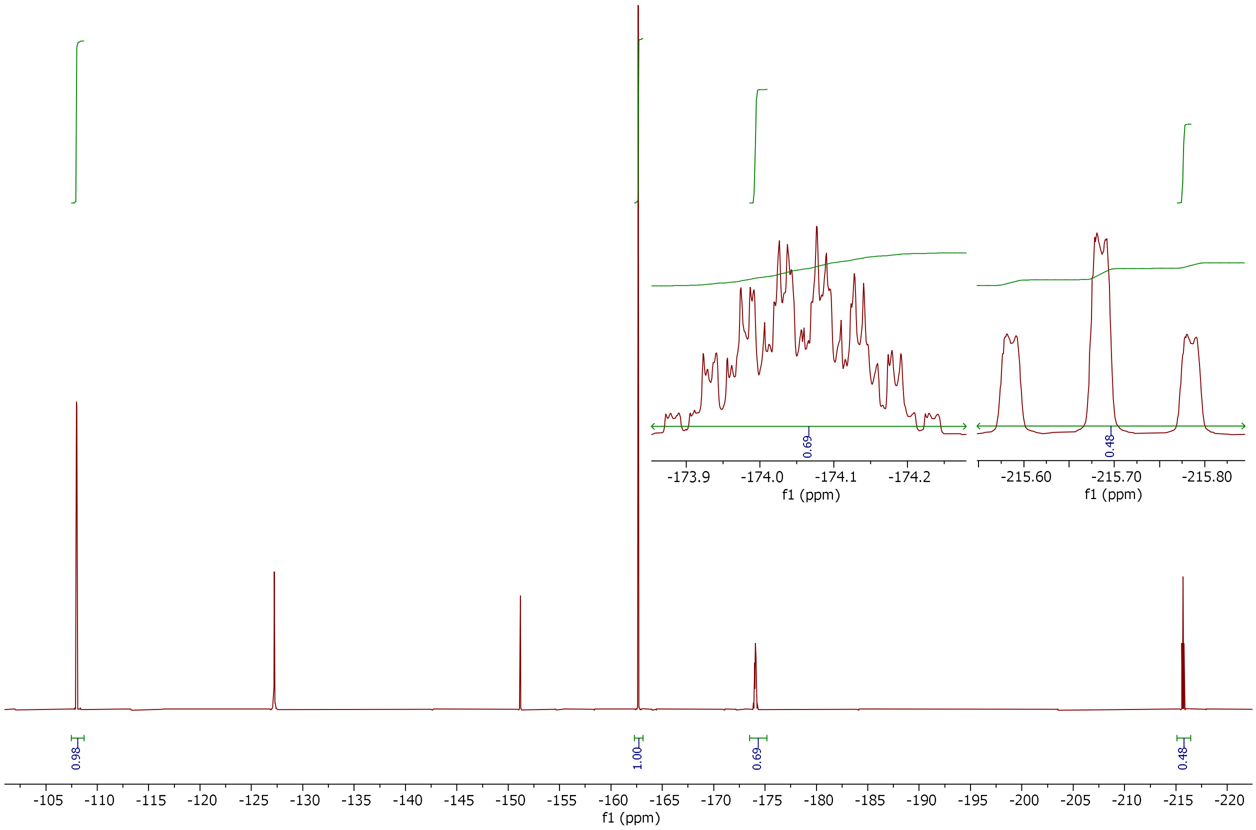

Figure S35. Crude ^{19}F NMR (471 MHz, CH_2Cl_2) spectrum showing competition between **2c** and **2b**.

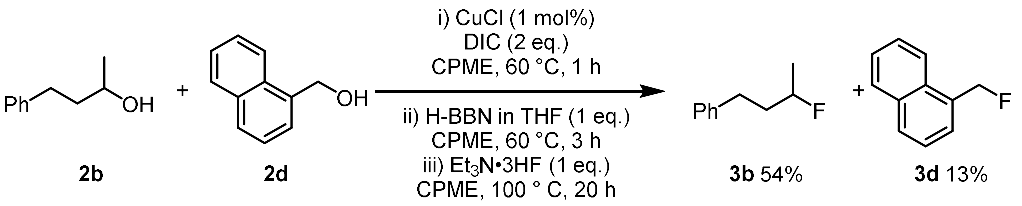

According to general procedure C, 4-phenylbutan-2-ol (75.0 mg, 0.500 mmol) and 1-naphthalenemethanol (79.0 mg, 0.500 mmol) were reacted to give 2-fluoro-4-phenylbutane (54%) and 1-fluoromethylnaphthalene (13%).

^{19}F NMR (565 MHz, CH_2Cl_2) δ −174.2 (m, 0.54F), −206.4 (t, *J* = 47.9, 0.13F).

The data were in accordance with those previously reported.[10]

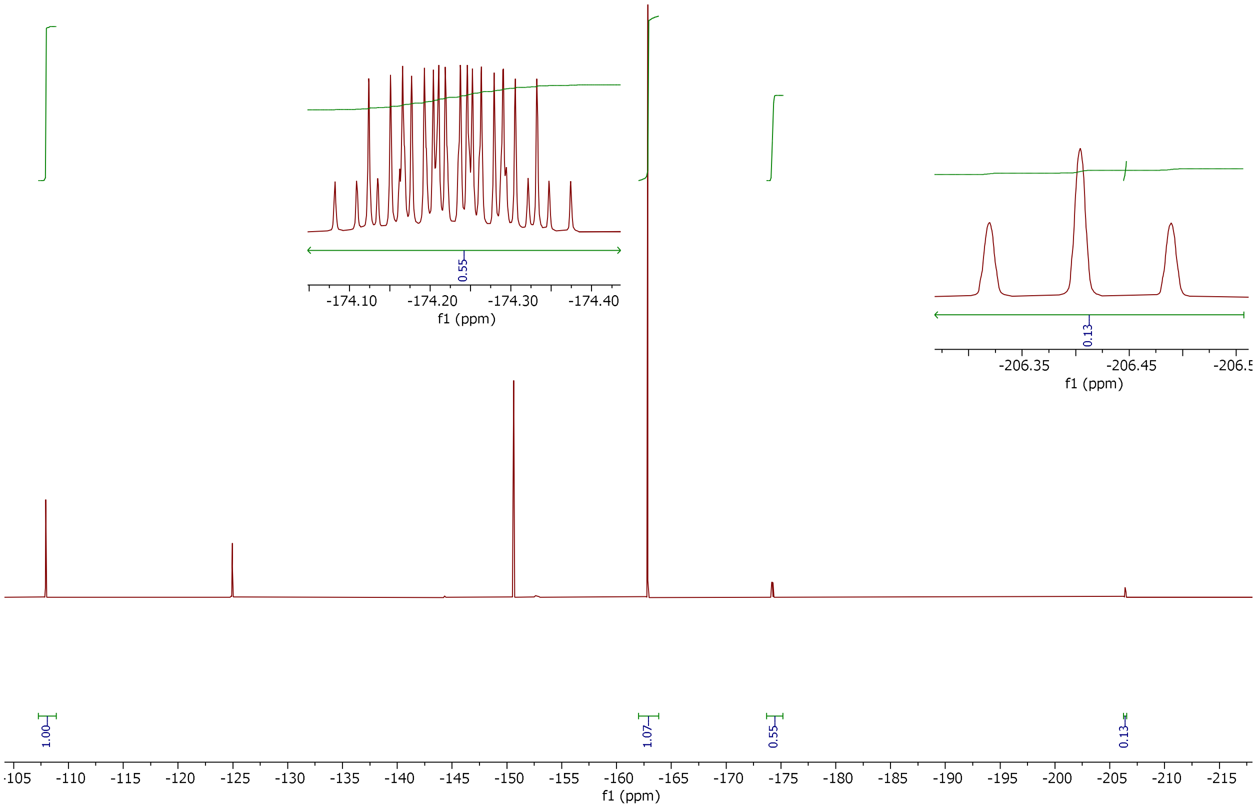

Figure S36. Crude ^{19}F NMR (565 MHz, CH_2Cl_2) spectrum showing competition between **2d** and **2b**.

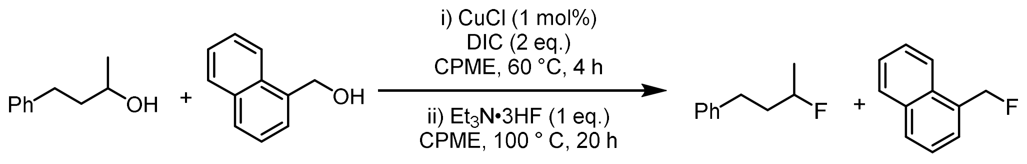

According to general procedure D, 4-phenylbutan-2-ol (75.0 mg, 0.500 mmol) and 1-naphthalenemethanol (79.0 mg, 0.500 mmol) were reacted to give 2-fluoro-4-phenylbutane (76%) and 1-fluoromethylnaphthalene (48%).

^{19}F NMR (471 MHz, CH_2Cl_2) δ −174.1 (m, 0.76F), −206.1 (t, *J* = 47.9, 0.48F).

The data were in accordance with those previously reported.[10]

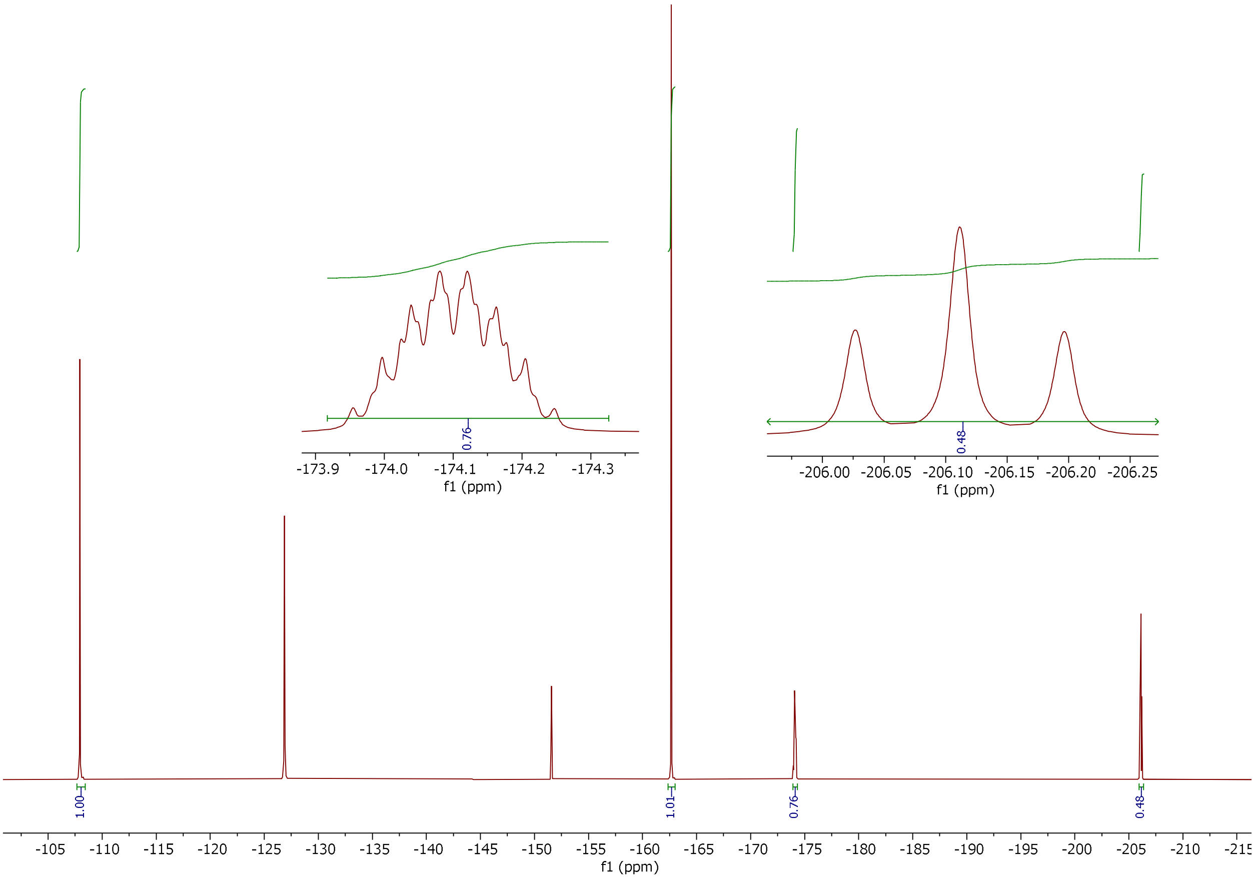

Figure S37. Crude ^{19}F NMR (471 MHz, CH_2Cl_2) spectrum showing competition between **2d** and **2b**.

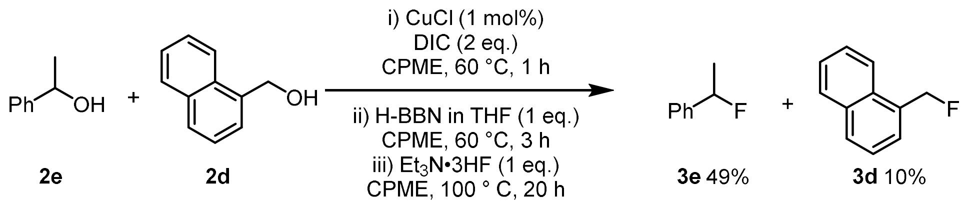

According to general procedure C, 1-phenylethanol (61.0 mg, 0.500 mmol) and 1-naphthalenemethanol (79.0 mg, 0.500 mmol) were reacted to give 1-fluoro-1-phenylethane (49%) and 1-fluoromethylnaphthalene (10%).

^{19}F NMR (565 MHz, CH_2Cl_2) δ −167.1 (m, 0.49F), −206.2 (m, 0.10F).

The data were in accordance with those previously reported.[6,10]

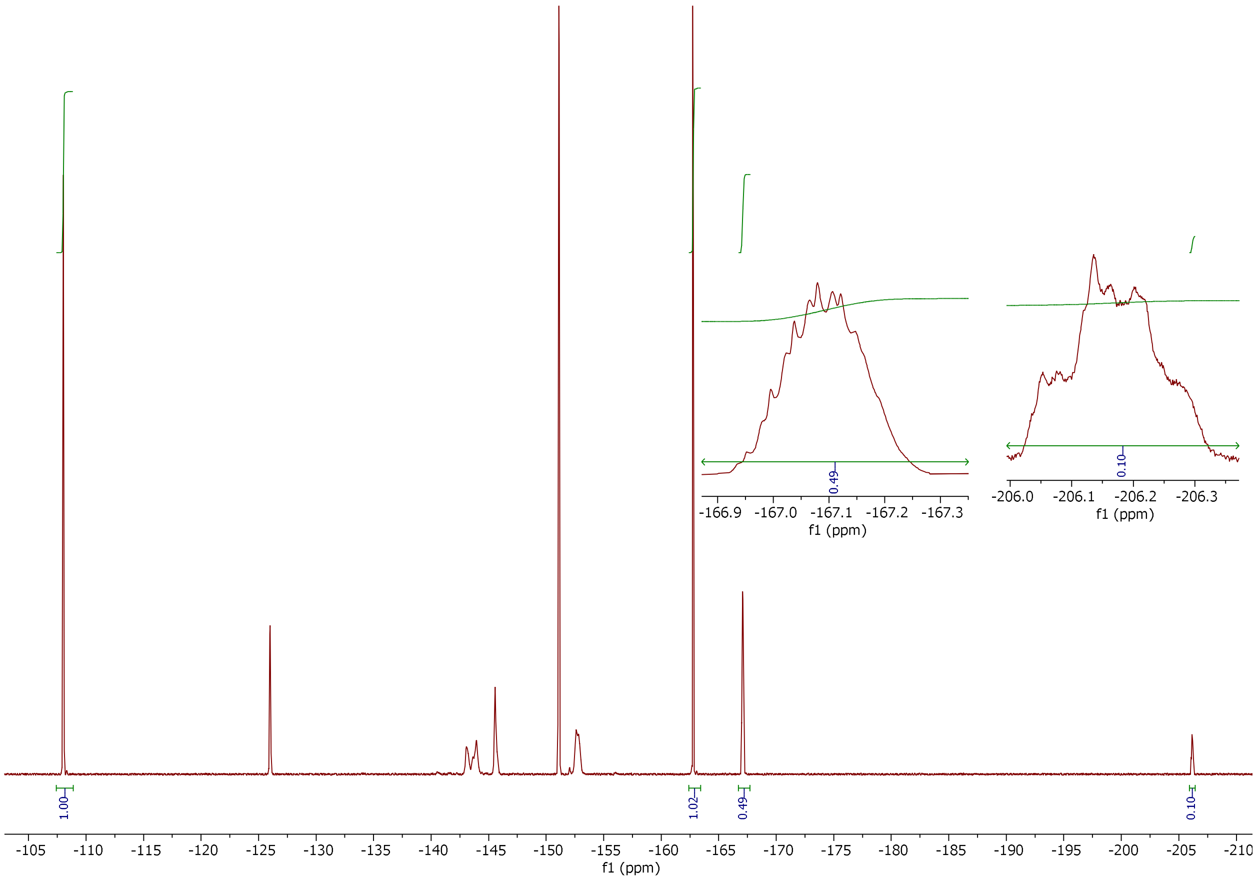

Figure S38. Crude ^{19}F NMR (565 MHz, CH_2Cl_2) spectrum showing competition between **2d** and **2e**.

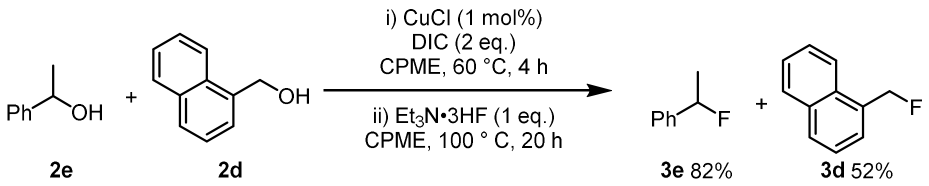

According to general procedure D, 1-phenylethanol (61.0 mg, 0.500 mmol) and 1-naphthalenemethanol (79.0 mg, 0.500 mmol) were reacted to give 1-fluoro-1-phenylethane (82%) and 1-fluoromethylnaphthalene (52%).

^{19}F NMR (471 MHz, CH_2Cl_2) δ −167.0 (m, 0.82F), −206.1 (m, 0.52F).

The data were in accordance with those previously reported.[6,10]

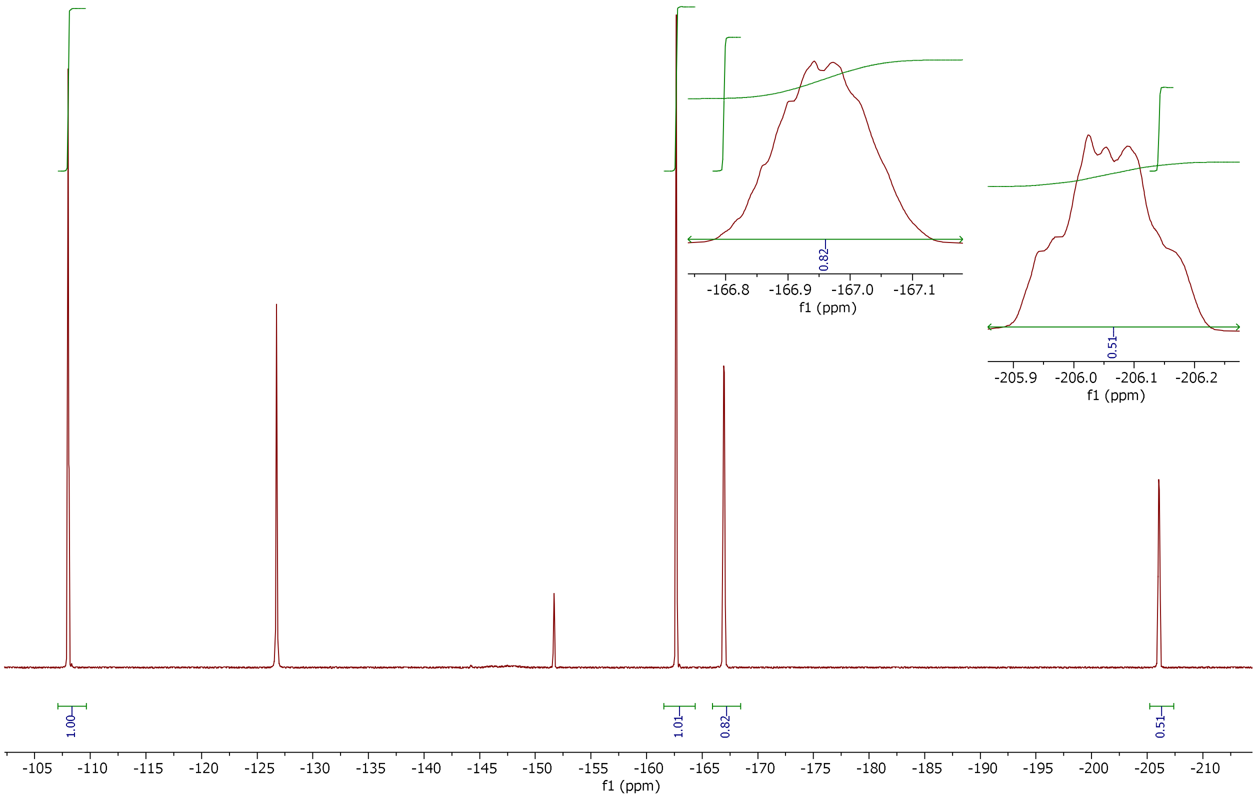

Figure S39. Crude ^{19}F NMR (471 MHz, CH_2Cl_2) spectrum showing competition between **2d** and **2e**.

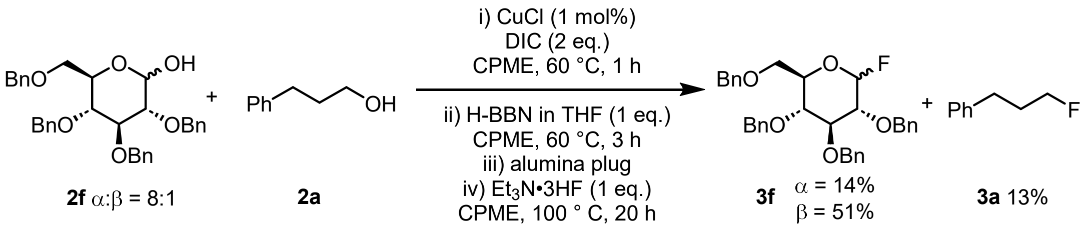

According to general procedure E, 2,3,4,6-tetra-*O*-benzyl-D-galactopyranose (270 mg, 0.500 mmol, α:β = 8:1) and 3-phenylpropan-1-ol (70.0 mg. 0.500 mmol) were reacted to give 2,3,4,6-tetra-*O*-benzyl-D-glucopyranosyl fluoride (α = 14%, β = 51%) and 1-fluoro-3-phenylpropane (13%).

^{19}F NMR (565 MHz, CH_2Cl_2) δ −138.0 (dd, *J* = 53.2, 11.9 Hz, 0.51F, β), −149.4 (dd, *J* = 53.4, 25.8 Hz, 0.14F, α), −220.01 (tt, *J* = 47.4, 25.2 Hz, 0.13F).

The data were in accordance with those previously reported.[7,17]

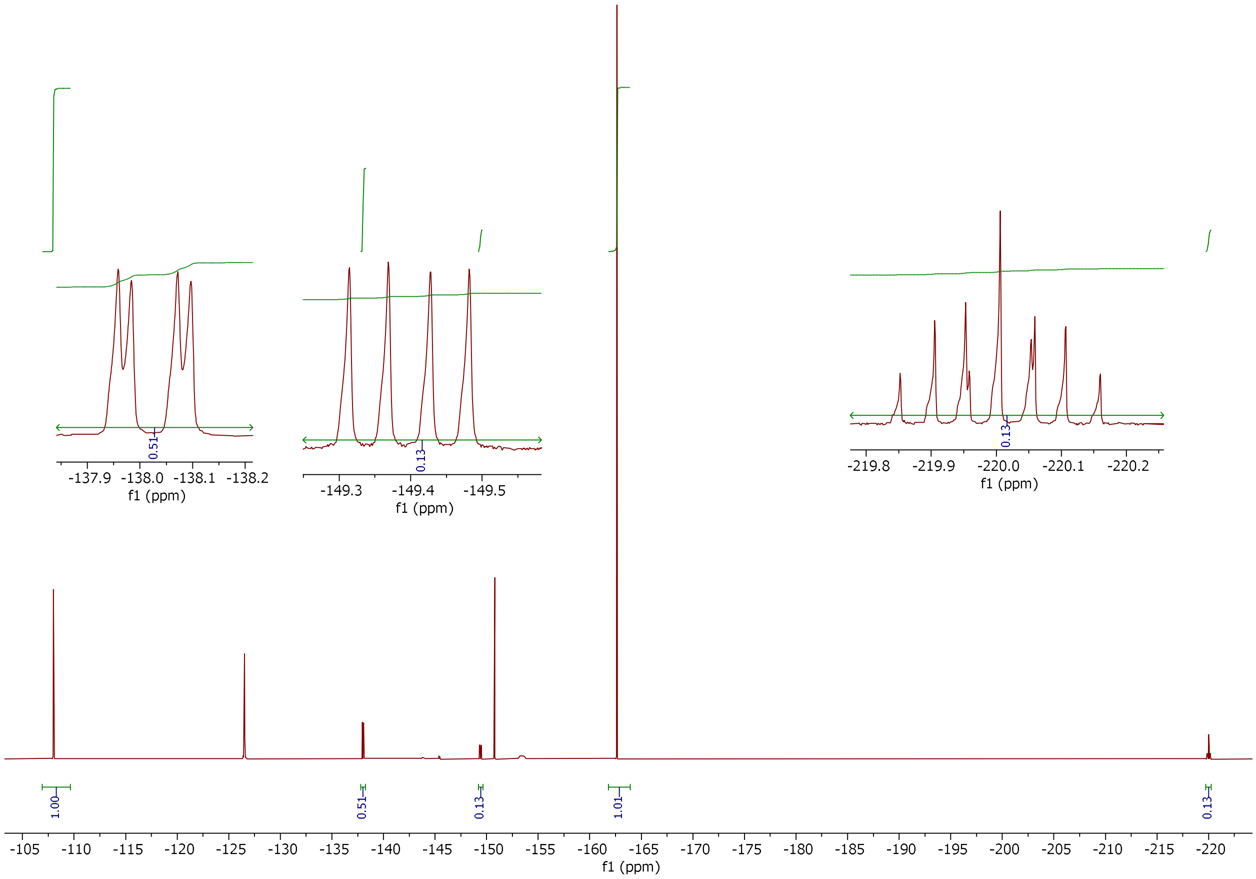

Figure S40. Crude ^{19}F NMR (565 MHz, CH_2Cl_2) spectrum showing competition between **2a** and **2f**.

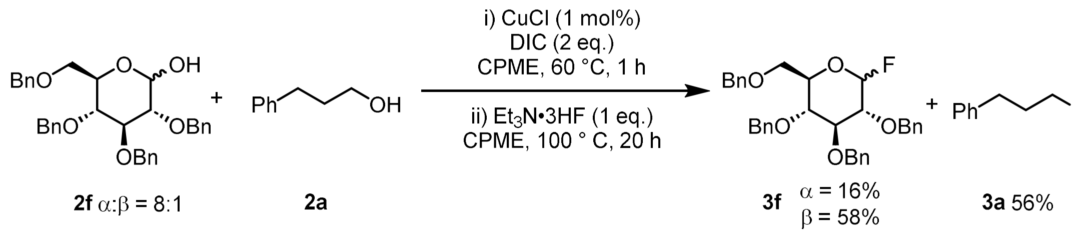

According to procedure B, 2,3,4,6-tetra-*O*-benzyl-D-galactopyranose (270 mg, 0.500 mmol, α:β = 8:1) and 3-phenylpropan-1-ol (70.0 mg. 0.500 mmol) were reacted to give 2,3,4,6-tetra-*O*-benzyl-D-glucopyranosyl fluoride (α = 16%, β = 58%) and 1-fluoro-3-phenylpropane (56%).

^{19}F NMR (565 MHz, CH_2Cl_2) δ −138.0 (dd, *J* = 53.2, 11.9 Hz, 0.58F, β), −149.4 (dd, *J* = 53.4, 25.8 Hz, 0.16F, α), −220.0 (tt, *J* = 47.4, 25.2 Hz, 0.56F).

The data were in accordance with those previously reported.[7,17]

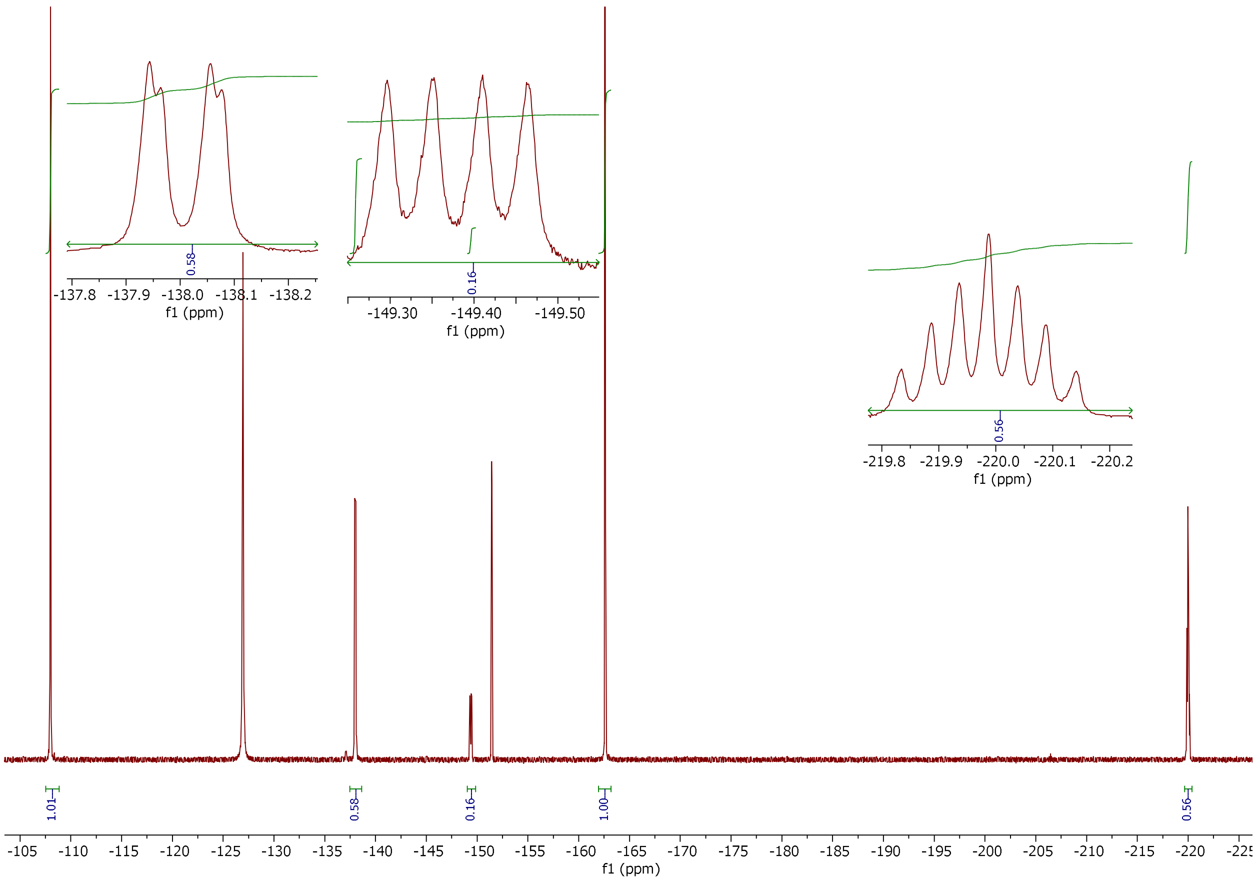

Figure S41. Crude ^{19}F NMR (565 MHz, CH_2Cl_2) spectrum showing competition between **2a** and **2f**.

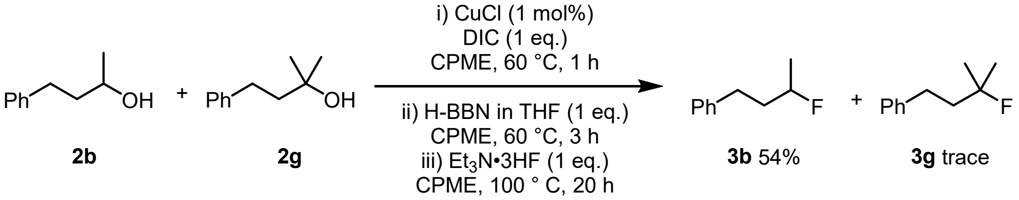

According to a modified general procedure C using only 1 equiv. of DIC, di*iso*propyl carbodiimide (83.0 μL, 0.500 mmol), 4-phenylbutan-2-ol (75.0 mg, 0.500 mmol) and 4-phenyl-2-methylbutan-2-ol (82.0 mg, 0.500 mmol) were reacted to give 2-fluoro-4-phenylbutane (54%) and 2-fluoro-4-phenyl-2-methylbutane (<1%).

^{19}F NMR (565 MHz, CH_2Cl_2) δ −174.3 (m, 0.54F).

The data were in accordance with those previously reported.[7]

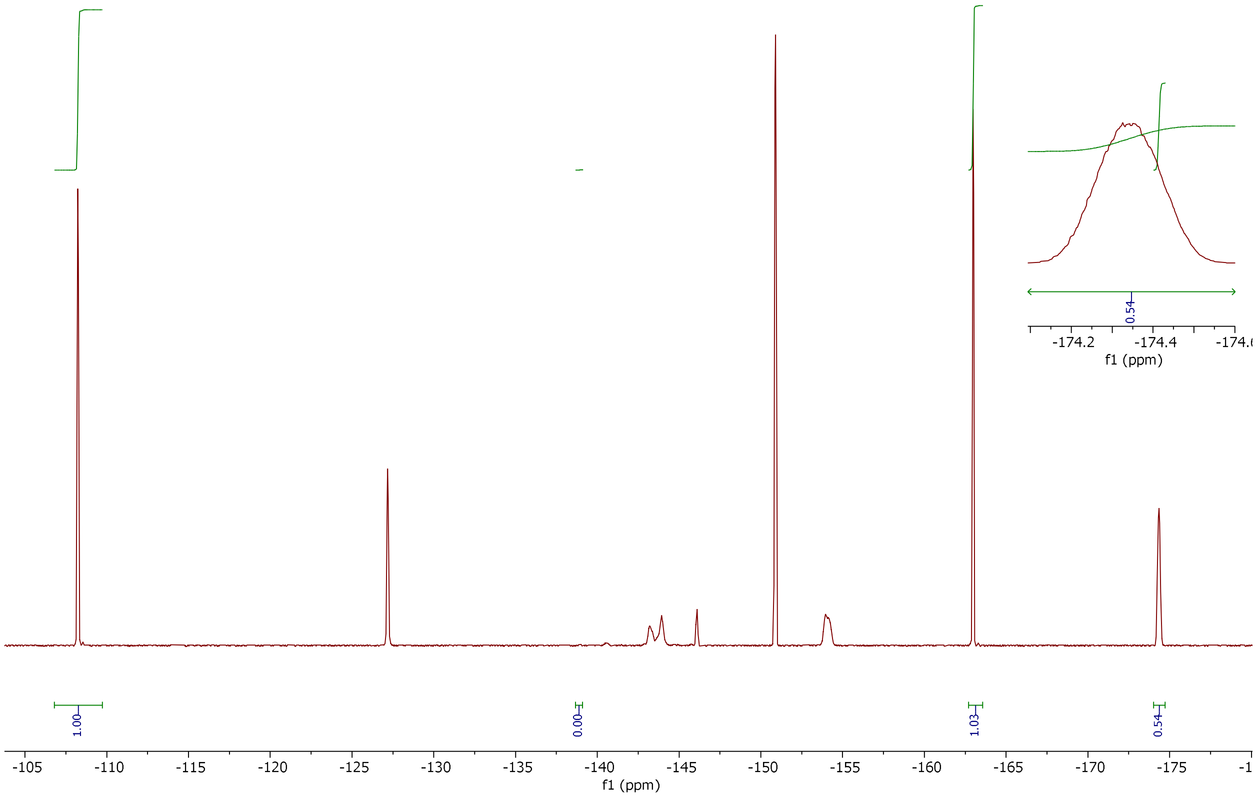

Figure S42. Crude ^{19}F NMR (565 MHz, CH_2Cl_2) spectrum showing competition between **2b** and **2g**.

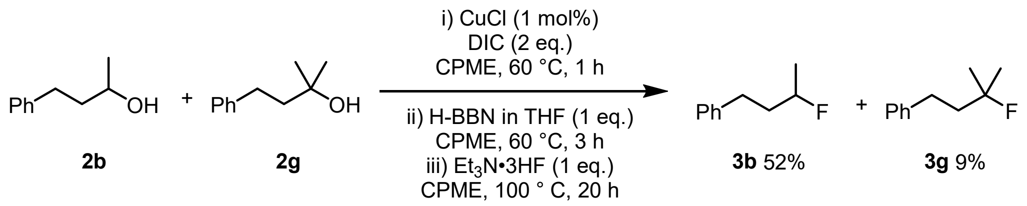

According to general procedure C, 4-phenylbutan-2-ol (75.0 mg, 0.500 mmol) and 4-phenyl-2-methylbutan-2-ol (82.0 mg, 0.500 mmol) were reacted to give 2-fluoro-4-phenylbutane (52%) and 2-fluoro-4-phenyl-2-methylbutane (9%).

^{19}F NMR (565 MHz, CH_2Cl_2) δ −138.9 (m, 0.09F), −174.3 (m, 0.52F).

The data were in accordance with those previously reported.[7]

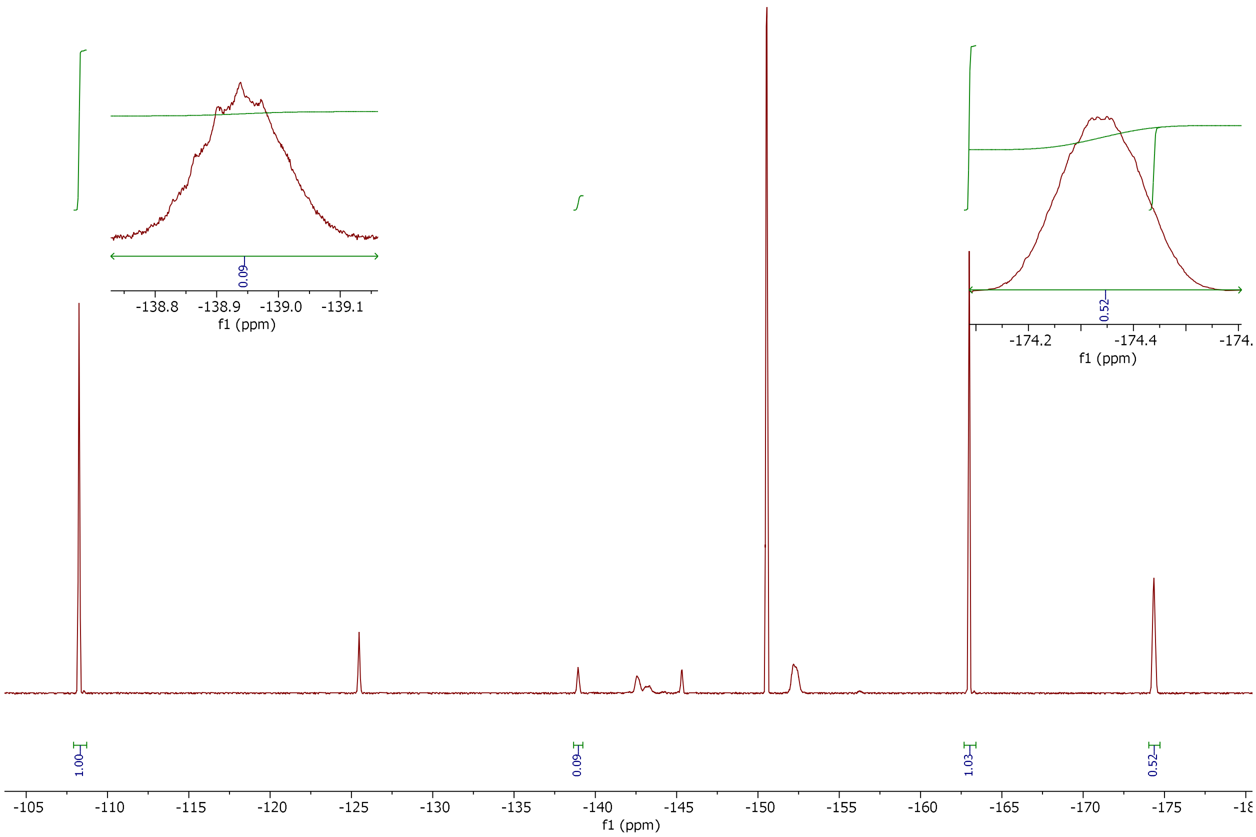

Figure S43. Crude ^{19}F NMR (565 MHz, CH_2Cl_2) spectrum showing competition between **2b** and **2g**.

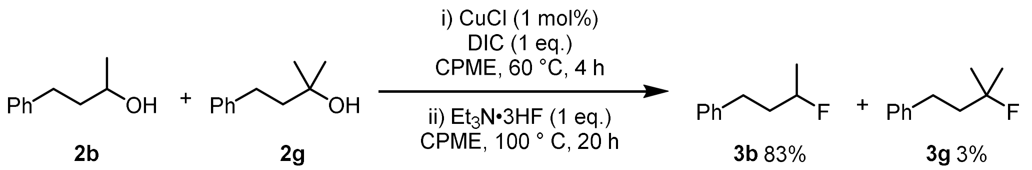

According to a modified general procedure D using 1 equiv. of DIC, di*iso*propyl carbodiimide (83.0 µL, 0.500 mmol), 4-phenylbutan-2-ol (75.0 mg, 0.500 mmol) and 4-phenyl-2-methylbutan-2-ol (82.0 mg, 0.500 mmol) were reacted to give 2-fluoro-4-phenylbutane (83%) and 2-fluoro-4-phenyl-2-methylbutane (3%).

^{19}F NMR (565 MHz, CH_2Cl_2) δ −138.8 (sept., *J* = 20.6 Hz, 0.03F), −174.3 (m, 0.83F).

The data were in accordance with those previously reported.[7]

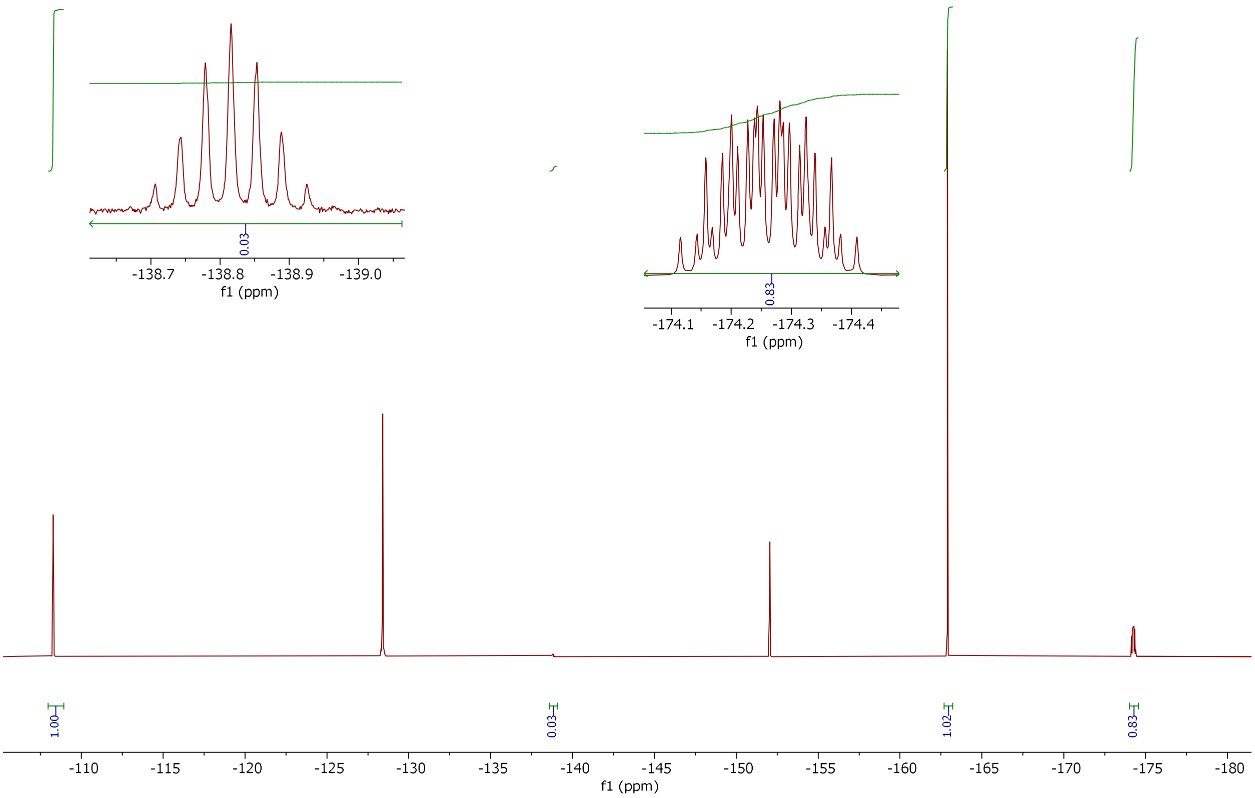

Figure S44. Crude ^{19}F NMR (565 MHz, CH_2Cl_2) spectrum showing competition between **2b** and **2g**.

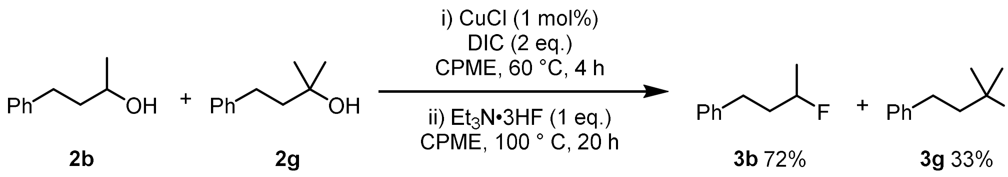

According to general procedure D, 4-phenylbutan-2-ol (75.0 mg, 0.500 mmol) and 4-phenyl-2-methylbutan-2-ol (82.0 mg, 0.500 mmol) were reacted to give 2-fluoro-4-phenylbutane (72%) and 2-fluoro-4-phenyl-2-methylbutane (33%).

^{19}F NMR (565 MHz, CH_2Cl_2) δ −138.8 (sept., J = 20.6 Hz, 0.33F), −174.3 (m, 0.72F).

The data were in accordance with those previously reported.[7]

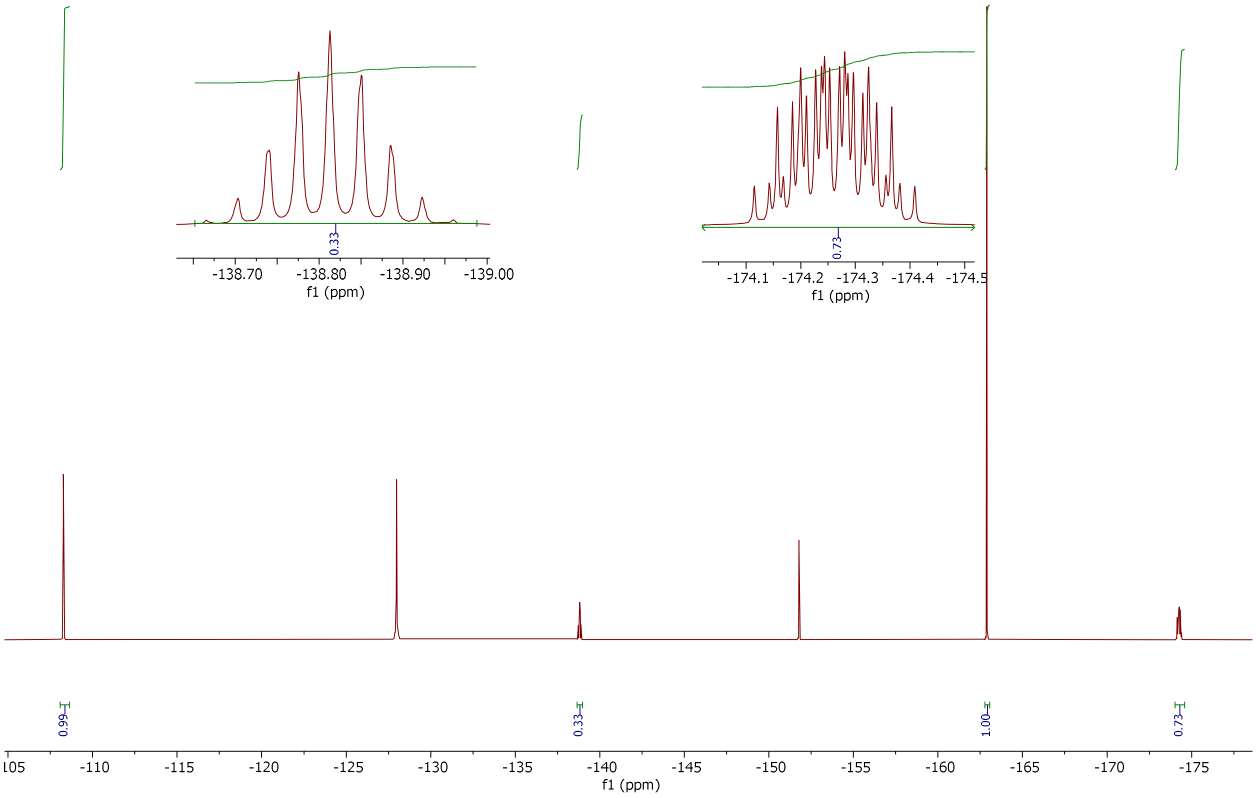

Figure S45. Crude ^{19}F NMR (565 MHz, CH_2Cl_2) spectrum showing competition between **2b** and **2g**.

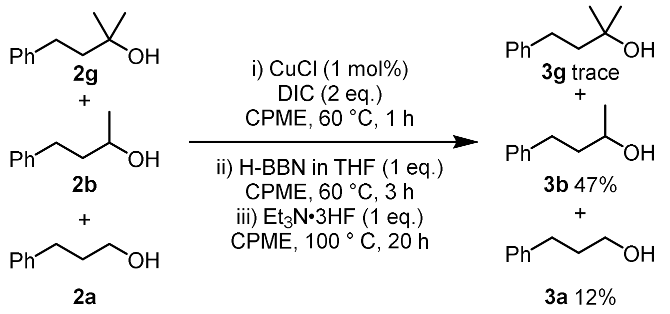

According to a modified general procedure C, 3-phenylpropan-1-ol (70.0 mg. 0.500 mmol), 4-phenylbutan-2-ol (75.0 mg, 0.500 mmol) and 4-phenyl-2-methylbutan-2-ol (82.0 mg, 0.500 mmol) were reacted to give 1-fluoro-3-phenylpropane (12%), 2-fluoro-4-phenylbutane (47%) and 2-fluoro-4-phenyl-2-methylbutane (<1%).

^{19}F NMR (565 MHz, CH_2Cl_2) δ −174.3 (m, 0.47F), −220.3 (m, 0.12F).

The data were in accordance with those previously reported.[7]

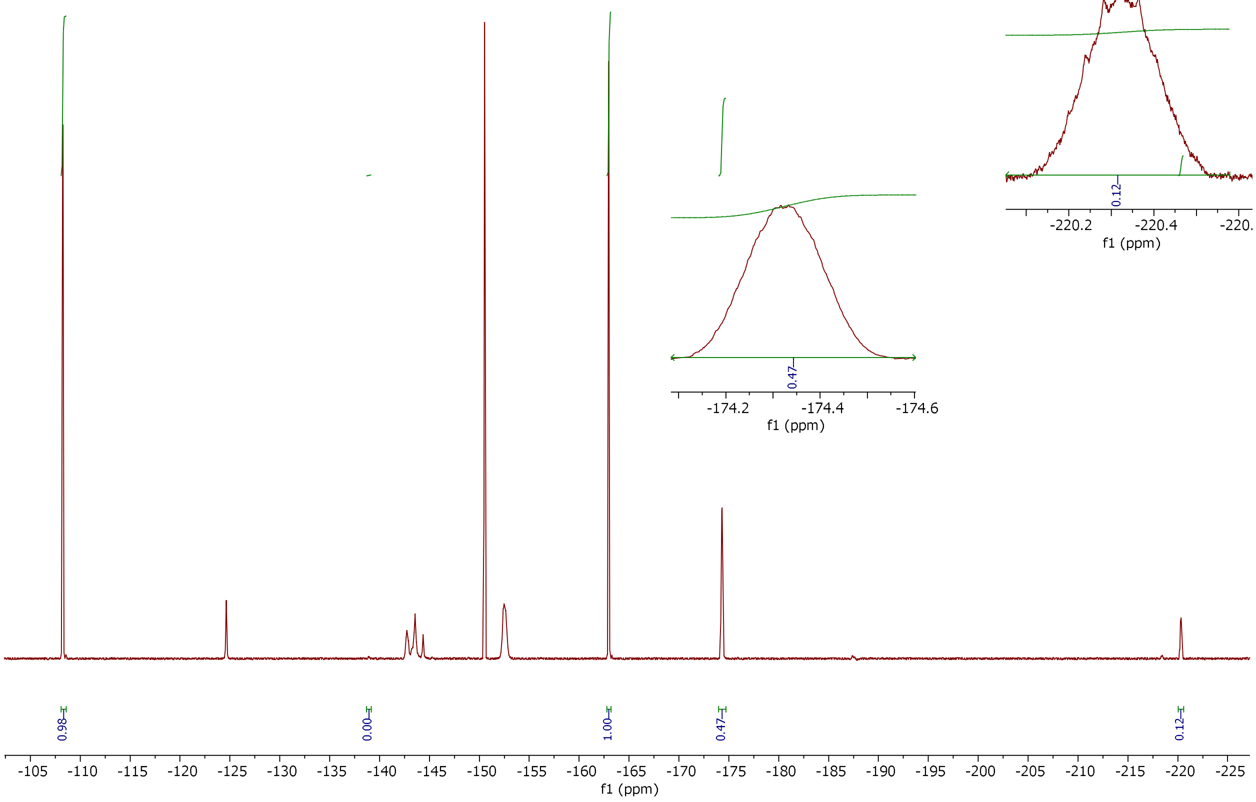

Figure S46. Crude ^{19}F NMR (565 MHz, CH_2Cl_2) spectrum showing competition between **2a**, **2b** and **2g**.

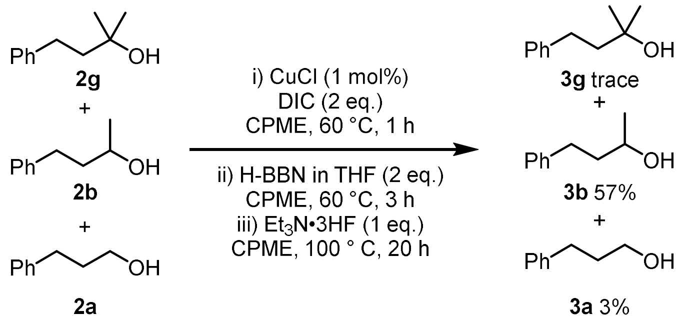

According to a modified general procedure C, 3-phenylpropan-1-ol (70.0 mg. 0.500 mmol), 4-phenylbutan-2-ol (75.0 mg, 0.500 mmol), 4-phenyl-2-methylbutan-2-ol (82.0 mg, 0.500 mmol), and H-BBN (2.00 mL, 0.5 M in THF, 1.00 mmol) were reacted to give 1-fluoro-3-phenylpropane (3%), 2-fluoro-4-phenylbutane (57%) and 2-fluoro-4-phenyl-2-methylbutane (<1%).

^{19}F NMR (565 MHz, CH_2Cl_2) δ −174.3 (m, 0.57F), −220.3 (tt, J = 47.4, 25.2 Hz, 0.03F).

The data were in accordance with those previously reported.[7]

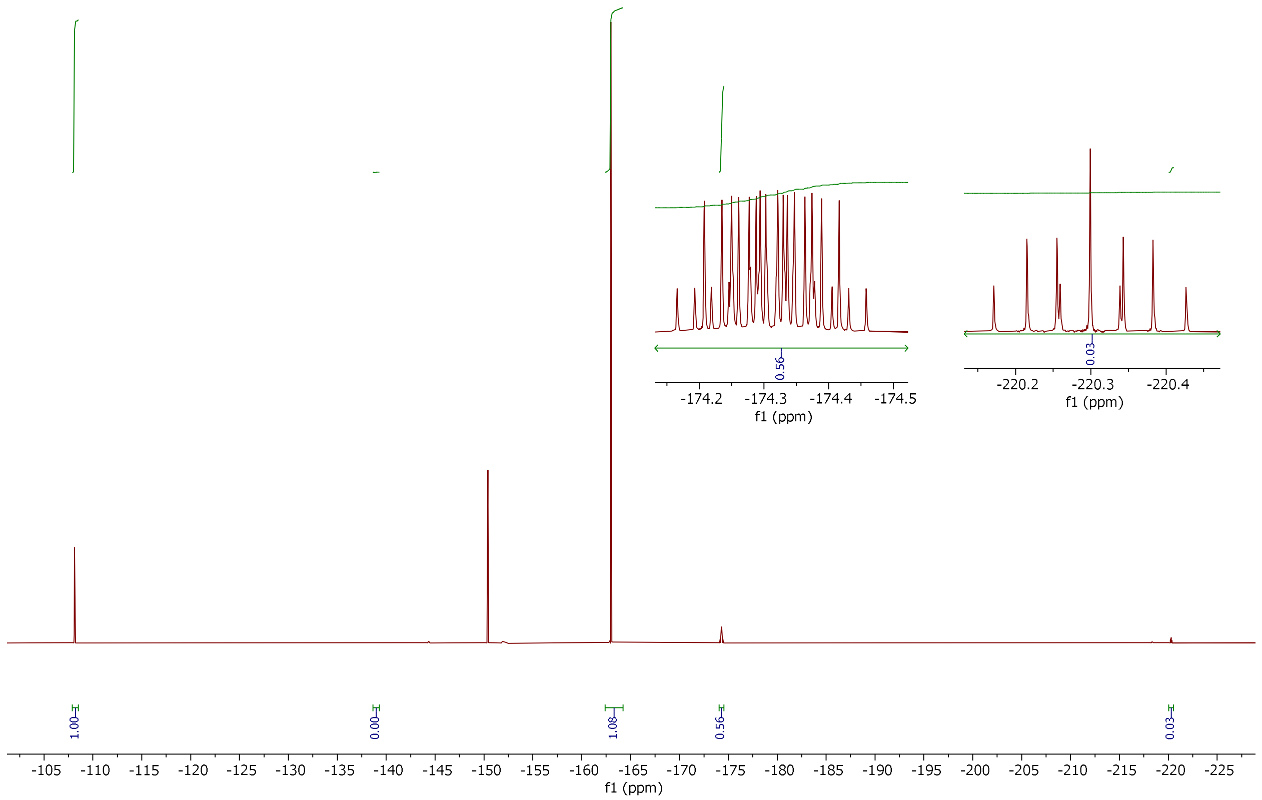

Figure S47. Crude ^{19}F NMR (565 MHz, CH_2Cl_2) spectrum showing competition between **2a**, **2b** and **2g**.

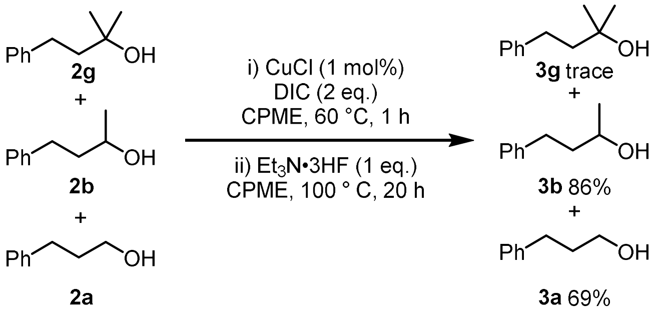

According to a modified general procedure D, 3-phenylpropan-1-ol (70.0 mg. 0.500 mmol), 4-phenylbutan-2-ol (75.0 mg, 0.500 mmol) and 4-phenyl-2-methylbutan-2-ol (82.0 mg, 0.500 mmol) were reacted to give 1-fluoro-3-phenylpropane (69%), 2-fluoro-4-phenylbutane (86%) and 2-fluoro-4-phenyl-2-methylbutane (<1%).

^{19}F NMR (565 MHz, CH_2Cl_2) δ −174.0 (m, 0.86F), −220.3 (m, 0.69F).

The data were in accordance with those previously reported.[7]

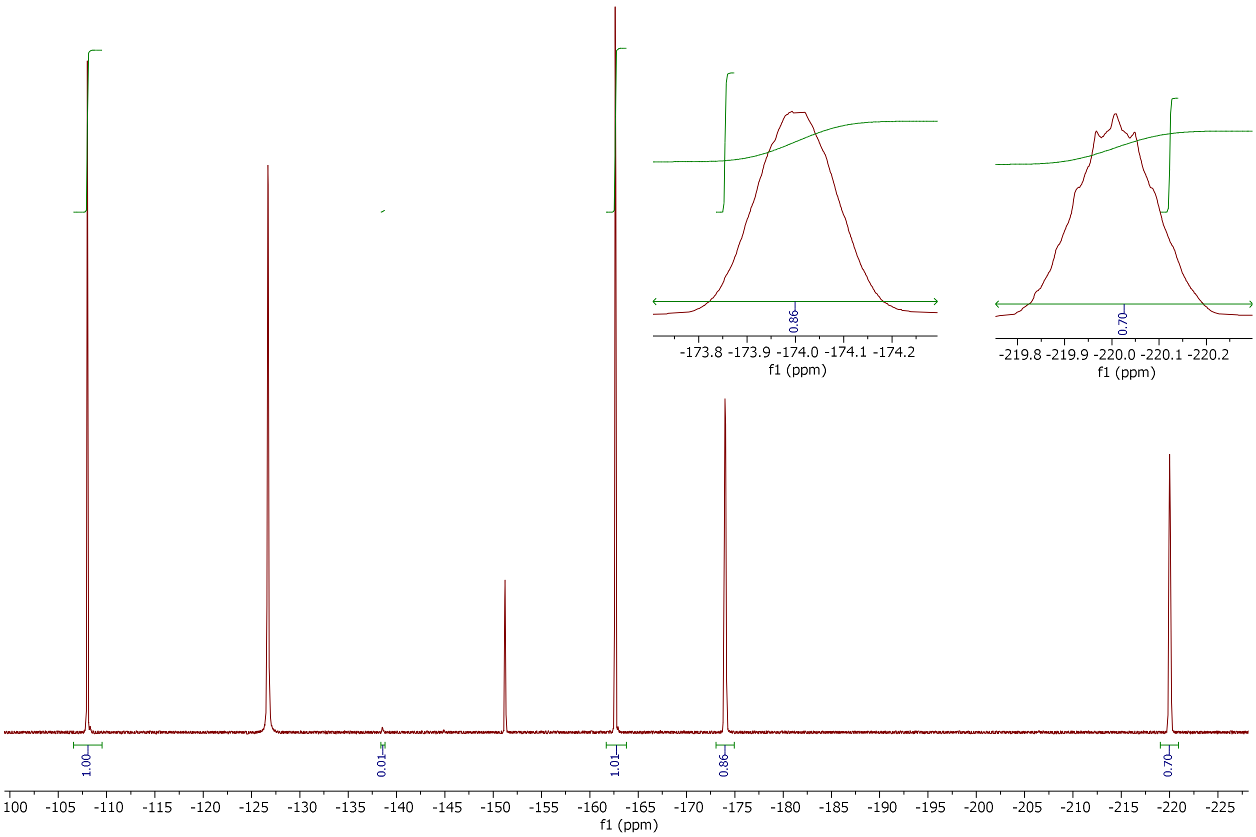

Figure S48. Crude ^{19}F NMR (565 MHz, CH_2Cl_2) spectrum showing competition between **2a**, **2b** and **2g**.

7. Secondary-Selective Deoxyfluorination of Diols

2-Fluoro-4-(4-(3-hydroxypropoxy)phenyl)butane 5a

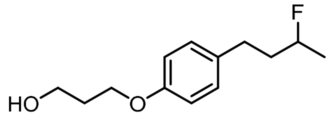

A solution of 4-(4-(3-hydroxypropoxy)phenyl)butan-2-ol (112 mg, 0.500 mmol), di*iso*propyl carbodiimide (165 µL, 1.00 mmol), and copper(I) chloride (1 mg, 5 µmol) in cyclopentyl methyl ether (1.00 mL) was stirred in a sealed vessel under air at 60 °C for one hour. The mixture was allowed to cool to room temperature and H-BBN (1.00 mL, 0.5 M in THF, 0.500 mmol) added in a single portion (caution: H_2 generation), and then the mixture was stirred at 60 °C for three hours. The mixture was allowed to cool to room temperature and $Et_3N{\cdot}3HF$ (81.0 µL, 0.500 mmol) was added in a single portion, and the mixture stirred in a sealed vessel at 100 °C for 20 hours. After cooling to room temperature, the mixture was diluted with dichloromethane (10 mL) and the *in situ* yield measured by ^{19}F NMR spectroscopy using 1,3,5-trifluorobenzene and hexafluorobenzene as internal standards (2°:1° = 63%:4%). This was followed by addition of saturated aqueous calcium chloride solution (5 mL), and further dilution with water (5 mL). The mixture was extracted with dichloromethane (3 × 10 mL), the combined extracts washed with brine (10 mL), dried (stirred over Na_2SO_4), filtered, and concentrated *in vacuo*. The crude product was purified by flash column chromatography (CombiFlash Isco NextGen300+, 12 g SiO_2, 50 mm Ø, 40/60 petroleum ether/EtOAc 100:0 to 0:100) to yield *2-fluoro-4-(4-(3-hydroxypropoxy)phenyl)butane* as a colourless oil (39.0 mg, 34%, 0.170 mmol, 2°:1° = >20:1).

^{1}H NMR (601 MHz, $CDCl_3$) δ 7.16 – 7.11 (m, 2H), 6.88 – 6.84 (m, 2H), 4.75 – 4.59 (m, 1H), 4.14 (t, *J* = 5.9 Hz, 2H), 3.89 (q, *J* = 5.5 Hz, 2H), 2.80 – 2.62 (m, 2H), 2.11 – 1.92 (m, 3H), 1.87 – 1.68 (m, 2H), 1.36 (dd, *J* = 23.9, 6.2 Hz, 3H).

^{13}C NMR (151 MHz, $CDCl_3$) δ 157.0, 133.8, 129.4, 114.5, 90.0 (d, *J* = 164 Hz), 66.0, 60.8, 38.9 (d, *J* = 20.1 Hz), 32.0, 30.4 (d, *J* = 4.8 Hz), 21.0 (d, *J* = 22.7 Hz).

^{19}F NMR (565 MHz, $CDCl_3$) δ −174.3 (ddqd, *J* = 48.0, 30.6, 23.9, 15.3 Hz).

HRMS (ESI$^+$) m/z: Found 227.1450 ($C_{13}H_{20}FO_2$) requires 227.1442.

3β-Fluoro-24-hydroxy-5β-cholane 5b

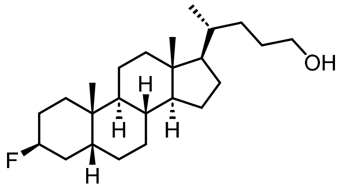

A solution of 5β-cholane-3α,24-diol (181 mg, 0.500 mmol), di*iso*propyl carbodiimide (165 μL, 1.00 mmol), and copper(I) chloride (1 mg, 5 μmol) in cyclopentyl methyl ether (1.00 mL) was stirred in a sealed vessel under air at 60 °C for one hour. The mixture was allowed to cool to room temperature and H-BBN (1.00 mL, 0.5 M in THF, 0.500 mmol) added in a single portion (caution: H_2 generation), and then the mixture was stirred at 60 °C for three hours. The mixture was allowed to cool to room temperature and $Et_3N\cdot 3HF$ (81.0 μL, 0.500 mmol) was added in a single portion, and the mixture stirred in a sealed vessel at 100 °C for 20 hours. After cooling to room temperature, the mixture was diluted with dichloromethane (10 mL) and the *in situ* yield measured by ^{19}F NMR spectroscopy using 1,3,5-trifluorobenzene and hexafluorobenzene as internal standards (2°:1° = 42%:11%). This was followed by addition of saturated aqueous calcium chloride solution (5 mL), and further dilution with water (5 mL). The mixture was extracted with dichloromethane (3 × 10 mL), the combined extracts washed with brine (10 mL), dried (stirred over Na_2SO_4), filtered, and concentrated *in vacuo*. The crude product was purified by flash column chromatography (CombiFlash Isco NextGen300+, 12 g SiO_2, 50 mm Ø, 40/60 petroleum ether/EtOAc 100:0 to 0:100) to yield 3β-fluoro-24-hydroxy-5β-cholane as a colourless amorphous solid (61 mg, 33%, 0.167 mmol, 2°:1° = 10:1). *Note: <10% of the elimination product is observed from decomposition during purification.*

^{1}H NMR (601 MHz, $CDCl_3$) δ 4.87 (dt, *J* = 49.0, 2.8 Hz, 1H), 4.46 – 4.34 (m, 2H), 3.81 – 3.72 (m, 2H), 2.01 – 0.87 (m, 33H, overlapping with H_2O and uncharacterised minor impurities), 0.65 (s, 3H).

^{13}C NMR (151 MHz, $CDCl_3$) δ 90.4 (d, *J* = 167 Hz), 71.0 (d, *J* = 11.1 Hz), 63.8, 63.2, 56.8, 56.4, 42.9, 40.4, 40.1, 37.1, 35.8, 35.6, 35.0, 32.4, 32.2, 32.0, 31.8, 31.7, 30.3, 29.6, 28.4, 28.4, 28.3, 26.7, 26.5, 26.4, 26.3, 26.3, 26.1, 24.4, 23.9, 23.1, 22.2, 22.2, 21.3, 18.8, 12.2. (Note: a ^{13}C-^{19}F doublet versus two individual peaks could not be identified in all cases due to significant overlap).

^{19}F NMR (565 MHz, $CDCl_3$) δ −182.1 (m, 0.9F), −217.3 (ttd, *J* = 47.5, 24.2, 10.3 Hz, 0.1F).

Note: the parent ion was not able to be detected by EI^+, ESI^+, or ESI^- due to sensitivity towards elimination.

(±)-1-*cis*-Fluoro-2-(4-(1-hydroxypropan-2-yl)benzyl)cyclopentane 5c

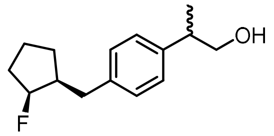

A solution of (±)-*trans*-2-(4-(1-hydroxypropan-2-yl)benzyl)cyclopentan-1-ol (117 mg, 0.500 mmol), di*iso*propyl carbodiimide (165 µL, 1.00 mmol), and copper(I) chloride (1 mg, 5 µmol) in cyclopentyl methyl ether (1.00 mL) was stirred in a sealed vessel under air at 60 °C for one hour. The mixture was allowed to cool to room temperature and H-BBN (1.00 mL, 0.5 M in THF, 0.500 mmol) added in a single portion (caution: H_2 generation), and then the mixture was stirred at 60 °C for three hours. The mixture was allowed to cool to room temperature and $Et_3N{\cdot}3HF$ (81.0 µL, 0.500 mmol) added in a single portion, and the mixture stirred in a sealed vessel at 100 °C for 20 hours. After cooling to room temperature, the mixture was diluted with dichloromethane (10 mL) and the *in situ* yield measured by ^{19}F NMR spectroscopy using 1,3,5-trifluorobenzene and hexafluorobenzene as internal standards (2°:1° = 34%:0%). This was followed by addition of saturated aqueous calcium chloride solution (5 mL), and further dilution with water (5 mL). The mixture was extracted with dichloromethane (3 × 10 mL), the combined extracts washed with brine (10 mL), dried (stirred over Na_2SO_4), filtered, and concentrated *in vacuo*. The crude product was purified by flash column chromatography (CombiFlash Isco NextGen300+, 12 g SiO_2, 50 mm Ø, 40/60 petroleum ether/EtOAc 100:0 to 0:100) to yield (±)-1-*cis*-fluoro-2-(4-(1-hydroxypropan-2-yl)benzyl)cyclopentane as a colourless oil (29 mg, 25%, 0.125 mmol, 2°:1° = >20:1).

^{1}H NMR (601 MHz, $CDCl_3$) δ 7.22 – 7.13 (m, 4H), 4.85 (dt, *J* = 54.3, 3.8 Hz, 1H), 3.69 (t, *J* = 6.0 Hz, 2H), 2.99 – 2.81 (m, 3H), 2.73 – 2.62 (m, 1H), 2.05 – 1.33 (m, 7H, overlapping with H_2O), 1.27 (d, *J* = 7.0 Hz, 3H).

^{13}C NMR (151 MHz, $CDCl_3$) δ 141.1, 140.0, 129.3, 127.6, 97.1 (d, *J* = 175 Hz), 68.9, 48.2 (d, *J* = 20.2 Hz), 42.2, 34.8 (d, *J* = 6.0 Hz), 32.7 (d, *J* = 21.7 Hz), 32.2, 29.2, 26.4, 22.2, 17.8.

^{19}F NMR (565 MHz, $CDCl_3$) δ -189.2 (dddd, *J* = 54.1, 40.5, 33.5, 26.8 Hz).

HRMS (ESI^+) m/z: Found 259.1459 ($C_{15}H_{21}FONa$) requires 259.1469.

The absolute configuration of the major diastereomer was unable to be determined. Only the *cis*-1,2-fluorocyclopentane configuration was assigned by comparison of ^{19}F NMR chemical shifts and coupling constants to *cis*-1-fluoro-2-methylcyclopentane.[19]

(±)-1-Fluoro-3-(2-hydroxy-ethyl)-2-((*Z*)-pent-2-enyl)-cyclopentane 5d

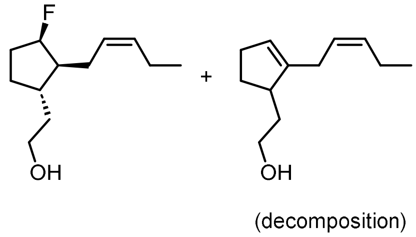

A solution of (±)-3-(2-*O*-(*N*,*N'*-di*iso*propylcarbamimidyl)-ethyl)-2-((*Z*)-pent-2-enyl)-cyclopentan-1-*O*-*N*,*N'*-diisopropylcarbamimidate (225 mg, 0.500 mmol) and H-BBN (61.0 mg, 0.500 mmol) in toluene (3.00 mL) was stirred at 60 °C for three hours. The mixture was allowed to cool to room temperature and $Et_3N{\cdot}3HF$ (81.0 µL, 0.500 mmol) added in a single portion, and then the mixture was stirred in a sealed vessel at 100 °C for 20 hours. After cooling to room temperature, the mixture was diluted with dichloromethane (10 mL) and the *in situ* yield measured by ^{19}F NMR spectroscopy using 1,3,5-trifluorobenzene and hexafluorobenzene as internal standards (2°:1° = 37%:0%). The product was not able to be isolated free of impurities due to rapid decomposition to the presumed elimination product (±)-3-(2-hydroxy-ethyl)-2-((*Z*)-pent-2-enyl)-cyclopent-1-ene. Characterisation data of the product and decomposition products are included after flash chromatography on silica. Note the ^{19}F NMR data is characteristic of the 2° fluoride as it is closely comparable to related *cis*-substituted fluorocyclopentanes (e.g., see above).

Selected characteristic data:

^{1}H NMR (601 MHz, $CDCl_3$) δ 4.95 (dtd, *J* = 54.2, 3.9, 1.9 Hz, FC−H).

^{13}C NMR (151 MHz, $CDCl_3$) δ 97.2 (d, *J* = 175 Hz, C−F).

^{19}F NMR (565 MHz, $CDCl_3$) δ -187.4 (m).

HRMS (ESI^+) m/z: Found 223.1467 ($C_{12}H_{21}FONa$) requires 223.1469.

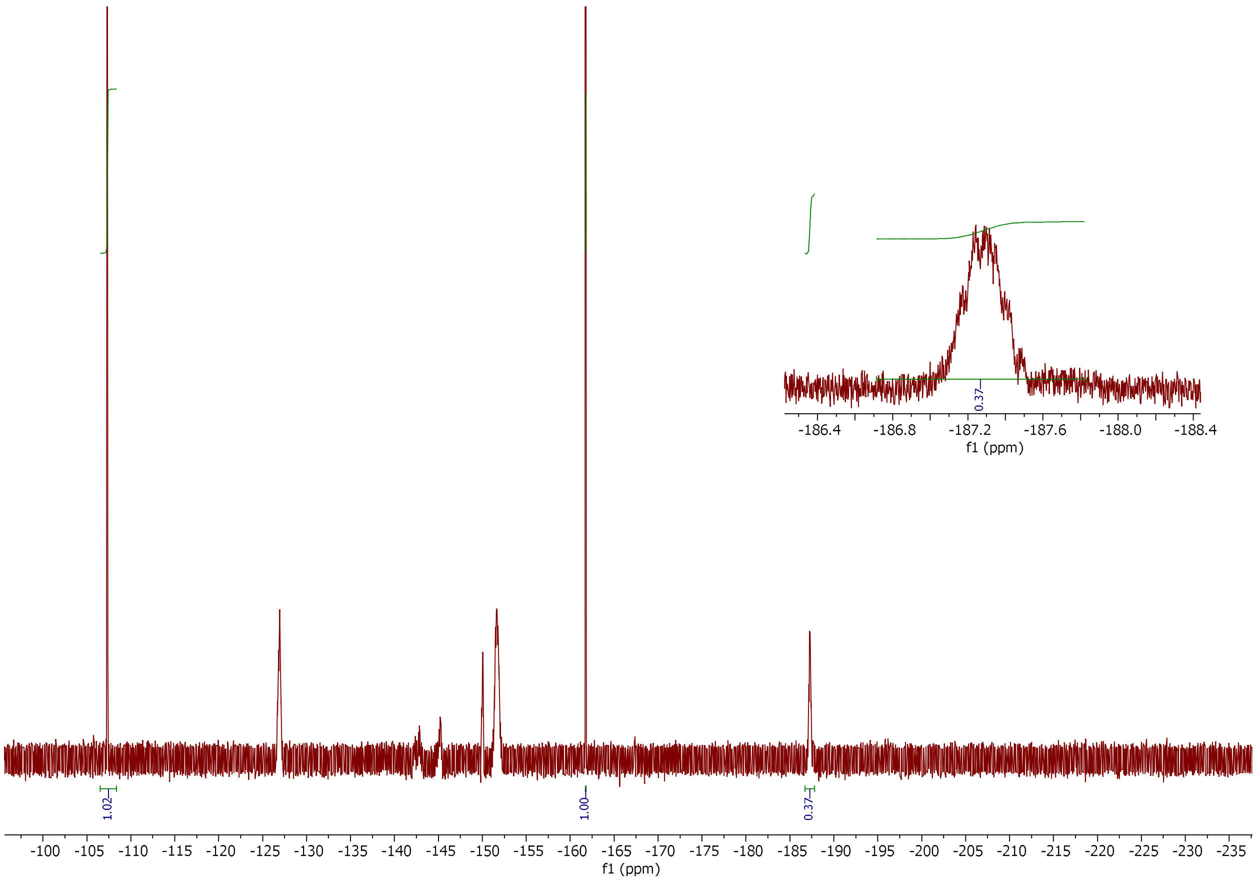

Figure S49. Crude ^{19}F NMR (565 MHz, CH_2Cl_2) spectrum of reaction mixture with internal standards. Other resonances seen are consistent with fluoroboranes seen in other crude reaction mixtures.

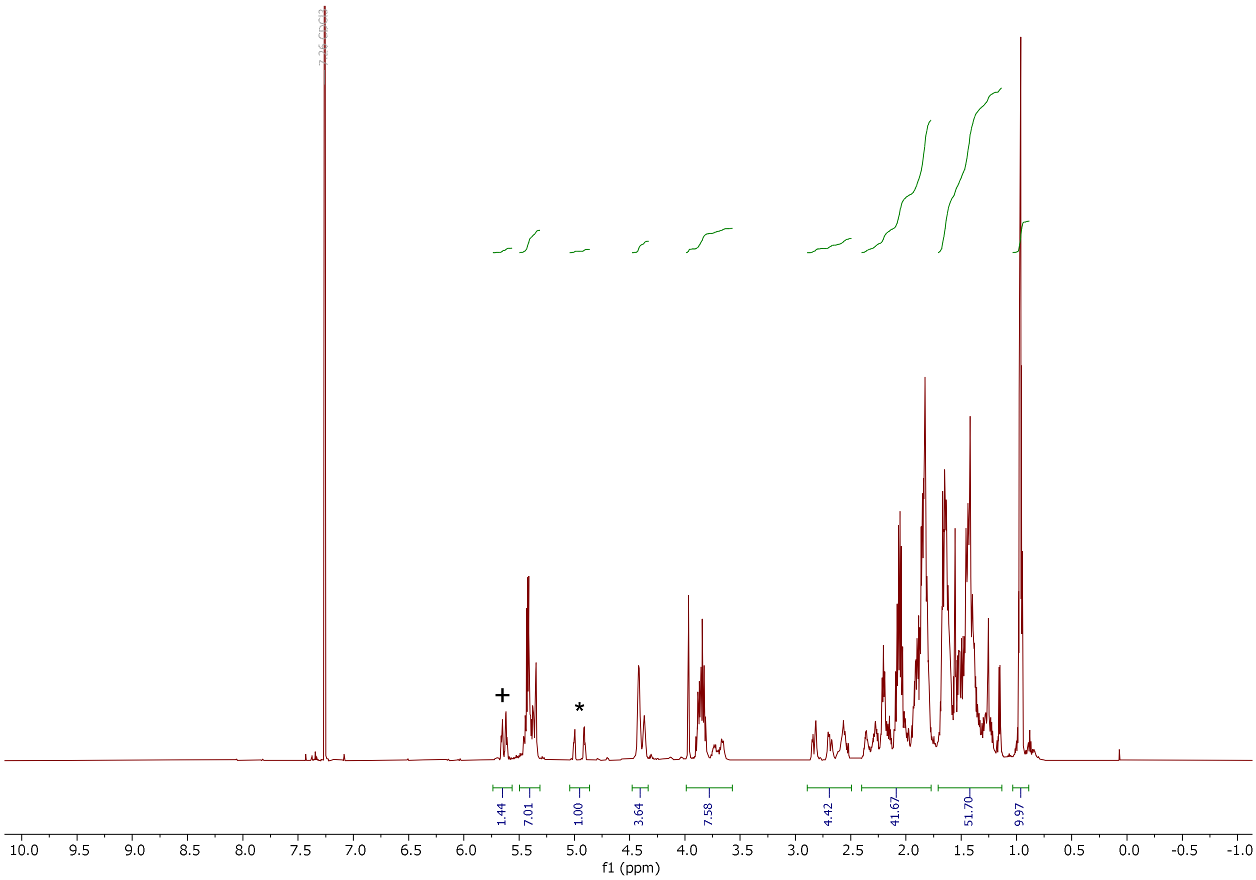

Figure S50. ^{1}H NMR (601 MHz, $CDCl_3$) spectrum of product after column chromatography. * Signal attributed to expected fluorination product. $^+$ Signal attributed to elimination product.

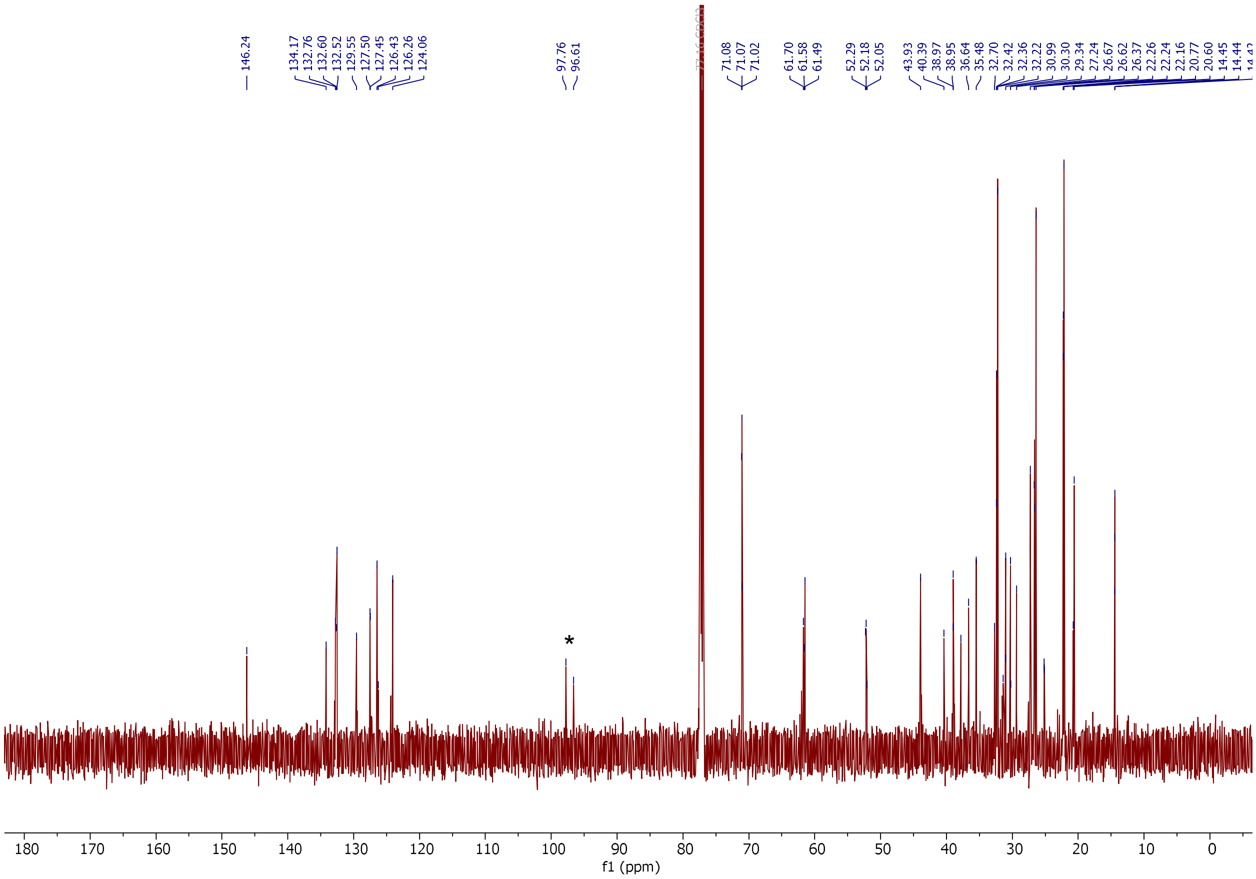

Figure S51. ^{13}C NMR (151 MHz, $CDCl_3$) spectrum of product after column chromatography. * Signal attributed to expected fluorination product.

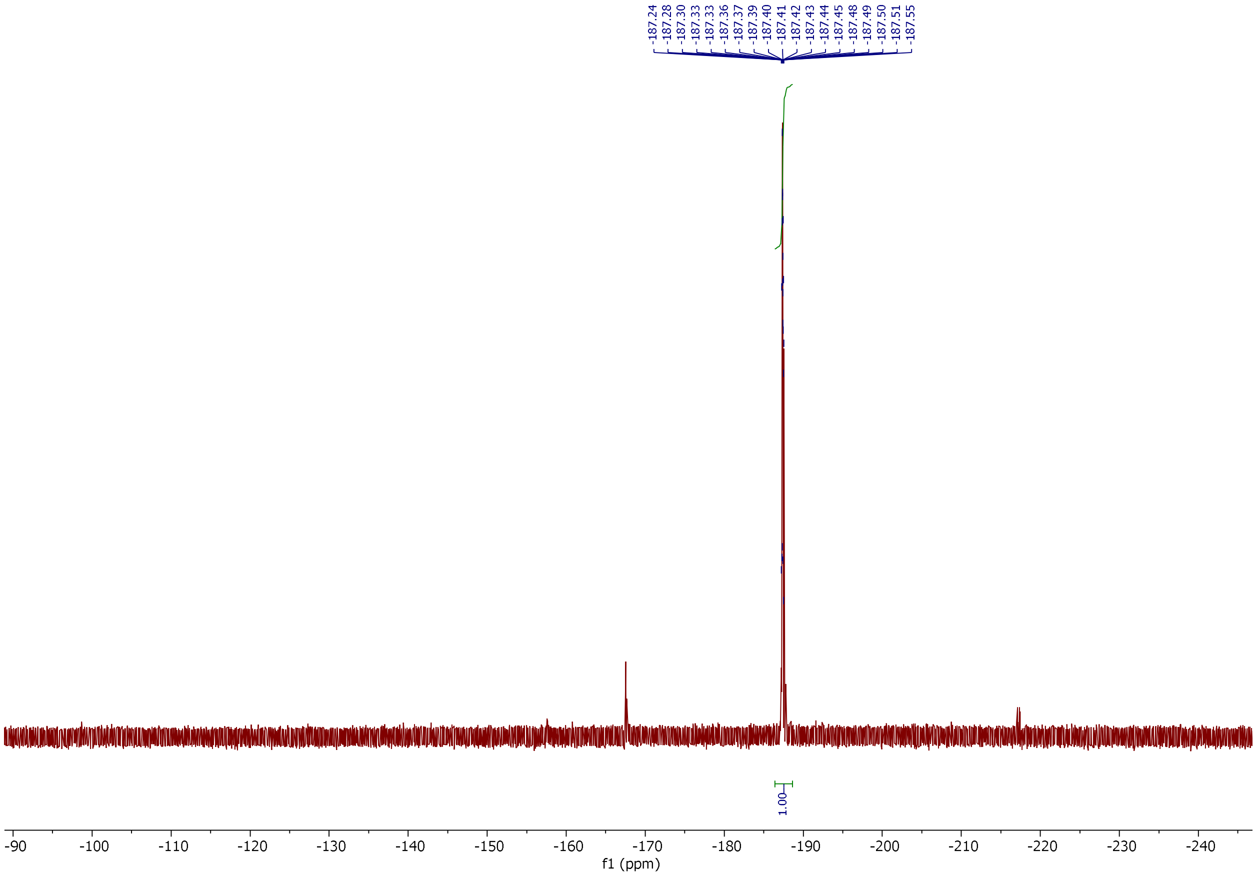

Figure S52. ^{19}F NMR (565 MHz, $CDCl_3$) spectrum of reaction after column chromatography.

8. Enantiospecificity Investigations

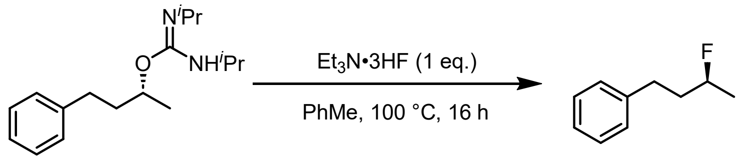

To a stirred solution of (*R*)-*O*-(4-phenyl-2-butyl)-*N,N'*-diisopropylisourea **(*R*)-6b** (0.100 mmol, 28.0 mg) in toluene (0.600 mL) was added $Et_3N\cdot 3HF$ (0.100 mmol, 16.0 μL), and the mixture was heated at 100 °C for 16 hours. Upon cooling to room temperature, a saturated solution of $CaCl_{2(aq)}$ (1 mL) was added, the mixture then was extracted with diethyl ether (3 × 5 mL), dried (stirred over Na_2SO_4), and volatiles removed *in vacuo*. The crude reaction mixture was analysed by chiral HPLC to determine enantiomeric excess (>98% *e.e.*).

HPLC >99:1 *e.r.* [Chiralpak OD-H (0.46 × 25 cm), particle size = 5 μm, hexane (100%), v = 1.0 mL min^{-1}, λ = 210 nm, t (minor) = not observed, t (major) = 15.268 minutes].

The racemate **3b** was prepared by deoxyfluorination of the racemic alcohol using DAST, according to literature procedure.[20] The data were in accordance with those previously reported.[20]

Absolute configuration of the fluoride was determined by comparison with a genuine sample of (*S*)-4-phenyl-2-fluorobutane prepared using PyFluor, which is known to react with high S_N2 enantiospecificity.[21]

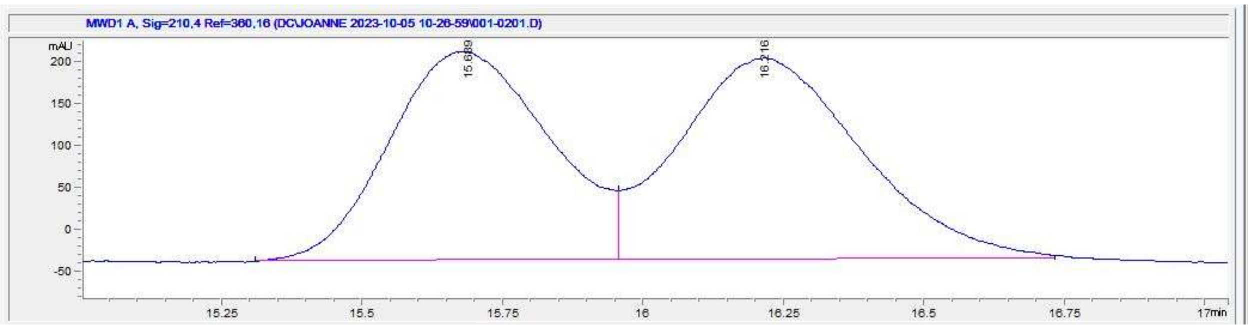

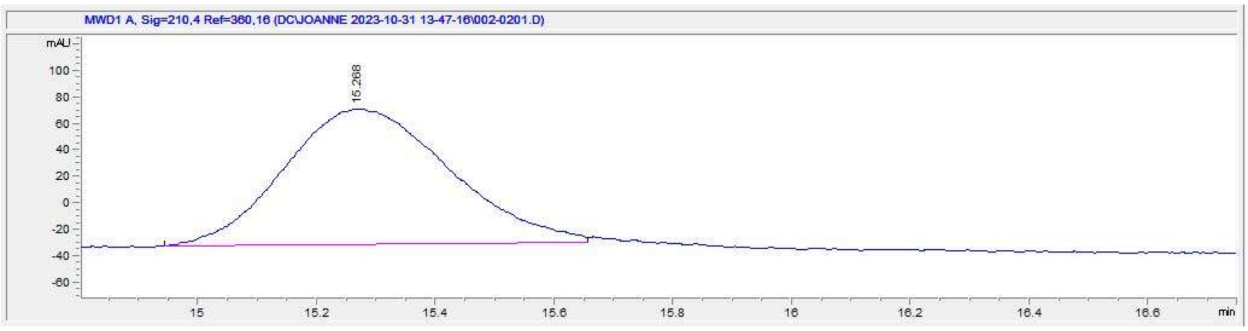

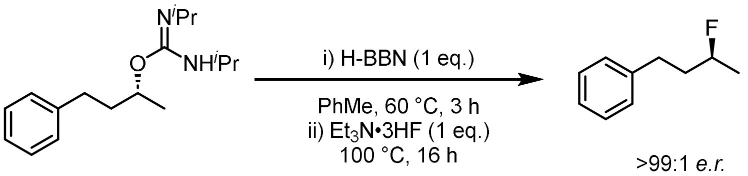

To a stirred solution of (*R*)-*O*-(4-phenyl-2-butyl)-*N,N'*-diisopropylisourea ***(R)*-6b** (0.100 mmol, 28.0 mg) in toluene (0.600 mL) was added H-BBN dimer (50.0 μmol, 12.2 mg), and the mixture was heated at 60 °C for 3 hours. To the reaction mixture was added $Et_3N\cdot 3HF$ (0.100 mmol, 16.0 μL), and the mixture was heated at 100 °C for 16 hours. Upon cooling to room temperature, a saturated solution of $CaCl_{2(aq)}$ (1 mL) was added, the mixture then was extracted with diethyl ether (3 × 5 mL), dried (stirred over Na_2SO_4), and volatiles removed *in vacuo*. The crude reaction mixture was analysed by chiral HPLC to determine enantiomeric excess (>98% *e.e.*).

HPLC >99:1 *e.r.* [Chiralpak OD-H (0.46 × 25 cm), particle size = 5 μm, hexane (100%), v = 1.0 mL min^{-1}, λ = 210 nm, t (minor) = not observed, t (major) = 15.526 minutes].

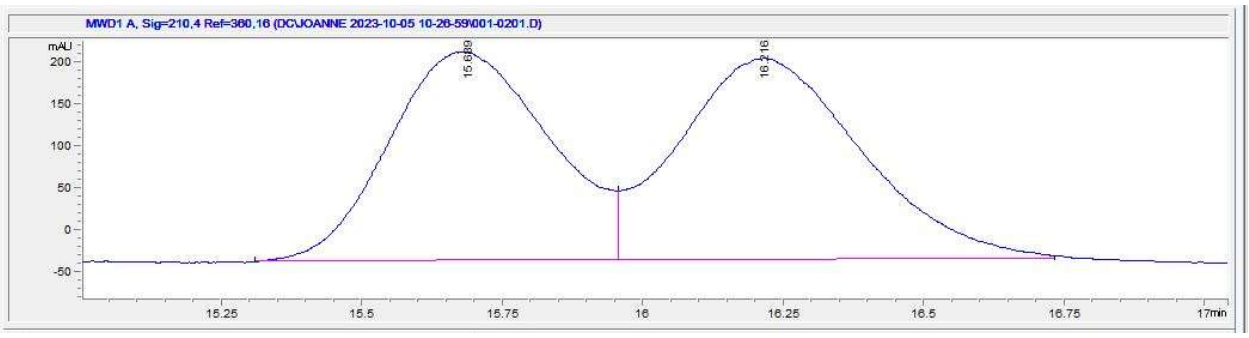

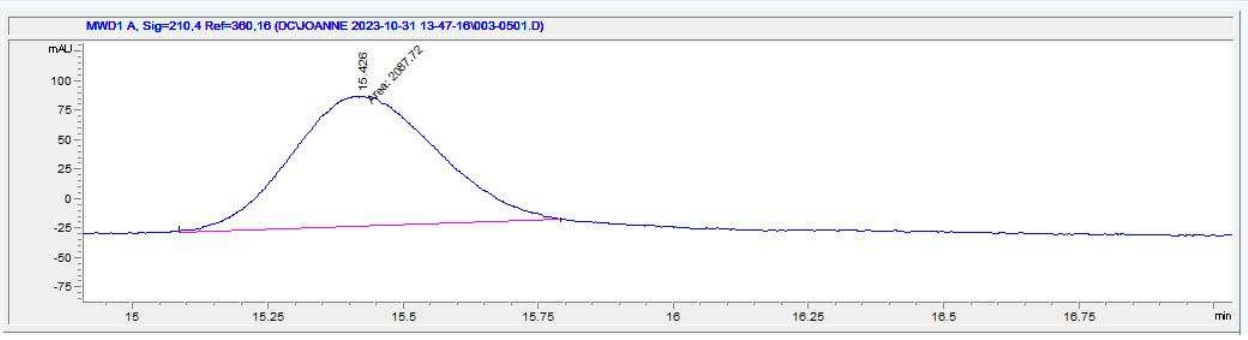

9. NMR Investigations

Dehydrocoupling and DIC elimination of primary isourea

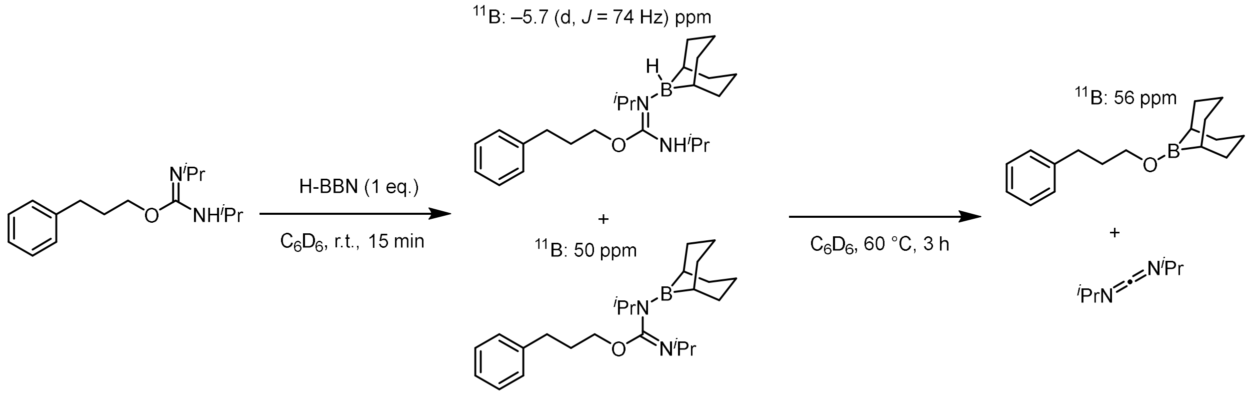

In a J-Young NMR tube, to a solution of *O*-(3-phenyl-1-propyl)-*N,N'*-diisopropylisourea (0.100 mmol, 28.0 mg) in C_6D_6 (0.600 mL) was added H-BBN dimer (50.0 µmol, 12.2 mg), and the mixture was analysed by NMR spectroscopy (after *ca.* 15 minutes). The mixture then was heated at 60 °C for 3 hours and analysed by NMR spectroscopy.

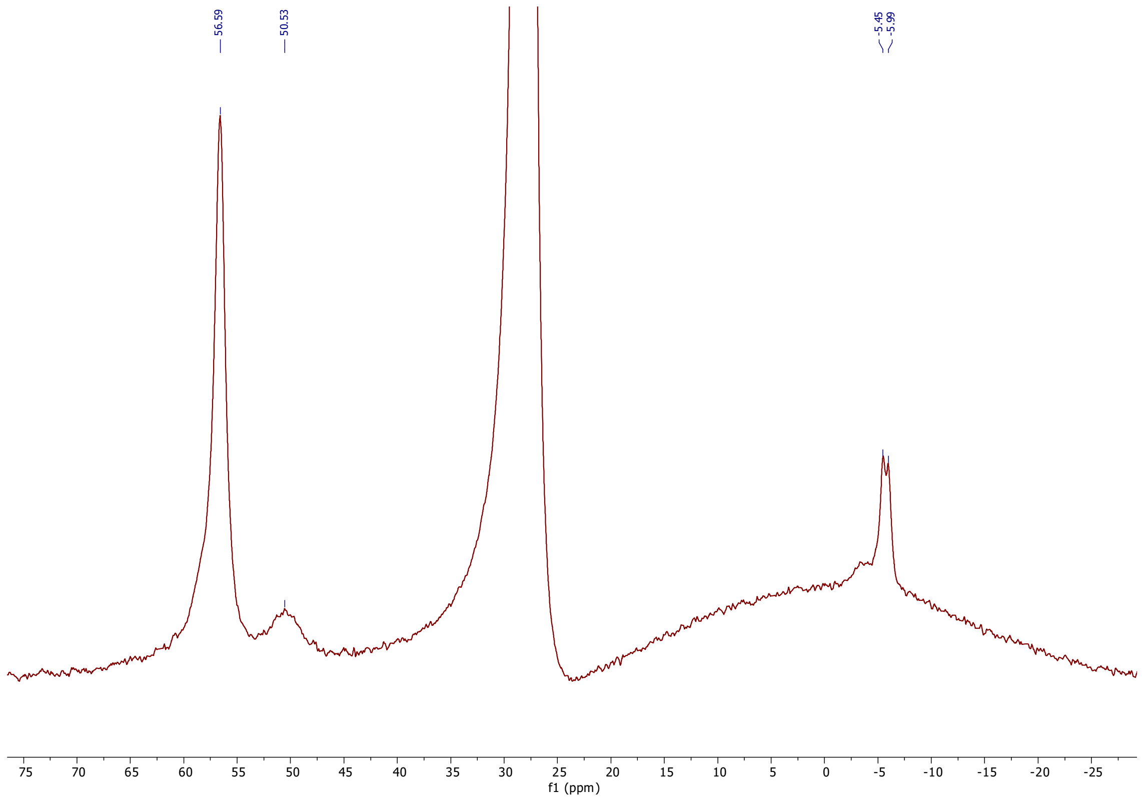

Figure S53. ^{11}B NMR spectrum (160 MHz, C_6D_6) of the reaction mixture 15 minutes after addition of H-BBN.

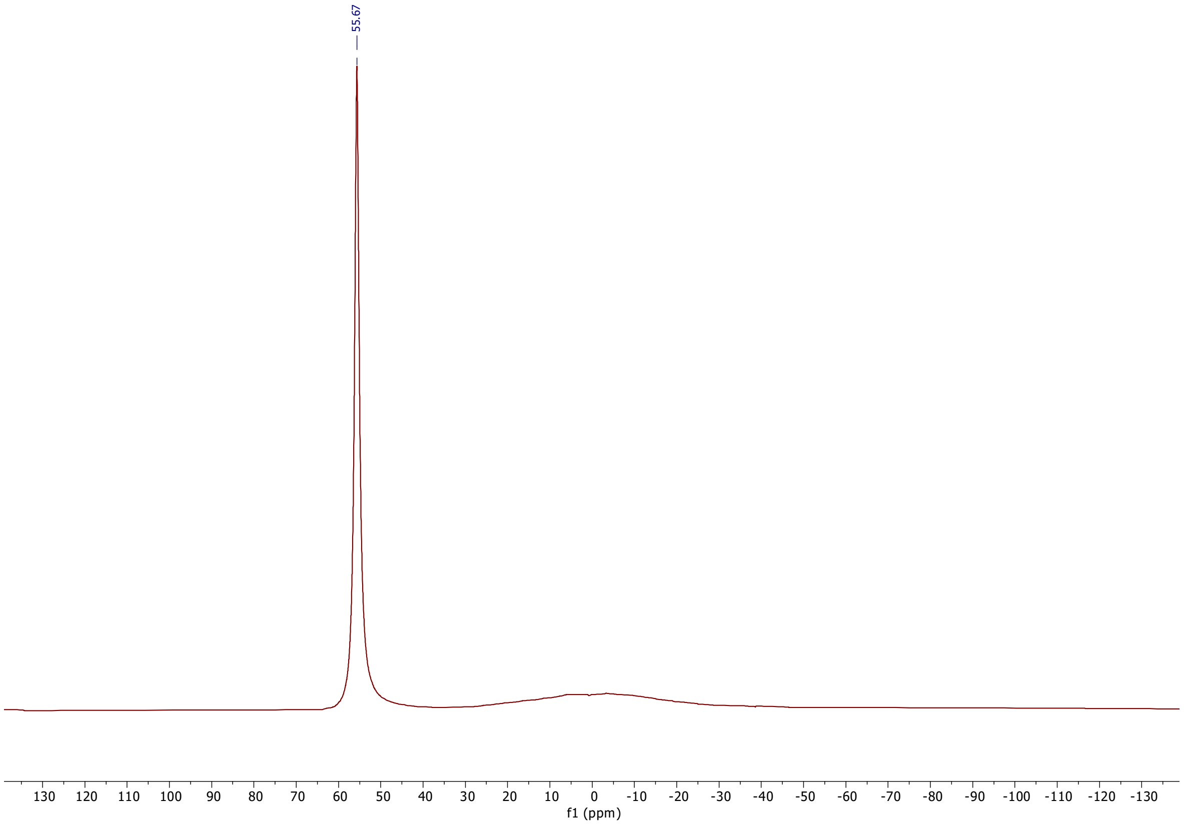

Figure S54. ^{11}B NMR spectrum (160 MHz, C_6D_6) of reaction mixture after heating.

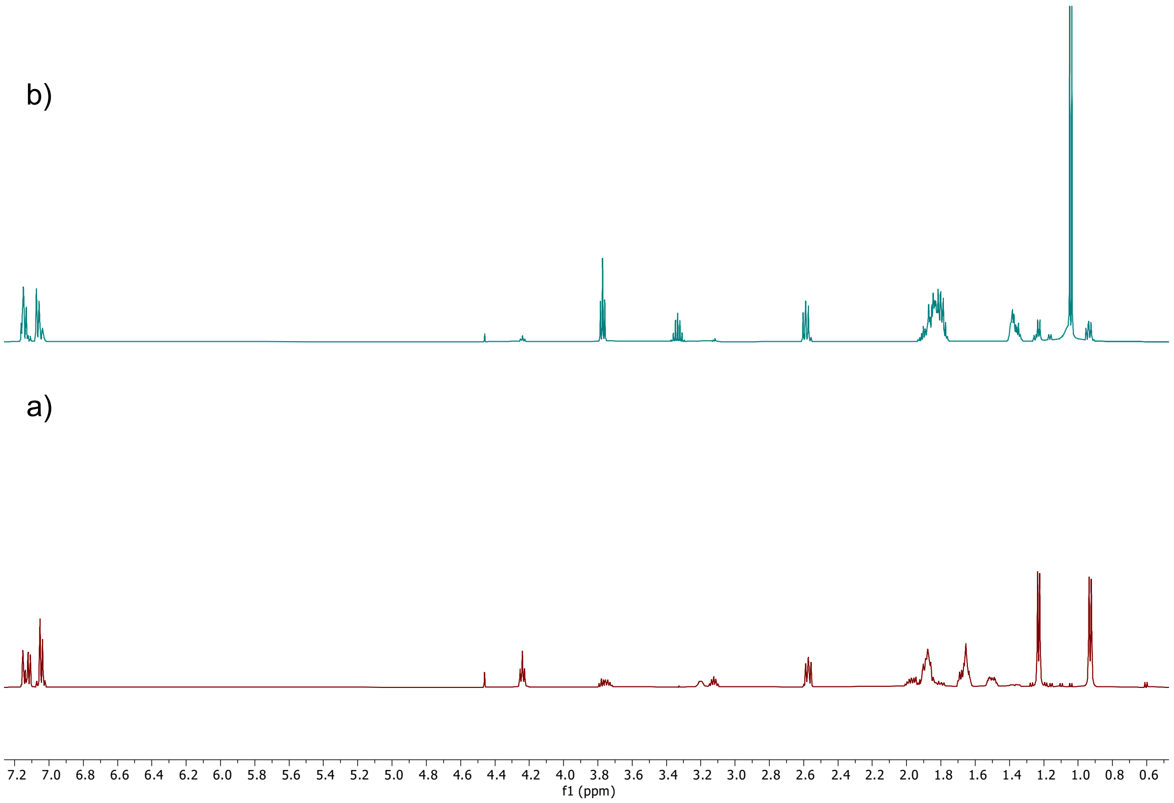

Figure S55. a) ^{1}H NMR spectrum (500 MHz, C_6D_6) of reaction mixture 15 minutes after addition of H-BBN. b) ^{1}H NMR spectrum (500 MHz, C_6D_6) of reaction mixture after heating. Note H_2 is observed at 4.5 ppm in a) and b).

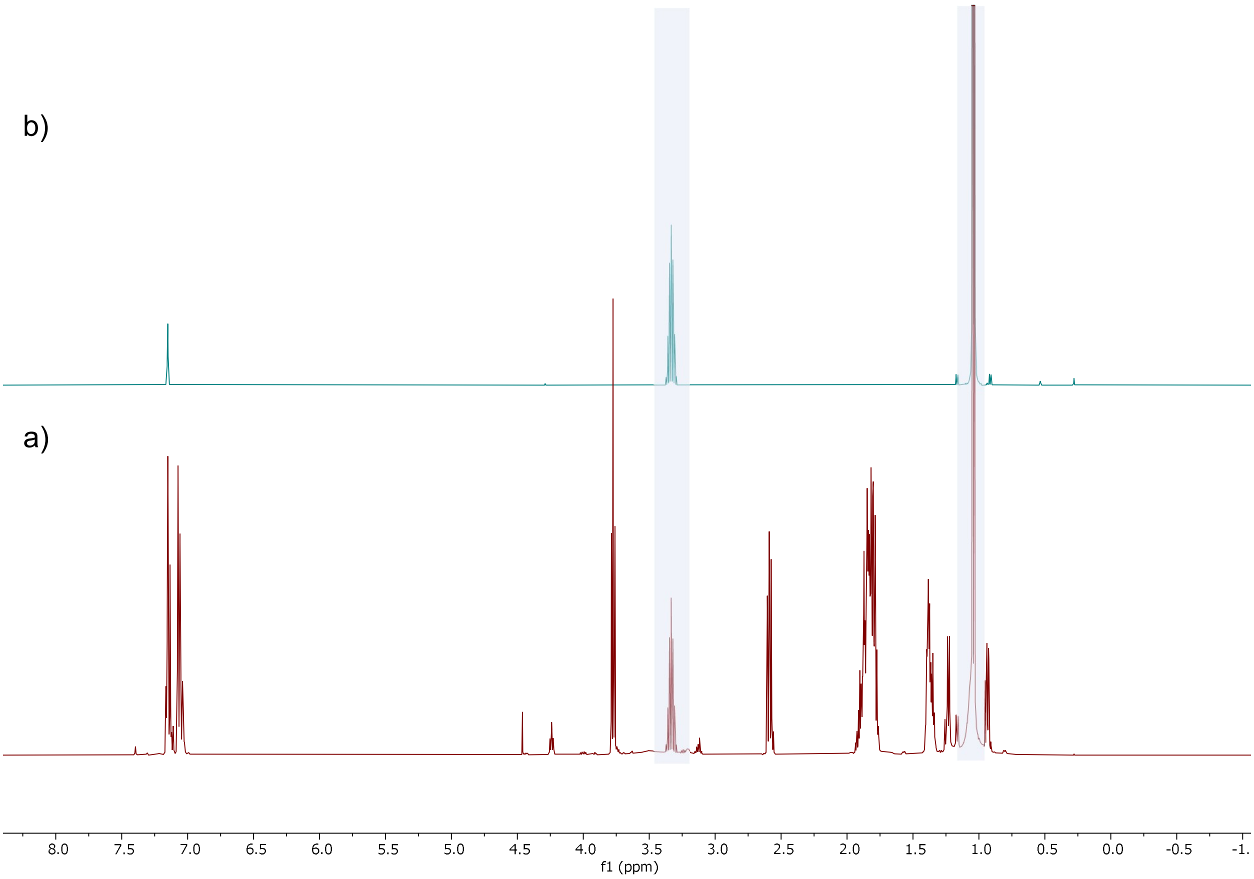

Figure S56. a) ^{1}H NMR spectrum (500 MHz, C_6D_6) of reaction mixture after heating. b) ^{1}H NMR spectrum (500 MHz, C_6D_6) of di*iso*propylcarbodiimide.

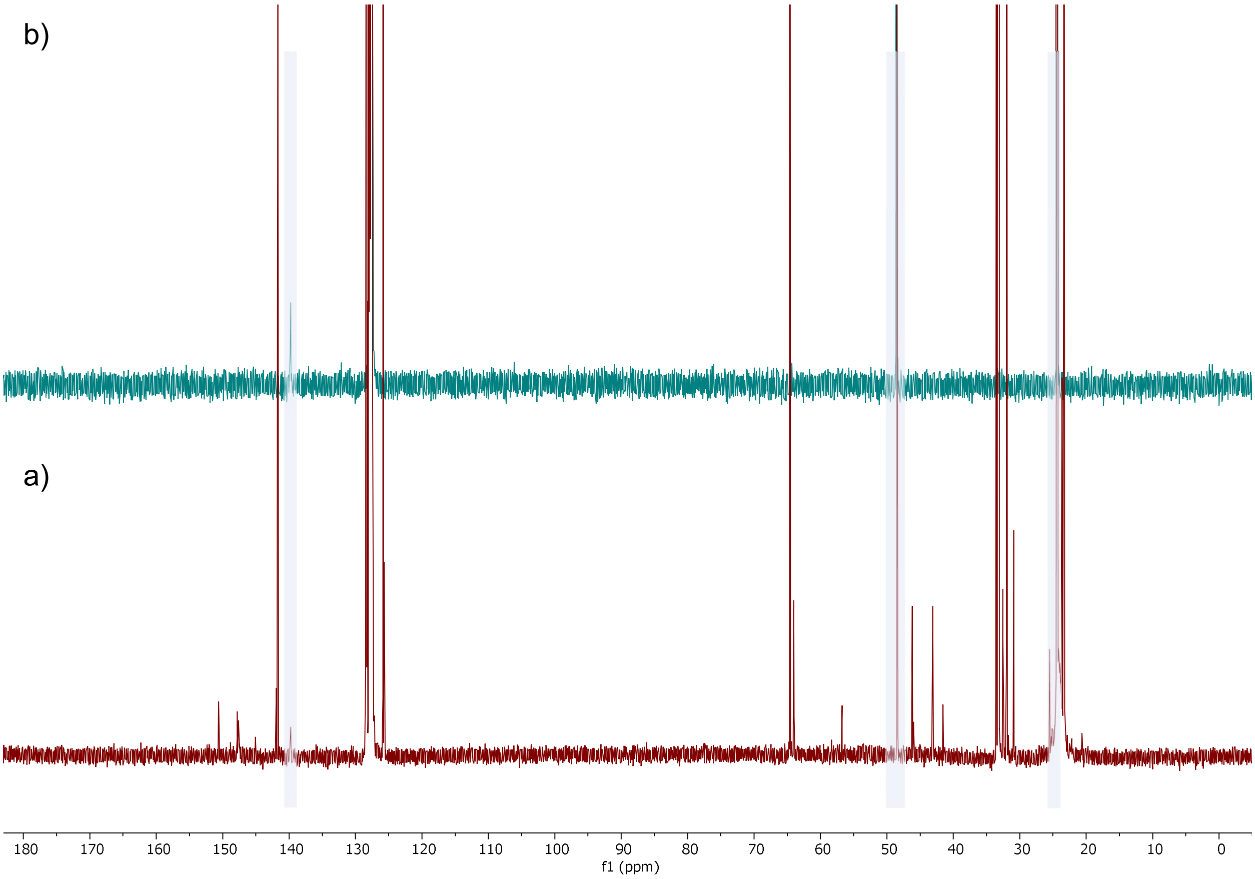

Figure S57. a) ^{13}C NMR spectrum (126 MHz, C_6D_6) of reaction mixture after heating. b) ^{13}C NMR spectrum (126 MHz, C_6D_6) of di*iso*propylcarbodiimide.

Dehydrocoupling and DIC elimination of secondary isourea

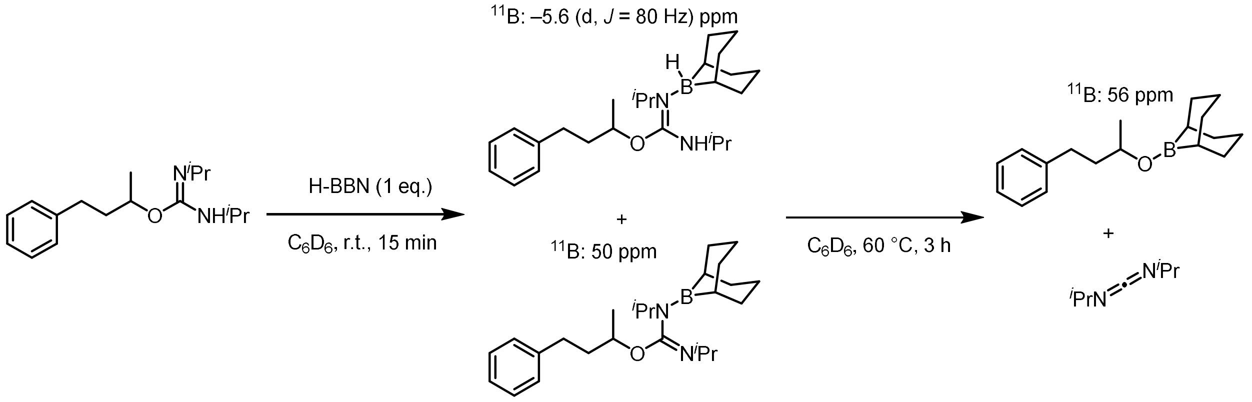

In a J-Young NMR tube, to a solution of (±)-*O*-(4-phenyl-2-butyl)-*N,N'*-diisopropylisourea (0.100 mmol, 28.0 mg) in C_6D_6 (0.600 mL) was added H-BBN dimer (50.0 μmol, 12.2 mg), and the mixture was analysed by NMR spectroscopy (after ca. 15 minutes). The mixture then was heated at 60 °C for 3 hours and analysed by NMR spectroscopy.

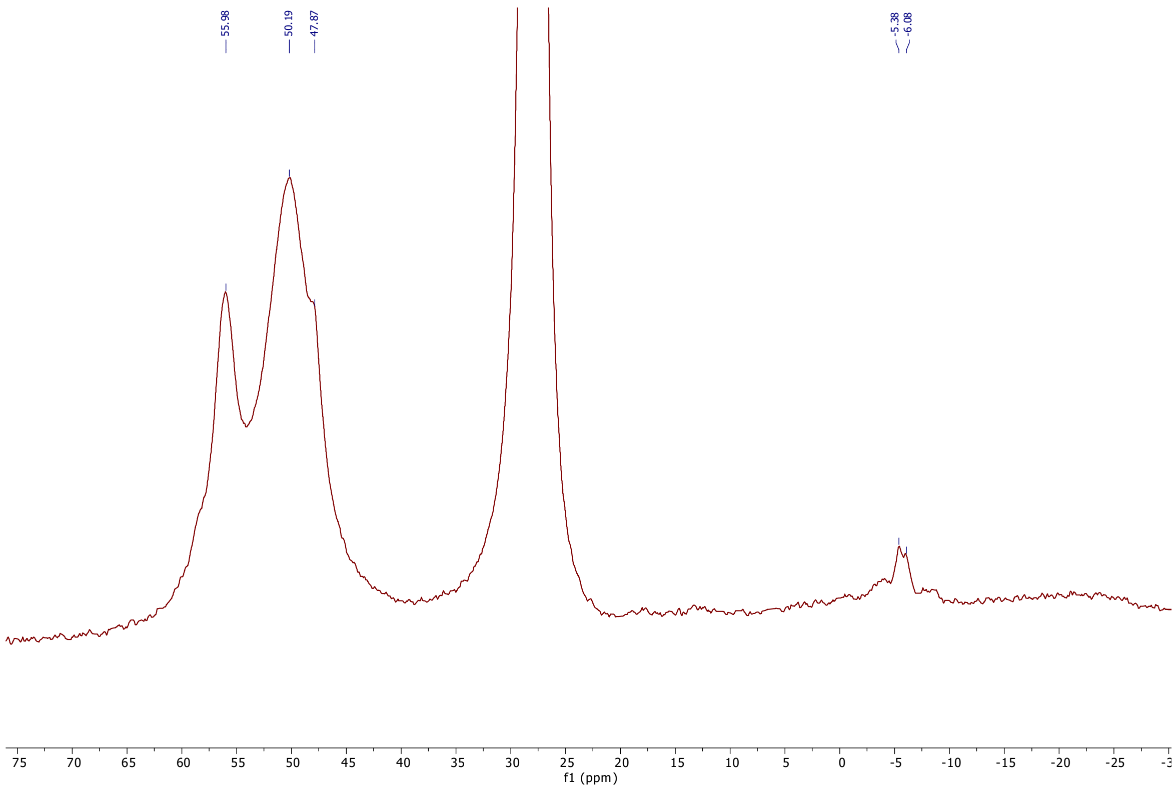

Figure S58. ^{11}B NMR spectrum (160 MHz, C_6D_6) of reaction mixture after addition of H-BBN.

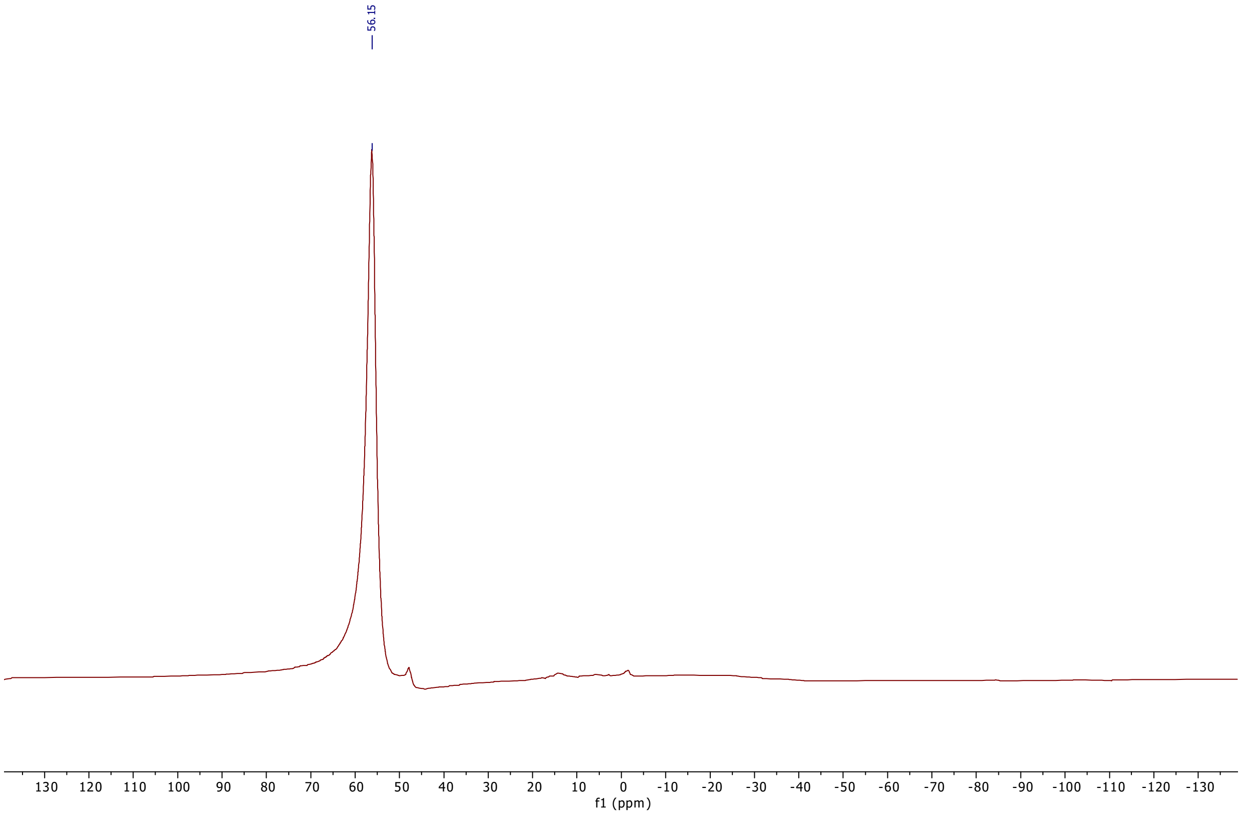

Figure S59. ^{11}B NMR spectrum (160 MHz, C_6D_6) of reaction mixture after heating.

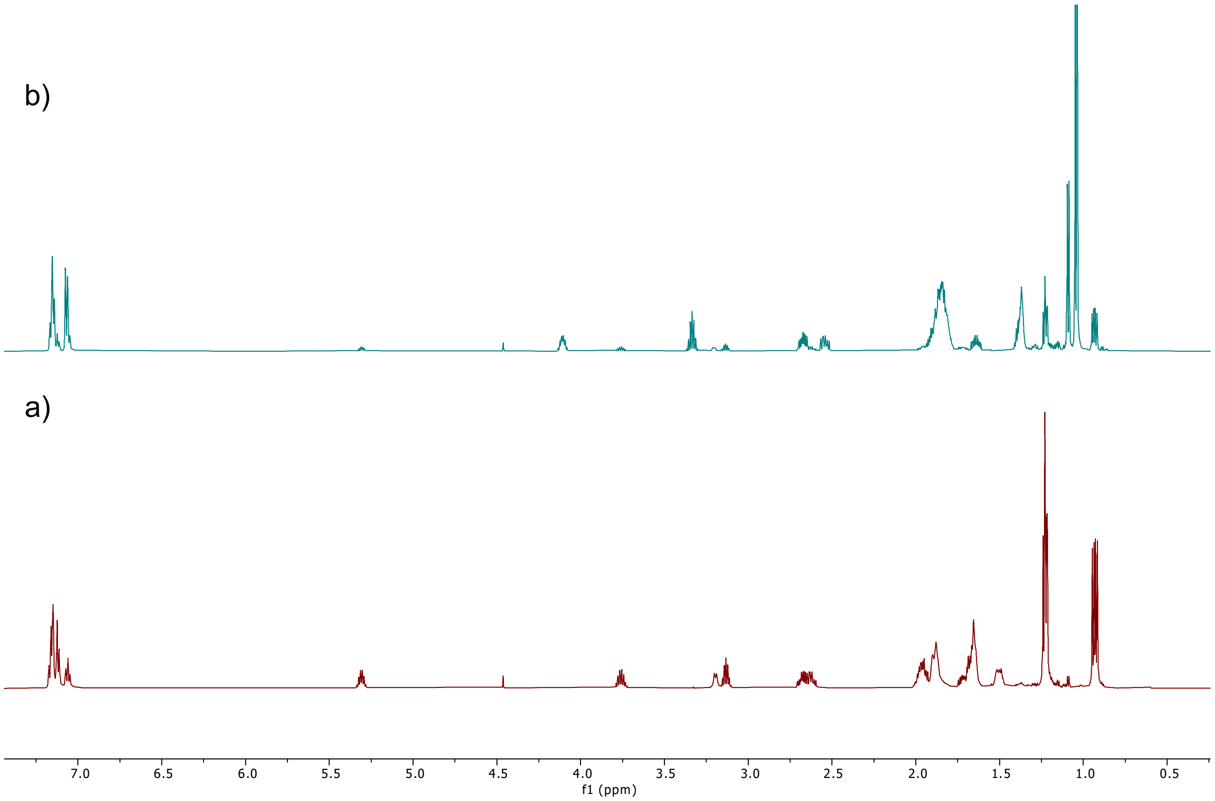

Figure S60. a) ^{1}H NMR spectrum (500 MHz, C_6D_6) of reaction mixture 15 minutes after addition of H-BBN. b) ^{1}H NMR spectrum (500 MHz, C_6D_6) of reaction mixture after heating. Note H_2 is observed at 4.5 ppm in a) and b).

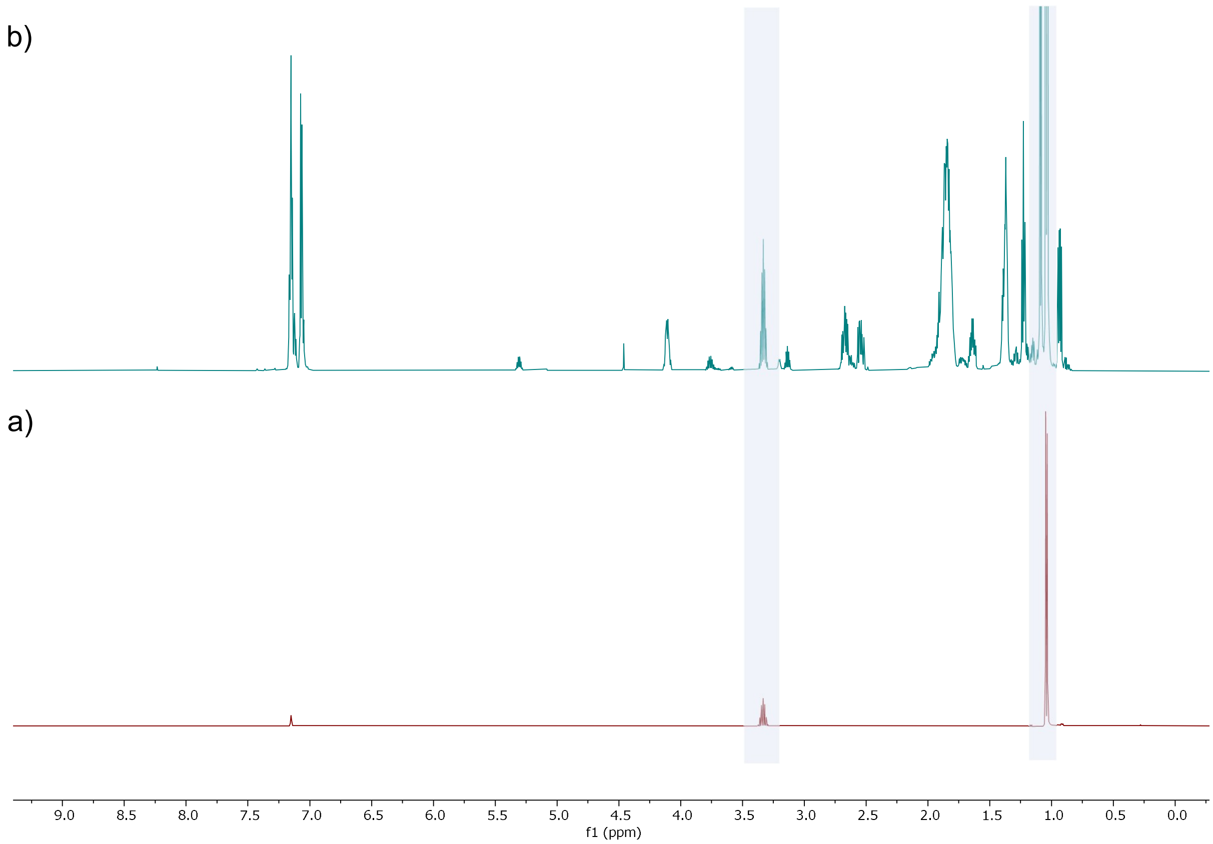

Figure S61. a) ^{1}H NMR spectrum (500 MHz, C_6D_6) of reaction mixture 15 minutes after heating. b) ^{1}H NMR spectrum (500 MHz, C_6D_6) of di*iso*propylcarbodiimide.

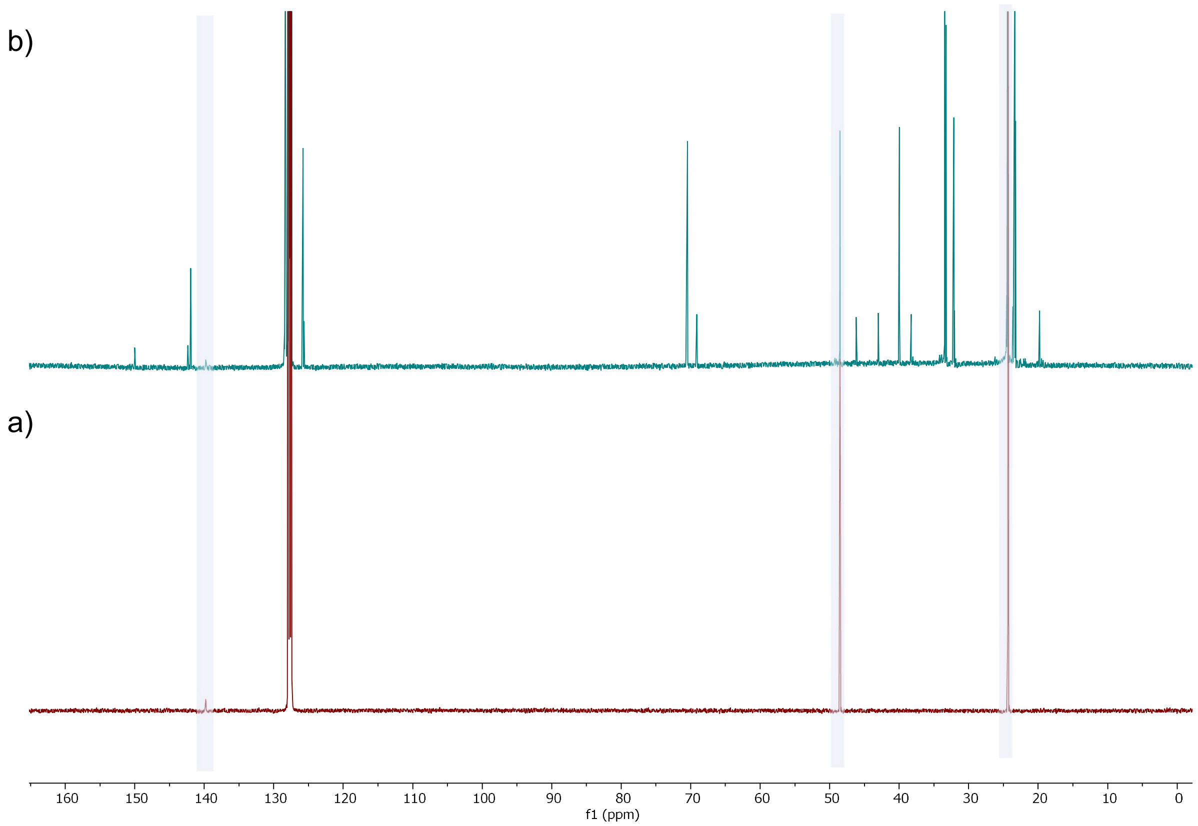

Figure S62. a) ^{13}C NMR spectrum (126 MHz, C_6D_6) of reaction mixture after heating. b) ^{13}C NMR spectrum (126 MHz, C_6D_6) of di*iso*propylcarbodiimide.

Independent Borinic Ester Synthesis

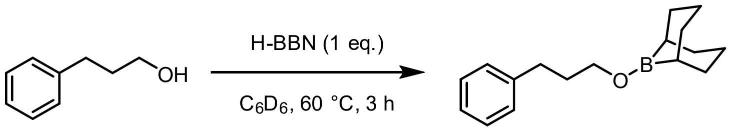

A solution of 3-phenylpropan-1-ol (14.0 μL, 0.100 mmol) and H-BBN (12.0 mg, 0.100 mmol) in C_6D_6 (0.600 mmol) was heated at 60 °C for 3 hours. After cooling to room temperature, the mixture was analysed by NMR spectroscopy which revealed full conversion to *B*-(*O*-1-(3-phenyl)propoxy)-9-borabicyclo[3.3.1]nonane (δ ^{11}B = 56 ppm), matching the *in situ* observed species from the O-alkyl-isourea + H-BBN reaction.

^{1}H NMR (500 MHz, C_6D_6) δ 7.18 – 7.10 (m, 2H), 7.09 – 7.02 (m, 3H), 3.78 (t, *J* = 6.4 Hz, 2H), 2.62 – 2.53 (m, 2H), 1.94 – 1.74 (m, 12H), 1.36 (ddd, *J* = 13.4, 9.7, 6.8 Hz, 4H).

^{11}B NMR (160 MHz, C_6D_6) δ 56.6.

^{13}C NMR (126 MHz, C_6D_6) δ 142.0, 128.8, 128.7, 126.2, 65.0, 33.9, 33.6, 32.4, 24.8 (br), 23.7.

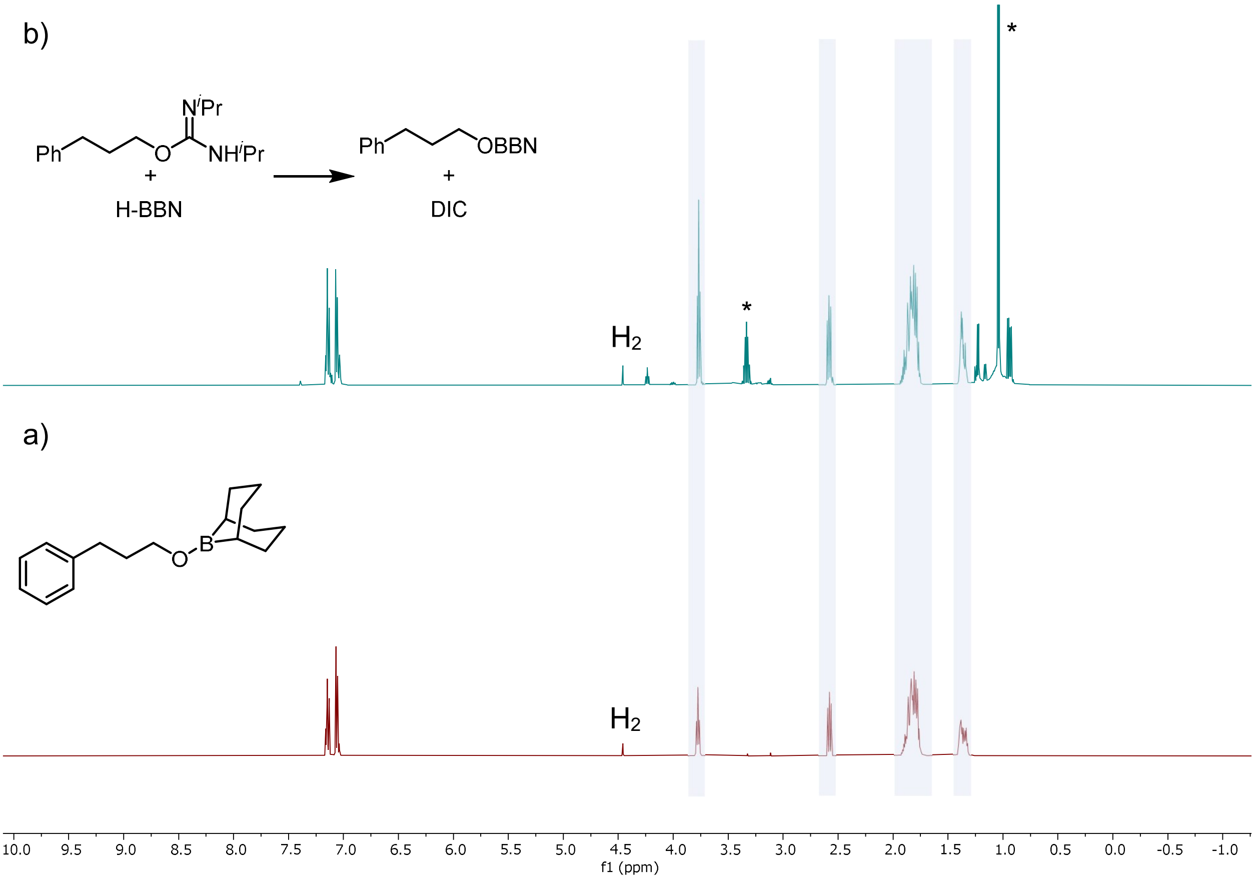

Figure S63. Overlaid ^{1}H NMR spectra (500 MHz, C_6D_6) of a) independently synthesised *B*-(*O*-1-(3-phenyl)propoxy)-9-borabicyclo[3.3.1]nonane b) reaction of *O*-(3-phenyl-1-propyl)-*N,N'*-diisopropylisourea with H-BBN. Blue = *B*-(*O*-1-(3-phenyl)propoxy)-9-borabicyclo[3.3.1]nonane. * = DIC.

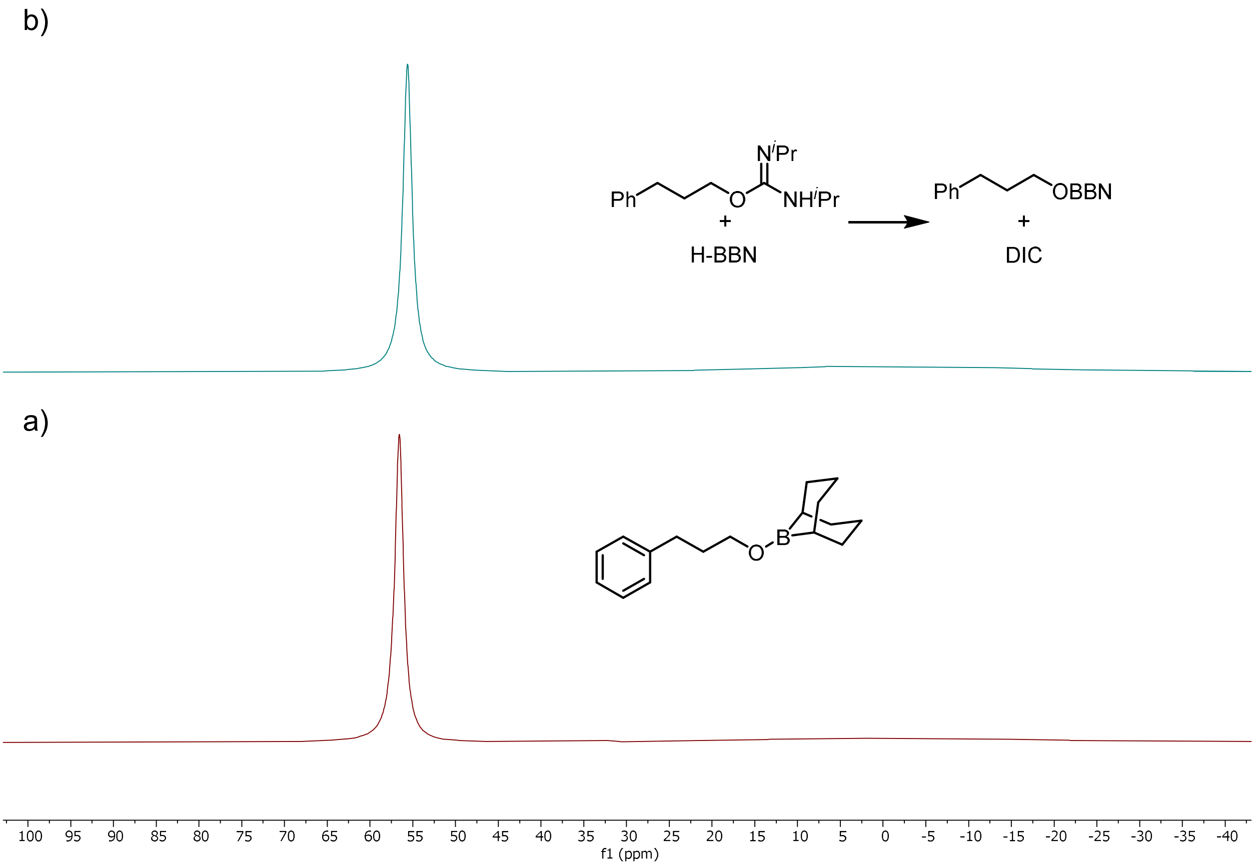

Figure S64. Overlaid ^{11}B NMR spectra (160 MHz, C_6D_6) of a) independently synthesised *B*-(*O*-1-(3-phenyl)propoxy)-9-borabicyclo[3.3.1]nonane b) reaction of *O*-(3-phenyl-1-propyl)-*N,N'*-diisopropylisourea with H-BBN.

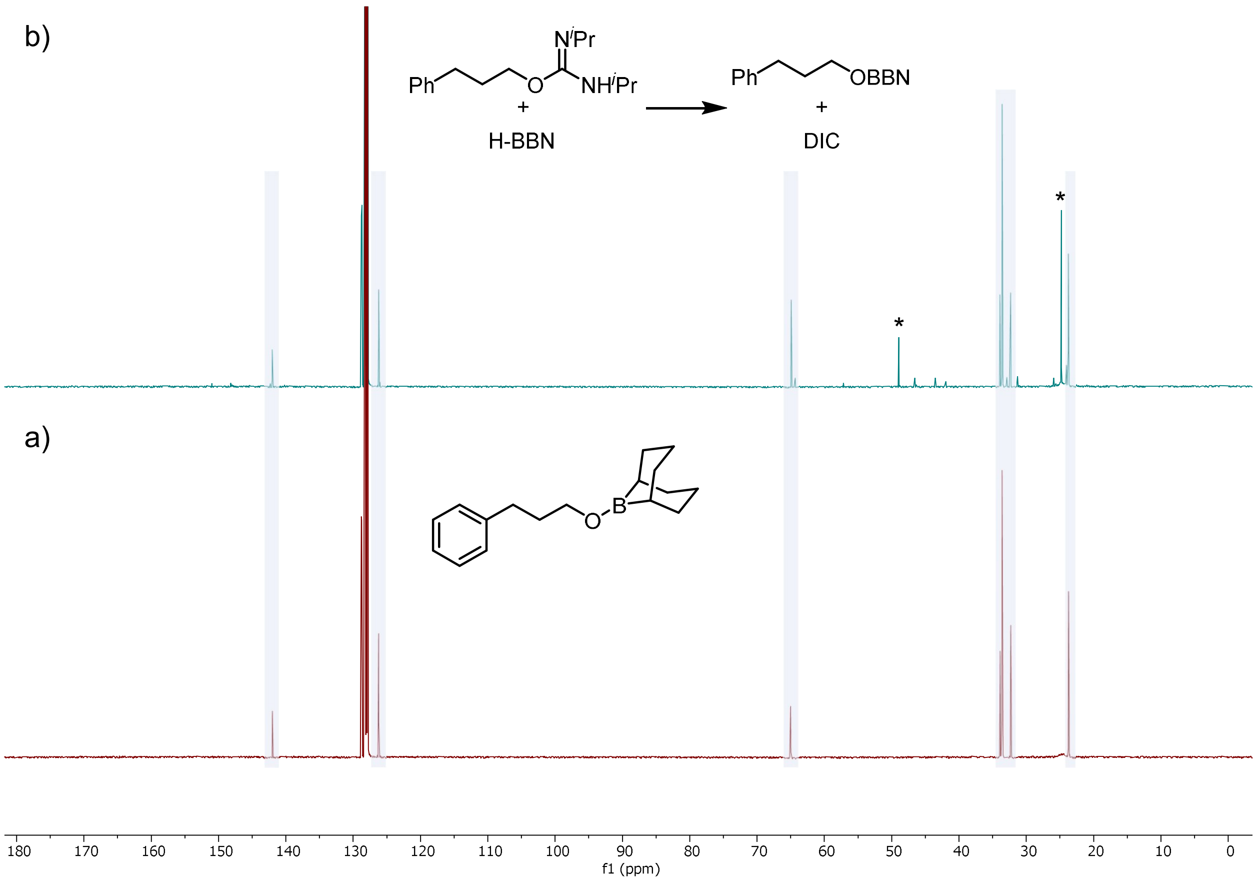

Figure S65. Overlaid ^{13}C NMR spectra (126 MHz, C_6D_6) of a) independently synthesised *B*-(*O*-1-(3-phenyl)propoxy)-9-borabicyclo[3.3.1]nonane b) reaction of *O*-(3-phenyl-1-propyl)-*N,N'*-diisopropylisourea with H-BBN. Blue = *B*-(*O*-1-(3-phenyl)propoxy)-9-borabicyclo[3.3.1]nonane. * = DIC.

DCC Elimination

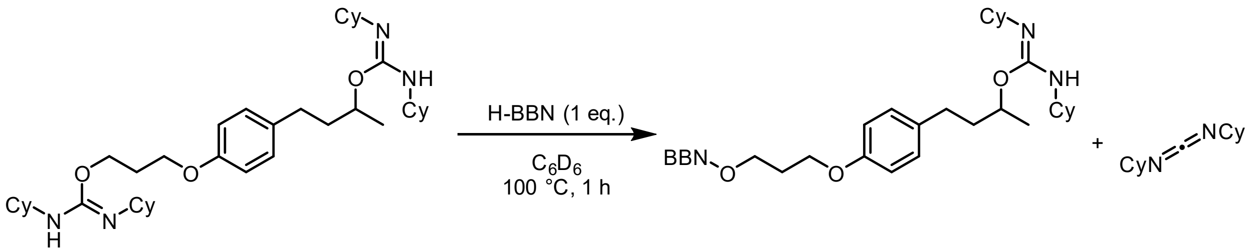

In a catalysis vial, a solution of 4-(4-(3-*O*-(*N*,*N'*-dicyclohexylcarbamimidyl)-propoxy)phenyl)butan-2-*O*-*N*,*N'*-dicyclohexylcarbamimidate (64.0 mg, 0.100 mmol), and H-BBN (12.0 mg, 0.100 mmol) in C_6D_6 (0.600 mL) was heated at 100 °C for 1 hour. The mixture was analysed by NMR spectroscopy to show formation of DCC and the borinic ester.

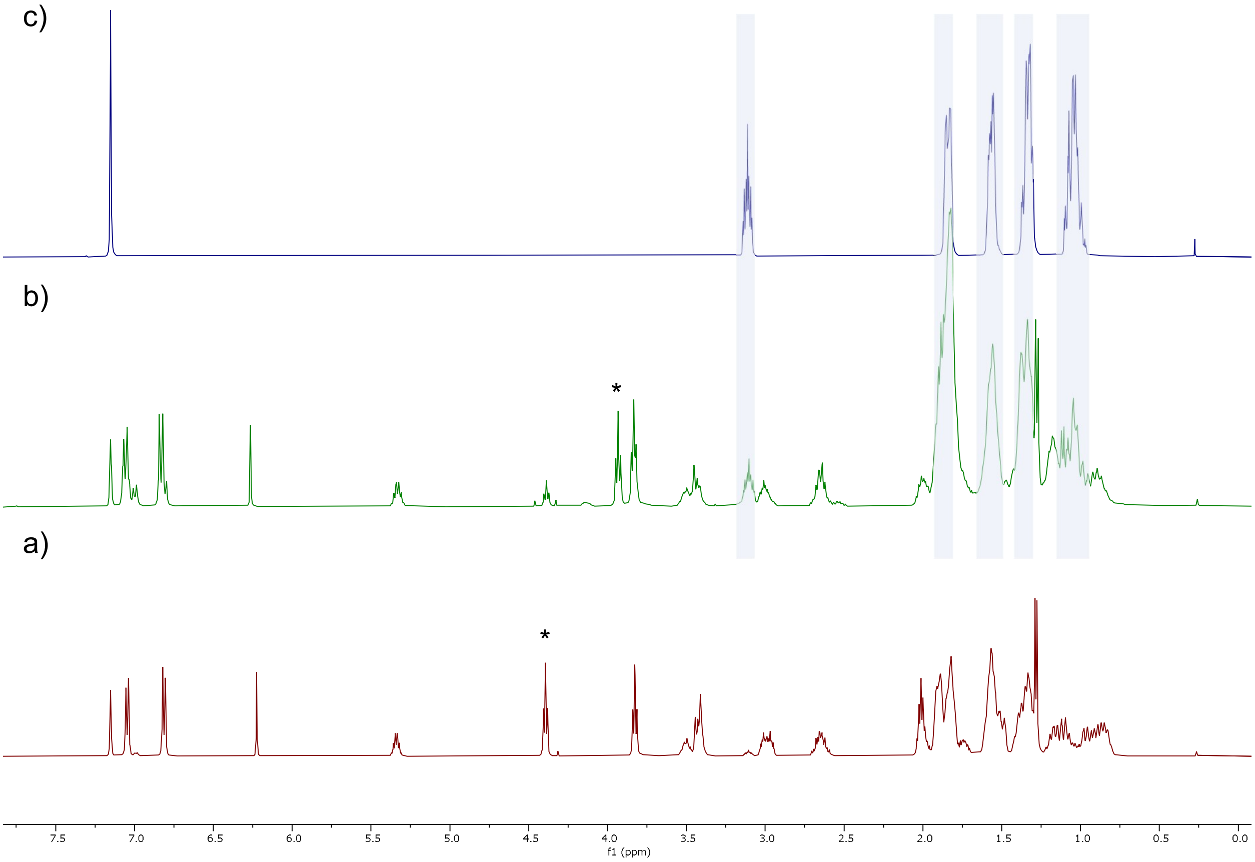

Figure S66. Overlaid ^{1}H NMR spectra (500 MHz, C_6D_6) of a) of 4-(4-(3-*O*-(*N*,*N'*-dicyclohexylcarbamimidyl)-propoxy)phenyl)butan-2-*O*-*N*,*N'*-dicyclohexylcarbamimidate b) reaction of 4-(4-(3-*O*-(*N*,*N'*-dicyclohexylcarbamimidyl)-propoxy)phenyl)butan-2-*O*-*N*,*N'*-dicyclohexylcarbamimidate with H-BBN. c) DCC. Blue = DCC. * primary OC<u>H</u>$_2$.

Reaction of H-BBN with Diisopropylurea

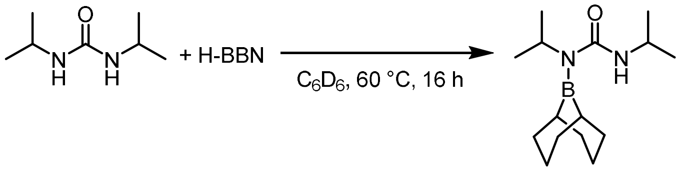

A suspension of *N*,*N'*-di*iso*propyl urea (14.0 mg, 0.100 mmol) and H-BBN (12.0 mg, 0.100 mmol) in C_6D_6 (0.600 mL) was heated at 60 °C for 16 hours. The mixture was analysed by NMR spectroscopy, revealing full conversion to *N*-(*B*-9-borabicyclo(3.3.1)nonanyl)-*N*,*N'*-di*iso*propyl urea.

^{1}H NMR (500 MHz, C_6D_6) δ 4.57 (d, *J* = 7.6 Hz, 1H), 3.95 (dsept., *J* = 8.0, 6.6 Hz, 1H), 3.82 (sept., *J* = 6.8 Hz, 1H), 2.06 – 1.84 (m, 7H), 1.77 (ddt, *J* = 18.5, 10.7, 5.1 Hz, 4H), 1.56 (q, *J* = 4.3 Hz, 2H), 1.49 – 1.39 (m, 2H), 1.35 (d, *J* = 6.8 Hz, 6H), 0.88 (d, *J* = 6.6 Hz, 6H).

^{11}B NMR (160 MHz, C_6D_6) δ 50.3.

^{13}C NMR (126 MHz, C_6D_6) δ 158.4, 49.0, 42.8, 41.9, 33.8, 24.3 (br), 23.8, 23.7, 23.4, 22.5.

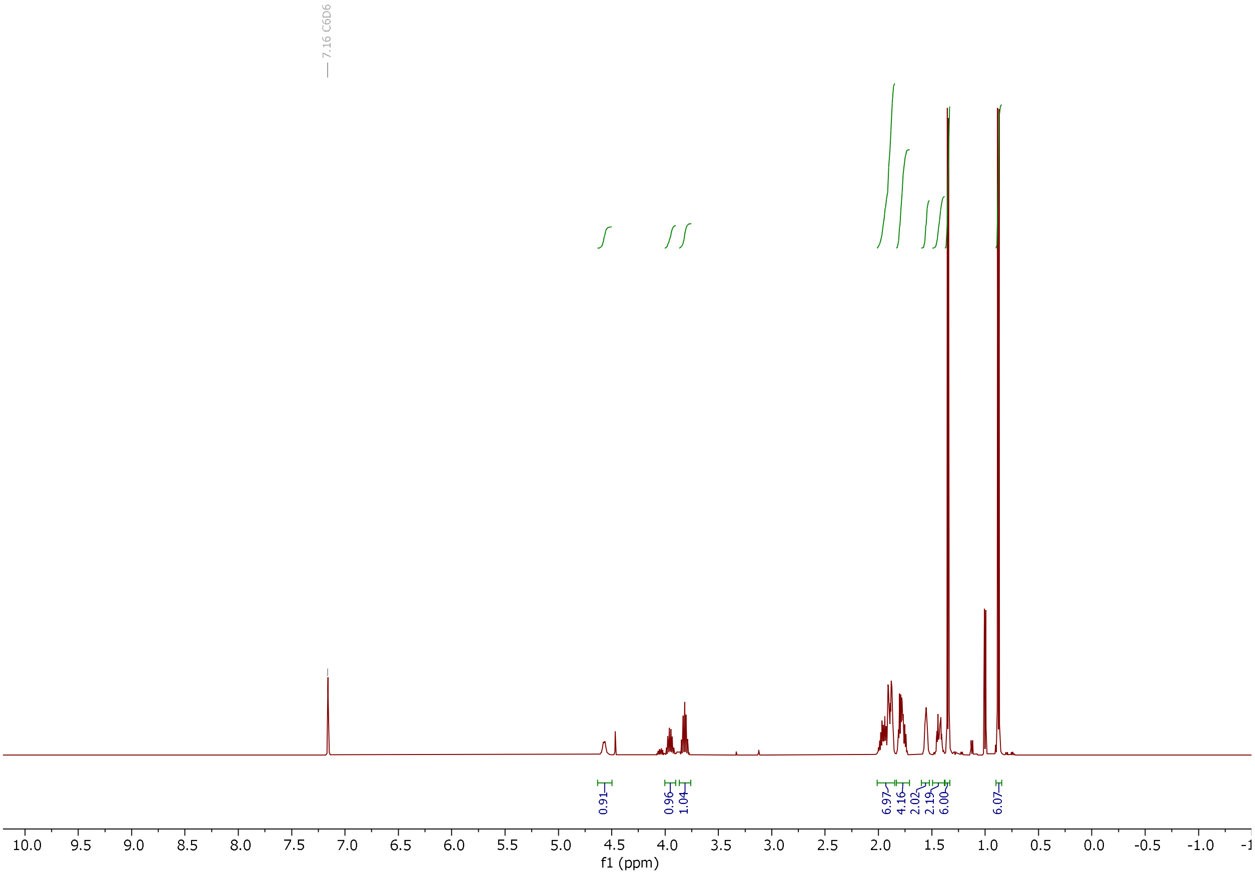

Figure S67. ^{1}H NMR (500 MHz, C_6D_6) spectrum of reaction between of *N*,*N'*-di*iso*propyl urea and H-BBN.

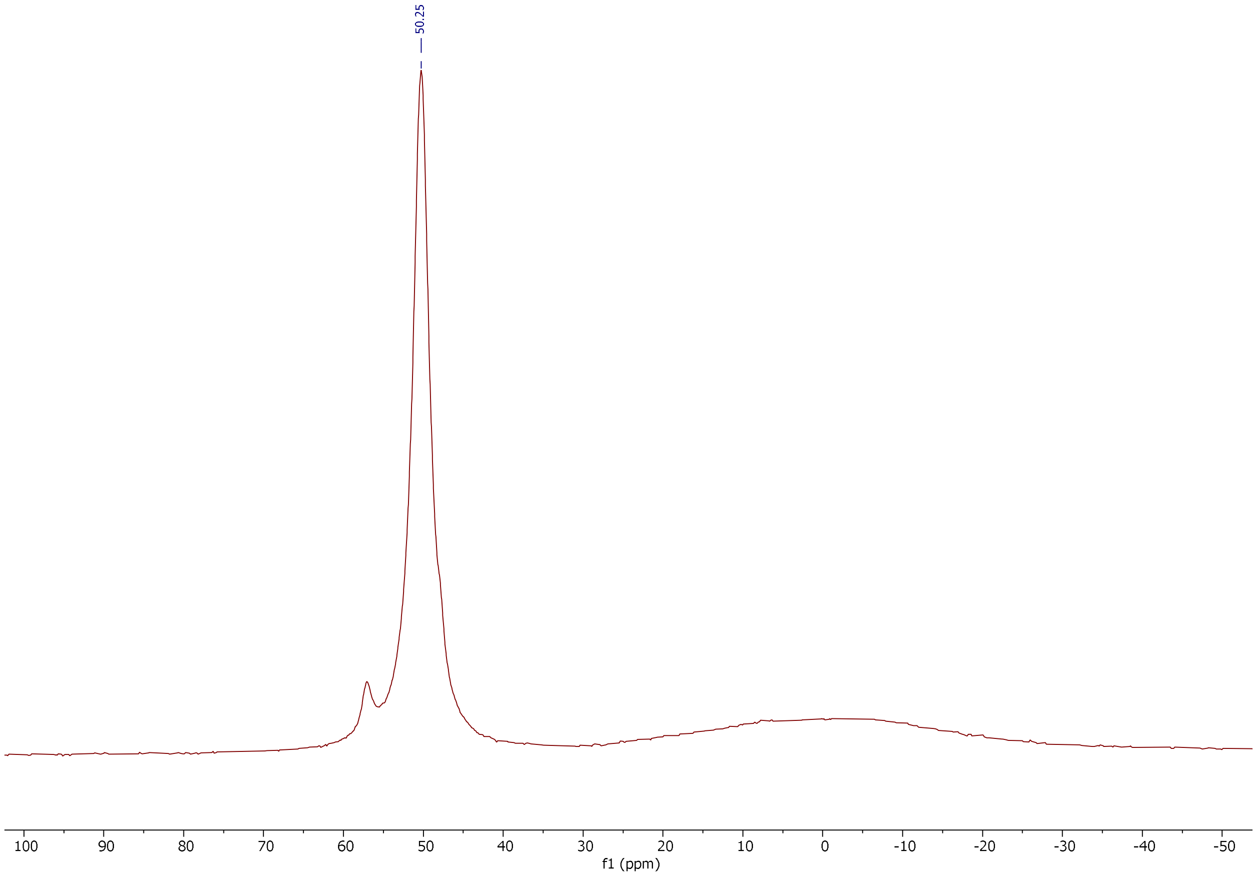

Figure S68. ^{11}B NMR (160 MHz, C_6D_6) spectrum of the reaction between *N*,*N'*-di*iso*propyl urea and H-BBN. Minor impurity at 56 ppm attributed to reaction of H-BBN with water present in urea sample.

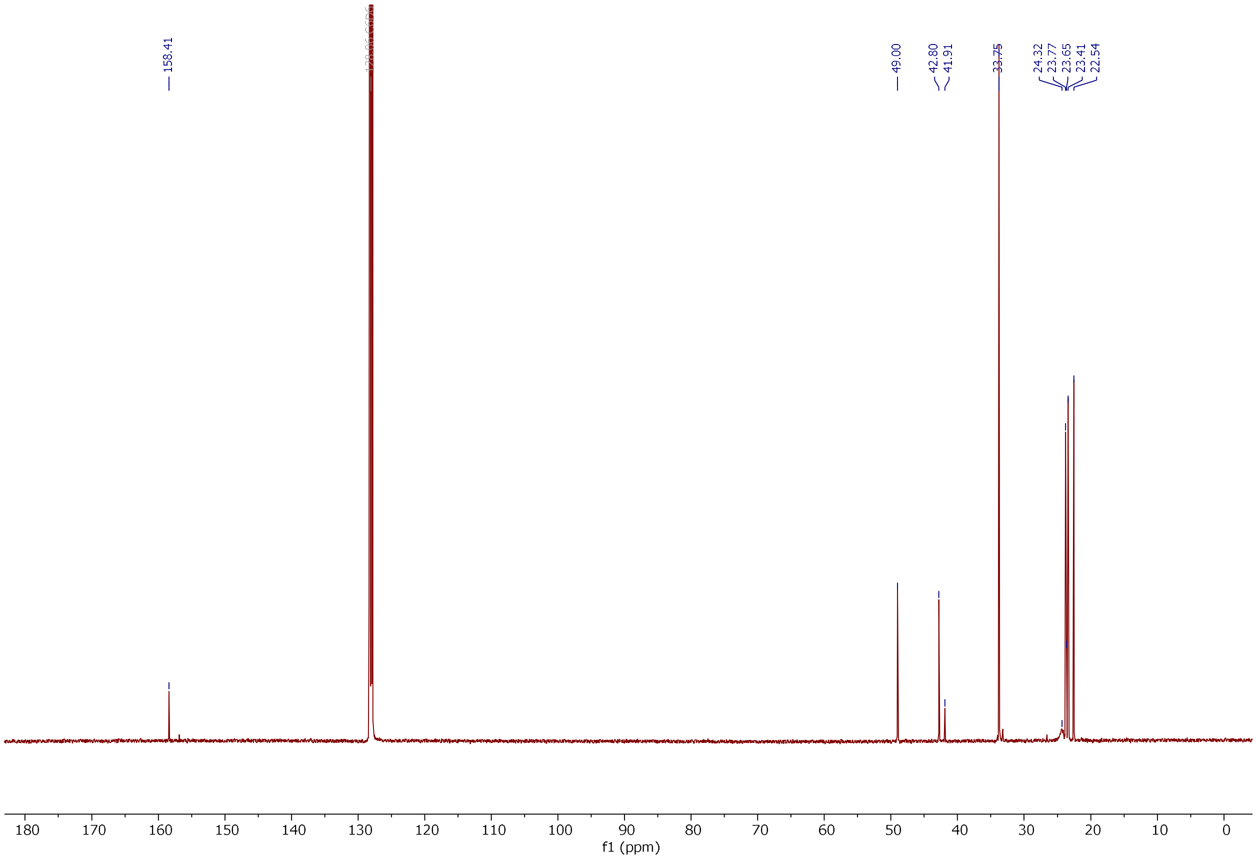

Figure S69. ^{13}C NMR (126 MHz, C_6D_6) spectrum of the reaction between *N*,*N'*-di*iso*propyl urea and H-BBN.

Reaction of DIC with MeO-BBN

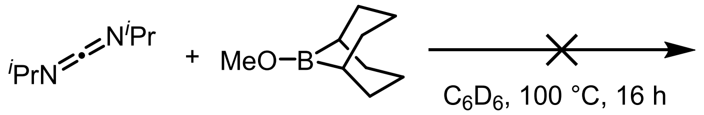

A solution of *B*-methoxy-9-borabicyclo[3.3.1]nonane (15.2 mg, 0.100 mmol) and di*iso*propyl carbodiimide (16.0 μL, 0.100 mmol) in C_6D_6 (0.600 mL) was heated at 100 °C for 16 hours. The mixture was analysed by NMR spectroscopy, showing no conversion of either reagent.

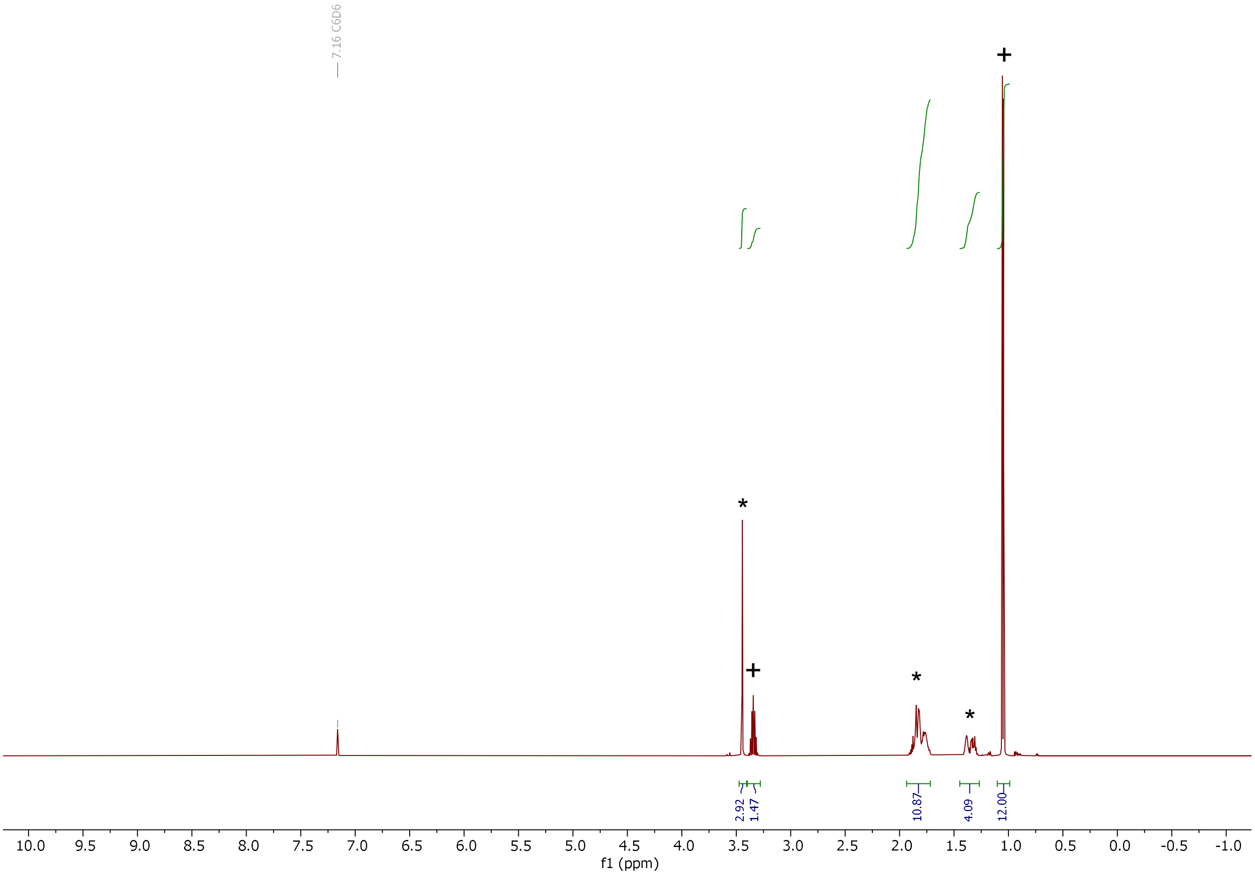

Figure S70. ^{1}H NMR (500 MHz, C_6D_6) spectrum of MeOBBN and DIC. * = MeOBBN. $^+$ = DIC.

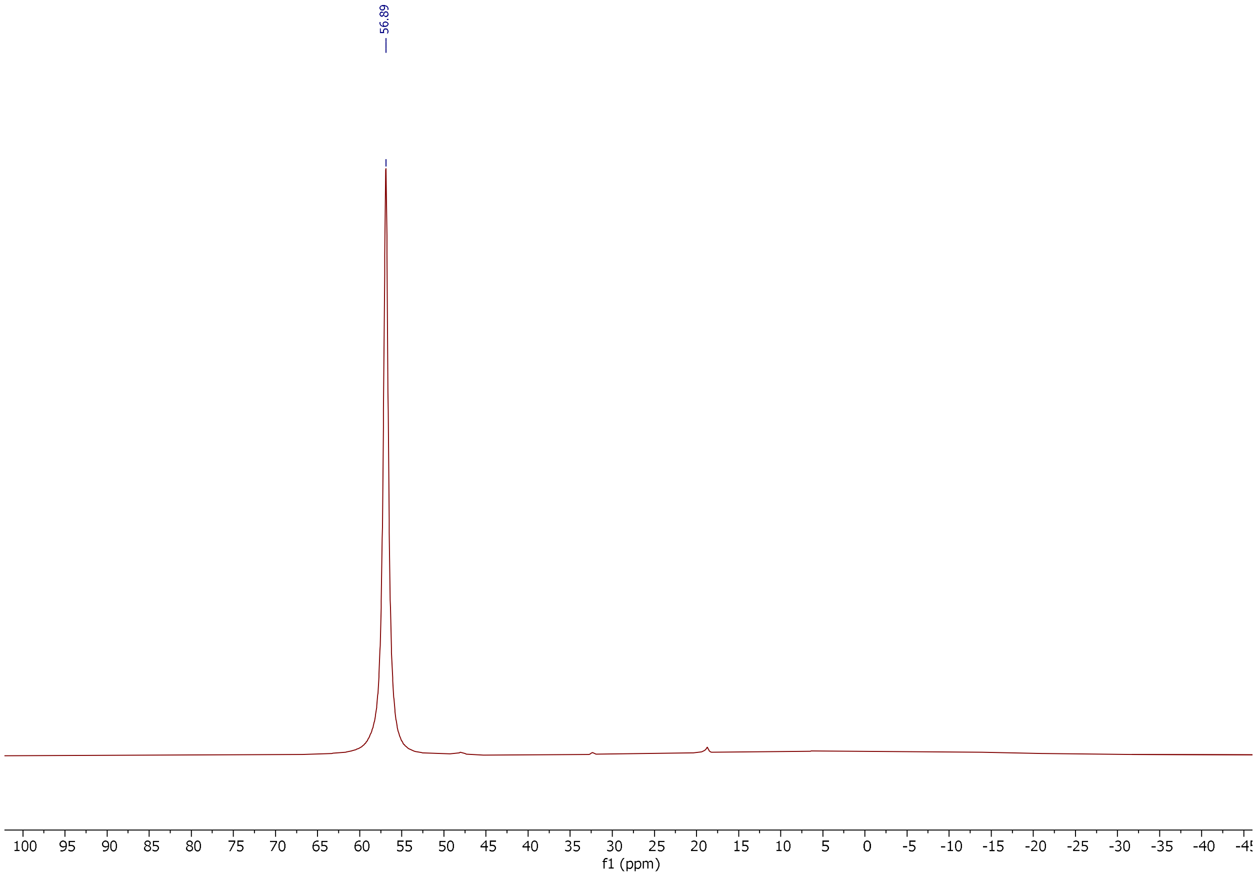

Figure S71. ^{11}B NMR (160 MHz, C_6D_6) spectrum of MeOBBN and DIC.

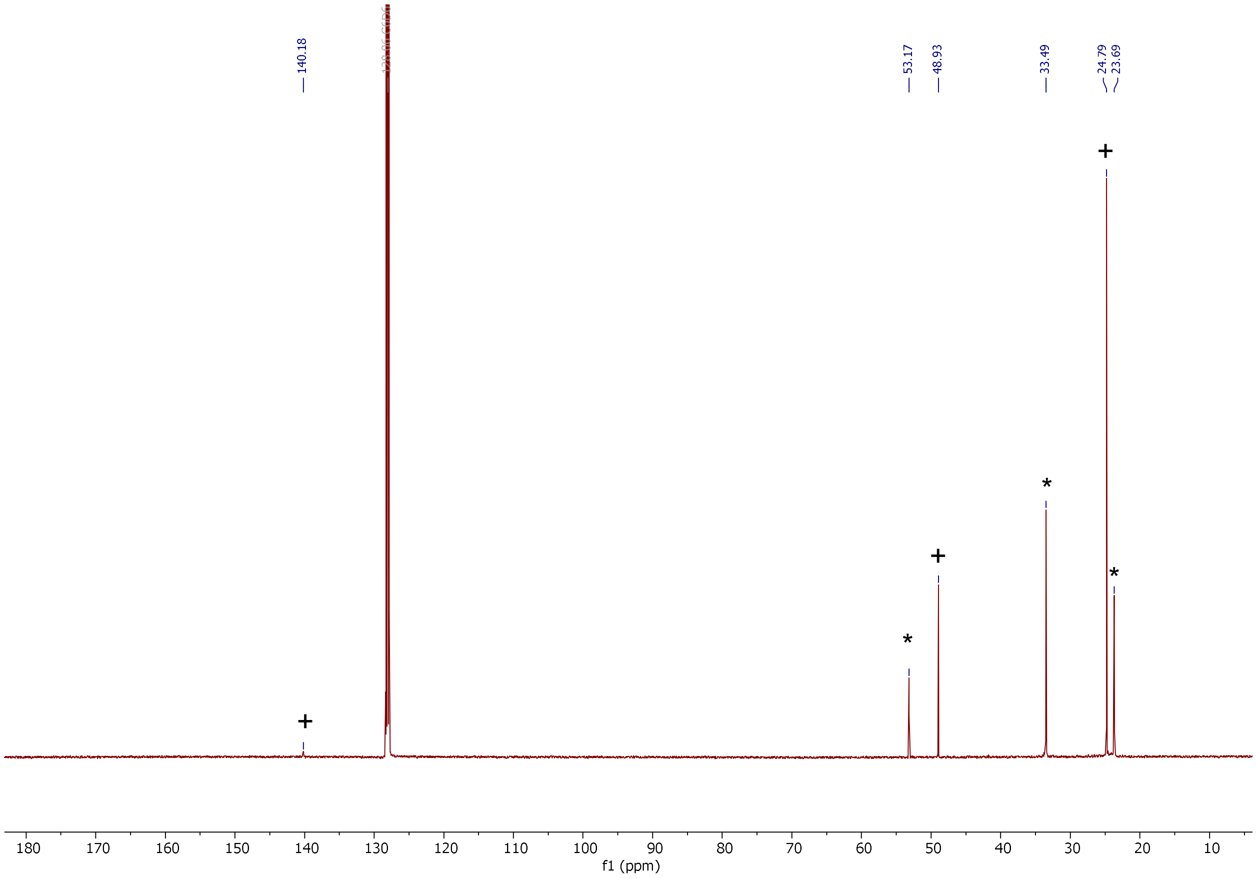

Figure S72. ^{13}C NMR (126 MHz, C_6D_6) spectrum of MeOBBN and DIC. * = MeOBBN. $^{+}$ = DIC.

Probing H/BBN Equilibrium

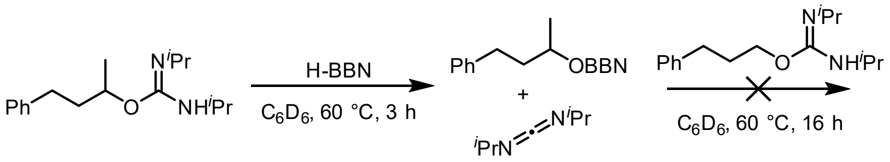

A solution of (±)-*O*-(2-(4-phenyl)butyl)-*N*,*N'*-di*iso*propylisourea (28.0 mg, 0.100 mmol) and H-BBN (12.0 mg, 0.100 mmol) in C_6D_6 (0.600 mL) was heated at 60 °C for 3 hours, then analysed by NMR spectroscopy, which showed conversion to the corresponding borinic ester (δ ^{11}B = 56 ppm) and DIC. *O*-(1-(3-Phenyl)propyl)-*N*,*N'*-di*iso*propylisourea (10.0 mg, 41.7 μmol) was added and the mixture heated at 60 °C for 16 hours. The mixture was analysed by NMR spectroscopy to show no exchange between the borinic ester and the isourea.

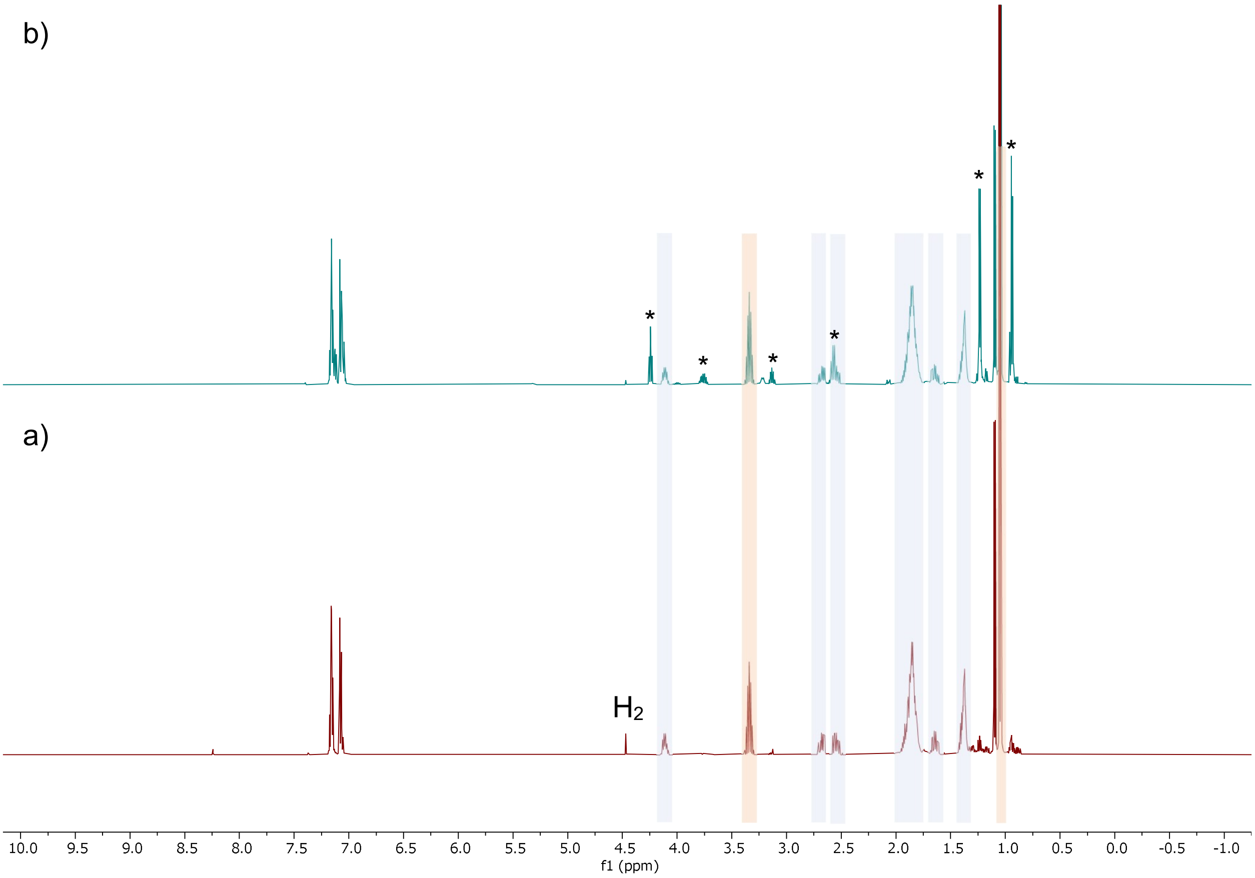

Figure S73. Overlaid ^{1}H NMR (500 MHz, C_6D_6) spectra of a) borinic ester formation b) after heating with *O*-(1-(3-phenyl)propyl)-*N*,*N'*-di*iso*propylisourea. Blue = borinic ester. Orange = DIC. * = *O*-(1-(3-Phenyl)propyl)-*N*,*N'*-di*iso*propylisourea.

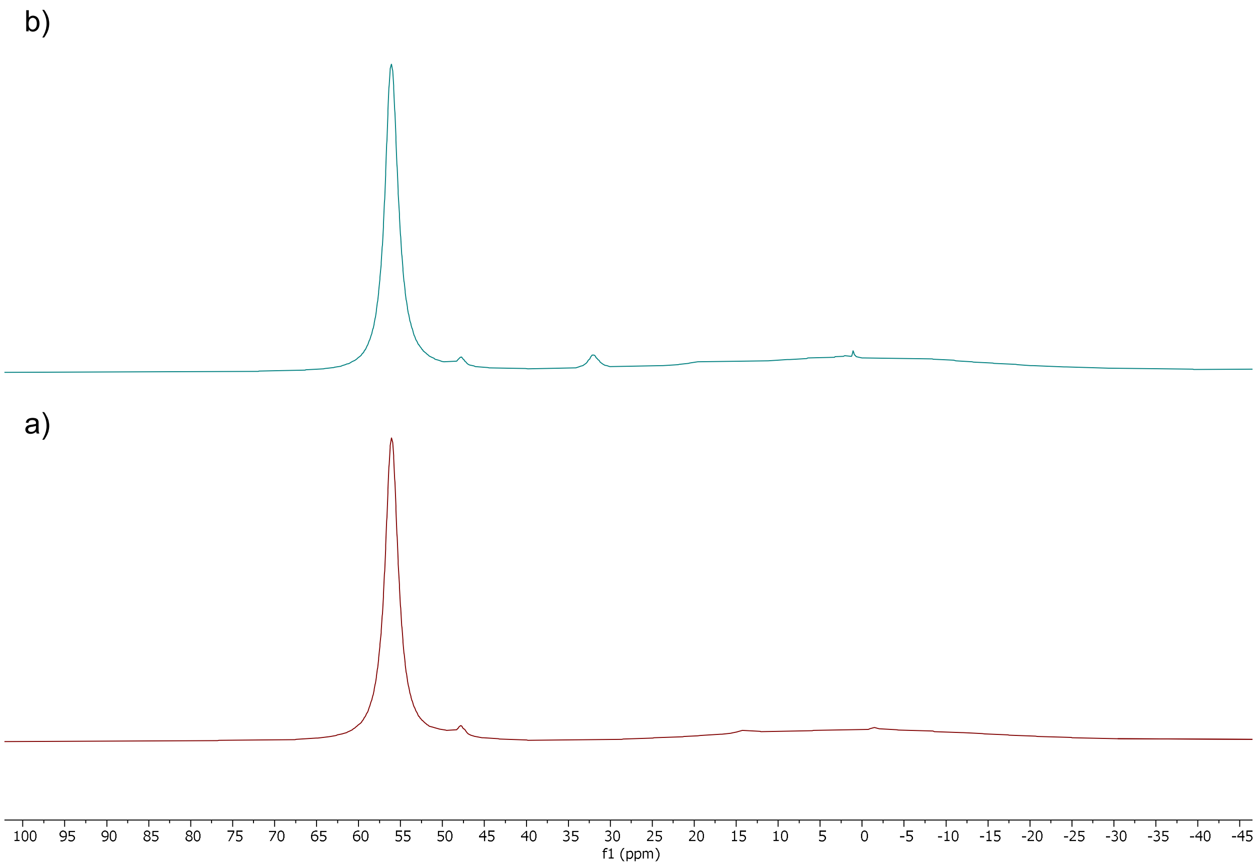

Figure S74. Overlaid ^{11}B NMR (160 MHz, C_6D_6) spectra of a) borinic ester formation b) after heating with *O*-(1-(3-phenyl)propyl)-*N*,*N'*-di*iso*propylisourea.

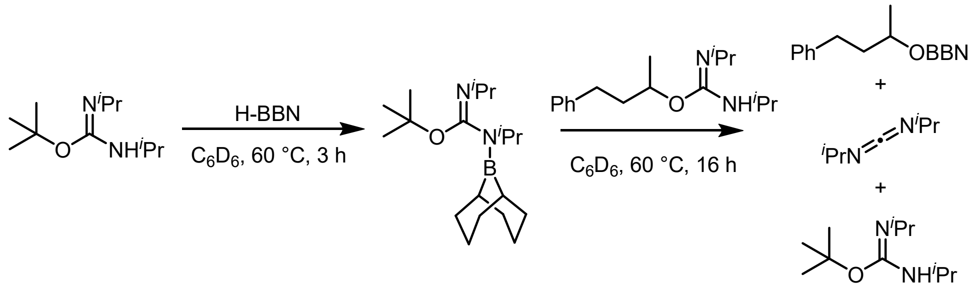

A solution of *O*-(*tert*-butyl)-*N*,*N'*-di*iso*propylisourea (20.0 mg, 0.100 mmol) and H-BBN (12.0 mg, 0.100 mmol) in C_6D_6 (0.600 mL) was heated at 60 °C for 3 hours then analysed by NMR spectroscopy. This showed complete conversion to the *O*-(*tert*-butyl)-*N*-(*B*-9-borabicyclo[3.3.1]nonyl)-*N*,*N'*-di*iso*propylisourea.

^{1}H NMR (500 MHz, C_6D_6) δ 3.68 (sept., *J* = 6.8 Hz, 1H), 3.55 (sept., *J* = 6.1 Hz, 1H), 2.00 – 1.76 (m, 10H), 1.63 – 1.58 (m, 2H), 1.51 (s, 9H), 1.46 – 1.37 (m, 2H), 1.27 (d, *J* = 6.7 Hz, 3H), 1.17 – 1.10 (m, 9H).

^{11}B NMR (160 MHz, C_6D_6) δ 49.9.

^{13}C NMR (126 MHz, C_6D_6) δ 149.3, 78.5, 49.4, 49.2, 34.6, 34.3, 32.6, 32.3, 28.2, 24.9, 24.7, 23.0, 22.4.

To this was added (±)-*O*-(2-(4-phenyl)butyl)-*N*,*N'*-di*iso*propylisourea (28.0 mg, 0.100 mmol) and the mixture was heated at 60 °C for 16 hours. The mixture then was analysed by NMR spectroscopy which showed the formation of (±)-*O*-(2-(4-phenyl)butoxy)-*B*-9-borabicyclo[3.3.1]nonane, DIC, and reformation of *O*-(*tert*-butyl)-*N*,*N'*-di*iso*propylisourea. Note, some degradation product, isobutylene, was also observed.[22]

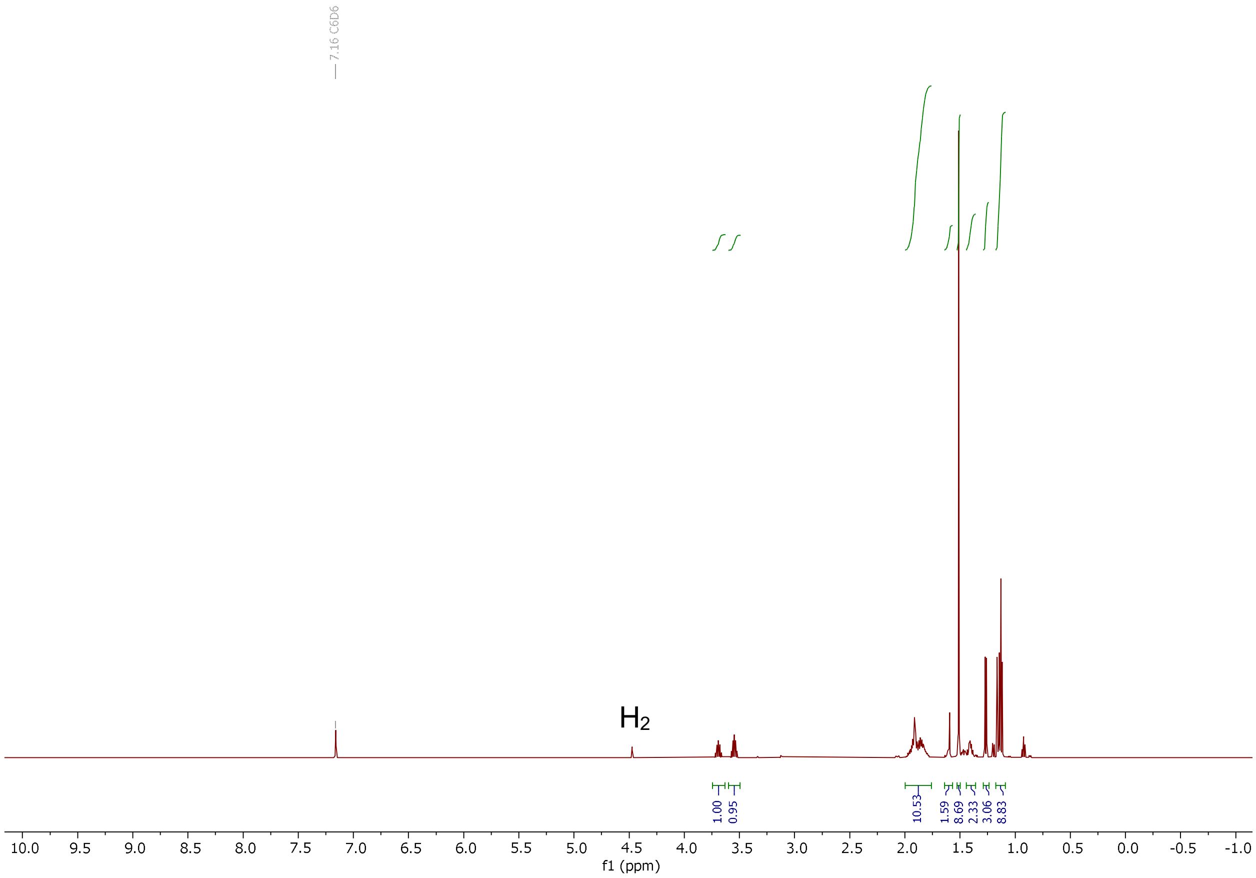

Figure S75. ^{1}H NMR (500 MHz, C_6D_6) spectrum of the reaction between *O*-(*tert*-butyl)-*N*,*N'*-di*iso*propylisourea and H-BBN.

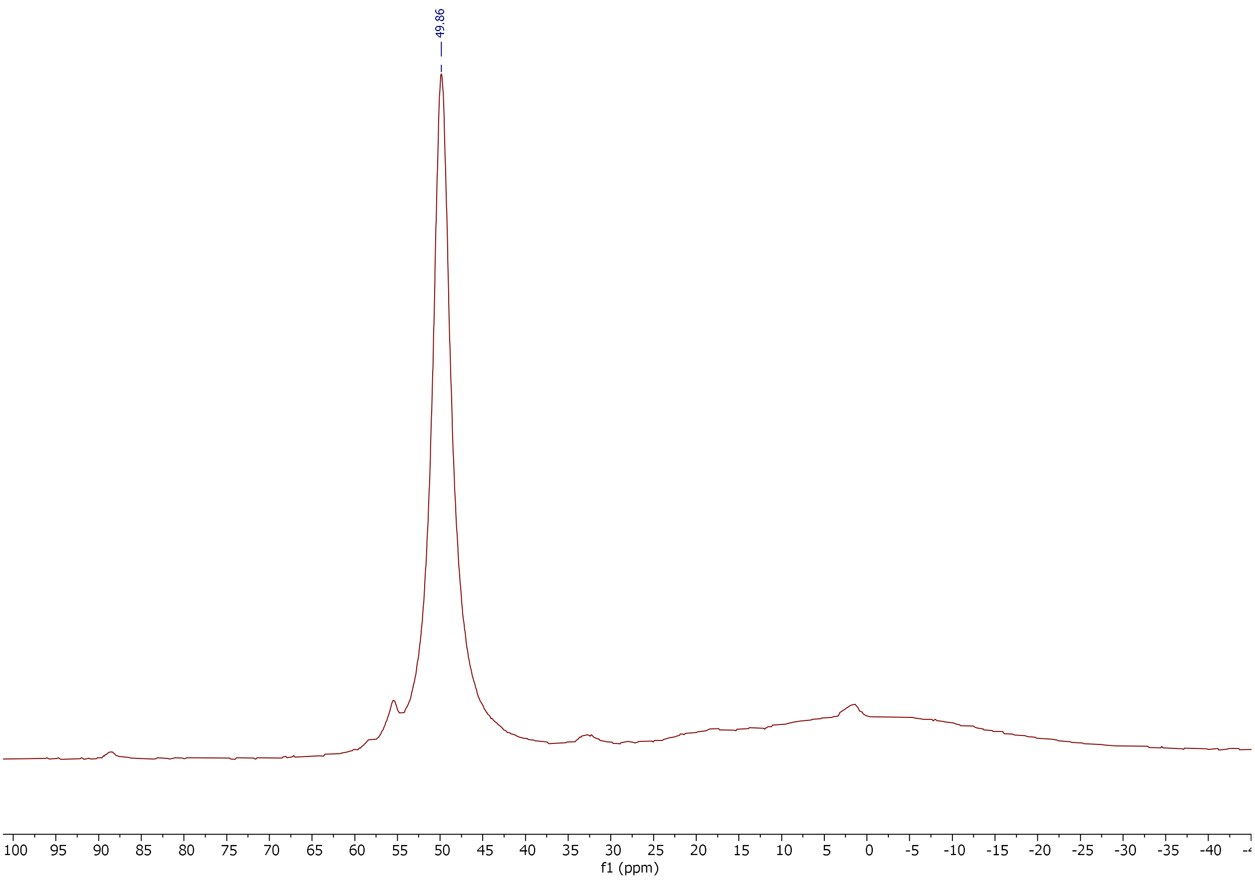

Figure S76. ^{11}B NMR (160 MHz, C_6D_6) spectrum of the reaction between *O*-(*tert*-butyl)-*N*,*N'*-di*iso*propylisourea and H-BBN.

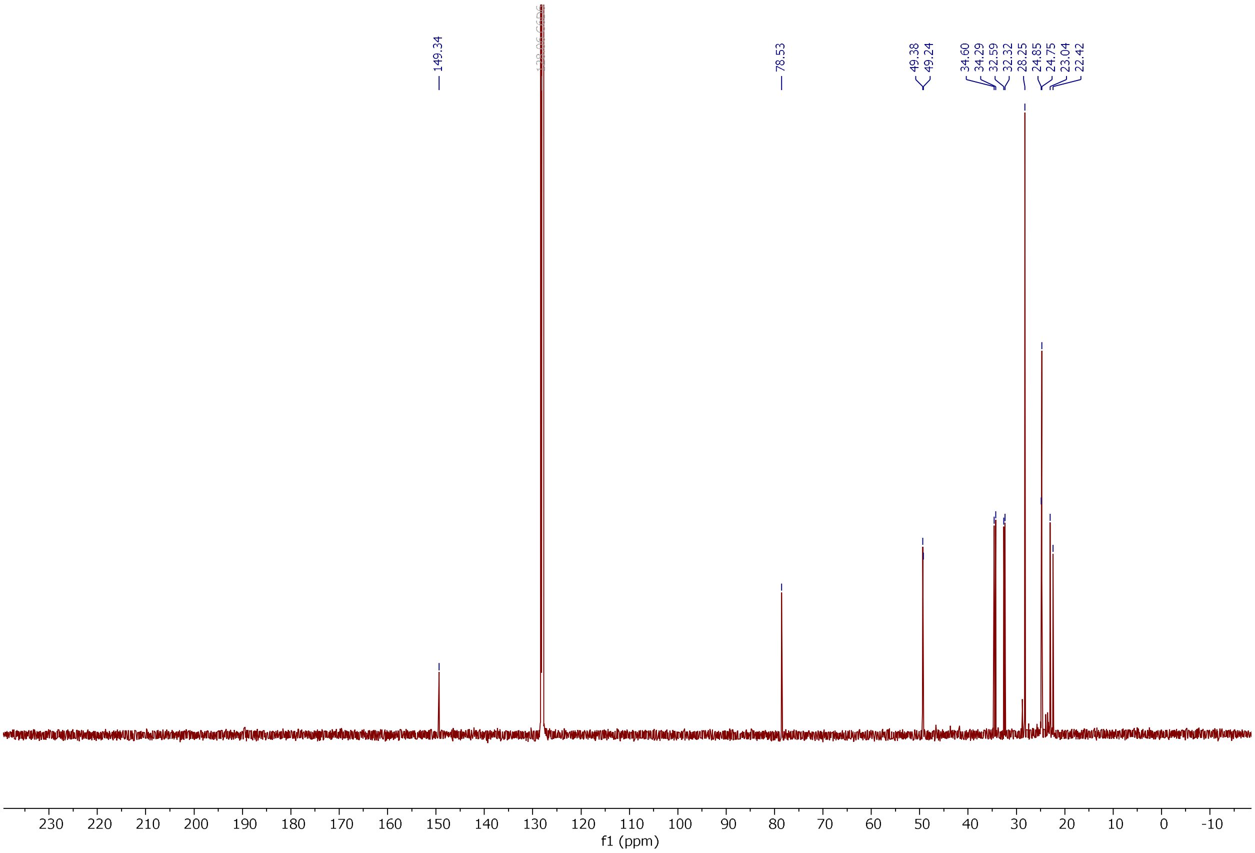

Figure S77. ^{13}C NMR (126 MHz, C_6D_6) spectrum of the reaction between *O*-(*tert*-butyl)-*N*,*N'*-di*iso*propylisourea and H-BBN.

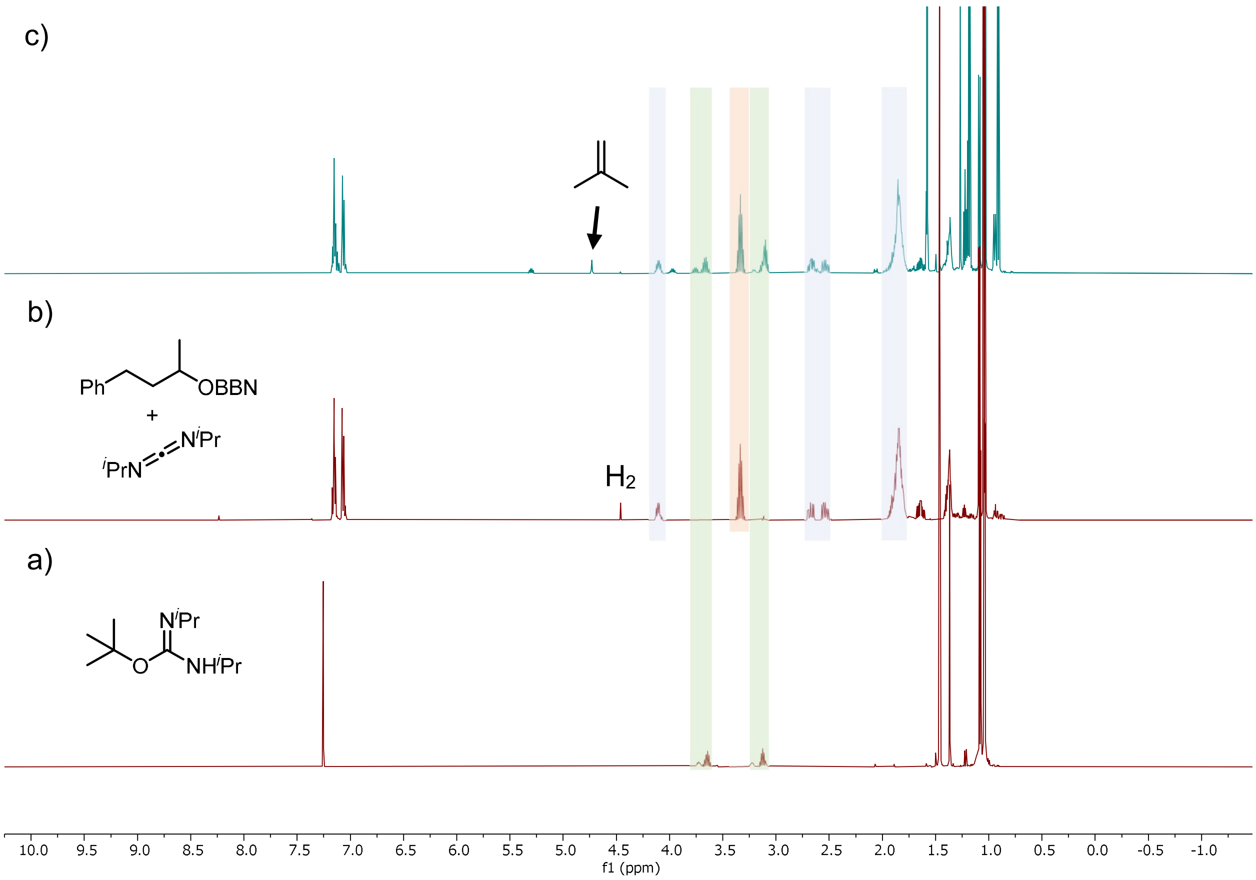

Figure S78. a) ^1H NMR (500 MHz, $CDCl_3$) spectrum of isolated *O*-(*tert*-butyl)-*N*,*N'*-di*iso*propylisourea for comparison. b) ^1H NMR (500 MHz, C_6D_6) spectrum of (±)-*O*-(2-(4-phenyl)butyl)-*N*,*N'*-di*iso*propylisourea and DIC for comparison. c) ^1H NMR (500 MHz, C_6D_6) spectrum after reaction of (±)-*O*-(2-(4-phenyl)butyl)-*N*,*N'*-di*iso*propylisourea. Blue = secondary borinic ester. Orange = DIC. Green = *O*-(*tert*-butyl)-*N*,*N'*-di*iso*propylisourea.

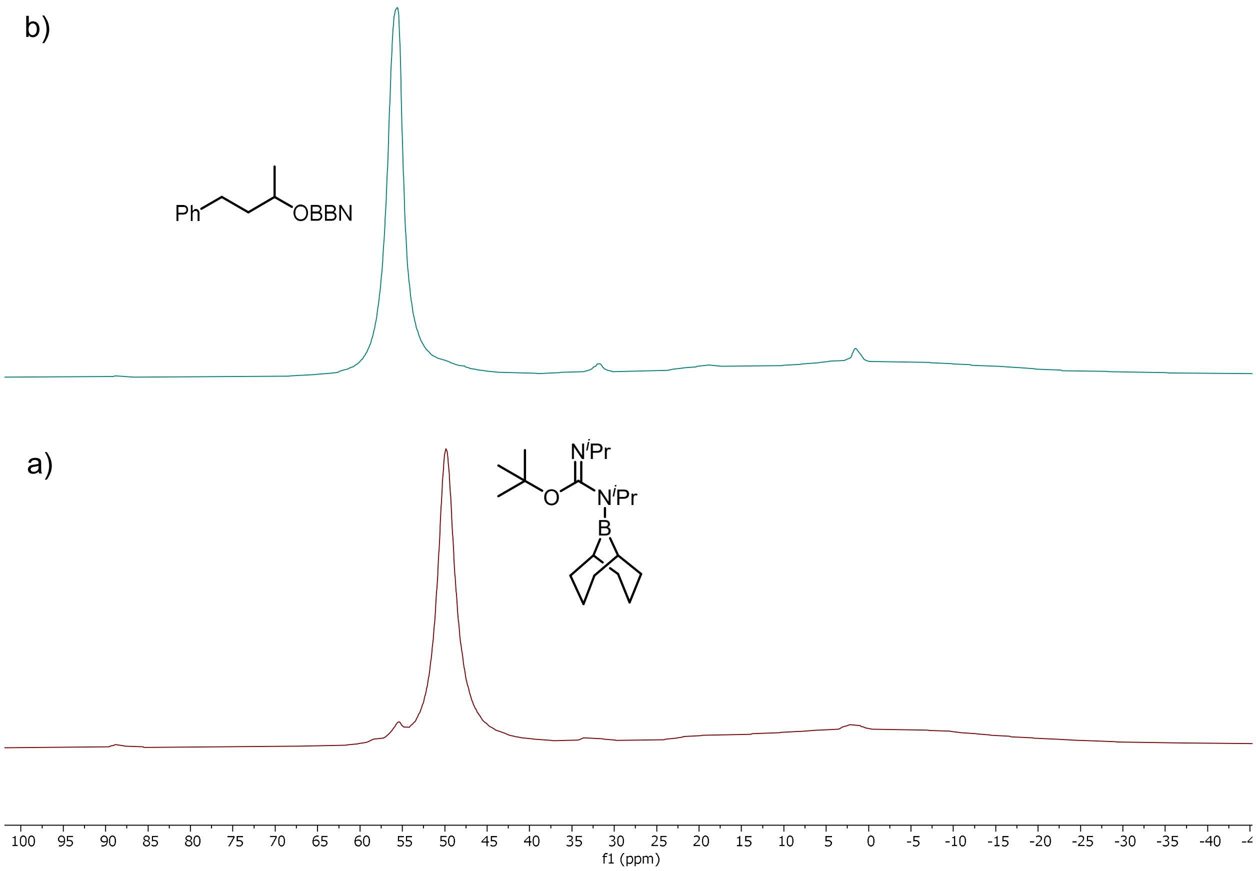

Figure S79. Overlaid ^{11}B NMR (160 MHz, C_6D_6) spectra of a) before addition of (±)-*O*-(2-(4-phenyl)butyl)-*N*,*N'*-di*iso*propylisourea b) after heating with (±)-*O*-(2-(4-phenyl)butyl)-*N*,*N'*-di*iso*propylisourea.

Investigation of BBN Role in Fluorination

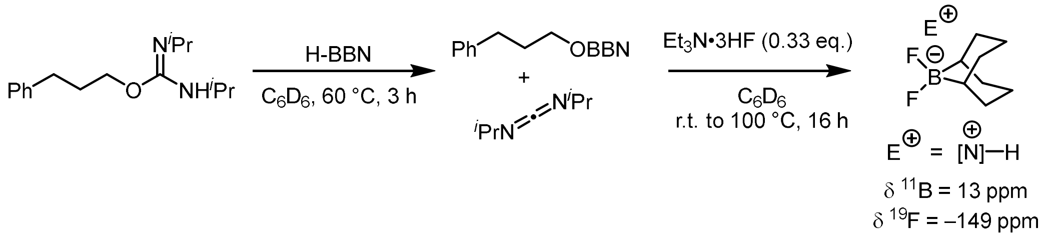

A solution of *O*-(1-(3-phenyl)propyl)-*N*,*N'*-di*iso*propylisourea (26.0 mg, 0.100 mmol) and H-BBN (12.0 mg, 0.100 mmol) in C_6D_6 (0.600 mL) was heated at 60 °C for 3 hours, then analysed by NMR spectroscopy, which showed conversion to the corresponding borinic ester (δ ^{11}B = 56 ppm) and DIC. Et_3N·3HF (6.0 μL, 33 μmol) then was added, the mixture was then analysed by NMR spectroscopy to show formation of a F_2BBN salt (δ ^{11}B = 13 ppm, δ ^{19}F = −149 ppm (br)) and complete consumption of Et_3N·3HF (lit: δ ^{19}F = −167 ppm[23]). The mixture then was heated at 100 °C for 16 hours and analysed by NMR spectroscopy, which gave trace 1-fluoro-3-phenylpropane (<1% by conversion), and minor degradation products, but the major species in both the ^{11}B and ^{19}F NMR spectra remained the F_2BBN salt. In all cases the ^{1}H NMR spectra were too broadened for any meaningful characterisation, thus the identity of $[E]^+$ is uncertain.

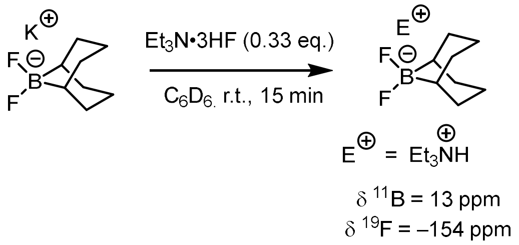

To a suspension of K[F_2BBN] (20.0 mg, 0.100 mmol) in C_6D_6 (0.600 mL) was added Et_3N·3HF (6.0 μL, 33 μmol), which led to a clear solution, and the mixture was then analysed by NMR spectroscopy to show a F_2BBN salt (δ ^{11}B = 13 ppm, δ ^{19}F = −154 ppm (br)), confirming the *in situ* characterised species to be a F_2BBN salt and complete consumption of Et_3N·3HF. The difference in the ^{19}F NMR shift is rationalised by a difference in the dominant cation, which in this case is tentatively assigned as $[Et_3NH]^+$.

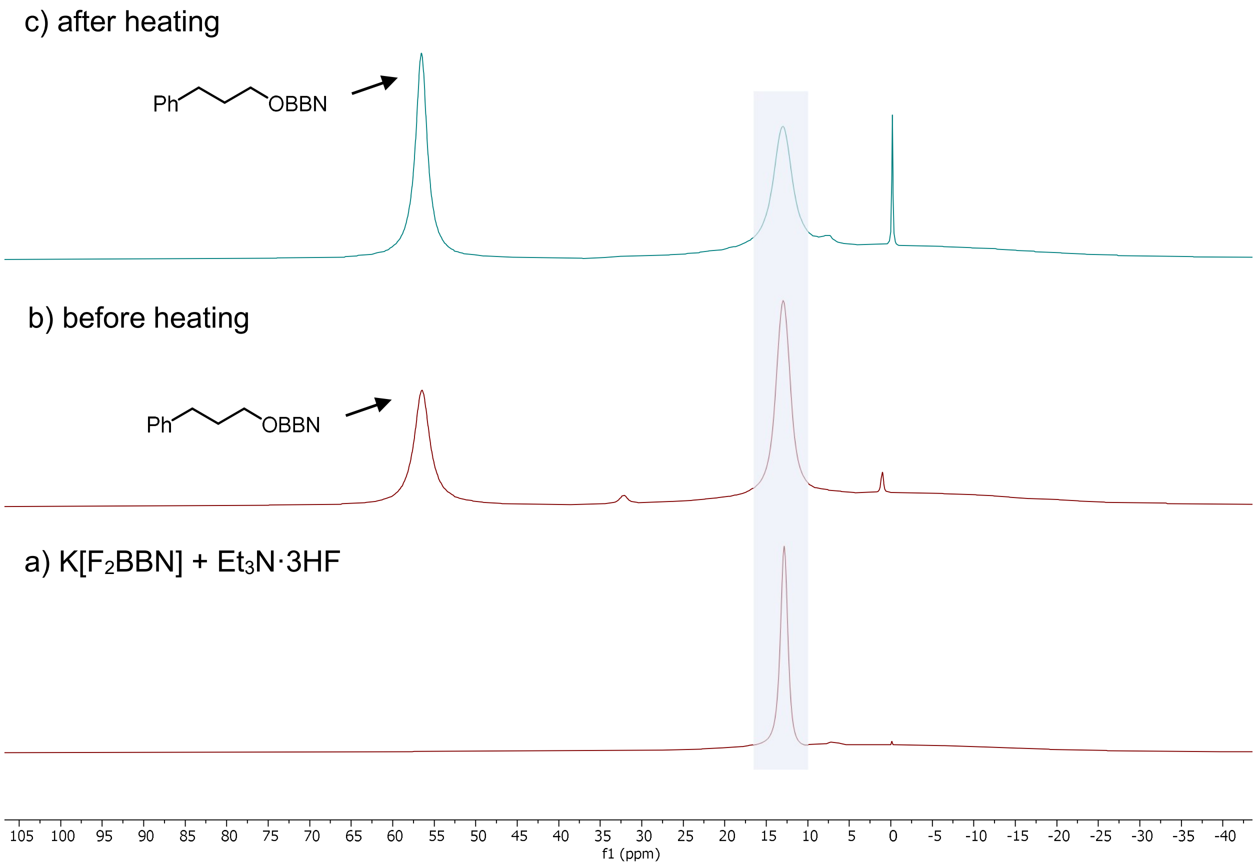

Figure S80. Overlaid ^{11}B NMR (160 MHz, C_6D_6) spectra of: a) K[F_2BBN] and $Et_3N{\cdot}3HF$, b) after addition of $Et_3N{\cdot}3HF$ to the borinic ester and DIC, c) after heating at 100 °C for 16 hours. Blue = F_2BBN species.

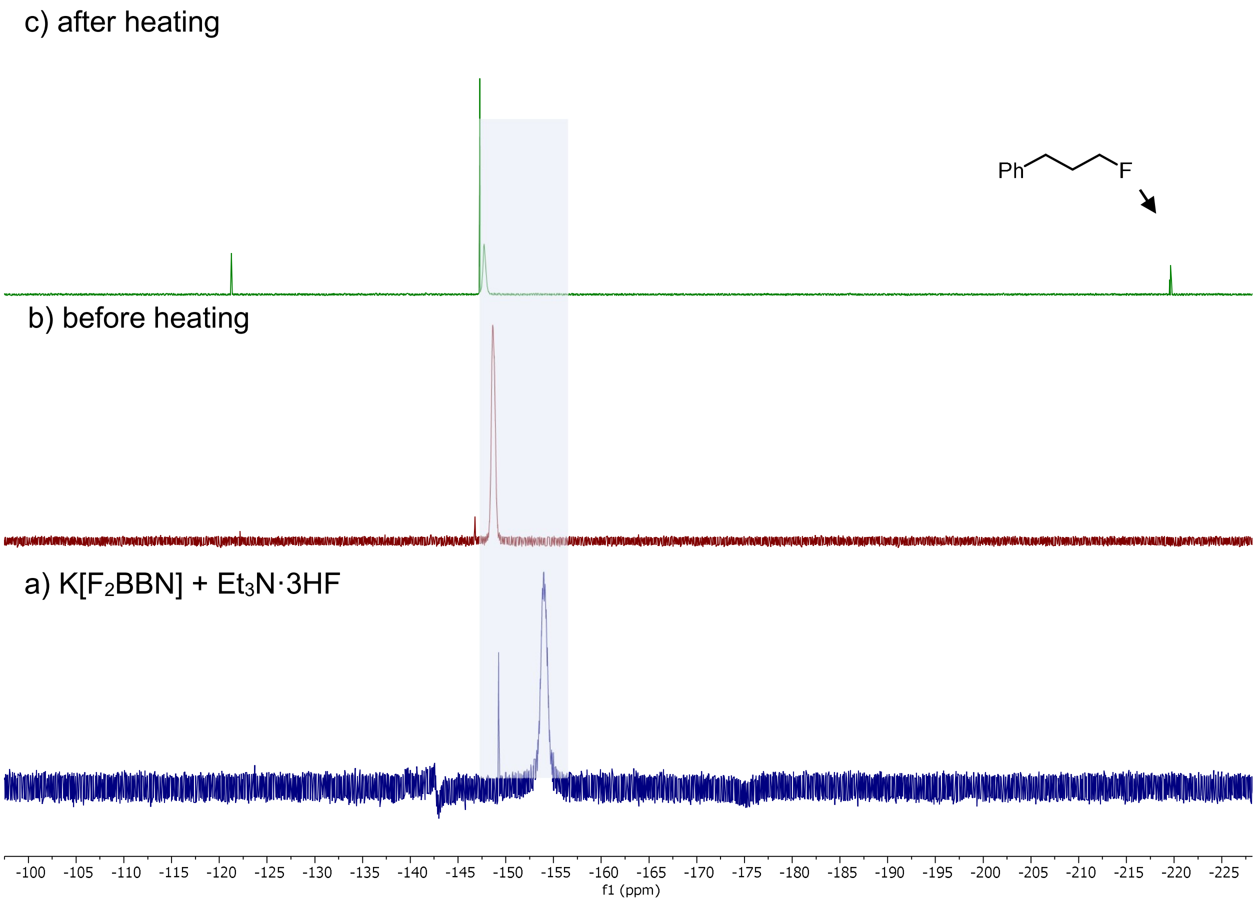

Figure S81. Overlaid ^{19}F NMR (160 MHz, C_6D_6) spectra of: a) K[F_2BBN] and Et_3N·3HF, b) after addition of Et_3N·3HF to the borinic ester and DIC, c) after heating at 100 °C for 16 hours. Blue = F_2BBN species region.

Using K[F_2BBN] as Fluoride Source

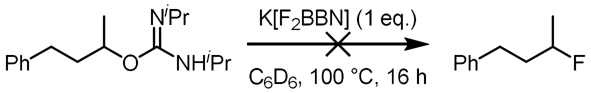

A suspension of (±)-*O*-(2-(4-phenyl)butyl)-*N*,*N'*-di*iso*propylisourea (28.0 mg, 0.100 mmol) and K[F_2BBN] (20.0 mg, 0.100 mmol) in C_6D_6 (0.600 mL) was heated at 100 °C for 16 hours, then analysed by NMR spectroscopy with hexafluorobenzene and 1,3,5-trifluorobenzene as internal standards, this revealed no fluorination.

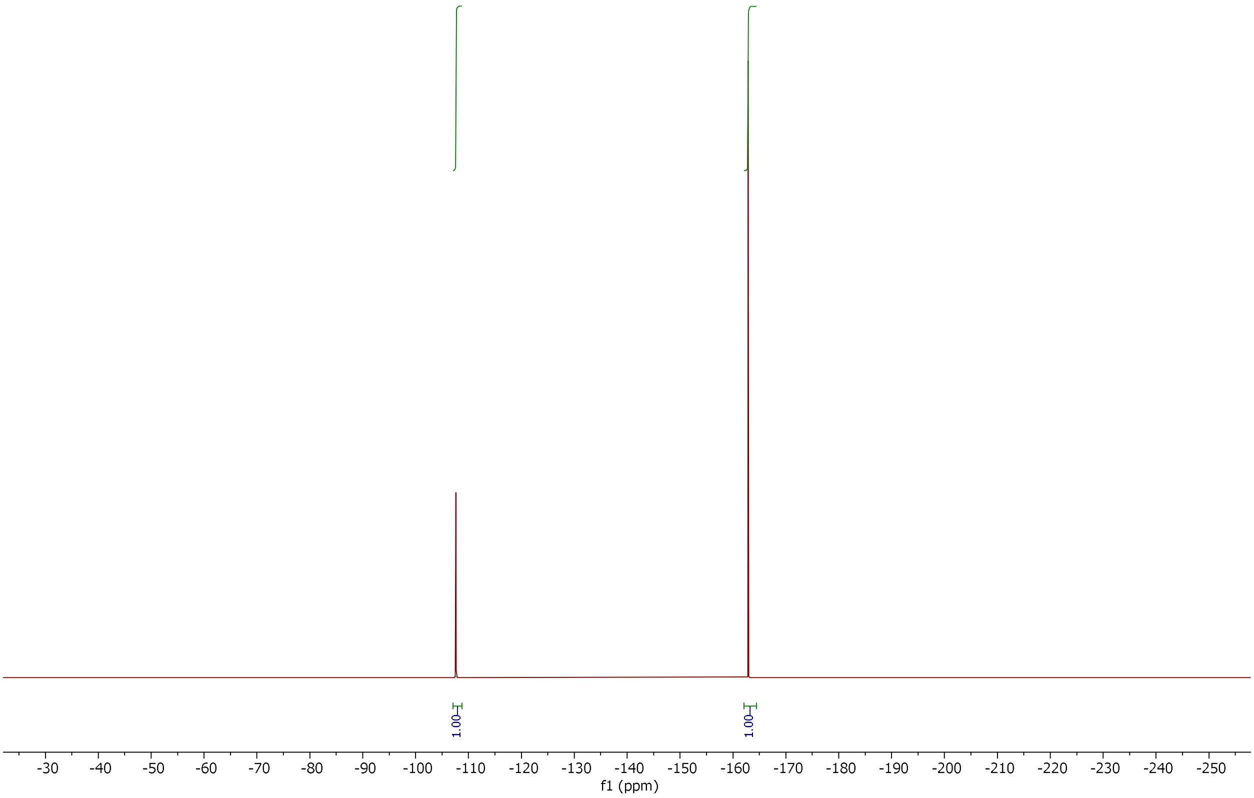

Figure S82. ^{19}F NMR (471 MHz, C_6D_6) spectrum of fluorination attempt with K[F_2BBN].

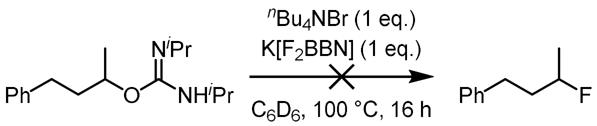

A suspension of (±)-*O*-(2-(4-phenyl)butyl)-*N*,*N'*-di*iso*propylisourea (28.0 mg, 0.100 mmol), K[F_2BBN] (20.0 mg, 0.100 mmol), and tetrabutylammonium bromide (32.0 mg, 0.100 mmol) in C_6D_6 (0.600 mL) was heated at 100 °C for 16 hours, then analysed by NMR spectroscopy with hexafluorobenzene and 1,3,5-trifluorobenzene as internal standards, this revealed no fluorination.

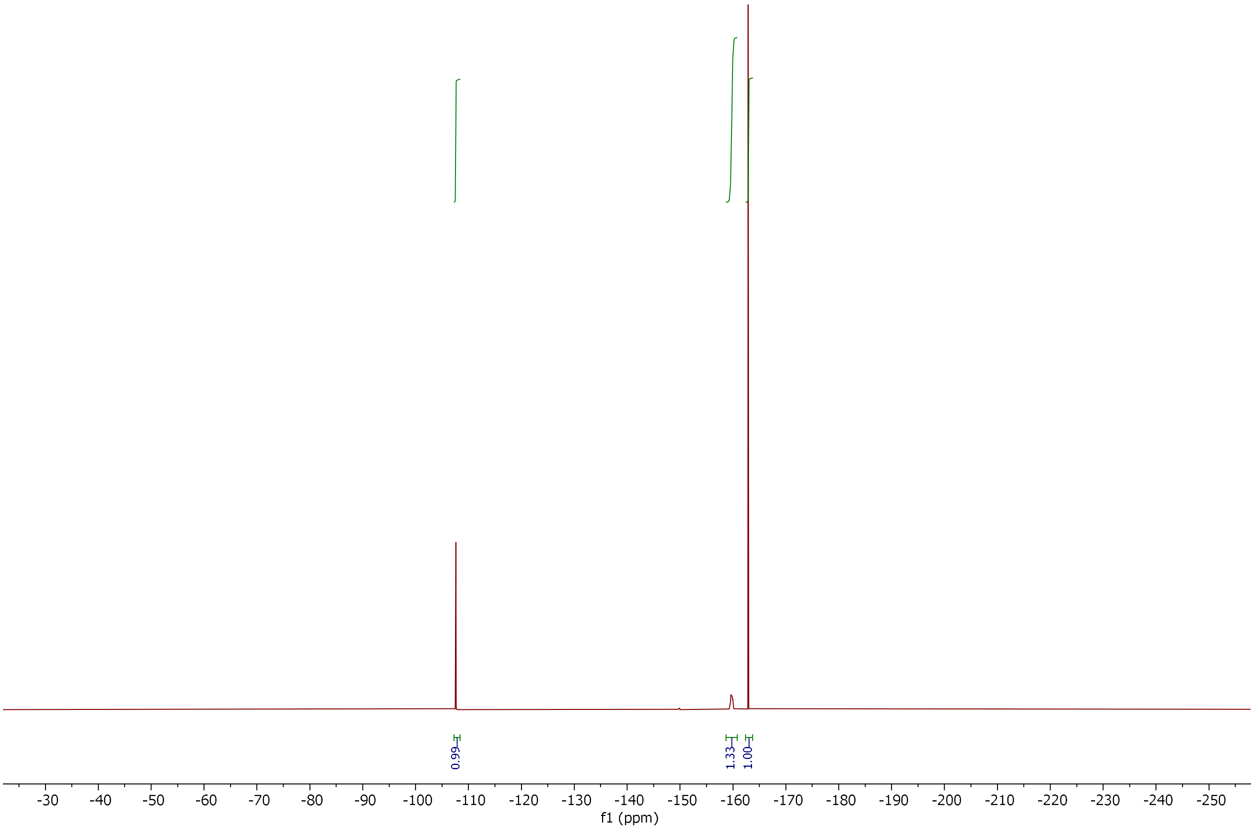

Figure S83. ^{19}F NMR (471 MHz, C_6D_6) spectrum of fluorination attempt with K[F_2BBN] and TBAB.

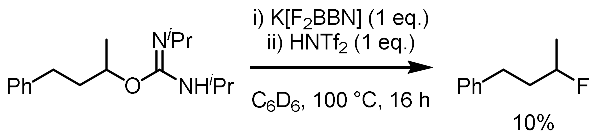

To a suspension of (±)-*O*-(2-(4-phenyl)butyl)-*N*,*N'*-di*iso*propylisourea (28.0 mg, 0.100 mmol) and K[F_2BBN] (20.0 mg, 0.100 mmol) in C_6D_6 (0.600 mL) was added $HNTf_2$ (28.0 mg, 0.100 mmol) and the mixture heated at 100 °C for 16 hours, then analysed by NMR spectroscopy with hexafluorobenzene and 1,3,5-trifluorobenzene as internal standards, which gave minimal 2-fluoro-4-phenylbutane (ca. 10%). No other significant species were observed by ^{19}F NMR spectroscopy other than $[NTf_2]^-$ (δ ^{19}F = −78.9 ppm).

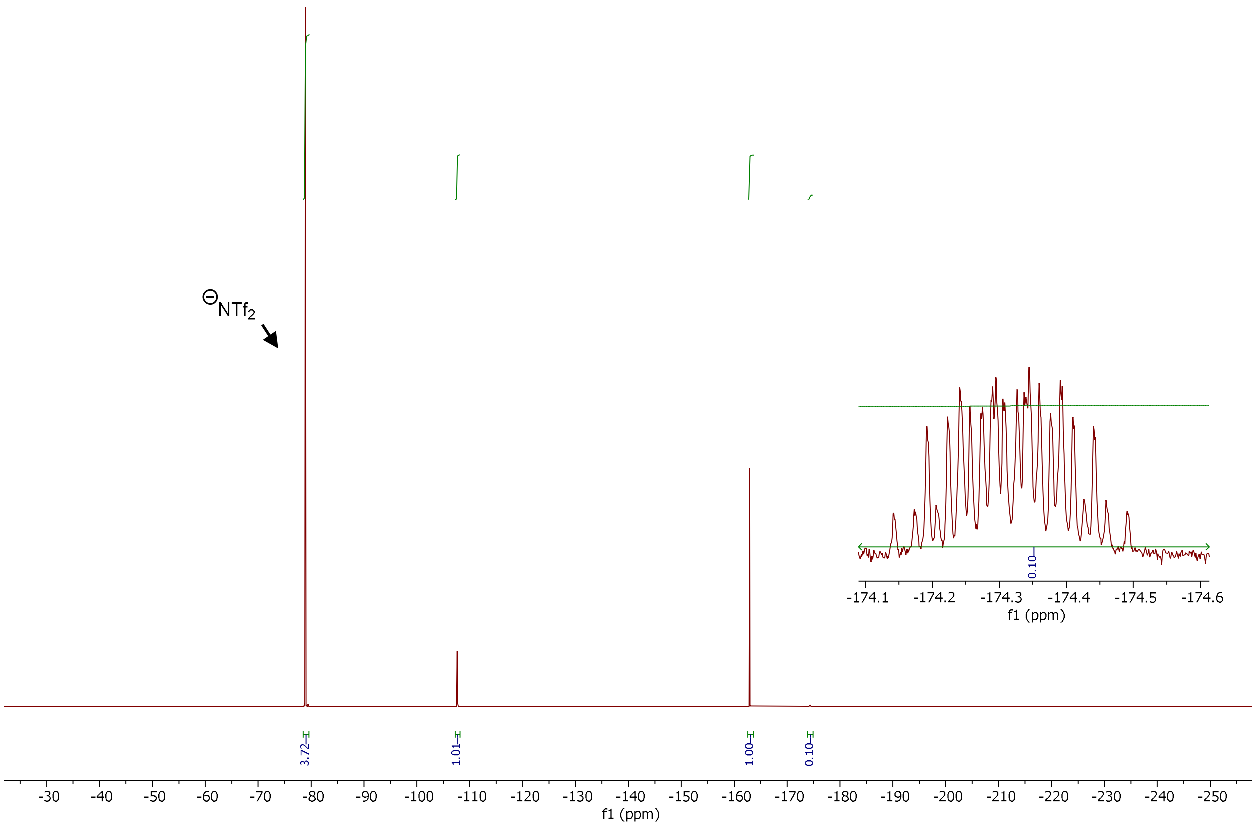

Figure S84. ^{19}F NMR (471 MHz, C_6D_6) spectrum of fluorination with K[F_2BBN] and $HNTf_2$.

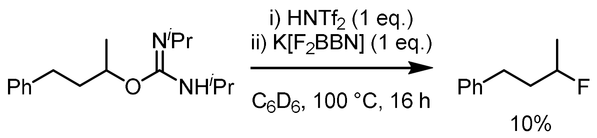

To a solution of (±)-*O*-(2-(4-phenyl)butyl)-*N*,*N'*-di*iso*propylisourea (28.0 mg, 0.100 mmol) and $HNTf_2$ (28.0 mg, 0.100 mmol) in C_6D_6 (0.600 mL) was added K[F_2BBN] (20.0 mg, 0.100 mmol) and the mixture heated at 100 °C for 16 hours, then analysed by NMR spectroscopy with hexafluorobenzene and 1,3,5-trifluorobenzene as internal standards, which gave minimal 2-fluoro-4-phenylbutane (ca. 10%). A number of uncharacterised B−F species were observed in the region of δ ^{19}F = −140 to −165 ppm, as well $[NTf_2]^-$ (δ ^{19}F = −78.9 ppm).

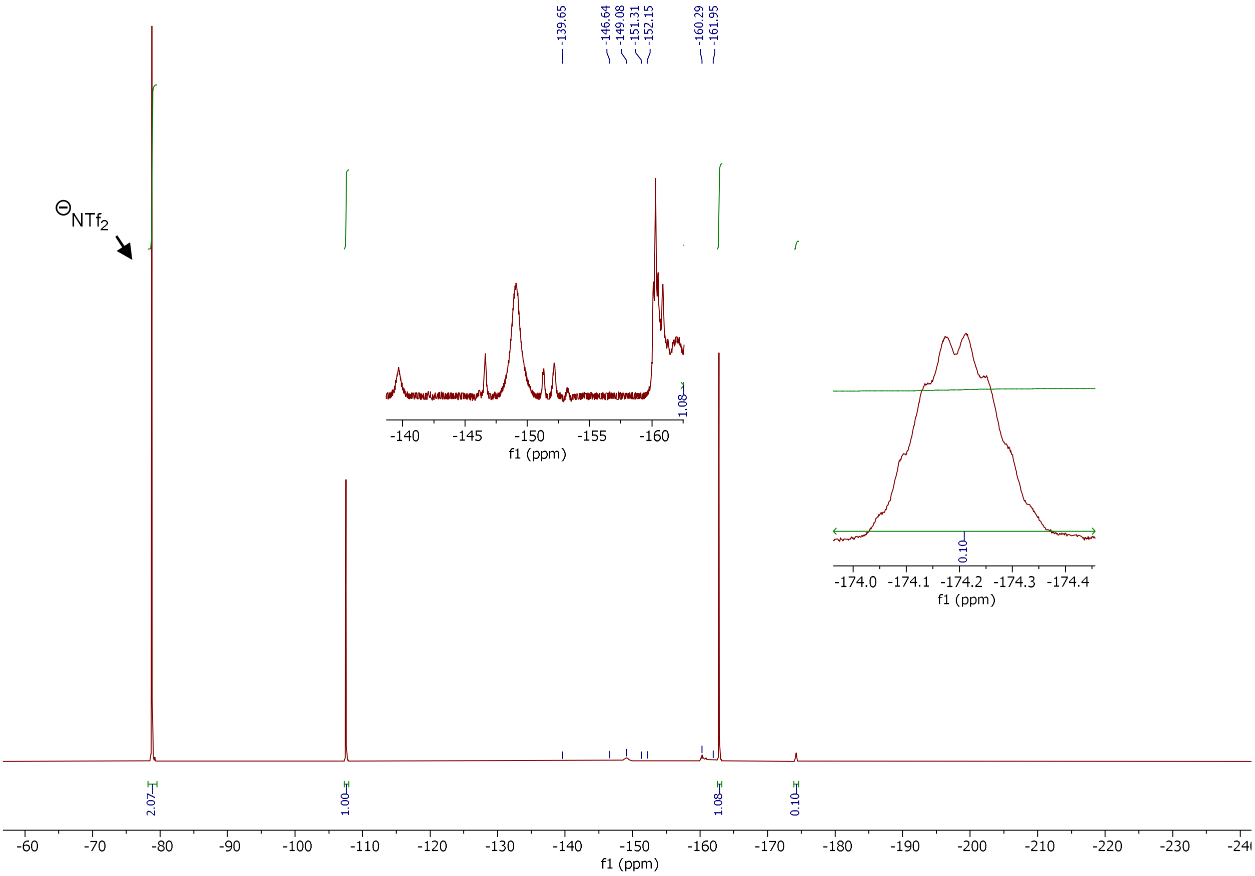

Figure S85. ^{19}F NMR (471 MHz, C_6D_6) spectrum of fluorination with K[F_2BBN] and $HNTf_2$.

Investigation of stepwise protonation then addition of Fluoride Sources

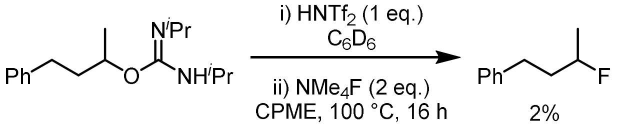

To a solution of (±)-*O*-(2-(4-phenyl)butyl)-*N*,*N'*-di*iso*propylisourea (28.0 mg, 0.100 mmol) and $HNTf_2$ (28.0 mg, 0.100 mmol) in C_6D_6 (0.200 mL) was added NMe_4F (19.0 mg, 0.200 mmol) in CPME (0.600 mL) and the mixture heated at 100 °C for 16 hours, then analysed by NMR spectroscopy with hexafluorobenzene as internal standard, which gave minimal 2-fluoro-4-phenylbutane (ca. 2%).

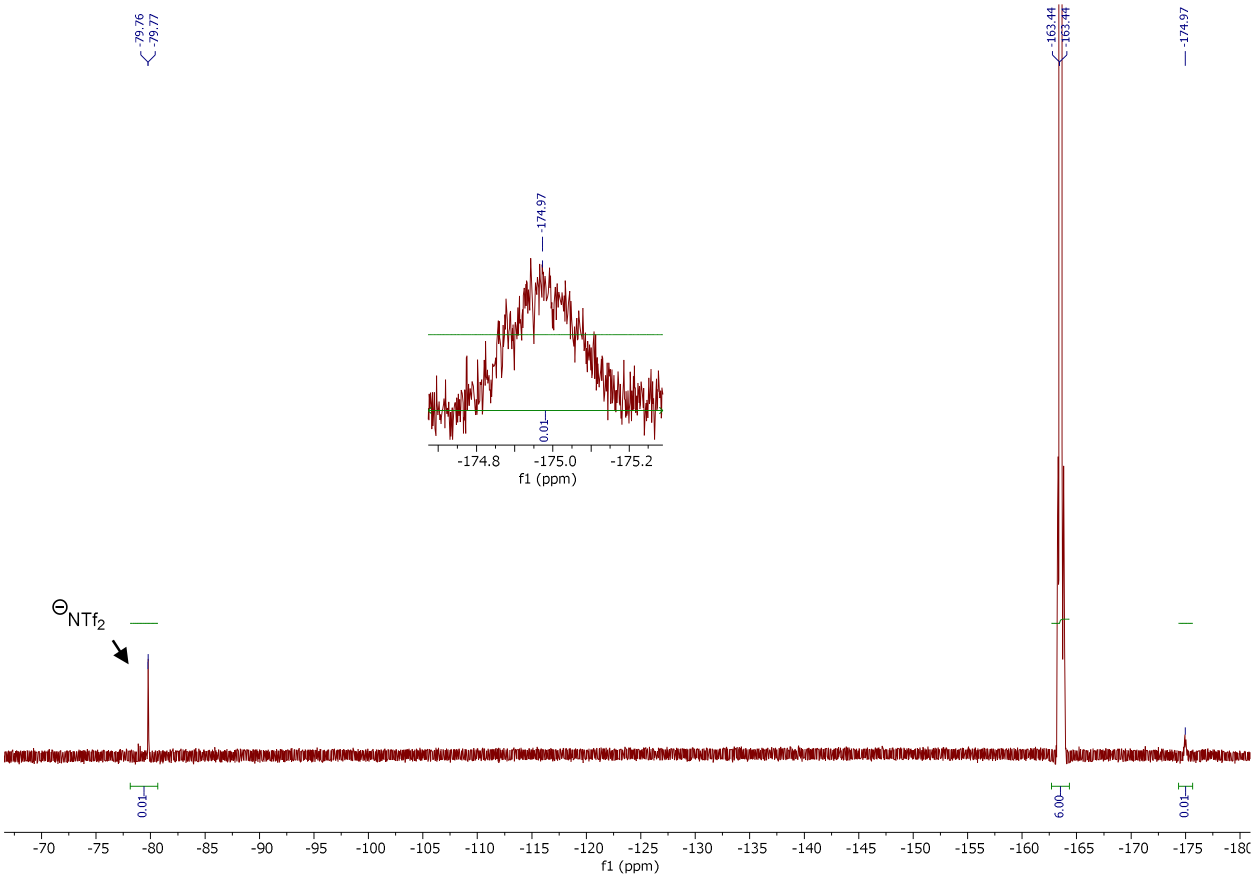

Figure S86. ^{19}F NMR (471 MHz, CPME/C_6D_6) spectrum of fluorination with NMe_4F and $HNTf_2$.

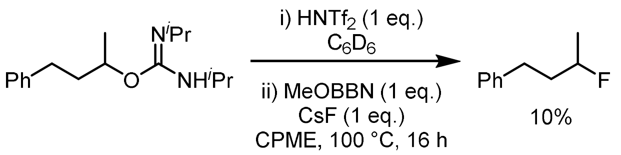

To a solution of (±)-*O*-(2-(4-phenyl)butyl)-*N*,*N'*-di*iso*propylisourea (28.0 mg, 0.100 mmol) and $HNTf_2$ (28.0 mg, 0.100 mmol) in C_6D_6 (0.200 mL) was added CsF (15.0 mg, 0.100 mmol) and MeOBBN (15.0 mg, 0.100 mmol) in CPME (0.600 mL) and the mixture heated at 100 °C for 16 hours, then analysed by NMR spectroscopy with hexafluorobenzene as internal standard, which gave minimal 2-fluoro-4-phenylbutane (ca. 10%).

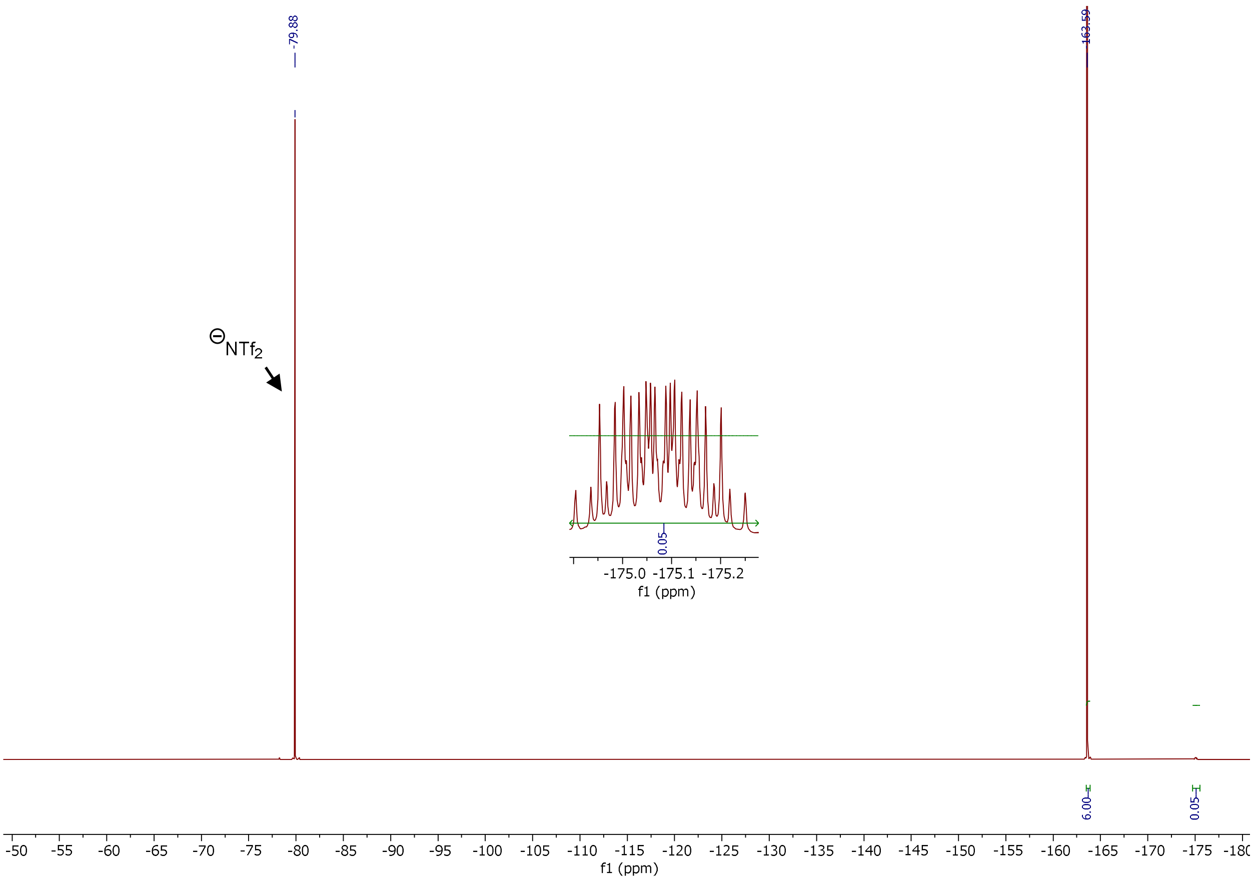

Figure S87. ^{19}F NMR (471 MHz, CPME/C_6D_6) spectrum of fluorination with CsF, MeOBBN and $HNTf_2$.

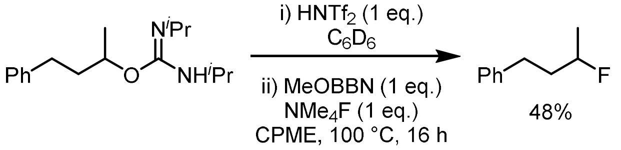

To a solution of (±)-*O*-(2-(4-phenyl)butyl)-*N*,*N'*-di*iso*propylisourea (28.0 mg, 0.100 mmol) and $HNTf_2$ (28.0 mg, 0.100 mmol) in C_6D_6 (0.200 mL) was added NMe_4F (9.0 mg, 0.100 mmol) and MeOBBN (15.0 mg, 0.100 mmol) in CPME (0.600 mL) and the mixture heated at 100 °C for 16 hours, then analysed by NMR spectroscopy with hexafluorobenzene as internal standard, which gave 2-fluoro-4-phenylbutane (ca. 48%).

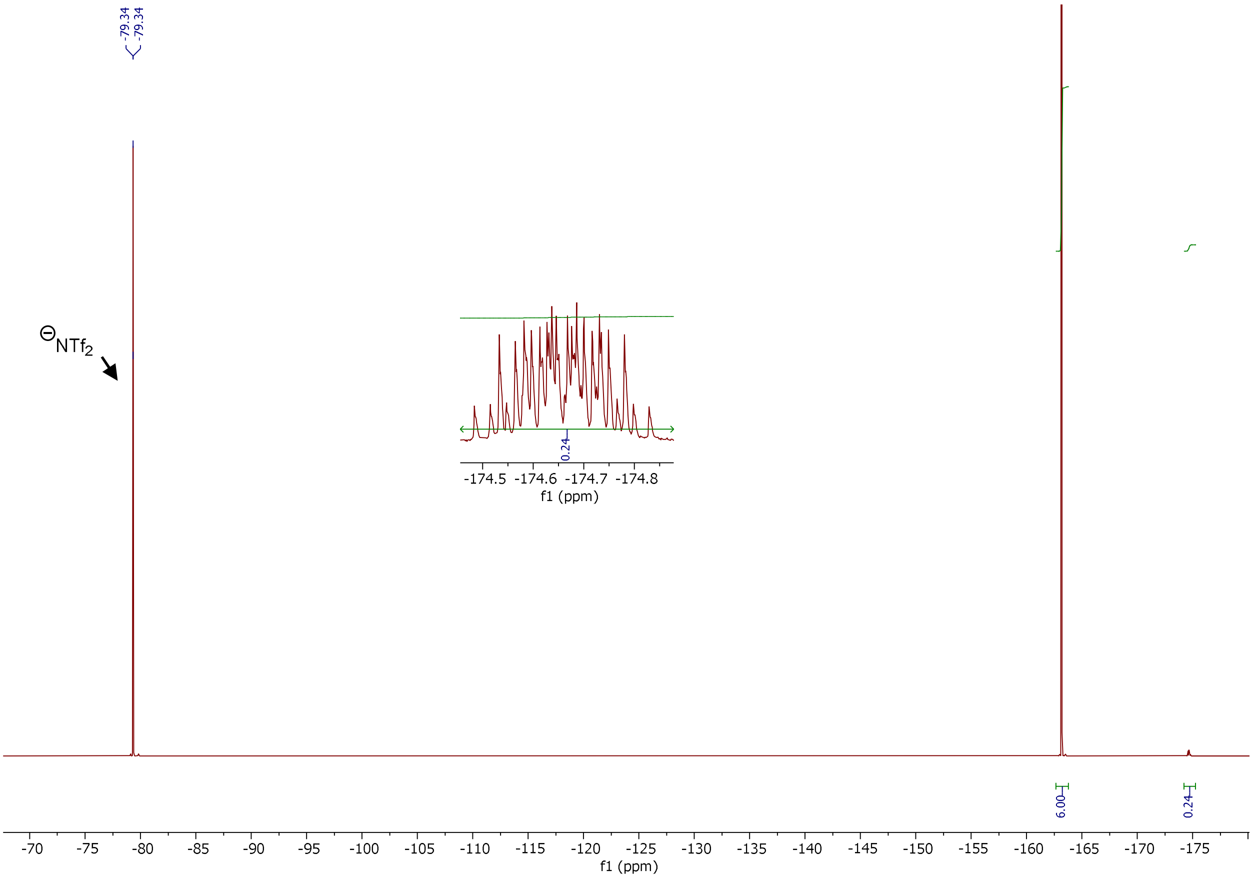

Figure S88. ^{19}F NMR (471 MHz, CPME/C_6D_6) spectrum of fluorination with NMe_4F, MeOBBN and $HNTf_2$.

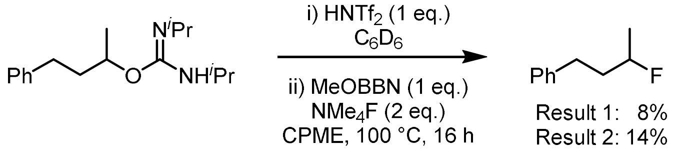

To a solution of (±)-*O*-(2-(4-phenyl)butyl)-*N*,*N'*-di*iso*propylisourea (28.0 mg, 0.100 mmol) and $HNTf_2$ (28.0 mg, 0.100 mmol) in C_6D_6 (0.200 mL) was added NMe_4F (18.0 mg, 0.200 mmol) and MeOBBN (15.0 mg, 0.100 mmol) in CPME (0.600 mL) and the mixture heated at 100 °C for 16 hours, then analysed by NMR spectroscopy with hexafluorobenzene as internal standard, which gave minimal 2-fluoro-4-phenylbutane (ca. 8%). F_2BBN species was also observed (δ ^{19}F = −155.0 ppm and ^{11}B = 10.6 ppm).

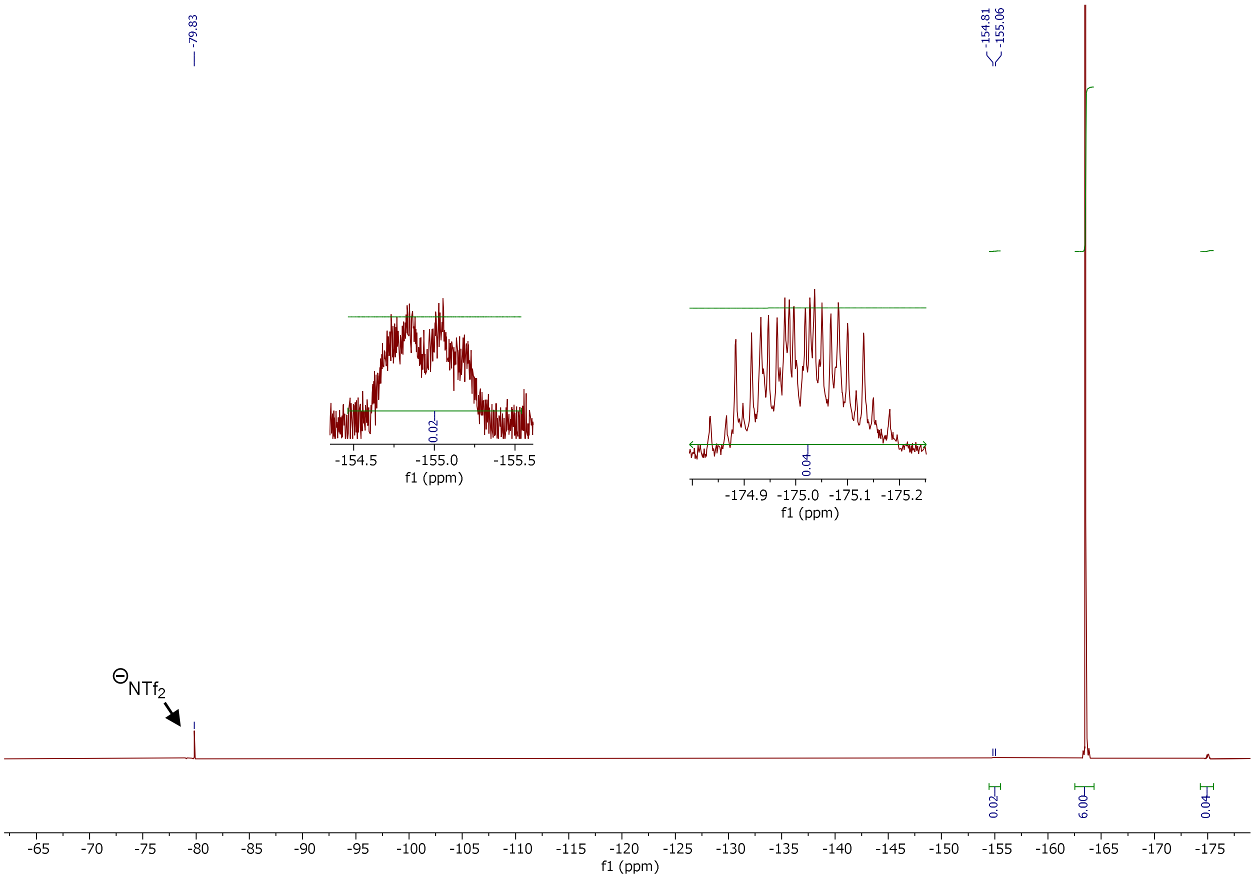

Figure S89. ^{19}F NMR (471 MHz, CPME/C_6D_6) spectrum of fluorination with NMe_4F, MeOBBN and $HNTf_2$.

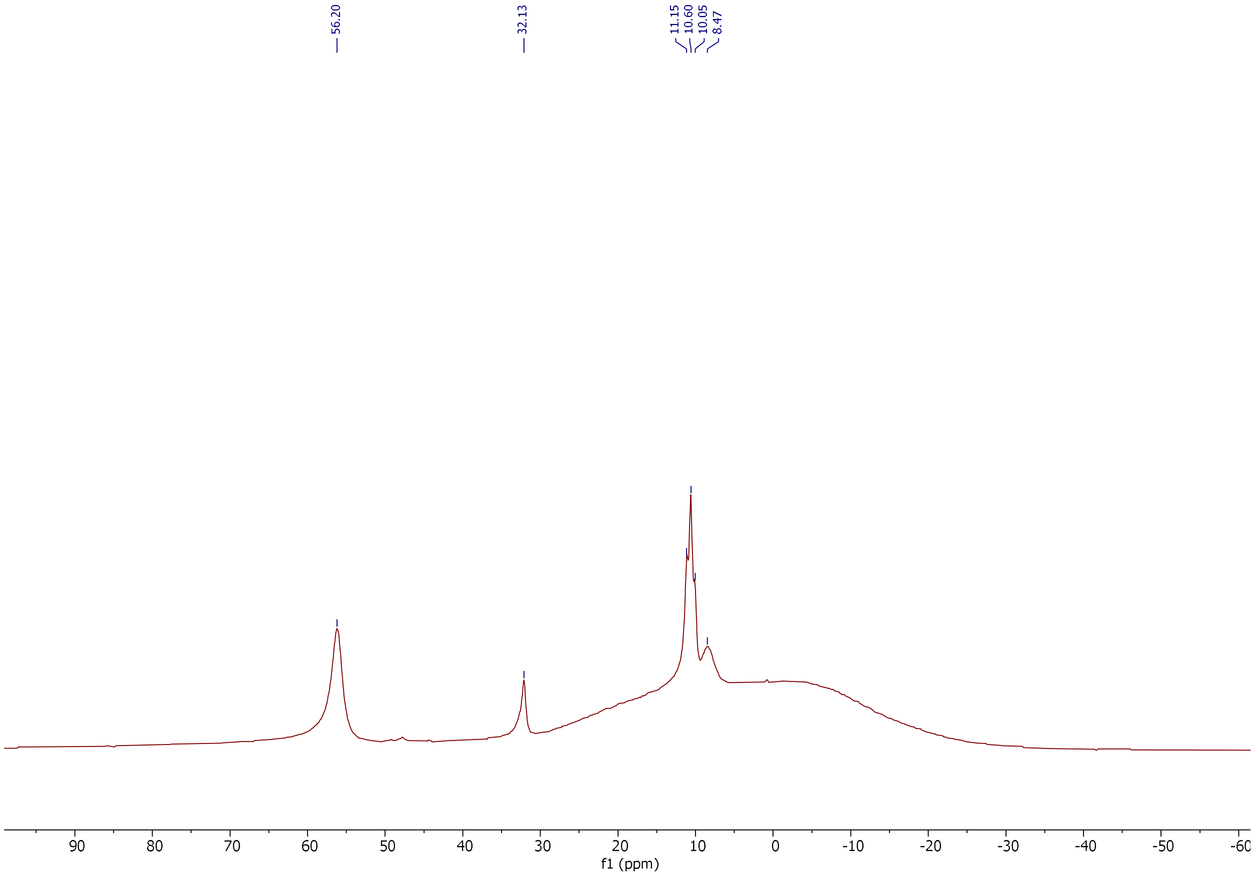

Figure S90. ^{11}B NMR (160 MHz, CPME/C_6D_6) spectrum of fluorination with NMe_4F, MeOBBN and $HNTf_2$.

Note, in this solvent mixture the $[F_2BBN]^-$ anion appears as a triplet in the ^{11}B NMR spectrum at +10.6 ppm, with a 1:1:1:1 quartet also observed in the ^{19}F NMR spectrum at -155 ppm. Again, the minor changes in chemical shift are attributed to the different cation. The minor species at 8.4 ppm in the ^{11}B NMR spectrum is tentatively assigned as the $[MeO(F)BBN]^-$ anion.

Table S16: Effect of exogenous borane additive.

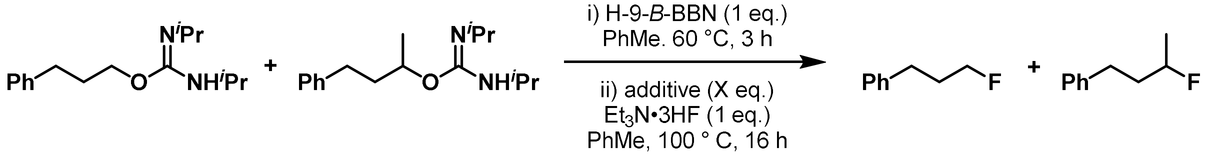

**Additive**	**X eq.**	**2° / %**	**1° / %**	**2°:1°**
none	-	53	2	27:1
MeO-9-*B*-BBN	0.5	70	1	70:1
	1.0	72	trace	-
	2.0	95	trace	-
K[F_2BBN]	0.5	64	3	21:1
	1.0	63	4	16:1
	2.0	61	4	15:1

10. Deoxychlorination

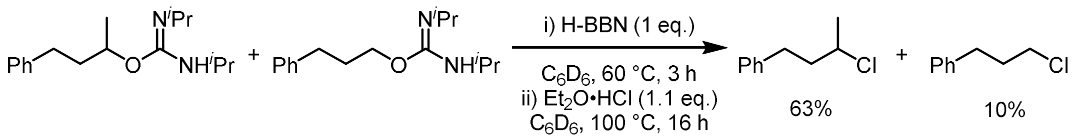

A solution of (±)-*O*-(2-(4-phenyl)butyl)-*N*,*N'*-di*iso*propylisourea (28.0 mg, 0.100 mmol), *O*-(1-(3-phenyl)propyl)-*N*,*N'*-di*iso*propylisourea (26.0 mg, 0.100 mmol) and H-BBN (12.0 mg, 0.100 mmol) in C_6D_6 (0.600 mL) was heated at 60 °C for 3 hours. $Et_2O{\cdot}HCl$ (55.0 µL, 2 M in Et_2O, 0.110 mmol) was added and the mixture heated at 100 °C for 16 hours in a sealed vessel. After cooling to room temperature, the mixture was diluted with diethyl ether (10 mL) and water (10 mL), extracted with diethyl ether (3 × 10 mL), dried (stirrer over Na_2SO_4), filtered, and concentrated *in vacuo*. The mixture was analysed by ^1H NMR spectroscopy with 1,3,5-trimethoxybenzene (10.0 µmol) as an internal standard which revealed 2-chloro-4-phenylbutane (63%, 2.86 (ddd, *J* = 14.2, 8.5, 5.9 Hz, 1H)) and 1-chloro-3-phenylpropane (10%, δ ^1H = 3.53 (t, *J* = 6.5 Hz, 2H), note: overlapping signals subtracted), determined from comparison to literature characterisation.[24]

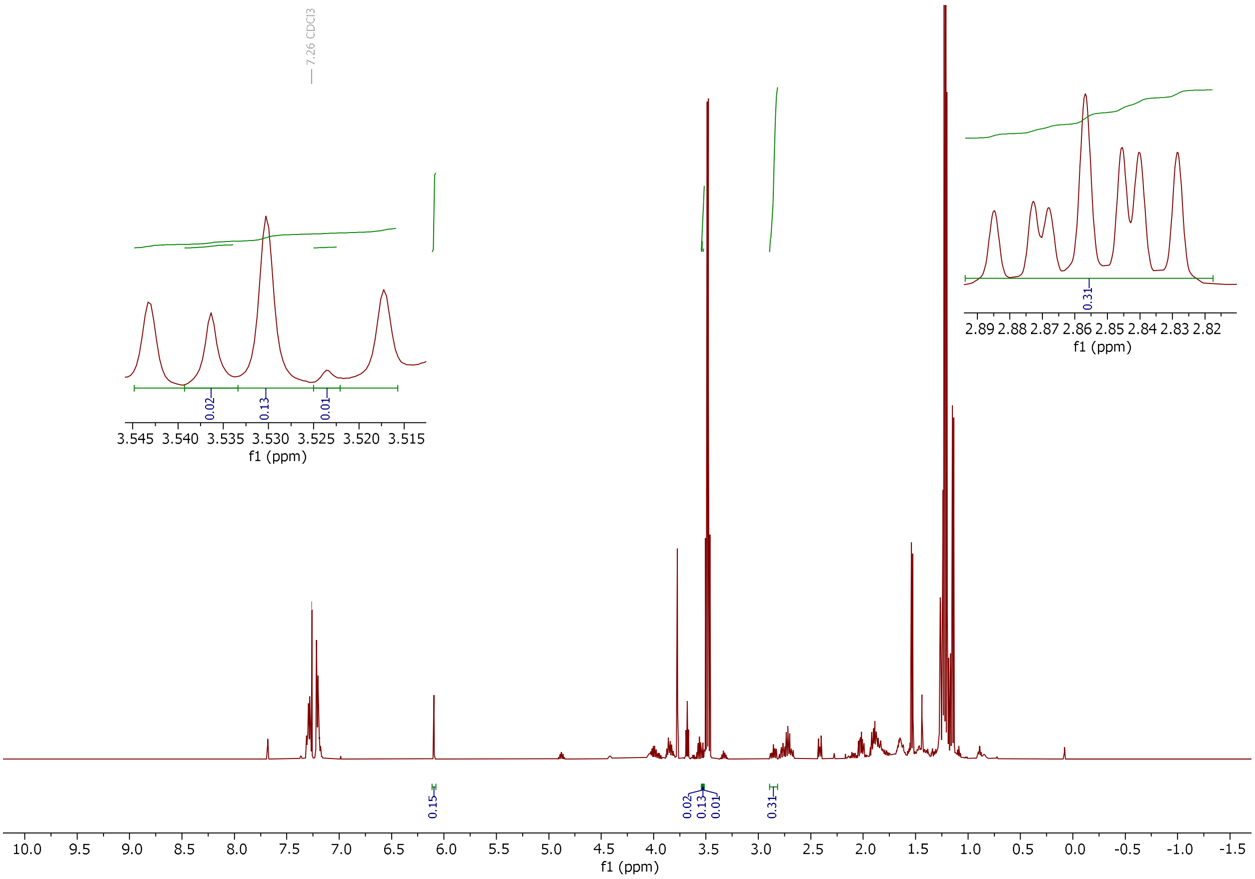

Figure S91. ^1H NMR (500 MHz, $CDCl_3$) spectrum of 2° vs 1° deoxychlorination with H-BBN.

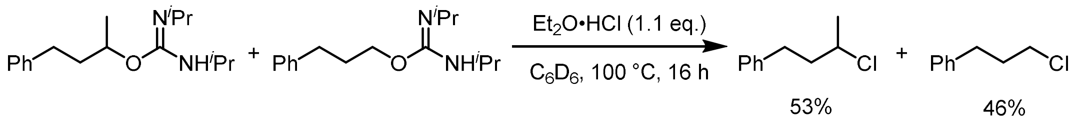

A solution of (±)-*O*-(2-(4-phenyl)butyl)-*N*,*N'*-di*iso*propylisourea (28.0 mg, 0.100 mmol), *O*-(1-(3-phenyl)propyl)-*N*,*N'*-di*iso*propylisourea (26.0 mg, 0.100 mmol) and $Et_2O{\cdot}HCl$ (55.0 µL, 2 M in Et_2O, 0.110 mmol) was heated at 100 °C for 16 hours in a sealed vessel. After cooling to room temperature, the mixture was diluted with diethyl ether (10 mL) and water (10 mL), extracted with diethyl ether (3 × 10 mL), dried (stirred over Na_2SO_4), filtered, and concentrated *in vacuo*. The mixture was analysed by ^{1}H NMR spectroscopy with 1,3,5-trimethoxybenzene (10.0 µmol) as an internal standard to give 2-chloro-4-phenylbutane (53%, 2.86 (ddd, *J* = 14.2, 8.5, 5.9 Hz, 1H)) and 1-chloro-3-phenylpropane (46%, δ ^{1}H = 3.53 (t, *J* = 6.5 Hz, 2H)), determined from comparison to literature characterisation.[24]

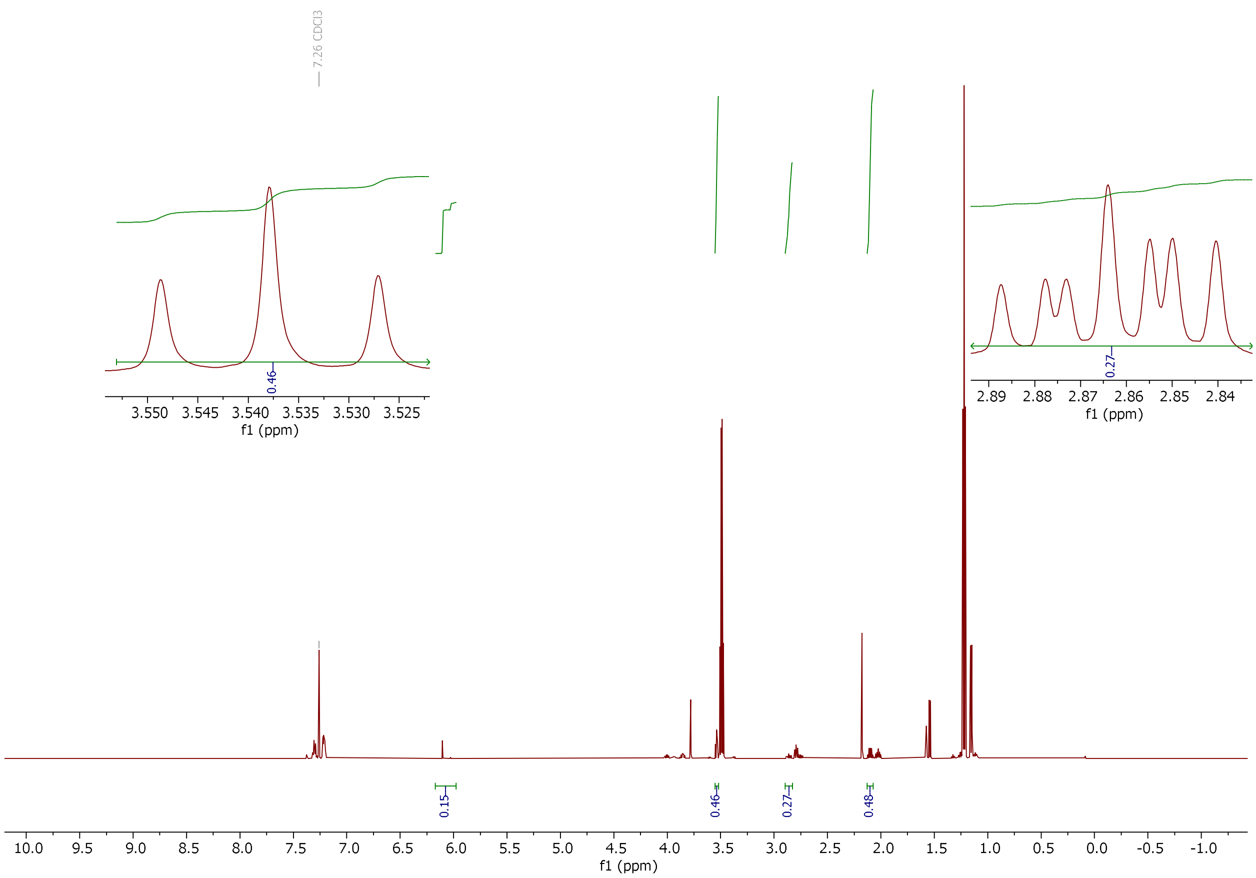

Figure S92. ^{1}H NMR (500 MHz, $CDCl_3$) spectrum of H-BBN-free 2° vs 1° deoxychlorination.

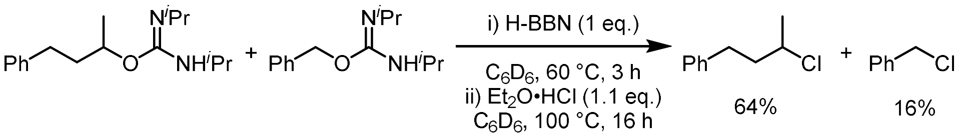

A solution of (±)-*O*-(2-(4-phenyl)butyl)-*N*,*N'*-di*iso*propylisourea (28.0 mg, 0.100 mmol), *O*-benzyl-*N*,*N'*-di*iso*propylisourea (24.0 mg, 0.100 mmol) and H-BBN (12.0 mg, 0.100 mmol) in C_6D_6 (0.600 mL) was heated at 60 °C for 3 hours. $Et_2O\cdot HCl$ (55.0 µL, 2 M in Et_2O, 0.110 mmol) was added and the mixture heated at 100 °C for 16 hours in a sealed vessel. After cooling to room temperature, the mixture was diluted with diethyl ether (10 mL) and water (10 mL), extracted with diethyl ether (3 × 10 mL), dried (stirred over Na_2SO_4), filtered, and concentrated *in vacuo*. The mixture was analysed by ^1H NMR spectroscopy with 1,3,5-trimethoxybenzene (10.0 µmol) as an internal standard to give 2-chloro-4-phenylbutane (64%, 2.86 (ddd, J = 14.2, 8.5, 5.9 Hz, 1H)) and benzyl chloride (16%, δ ^1H = 4.59 (s, 2H)), determined from comparison to literature characterisation.[24,25]

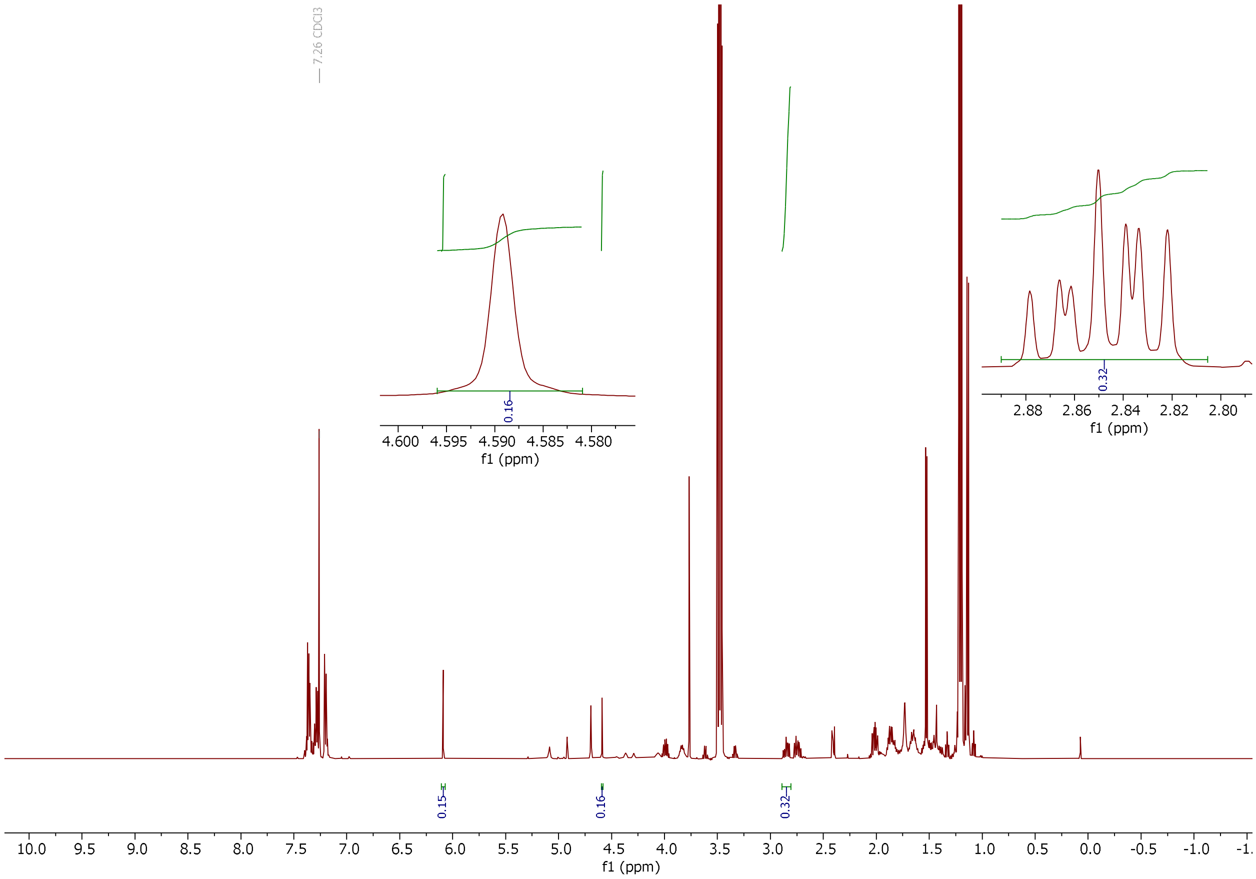

Figure S93. ^1H NMR (500 MHz, $CDCl_3$) spectrum of 2° vs Bn deoxychlorination with H-BBN.

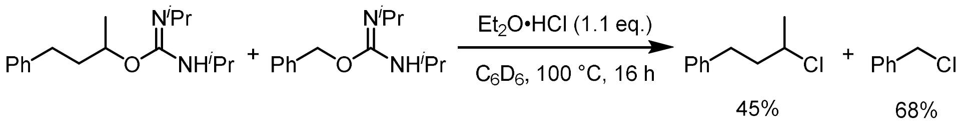

A solution of (±)-*O*-(2-(4-phenyl)butyl)-*N*,*N′*-di*iso*propylisourea (28.0 mg, 0.100 mmol), *O*-benzyl-*N*,*N′*-di*iso*propylisourea (24.0 mg, 0.100 mmol) and Et_2O·HCl (55.0 µL, 2 M in Et_2O, 0.110 mmol) was heated at 100 °C for 16 hours in a sealed vessel. After cooling to room temperature, the mixture was diluted with diethyl ether (10 mL) and water (10 mL), extracted with diethyl ether (3 × 10 mL), dried (stirred over Na_2SO_4), filtered, and concentrated *in vacuo*. The mixture was analysed by ^1H NMR spectroscopy with 1,3,5-trimethoxybenzene (10.0 µmol) as an internal standard to give 2-chloro-4-phenylbutane (45%, 2.87 (ddd, J = 14.2, 8.5, 5.9 Hz, 1H)) and benzyl chloride (68%, δ ^1H = 4.61 (s, 2H)), determined from comparison to literature characterisation.[24,25]

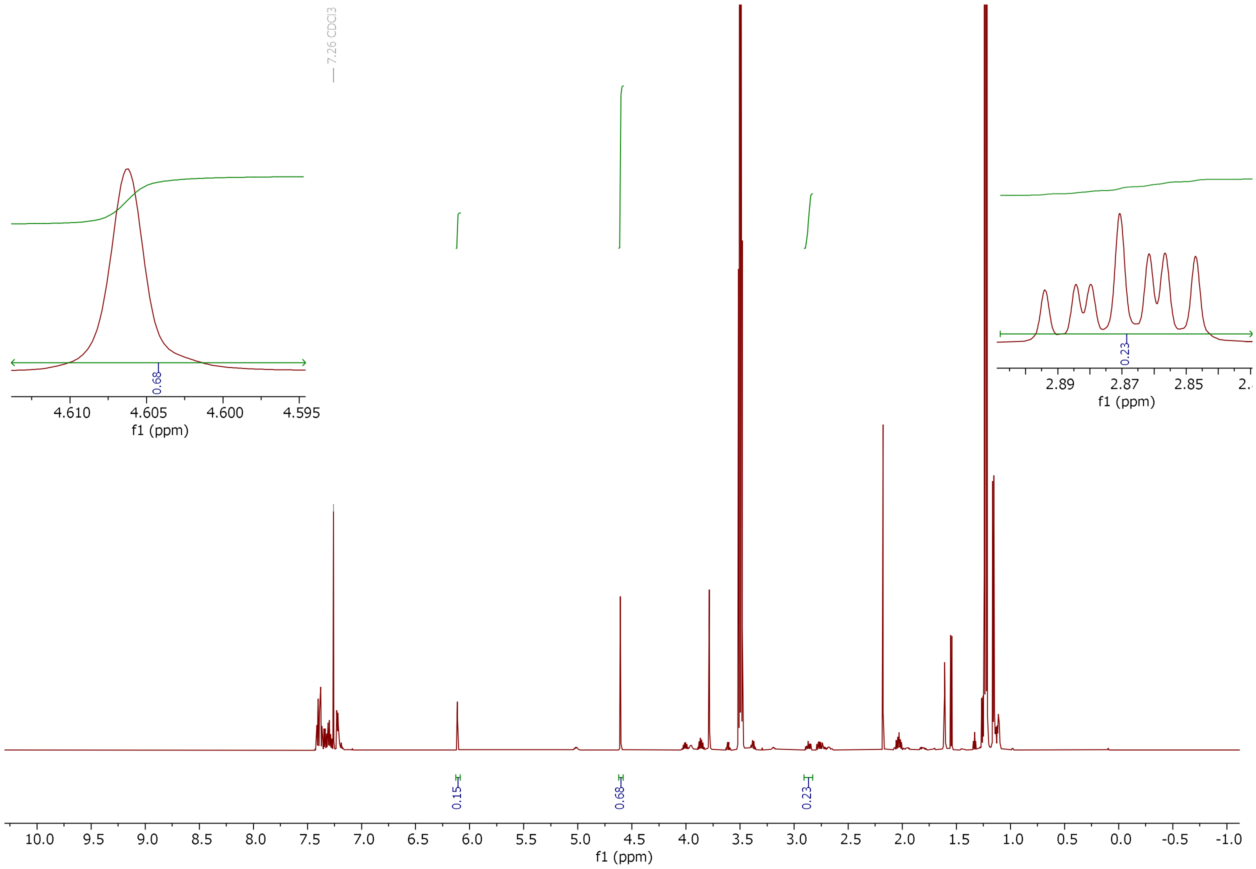

Figure S94. ^1H NMR (601 MHz, $CDCl_3$) spectrum of H-BBN-free 2° vs Bn deoxychlorination.

11. Synthesis of Alcohols

4-(4-(3-Hydroxypropoxy)phenyl)butan-2-ol 4a

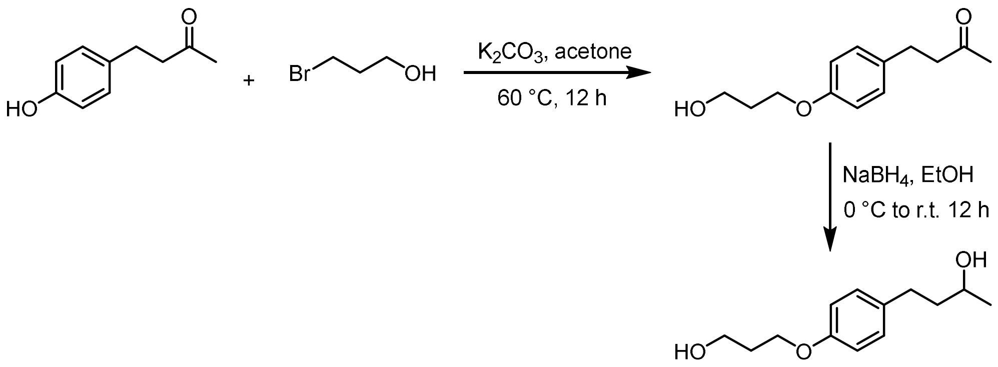

By the method of Hu,[26] 4-(4-hydroxyphenyl)butan-2-one (2.00 g, 12.0 mmol) was dissolved in acetone (24.0 mL) to which 3-bromopropan-1-ol (1.10 mL, 12.0 mmol) and K_2CO_3 (1.66 g, 12.0 mmol) were sequentially added. The reaction mixture was then stirred for 18 h at 60 °C forming a white suspension. After cooling the mixture, solids were filtered and the mother liquor diluted with water and the solution extracted with EtOAc (3 × 20 mL). The organic extracts were combined, dried with $MgSO_4$ and concentrated *in vacuo* to obtain a crude oil which was then purified by column chromatography on silica gel (EtOAc:40/60 petroleum ether = 50:50) to obtain 4-(4-(3-hydroxypropoxy)phenyl)butan-2-one as a colourless oil (1.14 g, 43%, 5.16 mmol).

Data were in accordance to those previously reported.[27]

4-(4-(3-hydroxypropoxy)phenyl)butan-2-one (1.14 g, 5.16 mol) was dissolved in EtOH (18.0 mL) and mixture was cooled to 0 °C to which $NaBH_4$ (195 mg, 5.10 mmol) was added. The reaction mixture was stirred at 0 °C for 30 min. and then warmed to room temperature and stirred for 16 h. Water was then added and the reaction mixture extracted with EtOAc (3 × 20 mL). The organic extracts were combined, dried with $MgSO_4$ and concentrated *in vacuo* to obtain crude off white solid which was then purified by column chromatography on silica gel (EtOAc:40/60 petroleum ether = 50:50) to obtain 4-(4-(3-hydroxypropoxy)phenyl)butan-2-ol as colourless block crystals (834 mg, 73%, 3.72 mmol).

^{1}H NMR (500 MHz, $CDCl_3$) δ 7.11 (d, J = 8.5 Hz, 2H), 6.83 (d, J = 8.6 Hz, 2H), 4.10 (t, J = 6.0 Hz, 2H), 3.87 – 3.78 (m, 3H), 2.72 – 2.58 (m, 2H), 2.04 (p, J = 6.0 Hz, 2H), 1.85 (bs, 1H), 1.79 – 1.68 (m, 2H), 1.37 (bs, 1H), 1.22 (d, J = 6.1 Hz, 2H).

^{13}C NMR (126 MHz, $CDCl_3$) δ 157.1, 134.5, 129.4, 114.6, 67.6, 66.0, 60.8, 41.1, 32.1, 31.3, 23.7.

Data were in accordance to those previously reported.[26]

5β-Cholane-3α,24-diol 4b

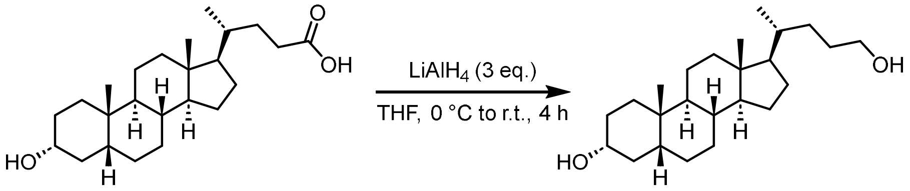

Lithium aluminium hydride (2.40 mL, 4.0 M in diethyl ether, 9.60 mmol) was added dropwise to a stirred solution of lithocholic acid (1.20 g, 3.20 mmol) in THF (27.0 mL) at 0 °C. The mixture was allowed to warm to room temperature and stirred for four hours. The mixture was cooled to 0 °C and saturated aqueous ammonium chloride (10 mL) was added dropwise, followed by aqueous HCl (10 mL, 1 M), and then the mixture was diluted with water (20 mL). The solution was extracted with ethyl acetate (3 × 20 mL), washed with brine (20 mL), dried (stirred over Na_2SO_4), filtered, and all volatiles removed *in vacuo*. The crude mixture was purified by flash column chromatography (CombiFlash Isco NextGen300+, 24 g SiO_2, 50 mm Ø, 40/60 petroleum ether/EtOAc 100:0 to 0:100) to give 5β-cholane-3α,24-diol as a colourless microcrystalline solid (686 mg, 59%, 1.89 mmol).

^{1}H NMR (500 MHz, $CDCl_3$) δ 3.67 – 3.56 (m, 3H), 1.99 – 1.94 (m, 1H), 1.90 – 1.71 (m, 3H), 1.69 – 0.78 (m, 31H, overlapping H_2O), 0.65 (s, 3H).

^{13}C NMR (151 MHz, $CDCl_3$) δ 72.0, 63.8, 56.7, 56.3, 42.9, 42.3, 40.6, 40.4, 36.6, 36.0, 35.7, 35.5, 34.7, 32.0, 30.7, 29.6, 28.5, 27.4, 26.6, 24.4, 23.5, 21.0, 18.8, 12.2.

Data were in accordance to those previously reported.[28]

(±)-*trans*-2-(4-(1-Hydroxypropan-2-yl)benzyl)cyclopentan-1-ol 4c

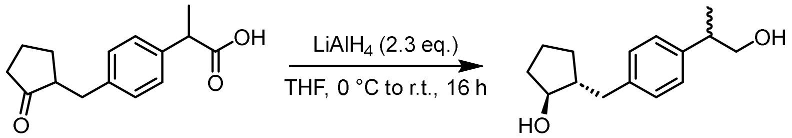

Lithium aluminium hydride (2.90 mL, 4.0 M in diethyl ether, 11.6 mmol) was added dropwise to a stirred solution of loxoprofen (1.28 g, 5.12 mmol) in THF (40.0 mL) at 0 °C. The mixture was allowed to warm to room temperature and stirred for 16 hours. The mixture was cooled to 0 °C and saturated aqueous ammonium chloride (10 mL) was added dropwise and the mixture then diluted with water (20 mL). The solution was extracted with ethyl acetate (3 × 30 mL), washed with brine (20 mL), dried (stirred over Na_2SO_4), filtered, and all volatiles removed *in vacuo*. The crude mixture was purified by flash column chromatography (CombiFlash Isco NextGen300+, 24 g SiO_2, 50 mm Ø, CH_2Cl_2/MeOH 100:0 to 90:10) to give (±)-*trans*-2-(4-(1-hydroxypropan-2-yl)benzyl)cyclopentan-1-ol as a colourless oil (956 mg, 80%, 4.08 mmol, d.r. = 4:1).

^{1}H NMR (500 MHz, $CDCl_3$) δ 7.22 – 7.12 (m, 4H), 4.09 (dq, *J* = 4.4, 2.6 Hz, 0.2H), 3.91 (qd, *J* = 5.7, 3.4 Hz, 0.8H), 3.69 (t, *J* = 6.4 Hz, 2H), 2.93 (hept., *J* = 6.9 Hz, 1H), 2.83 (dd, *J* = 13.7, 7.8 Hz, 0.2H), 2.74 (dd, *J* = 13.6, 6.8 Hz, 0.8H), 2.66 (dd, *J* = 13.7, 7.6 Hz, 0.2H), 2.52 (dd, *J* = 13.6, 8.4 Hz, 0.8H), 2.06 – 1.92 (m, 1.8H), 1.90 – 1.79 (m, 1.2H), 1.77 – 1.45 (m, 3H, overlapping H_2O), 1.33 – 1.16 (m, 6H).

^{13}C NMR (126 MHz, $CDCl_3$) δ 141.3, 141.1, 140.4, 139.6, 129.3, 129.2, 129.1, 127.7, 127.7, 127.6, 78.7, 74.6, 68.9, 68.9, 50.0, 47.7, 42.2, 39.5, 35.2, 35.0, 34.3, 30.0, 30.0, 28.9, 22.0, 21.6, 17.7.

Commercial loxoprofen is supplied as a mixture of diastereomers. The major cyclopentanol configuration is assigned as *trans* from comparison of chemical shift and coupling constants to *trans*-methylcyclopentanol[29] and *cis*-methylcyclopentanol.[30] Data were in accordance to those previously reported where assignment was not noted.[31]

(±)-3-(2-Hydroxy-ethyl)-2-((*Z*)-pent-2-enyl)-cyclopentanol 4d

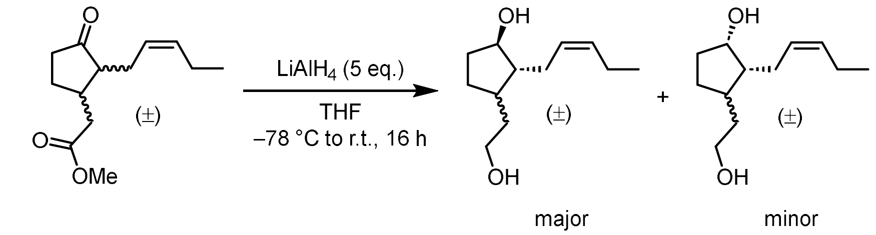

Lithium aluminium hydride (6.25 mL, 4.0 M in diethyl ether, 25.0 mmol) was added dropwise to a stirred solution of (±)-methyl jasmonate (1.09 mL, 5.00 mmol) in THF (50.0 mL) at −78 °C. The mixture was allowed to slowly warm to room temperature and stirred over 16 hours. The mixture was cooled to 0 °C, EtOAc (10 mL) then was added dropwise, followed by saturated aqueous ammonium chloride (10 mL) added dropwise, and then the mixture was diluted with water (20 mL). The solution was extracted with ethyl acetate (3 × 30 mL), washed with brine (20 mL), dried (stirred over Na_2SO_4), filtered, and all volatiles removed *in vacuo*. The crude mixture was purified by flash column chromatography (CombiFlash Isco NextGen300+, 40 g SiO_2, 50 mm Ø, 40/60 petroleum ether/EtOAc 100:0 to 0:100) to give *(±)-3-(2-hydroxy-ethyl)-2-((Z)-pent-2-enyl)-cyclopentanol* as a colourless oil (627 mg, 63%, 3.15 mmol, d.r. = 3:1).

^{1}H NMR (500 MHz, $CDCl_3$) δ 5.61 – 5.36 (m, 2H), 4.21 (td, *J* = 4.9, 2.1 Hz, 0.25H), 3.89 (dt, *J* = 6.6, 5.1 Hz, 0.75H), 3.77 – 3.57 (m, 2H), 2.23 – 2.15 (m, 1H), 2.13 – 1.97 (m, 3H), 1.94 – 1.74 (m, 3H), 1.72 – 1.17 (m, 7H, overlapping H_2O), 0.97 (t, *J* = 7.5 Hz, 3H).

^{13}C NMR (126 MHz, $CDCl_3$) δ 133.5, 132.8, 127.8, 127.3, 78.8, 74.5, 62.3, 61.9, 54.4, 54.1, 51.7, 40.4, 38.8, 38.6, 38.6, 38.2, 33.6, 33.5, 33.4, 30.4, 29.5, 29.1, 25.8, 25.7, 20.8, 20.8, 14.4, 14.4, 14.3.

HRMS (ESI^+) m/z: Found 199.1685 ($C_{12}H_{23}O_2$) requires 199.1693.

Note: the major diastereomer is assigned with the 1,2-groups on the cyclopentanol as *trans* and the minor as *cis* based on the coupling constants and comparison of chemical shifts to the previously isolated 1*R*,2*S*,3*R* diastereomer.[32] The relationship of the 2,3-groups could not be determined.

12. Synthesis of Isoureas

General Procedure F:

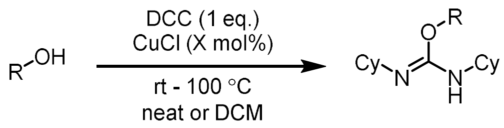

Dicyclohexylcarbodiimide (1 eq.) and alcohol (1 eq.) were mixed to which CuCl (X mol%) was added. If the alcohol was a solid, DCM (ca. 1-3 mL), was added to homogenise the mixture. The reaction mixture was then stirred at the appropriate temperature for 18 h in a sealed tube. Upon reaction completion DCM was added (ca. 3 mL) and the reaction mixture was filtered through a plug of celite/sand (4:1 volume ratio) or a plug of silica and the product was used without any further purification.

***O*-(2-Ethyl-hexyl)-*N*,*N'*-dicyclohexylisourea S1a**

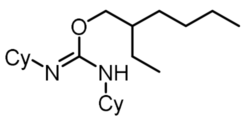

According to general procedure F, dicyclohexylcarbodiimide (1 g, 4.8 mmol), 2-ethylhexanol (758 μL, 4.8 mmol) and CuCl (25 mg, 5 mol%) were reacted neat in a sealed tube at 60 °C for 48 h. DCM (ca. 2 mL) was then added to the reaction mixture which was then filtered through a plug of silica and concentrated *in vacuo* yielding the isourea product as a yellow oil (1102 mg, 3.28 mmol, 68%).

^{1}H NMR (500 MHz, $CDCl_3$) δ 3.92 (d, *J* = 5.5 Hz, 2H), 3.48 – 3.47 (m, 1H), 3.38 – 3.34 (m, 1H), 1.92 – 1.90 (m, 2H), 1.77 – 1.69 (m, 6H), 1.60 – 1.51 (m, 3H), 1.42 – 1.04 (m, 19H), 0.90 – 0.87 (m, 6H).

^{13}C NMR (126 MHz, $CDCl_3$) δ 152.0, 67.1, 55.1, 50.6, 39.4, 34.7, 34.6, 30.9, 29.2, 26.1, 25.8, 25.5, 25.2, 24.3, 23.1, 14.2, 11.4.

HRMS (ESI^+) m/z: Calcd for $C_{21}H_{41}N_2O$ 337.3213 $[M+H]^+$; Found 337.3212.

***O*-(*iso*Propyl)-*N*,*N'*-dicyclohexylisourea S1b**

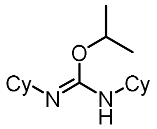

According to general procedure F, dicyclohexylcarbodiimide (3 g, 14 mmol), *iso*propanol (1.1 mL, 14 mmol) and CuCl (50 mg, 4 mol%) were reacted neat in a sealed tube at 60 °C for 18 h. DCM (ca. 2 mL) was then added to the reaction mixture which was then filtered through a plug of silica and concentrated *in vacuo* yielding the isourea product as a yellow oil (2634 mg, 9.94 mmol, 71%).

^{1}H NMR (500 MHz, $CDCl_3$) δ 5.01 – 4.96 (m, 1H), 3.44 – 3.36 (m, 2H), 2.78 (s, 1H), 1.91 – 1.89 (m, 2H), 1.73 – 1.57 (m, 8H), 1.32 – 1.05 (m, 16H).

^{13}C NMR (126 MHz, $CDCl_3$) δ 150.8, 77.3, 66.7, 54.9, 50.2, 34.7, 34.5, 26.2, 25.9, 25.3, 25.1, 22.0.

HRMS (ESI^+) m/z: Calcd for $C_{16}H_{30}N_2O$ 266.2352 $[M+H]^+$; Found 266.2351.

***O*-(Cyclopentyl)-*N*,*N'*-dicyclohexylisourea s1c**

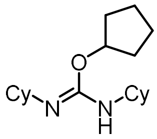

According to general procedure F, dicyclohexylcarbodiimide (700 mg, 3.39 mmol), cyclopentanol (308 µL, 3.39 mmol) and CuCl (30 mg, 9 mol%) were reacted neat in a sealed tube at 60 °C for 18 h. DCM (ca. 2 mL) was then added to the reaction mixture which was then filtered through a plug of silica and concentrated *in vacuo* yielding the isourea product as a yellow/brown solid (950 mg, 3.25 mmol, 96%).

^{1}H NMR (500 MHz, $CDCl_3$) δ 5.18 – 5.14 (m, 1H), 3.34 – 3.28 (m, 1H), 2.79 – 2.74 (m, 1H), 1.90 – 1.87 (m, 2H), 1.80 – 1.66 (m, 12H), 1.61 – 1.51 (m, 4H), 1.34 – 1.02 (m, 10H).

^{13}C NMR (126 MHz, $CDCl_3$) δ 150.9, 76.4, 54.7, 50.3, 34.5, 34.4, 32.5, 26.0, 25.7, 25.2, 25.0, 23.7.

HRMS (ESI^+) m/z: Calcd for $C_{18}H_{33}N_2O$ 293.2587 $[M+H]^+$; Found 293.2586.

O-(Cycloheptyl)-N,N'-dicyclohexylisourea S1d

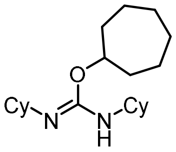

According to general procedure F, dicyclohexylcarbodiimide (1 g, 4.8 mmol), cycloheptanol (578 µL, 4.8 mmol) and CuCl (30 mg, 6 mol%) were reacted neat in a sealed tube at 60 °C for 18 h. DCM (ca. 2 mL) was then added to the reaction mixture which was then filtered through a plug of silica followed by vacuum distillation (300 °C, 2 x 10^{-1} mbar) yielding the isourea product as a colourless oil (1.06 g, 3.31 mmol, 69%).

^{1}H NMR (500 MHz, $CDCl_3$) δ 4.95 – 4.92 (m, 1H), 3.40 – 3.34 (m, 1H), 2.80 – 2.76 (m, 1H), 1.92 – 1.86 (m, 4H), 1.74 – 1.54 (m, 18H), 1.46 – 1.41 (m, 2H), 1.36 – 1.02 (m, 10H).

^{13}C NMR (126 MHz, $CDCl_3$) δ 150.6, 73.9, 54.8, 50.3, 34.7, 34.6, 33.7, 28.7, 26.2, 25.9, 25.3, 25.2, 23.1.

HRMS (ESI$^+$) m/z: Calcd for $C_{20}H_{37}N_2O$ 321.2900 [M+H]$^+$; Found 321.2907.

O-(1-Phenylethyl)-N,N'-dicyclohexylisourea S1e

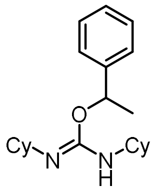

According to general procedure F, dicyclohexylcarbodiimide (674 mg, 3.27 mmol), 1-phenylethanol (395 µL, 3.27 mmol) and CuCl (30 mg, 9 mol%) were reacted neat in a sealed tube at 60 °C for 18 h. DCM (ca. 2 mL) was then added to the reaction mixture which was then filtered through a plug of silica and concentrated *in vacuo* yielding the isourea product as a yellow oil (752 mg, 2.29 mmol, 70%).

^{1}H NMR (500 MHz, Methanol-d4) δ = 7.38 – 7.32 (m, 4H), 7.28 – 7.25 (m, 1H), 5.80 (q, J = 6.5 Hz, 1H), 3.28 – 3.24 (m, 2H), 1.86 – 1.83 (m, 2H), 1.78 – 1.75 (m, 2H), 1.71 – 1.62 (m, 6H), 1.52 (d, J = 6.5 Hz, 3H), 1.42 – 1.12 (m, 10H).

^{13}C NMR (126 MHz, Methanol-d4) δ = 153.8, 144.2, 129.2, 128.5, 127.1, 74.7, 35.1, 26.9, 26.4, 26.3, 22.9.

HRMS (ESI^{+}) m/z: Calcd for $C_{21}H_{33}N_2O$ 329.2587 $[M+H]^{+}$; Found 329.2585.

***O*-(Benzhydryl)-*N*,*N'*-dicyclohexylisourea S1f**

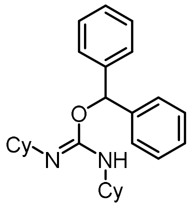

According to general procedure F, dicyclohexylcarbodiimide (500 mg, 2.43 mmol), 1,1-diphenylmethanol (446 mg, 2.43 mmol) and CuCl (30 mg, 9 mol%) were reacted neat in a sealed tube at 60 °C for 18 h. DCM (ca. 2 mL) was then added to the reaction mixture which was then filtered through a plug of sand and concentrated *in vacuo* yielding the isourea product as a white solid (444 mg, 1.14 mmol, 47%).

^{1}H NMR (500 MHz, $CDCl_3$) δ 7.38 (d, *J* = 7.4 Hz, 4H), 7.30 (t, *J* = 7.4 Hz, 4H), 7.22 (t, *J* = 7.3 Hz, 2H), 7.06 (s, 1H), 3.63 – 3.56 (m, 1H), 2.83 – 2.78 (m, 1H), 1.96-1.92 (m, 2H), 1.74 – 1.54 (m, 8H), 1.40 – 1.06 (m, 10H).

^{13}C NMR (126 MHz, $CDCl_3$) δ 149.5, 142.5, 128.1, 127.5, 127.1, 76.2, 54.4, 50.6, 34.7, 34.3, 26.2, 25.8, 25.2, 24.8.

HRMS (ESI^+) m/z: Calcd for $C_{26}H_{35}N_2O$ 391.2743 $[M+H]^+$; Found 391.2739.

_O_-(3-Phenyl-1-propyl)-_N,N'_-dicyclohexylisourea S1g

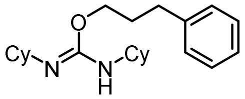

According to general procedure F, dicyclohexylcarbodiimide (1 g, 4.8 mmol), 3-phenylpropanol (653 µL, 4.8 mmol) and CuCl (30 mg, 6 mol%) were reacted neat in a sealed tube at 60 °C for 18 h. DCM (ca. 2 mL) was then added to the reaction mixture which was then filtered through a plug of silica and concentrated *in vacuo* yielding the isourea product as a yellow oil (1102 mg, 3.22 mmol, 67%).

^{1}H NMR (500 MHz, $CDCl_3$) δ 7.30 – 7.27 (m, 2H), 7.21 – 7.16 (m, 3H), 4.07 (t, *J* = 6.3 Hz, 2H), 3.51 – 3.50 (m, 1H), 3.44 – 3.37 (m, 1H), 2.80 – 2.75 (m, 1H), 2.73 – 2.69 (m, 2H), 1.98 – 1.92 (m, 4H), 1.74 – 1.70 (m, 6H), 1.62 – 1.59 (m, 2H), 1.37 – 1.07 (m, 10H).

^{13}C NMR (126 MHz, $CDCl_3$) δ 151.6, 142.2, 128.5, 128.4, 125.9, 64.3, 55.0, 50.6, 34.7, 34.6, 32.7, 31.0, 26.1, 25.8, 25.4, 25.2.

HRMS (ESI^+) m/z: Calcd for $C_{22}H_{35}N_2O$ 343.2743 $[M+H]^+$; Found 343.2745.

***O*-(Hexyl)-*N*,*N'*-dicyclohexylisourea S1h**

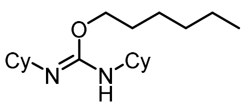

According to general procedure F, dicyclohexylcarbodiimide (4.4 g, 21.33 mmol), 1-hexanol (2.6 mL, 21.33 mmol) and CuCl (30 mg, 1 mol%) were reacted neat in a sealed tube at 60 °C for 18 h. DCM (ca. 2 mL) was then added to the reaction mixture which was then filtered through a plug of silica and concentrated *in vacuo* followed by vacuum distillation (112 °C, 7 x 10^{-2} mbar) yielding isourea product as a yellow oil (5.2 g, 17.51 mmol, 82%).

^{1}H NMR (500 MHz, $CDCl_3$) δ 3.99 (t, *J* = 6.5 Hz, 2H), 3.41 – 3.34 (m, 1H), 2.77 – 2.73 (m, 1H), 1.92 – 1.90 (m, 2H), 1.77 – 1.68 (m, 6H), 1.63 – 1.58 (m, 4H), 1.40 – 1.03 (m, 16H), 0.90 – 0.87 (m, 3H).

^{13}C NMR (126 MHz, $CDCl_3$) δ 151.8, 65.0, 55.0, 50.4, 34.7, 34.5, 31.7, 29.0, 26.1, 25.9, 25.8, 25.5, 25.2, 22.7, 14.1.

HRMS (ESI^+) m/z: Calcd for $C_{19}H_{37}N_2O$ 309.2900 $[M+H]^+$; Found 309.2907.

_O_-(Naphthylmethyl)-_N,N'_-dicyclohexylisourea S1i

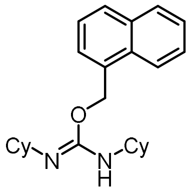

According to general procedure F, dicyclohexylcarbodiimide (1 g, 4.8 mmol), 1-naphthylmethanol (759 mg, 4.8 mmol) and CuCl (30 mg, 1 mol%) in DCM (ca. 1 mL) were reacted in a sealed tube at 60 °C for 18 h. DCM (ca. 2 mL) was then added to the reaction mixture which was then filtered through a plug of silica and concentrated *in vacuo* yielding the isourea product as a yellow oil (1.17 g, 3.21 mmol, 67%).

^{1}H NMR (500 MHz, $CDCl_3$) δ 8.14 – 8.12 (m 1H), 7.88 – 7.86 (m, 1H), 7.82 – 7.80 (d, *J* = 8.2 Hz, 1H), 7.57 – 7.43 (m, 4H), 5.57 (s, 2H), 3.56 – 3.54 (m, 1H), 3.39 – 3.32 (m, 1H), 2.89 – 2.85 (m, 1H), 1.83 – 1.77 (m, 6H), 1.65 – 1.60 (m, 3H), 1.53 – 1.50 (m, 1H), 1.41 – 1.00 (m, 10H).

^{13}C NMR (126 MHz, $CDCl_3$) δ 151.3, 133.8, 133.7, 132.0, 128.5, 128.5, 126.9, 126.0, 125.7, 125.3, 124.4, 65.2, 55.1, 50.4, 34.7, 34.6, 26.1, 25.7, 25.4, 25.0.

HRMS (ESI$^+$) m/z: Calcd for $C_{24}H_{33}N_2O$ 365.2587 $[M+H]^+$; Found 365.2592.

_O_-Benzyl-_N,N'_-dicyclohexylisourea S1j

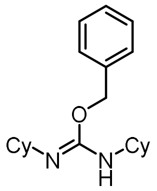

According to general procedure F, dicyclohexylcarbodiimide (4.4 g, 21.33 mmol), benzyl alcohol (2.2 mL, 21.33 mL) and CuCl (21 mg, 1 mol%) were reacted neat in a sealed tube at 60 °C for 18 h. The reaction mixture was then directly subjected to vacuum distillation (75 °C, 4 x 10^{-2} mbar) yielding the isourea product as a colourless oil (4.98 g, 15.84 mmol, 74%).

^{1}H NMR (500 MHz, $CDCl_3$) *δ* 7.37 – 7.26 (m, 5H), 4.70 (s, 2H), 3.22 – 3.16 (m, 2H), 1.93 – 1.90 (m, 4H), 1.74 – 1.72 (m, 5H), 1.58 – 1.55 (m, 2H), 1.37 – 1.18 (m, 10H).

^{13}C NMR (126 MHz, $CDCl_3$) *δ* 151.3, 138.3, 128.3, 127.6, 127.3, 66.6, 55.0, 35.0, 34.6, 34.6, 26.1, 25.8, 25.4, 25.1.

HRMS (ESI^{+}) m/z: Calcd for $C_{20}H_{31}N_2O$ 315.2430 $[M+H]^{+}$; Found 315.2431.

***O*-(4-Trifluoromethyl-benzyl)-*N,N'*-dicyclohexylisourea S1k**

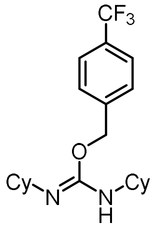

According to general procedure F, dicyclohexylcarbodiimide (500 mg, 2.42 mmol), 4-trifluoromethylbenzyl alcohol (331 µL, 2.42 mmol) and CuCl (30 mg, 13 mol%) were reacted neat in a sealed tube at 60 °C for 18 h. DCM (ca. 3 mL) was then added to the reaction mixture which was then filtered through a plug of silica and concentrated *in vacuo* yielding the isourea product as a yellow oil (661 mg, 1.73 mmol, 72%).

^{1}H NMR (500 MHz, $CDCl_3$) δ 7.59 (d, *J* = 8 Hz, 2H), 7.47 (d, *J* = 8 Hz, 2H), 5.17 (s, 2H), 3.49 – 3.42 (m, 1H), 2.84 – 2.79 (m, 1H), 1.94 – 1.91 (m, 2H), 1.75 – 1.70 (m, 6H), 1.62 – 1.59 (m, 2H), 1.36 – 1.07 (m, 10H).

^{13}C NMR (126 MHz, $CDCl_3$) δ 150.8, 142.5, 129.5 (q, *J* = 32 Hz), 127.6, 125.3 (q, *J* = 3.7 Hz), 65.7, 54.9, 50.6, 34.6, 34.6, 26.1, 25.8, 25.2, 25.1.

^{19}F NMR (471 MHz, $CDCl_3$) δ –62.45.

HRMS (ESI^+) m/z: Calcd for $C_{21}H_{30}FN_2O$ 383.2304 $[M+H]^+$; Found 383.2314.

***O*-(4-Chloro-benzyl)-*N,N'*-dicyclohexylisourea S1l**

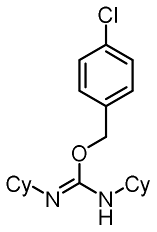

According to general procedure F, dicyclohexylcarbodiimide (500 mg, 2.42 mmol), 4-chlorobenzyl alcohol (345 mg, 2.42 mmol) and CuCl (30 mg, 13 mol%) in DCM (ca. 1 mL) were reacted in a sealed tube at 60 °C for 18 h. DCM (ca. 2 mL) was then added to the reaction mixture which was then filtered through a plug of silica and concentrated *in vacuo* yielding the isourea product as a white solid (552 mg, 1.59 mmol, 66%).

^{1}H NMR (500 MHz, $CDCl_3$) δ 7.30 (s, 4H), 5.07 (s, 2H), 3.54 – 3.52 (m, 1H) 3.46 – 3.39 (m, 1H), 2.83 – 2.78 (m, 1H), 1.92 – 1.89 (m, 2H), 1.75 – 1.68 (m, 6H), 1.64 – 1.58 (m, 2H), 1.35 – 1.05 (m, 10H).

^{13}C NMR (126 MHz, $CDCl_3$) δ 151.0, 136.8, 133.1, 129.1, 128.4, 65.8, 54.9, 53.5, 50.6, 34.6, 26.1, 25.8, 25.3, 25.1.

HRMS (ESI^+) m/z: Calcd for $C_{20}H_{30}ClN_2O$ 349.2041 $[M+H]^+$; Found 349.2053.

***O*-(4-Nitro-benzyl)-*N,N'*-dicyclohexylisourea S1m**

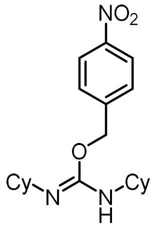

According to general procedure F, dicyclohexylcarbodiimide (500 mg, 2.42 mmol), 4-nitrobenzyl alcohol (370 mg, 2.42 mmol) and CuCl (30 mg, 13 mol%) in DCM (ca. 1 mL) were reacted in a sealed tube at 60 °C for 18 h. DCM (ca. 2 mL) was then added to the reaction mixture which was then filtered through a plug of silica and concentrated *in vacuo* yielding the isourea product as a yellow solid (643 mg, 1.79 mmol, 74%).

^{1}H NMR (500 MHz, $CDCl_3$) δ 8.22 (d, *J* = 8.7 Hz, 2H), 7.54 (d, *J* = 8.7 Hz, 2H), 5.24 (s, 2H), 3.59 – 3.58 (m, 1H), 3.52 – 3.50 (m, 1H), 3.49 – 3.45 (m, 1H) 2.86 – 2.81 (m, 1H), 1.97 – 1.95 (m, 2H), 1.76 – 1.58 (m, 8H), 1.37 – 1.08 (m, 10H).

^{13}C NMR (126 MHz, $CDCl_3$) δ 150.3, 147.2, 146.1, 127.8, 123.5, 65.2, 54.8, 50.7, 34.6, 34.5, 26.0, 25.7, 25.1, 25.1.

HRMS (ESI$^+$) m/z: Calcd for $C_{20}H_{30}N_3O_3$ 360.2281 $[M+H]^+$; Found 360.2281.

_O_-(4-Methoxy-benzyl)-_N,N'_-dicyclohexylisourea S1n

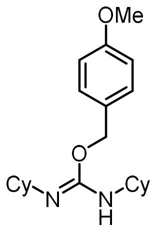

According to general procedure F, dicyclohexylcarbodiimide (745 mg, 3.6 mmol), 4-methoxybenzyl alcohol (499 mg, 3.6 mmol) and CuCl (40 mg, 11 mol%) were reacted in a sealed tube at 60 °C for 18 h. DCM (ca. 2 mL) was then added to the reaction mixture which was then filtered through a plug of celite and concentrated *in vacuo* yielding the isourea product as a yellow oil (775 mg, 2.25 mmol, 63%).

^{1}H NMR (600 MHz, $CDCl_3$) δ 7.30 (d, *J* = 8.7 Hz, 2H), 6.87 (d, *J* = 8.7 Hz, 2H), 5.04 (s, 2H), 3.81 (s, 3H), 3.45 – 3.38 (m, 1H), 2.83 – 2.78 (m, 1H), 1.91 – 1.88 (m, 2H), 1.78 – 1.56 (m, 8H), 1.34 – 1.04 (m, 10H).

^{13}C NMR (126 MHz, $CDCl_3$) δ 158.9, 151.3, 130.3, 129.2, 113.6, 66.2, 55.2, 54.9, 50.2, 34.5, 34.4, 26.0, 25.7, 25.3, 24.9.

HRMS (ESI$^+$) m/z: Calcd for $C_{21}H_{33}N_2O$ 345.2536 [M+H]$^+$; Found 345.2534.

***O*-(4-Methyl-benzyl)-*N*,*N'*-dicyclohexylisourea S1o**

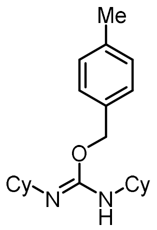

According to general procedure F, dicyclohexylcarbodiimide (507 mg, 2.46 mmol), 4-methylbenzyl alcohol (300 mg, 2.46 mmol) and CuCl (30 mg, 12 mol%) in DCM (ca. 1 mL) were reacted in a sealed tube at 60 °C for 18 h. DCM (ca. 2 mL) was then added to the reaction mixture which was then filtered through a plug of silica and concentrated *in vacuo* yielding the isourea product as a yellow oil (629 mg, 1.92 mmol, 78%).

^{1}H NMR (500 MHz, $CDCl_3$) δ 7.27 – 7.26 (m, 2H), 7.15 (d, *J* = 8.1 Hz, 2H), 5.07 – 5.06 (m, 2H), 3.46 – 3.41 (m 1H), 2.84 – 2.80 (m 1H), 2.35 (s, 3H), 1.94 – 1.90 (m, 2H), 1.77 – 1.57 (m, 8H), 1.32 – 1.05 (m, 10H).

^{13}C NMR (126 MHz, $CDCl_3$) δ 151.4, 137.0, 135.2, 129.0, 127.8, 66.6, 55.0, 50.4, 34.7, 34.6, 26.1, 25.8, 25.4, 25.1, 21.3.

HRMS (ESI^+) m/z: Calcd for $C_{21}H_{33}N_2O$ 329.2587 $[M+H]^+$; Found 329.2589.

O-(3-Methoxy-benzyl)-N,N'-dicyclohexylisourea S1p

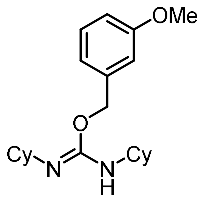

According to general procedure F, dicyclohexylcarbodiimide (1 g, 4.8 mmol), 3-methoxybenzyl alcohol (596 µL, 4.8 mmol) and CuCl (30 mg, 1 mol%) in DCM (ca. 1 mL) were reacted in a sealed tube at 60 °C for 18 h. DCM (ca. 2 mL) was then added to the reaction mixture which was then filtered through a plug of silica and concentrated *in vacuo* yielding the isourea product as a yellow oil (1.10 g, 3.2 mmol, 67%).

^{1}H NMR (600 MHz, $CDCl_3$) δ 7.25 (t, *J* = 8.1 Hz, 1H), 6.95 (m, 2H), 6.82 (d, *J* = 8.1 Hz, 2H), 5.09 (s, 2H), 3.80 (s, 3H), 3.56 – 3.55 (m, 1H), 3.50 – 3.44 (m, 1H), 2.83 – 2.81 (m, 1H), 1.95 – 1.93 (m, 2H), 1.76 – 1.69 (m, 6H), 1.62 – 1.58 (m, 2H), 1.34 – 1.08 (m, 10H).

^{13}C NMR (151 MHz, $CDCl_3$) δ 159.6, 151.3, 139.9, 129.3, 119.8, 113.1, 112.8, 66.5, 55.2, 55.0, 50.5, 34.6, 26.1, 25.8, 25.4, 25.1.

HRMS (ESI$^+$) m/z: Calcd for $C_{21}H_{33}N_2O_2$ 345.2536 $[M+H]^+$; Found 345.2530.

O-(2-Bromo-benzyl)-N,N'-dicyclohexylisourea S1q

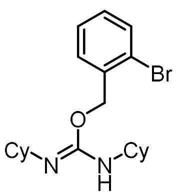

According to general procedure F, dicyclohexylcarbodiimide (1 g, 4.8 mmol), 2-bromobenzyl alcohol (898 mg, 4.8 mmol) and CuCl (30 mg, 1 mol%) in DCM (ca. 1 mL) were reacted in a sealed tube at 60 °C for 18 h. DCM (ca. 2 mL) was then added to the reaction mixture which was then filtered through a plug of silica and concentrated *in vacuo* yielding the isourea product as a yellow oil (1.08 g, 2.75 mmol, 57%).

^{1}H NMR (500 MHz, Methanol-d4) δ = 7.61 (dd, J = 7.9, 1.2 Hz, 1H), 7.48 (dd, J = 7.6, 1.8 Hz, 1H), 7.37 (td, J = 7.5, 1.2 Hz, 1H), 7.24 (td, J = 7.7, 1.7 Hz, 1H), 5.54 (s, 2H) ,3.28 – 3.18 (m, 2H), 1.82 – 1.72 (m, 9H), 1.65 – 1.61 (m, 2H), 1.39 – 1.14 (m, 10H).

^{13}C NMR (126 MHz, Methanol-d4) δ = 154.5, 138.0, 133.8, 131.0, 130.6, 128.5, 124.2, 68.4, 53.8, 35.2, 26.8, 26.4.

HRMS (ESI$^+$) m/z: Calcd for $C_{20}H_{30}N_2OBr$ 393.1536 [M+H]$^+$; Found 393.1540.

***O*-(2-(*E*)-3,7-Dimethyl-octa-2,6-dienyl)-*N*,*N'*-dicyclohexylisourea S1r**

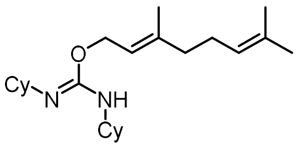

According to general procedure F, dicyclohexylcarbodiimide (1 g, 4.8 mmol), geraniol (842 µL, 4.8 mmol) and CuCl (25 mg, 5 mol%) were reacted neat in a sealed tube at 60 °C for 18 h. DCM (ca. 2 mL) was then added to the reaction mixture which was then filtered through a cotton/sand plug and concentrated *in vacuo* yielding the isourea product as a yellow/green oil (794 mg, 2.21 mmol, 46%)

^{1}H NMR (500 MHz, $CDCl_3$) δ 5.38 – 5.35 (m, 1H), 5.11 – 5.08 (m, 1H), 4.57 (d, *J* = 6.75 Hz, 2H), 3.41 – 3.36 (m, 1H), 2.12 – 2.07 (m, 2H), 2.04 – 2.01 (m, 2H), 1.92 – 1.89 (m, 2H), 1.77 – 1.69 (m, 13H), 1.60 – 1.55 (m, 5H), 1.36 – 1.02 (m, 10H).

^{13}C NMR (126 MHz, $CDCl_3$) δ 151.6, 139.8, 131.6, 124.2, 120.5, 62.0, 55.0, 50.3, 39.7, 34.7, 34.5, 26.5, 26.1, 25.8, 25.8, 25.4, 25.1, 17.8, 16.7.

HRMS (ESI$^+$) m/z: Calcd for $C_{23}H_{41}N_2O$ = 361.32134 [M+H]$^+$; Found 361.3218.

4-(4-(3-*O*-(*N*,*N'*-Dicyclohexylcarbamimidyl)-propoxy)phenyl)butan-2-*O*-*N*,*N'*-dicyclohexylcarbamimidate 1

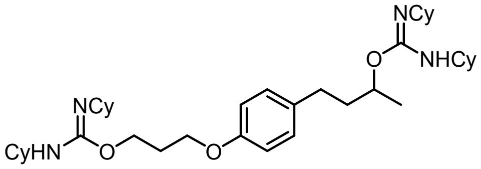

A mixture of 4-(4-(3-hydroxypropoxy)phenyl)butan-2-ol (500 mg, 2.20 mmol), dicyclohexylcarbodiimide (920 mg, 4.40 mmol), and copper(I) chloride (60.0 mg, 0.594 mmol) was stirred at 100 °C for 16 hours. The mixture was diluted with CH_2Cl_2 (2 mL), passed througha celite plug, and concentrated *in vacuo* to yield *4-(4-(3-O-(N,N'-dicyclohexylcarbamimidyl)-propoxy)phenyl)butan-2-O-N,N'-dicyclohexylcarbamimidate* as an orange oil (614 mg, 43%, 0.956 mmol).

^{1}H NMR (500 MHz, $CDCl_3$) δ 7.11 (d, J = 8.6 Hz, 2H), 6.82 (d, J = 8.5 Hz, 2H), 5.02 – 4.96 (m, 1H), 4.21 (t, J = 6.2 Hz, 2H), 4.06 (t, J = 6.5 Hz, 2H), 3.42 – 3.85 (m, 2H), 2.83 – 2.76 (m, 2H), 2.69 – 2.56 (m, 2H), 2.15 – 2.10 (m, 2H), 1.97 – 1.87 (m, 6H), 1.78 – 1.70 (m, 14H), 1.63 – 1.57 (m, 4H), 1.38 – 1.08 (m, 23H).

^{13}C NMR (126 MHz, $CDCl_3$) δ 157.2, 151.4, 150.5, 134.7, 129.3, 114.4, 69.5, 65.2, 61.7, 55.9, 54.9, 54.8, 50.6, 50.5, 50.4, 50.3, 38.5, 35.0, 34.7, 34.6, 34.6, 34.5, 31.0, 29.1, 26.2, 26.1, 25.9, 25.8, 25.6, 25.4, 25.3, 25.2, 25.2, 25.1, 24.8, 19.8.

HRMS (ESI$^+$) m/z: Calcd for $C_{39}H_{65}N_4O_3$ 637.5051 $[M+H]^+$; Found 637.5074.

4-(4-(3-*O*-(*N*,*N'*-Di*iso*propylcarbamimidyl)-propoxy)phenyl)butan-2-*O*-*N*,*N'*-di*iso*propylcarbamimidate S1s

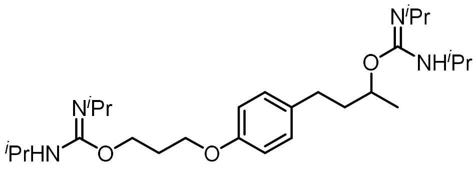

A mixture of 4-(4-(3-hydroxypropoxy)phenyl)butan-2-ol (997 mg, 4.44 mmol), di*iso*propyl carbodiimide (1.40 mL, 9.00 mmol), and copper(I) chloride (119 mg, 1.20 mmol) was stirred at 100 °C for 16 hours. The mixture was directly purified by flash column chromatography (CombiFlash Isco NextGen300+, 24 g neutral Al_2O_3, 40/60 petroleum ether/EtOAc 100:0 to 0:100) yielding *4-(4-(3-O-(N,N'-diisopropylcarbamimidyl)-propoxy)phenyl)butan-2-O-N,N'-diisopropylcarbamimidate* as a pale yellow oil (2.07 g, 97%, 4.31 mmol).

^{1}H NMR (601 MHz, $CDCl_3$) δ 7.12 – 7.06 (m, 2H), 6.84 – 6.78 (m, 2H), 4.97 (sext., *J* = 6.2 Hz, 1H), 4.19 (td, *J* = 6.2, 1.4 Hz, 2H), 4.05 (td, *J* = 6.6, 3.0 Hz, 2H), 3.80 – 3.71 (m, 2H), 3.38 (dd, *J* = 15.1, 7.7 Hz, 1H), 3.16 (dsept., *J* = 12.4, 6.2 Hz, 2H), 2.73 – 2.54 (m, 2H), 2.14 – 2.08 (m, 2H), 1.95 – 1.85 (m, 1H), 1.79 – 1.68 (m, 1H), 1.22 (app. dd, *J* = 6.3, 2.5 Hz, 4H), 1.16 – 1.04 (m, 24H).

^{13}C NMR (151 MHz, $CDCl_3$) δ 157.1, 151.5, 150.7, 134.6, 129.2, 114.3, 69.4, 65.1, 61.7, 46.2, 46.2, 43.4, 43.3, 41.1, 38.4, 31.2, 30.9, 29.1, 24.6, 24.4, 24.4, 24.3, 24.1, 24.0, 24.0, 19.7.

HRMS (ESI$^+$) m/z: Found 477.3796 ($C_{27}H_{49}N_4O_3$) requires 477.3799.

(±)-3-(2-*O*-(*N,N'*-Di*iso*propylcarbamimidyl)-ethyl)-2-((*Z*)-pent-2-enyl)-cyclopentan-1-*O*-*N,N'*-di*iso*propylcarbamimidate S1t

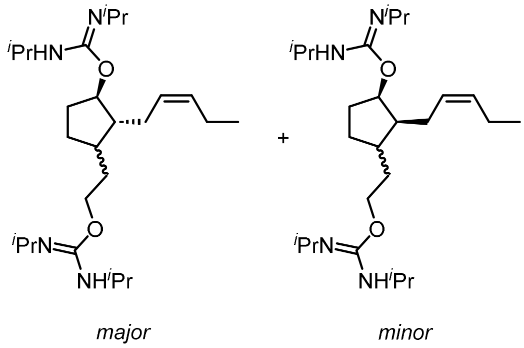

A mixture of (±)-3-(2-hydroxy-ethyl)-2-((*Z*)-pent-2-enyl)-cyclopentanol (397 mg, 2.00 mmol), di*iso*propyl carbodiimide (658 µL, 4.20 mmol), and copper(I) chloride (54.0 mg, 0.540 mmol) was stirred at 100 °C for 16 hours. The mixture was directly purified by flash column chromatography (CombiFlash Isco NextGen300+, 24 g neutral Al_2O_3, 40/60 petroleum ether/EtOAc 100:0 to 0:100) yielding *(±)-3-(2-O-(N,N'-Diisopropylcarbamimidyl)-ethyl)-2-((Z)-pent-2-enyl)-cyclopentan-1-O-N,N'-diisopropylcarbamimidate* as a pale yellow oil (637 mg, 71%, 1.41 mmol, d.r. = 6:1).

^{1}H NMR (500 MHz, $CDCl_3$) δ 5.49 – 5.29 (m, 2H), 5.14 (td, *J* = 4.9, 2.2 Hz, 0.15H minor diastereomer), 4.83 (dd, *J* = 6.1, 3.3 Hz, 0.85H major diastereomer), 4.26 – 3.63 (m, 4.15H), 3.43 – 3.28 (m, 1.85H), 3.14 (pd, *J* = 6.2, 4.4 Hz, 2H), 2.25 – 1.21 (m, 12H, overlapping with H_2O), 1.21 – 1.02 (m, 24H), 1.02 – 0.80 (m, 3H).

^{13}C NMR (126 MHz, $CDCl_3$) δ 152.0, 150.9, 132.5, 131.9, 128.4, 127.4, 80.4, 64.3, 64.2, 51.9, 50.7, 46.3, 46.3, 43.5, 43.5, 40.5, 40.3, 35.2, 31.5, 30.5, 30.3, 29.4, 24.6, 24.5, 24.5, 24.5, 24.3, 24.2, 24.1, 24.1, 23.7, 20.7, 20.6, 14.5.

HRMS (ESI$^+$) m/z: Found 451.4006 ($C_{26}H_{51}N_4O_2$) requires 451.4007.

Note: major diastereomer assumed to match the starting diol.

***O*-(1-(3-Phenyl)propyl)-*N,N'*-di*iso*propylisourea 6a/13a**

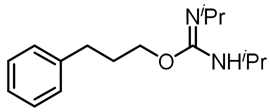

A mixture of 3-phenylpropanol (544 µL, 4.00 mmol), di*iso*propyl carbodiimide (626 µL, 4.00 mmol), and copper(I) chloride (20.0 mg, 0.200 mmol) was stirred at room temperature for 16 hours. The mixture was directly purified by flash column chromatography (CombiFlash Isco NextGen300+, 24 g neutral Al_2O_3, 40/60 petroleum ether/EtOAc 100:0 to 0:100) yielding *O-(1-(3-phenyl)propyl)-N,N'-diisopropylisourea* as a colourless oil (1.04 g, 99%, 3.96 mmol).

^{1}H NMR (500 MHz, $CDCl_3$) δ 7.31 – 7.25 (m, 2H), 7.22 – 7.15 (m, 3H), 4.07 (t, *J* = 6.3 Hz, 2H), 3.78 (dpent., *J* = 7.7, 6.3 Hz, 1H), 3.41 (d, *J* = 7.8 Hz, 1H), 3.16 (hept., *J* = 6.3 Hz, 1H), 2.76 – 2.67 (m, 2H), 2.00 – 1.93 (m, 2H), 1.14 (d, *J* = 6.4 Hz, 6H), 1.09 (d, *J* = 6.2 Hz, 6H).

^{13}C NMR (126 MHz, $CDCl_3$) δ 151.9, 142.2, 128.6, 128.5, 125.9, 64.4, 46.3, 43.6, 32.7, 31.0, 24.5, 24.2.

HRMS (ESI$^+$) m/z: Found 263.2118 ($C_{16}H_{27}N_2O$) requires 263.2118.

(±)-*O*-(2-(4-Phenyl)butyl)-*N*,*N'*-di*iso*propylisourea 6b/13b

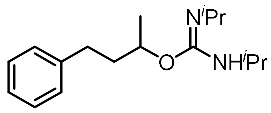

A mixture of (±)-4-phenylbutan-2-ol (774 µL, 5.00 mmol), di*iso*propyl carbodiimide (861 µL, 5.50 mmol), and copper(I) chloride (25.0 mg, 0.250 mmol) was stirred at 60 °C for 16 hours. The mixture was directly purified by flash column chromatography (CombiFlash Isco NextGen300+, 24 g neutral Al_2O_3, 40/60 petroleum ether/EtOAc 100:0 to 0:100) yielding (±)-*O*-(2-(4-phenyl)butyl)-*N*,*N'*-di*iso*propylisourea as a colourless oil (1.15 g, 83%, 4.17 mmol).

^{1}H NMR (500 MHz, $CDCl_3$) δ 7.31 – 7.24 (m, 2H), 7.22 – 7.15 (m, 3H), 5.04 – 4.95 (m, 1H), 3.76 (dt, *J* = 12.9, 6.4 Hz, 1H), 3.22 – 3.11 (m, 1H), 2.68 (dddd, *J* = 33.9, 13.8, 10.6, 5.8 Hz, 2H), 1.93 (dddd, *J* = 13.5, 10.6, 7.0, 5.5 Hz, 1H), 1.85 – 1.72 (m, 1H), 1.23 (app. dd, *J* = 6.3, 5.0 Hz, 4H), 1.13 (dd, *J* = 6.5, 5.5 Hz, 6H), 1.08 (dd, *J* = 6.2, 1.9 Hz, 6H).

^{13}C NMR (126 MHz, $CDCl_3$) δ 150.8, 142.7, 128.6, 128.4, 125.8, 69.5, 49.1, 46.3, 43.4, 43.3, 38.3, 32.0, 24.8, 24.5, 24.5, 24.2, 24.1, 19.9.

Data were in accordance to those previously reported.[17]

(*R*)-*O*-(2-(4-Phenyl)butyl)-*N*,*N'*-di*iso*propylisourea **(*R*)-6b** was prepared from commercial (*R*)-4-phenylbutan-2-ol following the same procedure.

***O*-Benzyl-*N*,*N'*-di*iso*propylisourea 13c**

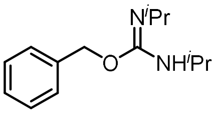

A mixture of benzyl alcohol (207 µL, 2.00 mmol), di*iso*propyl carbodiimide (344 µL, 2.20 mmol), and copper(I) chloride (10.0 mg, 0.100 mmol) was stirred at room temperature for 16 hours. The mixture was directly purified by flash column chromatography (CombiFlash Isco NextGen300+, 24 g neutral Al_2O_3, 40/60 petroleum ether/EtOAc 100:0 to 0:100) yielding *O*-benzyl-*N*,*N'*-di*iso*propylisourea as a colourless oil (459 mg, 98%, 1.96 mmol).

^{1}H NMR (500 MHz, $CDCl_3$) δ 7.40 – 7.37 (m, 2H), 7.36 – 7.32 (m, 2H), 7.30 – 7.25 (m, 1H), 5.10 (s, 2H), 3.83 (dpent., *J* = 7.9, 6.4 Hz, 1H), 3.45 (d, *J* = 7.9 Hz, 1H), 3.20 (sept., *J* = 6.2 Hz, 1H), 1.13 (dd, *J* = 6.3, 1.8 Hz, 12H).

^{13}C NMR (126 MHz, $CDCl_3$) δ 151.6, 138.2, 128.4, 127.8, 127.5, 66.8, 46.4, 43.6, 24.5, 24.2.

Data were in accordance to those previously reported.[33]

O-(tert-Butyl)-N,N'-diisopropylisourea 11

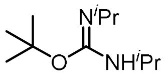

Following the procedure of Kozlowski and Zacuto,[34] a mixture of di*iso*propyl carbodiimide (1.03 mL, 6.60 mmol) and copper(I) chloride (17.0 mg, 0.170 mmol) was stirred in air for 1 hour at room temperature. A premixed solution of di*iso*propyl carbodiimide (1.57 mL, 10.0 mmol) and *tert*-butanol (1.91 mL, 20.0 mmol) was added, the vessel sealed, and the mixture stirred at room temperature for 20 hours. The mixture was purified by *kugelrohr* distillation (80 °C, 9 mbar), to yield *O*-(*tert*-butyl)-*N*,*N'*-di*iso*propylisourea as a colourless oil (1.50 g, 45%, 7.49 mmol, *E*/*Z* = 3:1).

^{1}H NMR (500 MHz, $CDCl_3$) δ 3.81 – 3.59 (m, 1H), 3.29 – 3.07 (m, 1H), 1.47 (s, 6.75H, *E*-isomer), 1.37 (s, 2.25H, *Z*-isomer), 1.09 (d, *J* = 6.4 Hz, 6H), 1.05 (d, *J* = 6.2 Hz, 6H).

^{13}C NMR (126 MHz, $CDCl_3$) δ 149.8, 148.4, 78.4, 46.4, 43.7, 43.7, 29.3, 28.6, 25.0, 24.1, 24.1.

Data were in accordance to those previously reported.[34]

As noted in the procedure by Kozlowski and Zacuto, an appropriate oxygenated atmosphere is required for catalyst activation. Stored at −20 °C *O*-(*tert*-butyl)-*N*,*N'*-di*iso*propylisourea will decompose within days.

13. Synthesis of F-BBN

Potassium [9,9-difluoro-9-borabicyclo-[3.3.1]-nonanate] (K[F$_2$BBN])

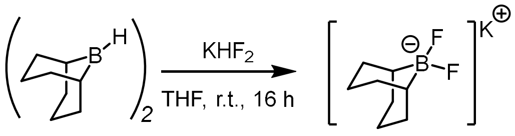

A Schlenk flask was charged with KHF_2 (3.9 g, 50 mmol) and the crystalline salt was evacuated for 1 h. Neat 9-borabicyclo-[3.3.1]-nonane (6.1 g, 50 mmol) was added, followed by dry THF (200 mL). The resulting slurry was stirred at room temperature overnight. Gas was observed to evolve slowly as KHF_2 dissolved. All volatiles were removed from the resultant clear solution under reduced pressure and the residue was washed with dry CH_2Cl_2 (2 × 20 mL). The remaining solid was dried (18 h, 60 °C, 4 × 10^{-2} mbar) to remove traces of water, giving *potassium [9,9-difluoro-9-borabicyclo-[3.3.1]-nonanate]* as a colourless solid (9.50 g, 48.0 mmol, 96%).

^{1}H NMR (500 MHz, CD_3CN) δ 1.84 – 1.66 (m, 6H), 1.59 – 1.51 (m, 4H), 1.44 – 1.32 (m, 2H), 0.23 (s, 2H).

^{11}B NMR (160 MHz, CD_3CN) δ 10.2 (t, $^1J_{BF}$ = 86.3).

^{13}C NMR (126 MHz, CD_3CN) δ 33.8 (t, J_{CF} = 3.3), 26.4, 14.3 (br).

^{19}F NMR (471 MHz, CD_3CN) δ –161.6 (q, $^1J_{BF}$ = 86.3).

HRMS (EI$^-$): Calc'd for $C_8H_{14}BF_2$: 159.11621 ([M–K]$^-$), Found: 159.11620.

9-Fluoro-9-borabicyclo-[3.3.1]-nonane (F-BBN)

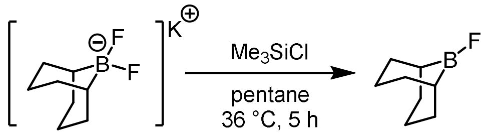

To a slurry of potassium 9,9-difluoro-9-borabicyclo-[3.3.1]-nonanate (5.00 g, 25.2 mmol) in dry pentane (40 mL) was added dry Me_3SiCl (3.20 mL, 25.2 mmol). The colourless suspension was heated to reflux temperature for 5 h. **Note**: The coolant circulating in the condenser needs to be at least 20 °C warm to prevent Me_3SiF from condensing. After cooling to room temperature, the settled mixture was filtered using a double-tipped Teflon® cannula with a glass-fibre filter. Under inert atmosphere the filtrate was then blown-dry with a stream of N_2 gas until *ca.* 1-2 mL of total volume was left. The flask was then promptly evacuated and backfilled with N_2 atmosphere 3 times (410^{-2} mbar) and F-BBN product as an off-white/yellow solid (1.45 g, 10.1 mmol, 41%).

^{1}H NMR (500 MHz, $CDCl_3$) δ 1.97 – 1.77 (m, 10H), 1.43 – 1.32 (m, 4H), 1.25 – 1.21 (m, 4H).

^{11}B NMR (160 MHz, $CDCl_3$) δ 64.1 (d, $^{1}J_{BF}$ = 133.1).

^{13}C NMR (126 MHz, $CDCl_3$) δ 33.6 (d, J_{CF} = 2.9), 26.1 (br), 23.1.

^{19}F NMR (471 MHz, $CDCl_3$) δ –46.9 (q, $^{1}J_{BF}$ = 133.1).

Data were in accordance to those previously reported.[35]

14. Computational Details

General

All DFT calculations were performed using Gaussian 16.[36] Automated job script generation and data processing was carried out using in-house developed Python code. Molecules were visualised using Chemcraft.[37] Free energy profiles were generated using modified code from autodE.[38]

DIC Elimination Free Energy Profile

Conformer ensembles were generated using CREST v2.12[39] at the GFN2-xTB(ALPB: toluene) level of theory.[40] For ground-state calculations the lowest energy conformer at the GFN2-xTB(ALPB: toluene) level of theory was taken forward for final DFT optimisation without further ranking. For transition states, an initial transition state was found at the M06-2X(D3)/Def2-SVP(SMD: toluene) level of theory (confirmed by a single imaginary frequency and IRC calculations).[41–43] This was used as a starting point for CREST, where the atoms involved in the imaginary frequency were frozen using the "--constrain" option and the conformational ensemble generated as above. The ensemble was optimised at the M06-2X(D3)/Def2-SVP(SMD: toluene) level of theory with the same atoms frozen, and ranked by the SCF energy. Structures which did not converge within a 48-hour period were dropped. Structures within 1×10^{-4} Eh were counted as rotamers, and a single structure taken forward. The ensemble was re-optimised at the same level of theory without constraints, ranked by SCF, and confirmed as transition states by frequency (one imaginary frequency) and IRC calculations, the lowest energy transition state was taken forward for final optimisation.

Final geometry optimisations were performed at the MN15/Def2-TZVP(SMD: toluene) level of theory[42,44] followed by frequency calculations at the same level to confirm a minima (zero imaginary frequencies) or saddle-point (one imaginary frequency). Transition states were confirmed by IRC calculations at the same level of theory.

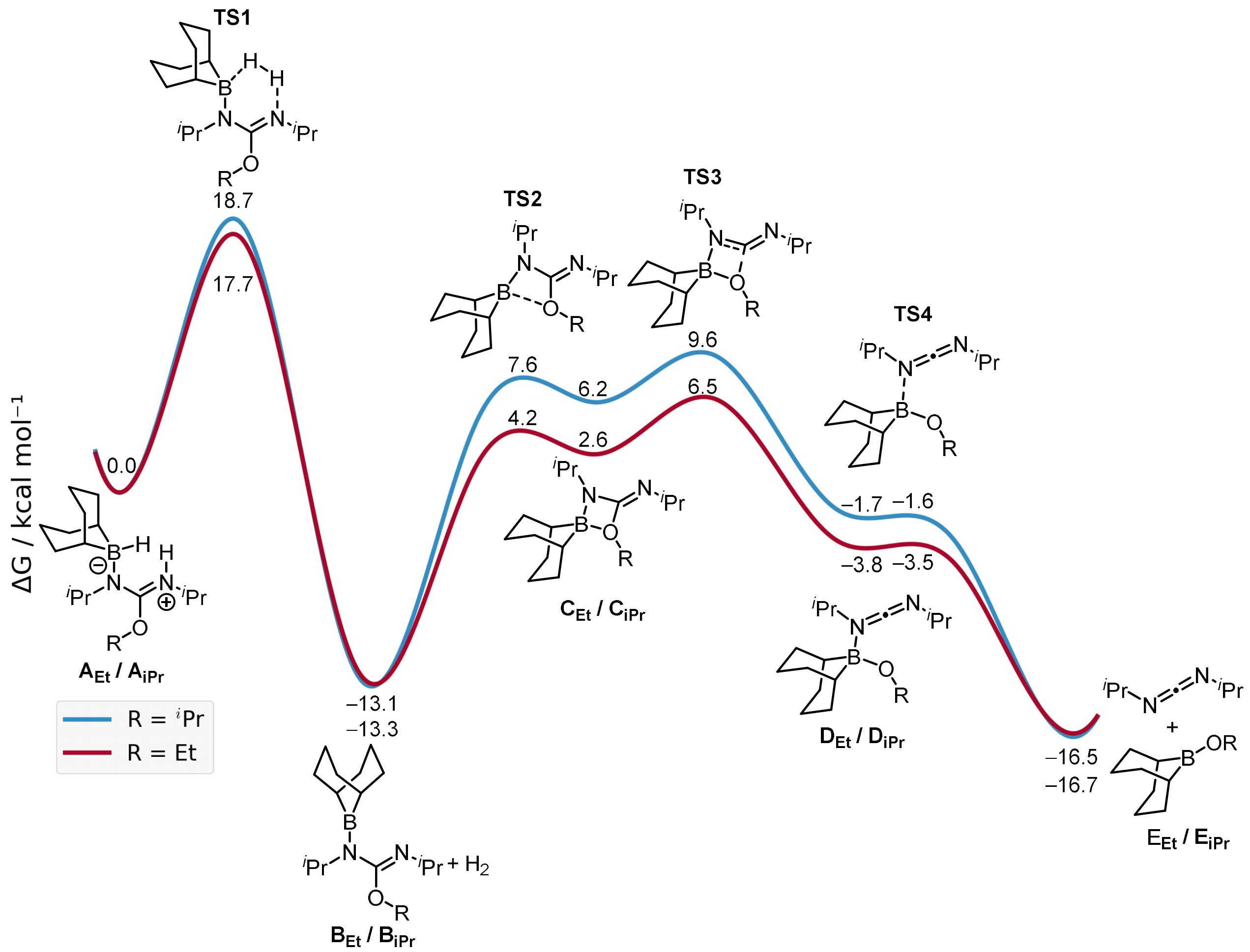

Figure S95. Free energy profile for DIC elimination.

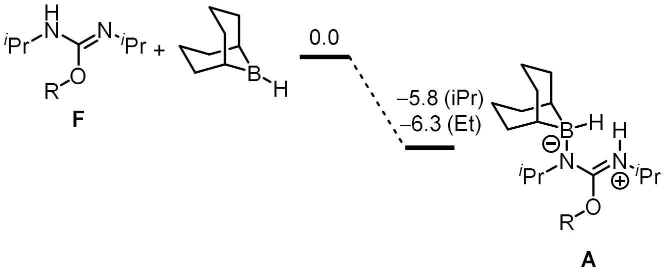

Figure S96. Formation of adduct **A** from **F** and H-BBN (in kcal mol[-1]).

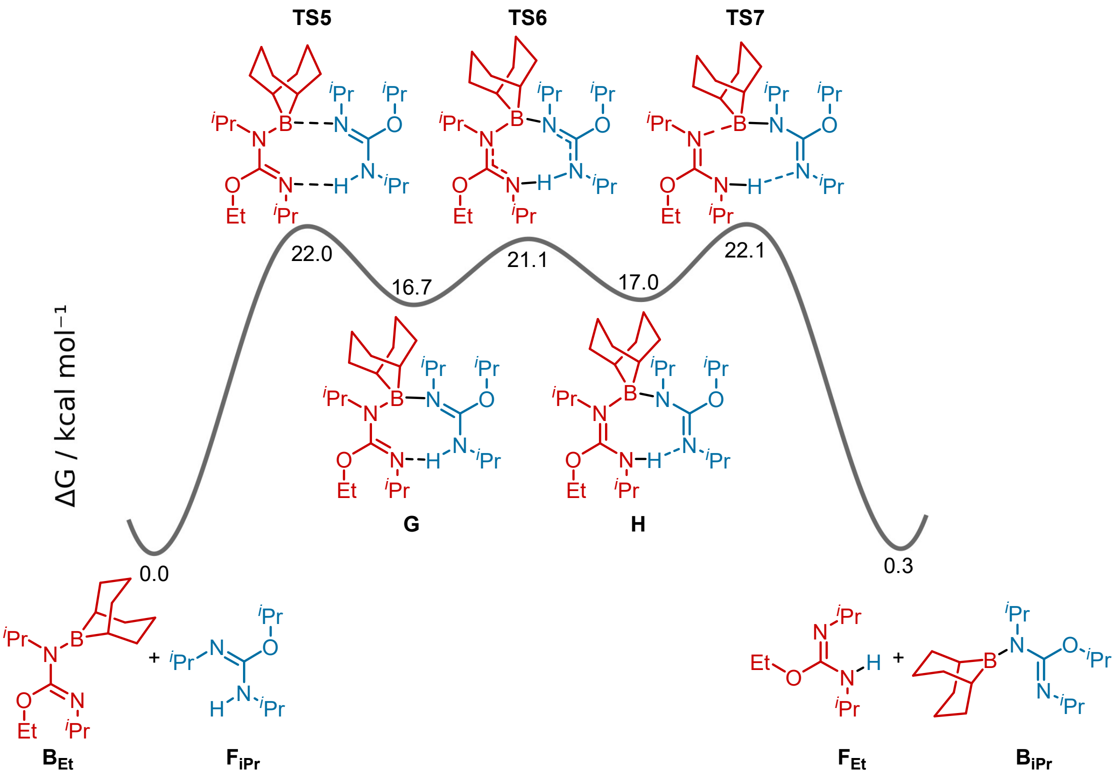

Figure S97. Free energy profile for BBN/H^+ exchange.

Distortion Energy

By a modified method of Bickelhaupt,[45] the components that make up the minima of **B** and the transition state **TS3** were heterolytically cleaved into their respective ions. Single point and thermochemical calculations were performed at the MN15/Def2-TZVP(SMD: toluene) level of theory on the fragments without optimisation. Using this model shows that the isopropyl-substituted $\mathbf{K_{iPr}}$ and $\mathbf{L_{iPr}}$ had to distort significantly more than the ethyl-substituted $\mathbf{K_{Et}}$ and $\mathbf{L_{Et}}$ in the transition state **TS3**.

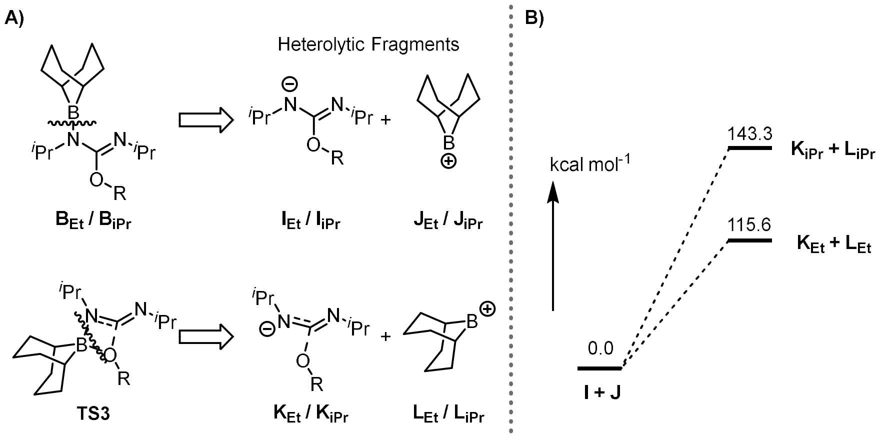

Figure S98. A) Heterolytic fragmentation of **B** and **TS3** into distorted fragments, **I**, **J**, **K**, and **L**. B) Free energy profile show distortion energy of the respective fragments (not to scale).

Fluoride Ion Affinity

According to our previously reported methodology,[46] conformer ensembles were generated using CREST v2.12[39] at the GFN2-xTB level of theory[40] the lowest energy conformer at the same level of theory was taken forward for DFT optimisation without further ranking. Geometry optimisations were completed with the DFT method using the B3LYP functional with Grimme's D3(BJ) dispersion corrections and Def2TZVPP as a basis set. All geometry optimizations were full, with no restrictions. Stationary points located in the potential energy surface were characterized as minima (no imaginary frequencies) by vibrational analysis. Single point energy calculations were performed at the DSD-BLYP(D3BJ)/Def2TZVP level of theory. Solvation free energies in dichloromethane were computed using the universal solvation model based on solute electron density (SMD) at the M05-2X/6-31G(d) level of theory. The fluoride ion affinity (FIA) for corresponding Lewis acids (LA) was calculated using TMS-isodesmic reactions according to the work by Greb.[47]

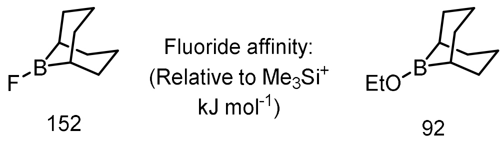

Figure S99. Calculated fluoride ion affinities of F-BBN and EtO-BBN.

Cartesian Coordinates

Cartesian coordinates of all final structures are included as .xyz files in a separate supplementary folder.

15. X-Ray Crystallography Data

Compound K[F_2BBN] was provided as large colourless block-shaped crystals (**MI23010x1**, this structure) and small colourless plate-shaped crystals (**MI23010x2**).

Crystal Data and Experimental

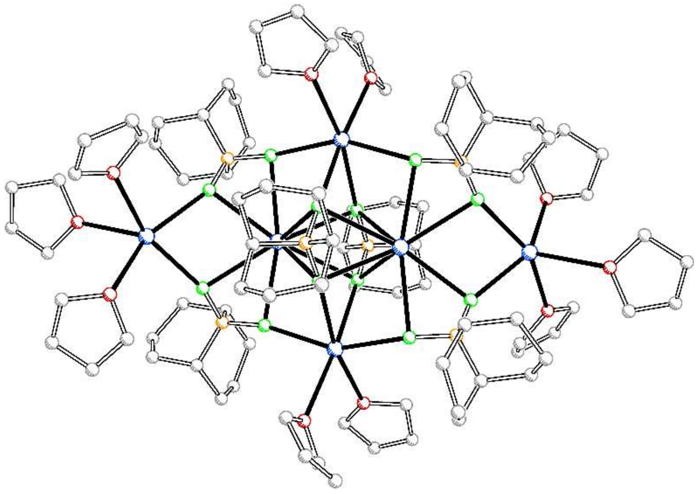

Experimental. Single clear colourless block-shaped crystals of **MI23010x1** recrystallised from dichloromethane by slow cooling. A suitable crystal with dimensions 0.34 × 0.20 × 0.20 mm^3 was selected and mounted on a MITIGEN holder in Paratone oil. on a Rigaku Oxford Diffraction SuperNova diffractometer. The crystal was kept at a steady T = 120.00(10) K during data collection. The structure was solved with the **ShelXT** 2018/2[48] solution program using dual methods and by using **Olex2** 1.5-beta[49] as the graphical interface. The model was refined with **ShelXL** 2018/3[50] using full matrix least squares minimisation on $\boldsymbol{F^2}$.

Crystal Data. $C_{88}H_{162}B_6F_{12}K_6O_{10}$, M_r = 1907.63, monoclinic, $P2_1/n$ (No. 14), a = 16.6265(3) Å, b = 13.6470(2) Å, c = 23.1149(5) Å, α = 101.234(2)°, β = γ = 90°, V = 5144.32(17) Å^3, T = 120.00(10) K, Z = 2, Z' = 0.5, μ(Cu K$_\alpha$) = 2.865, 128172 reflections measured, 10705 unique (R_{int} = 0.0885) which were used in all calculations. The final wR_2 was 0.2236 (all data) and R_1 was 0.0785 (I≥2 σ(I)).

**Compound**	**MI23010x1** CCDC 2372261
Formula	$C_{88}H_{162}B_6F_{12}K_6O_{10}$
$D_{calc.}$/ g cm^{-3}	1.232

μ/mm^{-1}	2.865
Formula Weight	1907.63
Colour	clear colourless
Shape	block-shaped
Size/mm^3	0.34×0.20×0.20
T/K	120.00(10)
Crystal System	monoclinic
Space Group	$P2_1/n$
a/Å	16.6265(3)
b/Å	13.6470(2)
c/Å	23.1149(5)
α/°	90
β/°	101.234(2)
γ/°	90
V/Å^3	5144.32(17)
Z	2
Z'	0.5
Wavelength/Å	1.54184
Radiation type	Cu K□
Θ_{min}/°	3.634
Θ_{max}/°	76.271
Measured Refl's.	128172
Indep't Refl's	10705
Refl's I≥2 σ(I)	9197
R_{int}	0.0885
Parameters	695
Restraints	602
Largest Peak	0.421

Deepest Hole	-0.456	wR_2	0.2162
GooF	1.054	R_1 (all data)	0.0868
wR_2 (all data)	0.2236	R_1	0.0785

Structure Quality Indicators

**Reflections:**	d min (Cu\a) 2Θ=152.5° 0.79	I/σ(I) 30.9	Rint m=12.19 8.85%	Full 135.4° 99% to 152.5° 100
**Refinement:**	Shift 0.000	Max Peak 0.4	Min Peak -0.5	GooF 1.054

A clear colourless block-shaped-shaped crystal with dimensions 0.34 × 0.20 × 0.20 mm^3 was mounted on a MITIGEN holder in Paratone oil. Data were collected using a Rigaku Oxford Diffraction SuperNova diffractometer equipped with an Oxford Cryosystems Cryostream 700+ low-temperature device operating at T = 120.00(10) K.

Data were measured using ω scans with Cu K_α radiation. The diffraction pattern was indexed and the total number of runs and images was based on the strategy calculation from the program CrysAlisPro 1.171.42.81a.[51] The maximum resolution that was achieved was Θ = 76.271° (0.79 Å).

The unit cell was refined using CrysAlisPro 1.171.42.81a[51] on 34954 reflections, 27% of the observed reflections.

Data reduction, scaling and absorption corrections were performed using CrysAlisPro 1.171.42.81a.[51] The final completeness is 100.00% out to 76.271° in Θ. A multi-scan absorption correction was performed using CrysAlisPro 1.171.42.81a[51] Spherical absorption correction using equivalent radius and absorption coefficient. Empirical absorption correction using spherical harmonics, implemented in SCALE3 ABSPACK scaling algorithm. The absorption coefficient μ of this material is 2.865 mm^{-1} at this wavelength (λ = 1.54184Å) and the minimum and maximum transmissions are 0.960 and 0.960.

The structure was solved and the space group $P2_1/n$ (# 14) determined by the **ShelXT** 2018/2[48] structure solution program using using dual methods and refined by full matrix least squares minimisation on $\boldsymbol{F}^2$ using version 2018/3 of **ShelXL** 2018/3.[50] All non-hydrogen atoms were refined anisotropically. Hydrogen atom positions were calculated geometrically and refined using the riding model. Hydrogen atom positions were calculated geometrically and refined using the riding model.

_refine_special_details: Three of the five THF ligands in the asymmetric unit, and the potassium site to which they are ligated, were modelled as disordered using the Frag DB function of Olex2. Geometric and displacement similarity restraints were used on all THF ligands.

The value of Z' is 0.5. This means that only half of the formula unit is present in the asymmetric unit, with the other half consisting of symmetry equivalent atoms.

16. NMR Spectra of Isolated Compounds

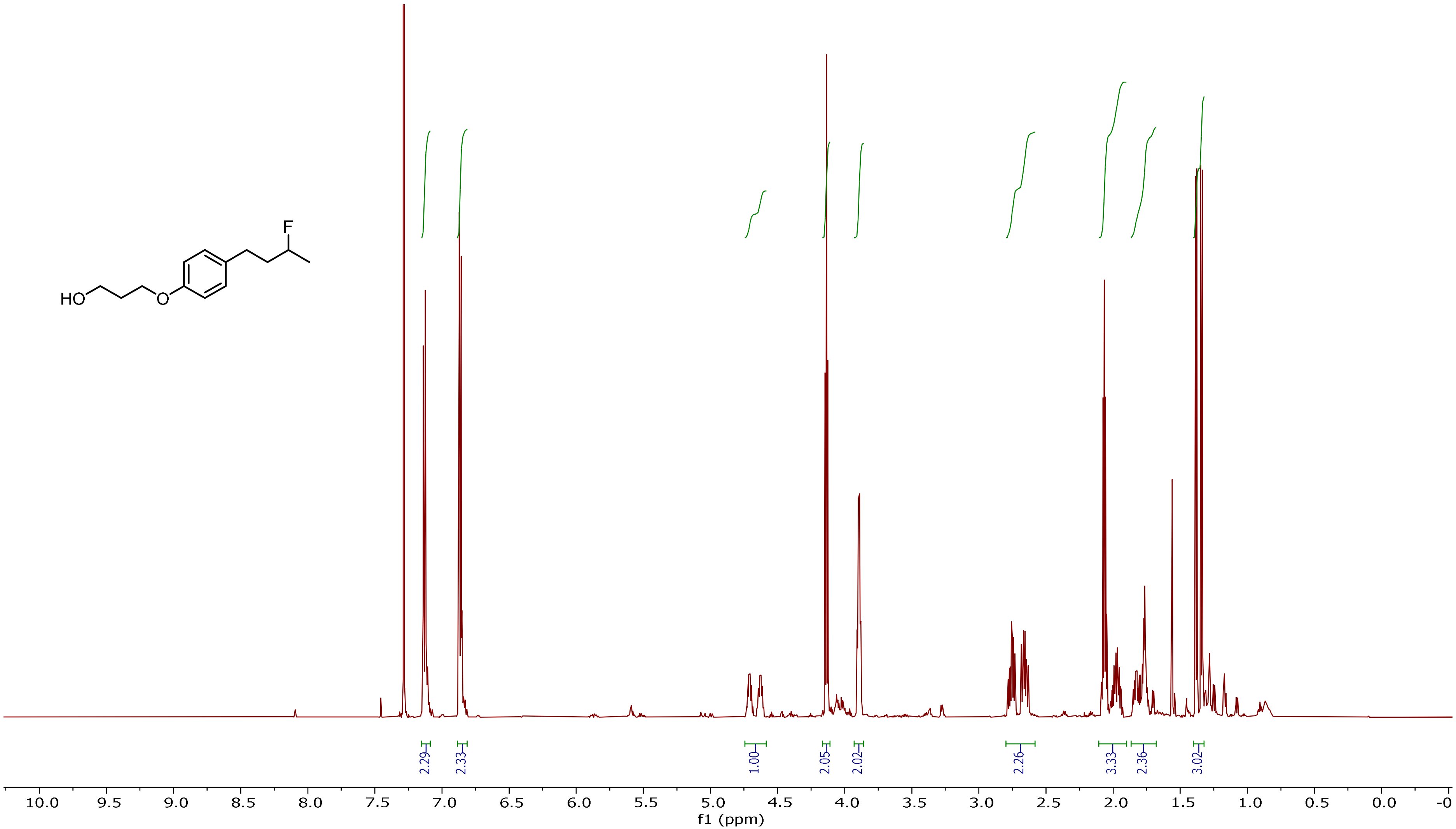

^{1}H NMR (601 MHz, $CDCl_3$) spectrum of 2-fluoro-4-(4-(3-hydroxypropoxy)phenyl)butane.

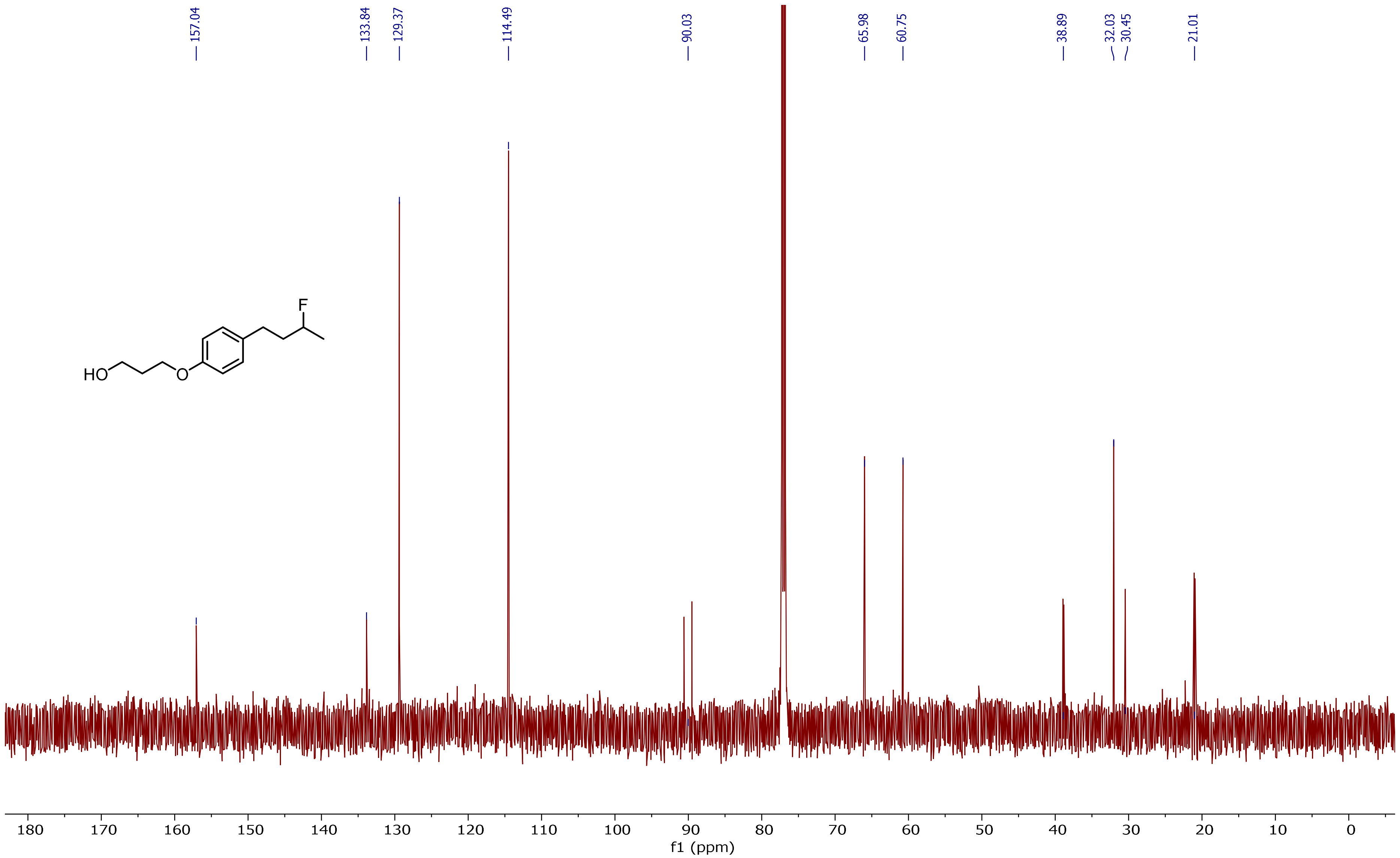

^{13}C NMR (151 MHz, $CDCl_3$) spectrum of 2-fluoro-4-(4-(3-hydroxypropoxy)phenyl)butane.

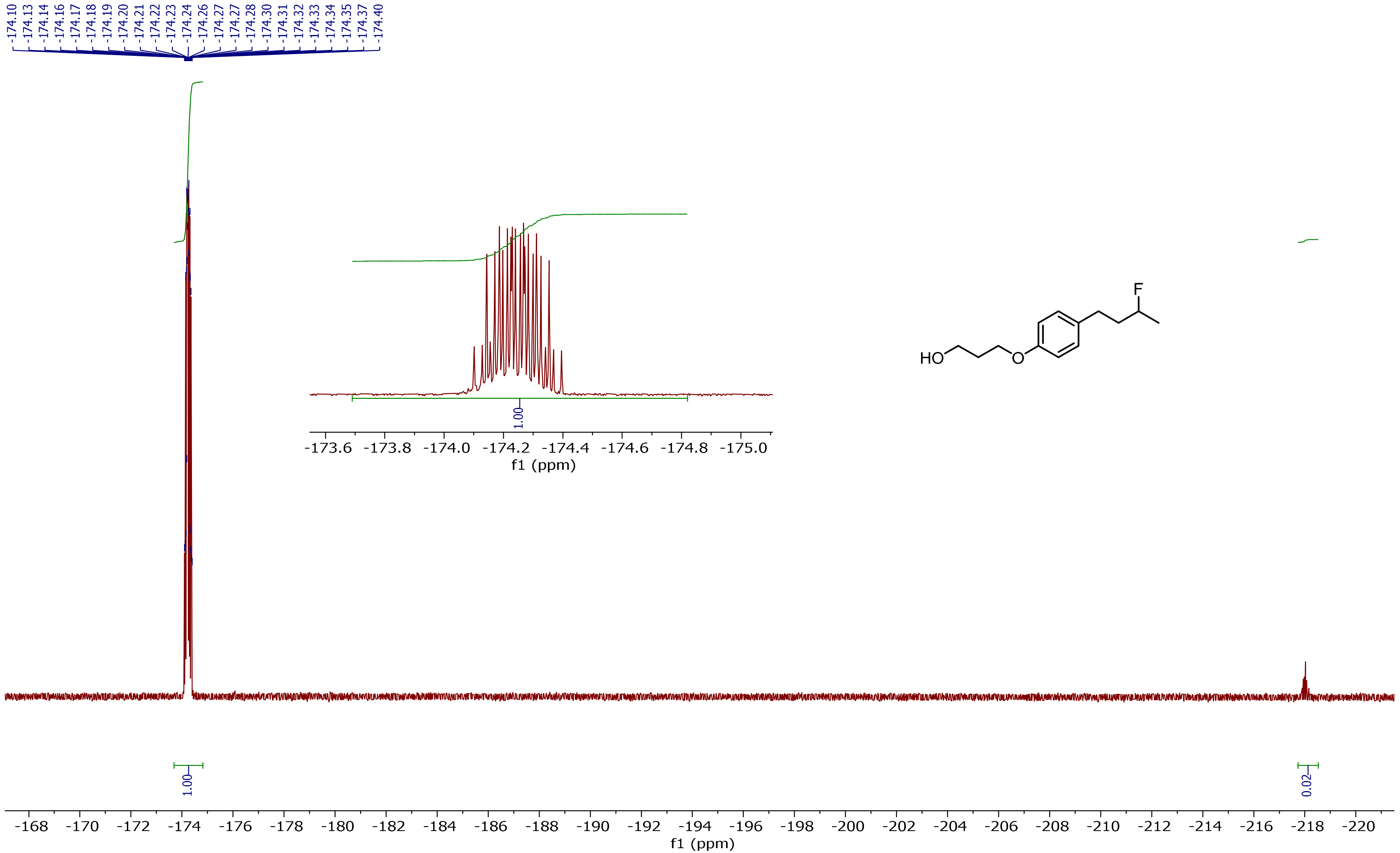

^{19}F NMR (565 MHz, $CDCl_3$) spectrum of 2-fluoro-4-(4-(3-hydroxypropoxy)phenyl)butane.

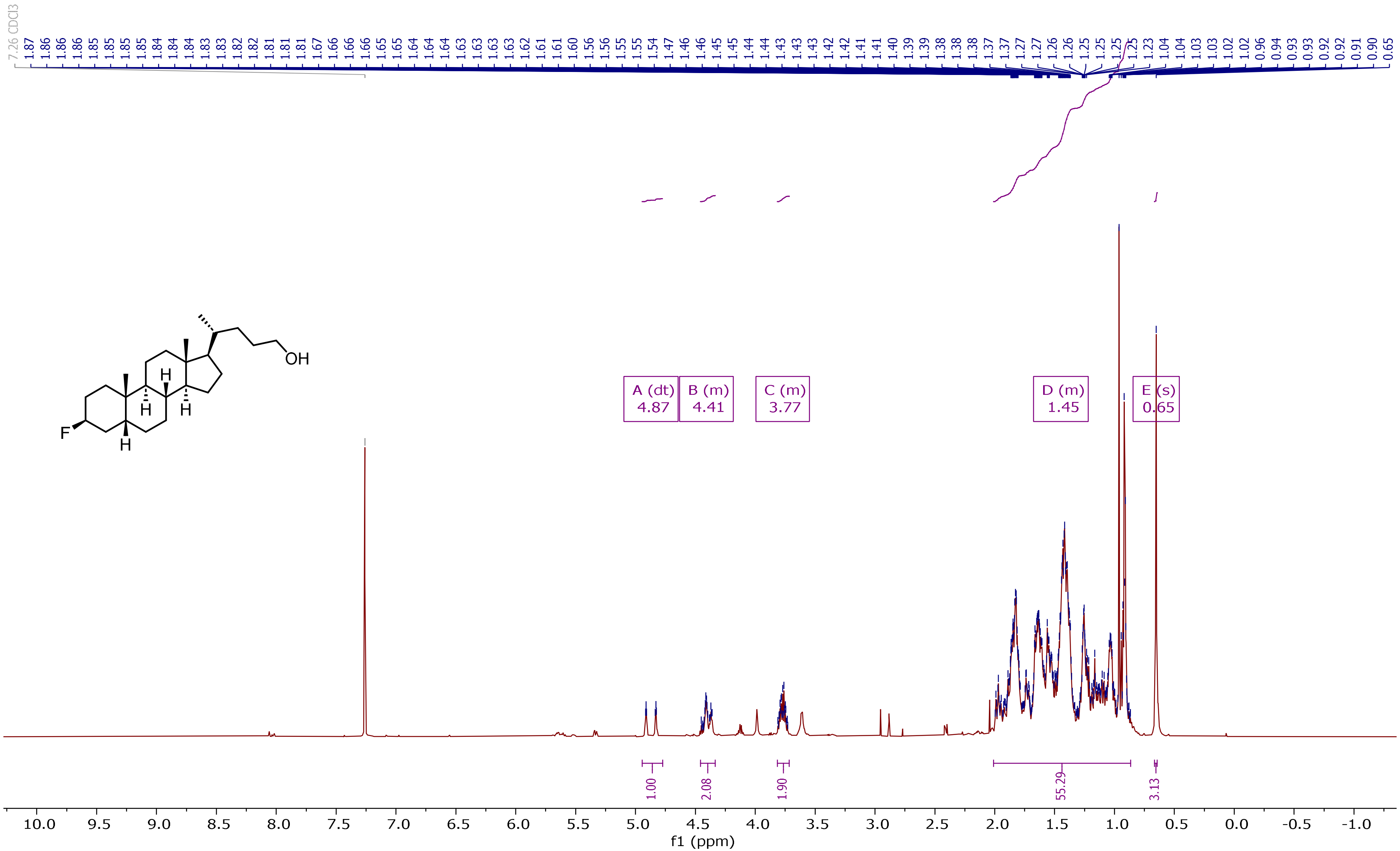

^{1}H NMR (601 MHz, $CDCl_3$) spectrum of 3β-fluoro-24-hydroxy-5β-cholane.

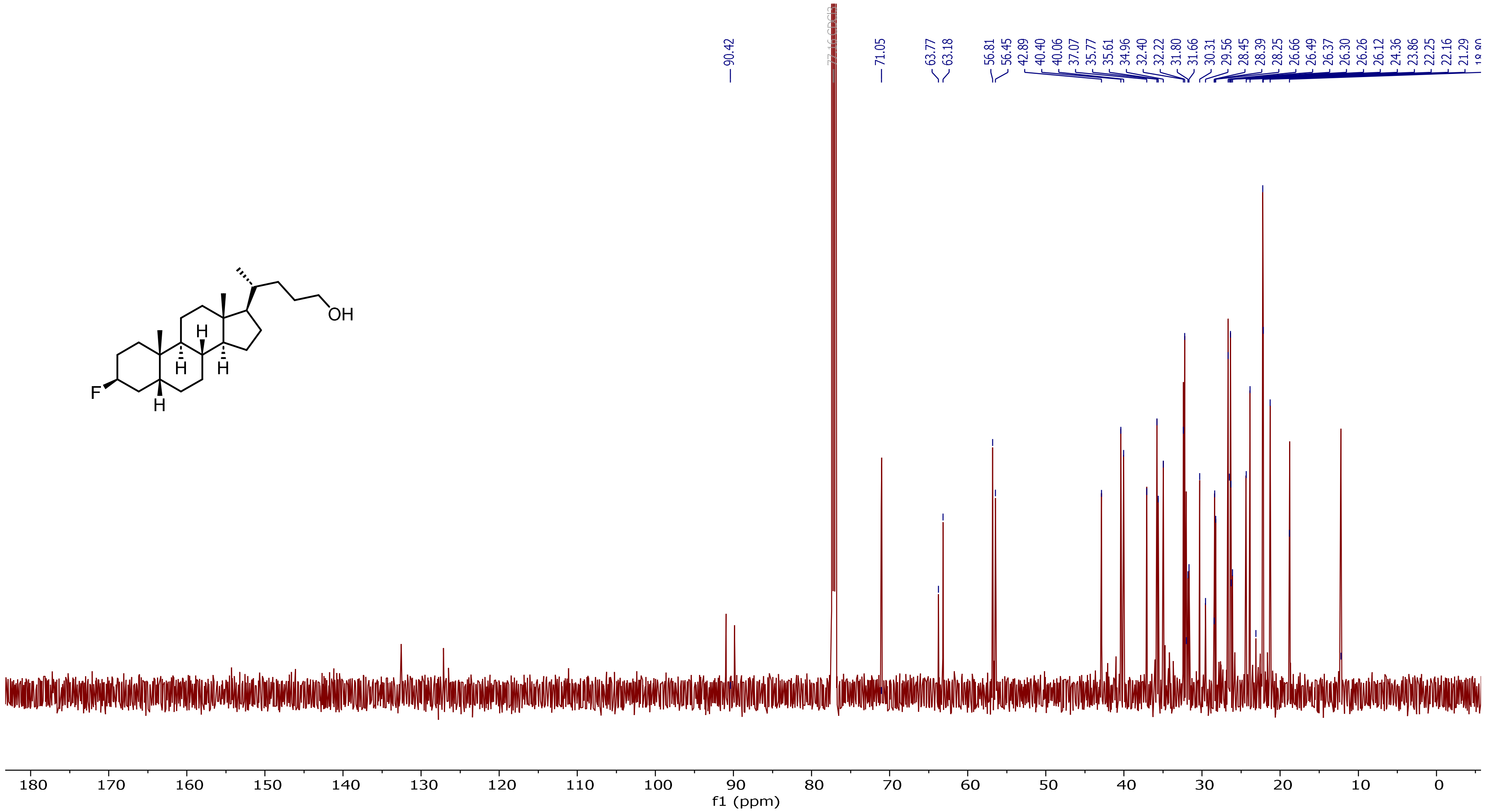

^{13}C NMR (151 MHz, $CDCl_3$) spectrum of 3β-fluoro-24-hydroxy-5β-cholane.

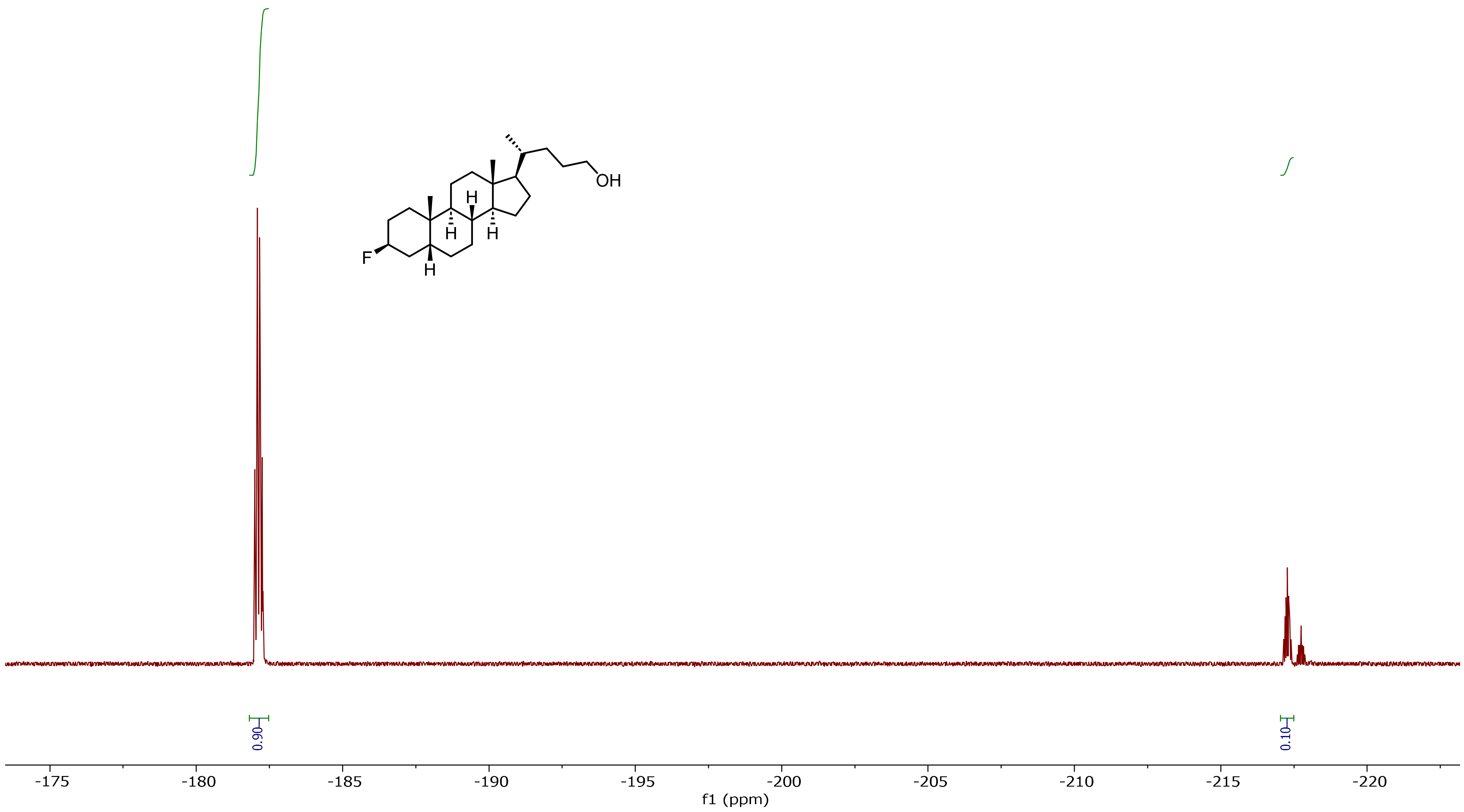

^{19}F NMR (565 MHz, $CDCl_3$) spectrum of 3β-fluoro-24-hydroxy-5β-cholane and primary fluorination product.

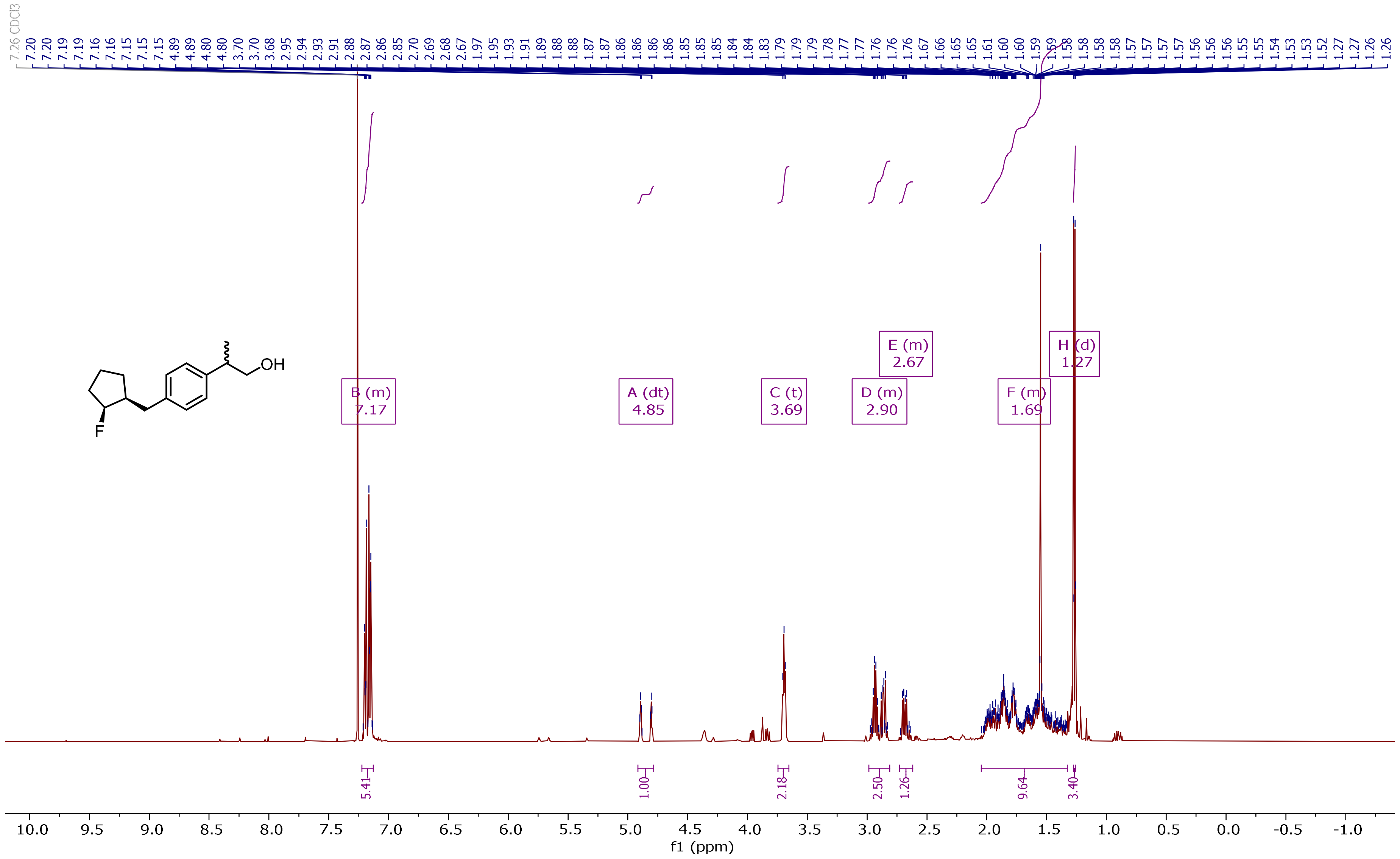

^{1}H NMR (601 MHz, $CDCl_3$) spectrum of (±)-1-*cis*-fluoro-2-(4-(1-hydroxypropan-2-yl)benzyl)cyclopentane.

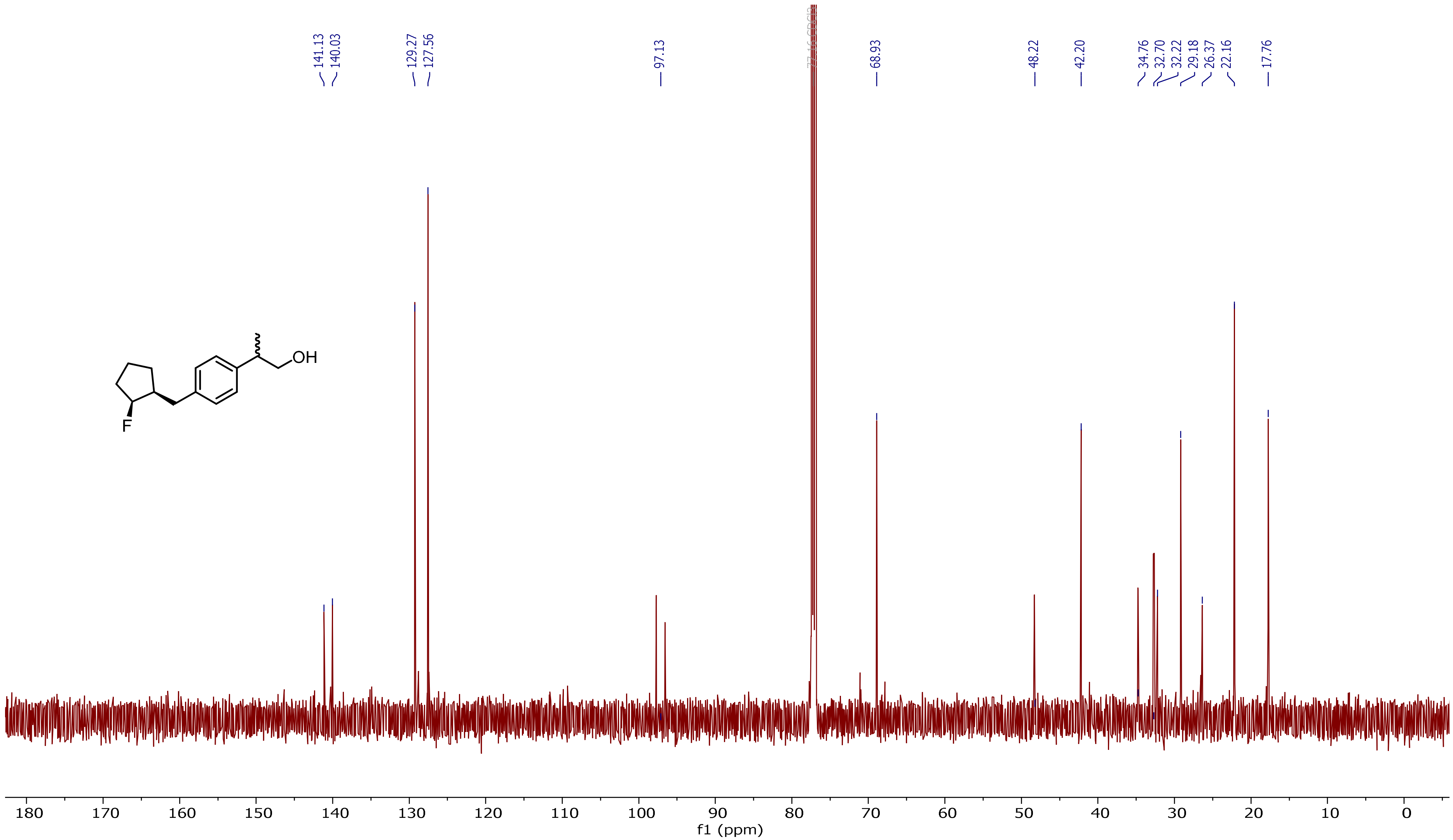

^{13}C NMR (151 MHz, $CDCl_3$) spectrum of (±)-1-*cis*-fluoro-2-(4-(1-hydroxypropan-2-yl)benzyl)cyclopentane.

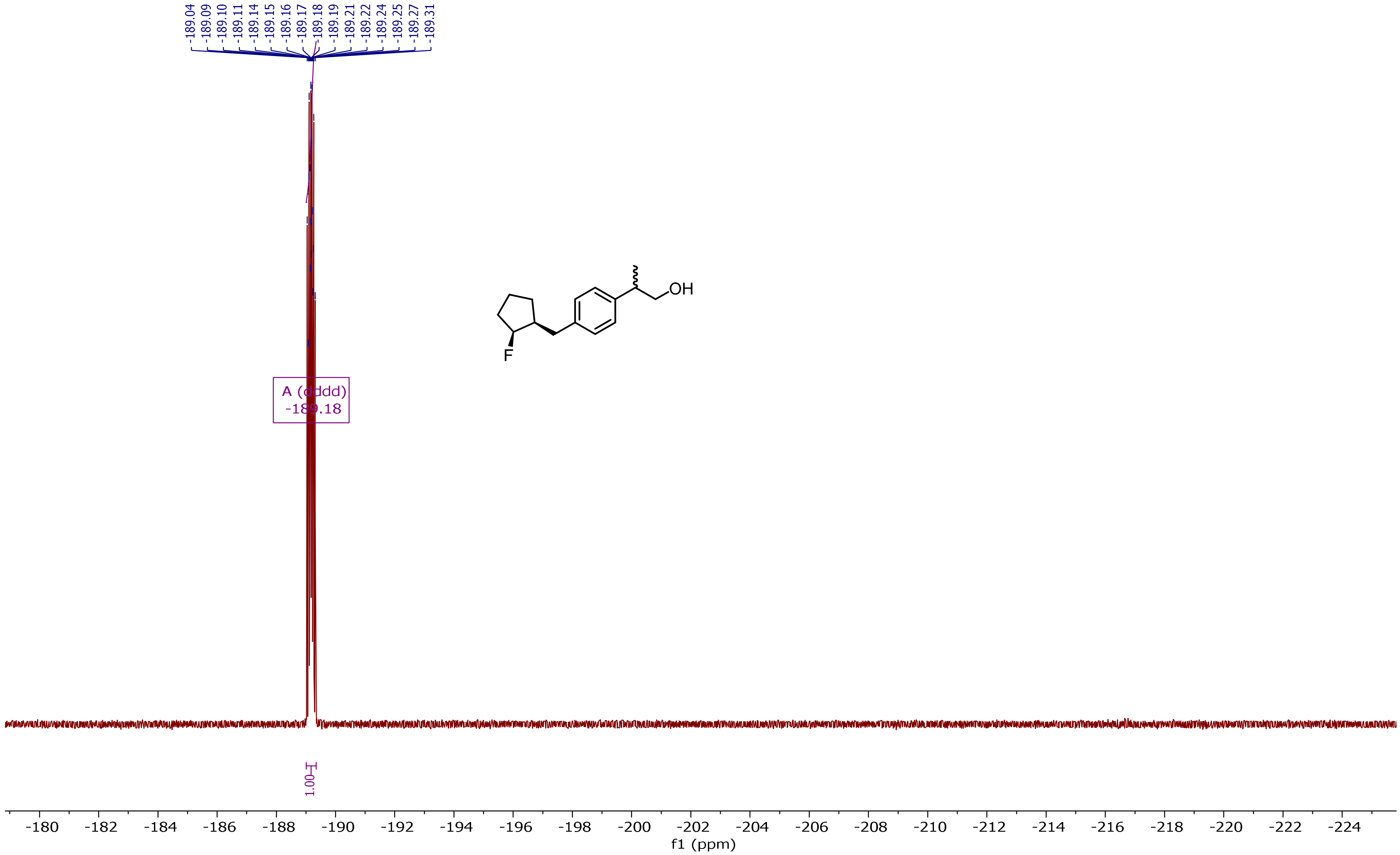

^{19}F NMR (565 MHz, $CDCl_3$) spectrum of (±)-1-*cis*-fluoro-2-(4-(1-hydroxypropan-2-yl)benzyl)cyclopentane.

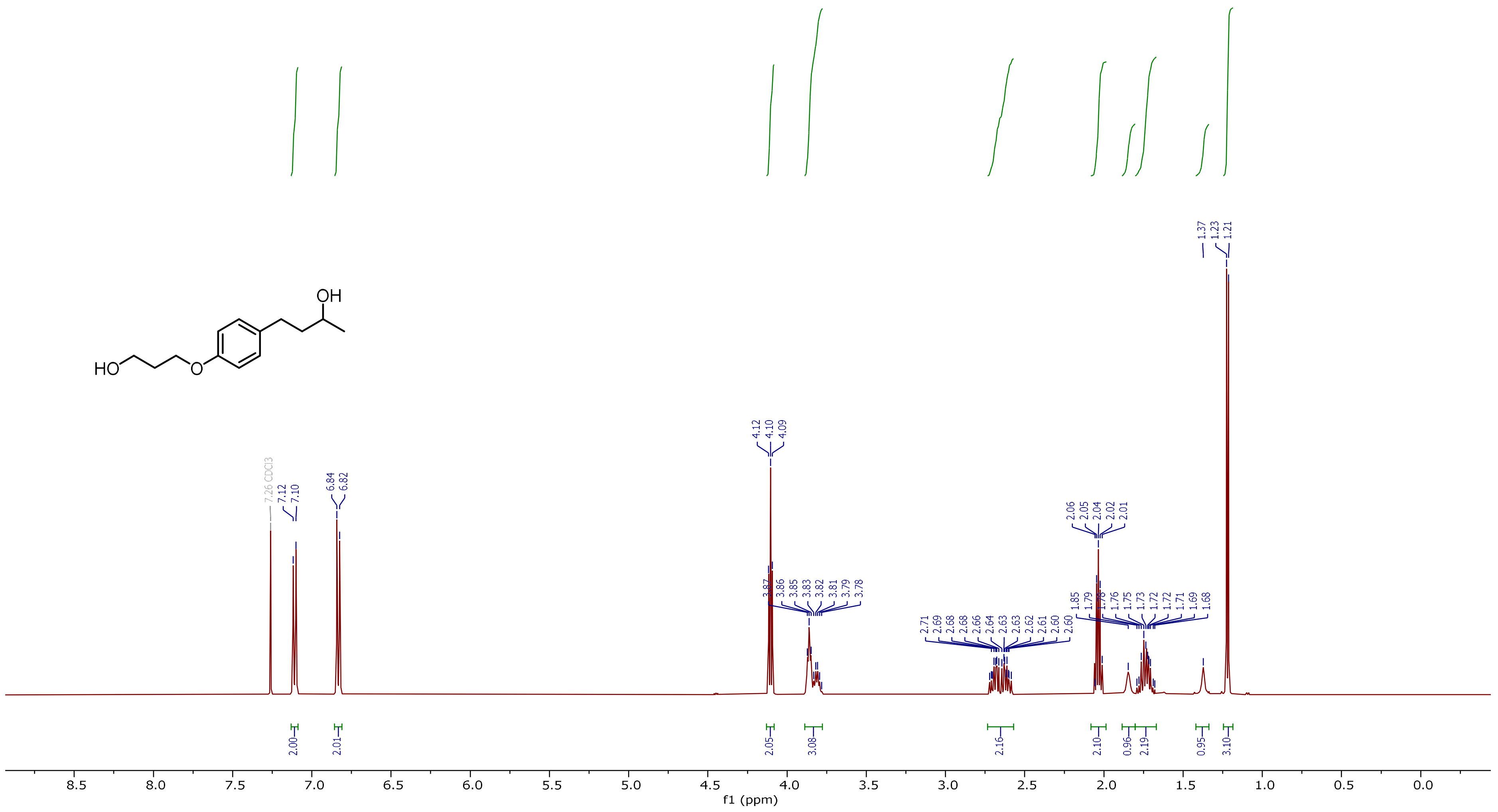

^1H NMR (500 MHz, $CDCl_3$) spectrum of 4-(4-(3-hydroxypropoxy)phenyl)butan-2-ol.

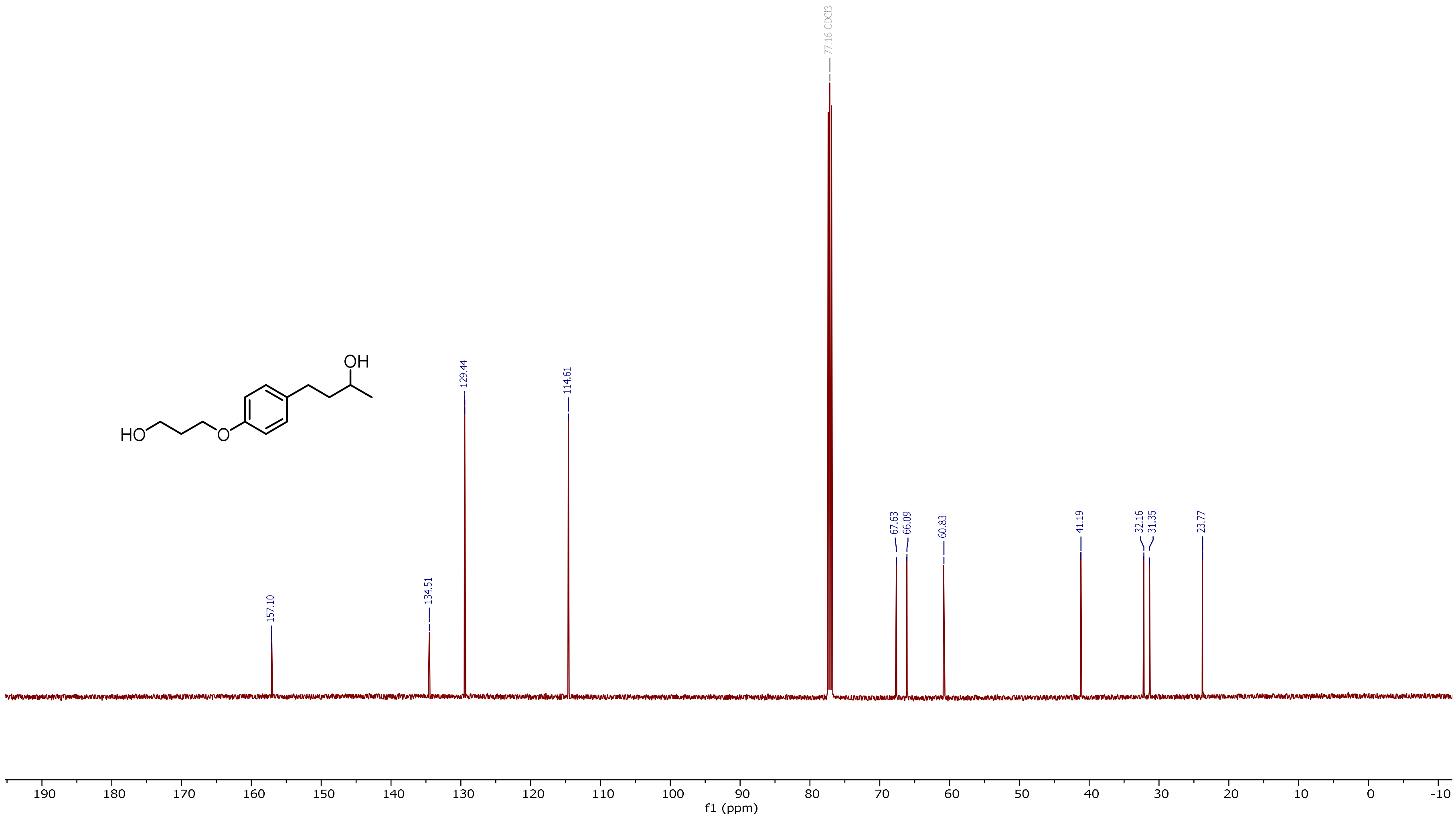

^{13}C NMR (126 MHz, $CDCl_3$) spectrum of 4-(4-(3-hydroxypropoxy)phenyl)butan-2-ol.

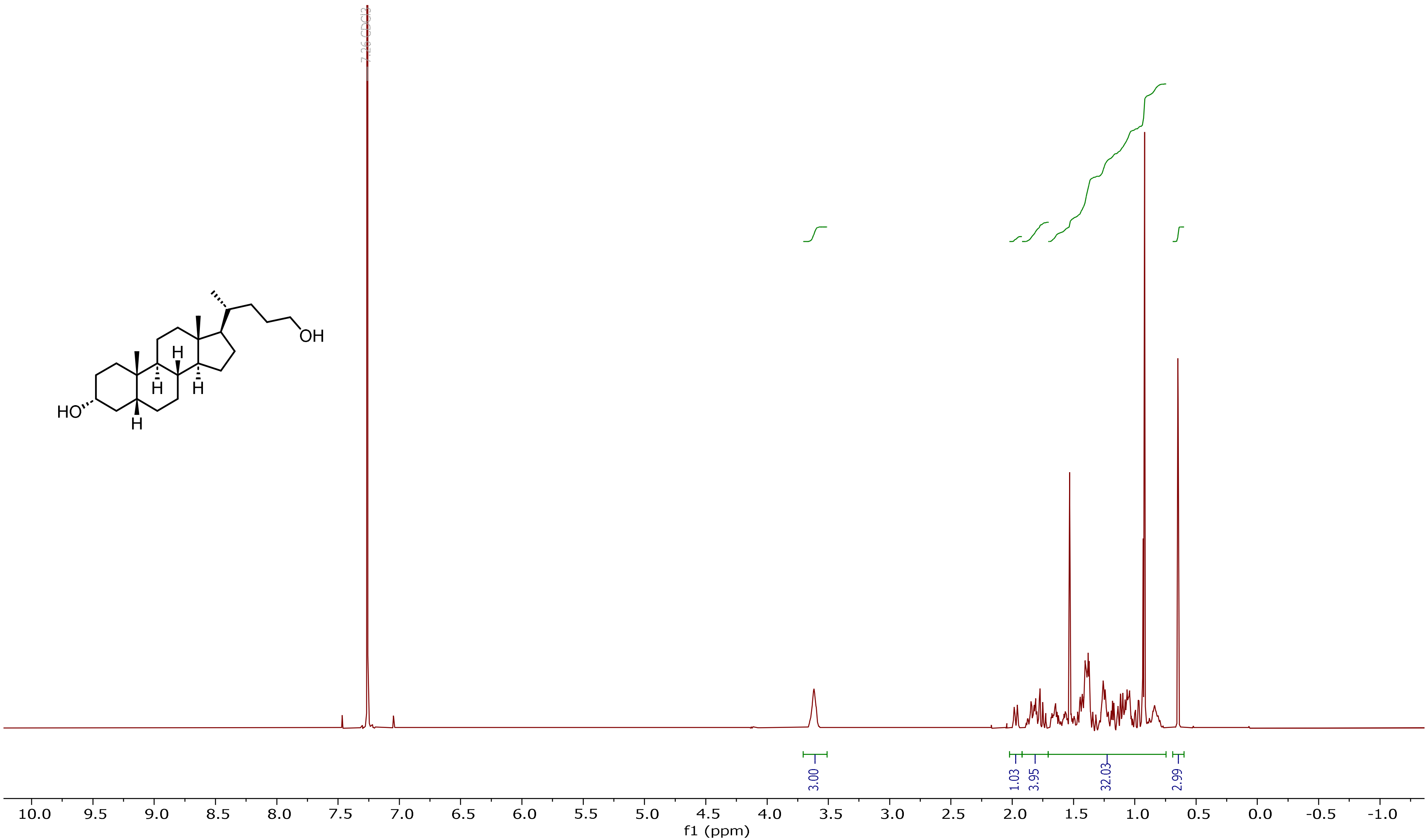

^{1}H NMR (500 MHz, $CDCl_3$) spectrum of 5β-cholane-3α,24-diol.

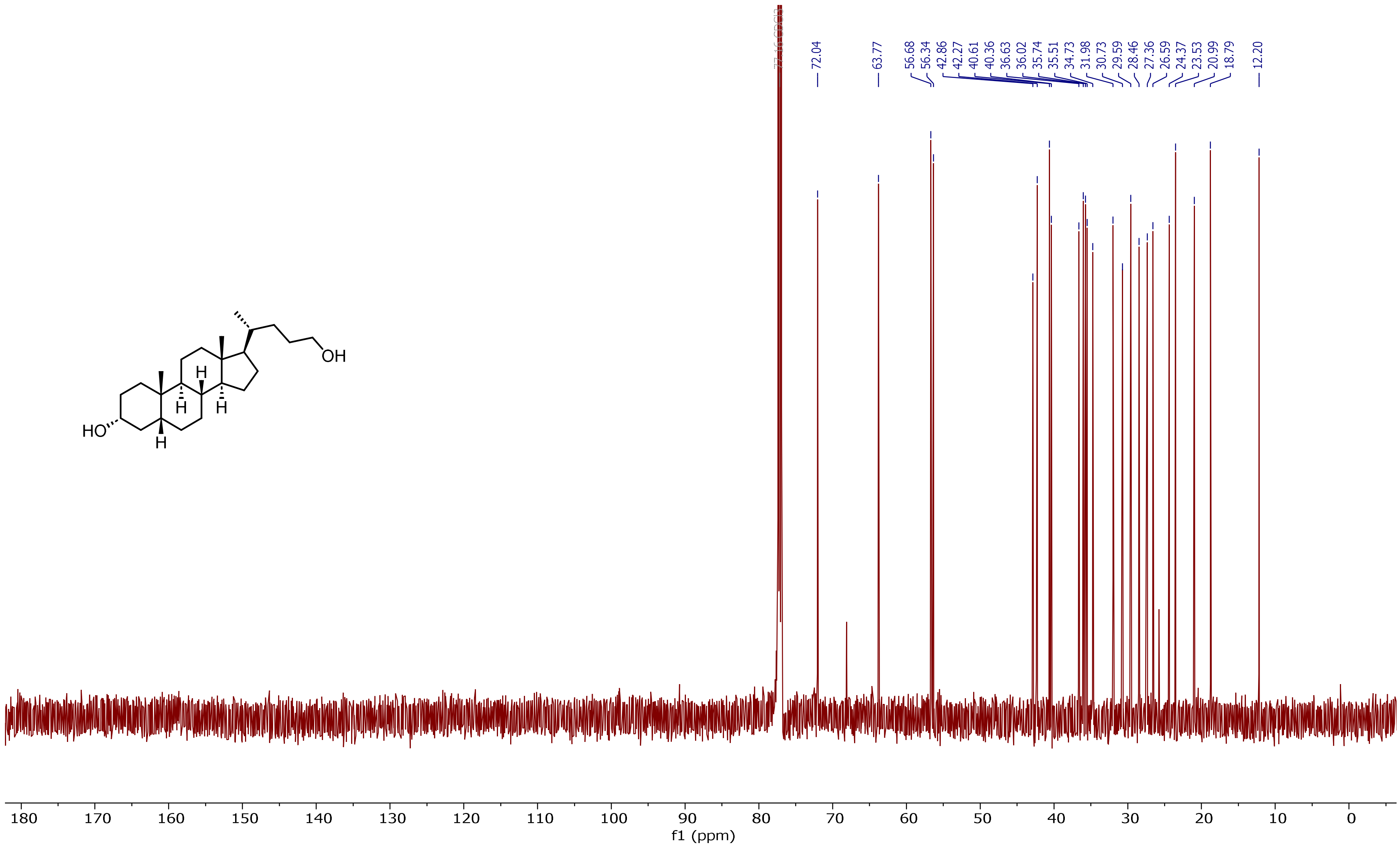

^{13}C NMR (151 MHz, $CDCl_3$) spectrum of 5β-cholane-3α,24-diol.

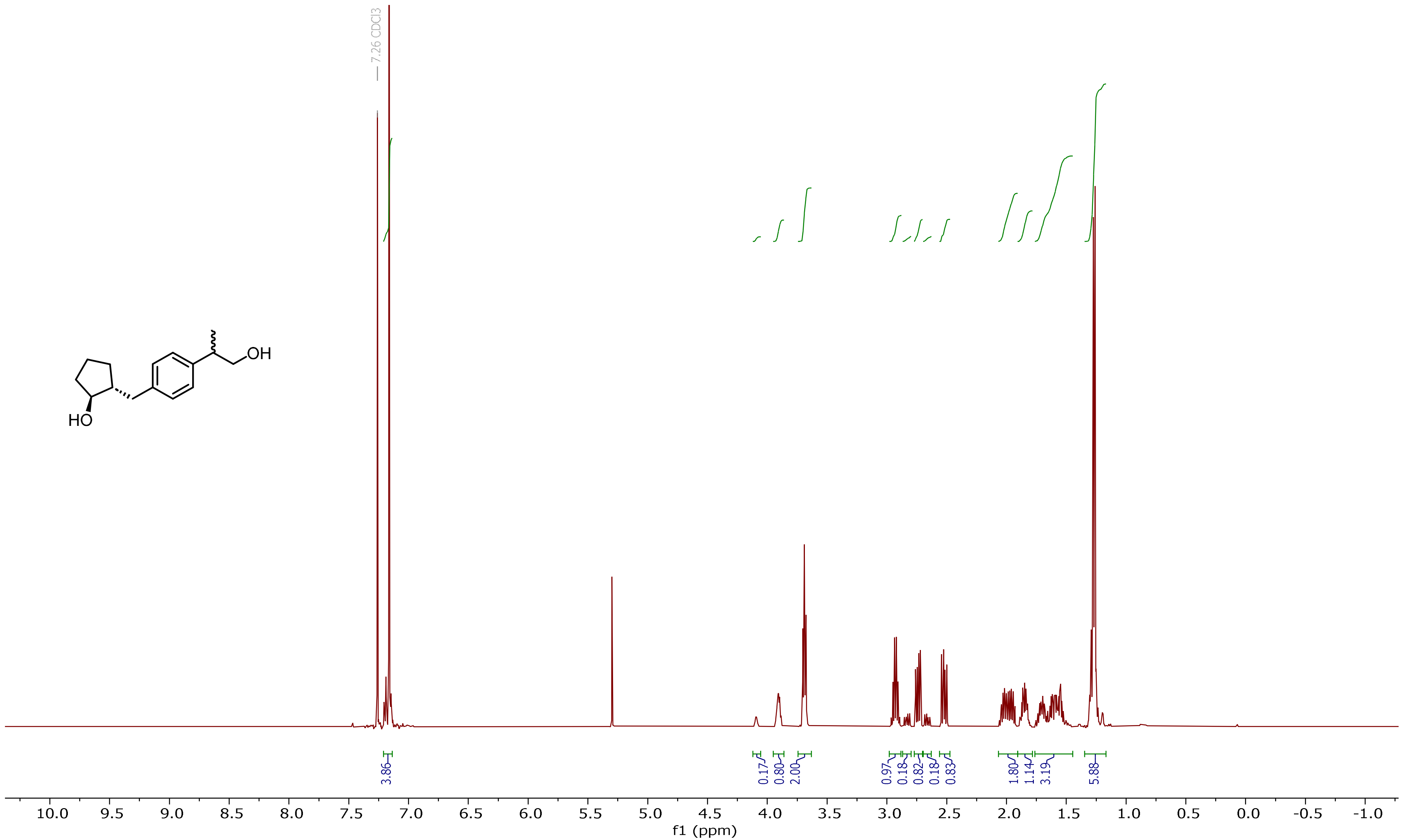

^{1}H NMR (500 MHz, $CDCl_3$) spectrum of 2-(4-(1-hydroxypropan-2-yl)benzyl)cyclopentan-1-ol.

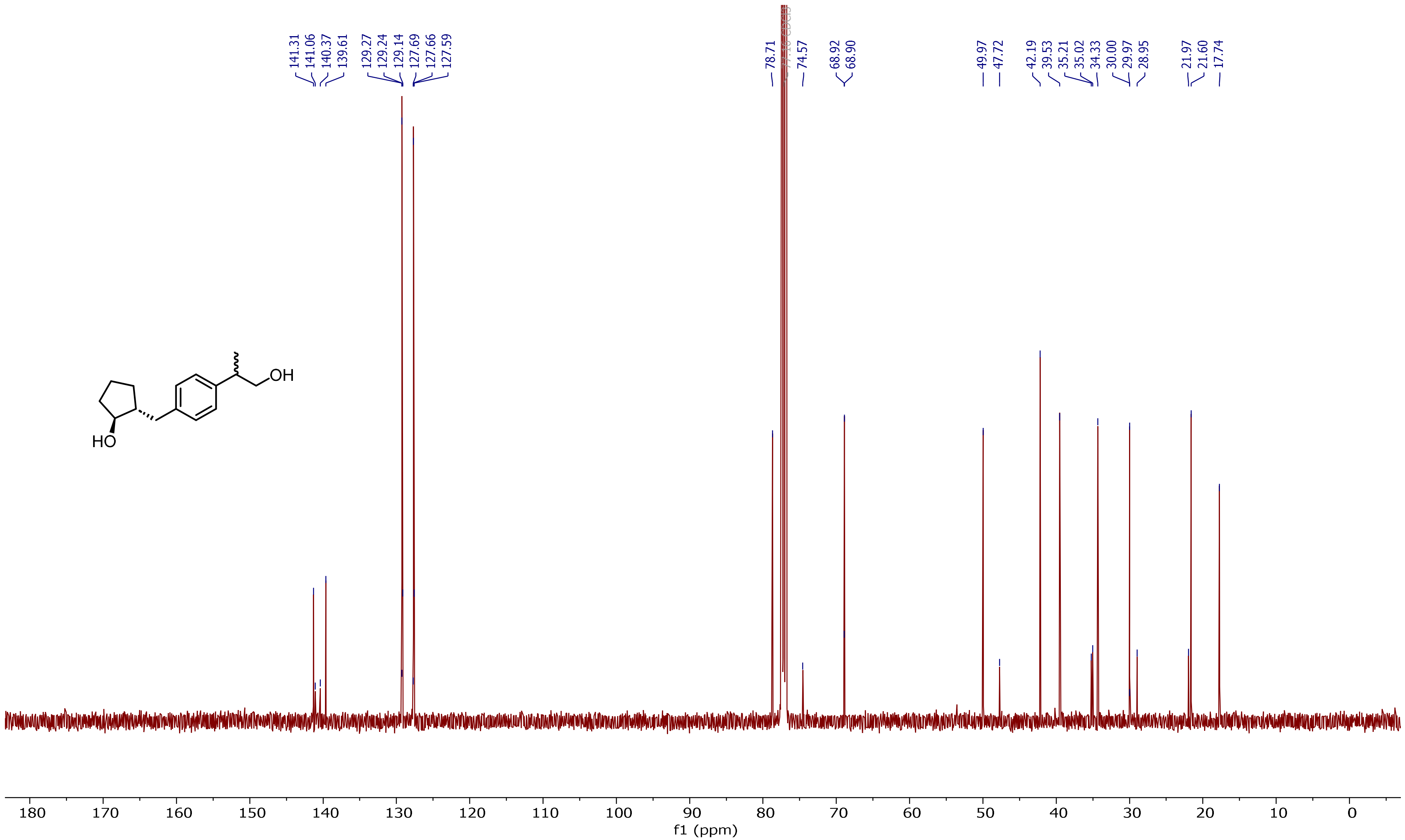

^{13}C NMR (151 MHz, $CDCl_3$) spectrum of 2-(4-(1-hydroxypropan-2-yl)benzyl)cyclopentan-1-ol.

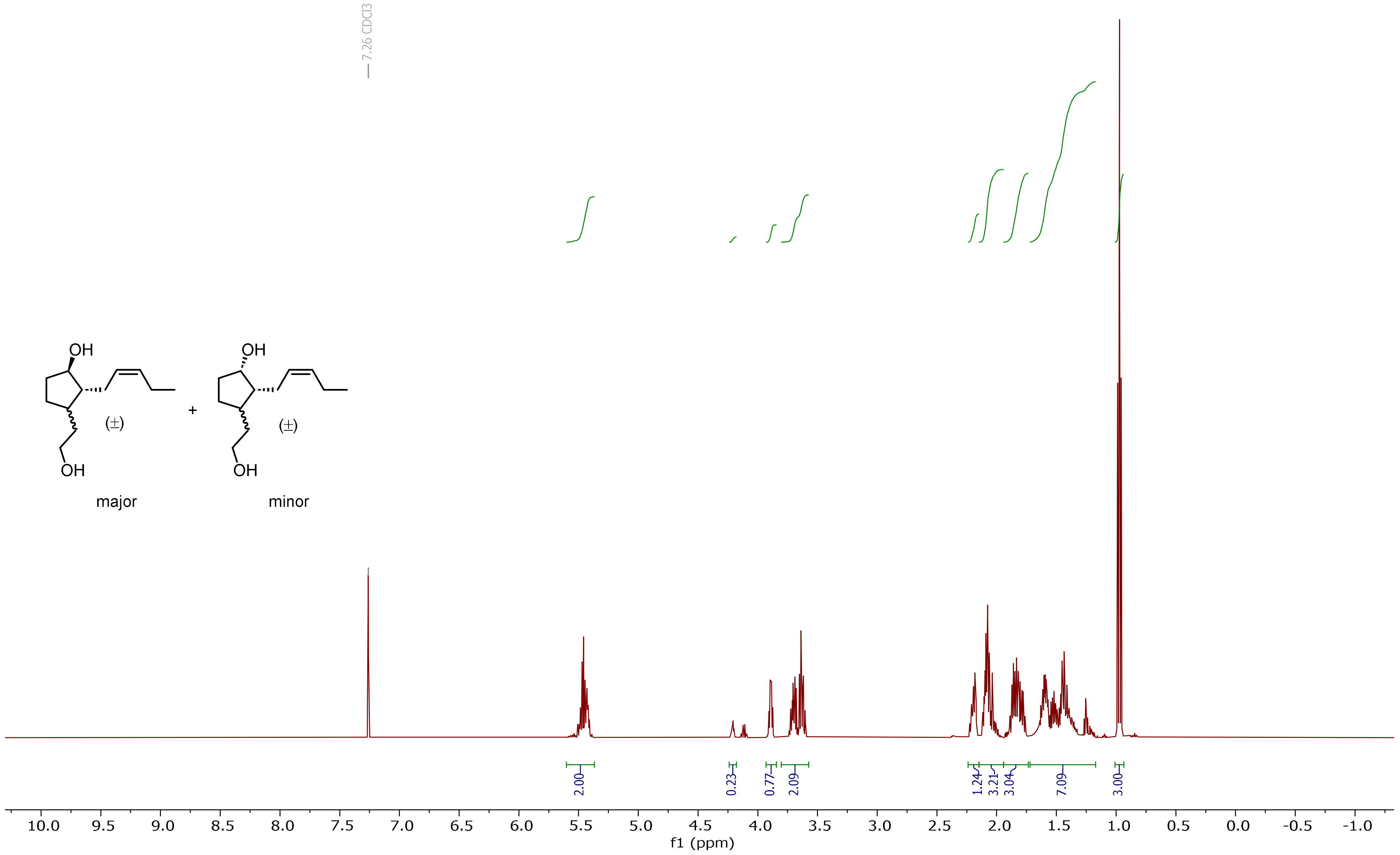

^{1}H NMR (500 MHz, $CDCl_3$) spectrum of (±)-3-(2-hydroxy-ethyl)-2-((*Z*)-pent-2-enyl)-cyclopentanol.

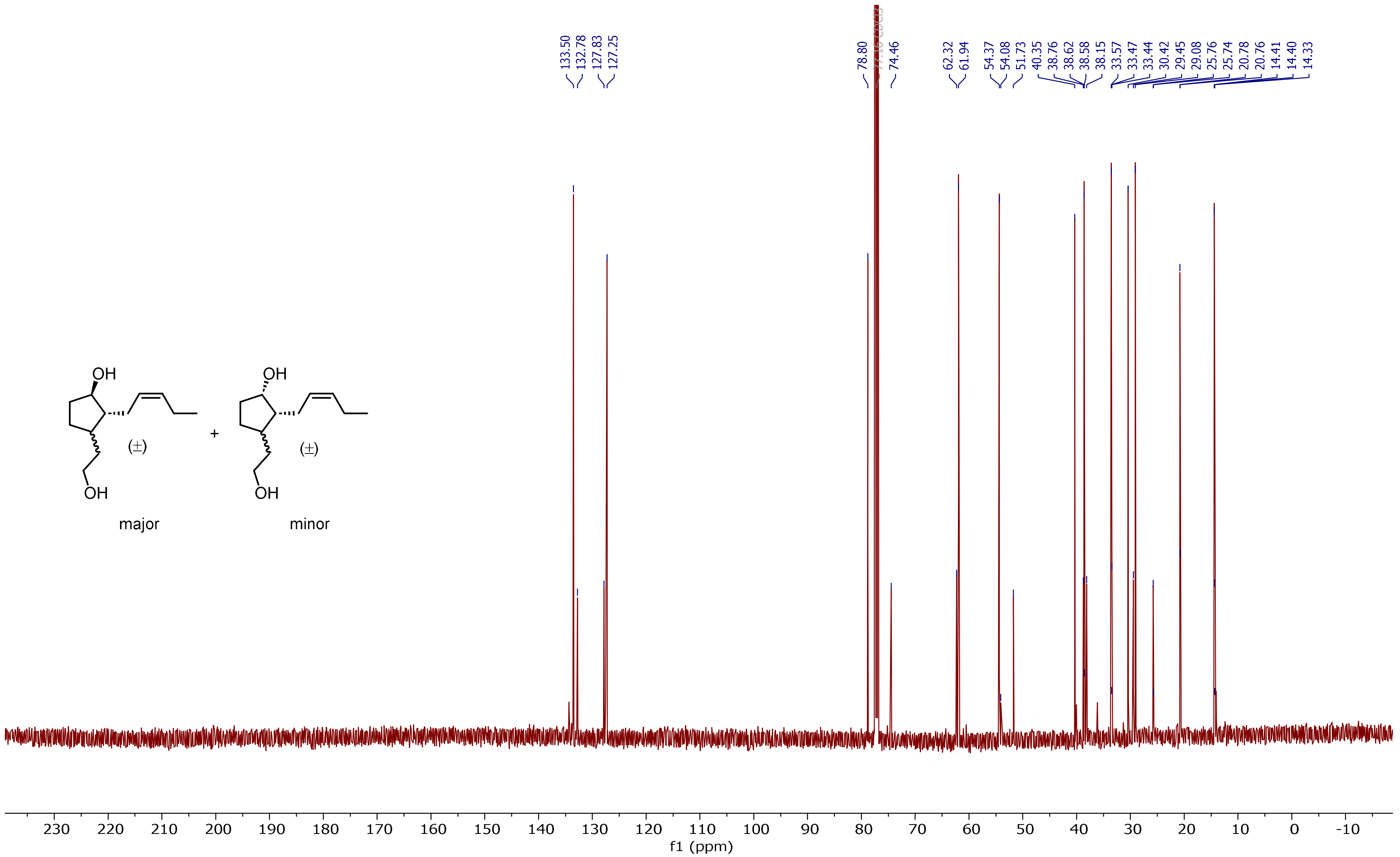

^{13}C NMR (151 MHz, $CDCl_3$) spectrum of (±)-3-(2-hydroxy-ethyl)-2-((*Z*)-pent-2-enyl)-cyclopentanol.

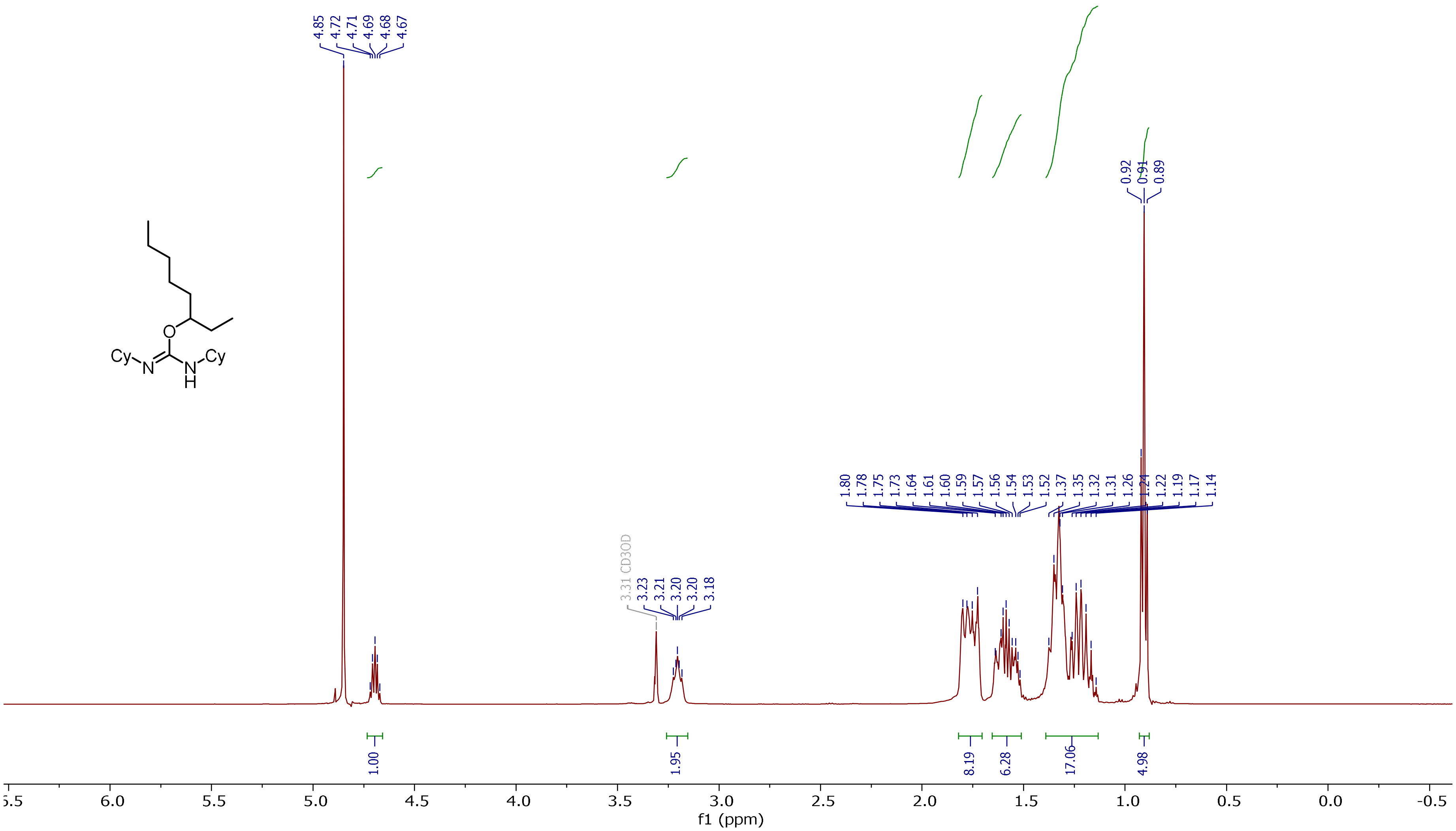

^{1}H NMR (500 MHz, CD_3OD) spectrum of O-(3-octyl)-*N*,*N'*-dicyclohexylisourea.

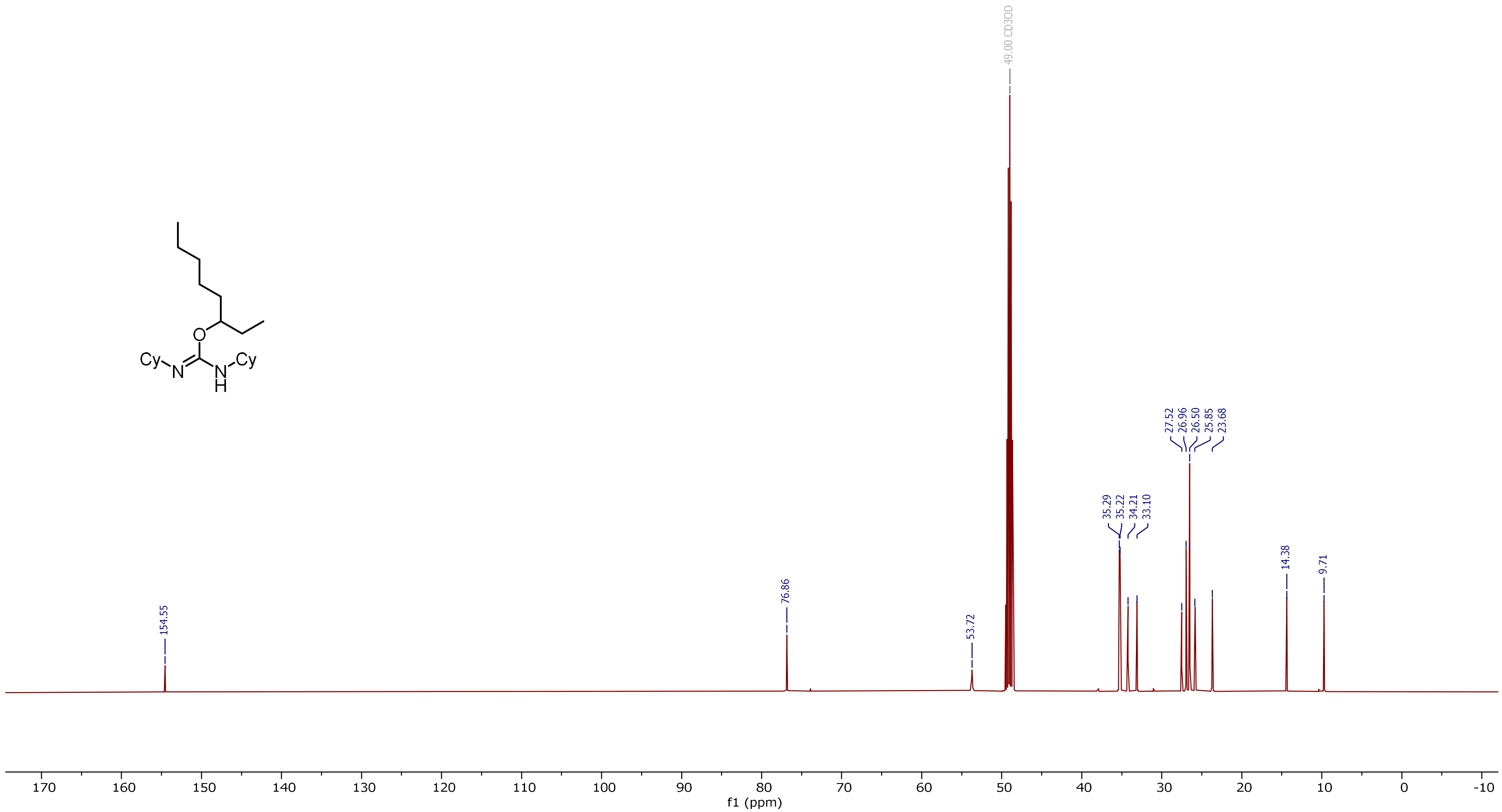

^{13}C NMR (126 MHz, CD_3OD) spectrum of O-(3-octyl)-*N*,*N′*-dicyclohexylisourea.

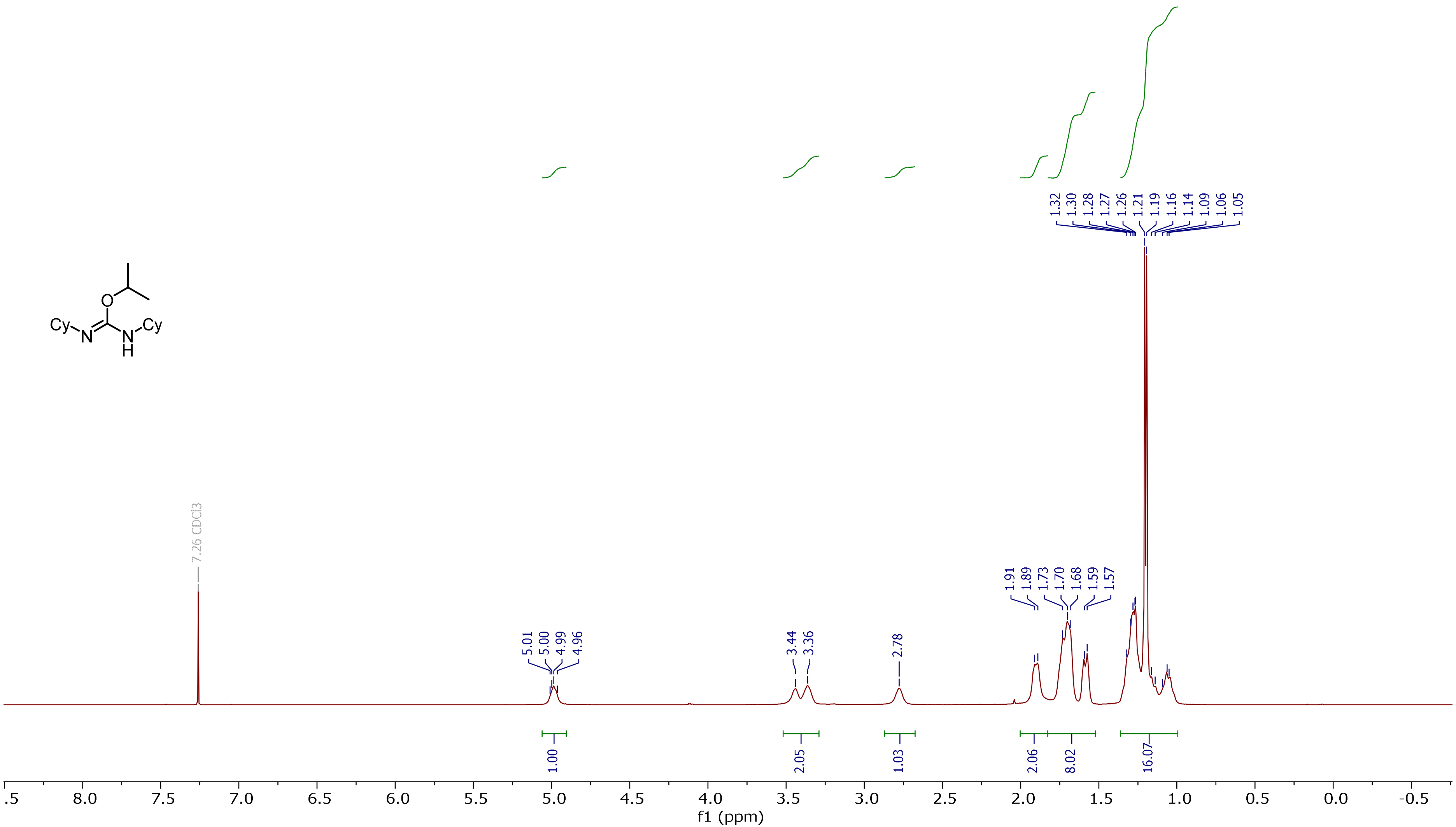

^{1}H NMR (500 MHz, $CDCl_3$) spectrum of O-(isopropyl)-*N*,*N′*-dicyclohexylisourea.

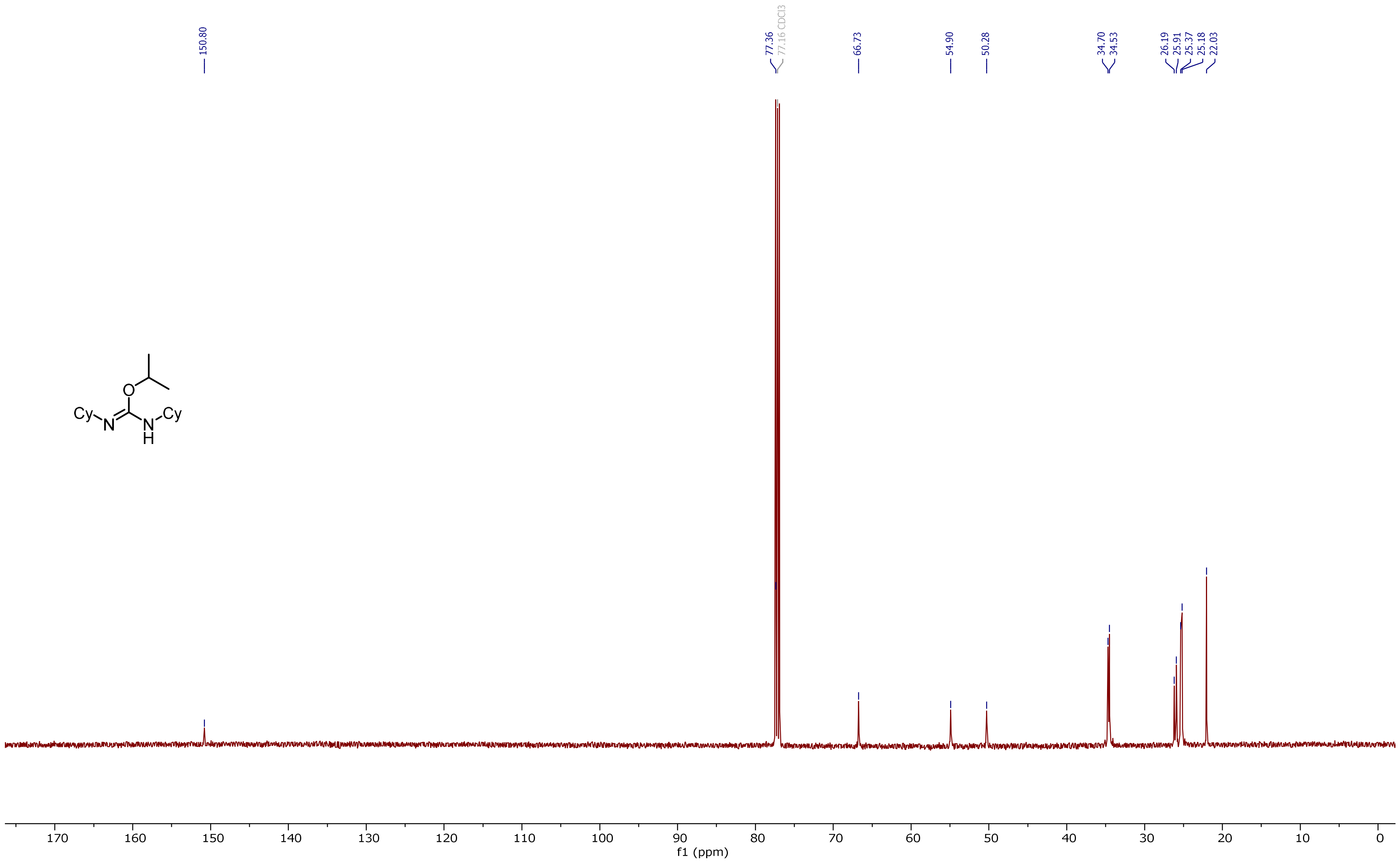

^{13}C NMR (126 MHz, $CDCl_3$) spectrum of O-(isopropyl)-*N*,*N′*-dicyclohexylisourea.

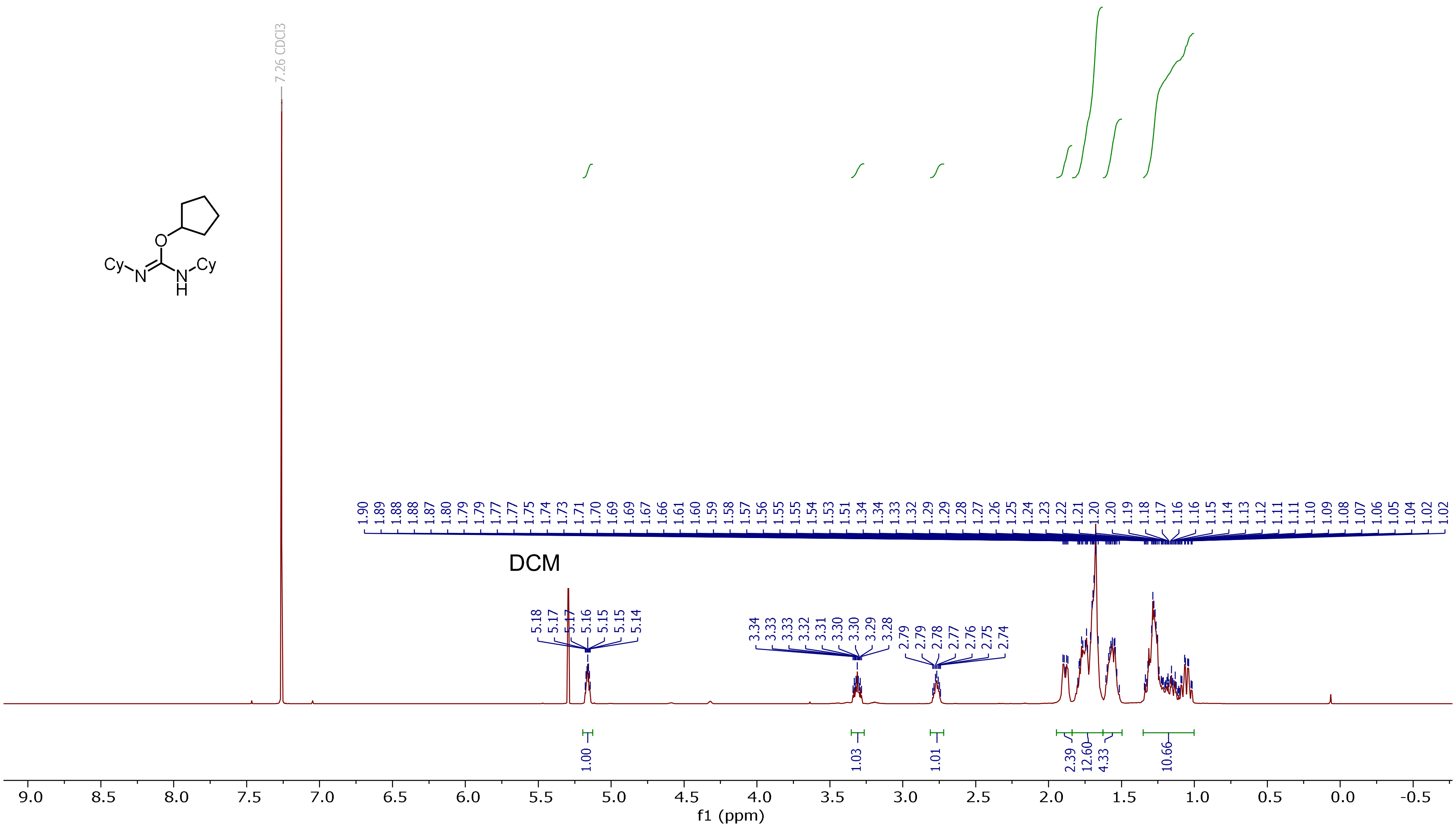

^{1}H NMR (500 MHz, $CDCl_3$) spectrum of O-(cyclopentyl)-*N*,*N'*-dicyclohexylisourea.

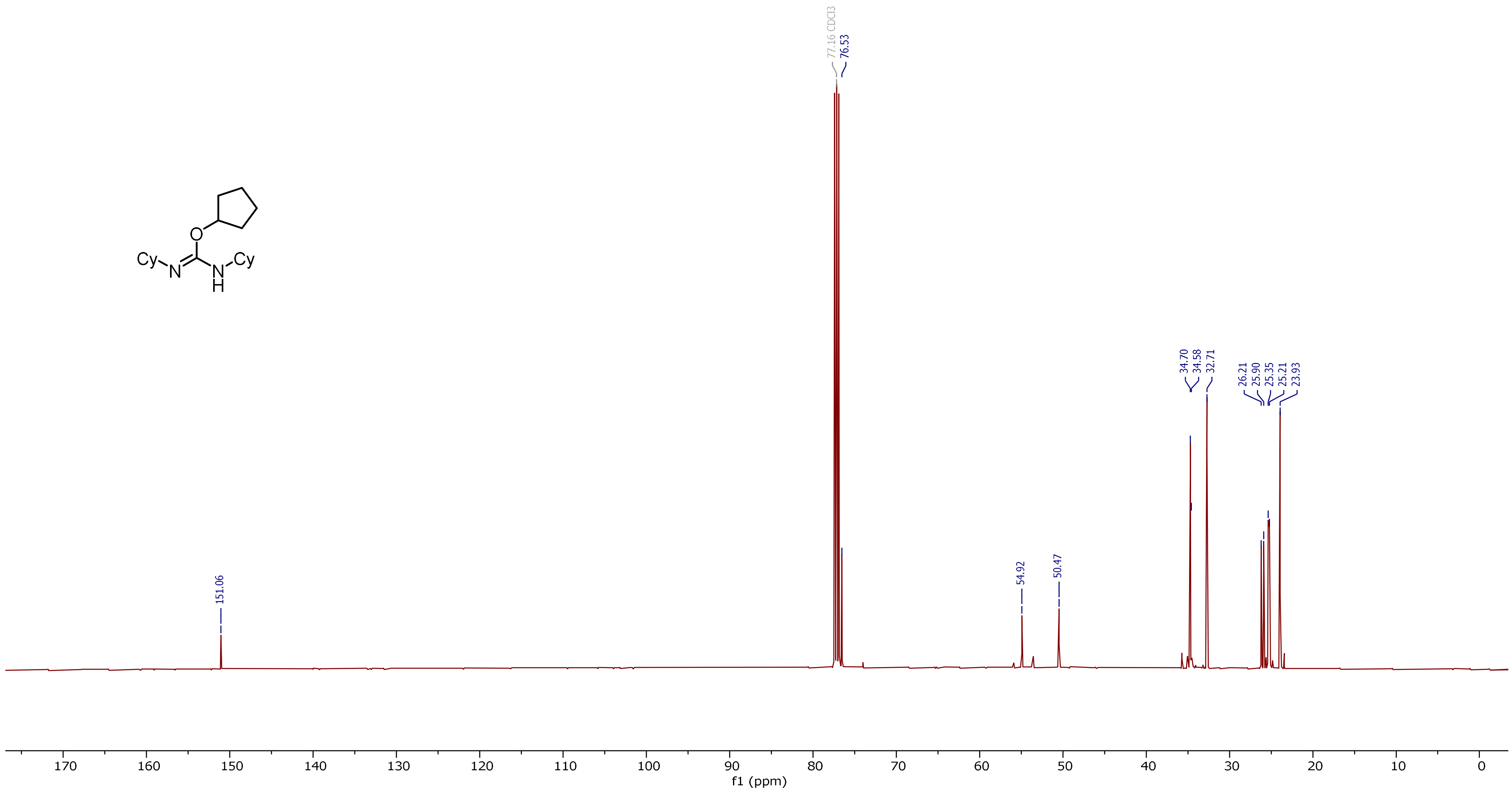

^{13}C NMR (126 MHz, $CDCl_3$) spectrum of O-(cyclopentyl)-*N*,*N'*-dicyclohexylisourea.

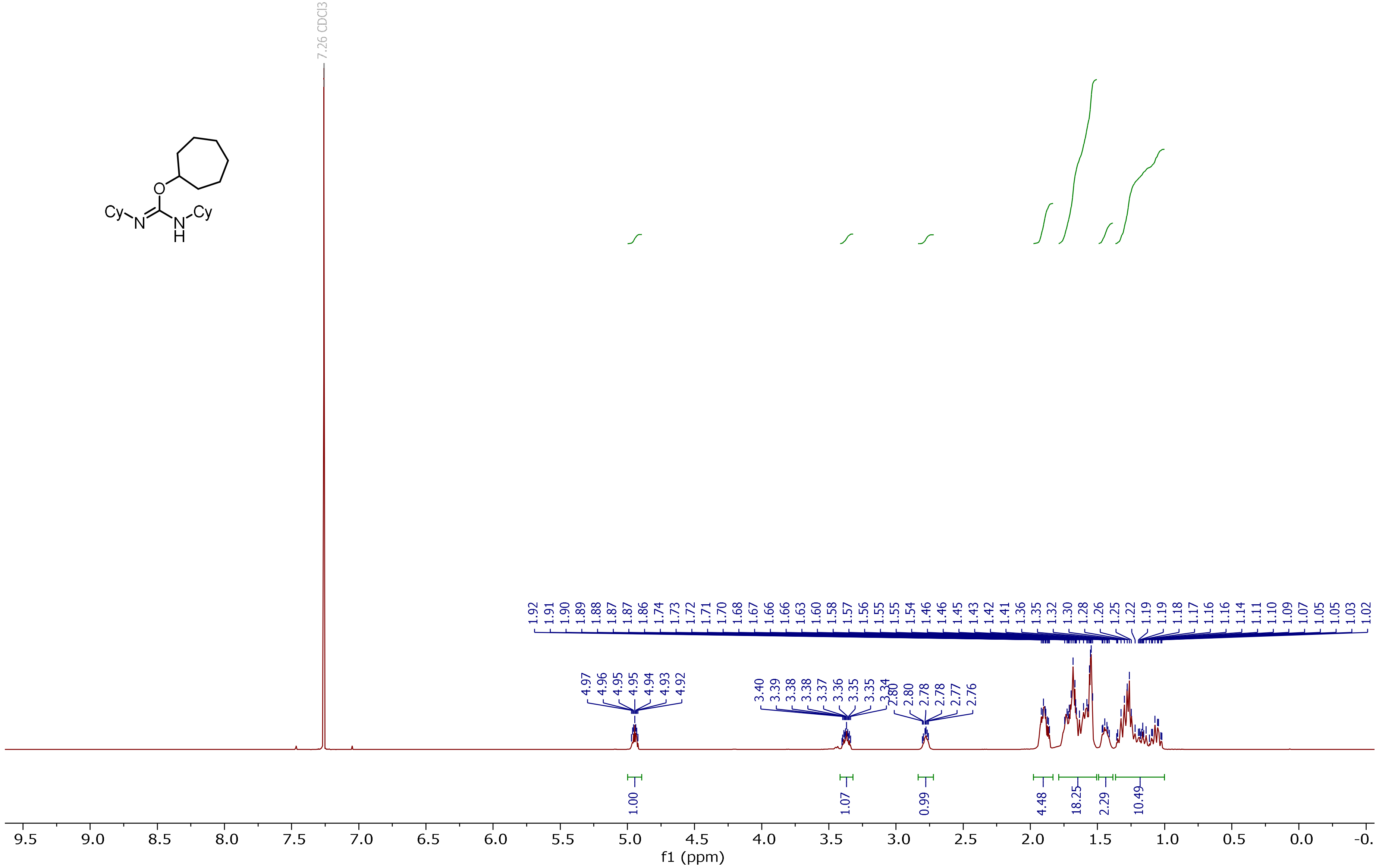

^{1}H NMR (500 MHz, $CDCl_3$) spectrum of O-(cycloheptyl)-*N*,*N'*-dicyclohexylisourea.

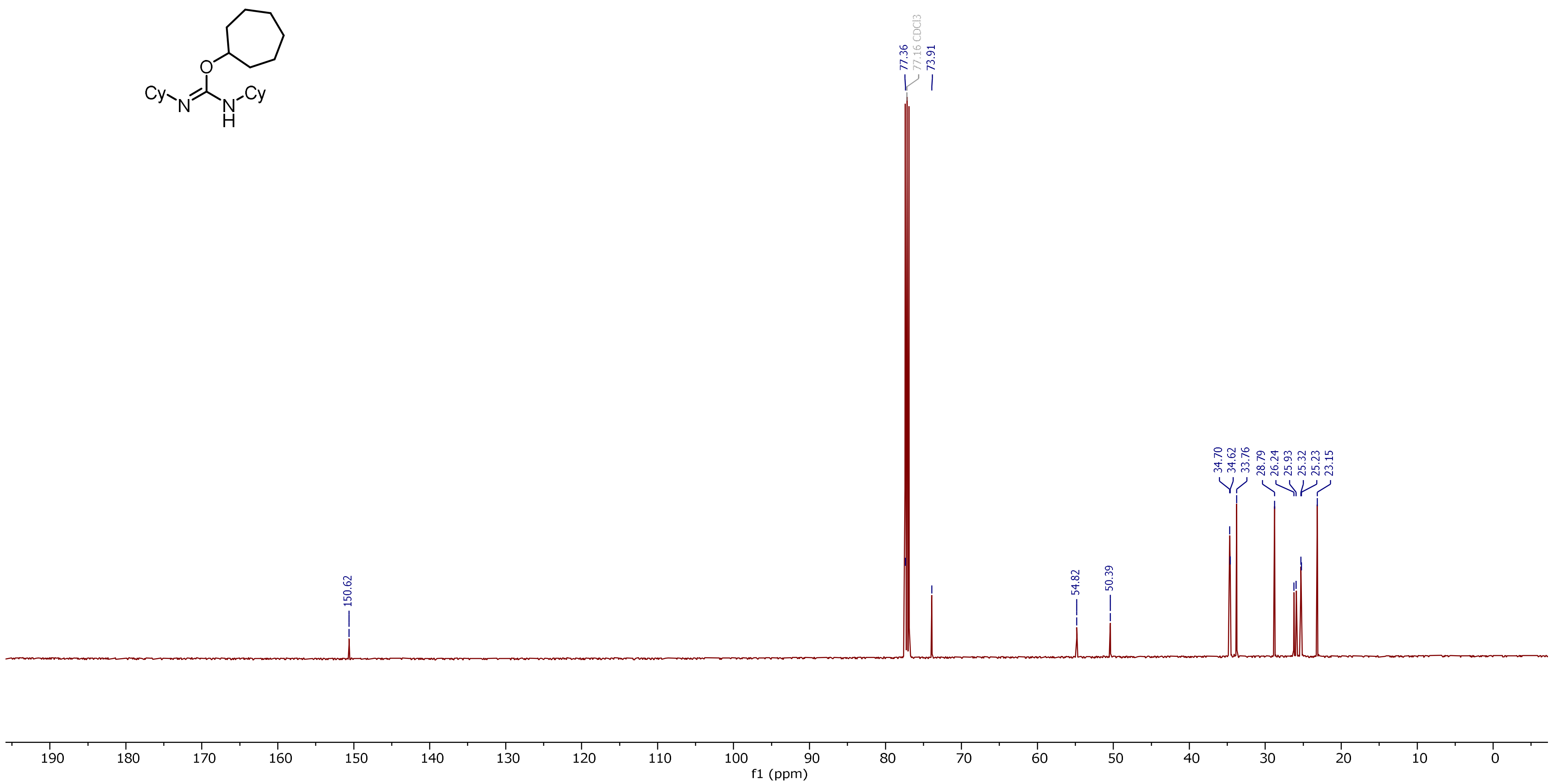

^{13}C NMR (126 MHz, $CDCl_3$) spectrum of O-(cycloheptyl)-*N*,*N'*-dicyclohexylisourea.

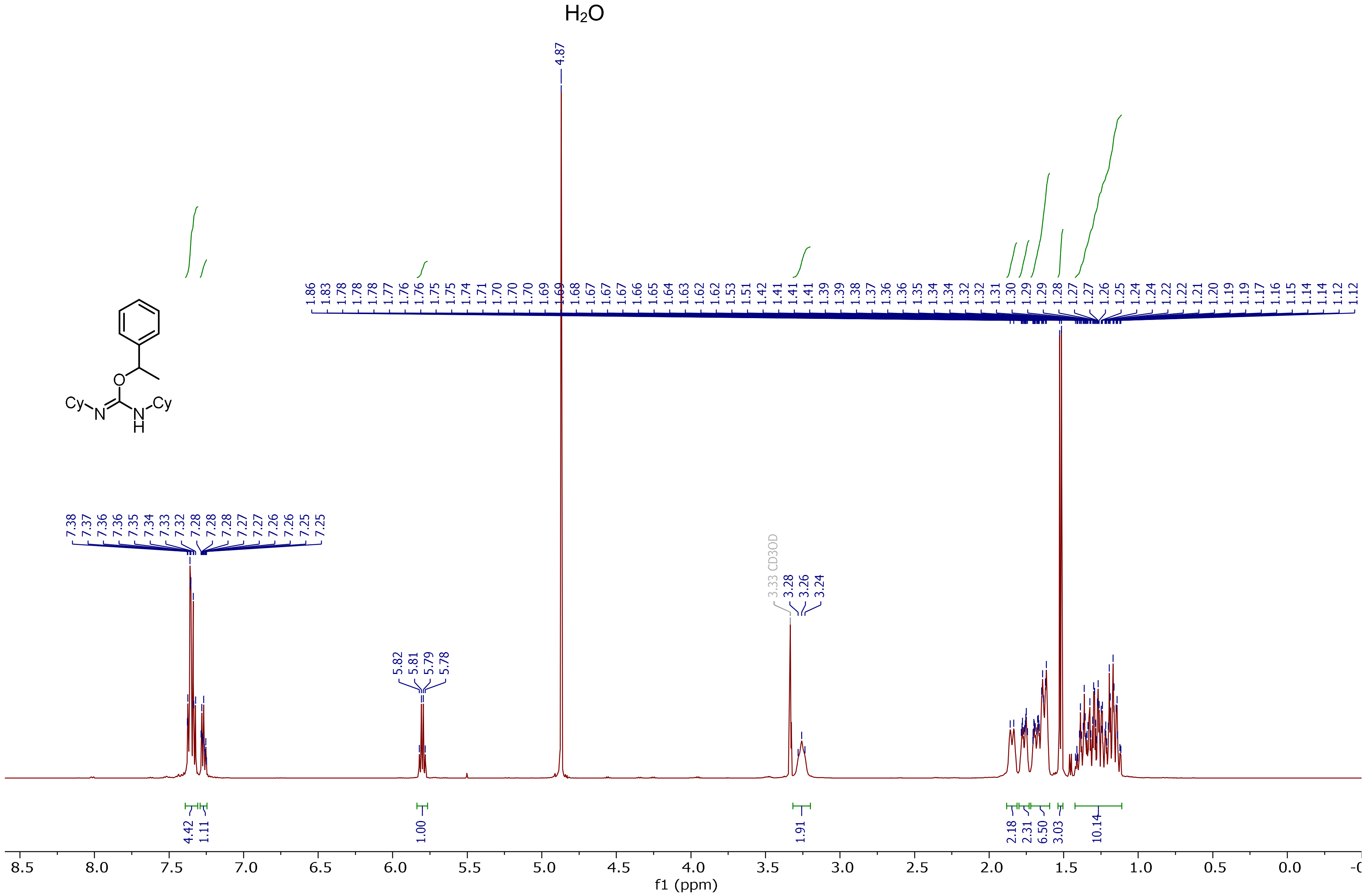

^{1}H NMR (500 MHz, CD_3OD) spectrum of O-(1-phenylethyl)-*N*,*N′*-dicyclohexylisourea.

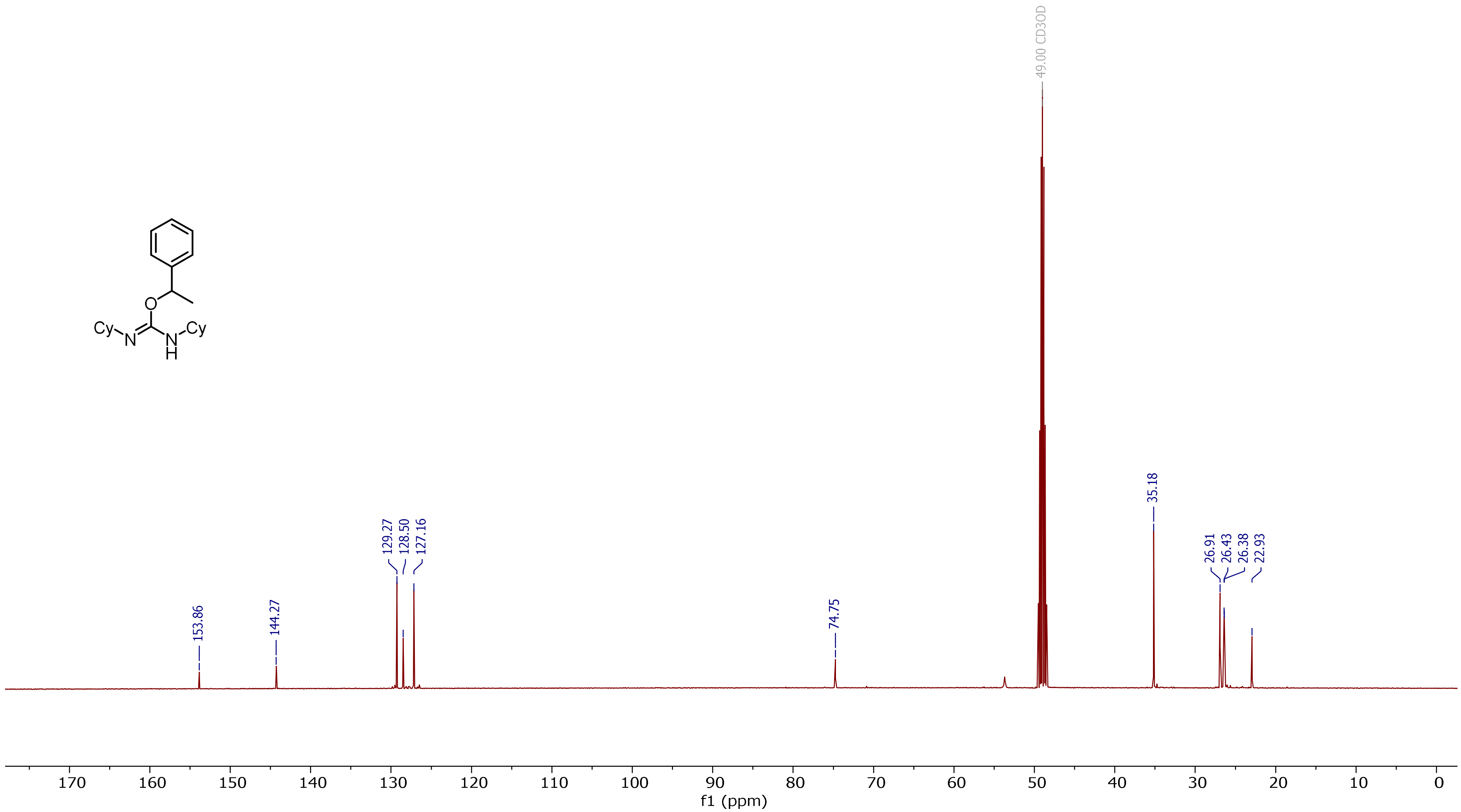

^{13}C NMR (126 MHz, CD_3OD) spectrum of O-(1-phenylethyl)-*N*,*N'*-dicyclohexylisourea.

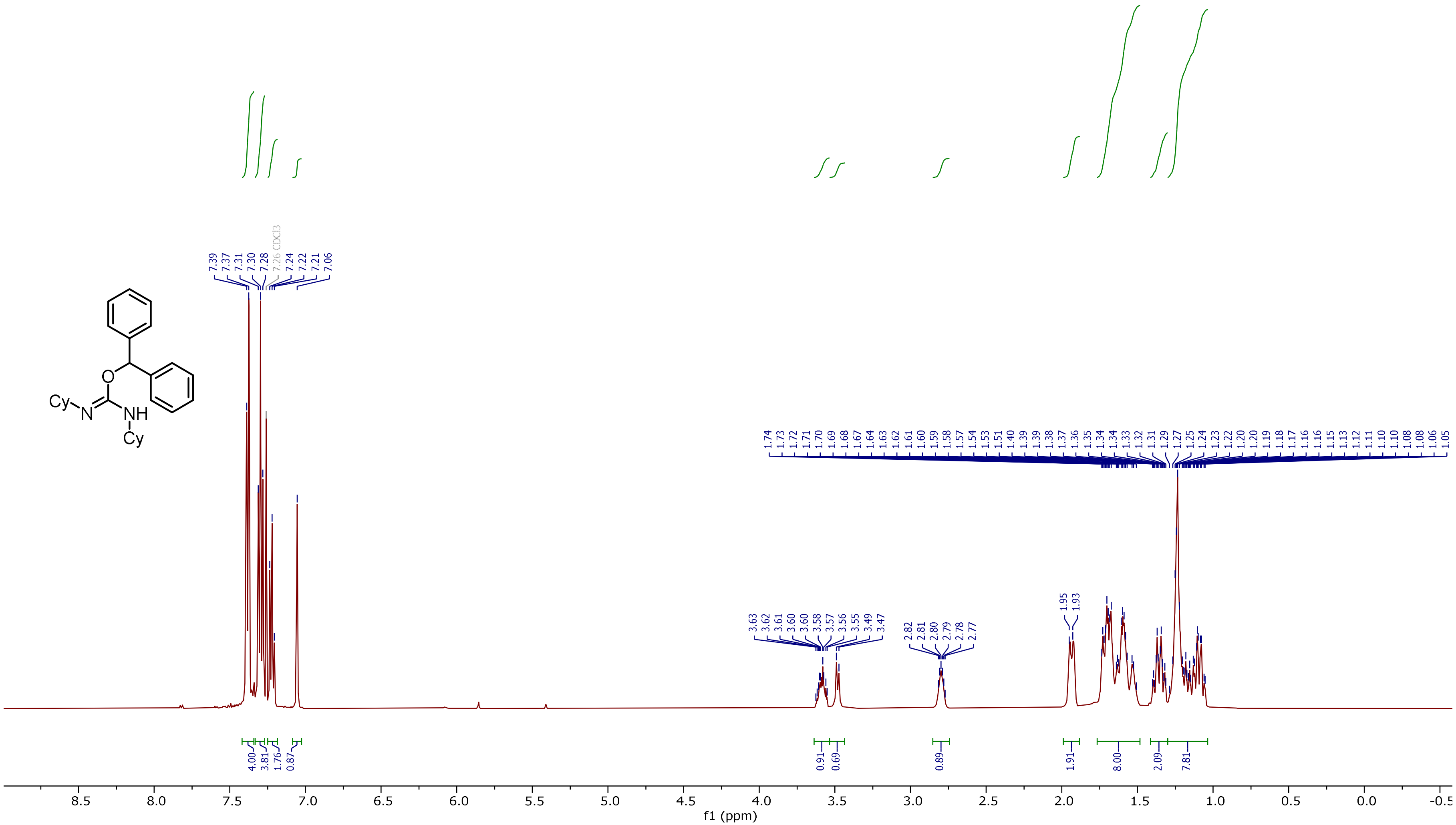

^{1}H NMR (500 MHz, $CDCl_3$) spectrum of O-(benzhydryl)-*N*,*N'*-dicyclohexylisourea.

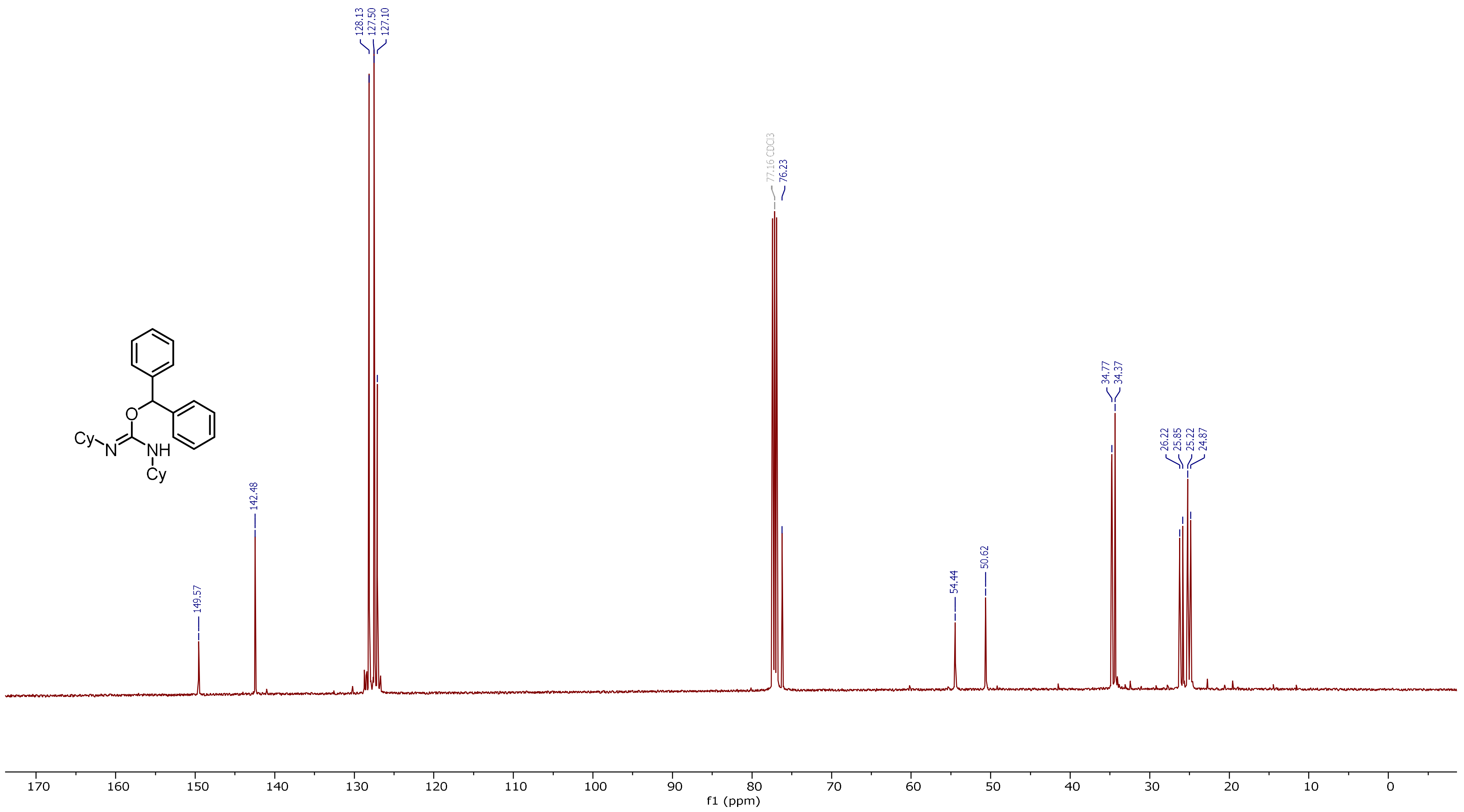

^{13}C NMR (126 MHz, $CDCl_3$) spectrum of O-(benzhydryl)-*N*,*N′*-dicyclohexylisourea.

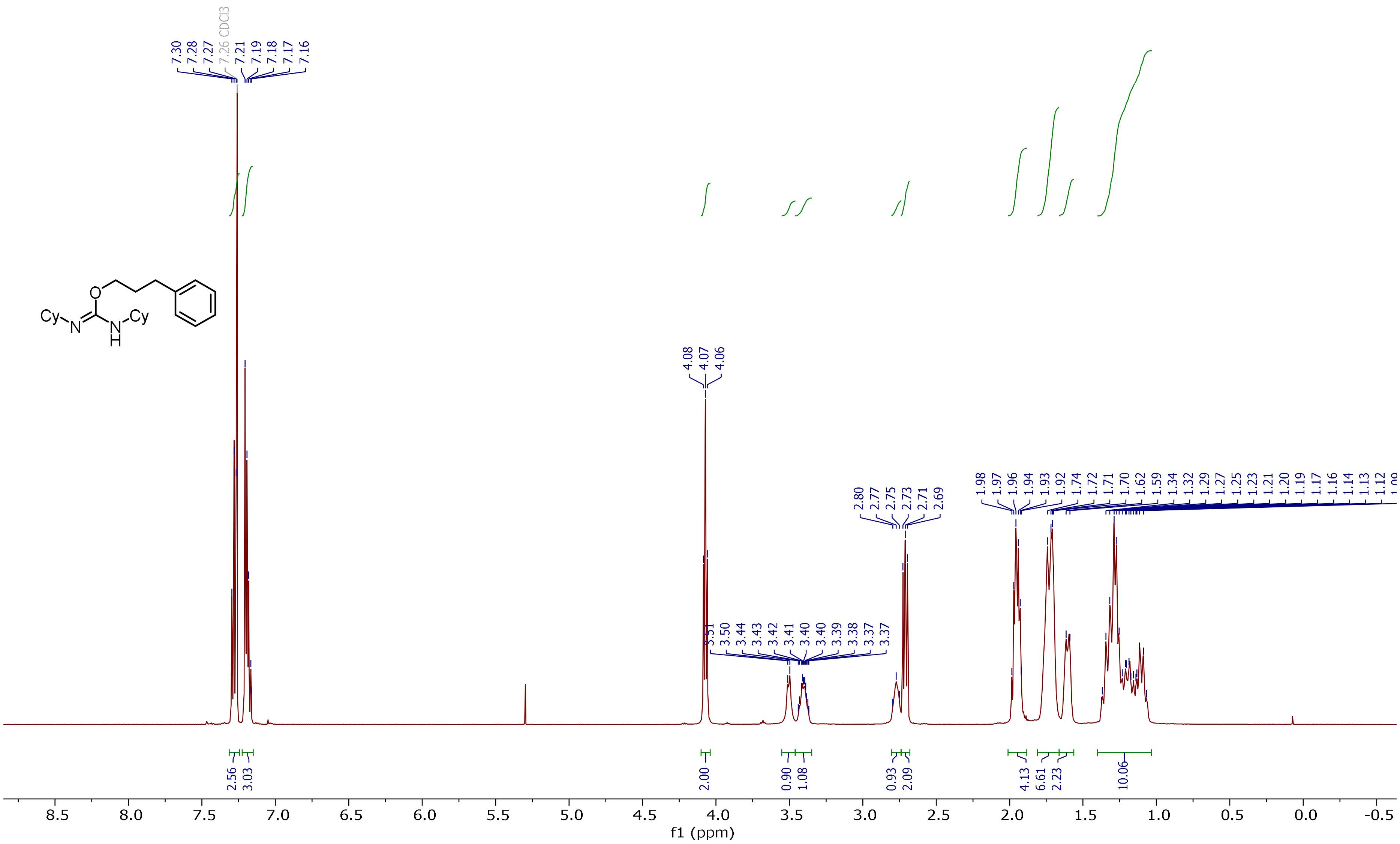

^{1}H NMR (500 MHz, $CDCl_3$) spectrum of O-(3-phenyl-1-propyl)-*N*,*N'*-dicyclohexylisourea.

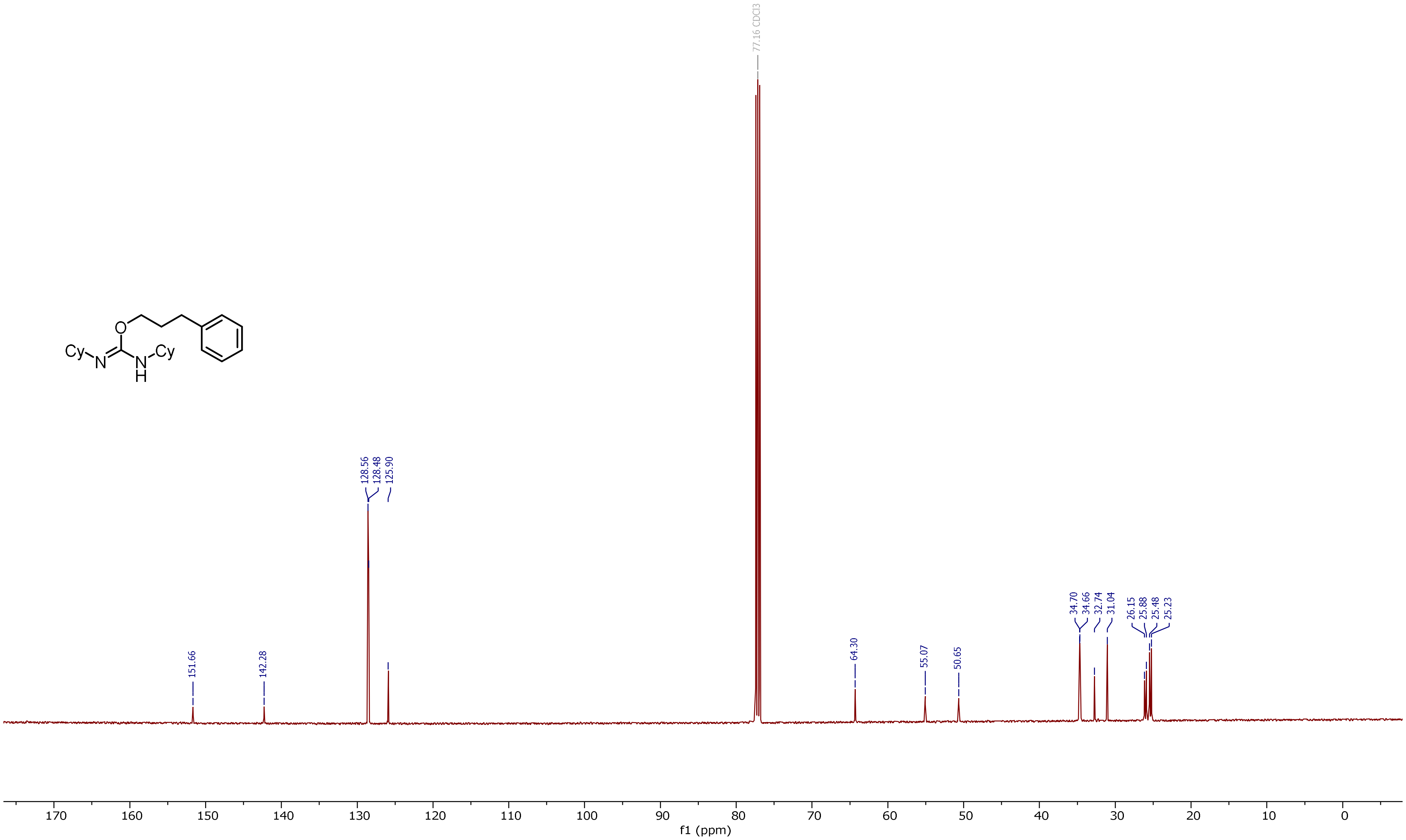

^{13}C NMR (126 MHz, $CDCl_3$) spectrum of O-(3-phenyl-1-propyl)-*N*,*N'*-dicyclohexylisourea.

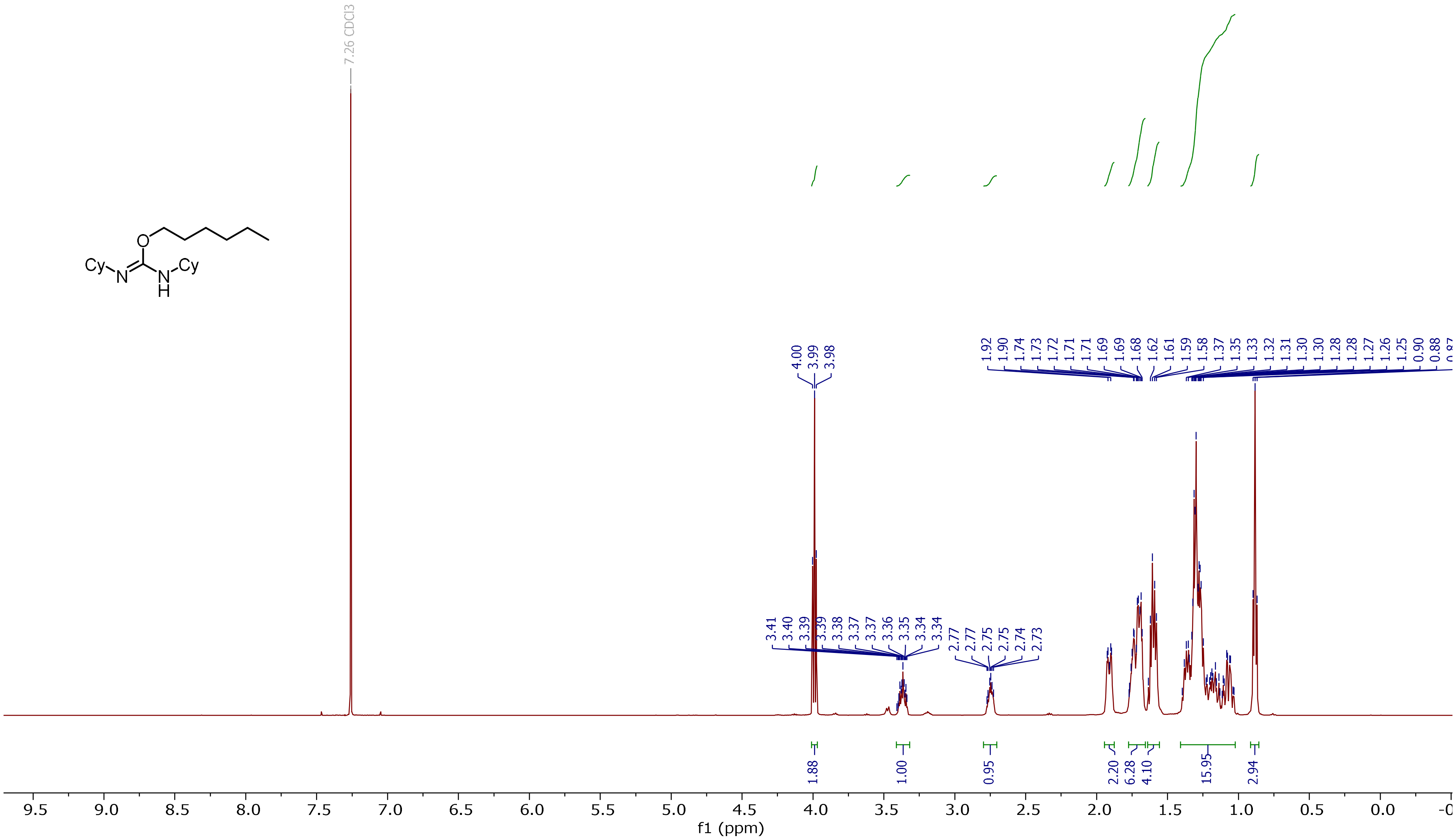

^{1}H NMR (500 MHz, $CDCl_3$) spectrum of O-(hexyl)-*N*,*N'*-dicyclohexylisourea.

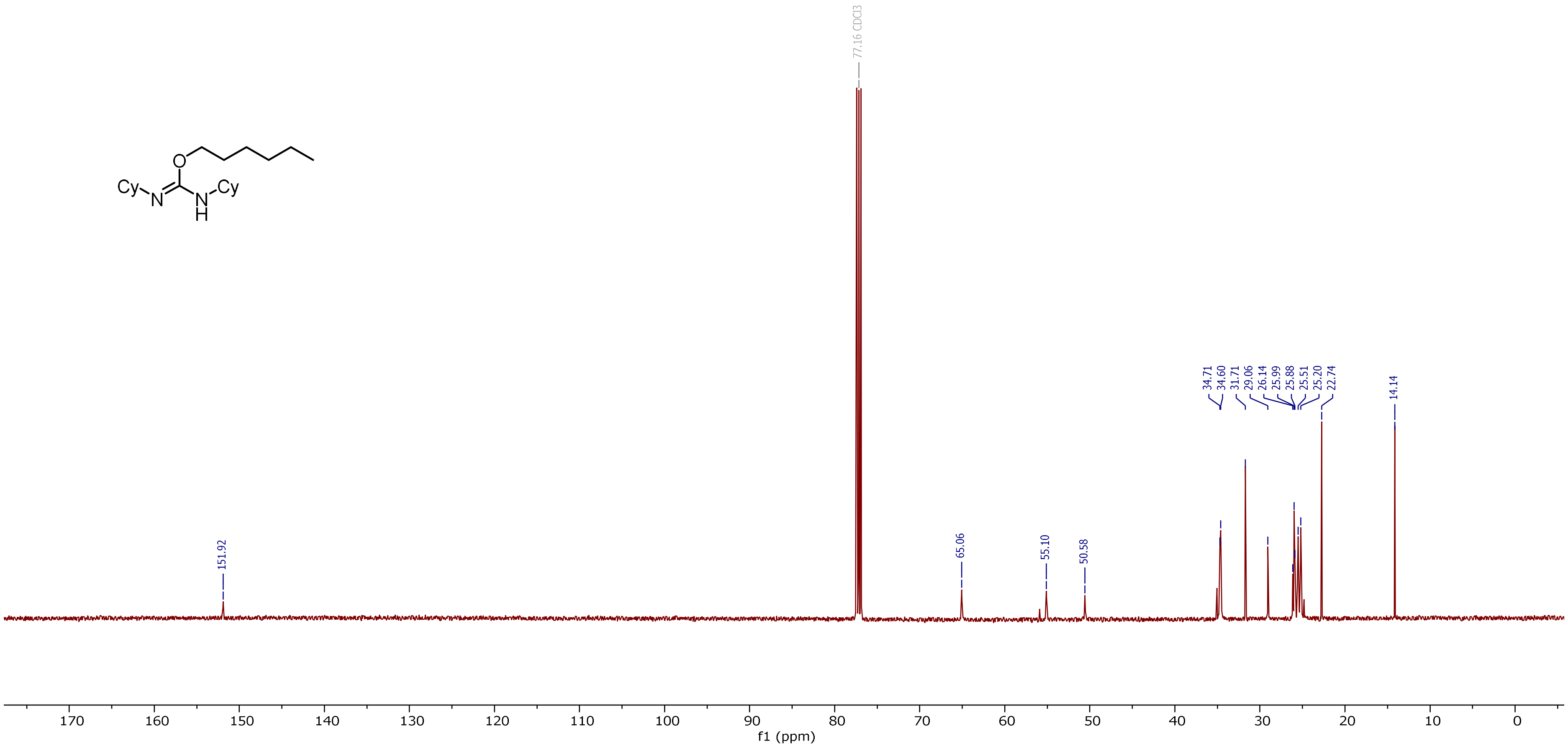

^{13}C NMR (126 MHz, $CDCl_3$) spectrum of O-(hexyl)-*N*,*N′*-dicyclohexylisourea.

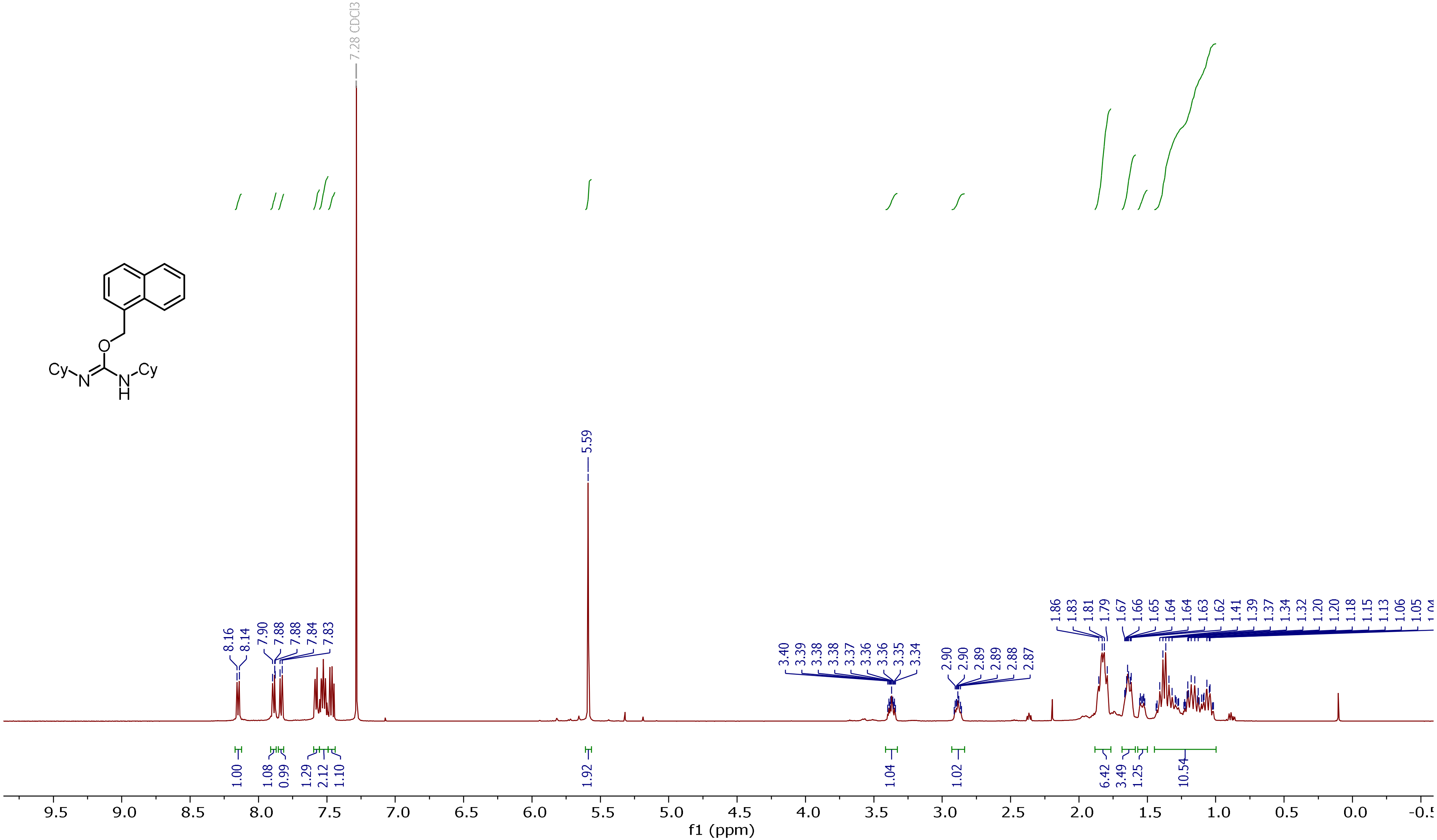

^{1}H NMR (500 MHz, $CDCl_3$) spectrum of O-(naphthylmethyl)-*N*,*N'*-dicyclohexylisourea.

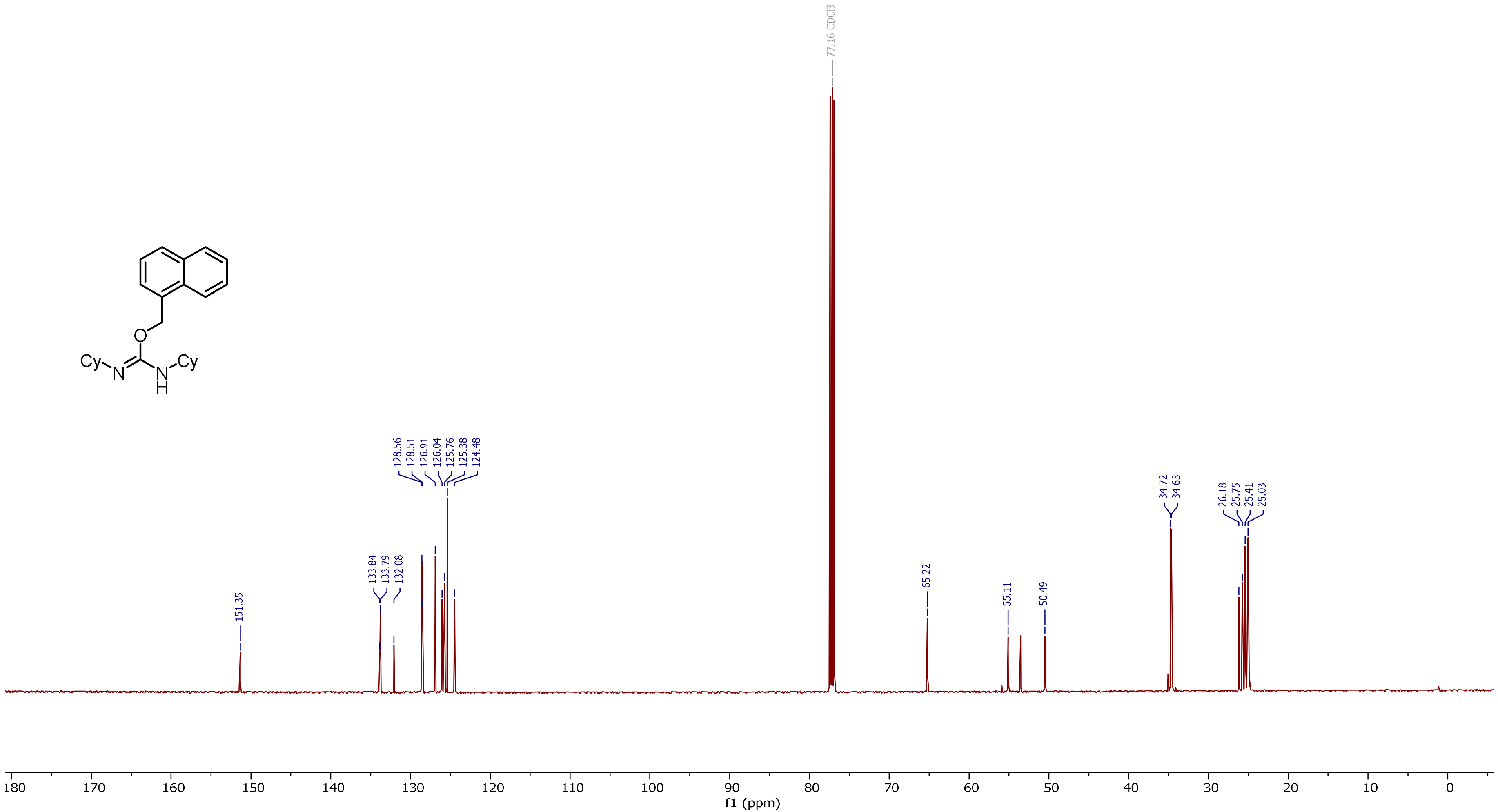

^{13}C NMR (126 MHz, $CDCl_3$) spectrum of O-(naphthylmethyl)-*N*,*N'*-dicyclohexylisourea.

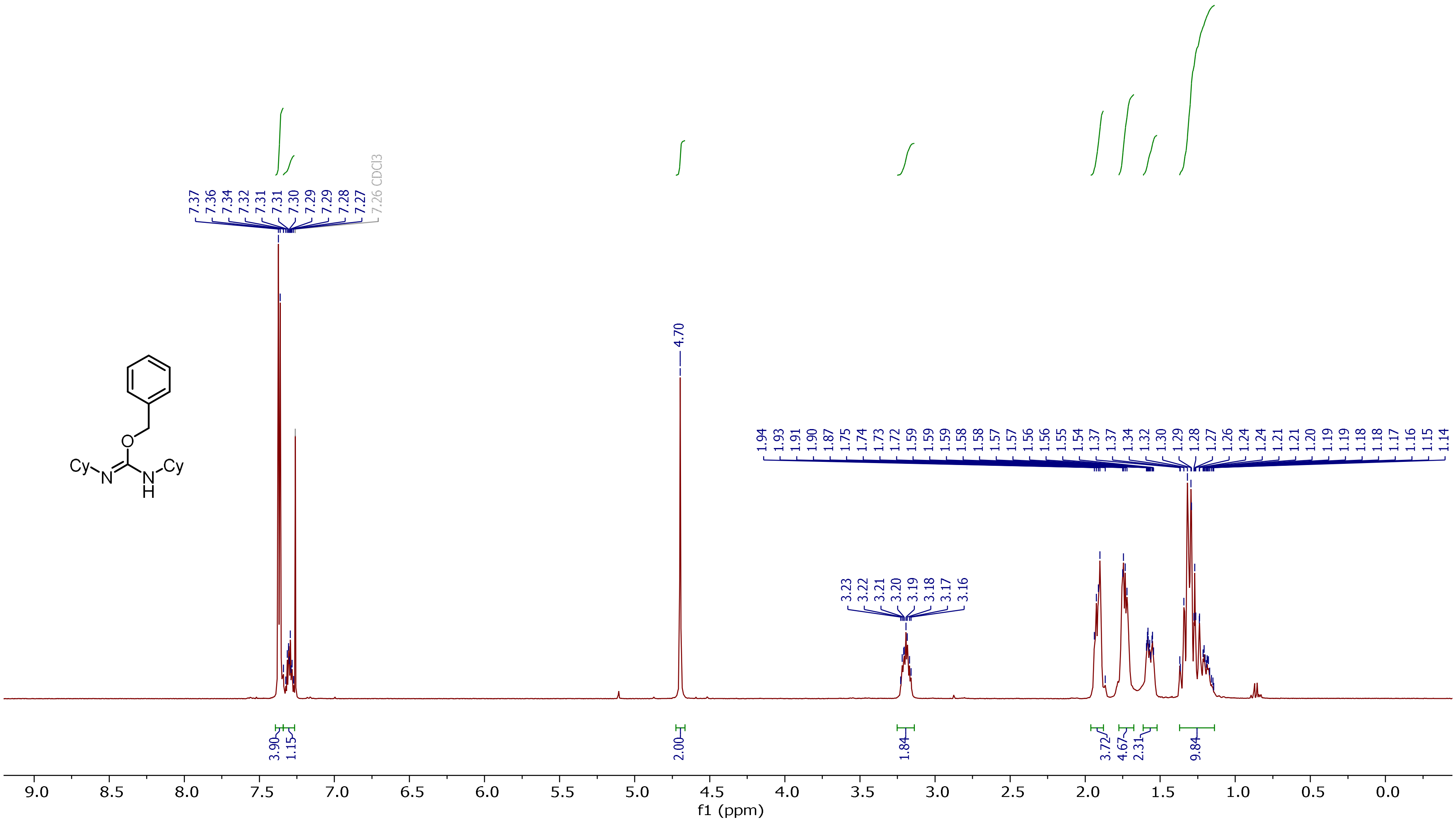

^{1}H NMR (500 MHz, $CDCl_3$) spectrum of *O*-benzyl-*N*,*N'*-dicyclohexylisourea.

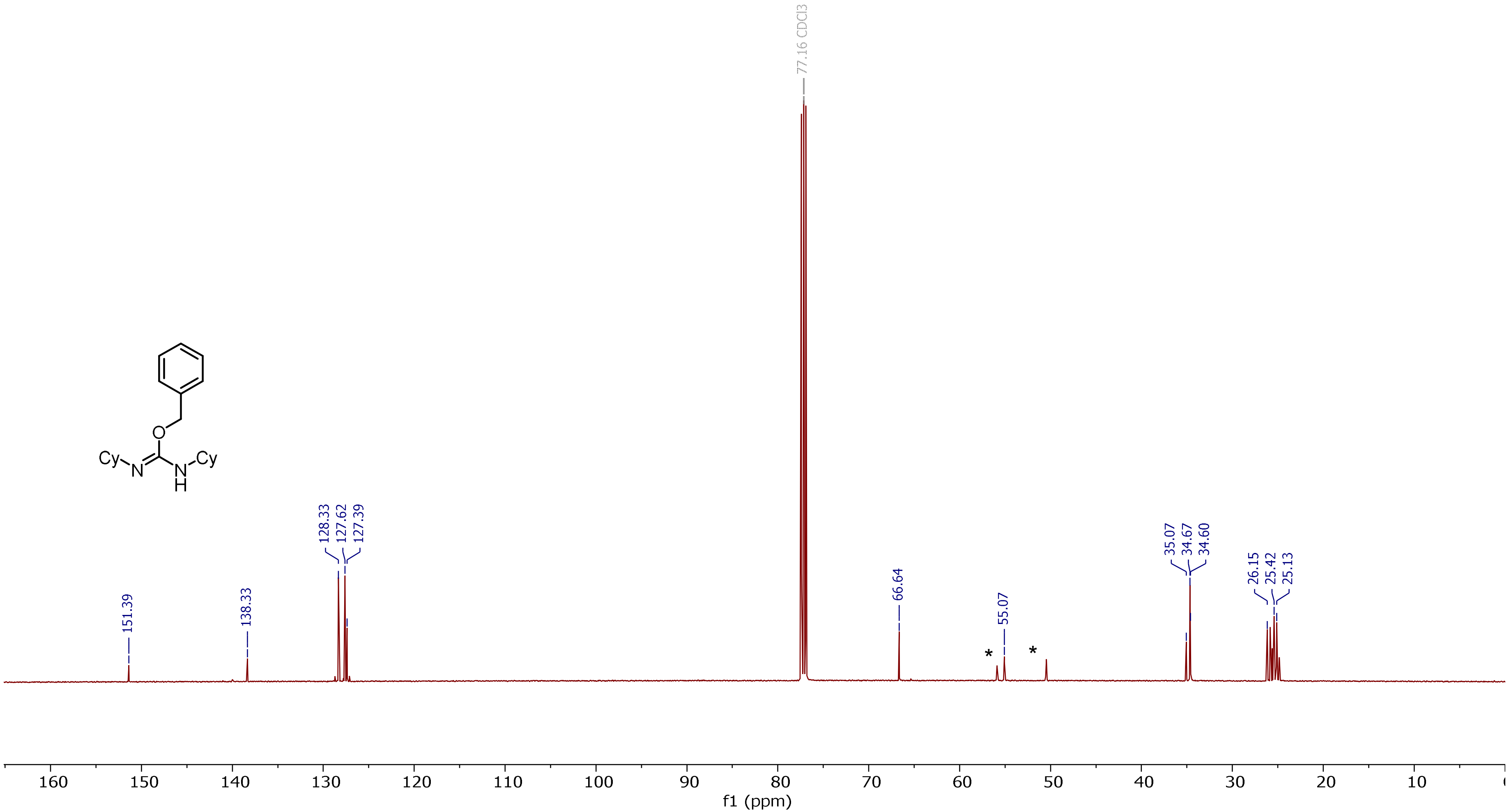

^{13}C NMR (126 MHz, $CDCl_3$) spectrum of *O*-benzyl-*N*,*N'*-dicyclohexylisourea**.** * DCC impurity

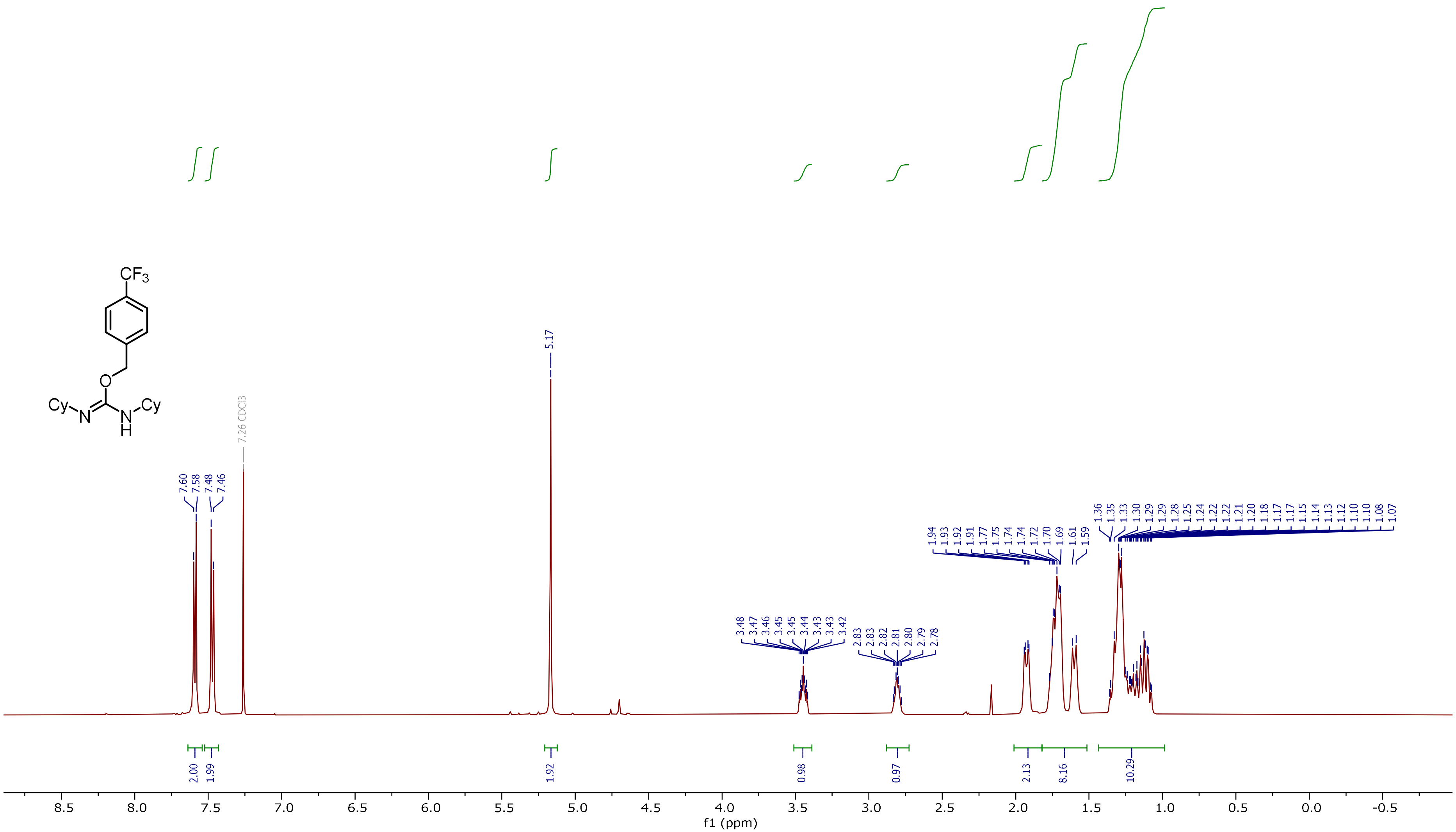

^1H NMR (500 MHz, $CDCl_3$) spectrum of O-(4-trifluoromethyl-benzyl)-*N*,*N'*-dicyclohexylisourea.

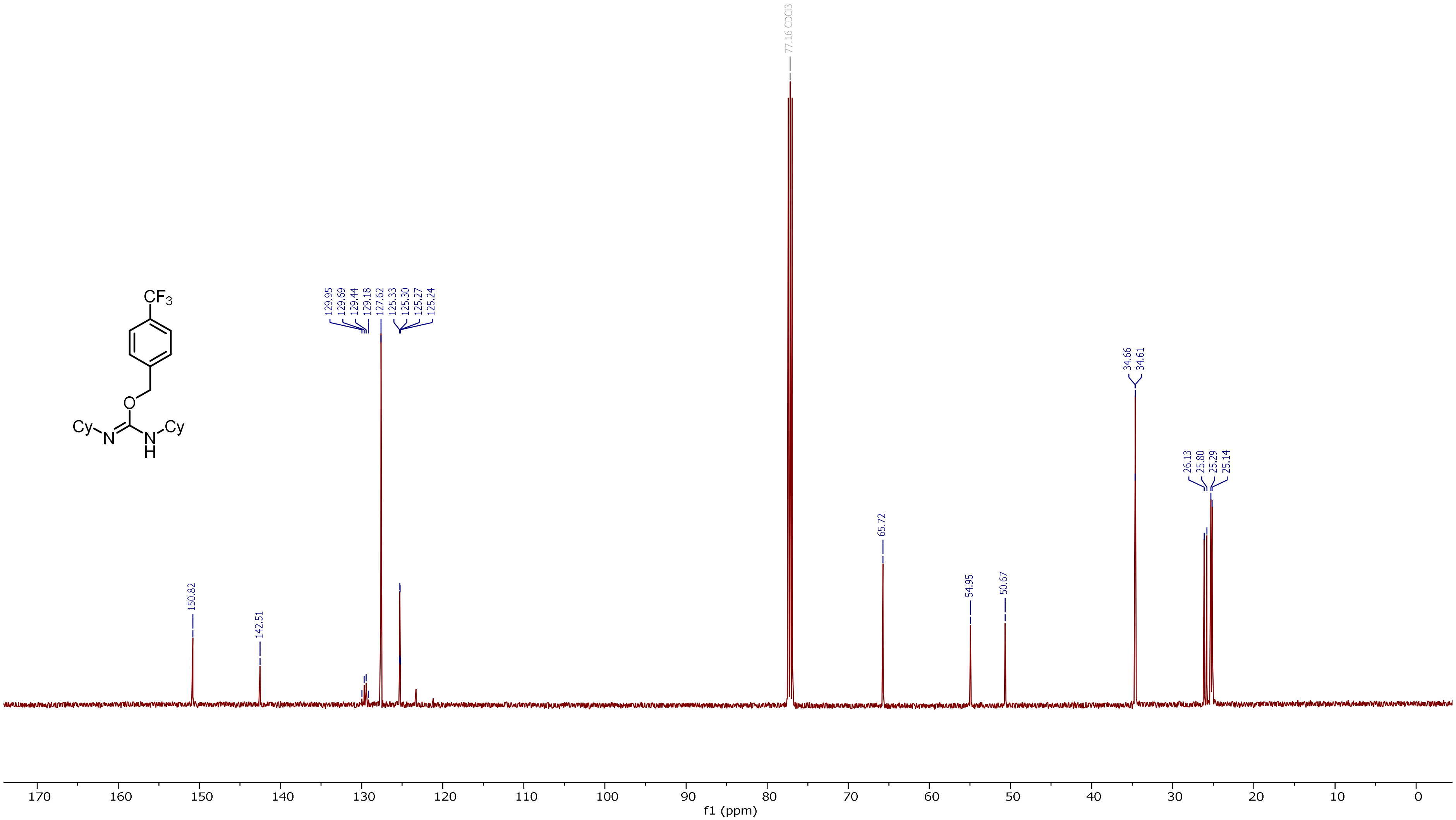

^{13}C NMR (126 MHz, $CDCl_3$) spectrum of O-(4-trifluoromethyl-benzyl)-*N*,*N'*-dicyclohexylisourea.

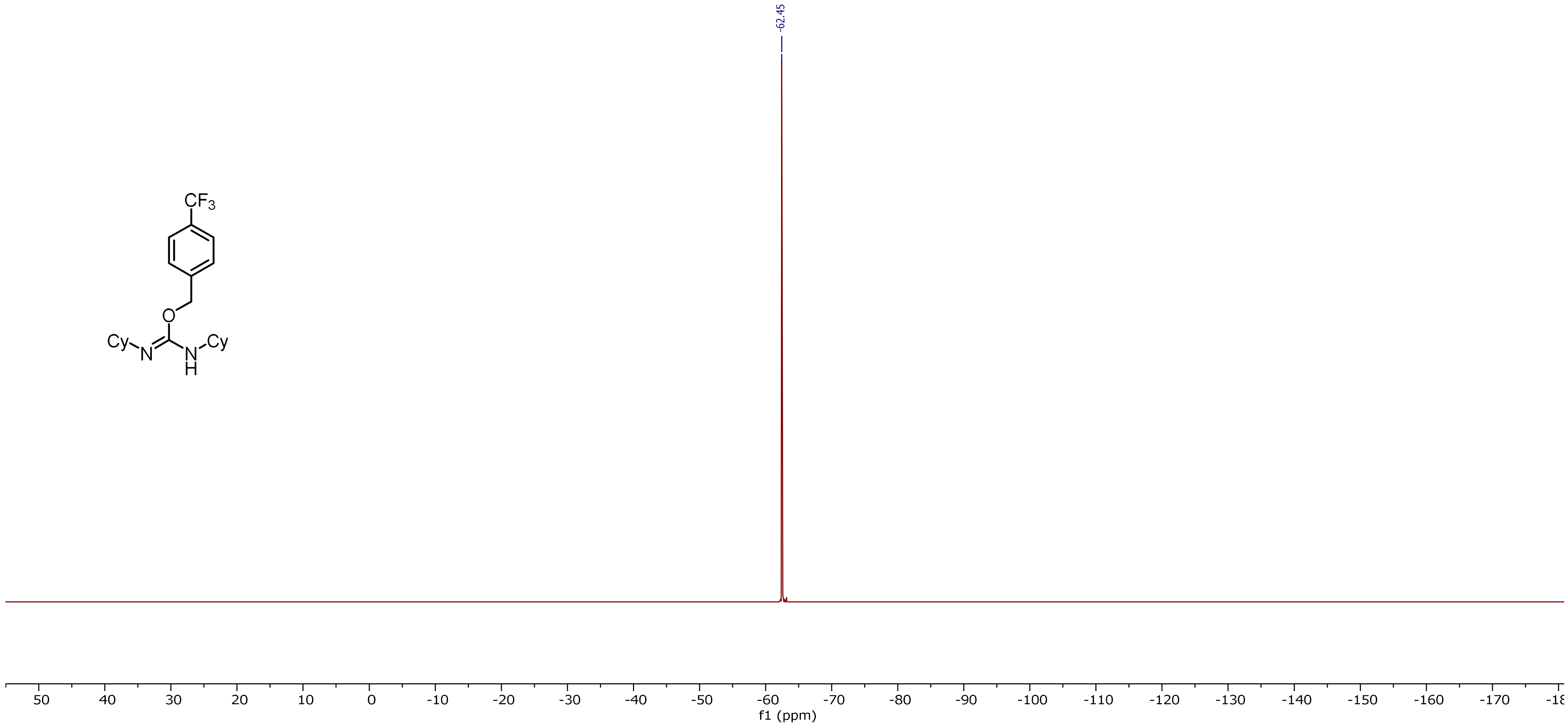

^{19}F NMR (471 MHz, $CDCl_3$) spectrum of O-(4-trifluoromethyl-benzyl)-*N*,*N'*-dicyclohexylisourea.

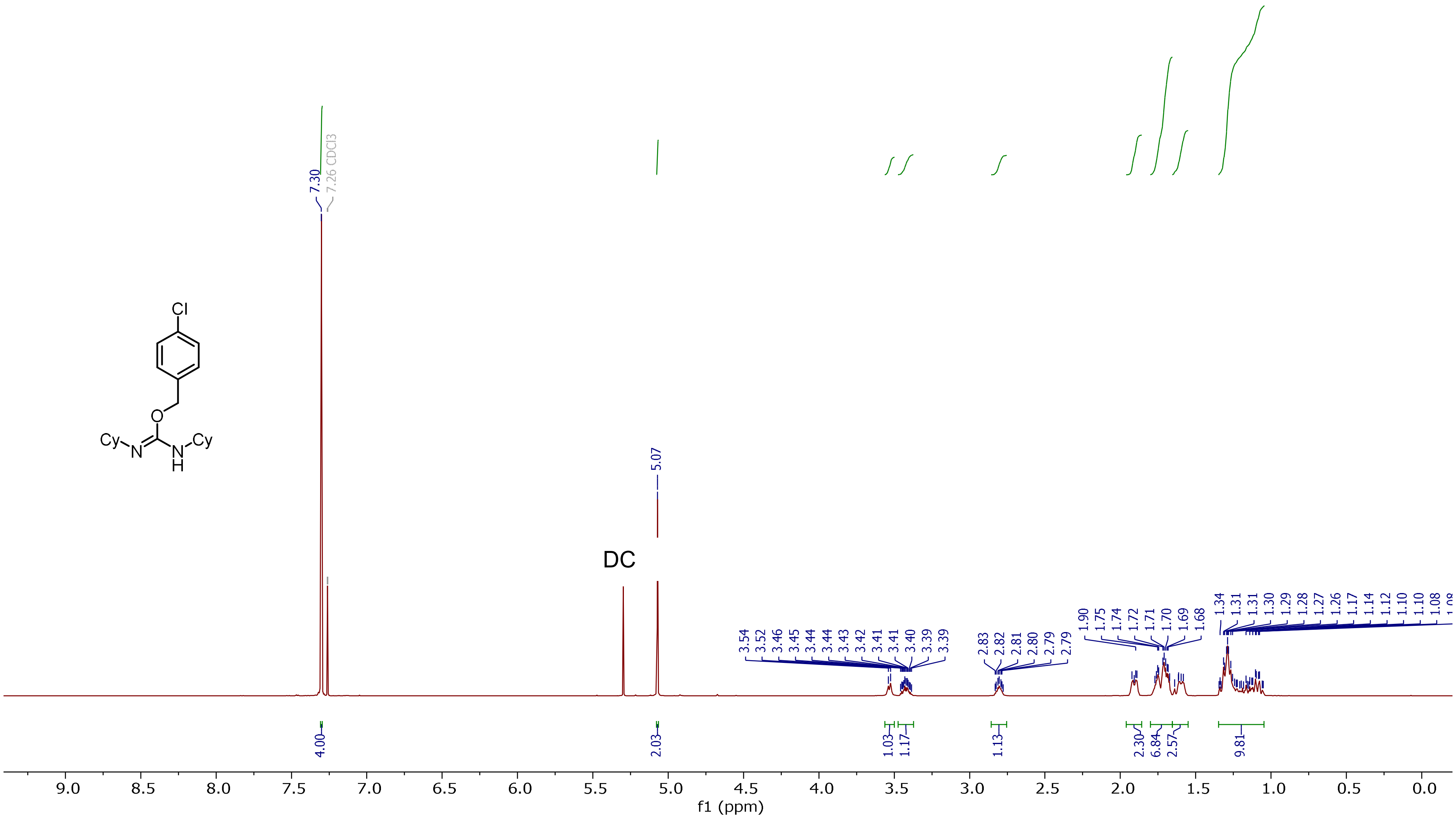

^{1}H NMR (500 MHz, $CDCl_3$) spectrum of O-(4-chloro-benzyl)-*N*,*N′*-dicyclohexylisourea.

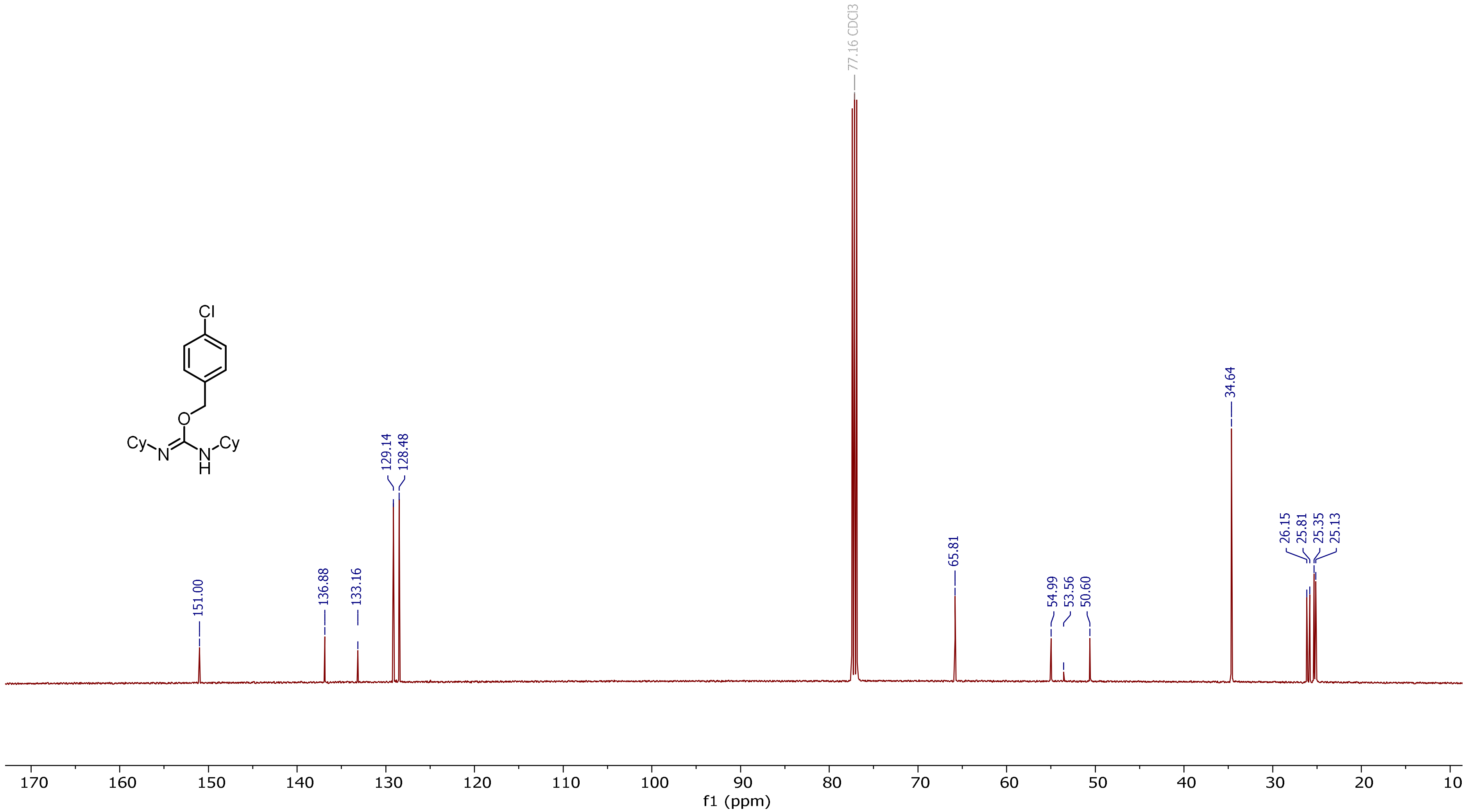

^{13}C NMR (126 MHz, $CDCl_3$) spectrum of O-(4-chloro-benzyl)-*N*,*N'*-dicyclohexylisourea.

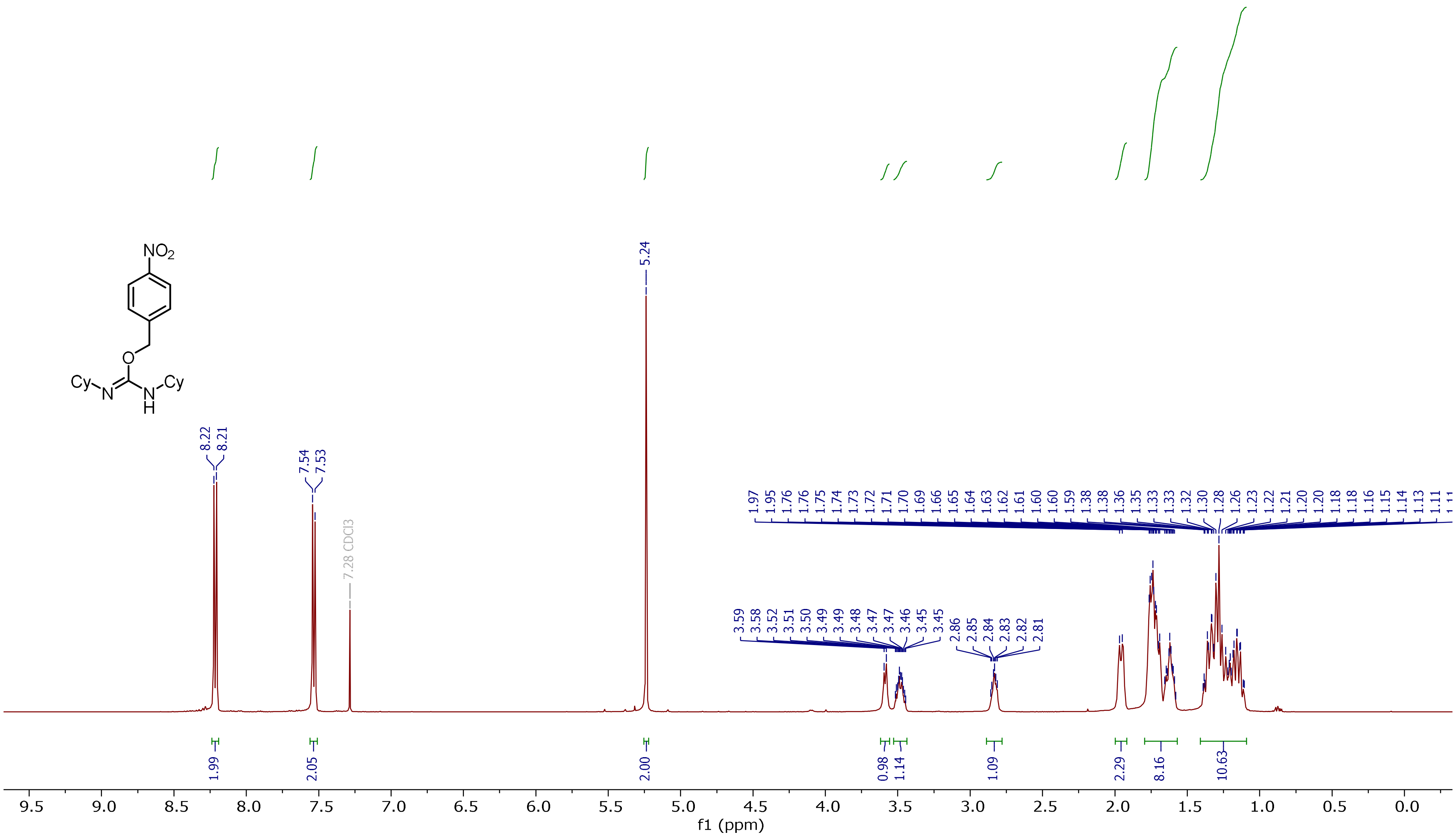

^{1}H NMR (500 MHz, $CDCl_3$) spectrum of O-(4-nitro-benzyl)-*N*,*N'*dicyclohexylisourea.

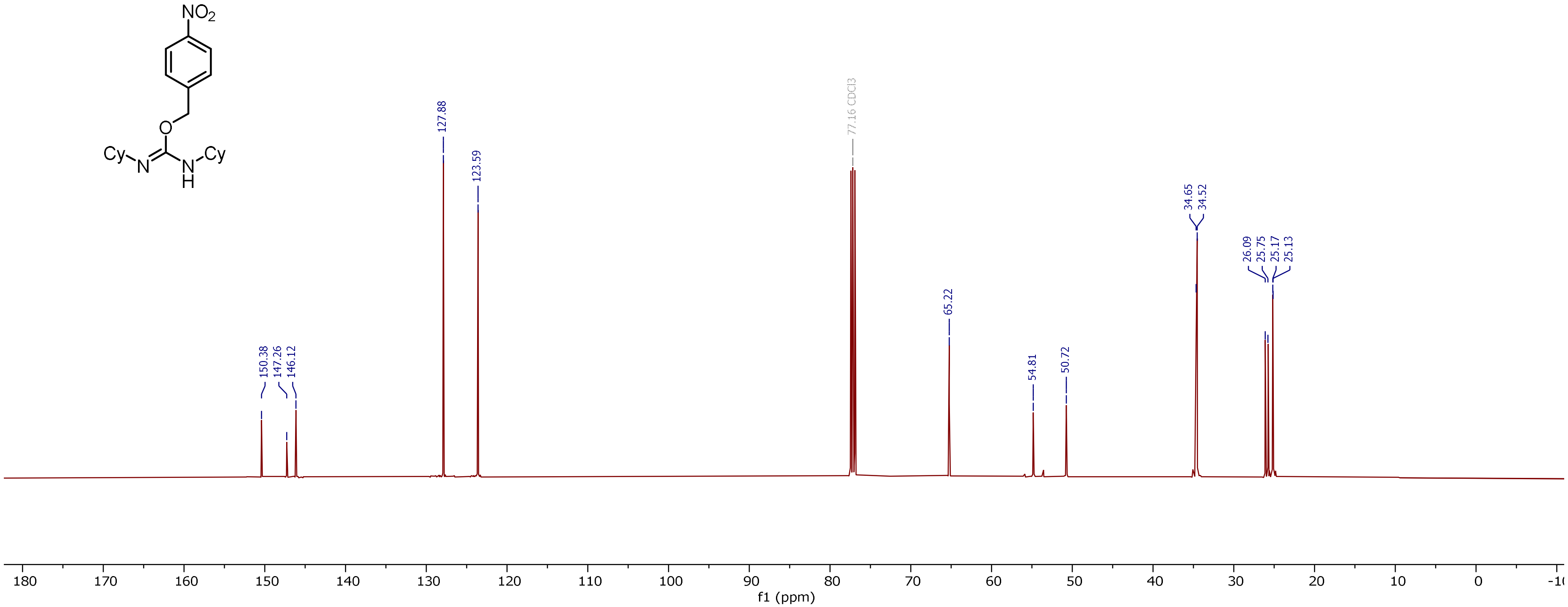

^{13}C NMR (126 MHz, $CDCl_3$) spectrum of O-(4-nitro-benzyl)-*N*,*N'*-dicyclohexylisourea.

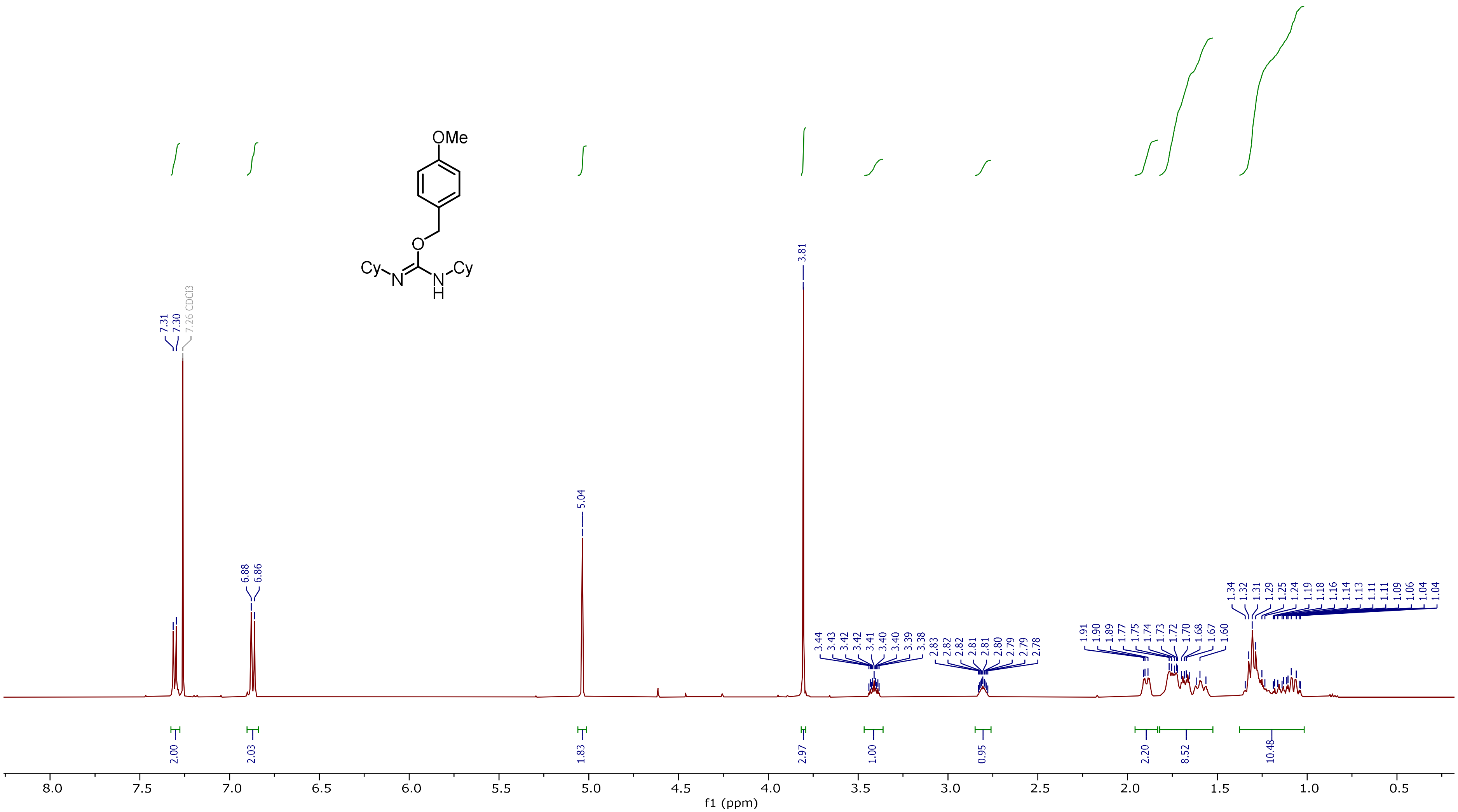

^{1}H NMR (500 MHz, $CDCl_3$) spectrum of O-(4-methoxy-benzyl)-*N*,*N′*-dicyclohexylisourea.

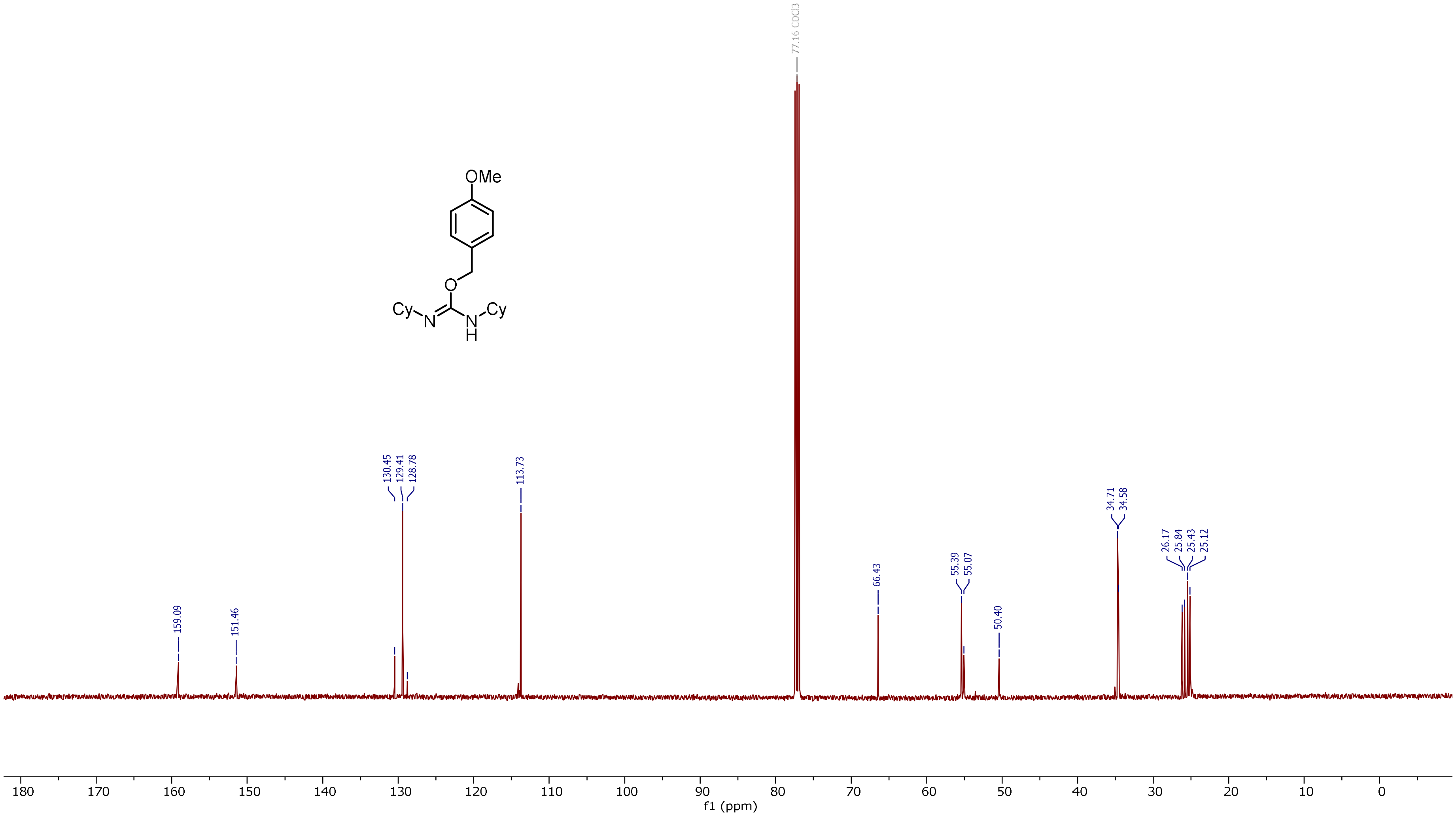

^{13}C NMR (126 MHz, $CDCl_3$) spectrum of O-(4-methoxy-benzyl)-*N*,*N'*-dicyclohexylisourea.

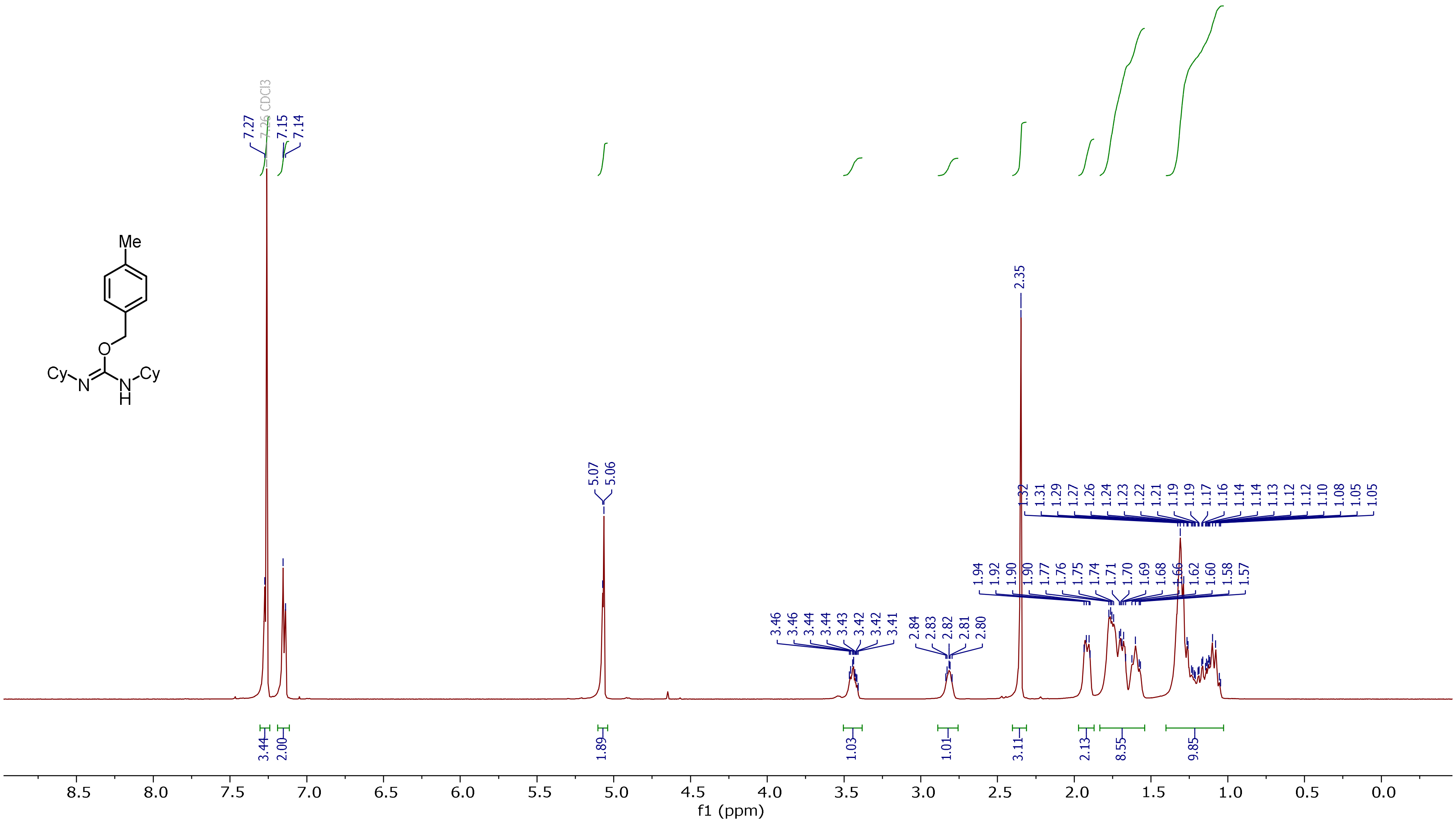

^{1}H NMR (500 MHz, $CDCl_3$) spectrum of O-(4-methyl-benzyl)-*N*,*N′*-dicyclohexylisourea.

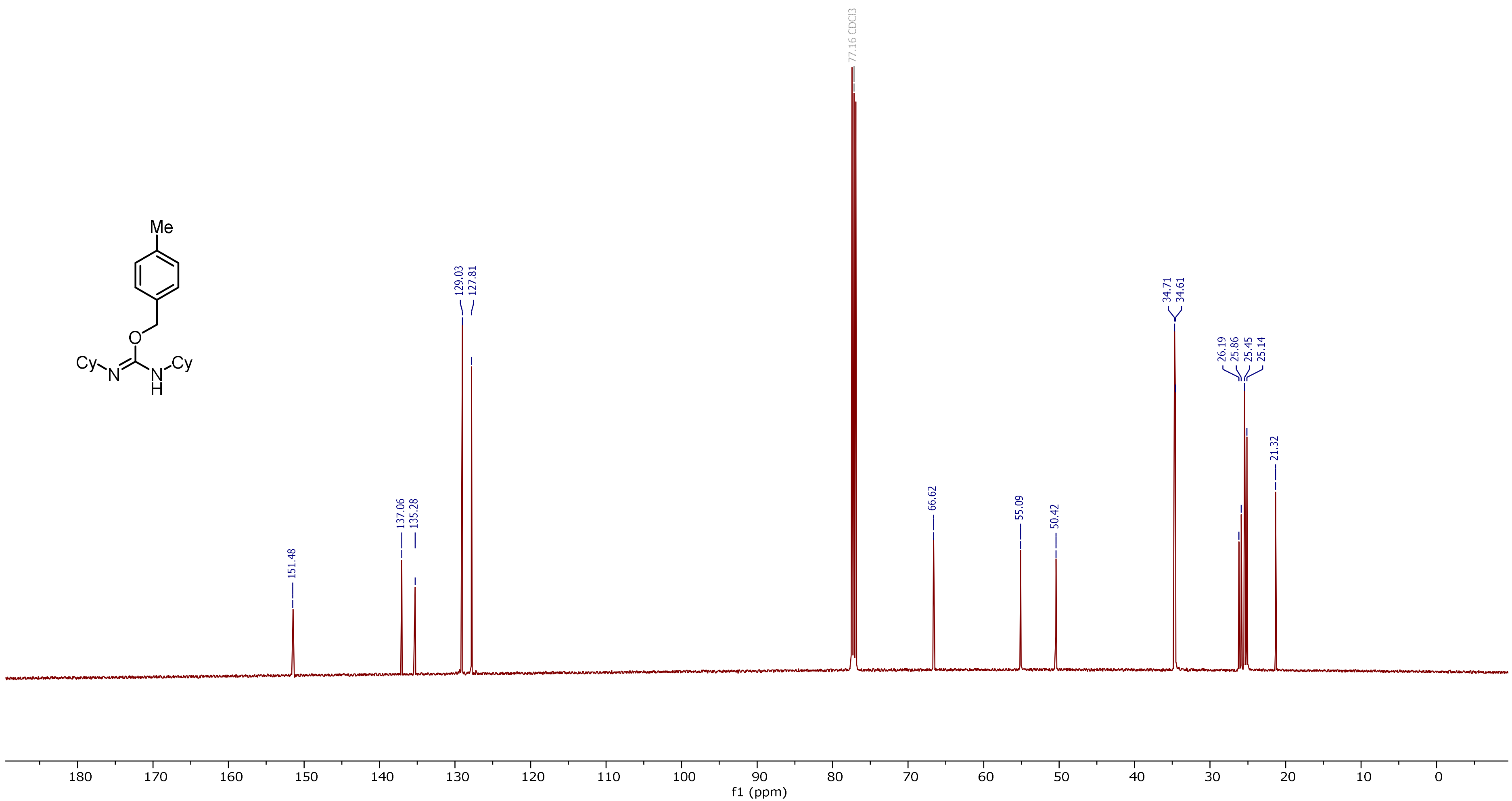

^{13}C NMR (126 MHz, $CDCl_3$) spectrum of O-(4-methyl-benzyl)-*N*,*N'*-dicyclohexylisourea.

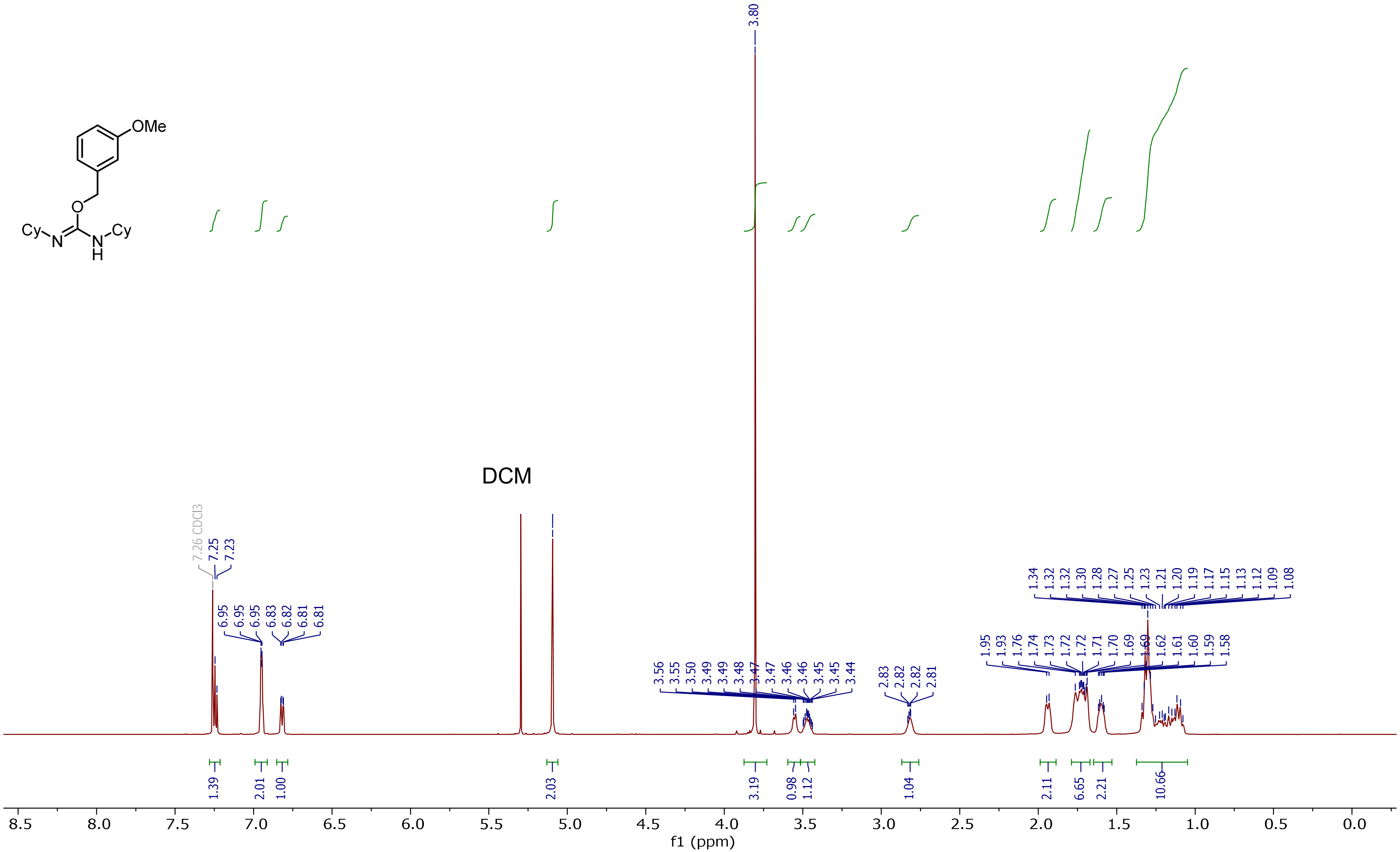

^{1}H NMR (500 MHz, $CDCl_3$) spectrum of O-(3-methoxy-benzyl)-*N*,*N'*-dicyclohexylisourea.

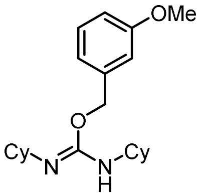

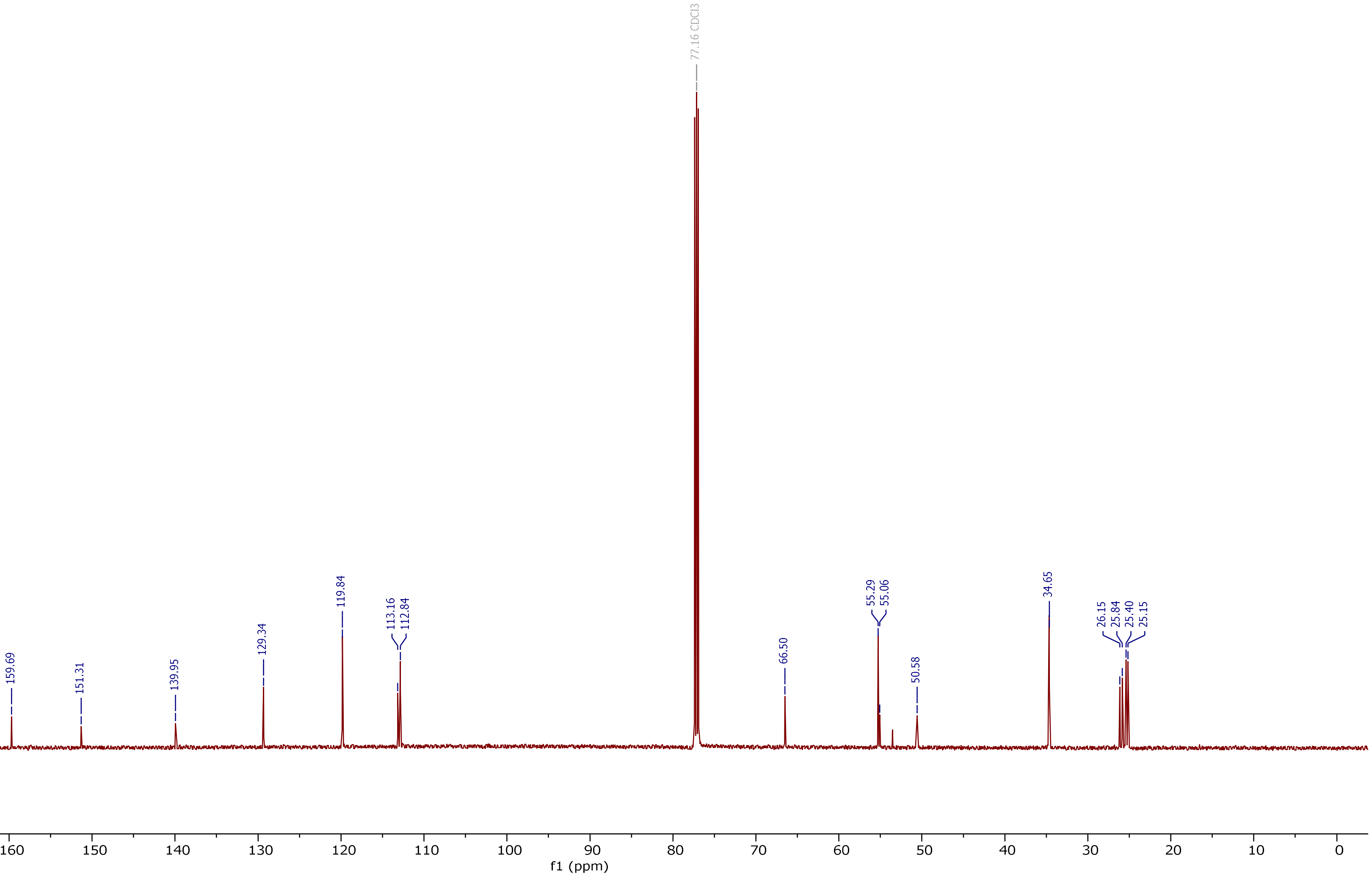

^{13}C NMR (126 MHz, $CDCl_3$) spectrum of O-(3-methoxy-benzyl)-*N*,*N′*-dicyclohexylisourea.

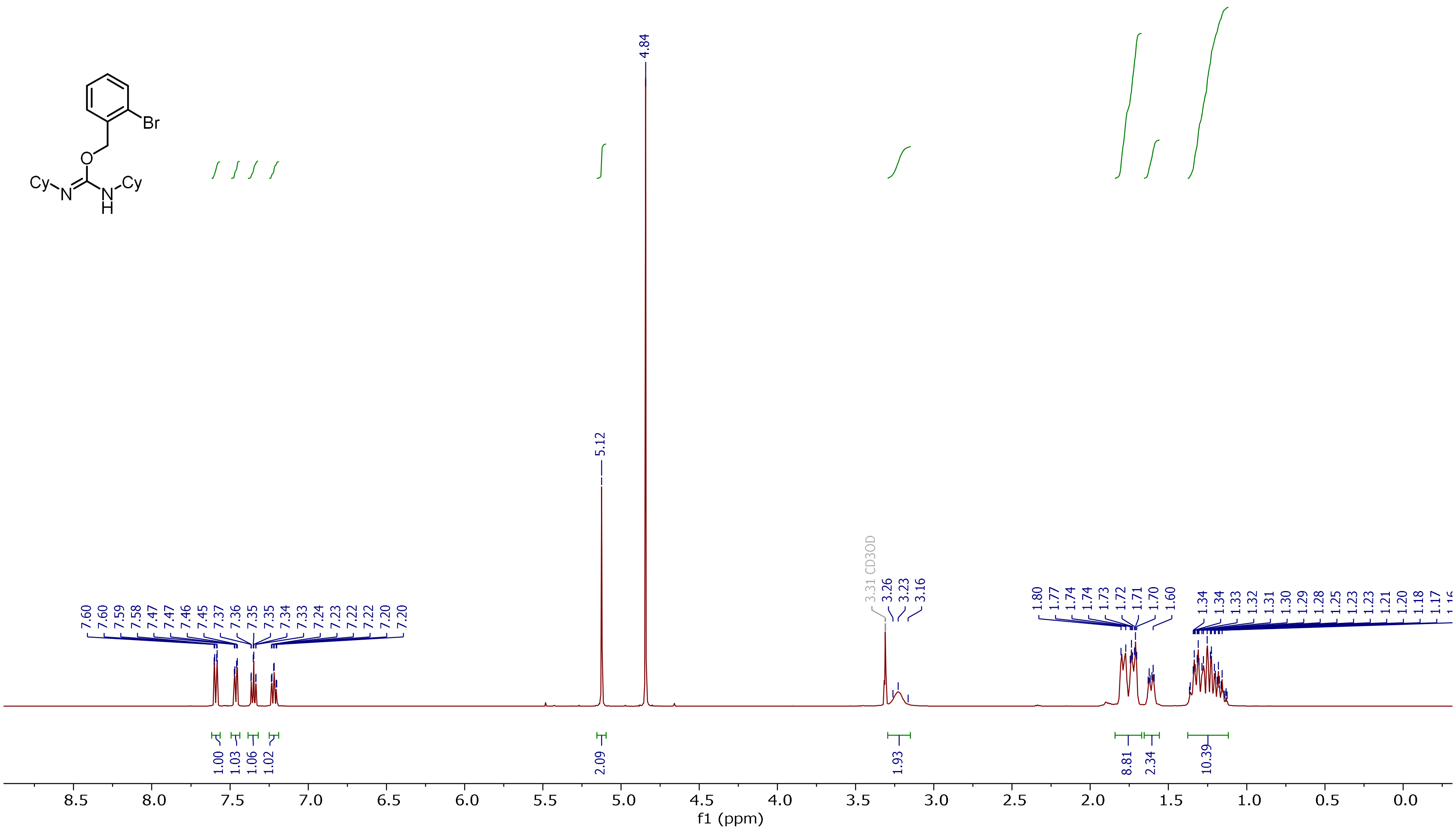

^{1}H NMR (500 MHz, CD_3OD) spectrum of O-(2-bromo-benzyl)-*N*,*N'*-dicyclohexylisourea.

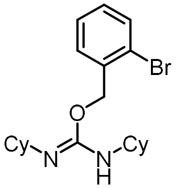

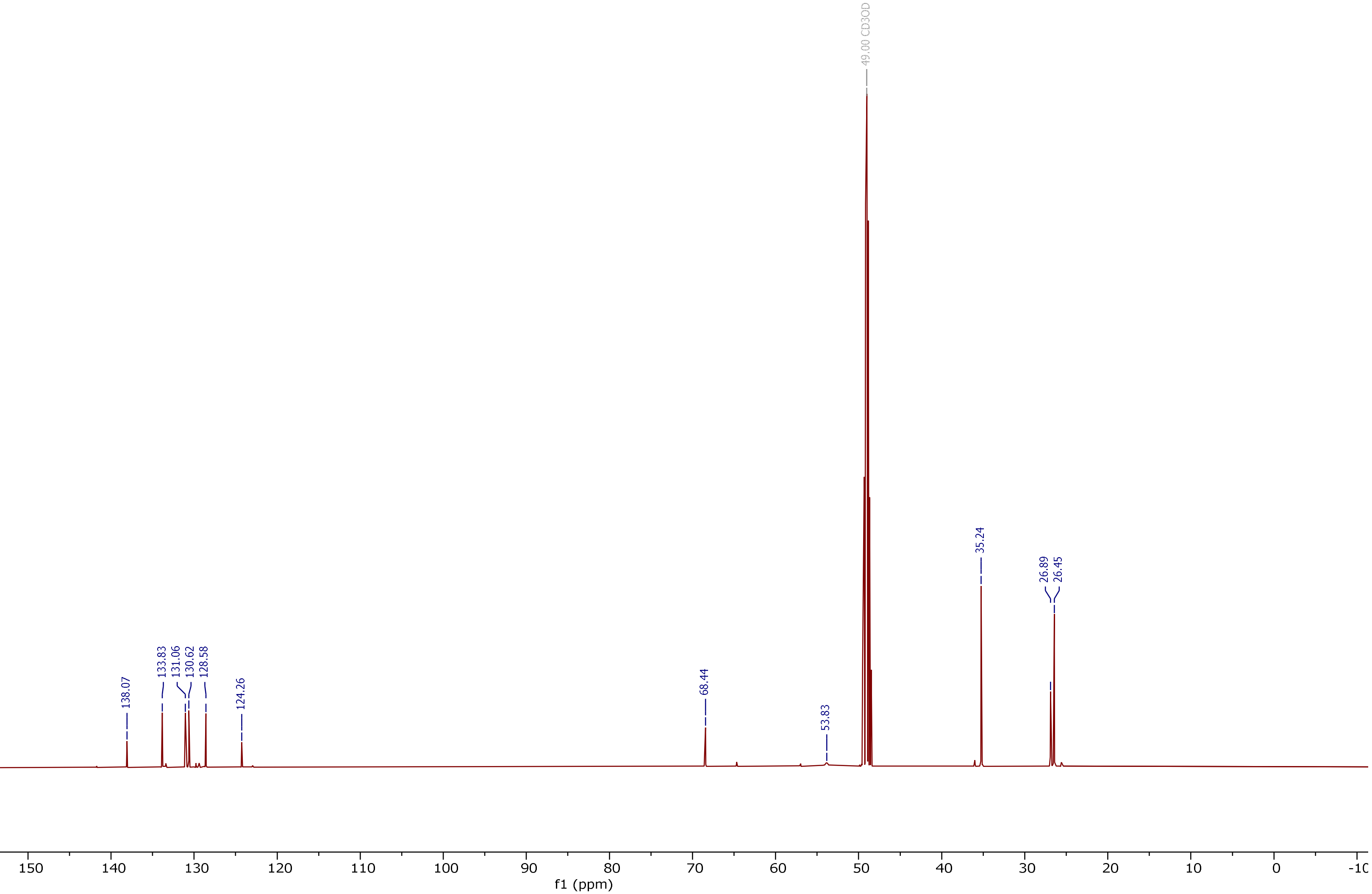

^{13}C NMR (126 MHz, CD_3OD) spectrum of O-(2-bromo-benzyl)-*N*,*N'*-dicyclohexylisourea.

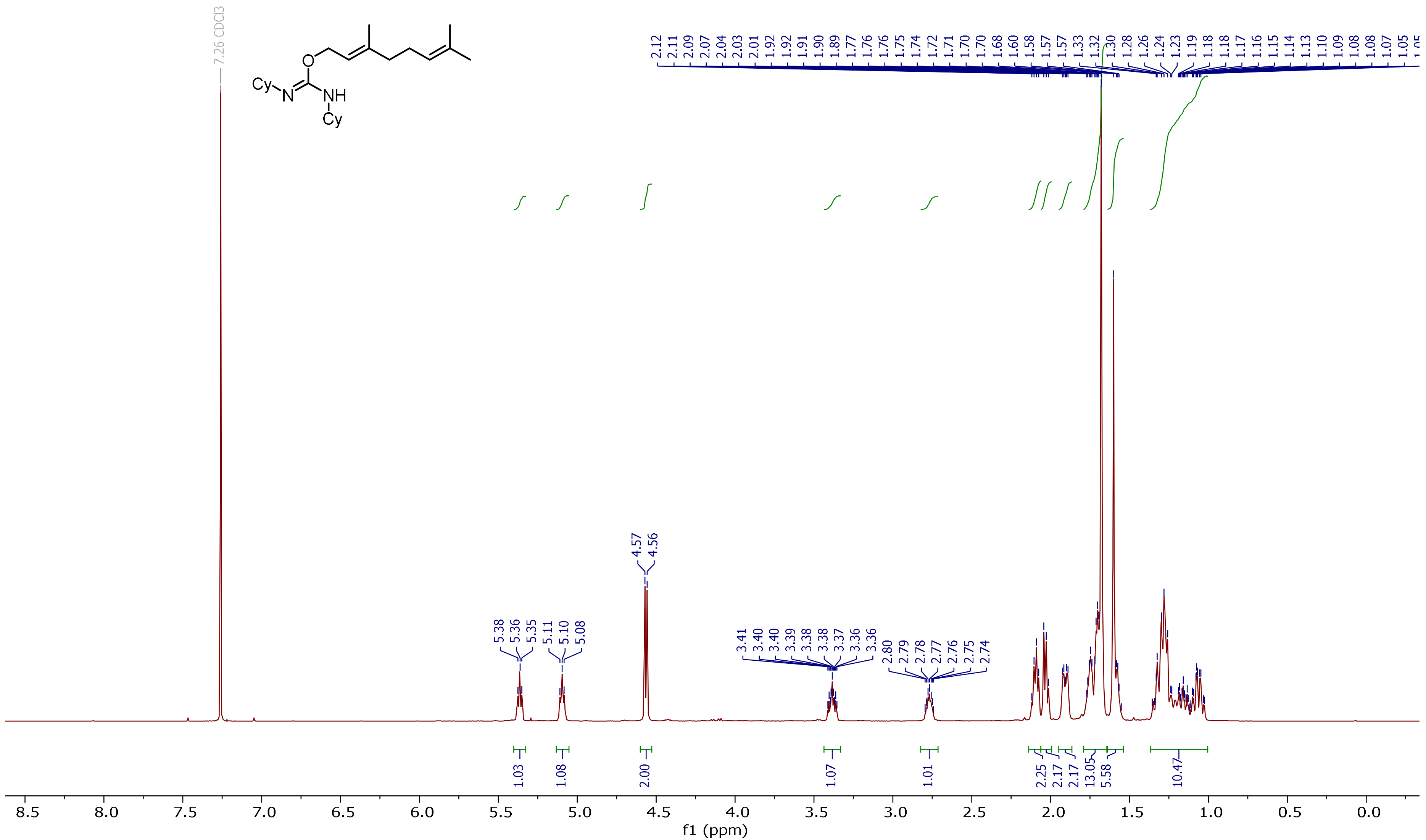

^{1}H NMR (500 MHz, $CDCl_3$) spectrum of O-(2-(E)-3,7-dimethyl-octa-2,6-dienyl)-*N*,*N′*-dicyclohexylisourea.

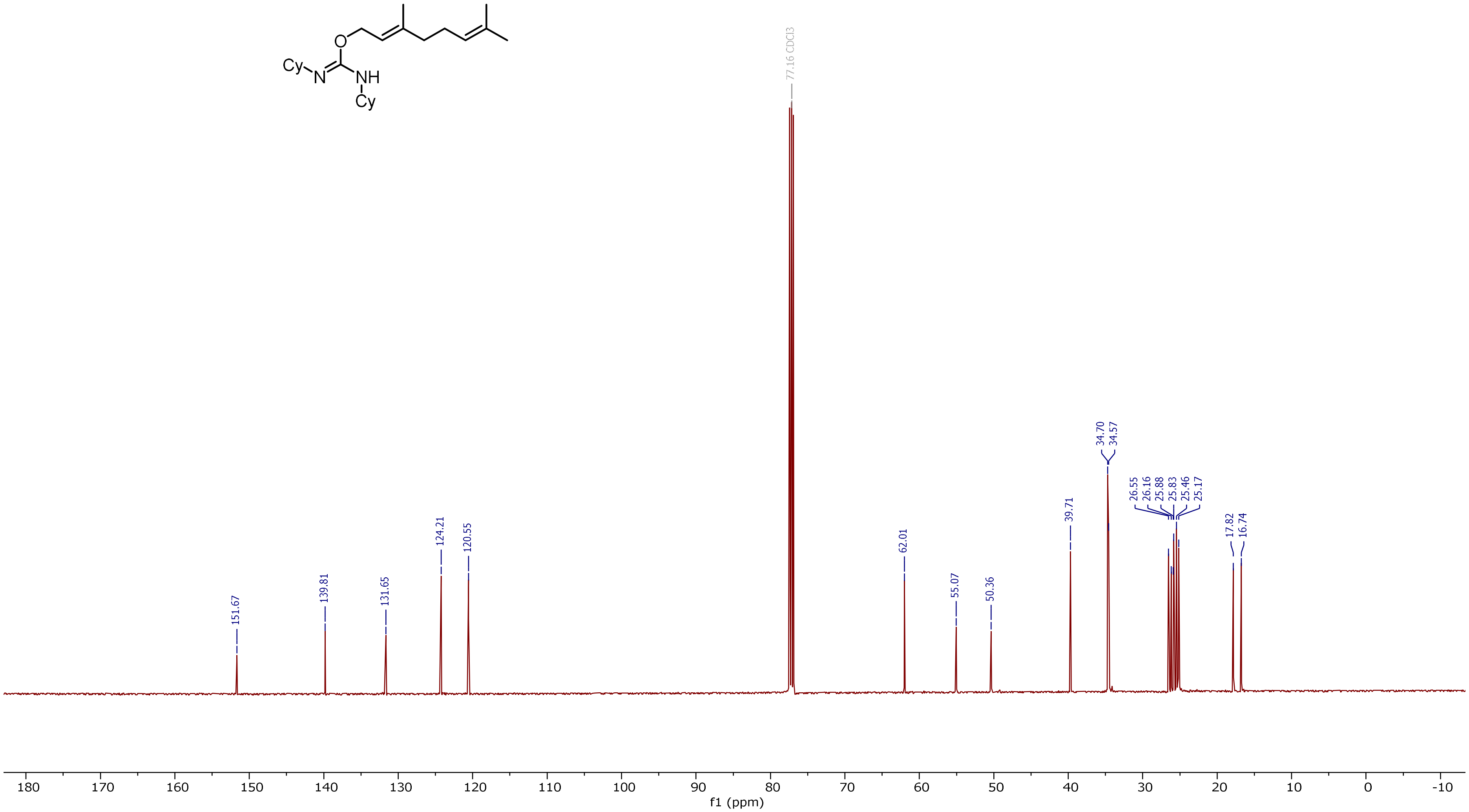

^{13}C NMR (126 MHz, $CDCl_3$) spectrum of O-(2-(E)-3,7-dimethyl-octa-2,6-dienyl)-*N*,*N′*-dicyclohexylisourea.

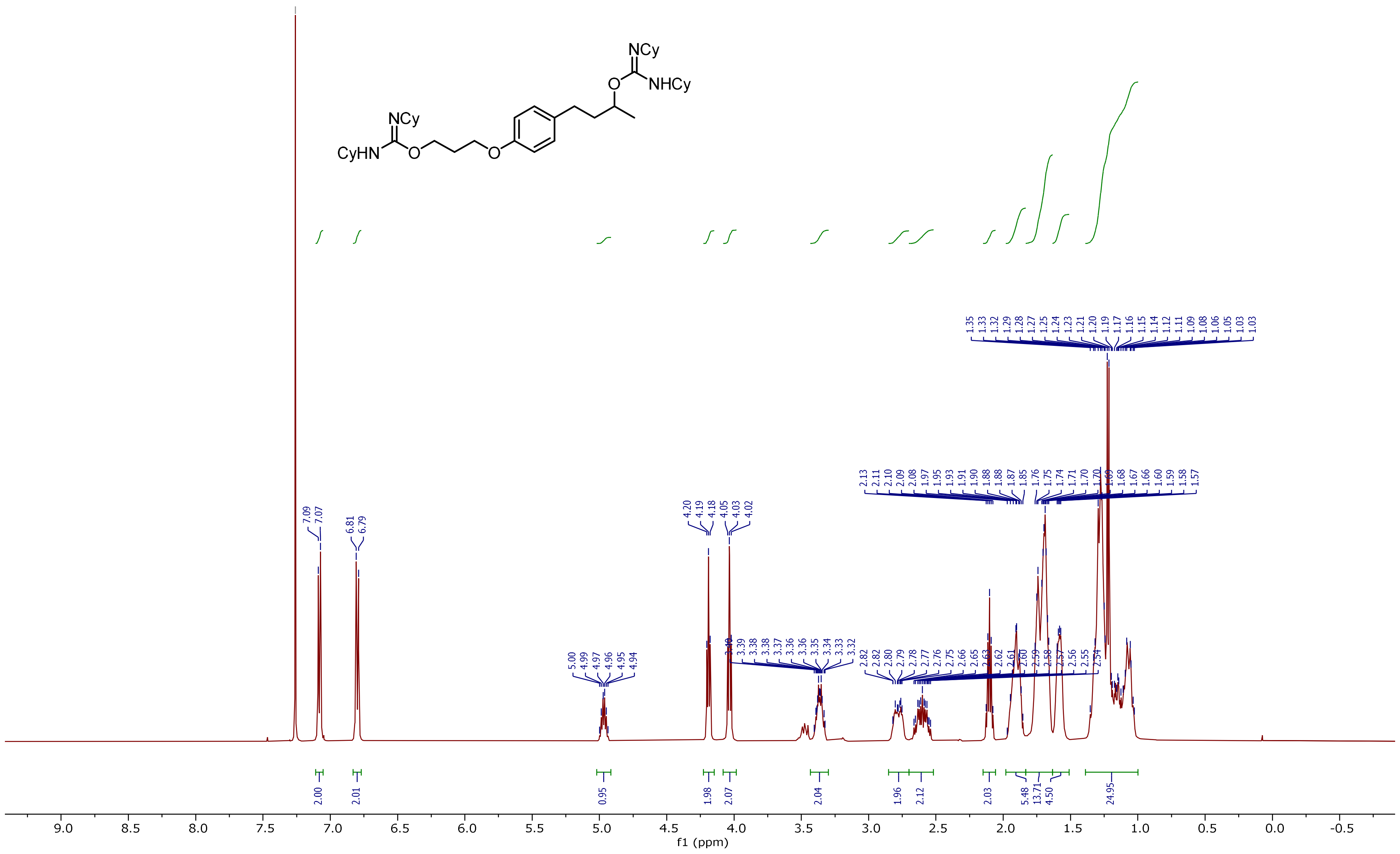

^{1}H NMR (500 MHz, CDCl3) spectrum of 4-(4-(3-*O*-(*N*,*N'*-dicyclohdexylcarbamimidyl)-propoxy)phenyl)butan-2-*O*-*N*,*N'*-dicyclohexylcarbamimidate.

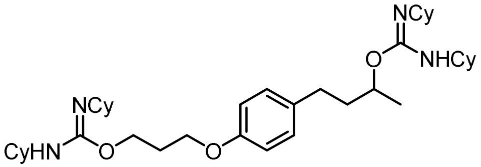

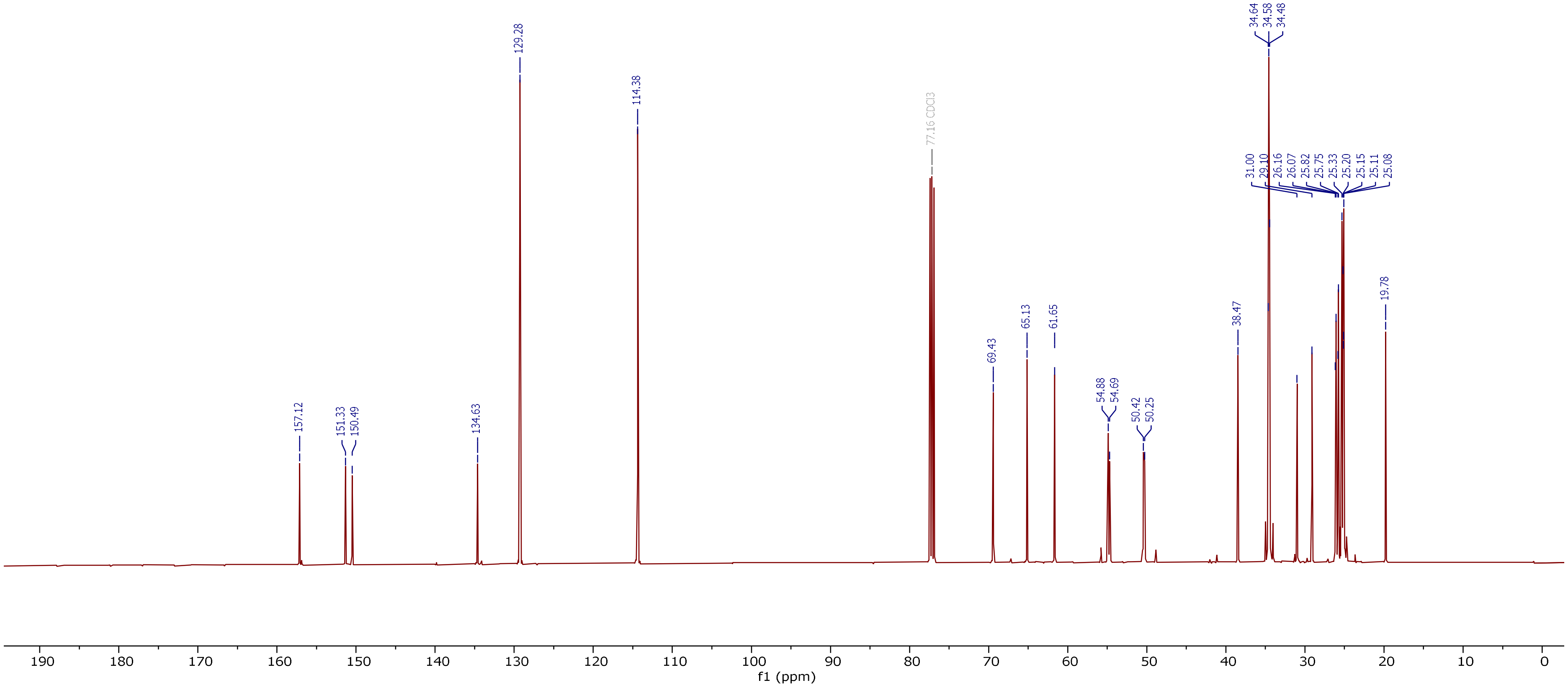

^{13}C NMR (151 MHz, $CDCl_3$) spectrum of 4-(4-(3-*O*-(*N*,*N′*-dicyclohdexylcarbamimidyl)-propoxy)phenyl)butan-2-*O*-*N*,*N′*-dicyclohexylcarbamimidate.

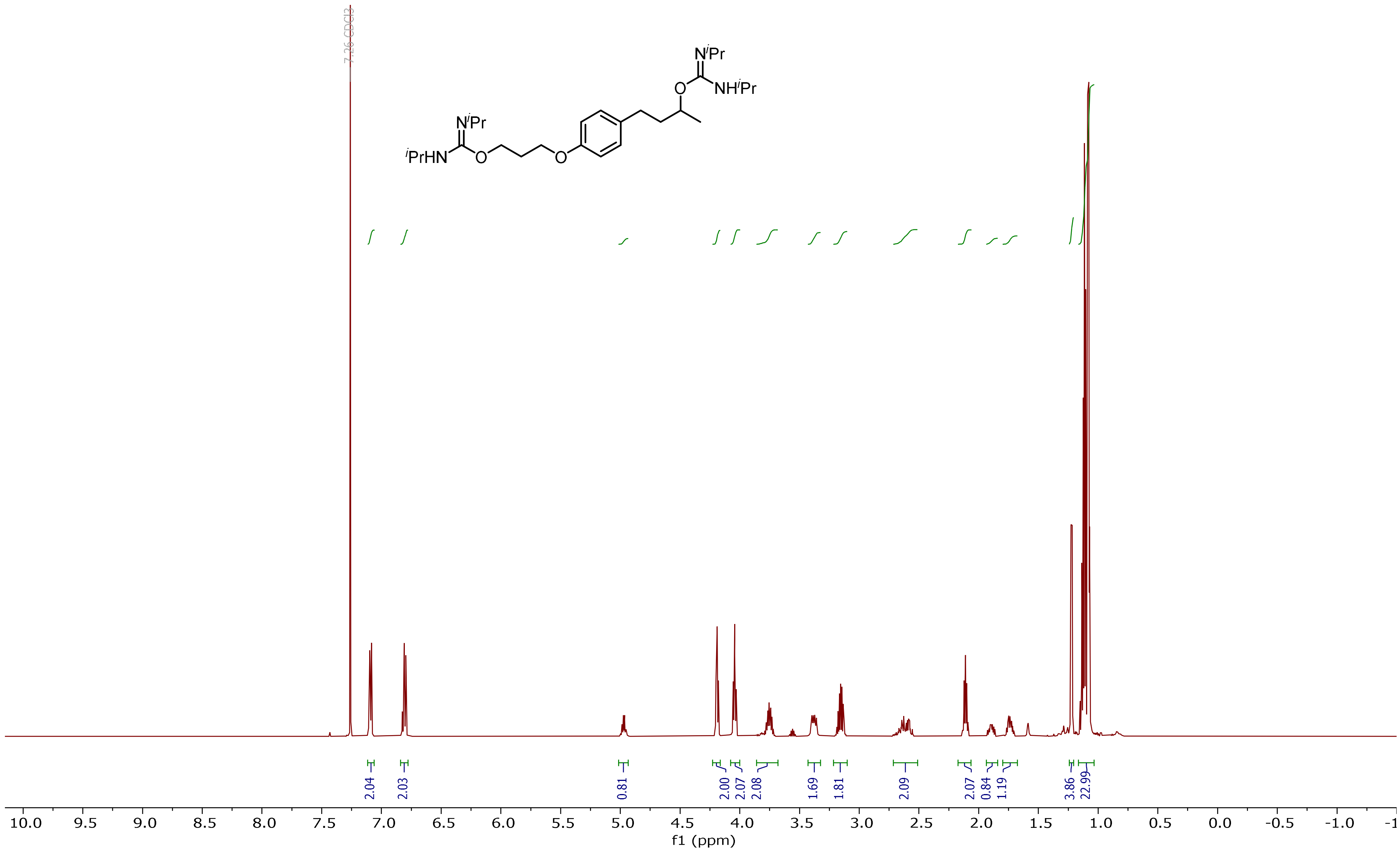

^{1}H NMR (500 MHz, $CDCl_3$) spectrum of 4-(4-(3-*O*-(*N*,*N'*-di*iso*propylcarbamimidyl)-propoxy)phenyl)butan-2-*O*-*N*,*N'*-di*iso*propylcarbamimidate.

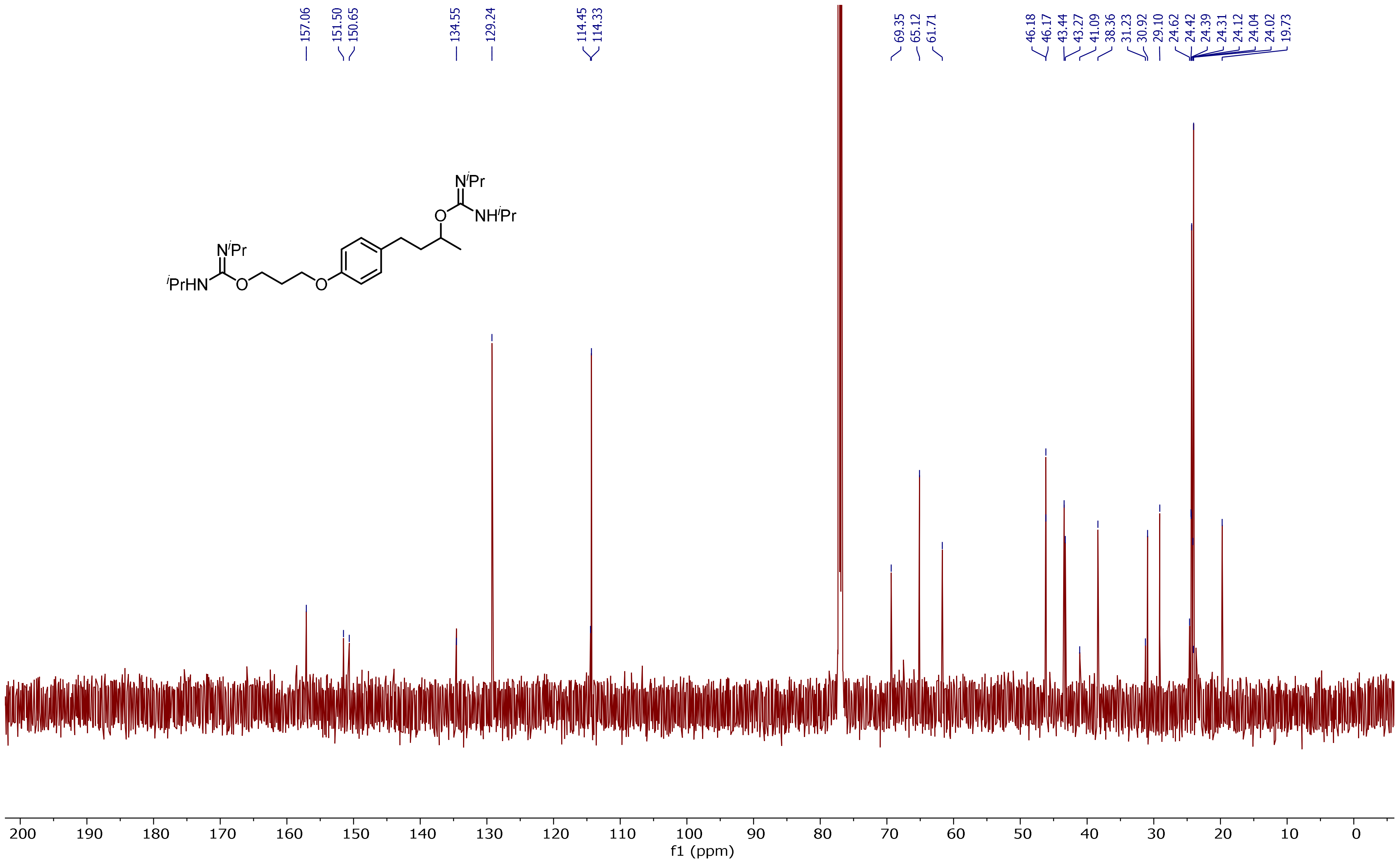

^{13}C NMR (126 MHz, $CDCl_3$) spectrum of 4-(4-(3-*O*-(*N*,*N'*-di*iso*propylcarbamimidyl)-propoxy)phenyl)butan-2-*O*-*N*,*N'*-di*iso*propylcarbamimidate.

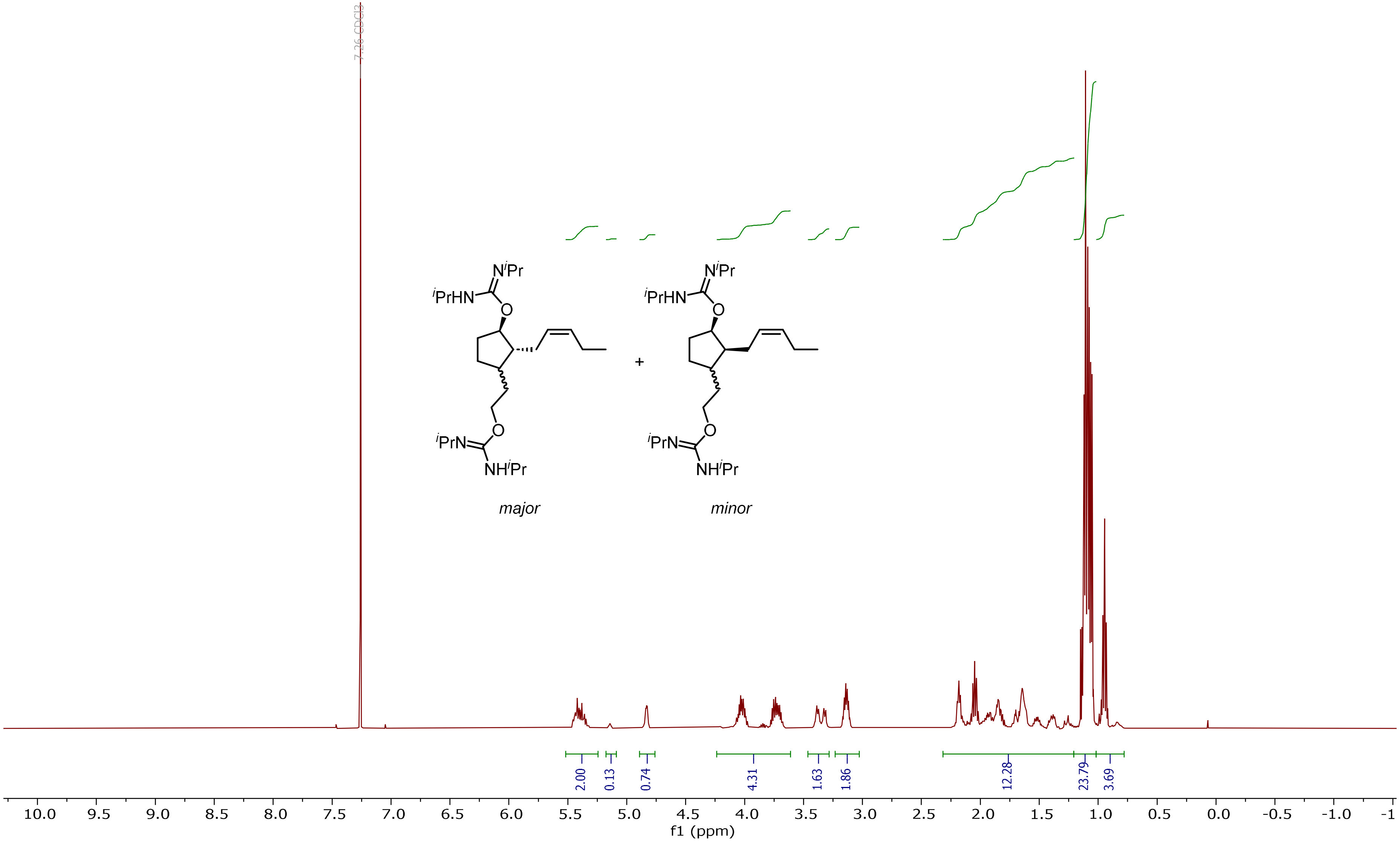

^{1}H NMR (500 MHz, $CDCl_3$) spectrum of (±)-3-(2-*O*-(*N*,*N'*-di*iso*propylcarbamimidyl)-ethyl)-2-((*Z*)-pent-2-enyl)-cyclopentan-1-*O*-*N*,*N'*-di*iso*propylcarbamimidate.

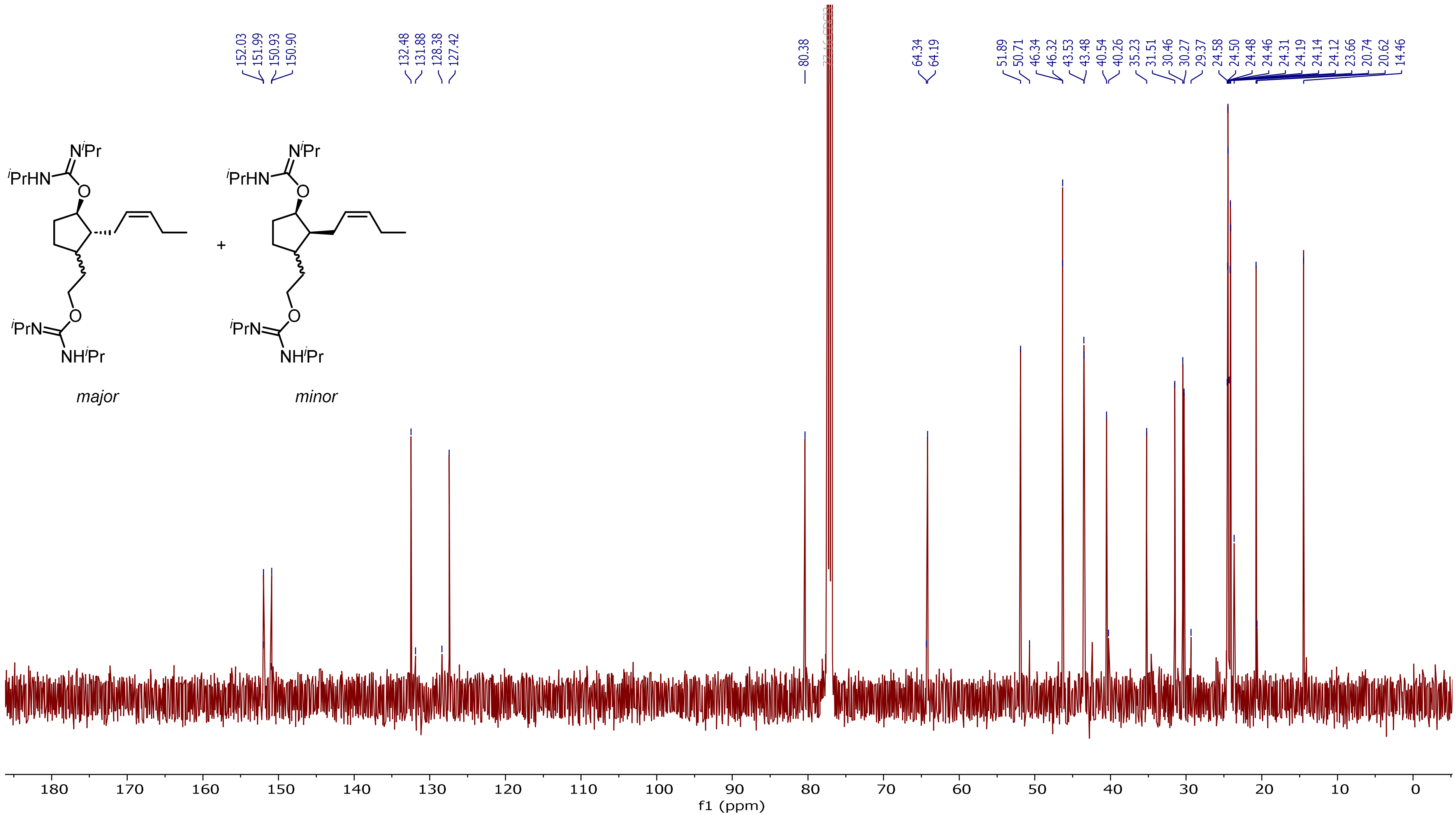

^{13}C NMR (126 MHz, $CDCl_3$) spectrum of (±)-3-(2-*O*-(*N*,*N'*-di*iso*propylcarbamimidyl)-ethyl)-2-((*Z*)-pent-2-enyl)-cyclopentan-1-*O*-*N*,*N'*-di*iso*propylcarbamimidate.

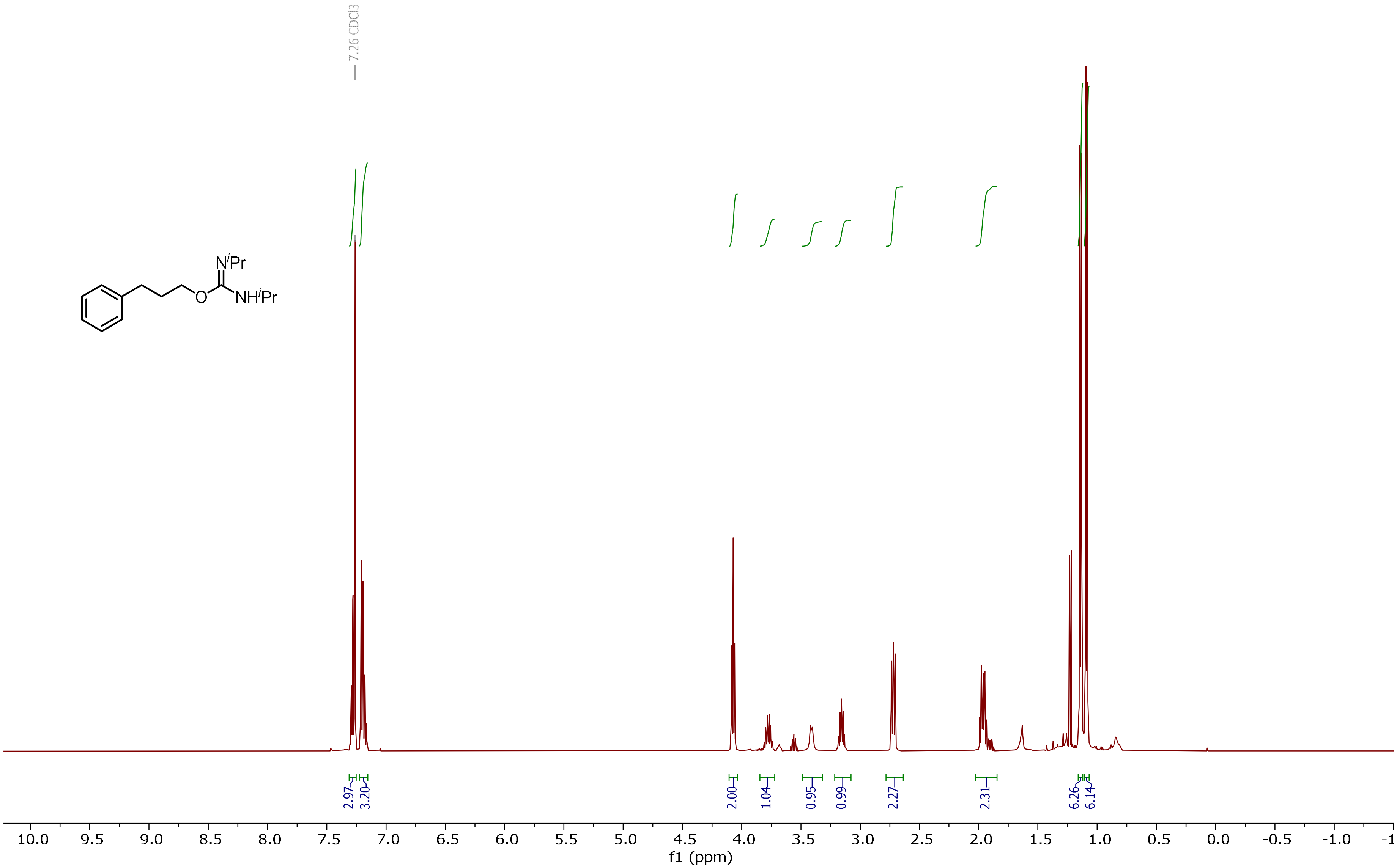

^{1}H NMR (500 MHz, $CDCl_3$) spectrum of *O*-(1-(3-phenyl)propyl)-*N*,*N'*-di*iso*propylisourea.

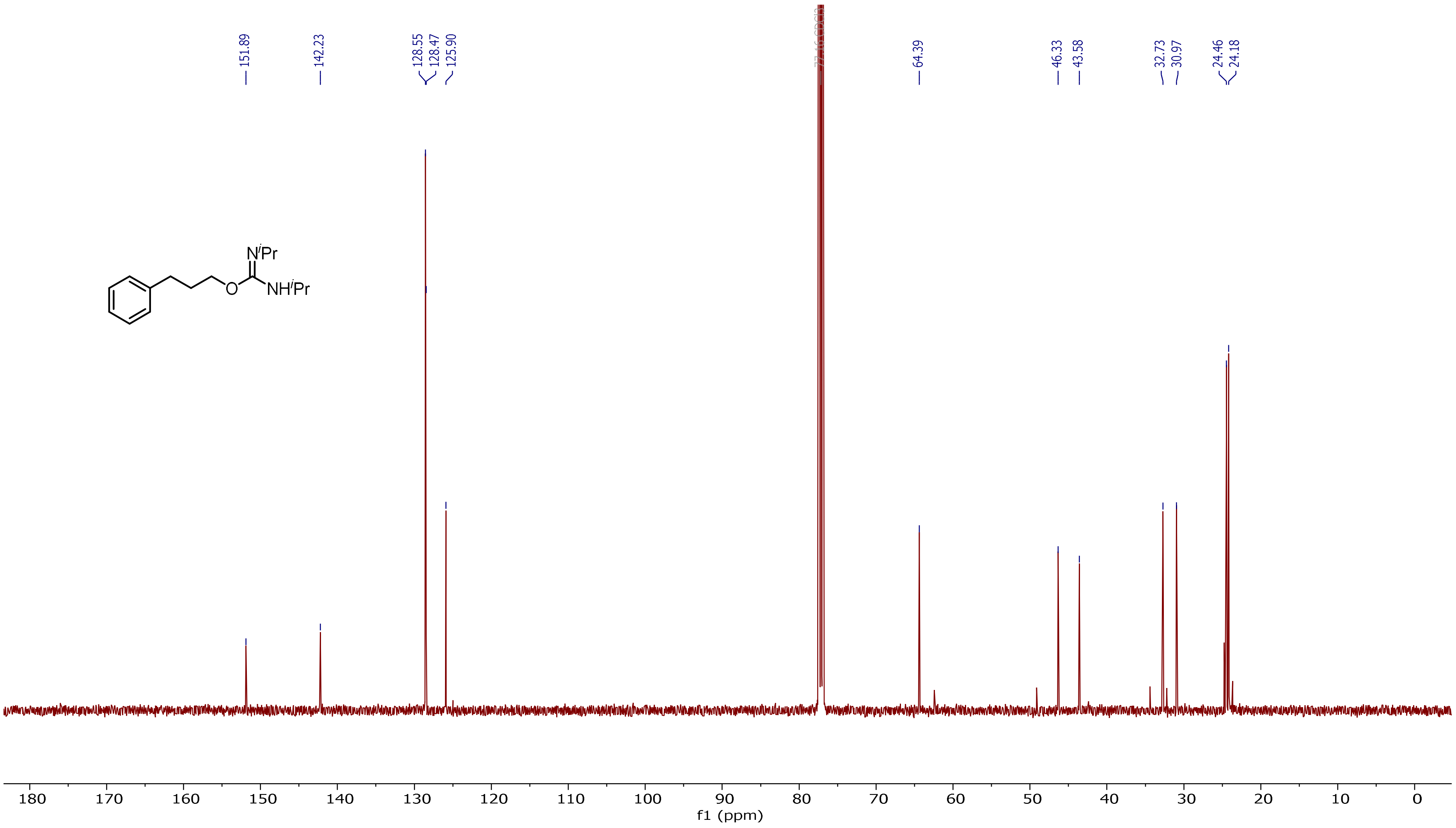

^{13}C NMR (126 MHz, $CDCl_3$) spectrum of *O*-(1-(3-phenyl)propyl)-*N*,*N'*-di*iso*propylisourea.

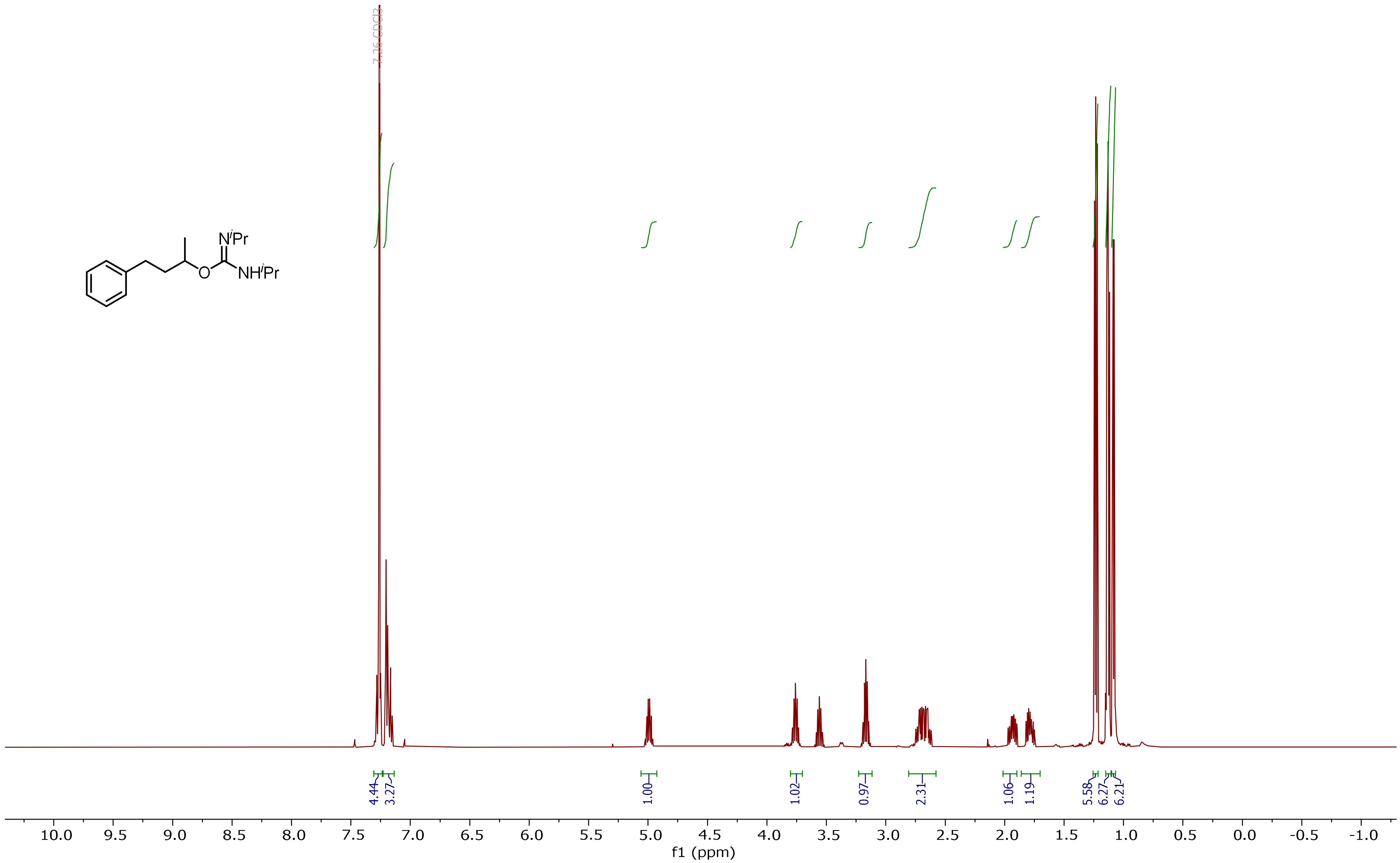

^{1}H NMR (500 MHz, $CDCl_3$) spectrum of (±)-*O*-(2-(4-phenyl)butyl)-*N*,*N′*-di*iso*propylisourea.

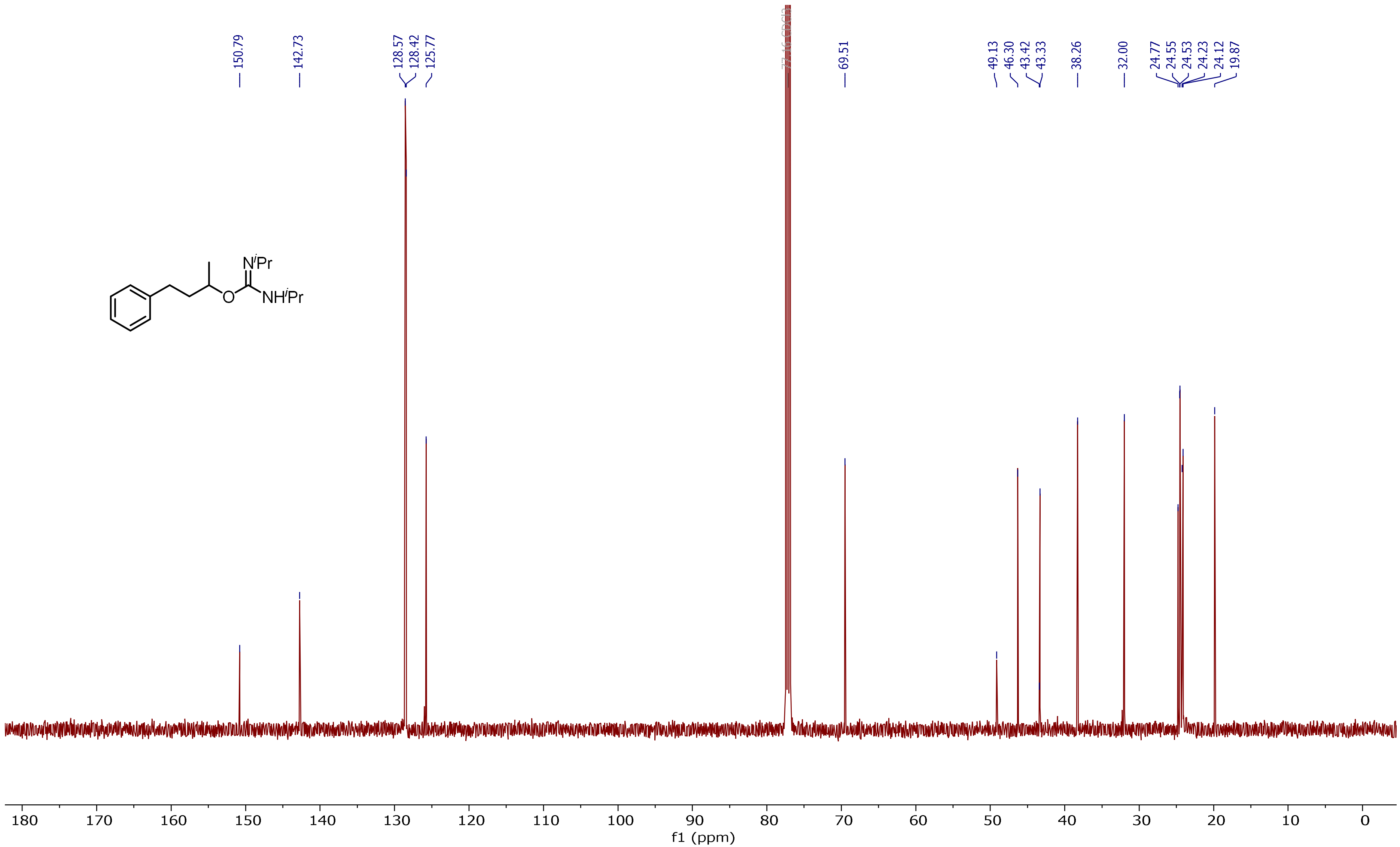

^{13}C NMR (126 MHz, $CDCl_3$) spectrum of (±)-*O*-(2-(4-phenyl)butyl)-*N*,*N'*-di*iso*propylisourea.

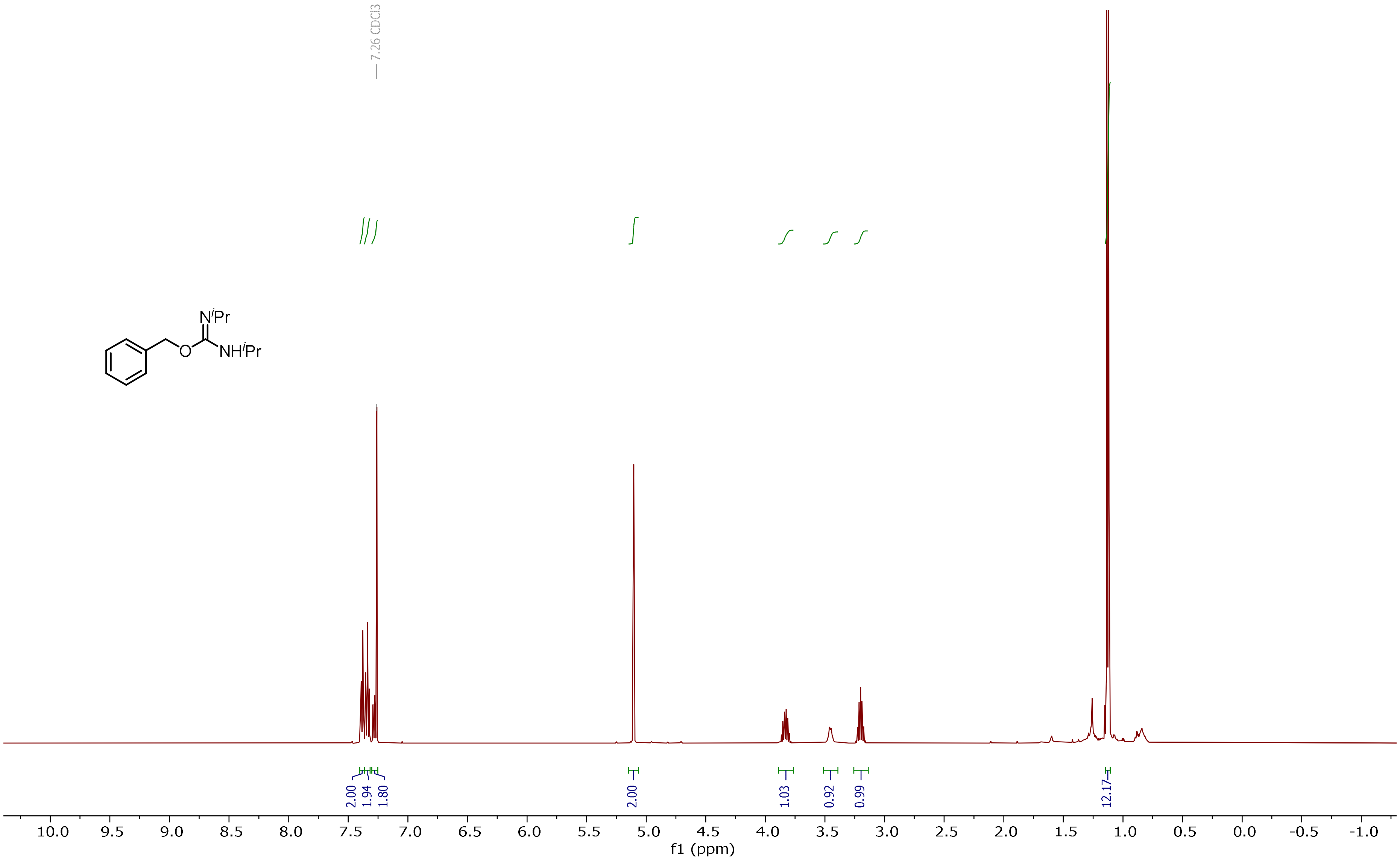

^{1}H NMR (500 MHz, $CDCl_3$) spectrum of *O*-benzyl-*N*,*N'*-di*iso*propylisourea.

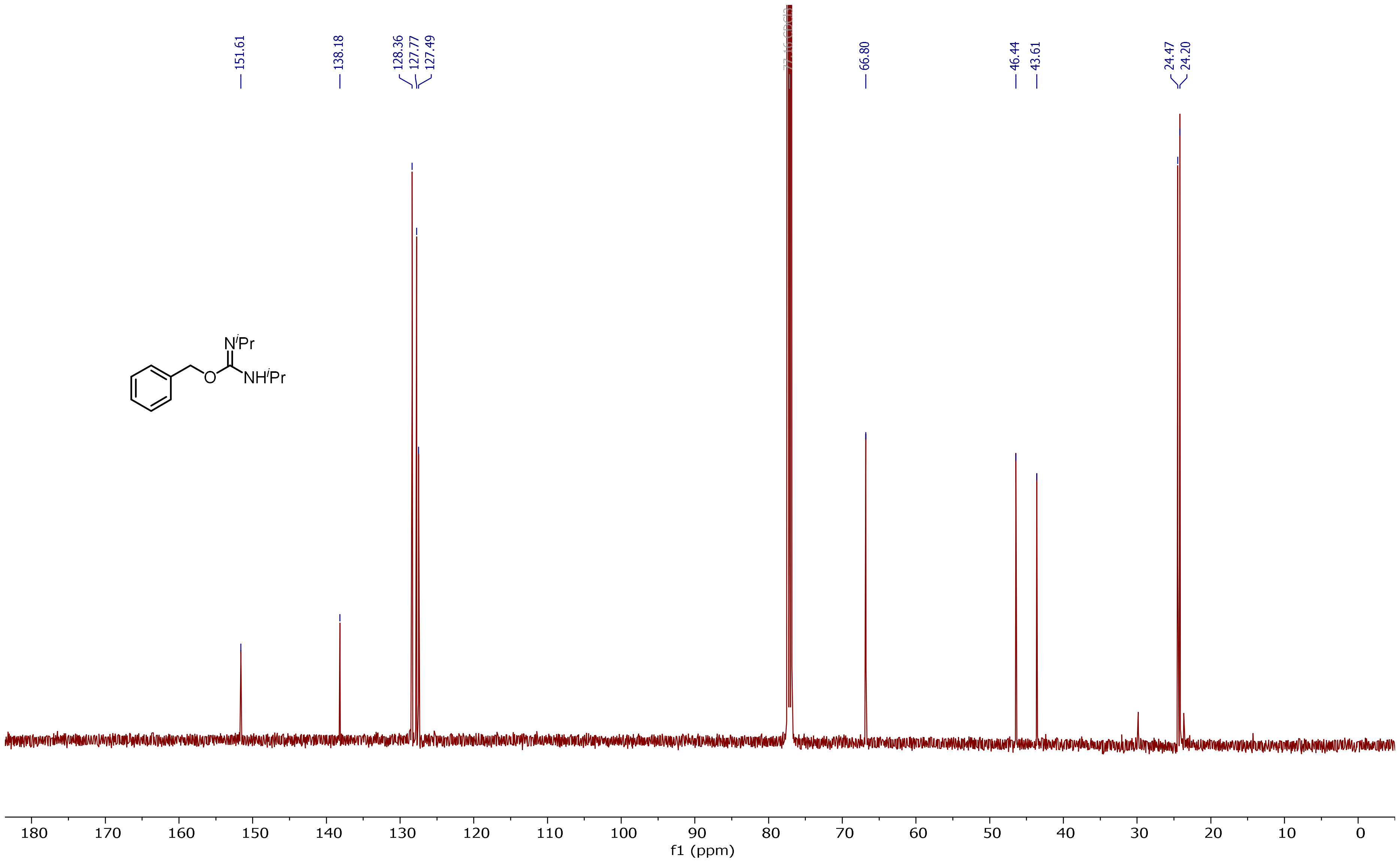

^{13}C NMR (126 MHz, $CDCl_3$) spectrum of *O*-benzyl-*N*,*N'*-di*iso*propylisourea.

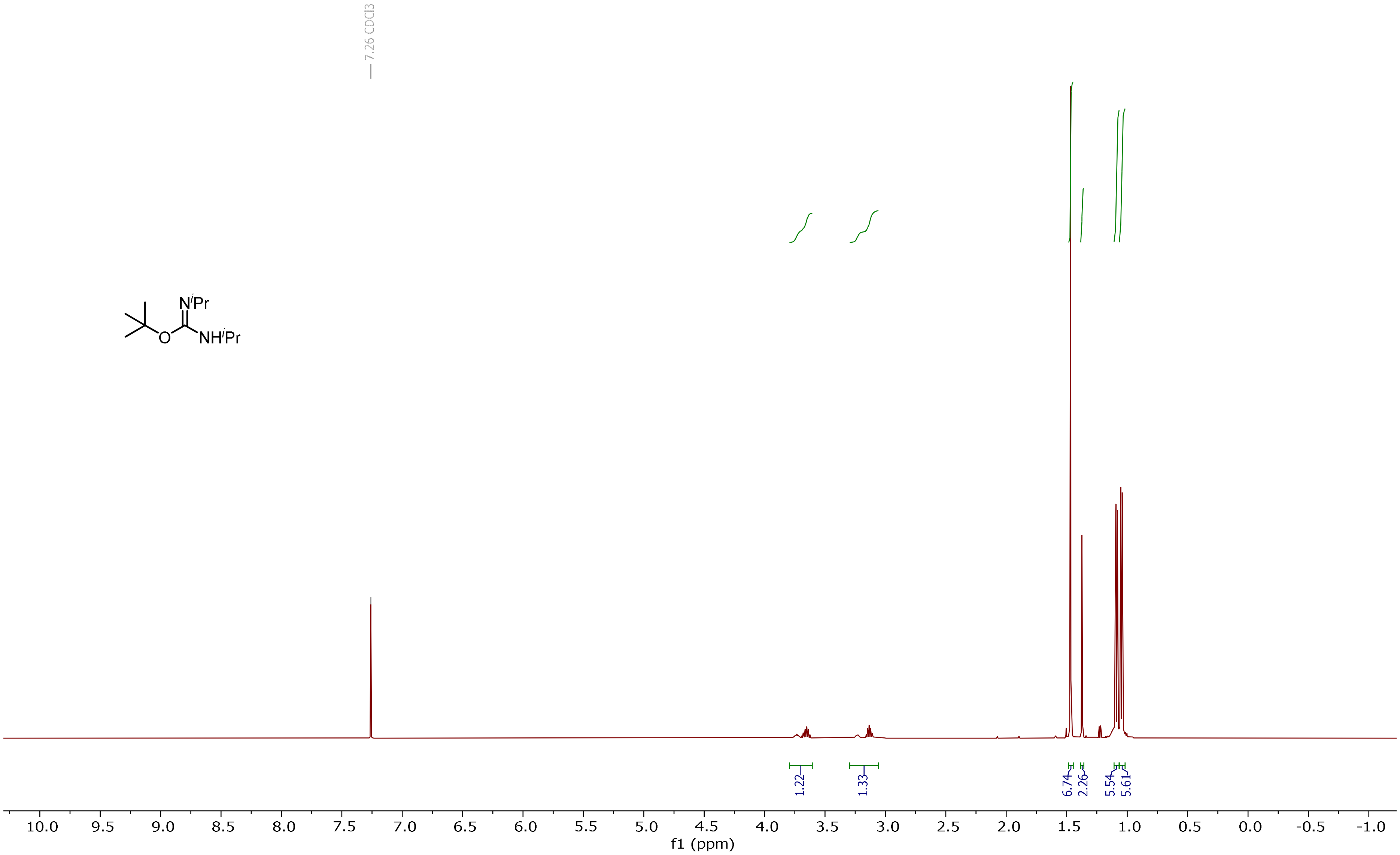

^{1}H NMR (500 MHz, $CDCl_3$) spectrum of *O*-(*tert*-butyl)-*N*,*N'*-di*iso*propylisourea.

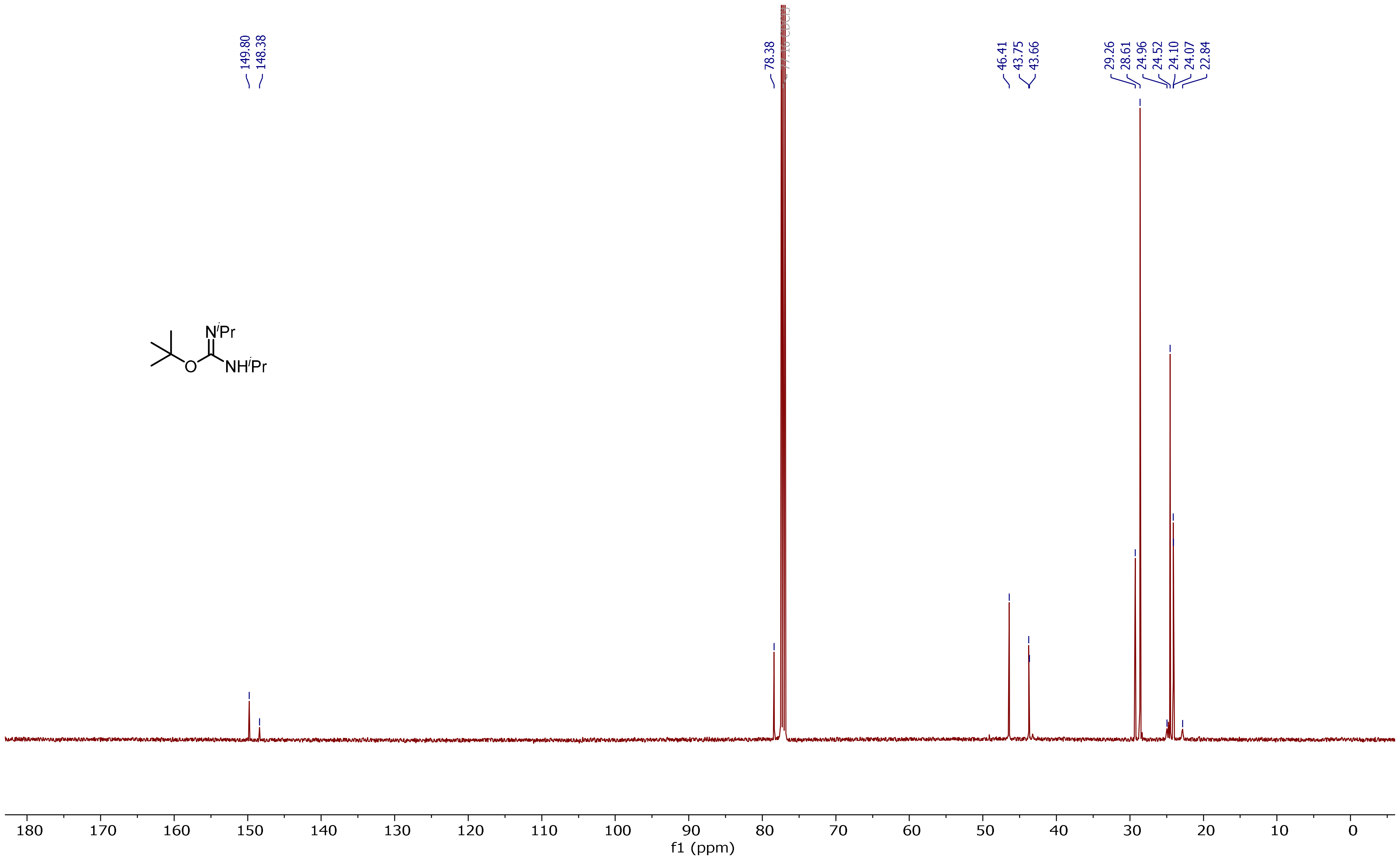

^{13}C NMR (126 MHz, $CDCl_3$) spectrum of *O*-(*tert*-butyl)-*N*,*N′*-di*iso*propylisourea.

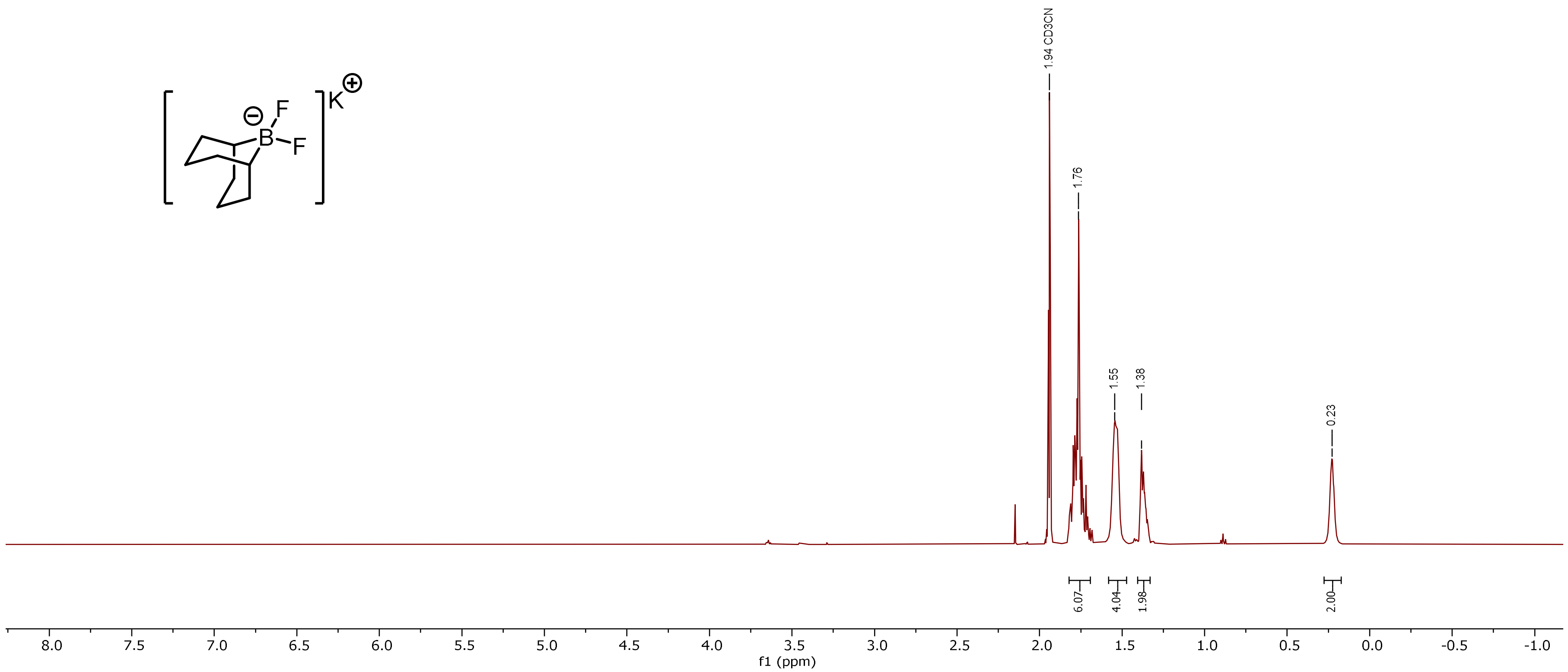

^{1}H NMR (500 MHz, CD_3CN) spectrum of potassium [9,9-difluoro-9-borabicyclo-[3.3.1]-nonanate].

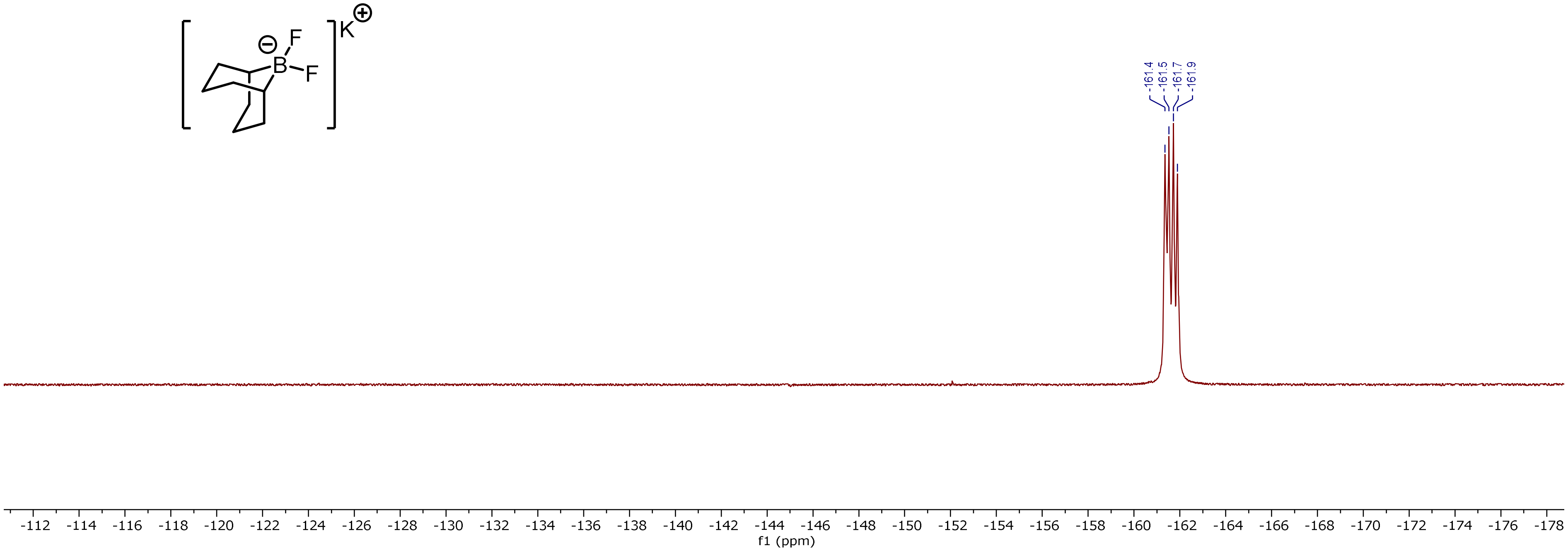

^{19}F NMR (471 MHz, CD_3CN) spectrum of potassium [9,9-difluoro-9-borabicyclo-[3.3.1]-nonanate].

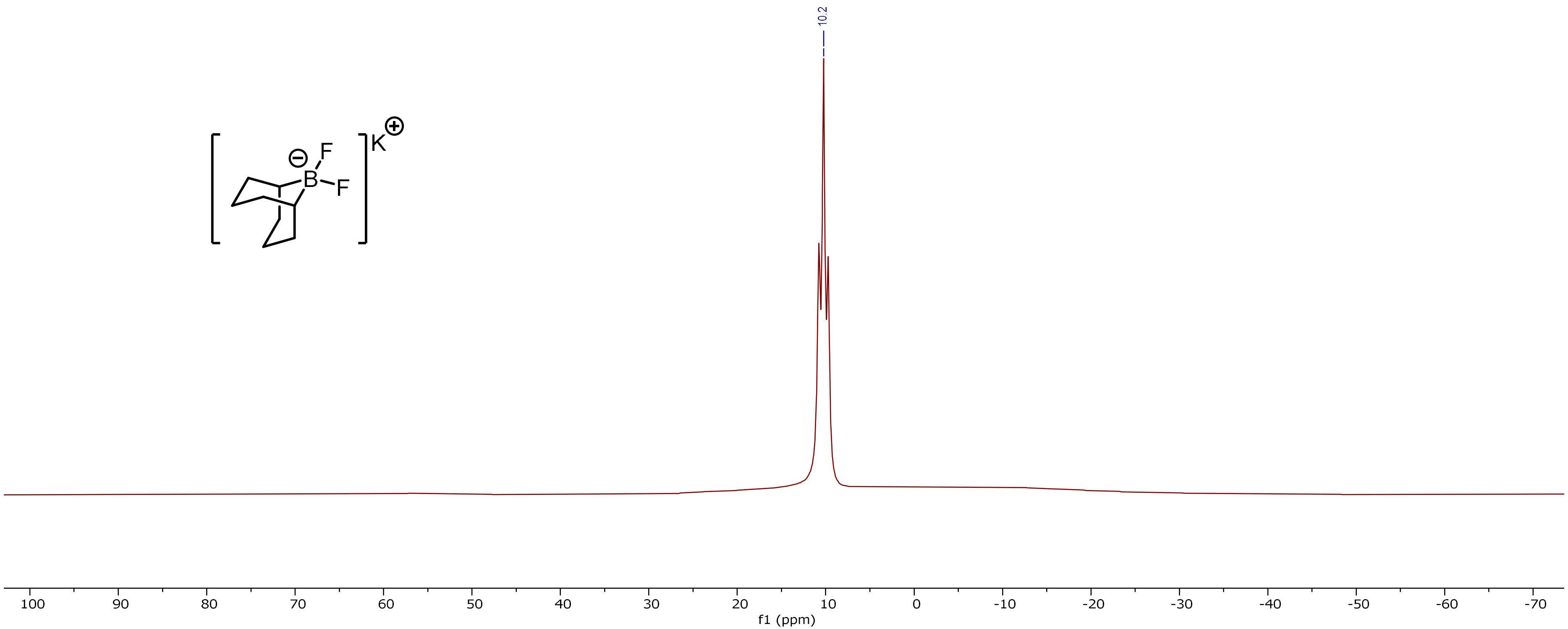

^{11}B NMR (128 MHz, CD_3CN) spectrum of potassium [9,9-difluoro-9-borabicyclo-[3.3.1]-nonanate].

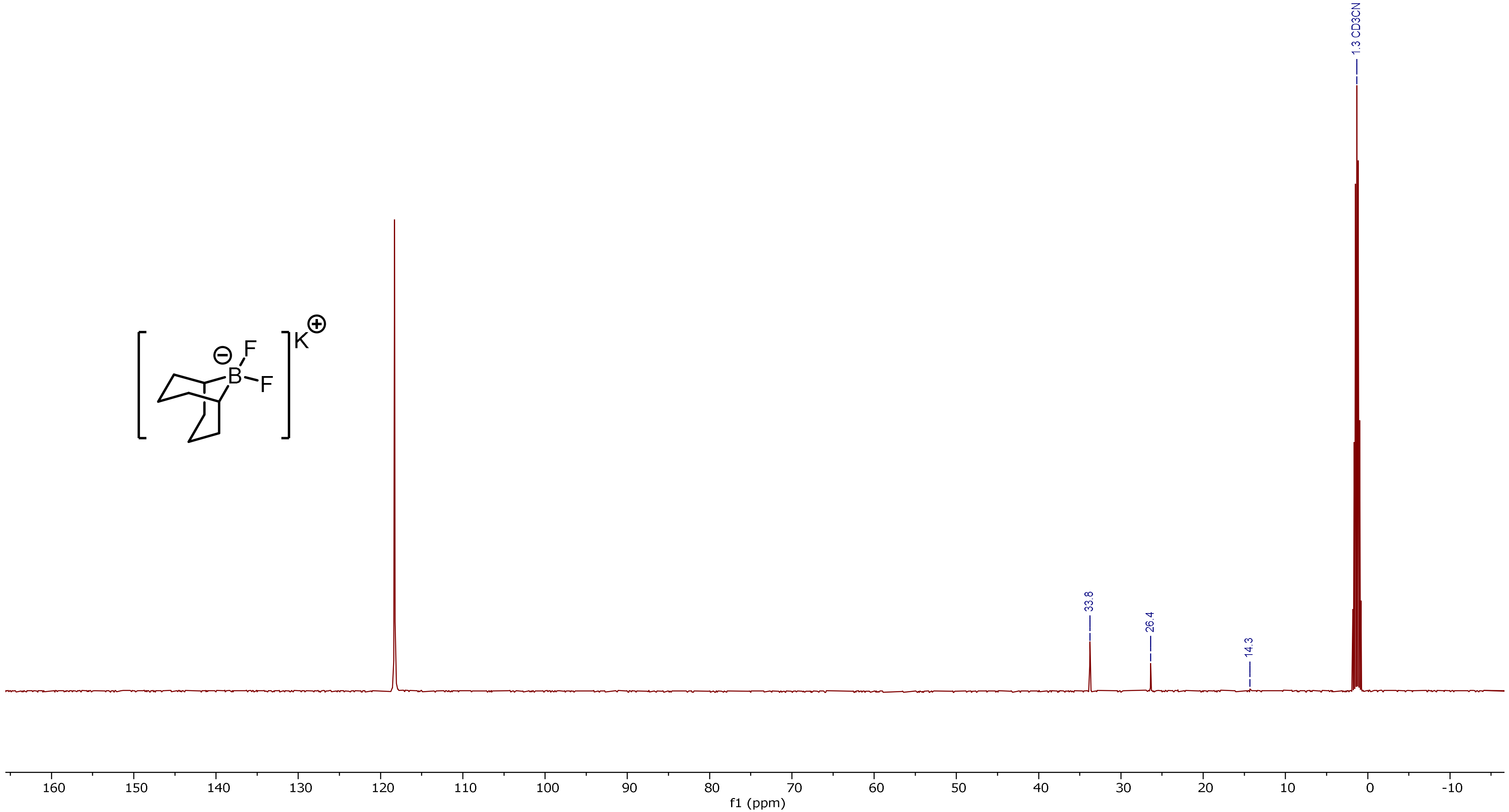

^{13}C NMR (126 MHz, CD_3CN) spectrum of potassium [9,9-difluoro-9-borabicyclo-[3.3.1]-nonanate].

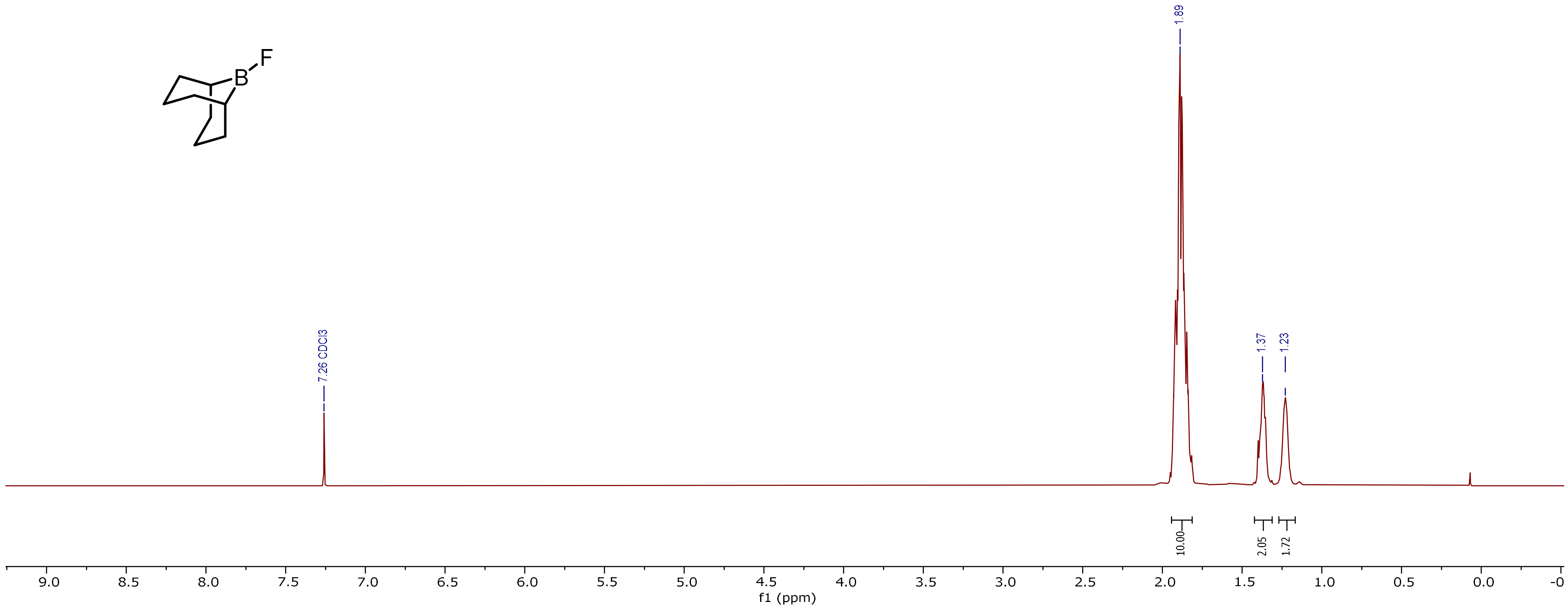

^{1}H NMR (500 MHz, $CDCl_3$) spectrum of 9-fluoro-9-borabicyclo-[3.3.1]-nonane.

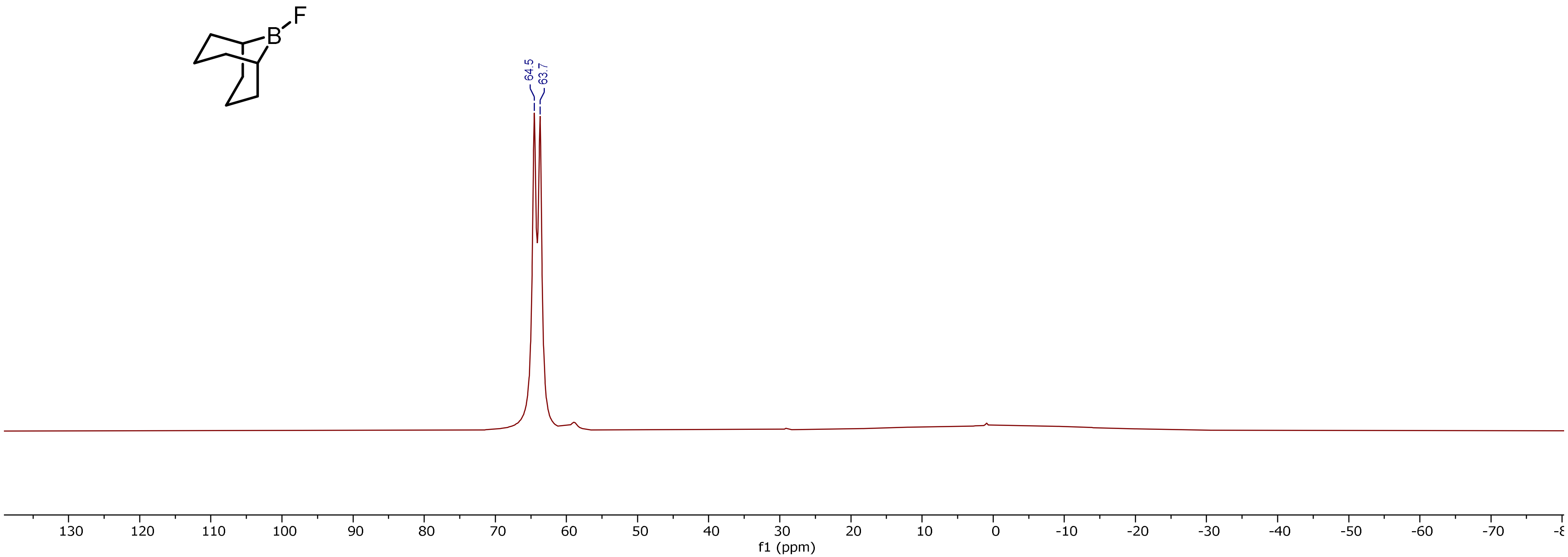

^{11}B NMR (128 MHz, $CDCl_3$) spectrum of 9-fluoro-9-borabicyclo-[3.3.1]-nonane.

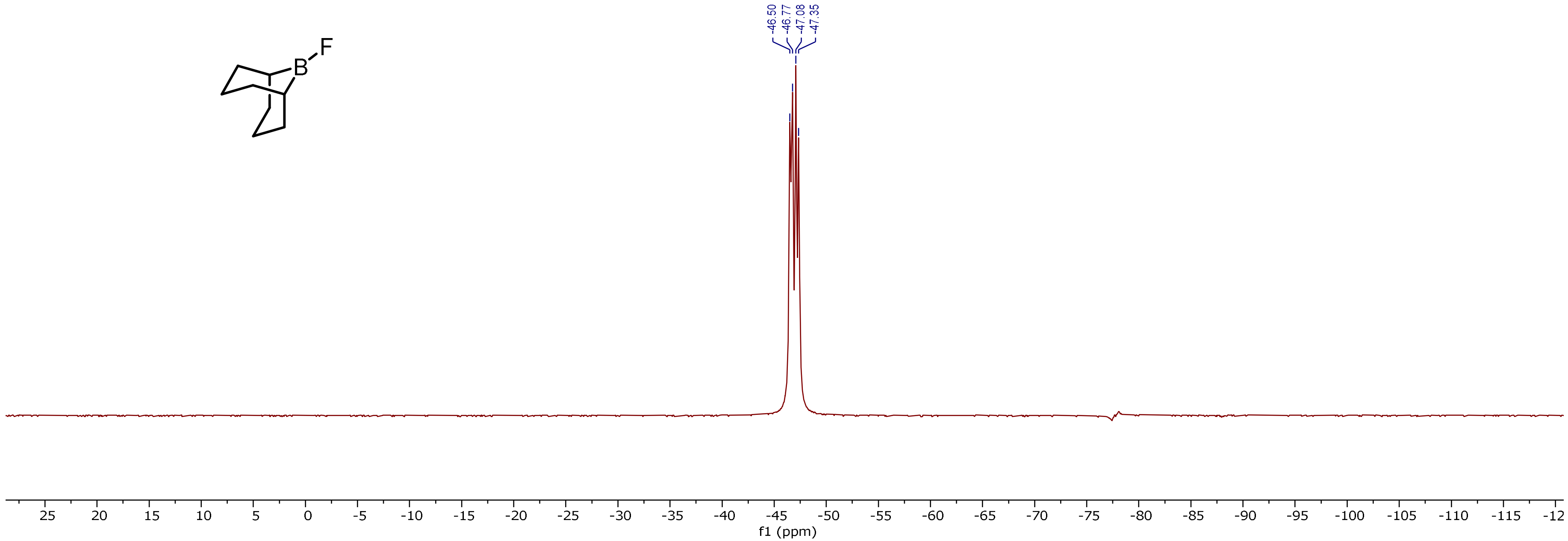

^{19}F NMR (471 MHz, $CDCl_3$) spectrum of 9-fluoro-9-borabicyclo-[3.3.1]-nonane.

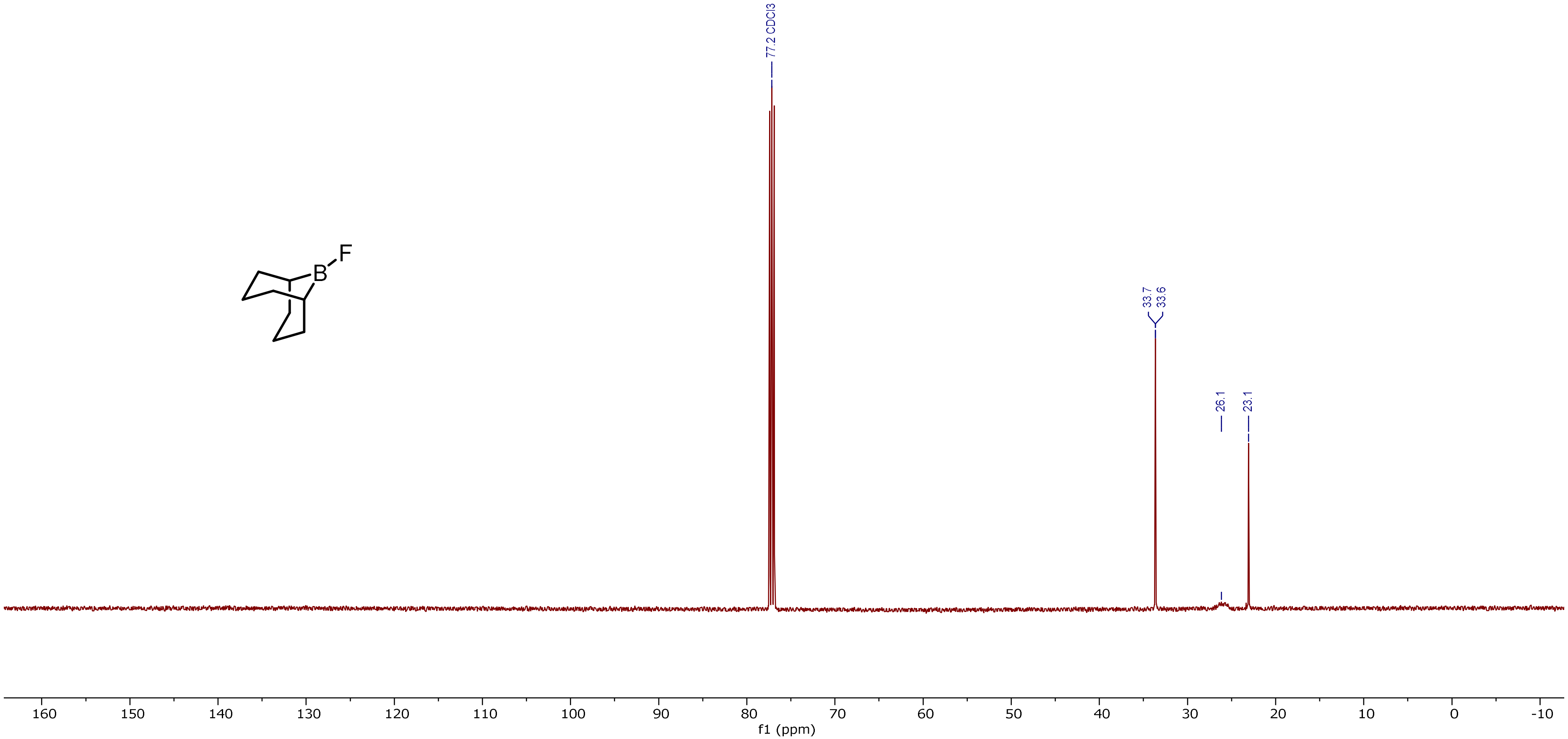

^{13}C NMR (126 MHz, $CDCl_3$) spectrum of 9-fluoro-9-borabicyclo-[3.3.1]-nonane.

17. References

[1] J. A. Soderquist, I. Kock, M. E. Estrella, *Org. Process Res. Dev.* **2006**, *10*, 1076–1079.
[2] F. J. Weigert, *J. Org. Chem.* **1980**, *45*, 3476–3483.
[3] N. R. Patel, R. A. I. Flowers, *J. Org. Chem.* **2015**, *80*, 5834–5841.
[4] M. González-Esguevillas, J. Miró, J. L. Jeffrey, D. W. C. MacMillan, *Tetrahedron* **2019**, *75*, 4222–4227.
[5] G. H. Lovett, S. Chen, X.-S. Xue, K. N. Houk, D. W. C. MacMillan, *J. Am. Chem. Soc.* **2019**, *141*, 20031–20036.
[6] S. Ortalli, J. Ford, A. A. Trabanco, M. Tredwell, V. Gouverneur, *J. Am. Chem. Soc.* **2024**, *146*, 11599–11604.
[7] H. W. Moon, M. N. Lavagnino, S. Lim, M. D. Palkowitz, M. D. Mandler, G. L. Beutner, M. J. Drance, J. M. Lipshultz, P. M. Scola, A. T. Radosevich, *J. Am. Chem. Soc.* **2023**, *145*, 22735–22744.
[8] J.-B. Xia, C. Zhu, C. Chen, *Chem. Commun.* **2014**, *50*, 11701–11704.
[9] J. R. Box, M. E. Avanthay, D. L. Poole, A. J. J. Lennox, *Angew. Chem. Int. Ed.* **2023**, *62*, e202218195.
[10] L.-Y. Ran, X. Ding, X.-P. Yan, C.-P. Zhang, *Org. Biomol. Chem.* **2023**, *21*, 1235–1241.
[11] A. Garg, N. J. Gerwien, C. Fasting, A. Charlton, M. N. Hopkinson, *Angew. Chem. Int. Ed.* **2023**, *62*, e202302860.
[12] P. A. Champagne, Y. Benhassine, J. Desroches, J.-F. Paquin, *Angew. Chem. Int. Ed.* **2014**, *53*, 13835–13839.
[13] C. Houle, P. R. Savoie, C. Davies, D. Jardel, P. A. Champagne, B. Bibal, J.-F. Paquin, *Chem. – Eur. J.* **2020**, *26*, 10620–10625.
[14] M. B. Johansen, A. T. Lindhardt, *Chem. Commun.* **2018**, *54*, 825–828.
[15] A. L’Heureux, F. Beaulieu, C. Bennett, D. R. Bill, S. Clayton, F. LaFlamme, M. Mirmehrabi, S. Tadayon, D. Tovell, M. Couturier, *J. Org. Chem.* **2010**, *75*, 3401–3411.
[16] J. Walkowiak, M. Tomas-Szwaczyk, G. Haufe, H. Koroniak, *J. Fluor. Chem.* **2012**, *143*, 189–197.
[17] D. E. Sood, S. Champion, D. M. Dawson, S. Chabbra, B. E. Bode, A. Sutherland, A. J. B. Watson, *Angew. Chem. Int. Ed.* **2020**, *59*, 8460–8463.
[18] P. Song, S. Zhu, *ACS Catal.* **2020**, *10*, 13165–13170.
[19] D. F. Shellhamer, A. A. Briggs, B. M. Miller, J. M. Prince, D. H. Scott, V. L. Heasley, *J. Chem. Soc. Perkin Trans. 2* **1996**, 973–977.
[20] M. Rueda-Becerril, C. Chatalova Sazepin, J. C. T. Leung, T. Okbinoglu, P. Kennepohl, J.-F. Paquin, G. M. Sammis, *J. Am. Chem. Soc.* **2012**, *134*, 4026–4029.
[21] M. K. Nielsen, C. R. Ugaz, W. Li, A. G. Doyle, *J. Am. Chem. Soc.* **2015**, *137*, 9571–9574.
[22] A. Y. Jordan, T. Y. Meyer, *J. Organomet. Chem.* **1999**, *591*, 104–113.
[23] Z. Zhang, F. Wang, X. Mu, P. Chen, G. Liu, *Angew. Chem. Int. Ed.* **2013**, *52*, 7549–7553.
[24] X. Xia, P. H. Toy, *Beilstein J. Org. Chem.* **2014**, *10*, 1397–1405.
[25] P. V. Ramachandran, A. A. Alawaed, H. J. Hamann, *Org. Lett.* **2023**, *25*, 4650–4655.
[26] L. Li, C. Ni, F. Wang, J. Hu, *Nat. Commun.* **2016**, *7*, 13320.
[27] M. D. R. Lutz, V. C. M. Gasser, B. Morandi, *Chem* **2021**, *7*, 1108–1119.
[28] J. Guo, C. Kuang, J. Rong, L. Li, C. Ni, J. Hu, *Chem. – Eur. J.* **2019**, *25*, 7259–7264.
[29] C. Schneider, J. Brauner, *Eur. J. Org. Chem.* **2001**, *2001*, 4445–4450.
[30] P. V. Ramachandran, A. A. Alawaed, H. J. Hamann, *J. Org. Chem.* **2022**, *87*, 13259–13269.
[31] Y. Yu, D. Zhai, Z. Zhou, S. Jiang, H. Qian, S. Ma, *Chem. Commun.* **2023**, *59*, 5281–5284.
[32] H. Nonaka, N. Ogawa, N. Maeda, Y.-G. Wang, Y. Kobayashi, *Org. Biomol. Chem.* **2010**, *8*, 5212–5223.
[33] J. Pícha, M. Buděšínský, I. Hančlová, M. Šanda, P. Fiedler, V. Vaněk, J. Jiráček, *Tetrahedron* **2009**, *65*, 6090–6103.

[34] L. A. Wells, M. R. Gau, M. B. Peddicord, K. F. Bostwick, M. C. Kozlowski, M. J. Zacuto, *Org. Process Res. Dev.* **2022**, *26*, 1803–1811.
[35] D. R. Willcox, G. S. Nichol, S. P. Thomas, *ACS Catal.* **2021**, *11*, 3190–3197.
[36] M. J. Frisch, G. W. Trucks, H. B. Schlegel, G. E. Scuseria, M. A. Robb, J. R. Cheeseman, G. Scalmani, V. Barone, G. A. Petersson, H. Nakatsuji, X. Li, M. Caricato, A. V. Marenich, J. Bloino, B. G. Janesko, R. Gomperts, B. Mennucci, H. P. Hratchian, J. V. Ortiz, A. F. Izmaylov, J. L. Sonnenberg, Williams, F. Ding, F. Lipparini, F. Egidi, J. Goings, B. Peng, A. Petrone, T. Henderson, D. Ranasinghe, V. G. Zakrzewski, J. Gao, N. Rega, G. Zheng, W. Liang, M. Hada, M. Ehara, K. Toyota, R. Fukuda, J. Hasegawa, M. Ishida, T. Nakajima, Y. Honda, O. Kitao, H. Nakai, T. Vreven, K. Throssell, J. A. Montgomery Jr., J. E. Peralta, F. Ogliaro, M. J. Bearpark, J. J. Heyd, E. N. Brothers, K. N. Kudin, V. N. Staroverov, T. A. Keith, R. Kobayashi, J. Normand, K. Raghavachari, A. P. Rendell, J. C. Burant, S. S. Iyengar, J. Tomasi, M. Cossi, J. M. Millam, M. Klene, C. Adamo, R. Cammi, J. W. Ochterski, R. L. Martin, K. Morokuma, O. Farkas, J. B. Foresman, D. J. Fox, Gaussian 16 Rev. C.01, **2016**.
[37] Chemcraft - Graphical Software for Visualization of Quantum Chemistry Computations. Version 1.8, Build 682. Https://www.chemcraftprog.com, **2024**.
[38] T. A. Young, J. J. Silcock, A. J. Sterling, F. Duarte, *Angew. Chem. Int. Ed.* **2021**, *60*, 4266–4274.
[39] P. Pracht, F. Bohle, S. Grimme, *Phys. Chem. Chem. Phys.* **2020**, *22*, 7169–7192.
[40] C. Bannwarth, S. Ehlert, S. Grimme, *J. Chem. Theory Comput.* **2019**, *15*, 1652–1671.
[41] Y. Zhao, D. G. Truhlar, *Theor. Chem. Acc.* **2008**, *120*, 215–241.
[42] F. Weigend, R. Ahlrichs, *Phys. Chem. Chem. Phys.* **2005**, *7*, 3297–3305.
[43] S. Grimme, J. Antony, S. Ehrlich, H. Krieg, *J. Chem. Phys.* **2010**, *132*, 154104.
[44] H. S. Yu, X. He, S. L. Li, D. G. Truhlar, *Chem. Sci.* **2016**, *7*, 5032–5051.
[45] F. M. Bickelhaupt, K. N. Houk, *Angew. Chem. Int. Ed.* **2017**, *56*, 10070–10086.
[46] S. Kirschner, M. Peters, K. Yuan, M. Uzelac, M. J. Ingleson, *Chem. Sci.* **2022**, *13*, 2661–2668.
[47] P. Erdmann, J. Leitner, J. Schwarz, L. Greb, *ChemPhysChem* **2020**, *21*, 987–994.
[48] G. M. Sheldrick, *Acta Crystallogr. Sect. Found. Adv.* **2015**, *71*, 3–8.
[49] O. V. Dolomanov, L. J. Bourhis, R. J. Gildea, J. a. K. Howard, H. Puschmann, *J. Appl. Crystallogr.* **2009**, *42*, 339–341.
[50] G. M. Sheldrick, *Acta Crystallogr. Sect. C Struct. Chem.* **2015**, *71*, 3–8.
[51] CrysAlisPro, Rigaku, V1.171.42.81a, **2023**.
